# Will the “Prosperity of the Population and Son” Affect the Hiring of the “Shopkeeper”?—Research on Succession Decisions of Actual Controllers in Chinese Family Firms

**DOI:** 10.3389/fpsyg.2022.825223

**Published:** 2022-05-13

**Authors:** Xiaofei Shi, Zhangjing Tui, Xiaoyu Zhou

**Affiliations:** School of Business Administration, Hebei University of Economics and Business, Shijiazhuang, China

**Keywords:** family firms, actual controller, characteristics of children, professional management, succession decision

## Abstract

“The prosperity of the population and son, the prosperity of the country” means that the more family members and male offspring, the more stable and prosperous the family foundation of future generations. This concept is deeply engraved in China’s traditional culture, and affects the behavior and decision-making of the actual controller as a parent. Chinese family enterprises are facing urgent problems such as power transfer and leader replacement. Although some studies have paid attention to the relationship between children’s characteristics and enterprise inheritance, they have not connected this relationship with the internal mechanism of successor selection. The existing theoretical research lacks in-depth analysis of the influence mechanism of children’s characteristics on professional manager appointment. Therefore, we attempt to make up for this deficiency. Based on this, we take Chinese family listed companies from 2009 to 2019 as a sample to test the impact of the number and the gender structure of actual controllers’ children on family enterprises’ professional management. The study found that the number of children, especially the number of sons, will inhibit the professional operation of enterprises. This conclusion is still true after the robustness test. Further research found that compared with daughters, sons have a more significant impact on professional management. At the same time, the greater the ownership proportion of the actual controller, the stronger the correlation between children’s characteristics and professional management. We not only studied the influencing factors of enterprise succession from the perspective of children’s characteristics, but also enriched the organization theory in the context of family business, which is helpful to promote the benign evolution and healthy and sustainable development of enterprises.

## Introduction

The Chinese saying, “the prosperity of the population and son, the prosperity of the country,” is contained in Chinese traditional culture. The more the population of future generations, especially “men,” the more stable and prosperous the family foundation. This concept also affected the management of their own business by the ancient “owners.” Due to the inconvenience of communication and transportation, the “owner” cannot personally operate business in each region and has to hire the “shopkeeper.” However, in the era of imperfect laws, this management model is likely to have moral hazard problems. If the “owner” has more children, especially boys, the appointment of the “shopkeeper” can be reduced to ensure that the family business continues to prosper for generation to generation in the family. Since their reform and opening, family enterprises have achieved a leapfrog development, from weak to strong, and made important contributions to China’s economic growth. Most of the first generation enterprise managers are nearly 60 years old. Many enterprises are facing urgent problems such as power transfer and leadership replacement. In family enterprises, the actual controller is the “owner,” while the professional manager is the “shopkeeper.” The “family planning” policy in 1982 affected many families, but the reproductive behavior of the actual controllers of family enterprises is still relatively special. Compared with ordinary people, they have a stronger reproductive desire and lower cost of extra birth. Hence, they often have more children (Guo, 2014). This creates the following questions: Will children’s number of the actual controller affect the succession decision? and Will children’s gender structure of the actual controller also affect the professional operation?

Succession decision refers to the decision of the actual controller to choose a successor; that is, the source of the final choice of successor (Deng et al., 2016). Theoretically, there are generally two sources of family business successors. One is to train children to succeed, and the other is to find professional managers from outside the enterprise (Chen and Ying, 2003). As a part of the characteristics of the actual controller, only a few scholars have paid attention to the relationship between the characteristics of the actual controller’s children and the enterprise inheritance. On the one hand, the number of children affects the success rate and risk of enterprise inheritance (Bennedsen et al., 2005; Guo, 2014, 2015). Particularly, the possibility of the existence of the enterprise in the form of family (Dou and Jia, 2008; Pan et al., 2019) and the degree of actual control efforts for the sustainable development of the enterprise (Shen and Chen, 2020). On the other hand, the gender of children may also have an impact on the inheritance of enterprises. Keating and Little (1997) pointed out that gender is an important standard for choosing successors. Under this standard, men often win, as even the eldest daughter is difficult to become the successor of the family enterprises. When the next generation of the family is only comprised of women, the probability of inheritance within the enterprise will be smaller. Notably, even some business owners would rather sell their businesses than pass them on to their daughters (Bennedsen et al., 2005; Shepherd and Zacharakis, 2017). The actual controllers are deeply influenced by China’s concept of “son inherits father’s business” and may strive to sire more boys to ensure the inheritance of his wealth when he is old (Guo, 2015). Bertrand et al. (2008) verified the impact of children’s gender differences on the inheritance of business groups by using the data of Thai family enterprises. They found that the more male offspring there are in the family, the more likely is the family to develop a family enterprise group structure when inheriting. Although these studies have noticed that there is a certain relationship between the characteristics of children and the inheritance of enterprises, the existing studies on the internal mechanism of the selection of heirs of family enterprises mainly focus on the legal system, social environment, traditional culture, and the discussion of entrepreneurs’ growth experience in many aspects (Li, 2003; Chen and Wang, 2013; Giuliano and Spilimbergo, 2013; Ansari et al., 2014; Bennedsen et al., 2015; Lv et al., 2016). There is still a lack of research on the important factor of family that is emphasized in China’s “family culture” on professional management.

**FIGURE 1:**
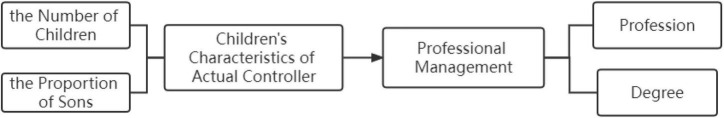
Conceptual model.

In view of this, we use Chinese family listed companies from 2009 to 2019 as the research sample, and take the characteristics of the children of the actual controller as the explanatory variable to investigate its impact on professional management. The empirical test shows that (1) when the number of children of the actual controller is large, the possibility of selecting excellent successors and the degree of absorption of the specific knowledge of the actual controller increases. On the other hand, family enterprises have altruistic behavior. The increase in the number of children entering the enterprise drives closer the relationships between family members, which is conducive to the formation of a common vision of family members, reduces the risk of enterprise succession, and making the enterprise more likely to be internally inherited. (2) When the actual controller has more sons, the traditional “son inherits the father’s business,” that is, male preference, enhances the actual controller’s desire to continue the family business from generation to generation within the family. At the same time, the family authority will increase with the increase of sons, thereby inhibiting the professional operation of the enterprise the conceptual model of our study is shown in Figure 1.

The possible contributions of our study are as follows. We firstly discuss the factors affecting the appointment of professional managers in family enterprises from a new perspective of the characteristics of the children of the actual controller. Previous studies mostly discussed the influencing factors of professional operation of family enterprises from the aspects of external institutional environment, professional managers, and family enterprise governance structure (Zhang and Wang, 2010; Chen and Wang, 2013; Ansari et al., 2014), ignoring the influence of family background. Therefore, our study starts from the inside of the family, enriching the organization theory in the context of family business by establishing the logical relationship between the characteristics of the children of the actual controller and professional management decision-making. In addition, it is helpful to deeply understand the influence of endogenous factors on succession decision-making in family enterprises. Second, with further research, we expand the influence of children’s gender on the decision-making of the actual controller. At the same time, we investigate the regulatory effect of the shareholding ratio of the actual controller, enriching the relevant literature of family business holding and help to deeply understand the impact of the actual controller’s family on the succession of family business. Third, the research conclusion of our study provides reference value for family enterprises at the peak of inheritance. The succession process of family enterprises is synchronized with the development of business families, and the number of children will affect the inheritance planning of the first-generation entrepreneurs (Chen, 2003). Therefore, combined with the governance model of Chinese family enterprises, our study analyzes the impact of the characteristics of children of actual controllers on professional operation. Hence, it can be used as a reference for family enterprises to make succession planning at the peak of intergenerational inheritance easier, help to establish a modern enterprise system, build a harmonious “collaborative symbiosis system” of family governance and corporate governance, and promote the benign evolution and healthy and sustainable development of enterprises.

## Literature Review and Research Hypothesis

“The prosperity of the population and son, the prosperity of the country” is contained in China’s unique “family” culture, which makes the characteristics of children have a far-reaching impact on Chinese family enterprises (Shen and Chen, 2020). Parents’ upbringing of their children is a process of mutual influence and change (Steel et al., 1992). As the actual controller of the family business of parents, they are also the decision-makers of the enterprise (Du et al., 2019). Their behavior decisions include the future development and management strategy of the enterprise. Hence, management decisions will also be significantly affected (Steel et al., 1992; Bennedsen et al., 2005; Dahl et al., 2012).

### The Number of Actual Controller’s Children and Professional Management

Regarding on what specific elements are needed to be inherited by the family, Dou and Jia (2008) found that entrepreneur tacit knowledge, entrepreneur relationship network, and entrepreneurship are the three types of elements that need to be inherited at the individual level of entrepreneurs in the process of intergenerational inheritance of family enterprises. Among them, the tacit knowledge of entrepreneurs is an important source of the competitiveness of family enterprises. Whether they can have this specific knowledge is very important to the success of the succession of successors (Lee et al., 2003; Tan and Fock, 2010; Minichilli et al., 2014). Since specific knowledge of family business is not institutionalized, this knowledge and ability must be inherited in long-term and frequent interactions (Williamson, 1979; Chrisman et al., 2017). Unlike professional managers, the children of the actual controller have inherent advantages in absorbing and acquiring the tacit knowledge of entrepreneurs. Guo (2014) found that compared with ordinary people, entrepreneurs have stronger desire to have children and lower costs of extra birth. Hence, there are very few private entrepreneurs who have only one child. For the actual controller who has only one child, the successor cannot fully absorb the knowledge of his parents without competition from other brothers and sisters and a lack of social trust in the external professional manager market. This because the risk of making its ability insufficient to manage family businesses is very large (Guo, 2014). The larger the number of children, the more will a successor actively participate in the operation and management of the enterprise in order to be recognized and take over the position. While trying to absorb the management experience of his parents, he will share new ideas with enterprise members, give a full play to his expertise, and reduce the transaction cost of specific knowledge. At the same time, it promotes the frequency of interaction and cooperation within the family (Shen and Cannella, 2010; Anderson et al., 2012), which makes the family provide more potential candidates for the enterprise, helps the actual controller to select excellent family successors to take appropriate positions, and reduces the risk of management failure, reducing the demand for professional managers in important positions such as chairman and general manager.

At the same time, the perspective of the family’s social emotional wealth retention is a common perspective to explain family behavior. Not all family businesses take the maximization of economic interests as the only goal of the enterprise. In addition, most family businesses also pay attention to some non-economic goals that can cause the family to continue to develop (Chua et al., 2010). Family business has gradually become an important carrier of family members’ emotional sustenance and family culture. Maintaining social emotional wealth is the core concept of family business governance and decision-making (Gómez-Mejía et al., 2007). Therefore, when considering the issue of family business successors, the ability of successors to make profits for the enterprise may not be as important as the harmonious relationship with family members, possibly affecting the selection of the next successor (Stavrou, 1999). The greater the number of children, the closer the relationship between family members, and the higher the degree of attachment to the family and organization. At the same time, relationships and attachments will be clearer, reminding family members of their identity in the family and business (Zellweger and Sieger, 2012), thereby promoting the frequent communication of family members in the management of the enterprise and enhancing their cooperation for the same interests (Mustakallio et al., 2010). During this period of cooperation, the joint operation of brothers and sisters creates more advantages in the modern fierce market competition (Lansberg, 1999), reducing the appointment of professional managers in the selection of successors to important positions.

**Hypothesis 1**. In a family firm, the more children the actual controller has, the lower the possibility of professional management.

### The Proportion of Sons of Actual Controllers and Professional Management

As a part of the internal gender structure of the family, children’s gender will affect the concept and behavior of family members. This is mainly reflected in parents’ investment, mental health, and savings in their children (Chen, 2016; Du and Zhang, 2018) and in the impact on parents’ decision-making. Scholars have found that in family businesses, it is difficult for even the eldest daughter to become a successor, especially when the next generation of the family is composed of only women. Hence, the probability of the enterprise passing on to the daughter is very small (Bennedsen et al., 2005; Shepherd and Zacharakis, 2017). Wang et al. (2019) included their son-in-law and daughter-in-law in the category of candidates for heirs in order to ensure the equality of gender and number of candidates when studying the candidates of the second generation heirs. The research results show that family businesses seem to prefer male heirs. Guo (2014) also found that only when the actual controller has no son or the son’s ability is insufficient will he choose his daughter to take over.

On the one hand, in China’s traditional inheritance system, only the successor of Zongyi can inherit property (Deng and Sang, 2016). The Chinese family continues the paternal generation relationship composed of male blood relatives. Therefore, only male offspring have the right to inherit Zongyi, and women are excluded from inheritance, resulting in the difference in the status of sons and daughters in the inheritance of family property (Zhang, 2002). The actual controller only makes decisions based on personal preferences. Therefore, under the influence of patriarchal culture and male preferences, when Chinese private entrepreneurs continue their family business, they first think of inheriting their father’s business (Liu and Zhao, 2020). If the family only has daughters, the will of inheritance within the family may be missing. If there are more sons in the family, the actual controller will give more consideration to the continuity and inheritance of the family business (Shen and Chen, 2020). Hence, the willingness of inheritance within the family is strengthened and important positions are mainly held by the second generation, reducing the employment of professional managers.

On the other hand, based on gender role theory, male characteristics are more strong, decisive, ambitious, and challenging. In China’s “family culture,” sons naturally have better force and deterrence than women, which may be useful to some regions and some people (Chen, 2016). At the same time, male heirs are generally in the center of ownership and control of enterprises or groups and have a crowding out effect on other family members. When there are multiple potential male heirs to choose from, it can not only meet the urgent desire of the actual controller to establish their own authority (Bertrand et al., 2008; Mishina et al., 2010), but also provide better personal and property protection for the family (Chen, 2016) and improve the ability and authority of family members to extract resources from the company (Bennedsen et al., 2005) to ensure the important position of the family in the enterprise. Therefore, the increase in the number of sons holding important positions, such as chairman and general manager, in the enterprise can ensure that the enterprise continues to exist in the form of a family business (Bertrand, 2008; Dou and Jia, 2008). Hence, the actual controller will reduce the employment of professional managers in important positions.

**Hypothesis 2.** In a family firm, the more sons the actual controller has, the lower the possibility of professional management.

## Materials and Methods

### Sample Selection

We draw on the definitions of family business in relevant studies, such as Su and Zhu (2003) and Xu and Lv (2011), as follows: (1) the ultimate controller can be traced to a natural person or family; and (2) the natural person or family has substantial control over the listed company. On January 1, 2008, China began to implement “the labor contract law,” which indicates that China’s labor market has become more mature. As a special labor market, the manager market is also supervised and protected by “the labor contract law” and “the labor law.” With the development of the labor market, the manager market is also becoming more mature. Therefore, we selected the next year after the implementation of the labor contract law to start the research, selected the samples of Chinese listed companies that meet the definition of the family business from 2009 to 2019 China Stock Market and Accounting Research (CSMAR), and formed panel data. The selection is mainly carried out according to the following steps. Firstly, we obtained the samples of enterprises that actually control human beings as natural persons or families according to the database of Chinese listed family enterprises provided by CSMAR. Secondly, according to the information of actual controllers, we obtained the list of board members and senior management team members disclosed in the annual report of listed companies whose kinship is further determined by blood and marriage, along with the family business samples that meet the definition. Finally, we took the children of the actual controller in CSMAR as the proxy variable to measure. We then used these data to screen the second-generation family members who have entered the enterprise.

Our analysis treated the initial sample according to the following principles: (1) financial publicly traded firms are excluded; (2) newly publicly traded firms in the current year are excluded; (3) deleting firms with missing major data; (4) the firms with significantly abnormal values of each variable are eliminated; (5) the sample of special treatment (ST) firms are removed. The firm’s financial data, governance performance, and basic firm data that were used are from CSMAR database. In addition, we excluded the samples of financial industry and missing data. In view of common practice, we searched Sina Finance, Oriental Wealth, Juchao information network, Baidu Finance, Baidu Encyclopedia, Baidu Post bar, Tianyan, and other websites and apps to manually supplement missing and suspicious data. Through the above screening steps, our analysis finally obtained 937 sample firms from 2009 to 2019, for a total of 5,564 samples. Excel and Stata15.0 were used to process the data during the research process. To avoid the influence of extreme values, the data was processed using Winsorize3%.

### Measures

#### Dependent Variables

We collectively referred to all the independent variables as professional management, which included *Profession* and *Degree* as two variables.

##### Profession

According to the CEO positions of family members (entrepreneurs and successors) and professional managers, Chen et al. (2013) divided family firms into Family CEO firms where family members serve as the CEO and Profession CEO firms where professional managers serve as the CEO. Based on this, we constructed the dummy variable “*Profession.*” If the firm does not introduce professional managers, we code it as 0. If the firm introduces professional managers into the firm, we code it as 1.

##### Degree

Claessens et al. (2000) found that in publicly traded firms in East Asian countries, general manager and chairman of the board are the key positions of the firm, and they constitute the most basic and significant ladder team in Chinese firms. The chairman of the board is responsible for making major strategic directions on major issues, while the general manager is usually responsible for daily operations and the achievement of business objectives. Therefore, the chairman is usually more powerful than the general manager (Kato and Long, 2006). Based on this, we used “*Degree*” as the classification of the professional management degree according to the importance of professional managers’ positions. If the professional manager is neither chairman nor general manager, we code it as 0. If the professional manager only served as general managers, and the chairman of the board is a family member, we code it as 1. If the professional manager only serves as the chairman of the board and the general manager is a family member, we code it as 2. If the professional manager holds concurrent positions of chairman and general manager, we code it as 3.

#### Independent Variables

We collectively refer to all the independent variables as the child characteristics of the actual controller, which includes “*Children*” and “*Sonprop*” as two variables.

##### Children

This variable indicates the number of children who had entered the enterprise by the end of the year.

##### SonProp

This variable indicates the proportion sons among the children who had entered the enterprise by the end of the year.

To test the impact of the number of sons and daughters of actual controllers on professional management, we add two more independent variables.

##### Sonnum

This variable indicates the number of sons who had entered the enterprise by the end of the year.

##### Girlnum

This variable indicates the number of daughters who had entered the enterprise by the end of the year.

#### Control Variables

Drawing on the research of Qi and Xinchun (2004), Bennedsen et al. (2005), Du and Gao (2011), Berrone et al. (2012), and Ma et al. (2018), we controlled the factors that may have an impact on professional operation as the control variables. These factors were as follows: (1) Company finance, namely, company size, asset liability ratio (Lev), return on total assets (ROA), profitability (EBIT), and Growth; (2) Corporate Governance, namely, number of board of directors (*Board*), proportion of independent directors (*Independent board*), executive compensation (Salary), and number of board meetings (Meet); and (3) Family and actual controller characteristics, namely, actual controller gender (Sex), actual controller education (Education), and company establishment time (Establish). In addition, we also introduce the dummy variables of Industry and Year. The definition and measurement of main variables are shown in Table 1.

**TABLE 1:** Variable definitions.

Type	Variable names	Variable definitions
Dependent variables	Profession	Whether the firm introduces professional managers to the position of chairman or general manager, we code it as 1; otherwise, we code it as 0.
	Degree	Firms introduce professional managers to the position of chairman or general manager: if the professional manager are neither chairman nor general manager, we code it as 0. If the professional manager only served as general managers, and the chairman of the board is a family member, we code it as 1. If the professional manager only serve as the chairman of the board and the general manager is a family member, we code it as 2. If the professional manager holding concurrent positions of chairman and general manager, we code it as 3.
Independent variables	Children	This variable indicates that by the end of the year, the number of children who had entered the enterprise.
	Sonprop	This variable indicates that by the end of the year, the sons’ proportion of the children who had entered the enterprise.
Independent variables (further research)	Sonnum	This variable indicates that by the end of the year, the number of sons who had entered the enterprise.
	Girlnum	This variable indicates that by the end of the year, the number of daughters who had entered the enterprise.
Controls	Size	(Ln Size)/10
	Lev	Total liabilities/total assets
	Roa	(Total profit + finance expenses)/Total assets
	Ebit	Ebit/Total assets
	Growth	(Operating income Amount of current period − operating income amount of the same period last year)/(Operating income amount of the same period last year)
	Board	Ln (Total number of directors)
	Independent board	Total number of independent directors in office/total number of directors
	Salary	(Ln Top three executives’ total compensation)/10
	Meet	Ln (The number of board meetings held during the year)
	Sex	If the actual controller is a man, we code it as 0, or female (=1)
	Education	Actual controller’s education: technical secondary school or below (=1), junior college (=2), undergraduate (=3), master (=4), doctor (=5).
	Establish	Ln (Date of statistics − Date of establishment + 1)
	Industry	The sample period was between 2009 and 2019
	Year	The latest industry classification of publicly traded firms firms issued by China Securities Regulatory Commission shall prevail
Moderator variable (further research)	Ownership	This variable represents the cumulative ownership proportion of the listed company held by the actual controller through all control chains, in which the ownership proportion of the top of each control chain to the terminal company is equal to the product of the shareholding proportion of shareholders at all levels of the control chain

#### Moderator Variable

To test the moderation effect of actual controller’s ownership, based on the research of La Porta et al. (1998, 2002), Claessens et al. (2002), and Wu (2015), we selected the *Ownership* proportion of the actual controller as the moderator variable.

##### Ownership

This variable represents the cumulative ownership proportion of the listed company held by the actual controller through all control chains, in which the ownership proportion of the top of each control chain to the terminal company is equal to the product of the shareholding proportion of shareholders at all levels of the control chain.

#### Research Model

To test the relationship between the children’s characteristics of the actual controller of a family firm and professional management, we established regression Equations 1–4 below. Equations 1, 2 are used to examine the impact of *Children* on *Profession* and *Degree*. Equations 3, 4 are used to examine the impact of *SonProp* on *Profession* and *Degree*.


$$Profession_{i,t}=\alpha_{0}+\alpha_{1}\times Children_{i,t}+\alpha_{2}\times Controls+\varepsilon_{i,t}$$

$$Degree_{i,t}=\alpha_{0}+\alpha_{1}\times Children_{i,t}+\alpha_{2}\times Controls+\varepsilon_{i,t}$$

$$Profession_{i,t}=\alpha_{0}+\alpha_{1}\times SonProp_{i,t}+\alpha_{2}\times Controls+\varepsilon_{i,t}$$

$$Degree_{i,t}=\alpha_{0}+\alpha_{1}\times SonProp_{i,t}+\alpha_{2}\times Controls+\varepsilon_{i,t}$$

Among them, the subscript “i” represents the “i”th publicly traded family firms, “t” is on behalf of the “year,” and the “ε” is on behalf of the residuals. Referring to the practice of classical literature, we performed model estimation on ordinary least squares (OLS) regression of various fixed effects and controlled the fixed effects of “*Industry*” and “*Year.*” The “*Control*” includes three aspects, namely, financial characteristics of the firm, corporate governance characteristics, and family and actual controller characteristics.

## Results

### Descriptive Statistics

Descriptive statistics were conducted for *dependent variables*, *independent variables*, and main *control variables* in the study, and the statistical results are shown in Table 2. As can be seen from Table 2, the average value of *Profession* is 0.465 and *Degree* is 0.705, indicating that publicly traded Chinese family firms are less likely to introduce professional managers. Even when firms bring in professional managers, they are reluctant to be put in important positions. The average number of *Children* of actual controllers in publicly traded Chinese family firms is 1.559, which translates to at least 1 and, at most 5, while the mean of *SonProp* is 0.682 (>0.667), which is more than 66.67%. The mean of *Sonnum* is 1.041, but the mean of *Girlnum* is 0.518, almost half the number of sons, which means that the actual controller is more willing to let sons enter the firm.

**TABLE 2:** Descriptive statistics.

Variable	Obs	Mean	S. D.	Min	Max	P5	P25	P50	P75	P95
Profession	5564	0.465	0.499	0	1	0	0	0	1	1
Degree	5564	0.705	0.958	0	3	0	0	0	1	3
Children	5564	1.559	0.653	1	5	1	1	1	2	3
SonProp	5564	0.682	0.420	0	1	0	0.333	1	1	1
Sonnum	5564	1.041	0.743	0	4	0	1	1	2	2
Girlnum	5564	0.518	0.701	0	4	0	0	0	1	2
Size	5564	21.773	1.066	18.187	26.859	20.297	20.986	21.681	22.399	23.704
Lev	5564	0.37	0.196	0.008	0.985	0.089	0.210	0.351	0.509	0.725
Roa	5564	0.066	0.046	−0.098	0.219	0.007	0.037	0.061	0.091	0.149
Ebit	5564	0.021	0.025	−0.035	0.208	−0.002	0.008	0.015	0.026	0.070
Growth	5564	0.185	0.452	−0.709	9.974	−0.215	0.004	0.125	0.277	0.702
Board	5564	2.106	0.186	1.609	2.89	1.609	1.946	2.197	2.197	2.398
Independent board	5564	0.372	0.051	0.333	0.667	0.333	0.333	0.333	0.429	0.444
Salary	5564	14.209	0.733	11.236	17.9	13.020	13.740	14.198	14.648	15.425
Meet	5564	2.154	0.377	0.693	3.497	1.609	1.950	2.197	2.398	2.773
Sex	5564	0.934	0.248	0	1	0	1	1	1	1
Education	5564	2.793	1.122	1	5	1	2	3	4	4
Establish	5564	2.674	0.447	0.693	3.555	1.792	2.485	2.773	2.996	3.219
Ownership	5564	37.922	15.955	6.143	71.560	12.942	25.380	37.293	50.371	64.600

### Correlation

This section shows the Pearson correlation coefficient matrix among *dependent*, *independent variables*, and main *control variables* in the study. Table 3 reports the correlation coefficient among variables. In order to prevent multicollinearity from affecting the empirical regression results, the variance inflation factor (VIF) diagnosis is also adopted in our study. The VIF of explanatory variables are all less than 2, and the mean VIF of all variables is less than 2, eliminating the multicollinearity problem and ensuring the unbiased and consistent model estimation.

**TABLE 3:** Correlation matrix.

	Profession	Degree	Children	SonProp	Sonnum	Girlnum	Size	Lev	Roa
Profession	1								
Degree	0.790***	1							
Children	−0.069***	−0.057***	1						
SonProp	−0.050***	−0.013	−0.081***	1					
Sonnum	−0.075***	−0.042***	0.502***	0.758***	1				
Girlnum	0.015	−0.009	0.399***	−0.879***	−0.592***	1			
Size	0.151***	0.117***	0.013	−0.029**	−0.017	0.031**	1		
Lev	0.148***	0.148***	−0.042***	0.019	−0.004	−0.035***	0.543***	1	
Roa	0.003	−0.0110	0.030**	−0.043***	−0.013	0.042***	−0.031**	−0.248***	1
Ebit	−0.054***	−0.065***	0.023*	−0.024*	−0.006	0.028**	−0.112***	−0.182***	0.556***
Growth	0	−0.006	0.007	−0.007	0.002	0.004	0.056***	0.064***	0.124***
Board	0.104***	0.089***	0.039***	−0.008	0.012	0.023*	0.195***	0.099***	0.064***
Independent board	−0.100***	−0.102***	0.023*	0	0.013	0.00800	−0.089***	−0.046***	−0.055***
Salary	0.059***	0.047***	0.030**	−0.060***	−0.035***	0.066***	0.511***	0.153***	0.151***
Meet	0.078***	0.061***	−0.011	−0.031**	−0.022*	0.014	0.300***	0.254***	−0.076***
Education	0.040***	0.045***	−0.141***	0.020	−0.071***	−0.056***	0.040***	0.044***	0.005
Sex	0.058***	0.045***	−0.101***	0.014	−0.046***	−0.045***	0.070***	0.043***	−0.005
Establish	0.076***	0.109***	0.002	0.023*	−0.017	−0.022*	0.178***	0.137***	−0.070***
Ownership	−0.248***	−0.250***	0.127***	−0.021	0.075***	0.039***	−0.154***	−0.143***	0.093***

	Ebit	Growth	Board	Indboard	Salary	Meet	Education	Sex	Establish	Ownership
Ebit	1									
Growth	0.006	1								
Board	0.047***	−0.003	1							
Indboard	−0.035***	0.005	−0.615***	1						
Salary	0.070***	0.024*	0.131***	−0.053*^***^	1					
Meet	−0.170***	0.123***	0.031**	−0.021	0.132***	1				
Education	−0.011	0.031**	0.017	−0.007	0.084***	0.110***	1			
Sex	0.005	−0.001	−0.006	−0.027**	0.006	0.036***	0.027**	1		
Establish	−0.131***	−0.033**	−0.044***	0.039***	0.217***	0.044***	0.036***	0.020	1	
Ownership	0.169***	0.007	−0.129***	0.111***	−0.004	−0.041***	−0.0170	−0.054***	−0.098***	1

*, **, and *** represent significant at 10, 5, and 1% levels, respectively.

Table 3 presents that *Children* is significantly negatively correlated with *Profession* with a coefficient of −0.069, and that *Children* is significantly negatively correlated with *Degree* with a coefficient of −0.057. In addition, *SonProp* is significantly negatively correlated with *Profession* with a coefficient of −0.050 and is negatively correlated with *Degree*, but not significantly. Overall, hypothesis 1 and hypothesis 2 in our study are preliminarily verified, but the above relationship needs to be further tested by regression analysis.

### Multiple Regression Analysis

#### The Number of Children of Actual Controllers and Professional Management

*Hypothesis 1* predicts that in family firms, there is a negative correlation between the actual controller’s number of children and professional management. This part verifies this conclusion on the basis of controlling related factors. To ensure robustness of the results, *Column (1)* and *(5)* of Table 4 only controls the *Industry* and *Year two* dummy variables. On this basis, the firm’s financial characteristic variables are added in *Column (2)* and *Column (4)* of Table 4. Then, corporate governance characteristic variables are added to *Column (3)* and *(7)* of Table 4. Finally, the characteristic variables of family and the actual controller are further added in *Column (4)* and *(8)* of Table 4, and the regression results are shown in Table 4.

**TABLE 4:** Multiple regression analysis: the number of children of actual controllers and professional management.

	(1)	(2)	(3)	(4)	(5)	(6)	(7)	(8)
	Profession	Profession	Profession	Profession	Degree	Degree	Degree	Degree
Children	−0.038***	−0.037***	−0.037***	−0.035***	−0.050***	−0.045**	−0.043**	−0.037*
	(−3.77)	(−3.56)	(−3.63)	(−3.43)	(−2.62)	(−2.38)	(−2.24)	(−1.96)
Size		0.046***	0.036***	0.037***		0.042***	0.018	0.023
		(5.87)	(4.01)	(4.19)		(2.83)	(1.05)	(1.40)
Lev		0.131***	0.127***	0.097**		0.288***	0.294***	0.207**
		(2.97)	(2.86)	(2.17)		(3.48)	(3.53)	(2.49)
Roa		0.466***	0.410**	0.354**		0.680**	0.508	0.349
		(2.60)	(2.27)	(1.96)		(2.03)	(1.50)	(1.04)
Ebit		−1.235***	−1.208***	−1.050***		−2.776***	−2.782***	−2.328***
		(−3.93)	(−3.82)	(−3.32)		(−4.72)	(−4.71)	(−3.95)
Growth		−0.024	−0.024	−0.020		−0.040	−0.039	−0.029
		(−1.63)	(−1.64)	(−1.40)		(−1.48)	(−1.42)	(−1.09)
Board			0.047	0.058			−0.093	−0.065
			(1.03)	(1.28)			(−1.09)	(−0.76)
Independent board			−0.569***	−0.554***			−1.516***	−1.49***
			(−3.58)	(−3.50)			(−5.09)	(−5.05)
Salary			0.008	0.007			0.049**	0.045**
			(0.69)	(0.64)			(2.30)	(2.13)
Meet			0.028	0.032*			0.032	0.041
			(1.49)	(1.70)			(0.92)	(1.19)
Education				0.0004				0.014
				(0.08)				(1.25)
Sex				0.079***				0.152***
				(3.03)				(3.14)
Establish				0.0993***				0.277***
				(5.74)				(8.59)
_Cons	0.974***	−0.0436	0.132	−0.239	2.700***	1.731***	2.321***	1.345***
	(9.19)	(−0.24)	(0.58)	(−1.02)	(13.64)	(4.98)	(5.42)	(3.07)
Ind	Control	Control	Control	Control	Control	Control	Control	Control
Year	Control	Control	Control	Control	Control	Control	Control	Control
*N*	5,564	5,564	5,564	5,564	5,564	5,564	5,564	5,564
*R* ^2^	0.110	0.126	0.131	0.138	0.158	0.169	0.175	0.187

t statistics in parentheses *p < 0.1, **p < 0.05, ***p < 0.01.

Table 4 presents the test results of *Children* and professional management (*Profession* and *Degree*). All the regression results of each column show that there is a significant negative correlation between the actual controller’s number of children and professional management (*Profession* and *Degree*). First of all, it can be seen from *Column (1)* and *Column (5)* that the regression coefficients of *Children* and *Profession* is −0.038, and that of *Children* and *Degree* is −0.050, which are significantly negative in the statistical sense of 1%, indicating that *Children* are significantly negatively correlated with professional management (*Profession* and *Degree*) without the addition of other major control variables. As *Children* increases by one unit, *Profession* decreases by 0.038 units and *Degree* decreases by 0.05 units. After adding the characteristic variables of corporate finance, corporate governance, family, and actual controller in the *Column (2)*, *Column (3)*, and *Column (4)*, respectively, it is found that the coefficient of *Children* and *Profession* is always significantly negative at the level of 1%. After the addition of the characteristic variables of corporate finance and corporate governance in *Column (6)* and *Column (7)*, respectively, *Children* and *Degree* are always significantly negative at the level of 5%. In *Column (8)*, although *Children* and *Degree* are affected by the characteristic variables of family and actual controller, they still show a significant negative correlation. In addition, it can be seen from *Column (4)* and *Column (8)* that as *Children* increases by one unit, *Profession* and *Degree* decreases by 0.035 and 0.037 units, respectively, and *Children*’s influence on *Profession* is greater than that on *Degree.* On the one hand, this result is due to the increase in the number of children, the tacit knowledge of the actual controller that can be fully absorbed (Yu et al., 2013), and how the actual controller is more likely to select excellent children, reducing the risk of children’s succession (Guo, 2014). On the other hand, the increase in the number of children promotes the interaction frequency of family members in the management of the enterprise, causing the relationship between family members to be closer (Mustakallio et al., 2010; Zellweger and Sieger, 2012), thereby helping in making consistent decisions for a common vision. That is, the more children of the actual controller of the family business enters the enterprise, the stronger the inhibition of professional operation. Therefore, Hypothesis 1 is supported.

#### The Proportion of the Sons of Actual Controllers and Professional Management

*Hypothesis 2* predicts that in family firms, there is a negative correlation between the proportion of sons of actual controllers and professional management. This part verifies this conclusion on the basis of controlling related factors. To ensure robustness of the results, *Column (1)* and *(5)* of Table 5 only controls the *Industry* and *Year two* dummy variables. On this basis, the firm’s financial characteristic variables are added in *Column (2)* and *Column (4)* of Table 5. Then, corporate governance characteristic variables are added to *Column (3)* and *(7)* of Table 5. Finally, the characteristic variables of family and actual controller are further added in *Column (4)* and *(8)* of Table 5, and the regression results are shown in Table 5.

**TABLE 5:** Multiple regression analysis: the proportion of sons of actual controllers and professional management.

	(1)	(2)	(3)	(4)	(5)	(6)	(7)	(8)
	Profession	Profession	Profession	Profession	Degree	Degree	Degree	Degree
SonProp	−0.051***	−0.053***	−0.052***	−0.055***	−0.018	−0.024	−0.020	−0.030
	(−3.27)	(−3.40)	(−3.32)	(−3.54)	(−0.61)	(−0.84)	(−0.70)	(−1.03)
Size		0.044***	0.035***	0.037***		0.040***	0.017	0.023
		(5.66)	(3.97)	(4.15)		(2.72)	(1.02)	(1.37)
Lev		0.145***	0.140***	0.109**		0.302***	0.305***	0.218***
		(3.29)	(3.15)	(2.45)		(3.65)	(3.67)	(2.62)
Roa		0.444**	0.394**	0.336*		0.668**	0.501	0.339
		(2.48)	(2.18)	(1.86)		(1.99)	(1.48)	(1.01)
Ebit		−1.262***	−1.229***	−1.075***		−2.799***	−2.797***	−2.348***
		(−4.02)	(−3.89)	(−3.40)		(−4.76)	(−4.73)	(−3.98)
Growth		−0.024*	−0.024*	−0.021		−0.042	−0.039	−0.030
		(−1.66)	(−1.66)	(−1.43)		(−1.50)	(−1.44)	(−1.10)
Board			0.034	0.047			−0.107	−0.077
			(0.75)	(1.03)			(−1.26)	(−0.91)
Independent board			−0.603***	−0.586***			−1.556***	−1.526***
			(−3.80)	(−3.70)			(−5.24)	(−5.17)
Salary			0.005	0.005			0.047**	0.043**
			(0.47)	(0.40)			(2.22)	(2.04)
Meet			0.026	0.030			0.031	0.040
			(1.42)	(1.59)			(0.91)	(1.15)
Education				0.004				0.017
				(0.59)				(1.52)
Sex				0.087***				0.161***
				(3.37)				(3.33)
Establish				0.100***				0.277***
				(5.78)				(8.58)
_Cons	0.990***	0.007	0.228	−0.152	2.671***	1.741***	2.374***	1.398***
	(9.27)	(0.04)	(0.99)	(−0.65)	(13.39)	(4.99)	(5.52)	(3.18)
Ind	Control	Control	Control	Control	Control	Control	Control	Control
Year	Control	Control	Control	Control	Control	Control	Control	Control
*N*	5,564	5,564	5,564	5,564	5,564	5,564	5,564	5,564
*R* ^2^	0.109	0.126	0.131	0.138	0.157	0.168	0.174	0.187

t statistics in parentheses *p < 0.1, **p < 0.05, ***p < 0.01.

Table 5 presents the test results of *SonProp* and professional management (*Profession* and *Degree*). The regression results in *Columns (1)* to *(4)* show that there is a significant negative correlation between *SonProp* and *Profession* at 1% level, while *Columns (2)* to *(8)* show that *SonProp* has no significant correlation with *Degree*. As can be seen from the *Column (1)*, the regression coefficient of SonProp is −0.051, which is statistically significant at 1%, indicating that as the *SonProp* increases by one unit, *Profession* decreases by 0.051 units. After adding the characteristic variables of corporate finance, corporate governance and family, and actual controller in *Column (2)*, *(3)*, and *(4)*, respectively, it is found that the coefficients of *SonProp* and *Profession* are significantly negative at the 1% level. Finally, *Column (4)* shows that for every unit added to *SonProp*, *Profession* decreases by 0.055 units.

On the one hand, under the influence of China’s traditional concept of “son inherits father’s business,” the more sons, especially adult sons, in the family, the more the actual controller will take into account the continuation and inheritance of the family business and show a strong willingness to inherit internally (Shen and Chen, 2020), inhibiting professional management. On the other hand, when there are multiple potential male heirs to choose from, the actual controller has a more urgent desire to establish his own authority. The increase in the number of sons holding important positions provides the actual controller with the possibility of establishing authority, thus reducing the employment of professional managers. Therefore, Hypothesis 2 is supported.

### Robustness Tests

#### Change the Sample Size

According to the data of “the report on the inheritance of Chinese family” enterprises released in 2015, 15% of the second generation clearly expressed their unwillingness to take over, another 45% of the second generation did not have a clear attitude towards taking over, and only 40% of the second generation clearly expressed their willingness to take over. It can be seen that the willingness of children to take over has decreased in recent years. In order to test whether our research results are still applicable in recent years, we firstly use the method of changing the sample time to separately select the recent samples from 2015 to 2019. Afterwards, we carried out robustness tests on the relationship between the characteristics of actual controller’s children and professional management. The test results are shown in Tables 6, 7.

**TABLE 6:** Change the sample size: the number of children of actual controllers and professional management.

	(1)	(2)	(3)	(4)	(5)	(6)	(7)	(8)
	Profession	Profession	Profession	Profession	Degree	Degree	Degree	Degree
Children	−0.051***	−0.052***	−0.052***	−0.0522***	−0.071***	−0.072***	−0.072***	−0.068***
	(−3.82)	(−3.91)	(−3.93)	(−3.91)	(−2.86)	(−2.93)	(−2.93)	(−2.75)
Size		0.058***	0.050***	0.051***		0.082***	0.054***	0.058***
		(5.90)	(4.41)	(4.51)		(4.42)	(2.58)	(2.76)
Lev		0.008	−0.004	−0.007		−0.050	−0.074	−0.077
		(0.13)	(−0.07)	(−0.11)		(−0.44)	(−0.63)	(−0.67)
Roa		0.252	0.214	0.206		0.124	0.004	−0.017
		(1.07)	(0.90)	(0.87)		(0.28)	(0.01)	(−0.04)
Ebit		−1.326***	−1.297***	−1.205***		−3.090***	−3.093***	−2.826***
		(−2.96)	(−2.89)	(−2.67)		(−3.71)	(−3.70)	(−3.37)
Growth		−0.011	−0.011	−0.010		−0.030	−0.031	−0.026
		(−0.61)	(−0.62)	(−0.54)		(−0.88)	(−0.92)	(−0.79)
Board			0.090	0.093			0.109	0.114
			(1.43)	(1.49)			(0.93)	(0.98)
Independent board			−0.402*	−0.395*			−0.892**	−0.886**
			(−1.85)	(−1.82)			(−2.20)	(−2.20)
Salary			0.001	−0.001			0.035	0.027
			(0.04)	(−0.07)			(1.24)	(0.97)
Meet			0.030	0.033			0.076*	0.078*
			(1.23)	(1.34)			(1.68)	(1.74)
Education				−0.002				0.025*
				(−0.19)				(1.70)
Sex				0.030				0.071
				(0.83)				(1.06)
Establish				0.083***				0.247***
				(2.88)				(4.63)
_Cons	1.079***	−0.158	−0.079	−0.362	3.103***	1.418***	1.501**	0.664
	(7.10)	(−0.63)	(−0.25)	(−1.09)	(10.97)	(3.03)	(2.54)	(1.08)
Ind	Control	Control	Control	Control	Control	Control	Control	Control
Year	Control	Control	Control	Control	Control	Control	Control	Control
*N*	3,256	3,256	3,256	3,256	3,256	3,256	3,256	3,256
*R* ^2^	0.102	0.119	0.124	0.127	0.147	0.159	0.164	0.171

t statistics in parentheses *p < 0.1, **p < 0.05, ***p < 0.01.

**TABLE 7:** Change in the sample size: the proportion of sons of actual controllers and professional management.

	(1)	(2)	(3)	(4)	(5)	(6)	(7)	(8)
	Profession	Profession	Profession	Profession	Degree	Degree	Degree	Degree
SonProp	−0.064***	−0.067***	−0.070***	−0.072***	−0.057	−0.067*	−0.069*	−0.076**
	(−3.11)	(−3.29)	(−3.42)	(−3.52)	(−1.50)	(−1.75)	(−1.81)	(−2.01)
Size		0.057***	0.050***	0.051***		0.080***	0.054**	0.057***
		(5.73)	(4.37)	(4.46)		(4.31)	(2.56)	(2.73)
Lev		0.027	0.016	0.014		−0.026	−0.049	−0.051
		(0.44)	(0.25)	(0.23)		(−0.23)	(−0.42)	(−0.44)
Roa		0.207	0.173	0.166		0.074	−0.042	−0.063
		(0.88)	(0.73)	(0.70)		(0.17)	(−0.10)	(−0.14)
Ebit		−1.325***	−1.290***	−1.205***		−3.075***	−3.071***	−2.819***
		(−2.96)	(−2.87)	(−2.67)		(−3.69)	(−3.67)	(−3.36)
Growth		−0.011	−0.011	−0.009		−0.029	−0.031	−0.026
		(−0.59)	(−0.59)	(−0.50)		(−0.88)	(−0.91)	(−0.77)
Board			0.070	0.073			0.080	0.087
			(1.12)	(1.17)			(0.69)	(0.75)
Independent board			−0.486**	−0.479**			−1.004**	−0.994**
			(−2.24)	(−2.22)			(−2.49)	(−2.47)
Salary			−0.003	−0.005			0.032	0.024
			(−0.17)	(−0.30)			(1.12)	(0.83)
Meet			0.028	0.0293			0.073	0.074*
			(1.14)	(1.21)			(1.61)	(1.65)
Education				0.002				0.030**
				(0.31)				(2.04)
Sex				0.040				0.084
				(1.12)				(1.26)
Establish				0.084***				0.248***
				(2.92)				(4.65)
_Cons	1.094***	−0.109	0.062	−0.233	3.091***	1.453***	1.654***	0.808
	(7.14)	(−0.43)	(0.19)	(−0.70)	(10.86)	(3.09)	(2.78)	(1.31)
Ind	Control	Control	Control	Control	Control	Control	Control	Control
Year	Control	Control	Control	Control	Control	Control	Control	Control
*N*	3,256	3,256	3,256	3,256	3,256	3,256	3,256	3,256
*R* ^2^	0.101	0.118	0.123	0.126	0.145	0.158	0.163	0.170

t statistics in parentheses *p < 0.1, **p < 0.05, ***p < 0.01.

Table 6 presents the test results of the relationship between *Children* and professional management (*Profession* and *Degree*) after changing the number of samples. From *Column (1)* to *(8)*, there is no overall difference in test results after partially controlling all variables and that *Children* are still significantly negatively correlated with professional management (*Profession* and *Degree*). This proves that the regression results of the relationship between *Children* and professional management (*Profession* and *Degree*) are robust.

Table 7 presents the test results of the relationship between *SonProp* and professional management (*Profession* and *Degree*). From *Column (1)*, it can be seen that there is no overall difference in test results after partial control and control of all variables. *SonProp* and *Profession* still show a significant negative correlation at 1% level. *Column (6)* shows a significant negative correlation between *SonProp* and *Degree* at the level of 10%, while *Column (7)* and *(8)* show a significant negative correlation at the level of 5%, which is generally consistent with the hypothesis of the *SonProp* and professional management (*Profession* and *Degree*), proving that the regression results of the relationship between *SonProp* and professional management (*Profession* and *Degree*) are robust.

#### Replacement of Control Variables

In order to ensure the robustness of the results, we used the method of replacing partial control variables. Referring to the research of Chen et al. (2013), Li et al. (2014), Zhao and Lv (2017), in terms of corporate finance, we replaced the *Roa* with the net profit margin of total assets (*Roe*). The calculation method was *Roe* = (net profit)/(total assets balance). In terms of corporate governance, the *Independent board* was replaced by institutional investor ownership ratio (*Inst*). At the level of family and actual controller characteristics, *Establish* was replaced with family time (*Family year*), and the calculation method was *Family year* = ln (statistical date − family date + 1). Robustness test was conducted on the relationship between the characteristics of actual controller’s children and professional management. The test results are shown in Tables 8, 9. It can be seen that there is no difference in the test results as a whole, which proves that the regression result of the relationship between the characteristics of actual controller’s children and the professional management is robust.

**TABLE 8:** Replacement of the control variables: the number of children of actual controllers and professional management.

	(1)	(2)	(3)	(4)	(5)	(6)	(7)	(8)
	Profession	Profession	Profession	Profession	Degree	Degree	Degree	Degree
Children	−0.038***	−0.037***	−0.039***	−0.030***	−0.0498***	−0.045**	−0.049***	−0.036*
	(−3.77)	(−3.65)	(−3.88)	(−3.37)	(−2.62)	(−2.38)	(−2.58)	(−1.91)
Size		0.047***	0.027***	−0.006		0.044***	−0.004	−0.075***
		(6.00)	(3.05)	(−0.66)		(2.97)	(−0.23)	(−4.30)
Lev		0.134***	0.102**	0.061		0.285***	0.221**	0.133
		(2.90)	(2.20)	(1.33)		(3.29)	(2.55)	(1.56)
Roe		0.343*	0.142	0.107		0.419	−0.114	−0.187
		(1.73)	(0.70)	(0.54)		(1.13)	(−0.30)	(−0.51)
Ebit		−1.072***	−1.159***	0.025		−2.473***	−2.765***	−0.237
		(−3.47)	(−3.75)	(0.08)		(−4.28)	(−4.79)	(−0.39)
Growth		−0.022	−0.022	−0.017		−0.036	−0.036	−0.024
		(−1.49)	(−1.55)	(−1.16)		(−1.34)	(−1.34)	(−0.91)
Board			0.133***	0.147***			0.139**	0.169**
			(3.67)	(4.10)			(2.05)	(2.53)
Inst			0.020***	0.021***			0.050***	0.051***
			(7.42)	(7.74)			(9.78)	(10.18)
Salary			0.003	0.012			0.037*	0.054***
			(0.29)	(1.08)			(1.76)	(2.61)
Meet			0.028	0.041**			0.032	0.058*
			(1.51)	(2.25)			(0.93)	(1.71)
Education				−0.0008				0.011
				(−0.13)				(1.01)
Sex				0.067***				0.126***
				(2.59)				(2.63)
Family year				0.105***				0.225***
				(10.83)				(12.43)
_Cons	0.974***	−0.058	−0.125	0.101	2.700***	1.706***	1.617***	2.100***
	(9.19)	(−0.31)	(−0.63)	(0.50)	(13.64)	(4.92)	(4.33)	(5.61)
Ind	Control	Control	Control	Control	Control	Control	Control	Control
Year	Control	Control	Control	Control	Control	Control	Control	Control
*N*	5,564	5,564	5,564	5,564	5,564	5,564	5,564	5,564
*R* ^2^	0.110	0.126	0.137	0.157	0.158	0.168	0.185	0.209

t statistics in parentheses *p < 0.1, **p < 0.05, ***p < 0.01.

**TABLE 9:** Replacement of the control variables: the proportion of sons of actual controllers and professional management.

	(1)	(2)	(3)	(4)	(5)	(6)	(7)	(8)
	Profession	Profession	Profession	Profession	Degree	Degree	Degree	Degree
SonProp	−0.051***	−0.054***	−0.056***	−0.061***	−0.018	−0.026	−0.030	−0.042
	(−3.27)	(−3.44)	(−3.59)	(−3.98)	(−0.61)	(−0.88)	(−1.03)	(−1.46)
Size		0.045***	0.027***	−0.007		0.042***	−0.005	−0.076***
		(5.78)	(2.98)	(−0.78)		(2.86)	(−0.28)	(−4.36)
Lev		0.148***	0.116**	0.074		0.299***	0.235***	0.144*
		(3.20)	(2.50)	(1.60)		(3.44)	(2.72)	(1.69)
Roe		0.327*	0.132	0.096		0.410	−0.118	−0.194
		(1.65)	(0.66)	(0.48)		(1.10)	(−0.31)	(−0.53)
Ebit		−1.106***	−1.194***	0.006		−2.500***	−2.792***	−0.252
		(−3.59)	(−3.86)	(0.02)		(−4.33)	(−4.83)	(−0.42)
Growth		−0.022	−0.023	−0.017		−0.037	−0.037	−0.025
		(−1.53)	(−1.59)	(−1.20)		(−1.36)	(−1.37)	(−0.93)
Board			0.125***	0.141***			0.130*	0.163**
			(3.47)	(3.93)			(1.93)	(2.44)
Inst			0.021***	0.021***			0.050***	0.052***
			(7.51)	(7.85)			(9.79)	(10.21)
Salary			0.0004	0.009			0.035*	0.052**
			(0.03)	(0.82)			(1.65)	(2.50)
Meet			0.027	0.039**			0.032	0.057*
			(1.44)	(2.14)			(0.92)	(1.67)
Education				0.002				0.014
				(0.38)				(1.29)
Sex				0.075***				0.134***
				(2.92)				(2.82)
Family year				0.107***				0.227***
				(11.02)				(12.51)
_Cons	0.990***	−0.005	−0.044	0.185	2.671***	1.719***	1.657***	2.153***
	(9.27)	(−0.03)	(−0.22)	(0.91)	(13.39)	(4.92)	(4.41)	(5.71)
Ind	Control	Control	Control	Control	Control	Control	Control	Control
Year	Control	Control	Control	Control	Control	Control	Control	Control
*N*	5,564	5,564	5,564	5,564	5,564	5,564	5,564	5,564
*R* ^2^	0.109	0.125	0.137	0.157	0.157	0.168	0.184	0.208

t statistics in parentheses *p < 0.1, **p < 0.05, ***p < 0.01.

After replacing some control variables, Table 8 presents the test results of the relationship between *Children* and professional management (*Profession* and *Degree*). It can be seen from *Column (1)* to *(8)* that there is no overall difference in the test results after partial control and control of all variables. In addition, there is still a significant negative correlation between *Children* and professional management (*Profession* and *Degree*). This proves that the regression results of the relationship between *Children* and professional management (*Profession* and *Degree*) are robust.

After replacing some control variables, Table 9 presents the test results of the relationship between *SonProp* and professional management (*Profession* and *Degree*). It can be seen from *Column (1)* to *(4)* that there is no overall difference in the test results after partial control and control of all variables and that there is still a significant negative correlation between *SonProp* and professional management (*Profession* and *Degree*). This proves that the regression results of the relationship between *SonProp* and professional management (*Profession* and *Degree*) are robust.

### Further Study

The previous study found that the characteristics of children of actual controllers were significantly negatively correlated with professional management. In this part, we tested the impact of the number of sons and daughters of actual controllers on professional management, and the effect of ownership ratio of actual controllers on the number of children and professional management.

#### Number of Sons, Number of Daughters, and Professional Management

It has been verified above that both *Children* and *SonProp* are significantly negatively correlated with professional management (*Profession* and *Degree*). In this part, we use *Sonnum* for the number of sons, and *Girlnum* for the number of daughters to reflect the difference between the number of children’s gender and professional management relationship. This will be verified on the basis of controlling related variables. To ensure the robustness of the results, *Columns (1)* and *(2)* of Tables 10, 11 control for only Industry and Year dummy variables. On this basis, the firm’s financial characteristic variables are added in *Columns (3)* and *(4)*. Then, corporate governance characteristic variables are added in *Columns (5)* and *(6)*. Finally, the characteristic variables of family and actual controller are added in *Columns (7)* and *(8)*. The regression results are shown in Tables 10, 11.

**TABLE 10:** Number of sons, daughters, and professions.

	(1)	(2)	(3)	(4)	(5)	(6)	(7)	(8)
	Profession	Profession	Profession	Profession	Profession	Profession	Profession	Profession
SonNum	−0.041***		−0.041***		−0.041***		−0.042***	
	(−4.62)		(−4.66)		(−4.61)		(−4.70)	
GirlNum		0.013		0.015		0.014		0.017*
		(1.39)		(1.55)		(1.52)		(1.83)
Size			0.045***	0.044***	0.036***	0.035***	0.037***	0.037***
			(5.72)	(5.67)	(3.99)	(3.95)	(4.17)	(4.12)
Lev			0.141***	0.144***	0.136***	0.139***	0.105**	0.109**
			(3.20)	(3.26)	(3.07)	(3.12)	(2.35)	(2.44)
Roa			0.451**	0.458**	0.401**	0.406**	0.343*	0.348*
			(2.52)	(2.55)	(2.22)	(2.24)	(1.91)	(1.93)
Ebit			−1.261***	−1.258***	−1.230***	−1.225***	−1.070***	−1.073***
			(−4.02)	(−4.00)	(−3.90)	(−3.87)	(−3.39)	(−3.39)
Growth			−0.024	−0.024*	−0.024*	−0.024*	−0.020	−0.021
			(−1.64)	(−1.66)	(−1.65)	(−1.67)	(−1.40)	(−1.44)
Board					0.0417	0.033	0.054	0.045
					(0.92)	(0.72)	(1.19)	(0.98)
Independent board					−0.584***	−0.610***	−0.566***	−0.594***
					(−3.68)	(−3.84)	(−3.58)	(−3.75)
Salary					0.00524	0.006	0.005	0.005
					(0.47)	(0.53)	(0.41)	(0.46)
Meet					0.0267	0.028	0.030*	0.031*
					(1.44)	(1.48)	(1.65)	(1.65)
Education							0.001	0.004
							(0.24)	(0.60)
Sex							0.083***	0.089***
							(3.19)	(3.41)
Establish							0.101***	0.099***
							(5.85)	(5.71)
_Cons	0.979***	0.939***	−0.012	−0.050	0.191	0.174	−0.190	−0.205
	(9.25)	(8.88)	(−0.07)	(−0.27)	(0.84)	(0.76)	(−0.81)	(−0.87)
Ind	Control	Control	Control	Control	Control	Control	Control	Control
Year	Control	Control	Control	Control	Control	Control	Control	Control
*N*	5,564	5,564	5,564	5,564	5,564	5,564	5,564	5,564
*R* ^2^	0.111	0.124	0.127	0.124	0.132	0.129	0.139	0.136

t statistics in parentheses *p < 0.1, **p < 0.05, ***p < 0.01.

**TABLE 11:** Number of sons, daughters, and degrees.

	(1)	(2)	(3)	(4)	(5)	(6)	(7)	(8)
	Degree	Degree	Degree	Degree	Degree	Degree	Degree	Degree
SonNum	−0.032*		−0.033**		−0.030*		−0.032*	
	(−1.90)		(−1.98)		(−1.80)		(−1.94)	
GirlNum		−0.007		−0.002		−0.003		0.005
		(−0.40)		(−0.11)		(−0.16)		(0.26)
Size			0.040***	0.040**	0.017	0.017	0.023	0.023
			(2.73)	(2.75)	(1.03)	(1.02)	(1.38)	(1.36)
Lev			0.300***	0.299***	0.304***	0.303***	0.216***	0.217***
			(3.63)	(3.61)	(3.66)	(3.64)	(2.60)	(2.61)
Roa			0.667**	0.678**	0.501	0.508	0.341	0.347
			(1.99)	(2.02)	(1.48)	(1.50)	(1.01)	(1.03)
Ebit			−2.802***	−2.791***	−2.802***	−2.791***	−2.347***	−2.345***
			(−4.77)	(−4.75)	(−4.74)	(−4.72)	(−3.98)	(−3.97)
Growth			−0.040	−0.041	−0.039	−0.039	−0.029	−0.030
			(−1.49)	(−1.50)	(−1.43)	(−1.44)	(−1.09)	(−1.11)
Board					−0.102	−0.106	−0.071	−0.077
					(−1.19)	(−1.25)	(−0.84)	(−0.91)
Independent board					−1.541***	−1.555***	−1.511***	−1.528***
					(−5.19)	(−5.23)	(−5.12)	(−5.18)
Salary					0.046**	0.048**	0.042**	0.043**
					(2.19)	(2.26)	(2.02)	(2.07)
Meet					0.031	0.032	0.040	0.040
					(0.90)	(0.92)	(1.16)	(1.17)
Education							0.016	0.017
							(1.39)	(1.51)
Sex							0.158***	0.161***
							(3.26)	(3.33)
Establish							0.278***	0.276***
							(8.62)	(8.56)
_Cons	2.685***	2.652***	1.748***	1.709***	2.373***	2.340***	1.385***	1.363***
	(13.57)	(13.45)	(5.03)	(4.92)	(5.54)	(5.46)	(3.16)	(3.11)
Ind	Control	Control	Control	Control	Control	Control	Control	Control
Year	Control	Control	Control	Control	Control	Control	Control	Control
*N*	5,564	5,564	5,564	5,564	5,564	5,564	5,564	5,564
*R* ^2^	0.158	0.157	0.168	0.168	0.174	0.174	0.187	0.187

t statistics in parentheses *p < 0.1, **p < 0.05, ***p < 0.01.

Table 10 presents the test results of *SonNum*, *GirlNum*, and *Profession*. The regression results of each column show that *SonNum* is always negatively correlated with *Profession.* In terms of the relationship between *GirlNum* and Profession, except in *Column (8)* which is significant at 10% level after all control variables are added, there is no significant correlation between *GirlNum* and *Profession* in other columns.

Table 11 presents the test results of *SonNum*, *GirlNum*, and *Degree*. The regression results of each column show that *GirlNum* has no significant correlation with *Degree*, while *SonNum* and *Degree* show significant negative correlation at the level of ≥5%. According to Tables 10, 11, *SonNum* is more significantly related to professional management than *GirlNum*. This result is mainly due to the fact that the actual controller is influenced by the traditional Chinese concept of male preference, and that sons naturally have better force and deterrence than women (Chen, 2016), which reduces the risk of enterprise inheritance and affects the actual controller in appointing professional managers to important positions.

#### The Moderation Effect of Actual Controller’s Ownership

The strong relationship characteristics of Chinese family enterprises exacerbate the phenomenon of centralization of power (Li and Chen, 2005). The actual controller has the de facto ownership and control of the company, providing an important impact on the company’s governance and decision-making (Wang et al., 2007). Will the ownership proportion of the actual controller have an impact on the relationship between the number of children and professional management? This section discusses this problem.

With the improvement of the degree of marketization, family enterprises are more inclined to have a lasting foundation. The higher the family shareholding, the more consistent the family interests are with the enterprise interests (Shao and Lv, 2015; Liu and Lv, 2016). The inheritance of family enterprises pays more attention to control rather than simply changing leaders. It will have a far-reaching impact on the behavior and performance of family businesses (Sharma and Carney, 2012). In terms of the number of children, Bertrand et al. (2008) found that there is a strong positive correlation between family ownership and the number of enterprise family members. Due to the emotional preference of altruism, the actual controller of the enterprise tends to absorb the relatives of immediate blood relatives (birth relationship) to jointly manage the enterprise (Pan et al., 2019). In terms of the number of sons, with the increase of the number of sons, the number of “available” people in the family and the number of family members with shares in the enterprise are increased. The actual controller can take advantage of the “crowding out” effect of sons on the ownership and control of other family members in the company (Bertrand et al., 2008) to obtain a greater proportion of ownership to improve the influence of their families on the company’s decision-making (Chen, 2011). Therefore, in the process of power transfer, the actual controller has more ownership. On the one hand, the greater the power to act in the way of increasing emotional value gives the owner a greater ability to bring more family members into the enterprise, thereby maintaining family control over it (Demsetz and Lehn, 1985; Schepers et al., 2014; Bauweraerts and Colot, 2017). On the other hand, regarding family reputation, the smaller the impact of nonfamily members on social status and social capital (Xu and Nannan, 2014), the smaller the risk of intergenerational inheritance when inheritance is transmitted within the family. Therefore, to maintain and protect the family’s social emotional wealth, the actual controller increases the number of children and the proportion of sons entering the enterprise, avoiding employing the actual controller to hold important positions.

As far as professional managers are concerned, the degree of marketization in China is low, and the relevant legal system is not perfect (Wang et al., 2019). Family enterprises are still very worried about the moral hazard of professional managers. In addition, the highly centralized power gives family members greater responsibilities and obligations, making family businesses take a very cautious attitude in risky decision-making (Li and He, 2012). Therefore, with the increase of the ownership of the controlling family, family interests are highly related to the listed company, and more attention will be paid to the production and operation activities of the enterprise. Under the same conditions, the possibility of arranging professional managers to take important positions in the listed company will be reduced (Zhang, 2015).

Based on the above analysis, we use *Children* and *SonProp* as independent variables, *Profession* and *Degree* as dependent variables, and adopt hierarchical regression method to test the role of *Ownership*. The results are shown in Tables 12, 13.

**TABLE 12:** The moderation effect of actual controller’s ownership: the number of children of actual controllers and professional management.

	(1)	(2)	(3)	(4)
	Profession	Profession	Degree	Degree
Children	−0.035***	0.060**	−0.037*	0.128***
	(−3.43)	(2.44)	(−1.96)	(2.79)
Ownership		−0.003**		−0.007***
		(−2.54)		(−3.52)
Children * Ownership		−0.002***		−0.003***
		(−3.45)		(−3.08)
Size	0.037***	0.024***	0.023	−0.004
	(4.19)	(2.73)	(1.40)	(−0.26)
Lev	0.097**	0.077*	0.207**	0.166**
	(2.17)	(1.75)	(2.49)	(2.04)
Roa	0.354**	0.408**	0.349	0.457
	(1.96)	(2.30)	(1.04)	(1.39)
Ebit	−1.050***	−0.543*	−2.328***	−1.272**
	(−3.32)	(−1.73)	(−3.95)	(−2.19)
Growth	−0.020	−0.021	−0.029	−0.030
	(−1.40)	(−1.45)	(−1.09)	(−1.12)
Board	0.058	0.032	−0.065	−0.124
	(1.28)	(0.72)	(−0.76)	(−1.49)
Independent board	−0.554***	−0.428***	−1.49***	−1.247***
	(−3.50)	(−2.74)	(−5.05)	(−4.30)
Salary	0.007	0.013	0.045**	0.056***
	(0.64)	(1.14)	(2.13)	(2.73)
Meet	0.032*	0.037*	0.041	0.052
	(1.70)	(2.03)	(1.19)	(1.54)
Education	0.0004	0.001	0.014	0.014
	(0.08)	(0.10)	(1.25)	(1.28)
Sex	0.079***	0.067***	0.152***	0.127***
	(3.03)	(2.63)	(3.14)	(2.67)
Establish	0.0993***	0.077***	0.277***	0.228***
	(5.74)	(4.46)	(8.59)	(7.17)
_Cons	−0.239	0.149	1.345***	2.225***
	(−1.02)	(0.62)	(3.07)	(4.97)
Ind	Control	Control	Control	Control
Year	Control	Control	Control	Control
*N*	5,564	5,564	5,564	5,564
*R* ^2^	0.138	0.168	0.187	0.222

t statistics in parentheses *p < 0.1, **p < 0.05, ***p < 0.01.

**TABLE 13:** The moderation effect of actual controller’s ownership: the proportion of sons of actual controllers and professional management.

	(1)	(2)	(3)	(4)
	Profession	Profession	Degree	Degree
Sonprop	−0.055***	0.010	−0.030	0.058
	(−3.54)	(0.24)	(−1.03)	(0.78)
Ownership		−0.005***		−0.010***
		(−6.15)		(−7.24)
Sonprop * Ownership		−0.002*		−0.003
		(−1.91)		(−1.45)
Size	0.037***	0.023***	0.023	−0.004
	(4.15)	(2.66)	(1.37)	(−0.27)
Lev	0.109**	0.092**	0.218***	0.178**
	(2.45)	(2.08)	(2.62)	(2.19)
Roa	0.336*	0.387**	0.339	0.438
	(1.86)	(2.18)	(1.01)	(1.33)
Ebit	−1.075***	−0.543*	−2.348***	−1.266**
	(−3.40)	(−1.74)	(−3.98)	(−2.18)
Growth	−0.021	−0.019	−0.030	−0.027
	(−1.43)	(−1.34)	(−1.10)	(−1.01)
Board	0.047	0.012	−0.077	−0.148*
	(1.03)	(0.26)	(−0.91)	(−1.79)
Independent board	−0.586***	−0.484***	−1.526***	−1.317***
	(−3.70)	(−3.11)	(−5.17)	(−4.55)
Salary	0.005	0.011	0.043**	0.055***
	(0.40)	(0.97)	(2.04)	(2.68)
Meet	0.030	0.035*	0.040	0.050
	(1.59)	(1.90)	(1.15)	(1.48)
Education	0.004	0.002	0.017	0.013
	(0.59)	(0.28)	(1.52)	(1.21)
Sex	0.087***	0.070***	0.161***	0.125***
	(3.37)	(2.73)	(3.33)	(2.63)
Establish	0.100***	0.073***	0.277***	0.222***
	(5.78)	(4.28)	(8.58)	(7.00)
_Cons	−0.152	0.405*	1.398***	2.551***
	(−0.65)	(1.72)	(3.18)	(5.81)
Ind	Control	Control	Control	Control
Year	Control	Control	Control	Control
*N*	5,564	5,564	5,564	5,564
*R* ^2^	0.138	0.169	0.187	0.221

t statistics in parentheses *p < 0.1, **p < 0.05, ***p < 0.01.

*Column (1)* and *(3)* of Table 12 present the direct effect of *Children*, while *Column (2)* and *(4)* test the influence of *Ownership* on the relationship between *Children* and professional management (*Profession* and *Degree*). After adding the interaction item between the *Children* and *Ownership* (Children * Ownership), there is a significant negative correlation between Children * Ownership, and the professional management (*Profession* and *Degree*) at 1% level. Especially for *Column (2)*, the significant degree of the relationship between *Children* and *Profession* declines after the addition of interaction terms, indicating that *Children* significantly inhibits professional management (*Profession* and *Degree*) when the actual controller considers his ownership.

*Column (1)* and *(3)* in Table 13 present the direct effect of *SonProp*, while *Column (2)* and *(4)* test the influence of *Ownership* on the relationship between *SonProp* on and professional management (*Profession* and *Degree*). As can be seen from *Column (4)*, after the interaction item between the actual controller’s Ownership proportion and the *SonProp* (SonProp * Ownership) is added, the relationship between SonProp * Ownership and *Degree* is not significant. The results in the *Column (2)* show that the relationship between SonProp * Ownership and *Profession* is significantly negatively correlated at the level of 10%, and the significant degree of the relationship between SonProp * Ownership and *Profession* decreases compared with that in *Column (1).* The results show that *SonProp* significantly inhibits professional management (*Profession* and *Degree*) when considering the ownership proportion of actual controllers.

Tables 12, 13 present that the actual controller will consider ownership ratio as one of the factors when making successor selection decisions. Family firms’ decisions are often influenced by the social emotional wealth of family control and influence (Gómez-Mejía et al., 2007; Zellweger and Astrachan, 2008), and family ownership, as a part of social emotional wealth, is positively correlated with the number of family members (Bertrand, 2008). That is, the larger the ownership proportion of actual controllers, the more relatives with direct blood relatives will be recruited to jointly govern firms with the actual controller (Pan et al., 2019). At the same time, with the increase of the proportion of sons, their “crowding out” effect on the ownership of other family members in the company increases (Bertrand, 2008), improving the influence of the actual controller on the company’s decision-making (Chen, 2011). Therefore, in the process of power transfer, the ownership of the actual controller increases, and the greater its power to act in the way of increasing emotional value, giving the owner a greater ability to bring more family members into the enterprise. In order to maintain the family’s control over the enterprise, reduce the risk of intergenerational inheritance of the scepter within the family, and maintain and protect the social emotional wealth of the family and its cautious decision-making attitude (Li and He, 2012), the actual controller will increase the number of children and the proportion of sons entering the enterprise and reduce the employment of professional managers when making decisions. Therefore, with the increase of the ownership proportion of the actual controller, the negative correlation between the characteristics of children and the professional operation of enterprises will be strengthened, and the result is in line with the prediction of our study.

## Conclusion

At present, Chinese family enterprises have successively entered the peak of shift handover. Whether family enterprises choose internal inheritance or external professional managers, previous studies mostly discussed the influencing factors of professional management of family enterprises from the aspects of external institutional environment, professional managers, and family enterprise governance structure, ignoring the impact of the family endogenous factor of the actual controller on the appointment of professional managers. The actual controller of family enterprises, as the decision-maker of enterprises, under the background of the concept of “prosperity of people and prosperity of the country” in Chinese traditional culture, show how the characteristics of children will have an impact on their behavior and concept. Taking Chinese family listed companies as samples, we test the relationship between the characteristics of the children of the actual controller and professional management from two aspects: the number of children and the proportion of sons of the actual controller. It is found that there is a significant negative correlation between the characteristics of children of actual controllers and professional management. Specifically, when there are large numbers of children, on the one hand, the possibility of selecting excellent successors and the degree of absorption of the specific knowledge of the actual controller increases. On the other hand, the altruistic tendency of the family business makes the family members more closely connected, which is conducive to the family members to form a common vision and reduce the risks in the process of business succession. The strengthening of the internal inheritance will of the actual controller inhibits the professional operation of the enterprise. Influenced by the traditional concept of “son inherits father’s business” and male preference, the actual controller will give more consideration to the continuation and inheritance of family business. At the same time, the increase in number of sons makes it easy for the family to establish authority in the enterprise, and the reduction of internal succession risk has a strong inhibitory effect on professional operation. In further research, we found that sons have a more significant impact on professional management than daughters. At the same time, the greater the ownership proportion of the actual controller, the stronger the correlation between children’s characteristics and professional management.

Our conclusion provides evidence for the exploration of the relationship between the characteristics of the actual controller’s children and succession decision-making. Theoretically, on the one hand, it enriches the organization theory in the context of family business, expands the theoretical gap of the logical relationship between the actual controller’s family population structure and enterprise succession decision-making, and enriches the research on the antecedent variables of successor identity. Different from the previous research on the exogenous factors affecting professional operation, by establishing the relationship between the characteristics of the children of the actual controller and the enterprise succession decision, we expand the research on the influencing factors of family business succession from the perspective of family, which is helpful to deeply understand the influence of the endogenous factors of family business on the succession decision. On the other hand, in the further research, we will examine the regulatory effect of the actual controller’s shareholding ratio to enrich the relevant literature of family business holding and help deeply understand the impact of the actual controller’s family on the succession of family business. The practical significance of our study is that although the “family” management model may interfere with the effective governance of the company from a rational perspective, the starting point of the actual controller’s decision-making will still be affected by the traditional Chinese “family” culture. The succession process of family business is synchronized with the development of business family. It runs through the whole process, from the actual controller of the enterprise to the completion of the handover of leadership. Therefore, combined with the governance model of family enterprises, our study analyzes the impact of the characteristics of the children of the actual controller on professional operation, which is of significant reference for family enterprises to make succession planning at the peak of intergenerational inheritance, and helps to establish a modern enterprise system, promotes the benign evolution of enterprises, builds a harmonious “collaborative symbiosis system” of family governance and corporate governance, and promotes the healthy and sustainable development of private enterprises.

## Data Availability Statement

The datasets presented in this study can be found in online repositories. The names of the repository/repositories and accession number(s) can be found in the article/Supplementary Material.

## Author Contributions

XS designed the manuscript, collected the data, summarized the literature review, developed the hypotheses, conducted the empirical analysis, and finalized the manuscript. ZT contributed to the hypothesis development and performed the empirical analysis. XZ provided valuable suggestions and comments regarding the manuscript. All authors contributed to the article and approved the submitted version.

## Conflict of Interest

The authors declare that the research was conducted in the absence of any commercial or financial relationships that could be construed as a potential conflict of interest.qe

## Publisher’s Note

All claims expressed in this article are solely those of the authors and do not necessarily represent those of their affiliated organizations, or those of the publisher, the editors and the reviewers. Any product that may be evaluated in this article, or claim that may be made by its manufacturer, is not guaranteed or endorsed by the publisher.
