# Supplementary material for: Will the “Prosperity of the Population and Son” Affect the Hiring of the “Shopkeeper”?—Research on Succession Decisions of Actual Controllers in Chinese Family Firms
Source: Front Psychol. 2022 May 13;13:825223. doi: 10.3389/fpsyg.2022.825223 (PMC9138618; doi:10.3389/fpsyg.2022.825223)
Supplement: Supplementary file 1 [file Data_Sheet_1.PDF]

Profession	Degree	Children	Sonprop	Size	Lev	Roa	Ebit	Growth
1	1	2	0	20.48714	0.624158	0.022752	0.001474	0.891048
1	3	1	1	20.25586	0.100605	0.048377	0.009202	-0.08155
1	3	1	1	20.32878	0.112276	0.02892	0.010273	0.008977
1	3	1	1	22.00124	0.189809	0.066684	0.019288	3.215999
0	0	1	1	20.44458	0.667058	0.024893	0.008542	1.683524
0	0	1	1	20.40292	0.632778	0.042135	0.017869	0.466919
0	0	1	1	20.32038	0.58566	0.050145	0.008772	0.174627
0	0	1	1	20.34002	0.588963	0.038587	0.015374	-0.17343
0	0	1	1	20.4104	0.62581	0.01895	0.009379	-0.1341
0	0	1	1	20.87405	0.758028	0.04337	0.007841	0.108514
0	0	1	1	20.86736	0.72819	0.041817	0.014524	-0.28119
0	0	1	1	20.26515	0.494528	0.021654	0.004389	0.239685
0	0	1	1	20.26085	0.490804	0.018277	0.005207	0.385796
0	0	1	1	20.24053	0.475007	0.02416	0.00803	-0.25756
0	0	1	1	20.23577	0.463615	0.028816	0.007225	0.13266
1	1	1	0	23.84743	0.602118	0.025816	0.000818	0.675884
1	1	1	0	23.82991	0.626553	0.012769	0.006158	-0.31688
1	1	1	0	23.87759	0.638528	0.01632	-0.0008	0.232322
1	1	1	0	24.15515	0.710283	0.040625	-0.00113	1.219713
1	1	1	0	24.43043	0.763096	0.041029	0.004484	0.335652
1	3	1	0	24.98438	0.823456	0.037289	0.003151	0.021046
1	1	1	0	25.49696	0.87189	0.040684	0.007472	0.558338
1	1	1	0	25.84625	0.851677	0.038307	0.005542	0.971084
1	1	1	0	25.95851	0.85357	0.033749	0.006394	-0.54973
1	1	1	0	26.08031	0.865957	0.017287	0.011429	-0.40281
1	1	1	0	25.90433	0.813872	0.029613	0.019015	-0.3958
0	0	2	0	20.91532	0.592418	0.041304	0.02145	0.09292
0	0	2	0	21.00887	0.636463	0.015466	0.002291	0.077031
0	0	2	0	21.40785	0.695063	0.087326	0.005409	0.369964
0	0	2	0	21.38637	0.58659	0.132294	0.005663	0.073496
0	0	2	0	21.39608	0.578484	0.011431	0.00615	-0.3218
0	0	2	0	22.08547	0.818492	-0.04371	-0.01081	-0.00744
1	3	3	0.333333	25.29742	0.596883	0.109092	0.0902	0.1791
1	3	3	0.333333	25.51319	0.61983	0.118395	0.02998	0.171053
1	3	3	0.333333	25.58185	0.565113	0.12566	0.034038	-0.02278
1	3	3	0.333333	25.86259	0.595683	0.104974	0.031897	0.14882
1	3	3	0.333333	26.23713	0.665768	0.091375	0.023931	0.513495
1	3	3	0.333333	26.29808	0.649397	0.090823	0.027261	0.078735
1	3	3	0.333333	26.43355	0.644	0.091727	0.025951	0.071443
1	3	3	0.333333	22.34168	0.465132	0.042472	0.015642	0.444625
0	0	2	0	20.17295	0.664357	0.02949	0.002824	4.792161
1	3	2	0.5	20.70256	0.241461	0.097244	0.108011	0.000902
1	3	2	0.5	20.71567	0.232765	0.033586	0.000906	0.125307
1	3	2	0.5	20.61508	0.121643	0.067709	0.029445	-0.03063
1	3	2	0.5	20.68416	0.118874	0.093776	-0.0008	-0.29224
1	3	2	0.5	20.65641	0.119955	0.017597	0.030339	-0.00717
1	3	2	0.5	20.65658	0.049781	0.133773	-0.00436	-0.10827
1	3	3	0.333333	22.07875	0.506345	0.071957	0.013257	0.019161
1	3	3	0.333333	22.8029	0.561231	0.094421	0.021784	1.560398
1	3	3	0.333333	22.93649	0.566435	0.073192	0.022072	-0.02024
1	3	3	0.333333	22.85194	0.494206	0.052658	0.017752	-0.37135
1	3	1	1	22.94491	0.508342	0.062156	0.017387	0.264946
1	3	1	1	23.15484	0.54779	0.080791	0.023579	0.237886
1	3	3	0.333333	23.38546	0.582086	0.077843	0.027063	0.215417
1	3	3	0.333333	23.66169	0.631521	0.075696	0.026557	0.243937
1	3	3	0.333333	23.78378	0.614859	0.093394	0.027149	0.309141
1	3	3	0.333333	23.8829	0.586257	0.082652	0.030923	0.10532

0	0	3	0.333333	21.86776	0.486809	0.069046	0.00401	-0.03984
0	0	3	0.333333	21.9249	0.487297	0.066882	0.009528	1.315712
0	0	3	0.333333	22.17203	0.358713	0.033405	0.008753	-0.06512
0	0	3	0.333333	22.09265	0.299452	0.048967	0.028834	0.402081
0	0	3	0.333333	22.12795	0.337222	0.003456	0.005727	-0.28435
0	0	3	0.333333	23.00184	0.501488	0.020847	0.001154	0.043384
0	0	3	0.333333	22.95665	0.423948	0.100508	0.017126	1.438969
0	0	3	0.333333	22.91307	0.427777	0.033149	0.021941	0.155136
0	0	3	0.333333	22.94998	0.420149	0.032335	0.001371	-0.6418
1	1	1	1	20.15258	0.382128	0.082742	0.005156	-0.01663
1	1	1	1	19.55112	0.210434	-0.00118	-0.00627	-0.13091
1	1	1	1	19.41655	0.167579	0.014793	0.018579	1.107722
1	1	1	1	19.81866	0.475371	-0.06009	-0.01003	-0.67685
1	3	1	1	19.84738	0.472495	0.177711	-0.02022	-0.21064
1	3	1	1	19.32438	0.149953	-0.04545	-0.00118	-0.06291
1	2	2	1	21.20812	0.192789	0.011546	-0.00153	-0.04937
1	2	2	1	21.1646	0.166907	0.00626	0.010821	-0.27425
1	3	1	1	21.96026	0.558298	0.048031	0.006471	-0.04601
1	2	1	1	22.02856	0.576666	0.016407	-0.00125	-0.2859
1	2	1	1	21.99133	0.564651	0.005599	-0.00154	0.210918
0	0	1	1	22.09464	0.585663	0.02209	0.002006	0.332023
0	0	1	1	21.94566	0.507358	0.019313	0.001237	-0.12812
0	0	2	1	21.8625	0.287711	0.192875	0.016561	0.260952
0	0	2	1	22.02127	0.279313	0.145615	0.008844	0.050416
0	0	2	1	22.24995	0.345714	0.096119	0.012838	0.159971
0	0	2	1	22.45204	0.435524	0.096417	0.029905	0.246801
1	1	2	1	22.60517	0.451033	0.093491	0.028334	0.171206
1	1	2	1	22.7115	0.440964	0.092112	0.025792	0.200394
1	3	1	1	21.61113	0.611367	0.056636	-0.00156	0.133854
1	3	1	1	21.71905	0.605198	0.085533	0.016324	0.090801
1	3	1	1	21.86912	0.644467	0.067775	-0.00095	0.338022
1	3	1	1	22.01348	0.671566	0.061804	0.024658	0.007257
1	3	1	1	22.00743	0.644833	0.060977	0.020856	0.047913
1	3	1	1	21.97981	0.545332	0.136976	0.031439	0.089092
1	3	1	1	22.39349	0.373586	0.063169	0.037328	0.004383
1	3	1	1	22.43802	0.370194	0.049983	0.016569	0.007772
1	3	1	1	22.48673	0.377534	0.047733	0.016646	0.032773
1	3	1	1	23.02895	0.501937	-0.0341	0.004874	-0.51408
1	3	2	1	19.07335	0.221908	0.004189	-0.00739	1.470116
1	3	2	1	19.07558	0.184769	0.046312	-0.00868	2.731665
1	3	3	0.333333	24.17824	0.597952	0.093983	0.014585	0.04336
1	3	3	0.333333	24.46222	0.610437	0.129054	0.005615	0.577023
1	3	3	0.333333	24.81008	0.57753	0.111032	0.021508	0.248786
1	3	3	0.333333	24.83249	0.55017	0.088656	-0.00136	-0.2689
1	3	1	1	22.06669	0.497427	0.067654	0.010907	0.170669
1	3	1	1	22.08588	0.483665	0.068055	0.014206	0.686157
1	3	1	1	22.15005	0.49158	0.062539	0.021584	0.06003
1	1	1	0	22.5617	0.801839	0.094354	0.026334	0.795933
1	1	1	0	23.24063	0.848338	0.061417	0.02263	1.034919
1	1	1	0	23.61424	0.863577	0.041182	0.007504	0.000784
1	1	1	0	23.97745	0.89857	0.027359	0.011135	0.0672
1	1	1	0	24.31922	0.899756	0.045414	0.015927	1.131605
1	1	1	0	24.49361	0.824051	0.051603	0.011817	0.5035
1	1	1	0	24.73785	0.766098	0.055665	0.015806	0.350735
1	1	1	0	24.98819	0.781105	0.060767	0.023911	0.264814
1	1	1	0	25.41006	0.824242	0.041149	0.021621	-0.18844
1	1	1	0	25.3478	0.806701	0.042521	0.011313	-0.10515
1	1	1	0	25.50937	0.826794	0.027016	0.014578	-0.11002

0	0	1	1	19.29052	0.963965	-0.00027	0.004883	-0.3103
1	1	2	0.5	19.8627	0.573679	0.031326	0.063024	-0.32376
1	1	2	0.5	19.48569	0.301097	0.104823	-0.0031	-0.24685
1	1	2	0.5	22.13172	0.581223	0.086115	-0.00981	-0.1373
1	1	2	0.5	22.19899	0.543304	0.098347	-0.00429	0.358047
1	1	2	0.5	22.35641	0.544929	0.100677	0.001026	0.160219
1	1	2	0.5	22.80153	0.516107	0.080083	-0.0046	1.344347
1	2	2	0.5	22.85332	0.493778	0.087673	0.007542	0.571742
0	0	2	0.5	22.93711	0.464441	0.091333	0.007521	0.024728
1	1	1	0	21.65726	0.73576	0.074414	-0.00339	0.301687
1	1	1	0	22.04771	0.76416	0.064713	0.01723	0.625752
1	1	1	0	22.25414	0.781555	0.019982	-0.0002	-0.45536
1	1	1	0	22.49446	0.806471	0.012905	-0.00282	0.183576
1	1	1	0	22.55805	0.843875	0.024512	0.004874	0.782176
1	1	1	0	22.26614	0.6261	0.048639	-0.0046	0.774199
1	1	1	0	21.65735	0.45875	0.062486	0.024162	0.503182
1	1	1	0	21.73596	0.509575	0.006196	0.014321	-0.46953
1	1	1	1	22.48275	0.676859	0.080539	0.021027	0.148836
1	1	1	1	22.81248	0.499961	0.078397	0.064785	0.403919
1	1	1	1	22.89853	0.531791	0.072471	0.060504	0.045242
1	1	1	1	22.87075	0.535614	0.070658	0.048627	0.020353
1	1	1	1	22.99978	0.605592	0.087652	0.017123	0.110596
1	1	1	1	23.20007	0.633816	0.089438	0.026397	0.061186
1	1	1	1	23.26855	0.640637	0.083629	0.024545	0.041974
1	1	1	1	23.16715	0.589862	0.096652	0.023995	0.053155
1	3	1	1	23.16816	0.532298	0.095399	0.02634	0.034158
1	3	1	1	23.2122	0.546613	0.07298	0.026567	0.018633
1	3	1	1	23.21052	0.54705	0.053781	0.022276	-0.06874
1	1	1	0	21.9275	0.305101	0.065604	0.012335	-0.0253
1	1	1	0	22.21985	0.354891	0.029219	0.013452	-0.07587
1	1	1	0	22.30929	0.410649	0.017236	0.004566	-0.03675
1	3	3	1	21.66332	0.448261	0.02207	0.003657	0.485247
1	3	3	1	21.65427	0.437837	0.039276	0.008919	0.065389
1	3	3	1	21.64403	0.410128	0.046441	0.001308	0.56555
1	3	3	1	21.67887	0.423265	0.050826	0.000347	-0.03646
1	3	3	1	21.79668	0.450208	0.062287	0.007424	0.287585
1	3	3	1	21.92386	0.513962	0.020266	0.007037	-0.15237
1	3	3	1	21.88226	0.52116	-0.01813	0.000111	-0.64432
1	3	3	1	21.73866	0.377309	0.087648	0.00687	2.635199
1	3	3	1	21.80139	0.415724	0.016007	0.003153	-0.49728
1	3	2	0.5	21.11315	0.310645	0.031812	0.009636	-0.03822
1	1	2	0.5	21.09913	0.294006	0.011509	0.002324	0.055392
1	1	2	0.5	21.39525	0.384404	0.134775	-0.00509	-0.16375
1	1	2	0.5	21.17846	0.170435	0.107768	-0.0033	0.188519
1	3	2	0.5	21.23343	0.311863	0.068558	0.003042	-0.13849
1	3	2	0.5	21.18425	0.277351	0.083901	0.02128	0.20862
1	3	1	1	21.68175	0.340888	0.023904	0.033905	-0.28714
1	3	1	1	21.97609	0.463466	0.06289	0.014293	0.703862
1	3	1	1	22.02954	0.494543	0.002991	0.013493	-0.17401
1	3	1	1	22.03785	0.507186	0.011816	-0.00866	0.34724
1	3	1	1	22.00131	0.373958	0.150877	0.04734	0.780371
1	3	1	1	21.97341	0.402859	0.070772	0.020742	-0.2719
1	1	1	1	21.8066	0.296732	0.009074	0.018068	-0.4661
1	1	2	0.5	21.39017	0.461605	0.138879	-0.00411	1.551796
1	3	1	1	20.77743	0.738589	-0.0854	-0.00164	-0.1306
1	3	1	1	20.76827	0.720412	0.062063	-0.00129	0.369751
1	3	1	1	20.90606	0.448156	-0.06372	-0.00203	0.359853
1	3	1	1	21.32682	0.616965	-0.02954	0.008	-0.0079

1	3	1	1	21.76747	0.068488	0.157205	0.024223	-0.16736
1	3	1	1	22.16293	0.305247	0.145701	0.006319	0.943738
1	3	1	1	22.03401	0.420842	0.171485	0.029904	0.800268
1	3	1	1	22.19296	0.387562	0.172941	0.04273	0.207704
1	3	1	1	21.46794	0.631538	0.009898	0.012959	-0.31678
1	3	1	1	21.20028	0.727613	-0.08516	0.002296	-0.38901
1	3	1	1	21.424	0.726068	0.046542	0.008749	0.045364
1	3	1	1	21.39361	0.703136	0.045823	-0.00017	0.273247
1	1	2	0	21.4914	0.269647	0.03735	0.004652	0.06084
1	1	2	0	21.4045	0.189368	0.02877	0.012429	0.307439
1	3	2	0	21.35646	0.249239	-0.05094	0.002556	-0.10102
1	3	2	0	21.44826	0.255418	0.020656	-0.01608	-0.31226
1	3	2	0	21.49196	0.334133	-0.0449	-7.3E-05	0.241382
1	3	2	0	21.46924	0.293458	0.065443	0.117368	-0.16686
1	1	2	0	21.60257	0.259628	0.148221	-0.00702	-0.32902
1	3	1	1	22.69973	0.818693	0.060273	0.007367	1.9872
1	3	1	1	22.97801	0.816424	0.062685	0.014532	0.166386
1	3	1	1	23.08923	0.786991	0.06635	0.003756	0.144005
1	3	1	1	23.12819	0.746061	0.075021	0.006	0.282488
1	3	1	1	23.27916	0.766583	0.059713	0.003311	0.015161
1	3	1	1	23.20445	0.704834	0.070627	0.016697	0.747427
1	3	1	1	23.19912	0.68359	0.037986	0.018544	-0.29199
1	3	1	1	23.53504	0.662786	0.032189	0.005735	0.046401
1	3	1	1	23.34003	0.553553	0.077802	0.006184	0.837994
0	0	1	1	19.04556	0.265613	0.025111	-0.00403	-0.33785
1	1	1	1	20.61754	0.106485	0.120817	-2E-06	0.1951
1	1	1	1	21.06659	0.347867	0.095827	0.026237	0.530099
1	1	1	1	21.08153	0.284061	0.135178	0.021371	0.347674
1	1	1	1	21.08537	0.193606	0.148031	0.028046	-0.02142
1	1	1	1	21.34363	0.344343	0.077281	0.021582	-0.22947
1	1	1	1	21.27857	0.219564	0.101551	0.032486	0.199676
1	1	1	1	22.84387	0.695448	0.03209	0.028556	0.504257
1	1	1	1	22.76538	0.64059	0.09366	0.032101	0.664584
1	1	1	1	23.05344	0.51104	0.075258	0.017809	0.04035
1	1	1	1	20.78237	0.192029	0.199917	0.057089	0.121256
1	1	1	1	21.11408	0.382993	0.193925	0.067296	0.390791
1	1	1	1	21.46276	0.182733	0.162846	0.08016	-0.05444
1	3	1	1	21.56858	0.167416	0.117699	0.046754	-0.13803
1	3	1	1	21.67766	0.167517	0.161441	0.031577	0.252219
1	3	1	1	21.87266	0.175971	0.17944	0.040271	0.120109
1	3	1	1	22.0752	0.170875	0.144427	0.041685	0.413352
1	3	1	1	22.15056	0.166758	0.133355	0.032718	0.077569
1	3	1	1	22.25294	0.151987	0.156768	0.033124	0.1456
1	3	1	1	22.32958	0.126889	0.157971	0.041587	0.040366
0	0	2	0	24.34368	0.855081	0.037624	0.003408	0.7394
0	0	2	0	24.6748	0.846984	0.03251	-0.00176	0.048944
1	1	2	0	24.85675	0.840893	0.021668	0.008627	0.552822
1	1	2	0	25.13079	0.839565	0.011167	0.004773	0.078029
1	1	2	0	25.28294	0.83948	0.021485	0.001331	0.119783
1	3	2	0	25.4169	0.793754	0.023221	0.006295	0.661743
1	3	2	0	25.78183	0.857869	0.0217	0.001253	0.078242
1	3	2	0	26.16438	0.836295	0.022799	0.002877	0.18632
1	3	2	0	26.49659	0.837841	0.027872	0.001914	0.643641
1	1	2	0	22.86485	0.40591	0.045834	-0.00026	-0.13726
1	1	2	0	22.98742	0.474821	0.034982	0.000216	1.200835
1	1	2	0	23.11107	0.502322	0.024027	0.000234	-0.07255
1	1	2	0	23.29154	0.570583	0.022009	0.004623	-0.02256
1	1	2	0	23.43667	0.630405	0.011804	0.002321	0.955153

0	0	2	0	23.4907	0.645221	0.014189	0.001723	-0.39244
1	1	2	0	23.58287	0.652122	0.038396	0.001315	1.222326
1	1	2	0	23.6245	0.624281	0.063212	0.009693	0.112373
1	1	2	0	23.57078	0.564867	0.059015	0.007118	-0.1472
1	1	2	0	23.86303	0.662576	0.020718	-0.00587	-0.43214
1	1	2	0	24.03822	0.736813	-0.00053	-0.00361	0.451926
0	0	2	0.5	20.67366	0.217192	0.090438	0.008234	-0.52815
1	1	2	0.5	20.66282	0.213661	0.004864	0.004179	-0.42816
1	1	2	0.5	20.83246	0.333519	0.028917	-0.0017	-0.26998
1	1	2	0.5	21.14277	0.508794	0.027672	0.009401	0.688285
1	1	2	0.5	21.08876	0.494807	0.039878	-0.0012	8.851063
1	1	2	0.5	21.75975	0.659968	0.010278	-0.00034	-0.07971
1	1	2	0.5	21.72152	0.594564	0.041439	0.004743	0.685797
1	3	1	1	20.55455	0.229517	0.022568	0.007216	0.140321
1	3	1	1	20.58712	0.246866	0.028151	0.003226	0.275136
1	3	2	0.5	22.66075	0.773267	0.020751	0.008497	-0.31016
1	3	2	0.5	22.5222	0.75439	0.029752	0.006814	-0.04362
1	3	2	0.5	22.44384	0.815167	-0.03334	0.00577	-0.40998
1	3	2	0.5	22.22888	0.850198	-0.01478	-0.00227	-0.28669
1	3	1	1	21.14441	0.092627	0.105382	-0.02287	2.707529
1	3	1	1	21.74216	0.237634	0.076047	0.015283	0.388171
1	3	1	1	21.98274	0.337898	0.084972	0.010125	0.35475
1	3	1	1	22.13412	0.364926	0.09477	0.012023	0.10751
1	3	1	1	22.16547	0.312123	0.098847	0.016251	0.21271
0	0	1	1	22.13843	0.253447	0.069123	0.016132	-0.269
1	1	2	0	21.37657	0.222466	0.187126	0.013689	0.105105
1	3	2	0	21.39477	0.094674	0.148011	0.009009	0.021694
1	3	2	0	21.58905	0.077885	0.208603	0.051273	0.113199
0	0	1	1	21.96305	0.725851	0.0581	0.010746	0.202764
0	0	1	1	22.08276	0.763317	0.048409	0.000564	0.140966
0	0	1	1	22.08952	0.693056	0.055272	0.010765	-0.00335
0	0	1	1	22.11737	0.660946	0.079294	0.013749	0.254618
0	0	1	1	22.08111	0.599532	0.082734	0.013847	0.177224
0	0	1	1	22.09154	0.64609	0.092091	0.026534	0.170369
0	0	1	1	22.24009	0.470709	0.086673	0.023882	-0.07629
0	0	1	1	22.50403	0.477059	0.047595	0.020911	0.01743
0	0	1	1	22.74249	0.64302	0.034222	0.013219	0.360113
0	0	1	1	22.72442	0.685327	0.020239	0.00691	0.144119
0	0	2	0.5	22.78323	0.696701	0.037709	0.016958	0.105501
1	1	1	1	23.83322	0.678552	0.145402	-0.00283	0.6949
1	1	1	1	23.77918	0.671032	0.053718	0.034305	0.036767
1	1	1	1	23.89318	0.690292	0.022271	0.002765	-0.0588
1	1	1	1	24.00915	0.737606	0.003053	-0.00038	-0.08742
1	3	1	1	23.95046	0.669643	0.045801	0.007835	0.080362
1	3	1	1	24.0387	0.52011	0.051945	0.010388	0.06932
1	3	1	1	24.30132	0.506781	0.065831	0.021266	1.034573
1	3	1	1	24.81134	0.626552	0.056442	0.025894	0.287876
1	3	1	1	25.16863	0.659003	0.067848	0.012449	-0.06271
1	3	1	1	20.51607	0.544582	0.024391	-0.00048	0.06427
1	3	1	1	20.48991	0.523463	0.030823	-0.0001	0.144442
1	3	1	1	20.51301	0.556543	0.05168	0.005925	-0.0752
1	3	1	1	20.47243	0.538058	0.029755	-0.00079	0.033765
1	3	1	1	20.8153	0.642154	0.004165	0.007369	0.03171
0	0	1	1	22.93582	0.667191	0.118815	-0.00011	0.222067
0	0	1	1	23.44534	0.753112	0.077746	0.023031	0.243584
0	0	1	1	23.57688	0.74768	0.064242	0.000035	0.054997
0	0	1	1	23.67496	0.730568	0.062002	0.015845	0.202375
0	0	1	1	23.75325	0.79086	0.045492	0.023876	0.525275

0	0	1	1	23.78554	0.771962	0.057649	0.00224	-0.18807
0	0	1	1	23.93345	0.614116	0.058428	0.002897	0.351607
0	0	1	1	23.77693	0.611588	0.075331	0.003958	0.118145
0	0	1	1	23.7041	0.540393	0.080272	0.003682	-0.30056
0	0	1	1	23.66177	0.529465	0.07605	0.019339	-0.43762
0	0	1	1	23.61242	0.526626	0.098041	0.013751	0.209782
1	3	2	0.5	20.71605	0.49161	0.010119	0.00378	-0.49232
1	3	2	0.5	20.64405	0.457779	0.018319	0.010283	0.574238
1	3	2	0.5	20.65026	0.434366	0.041652	0.010582	0.119384
1	3	2	0.5	20.47935	0.390669	-0.05198	0.000271	-0.22074
1	3	2	0.5	20.60762	0.446698	0.065439	0.033385	0.058839
1	2	2	0.5	20.54509	0.401394	0.02234	0.016599	-0.20996
1	2	2	0.5	23.70083	0.527545	0.088015	0.033973	-0.06975
1	1	1	0	22.46843	0.228451	0.057175	0.013915	0.789719
1	1	1	0	22.51454	0.208321	0.074623	0.012679	0.376716
0	0	1	1	21.00012	0.144686	0.000619	-0.0024	-0.24922
0	0	1	1	21.07507	0.184741	0.029455	0.000547	0.338303
0	0	1	1	21.19604	0.267277	0.00789	0.001792	0.28566
0	0	1	1	21.10243	0.270842	-0.05185	-0.01018	-0.22646
1	3	2	0.5	20.31421	0.384119	0.10329	0.028947	-0.33332
1	3	2	0.5	20.34672	0.35992	0.081954	0.014132	0.228967
1	3	2	0.5	20.32726	0.328812	0.096565	0.028504	0.242584
1	3	2	0.5	20.29285	0.346197	0.011427	-0.00214	-0.17293
1	3	2	0.5	20.12273	0.230338	0.051646	0.026608	-0.12912
1	1	1	1	19.92746	0.084128	0.027381	0.030356	-0.54313
1	1	1	1	21.11204	0.766015	0.030527	-0.00445	-0.20375
1	1	1	1	21.04085	0.741976	-0.05902	-0.01073	-0.08595
1	1	1	1	21.15155	0.674912	0.14552	0.021597	-0.12777
1	1	1	0	21.37973	0.494387	0.046441	-0.00061	0.493456
1	1	1	0	21.64342	0.615841	0.02251	-0.00019	0.100567
1	1	1	0	21.4644	0.542445	0.011157	-0.00326	0.400582
1	1	1	0	21.5789	0.574331	0.059808	-0.00597	0.074476
1	1	1	0	21.62172	0.573919	0.043629	0.036087	-0.14933
1	1	1	0	21.54884	0.623431	-0.04533	-0.01043	-0.08868
1	1	1	0	21.93197	0.442136	0.068342	-0.00536	0.597198
1	3	1	1	21.43433	0.81178	0.040573	-0.00295	0.589599
1	3	1	1	21.71365	0.826006	0.038594	0.012457	0.343036
1	3	1	1	22.37578	0.696565	0.012498	-0.00572	0.121044
1	3	1	1	22.39158	0.685002	0.028781	0.005217	1.569466
1	3	1	1	22.87686	0.781799	0.024707	0.002407	0.050925
1	3	1	1	23.24698	0.841467	0.013921	0.000998	0.689992
1	3	1	1	23.5963	0.840901	0.007576	-0.00176	0.113613
1	3	1	1	20.8341	0.405371	0.173747	0.065129	-0.12402
1	3	1	1	21.03803	0.442136	0.172887	0.11877	0.3671
1	3	1	1	21.12709	0.481621	0.1687	0.106219	0.066543
1	3	1	1	21.15981	0.469115	0.197263	0.108348	0.103502
1	2	1	1	21.85348	0.351975	0.189944	0.14089	-0.06849
1	2	1	1	21.75703	0.276861	0.1926	0.104692	-0.16225
0	0	1	1	21.76807	0.311757	0.183913	0.105741	0.004779
1	3	1	1	21.85644	0.346	0.192033	0.111641	0.062879
1	1	1	0	22.8582	0.453141	0.08574	0.021026	-0.0641
1	1	1	0	23.80009	0.429759	0.185304	0.030317	0.348165
1	1	1	0	23.93002	0.413441	0.115023	0.000474	0.02231
1	1	1	0	24.10885	0.432343	0.112064	0.027945	-0.05247
1	1	1	0	24.23186	0.344664	0.099564	0.01822	0.008891
1	1	1	0	24.2848	0.314649	0.099163	0.025239	-0.1213
1	1	1	0	24.34454	0.316596	0.093423	0.026826	-0.01041
1	1	1	0	24.47163	0.38201	0.082717	0.022859	0.027716

1	1	1	0	24.5933	0.429838	0.069497	0.022496	0.103831
1	1	1	0	24.88554	0.491575	0.106899	0.022996	0.18805
0	0	2	0.5	21.28864	0.476566	0.162552	0.036777	0.437653
0	0	2	0.5	21.57893	0.511038	0.185411	0.081336	0.711137
0	0	2	0.5	21.83688	0.49899	0.154591	0.044678	0.210388
0	0	2	0.5	22.08602	0.495446	0.180415	0.042234	0.103469
0	0	2	0.5	22.14436	0.447296	0.139248	0.043926	0.235283
0	0	2	0.5	22.41451	0.433492	0.139794	0.038372	0.211091
0	0	2	0.5	22.73431	0.491278	0.132239	0.040641	0.298181
0	0	2	0.5	23.19688	0.441631	0.097746	0.038246	0.28141
0	0	2	0.5	23.45429	0.494019	0.102395	0.029663	0.40386
0	0	2	0.5	23.50047	0.476338	0.085956	0.031589	0.050751
0	0	2	0.5	23.62057	0.505901	0.048559	0.024183	-0.0535
1	1	2	0.5	22.31761	0.308152	0.073545	0.004885	-0.15234
0	0	1	0	21.37236	0.480595	0.174705	-0.01964	0.379
0	0	1	0	21.95951	0.346886	0.139851	0.023033	0.256259
0	0	1	0	22.35915	0.491163	0.104297	0.016315	0.073795
0	0	1	0	22.30611	0.466004	0.07025	0.013692	-0.02153
0	0	1	0	22.37713	0.452455	0.062334	0.011648	0.000791
1	1	3	0.666667	22.779	0.678719	0.073391	0.010653	0.125022
1	1	3	0.666667	22.87539	0.676589	0.073399	0.006776	0.076832
1	1	3	0.666667	22.92951	0.685593	0.063193	0.014395	0.056632
1	1	3	0.666667	22.89691	0.663305	0.068347	0.012411	0.13673
1	1	3	0.666667	22.8932	0.640861	0.064095	0.011016	-0.01476
1	1	3	0.666667	22.82605	0.623737	0.066942	0.010928	0.030511
1	1	3	0.666667	22.59128	0.625825	0.09679	0.01351	-0.0904
1	1	3	0.666667	22.50356	0.489455	0.123439	0.011259	-0.1492
1	1	3	0.666667	22.57043	0.42134	0.14345	0.012408	0.079138
1	1	3	0.666667	22.66024	0.351712	0.130925	0.014968	0.030224
1	1	3	0.666667	22.80319	0.3426	0.107636	0.013892	0.005058
1	3	2	0.5	22.08894	0.355843	0.033939	0.008419	0.202711
1	3	2	0.5	22.19721	0.423726	0.003808	0.015948	0.137238
1	3	2	0.5	22.27354	0.432672	0.046047	0.010639	0.225376
0	0	2	0	23.56472	0.767117	0.050591	0.006634	0.633791
0	0	2	0	23.90697	0.781472	0.046315	0.015312	0.607519
0	0	2	0	24.23684	0.794709	0.050934	0.01123	0.34641
0	0	2	0	24.59109	0.828624	0.043792	0.00896	0.059476
0	0	2	0	24.89938	0.848865	0.039285	0.009561	0.405986
0	0	1	0	25.15741	0.847284	0.022377	0.00521	0.189109
0	0	1	0	25.28997	0.86267	0.010558	0.002447	-0.0616
0	0	1	0	25.4866	0.862251	0.008153	0.002663	0.684116
0	0	1	0	25.88808	0.905155	0.007213	0.00221	-0.11287
0	0	1	0	26.1858	0.916869	0.014704	0.004295	0.312834
0	0	1	0	26.39511	0.90769	0.023505	0.003652	0.790839
1	3	1	0	22.48863	0.692913	0.122857	0.031809	0.240702
1	3	1	0	22.60101	0.641817	0.141285	0.033021	0.309742
1	3	1	0	22.76103	0.607066	0.138623	0.047358	0.146699
1	3	1	0	22.92129	0.720374	0.147317	0.042989	0.133353
1	3	1	0	23.15838	0.715924	0.1424	0.047148	0.146722
1	3	1	0	23.39441	0.471388	0.136712	0.042485	0.168096
1	3	1	0	23.49505	0.449002	0.148361	0.045824	0.096619
1	3	1	0	23.67908	0.457494	0.153977	0.049185	0.101738
1	3	1	0	23.78964	0.400548	0.161794	0.054137	0.155962
1	3	1	0	22.2285	0.207553	0.027616	0.006578	1.810062
1	3	1	0	22.32454	0.175341	0.118713	0.008594	-0.06661
1	3	1	0	22.34928	0.156804	0.042561	0.006767	-0.00398
1	3	1	0	22.59306	0.288905	0.064559	0.006812	-0.03417
1	1	1	1	23.93072	0.780558	0.029553	0.004852	0.339871

1	1	1	1	23.94963	0.739191	0.069637	0.006143	-0.04753
1	1	1	1	21.25673	0.790466	0.074733	0.011633	0.026148
1	1	1	1	21.52276	0.808854	0.072958	0.012648	0.538367
0	0	1	1	22.01518	0.770045	0.083984	0.015359	0.537358
0	0	1	1	22.34317	0.660087	0.099978	0.026029	0.340759
0	0	1	1	22.86785	0.769353	0.066201	0.023727	0.282182
0	0	1	0	22.22084	0.152761	0.120726	0.021676	-0.37986
1	1	1	0	22.29142	0.137648	0.155665	0.033067	2.067997
1	1	1	0	22.49375	0.233379	0.139952	0.031392	0.420157
1	1	1	0	22.3811	0.178599	0.074451	0.027827	-0.16726
1	1	1	0	22.31347	0.18614	0.053681	0.01844	0.006826
1	1	1	0	21.64378	0.269721	0.181785	0.002092	0.037801
1	1	1	0	21.93928	0.3661	0.125231	0.043258	0.131371
1	1	1	0	22.08207	0.475279	0.113348	0.003157	0.162721
1	1	1	0	22.24484	0.349727	0.096904	0.005335	-0.07531
1	1	1	0	22.30813	0.324185	0.08829	0.020567	0.023891
1	1	1	0	22.47971	0.363326	0.098356	0.022786	0.016795
1	1	1	0	22.59424	0.368849	0.090437	0.022613	0.019607
1	1	1	0	22.56403	0.360017	0.076575	0.021666	0.007927
1	1	1	0	22.65923	0.39629	0.073031	0.019006	0.101336
1	1	1	0	22.83492	0.448858	0.065682	0.015172	0.152406
1	1	1	0	22.85261	0.438047	0.072675	0.01613	-0.0336
1	1	1	1	20.85692	0.214941	0.135583	0.029802	-0.05419
1	1	1	1	21.02954	0.237477	0.113799	0.01781	-0.00605
1	1	1	1	22.15582	0.382744	0.081164	0.019146	0.178989
0	0	1	1	22.50198	0.500716	0.090209	0.018016	5.076488
0	0	1	1	22.75766	0.475353	0.065012	0.016034	0.151194
0	0	1	1	23.33099	0.582392	0.050847	0.015399	0.090298
0	0	1	1	23.70441	0.471831	0.046389	0.017213	0.268687
0	0	1	1	23.91077	0.558185	0.03709	0.013872	0.148757
0	0	3	0.666667	24.09366	0.542914	0.042855	0.012123	0.28425
1	1	3	0.666667	24.00235	0.519833	0.056766	0.014775	0.160802
1	1	3	0.666667	24.00554	0.502796	0.062455	0.013837	-0.04561
0	0	1	1	21.51932	0.682764	0.052045	-0.00053	-0.23813
1	1	1	1	22.34388	0.52337	0.0651	0.010457	0.350426
1	1	1	1	22.63591	0.597411	0.077584	0.021557	0.181169
1	1	1	1	22.94279	0.540303	0.031048	0.011272	-0.10042
1	1	1	1	23.11383	0.608422	0.023328	0.00766	0.134991
1	1	1	1	23.28328	0.544834	0.025084	0.003517	0.327436
1	1	1	1	23.32086	0.553921	0.013243	-0.00321	0.084632
1	1	1	1	23.32815	0.569605	0.013386	-0.00047	-0.10132
1	1	1	1	23.35829	0.537444	-0.04131	0.000536	0.037827
0	0	1	1	21.0098	0.753519	0.029909	-0.00184	0.043577
0	0	1	1	21.26253	0.762261	0.080232	0.009808	0.52214
0	0	1	1	21.71489	0.537408	0.180768	0.066754	1.412428
0	0	1	1	21.51634	0.540123	-0.08754	0.006936	-0.68721
0	0	1	1	21.16903	0.438473	0.020544	0.00435	-0.27779
0	0	1	1	21.20511	0.419551	0.040325	0.007282	0.070945
0	0	1	1	21.30115	0.42375	0.055492	0.006873	0.362495
1	1	1	1	21.37712	0.46119	0.007412	0.006769	0.056772
1	1	1	1	21.26408	0.465939	-0.06777	-0.01339	-0.13837
0	0	1	0	21.72937	0.056303	0.16342	0.059988	-0.23797
0	0	1	0	21.80936	0.0445	0.119079	0.03975	0.011509
0	0	1	0	22.01616	0.091277	0.146446	0.042025	0.149262
0	0	1	0	22.05024	0.035467	0.161319	0.054219	0.112629
0	0	1	0	22.11492	0.034094	0.160407	0.043052	0.183577
0	0	1	0	22.26023	0.046243	0.191039	0.051525	0.314525
0	0	1	0	22.37563	0.072278	0.175513	0.05919	0.224073

0	0	1	0	22.594	0.11096	0.214139	0.044787	0.358386
0	0	1	0	22.7492	0.092211	0.212842	0.046012	0.150158
1	3	1	1	21.17078	0.688376	0.020507	0.004933	-0.29172
1	3	1	1	21.65812	0.715498	0.04859	0.012662	0.754783
1	3	1	1	21.82575	0.757763	0.042326	0.011007	0.439177
1	3	1	1	21.89495	0.767271	0.044593	0.010346	0.112991
1	3	1	1	22.0896	0.616131	0.032291	0.013907	0.016309
1	3	1	1	22.0824	0.589513	0.041329	0.008513	0.019615
1	3	1	1	22.24754	0.564555	0.037357	0.008516	0.220992
1	3	1	1	22.29564	0.550989	0.034223	0.008717	0.120469
1	3	1	1	22.39502	0.58271	0.034935	0.009843	0.008842
1	2	1	1	22.52148	0.585447	0.03925	0.019707	0.424853
0	0	1	1	22.49282	0.642903	0.024321	0.009482	-0.09855
1	1	2	1	21.02938	0.373705	0.168404	0.026384	-0.0073
1	1	2	1	21.38356	0.225052	0.115588	0.03119	0.228305
1	1	2	1	21.58981	0.304144	0.086455	0.018811	0.268779
1	1	2	1	21.81925	0.370054	0.077985	0.002736	0.158635
1	1	2	1	22.01356	0.432607	0.08798	0.012046	0.221186
1	1	2	1	22.19947	0.505613	0.098766	0.021232	0.228233
0	0	2	1	23.3962	0.229641	0.059635	0.02063	0.061707
0	0	2	1	23.56296	0.311301	0.05407	0.007428	0.529252
0	0	2	1	23.9046	0.480685	0.039608	0.011865	1.352884
0	0	2	1	24.12211	0.549848	0.056077	0.007705	0.04854
0	0	2	1	24.14045	0.46239	0.077629	0.011853	-0.00024
1	3	2	0.5	20.28319	0.057358	0.008089	-0.01114	0.151048
1	1	2	0.5	23.90468	0.68091	0.07601	-0.00833	0.335
1	1	1	1	22.23347	0.616642	0.001781	0.005087	-0.56725
1	1	2	0	21.20519	0.54151	0.02637	0.011764	-0.37048
1	1	2	0	21.10744	0.569115	-0.05783	0.006701	-0.01202
0	0	1	1	20.8547	0.199318	0.03707	0.012651	-0.15406
0	0	1	1	20.78246	0.521535	0.054968	0.01375	0.083562
1	1	1	0	21.41011	0.558009	-0.03326	-0.00123	-0.23117
1	1	1	0	21.57398	0.599234	0.036249	0.006317	1.452106
1	1	1	0	21.68344	0.591098	0.043182	0.014395	0.279757
1	1	1	0	21.79576	0.630337	0.021522	0.006491	-0.15569
1	1	1	0	21.91781	0.664382	0.028392	0.007099	0.305672
1	3	1	0	22.1298	0.62182	0.01181	0.006918	-0.11889
1	1	2	1	20.57031	0.323816	0.08386	0.004033	0.112256
1	1	2	1	20.92024	0.211874	0.057153	0.013749	-0.09167
1	1	2	1	21.06491	0.276339	0.049045	0.012789	0.119278
1	1	2	1	21.25671	0.382068	0.060597	0.013205	0.273391
1	1	2	1	21.58146	0.317597	0.069292	0.01384	0.457964
1	1	2	1	22.54673	0.364823	0.018717	0.007895	0.210168
1	1	1	0	20.33849	0.22449	0.045091	0.005477	0.000398
1	1	1	0	20.44006	0.253751	0.063645	0.010616	0.251032
1	1	1	0	20.50001	0.233914	0.085443	0.014104	0.328358
1	1	1	0	20.59615	0.239667	0.079073	0.016047	0.007357
1	1	1	0	20.76255	0.320544	0.05656	0.012916	0.114207
1	1	1	0	21.40879	0.208234	0.05864	0.047077	0.122633
1	1	1	0	21.4264	0.190978	0.037208	0.005835	0.027261
1	1	1	0	21.74235	0.188393	0.038125	0.009407	0.425344
1	1	1	0	21.80435	0.202997	0.059317	0.015083	0.244682
1	1	1	0	21.84641	0.189776	0.050219	0.016396	0.131034
1	1	1	0	21.78155	0.190681	-0.03461	0.011397	-0.05228
0	0	1	1	23.6511	0.250479	0.124603	0.02289	-0.16599
0	0	2	1	21.45759	0.312808	0.11798	0.046016	0.202417
0	0	2	1	21.61358	0.3166	0.137344	0.052896	0.105946
0	0	2	1	21.8842	0.36062	0.157634	0.0581	0.328894

0	0	2	1	22.43506	0.216693	0.133065	0.073061	0.190512
0	0	2	1	22.64718	0.32247	0.064977	0.039457	-0.20233
0	0	2	1	22.6571	0.293625	0.04083	0.018936	-0.1379
0	0	2	1	22.72445	0.314665	0.024849	0.012905	0.039913
0	0	1	1	22.79261	0.336339	0.041084	0.011733	0.061587
0	0	1	1	22.87305	0.355939	0.040998	0.010955	0.168696
0	0	1	1	22.87413	0.324573	0.051543	0.012662	0.140082
1	1	1	1	22.94588	0.343818	0.042529	0.012417	0.030186
1	3	1	1	21.71306	0.089715	0.098029	0.021756	-0.11861
1	1	2	1	20.92125	0.565628	0.058309	-0.00094	-0.23309
1	1	2	1	20.90062	0.542443	0.041148	0.002848	0.283281
1	3	2	1	21.09272	0.535959	0.141782	0.015756	0.077426
1	3	2	1	21.16042	0.575082	0.030034	0.00082	-0.00869
1	3	2	1	21.11212	0.541461	0.041437	0.000969	0.016006
1	3	2	1	20.95782	0.582913	-0.01828	0.001696	-0.19074
1	3	2	1	20.49636	0.380993	0.06068	-0.00316	-0.17302
1	3	2	1	20.80838	0.222927	0.02127	0.00018	-0.08876
1	3	2	1	22.31752	0.321558	0.075124	0.00433	0.715894
1	3	2	1	22.54922	0.411027	0.063931	0.013461	-0.40467
1	3	2	1	22.92642	0.543869	0.06045	0.013124	0.356929
0	0	1	1	21.17148	0.391981	0.111538	0.078786	0.388989
0	0	1	1	21.39244	0.333623	0.191134	0.04843	0.619367
0	0	1	1	21.55078	0.259739	0.171971	0.05874	0.229457
0	0	1	1	21.80231	0.28729	0.136757	0.012817	0.015454
1	3	1	1	21.9746	0.258106	0.154928	0.038449	0.285759
1	3	1	1	22.09681	0.264858	0.136361	0.043047	-0.0168
1	3	1	1	22.18132	0.258884	0.128111	0.028551	0.034249
1	3	1	1	22.22009	0.18047	0.129484	0.037913	0.046942
1	3	1	1	22.21527	0.154428	0.0294	0.012533	-0.49844
1	3	1	1	22.04728	0.131382	-0.00092	0.002865	-0.05319
1	3	1	1	22.02839	0.147742	-0.00875	-0.0038	0.081611
0	0	1	0	21.29842	0.46381	0.086185	0.013694	0.058517
0	0	1	0	21.726	0.493495	0.064733	0.025003	0.686549
0	0	1	0	21.74885	0.490575	0.055499	0.00761	0.225896
0	0	1	0	21.60927	0.505057	-0.07478	0.01047	-0.15645
1	1	1	0	21.68605	0.523929	0.050063	0.010408	0.112314
1	3	1	0	21.66682	0.487681	0.052457	0.004831	-0.00288
1	3	1	0	21.78019	0.537999	0.027553	0.004706	0.107054
1	3	1	0	21.82333	0.529116	0.02273	0.003122	0.151255
1	3	1	0	22.14994	0.64045	0.046262	0.009663	0.576083
1	1	1	0	22.29309	0.643403	-0.03373	0.006256	-0.00171
1	3	1	0	22.1673	0.544514	0.100821	0.004511	0.099864
0	0	1	1	20.00888	0.131565	0.089055	0.013351	0.072969
0	0	2	1	21.61566	0.422003	0.14248	0.023795	-0.16513
0	0	2	1	22.22054	0.392558	0.106951	0.042237	0.525724
0	0	2	1	22.29959	0.374964	0.114748	0.030678	0.344941
1	1	2	1	22.31961	0.406143	0.079569	0.02752	-0.08611
1	1	2	1	22.6086	0.521138	0.0781	0.021912	0.378383
1	1	2	1	22.67213	0.494156	0.10387	0.021419	0.104232
1	1	2	1	22.80763	0.416924	0.093344	0.02002	0.057849
1	1	2	1	22.85134	0.361035	0.110312	0.021169	0.098751
1	1	2	1	23.23676	0.358174	0.129691	0.02592	0.415416
1	1	2	1	23.35747	0.375541	0.106738	0.029458	0.130959
1	1	2	1	23.41723	0.366782	0.111918	0.026013	0.041667
1	1	2	1	22.5332	0.719272	0.073478	0.017118	0.243924
1	1	2	1	22.81256	0.748213	0.064708	0.013995	0.008947
1	1	2	1	22.9518	0.76295	0.032245	0.017185	0.011105
1	1	2	1	23.15211	0.791309	0.02157	0.013362	0.116605

1	1	2	1	23.24898	0.801958	0.028322	0.008484	0.151359
1	1	2	1	23.35572	0.809151	0.027432	0.007339	0.01474
1	1	2	1	23.42444	0.816406	0.02613	0.007977	0.067017
1	1	2	1	23.31451	0.790311	0.040243	0.008703	0.034061
1	1	2	1	23.28946	0.768074	0.040139	0.008771	-0.07622
1	1	2	1	23.39941	0.78885	0.029505	0.009941	0.21668
1	1	2	1	23.47894	0.788431	0.041912	0.009238	0.207832
1	1	2	1	21.26839	0.200641	0.079711	-0.0124	0.177423
1	1	2	1	21.38832	0.145515	0.194322	0.053668	0.361214
1	1	2	1	21.48534	0.235281	0.035981	0.042708	-0.13129
1	3	2	1	21.60933	0.31687	0.022591	-0.01103	0.221471
1	3	2	1	21.74477	0.330334	0.120036	0.012427	0.373487
1	3	2	1	22.21227	0.291169	0.112343	0.041658	-0.0046
1	3	2	1	22.40689	0.377156	0.066207	0.025422	0.078688
1	3	2	1	22.40728	0.440223	-0.04323	0.007557	0.116539
1	3	2	1	22.43924	0.388707	0.092501	0.023571	0.458984
1	3	2	1	22.50472	0.359895	0.093251	0.027021	0.068928
1	3	2	1	23.55498	0.542589	0.130263	0.024689	2.107786
1	1	2	1	22.51663	0.381917	0.148342	0.032818	0.26681
1	1	2	1	22.79316	0.205575	0.142926	0.028648	0.16915
1	1	2	1	23.13531	0.228172	0.112627	0.023663	0.088641
1	1	2	1	23.24323	0.283393	0.083426	0.017414	0.150518
1	1	2	1	23.37327	0.362665	0.056738	0.040546	0.125585
1	1	2	1	23.49621	0.420781	0.057797	0.010243	0.161926
1	1	2	1	23.54673	0.429821	0.042419	0.010349	0.044675
0	0	1	1	21.15571	0.623722	0.080841	0.016091	0.387105
0	0	1	1	21.43026	0.471406	0.069124	0.022055	0.155205
0	0	1	1	21.41772	0.430569	0.065098	0.009134	0.167328
0	0	1	1	21.92906	0.512392	0.030793	0.014714	-0.02689
0	0	1	1	21.81014	0.437117	0.037634	0.009287	0.215475
0	0	1	1	21.80051	0.689594	0.028184	0.008361	-0.15015
0	0	1	1	21.7189	0.718668	-0.0205	0.000501	-0.45324
0	0	1	1	21.72341	0.737138	0.040894	0.008692	0.286039
0	0	1	1	20.20177	0.399709	0.015679	-0.00919	-0.1215
0	0	1	1	20.38729	0.37109	0.044843	0.013169	0.319506
0	0	1	1	20.71749	0.479044	0.029914	0.011558	0.136289
0	0	1	1	20.72778	0.597324	-0.08074	0.006967	-0.0942
0	0	1	1	20.72905	0.590237	0.026819	0.008333	0.006928
0	0	1	1	21.04478	0.354259	0.206351	0.007588	0.216524
1	3	1	1	21.79386	0.604505	0.136331	0.021846	0.956506
1	3	1	1	21.81585	0.6618	0.128226	0.015013	0.364568
1	3	1	1	22.04241	0.757566	0.081238	0.011398	-0.08136
1	1	1	1	21.8013	0.8241	-0.07349	0.01608	0.161704
1	1	1	1	20.78317	0.827822	-0.01332	0.008581	-0.20481
1	1	1	1	20.83328	0.832123	0.048226	0.003985	-0.18436
0	0	1	1	22.62735	0.54686	0.104571	0.013413	2.100482
0	0	1	1	23.04709	0.612323	0.121335	0.04816	0.732995
0	0	1	1	23.56216	0.517157	0.060968	0.021136	0.016849
0	0	1	1	23.74793	0.58469	0.036603	0.01086	0.059713
0	0	1	1	23.94894	0.64022	0.013617	0.013221	-0.03279
1	3	1	1	23.04016	0.584341	0.096484	0.106611	0.196463
1	3	1	1	22.82166	0.485543	0.030061	-0.00033	-0.19017
1	3	1	1	22.8494	0.505938	0.021376	0.005035	-0.11205
1	3	1	1	22.68862	0.410101	0.029148	0.004577	-0.04439
1	3	1	1	22.60274	0.381069	-0.02931	0.003967	-0.28625
1	3	1	1	22.78235	0.384815	0.07296	0.001394	0.031009
1	3	1	1	22.9908	0.363175	0.194555	0.033338	0.636592
1	3	2	1	23.16674	0.347229	0.104222	0.024217	-0.08414

1	1	1	1	20.00964	0.187457	0.046083	0.01299	-0.06305
1	1	1	1	20.08814	0.215714	0.042123	0.009448	0.076355
0	0	1	1	20.16358	0.257459	0.05935	0.009327	0.3268
0	0	1	1	20.1475	0.252342	0.032471	0.010748	-0.11019
0	0	1	1	20.15804	0.286794	0.042617	0.014429	-0.07191
0	0	1	1	20.2315	0.313759	0.030362	0.011628	0.127037
0	0	1	1	21.08245	0.299025	0.044909	0.007583	0.814207
0	0	1	1	21.23937	0.390069	0.024447	0.003055	0.014698
0	0	1	1	21.51703	0.525038	0.037918	0.007496	0.260845
0	0	3	0.333333	22.95099	0.603649	0.089038	0.011446	0.027811
0	0	3	0.333333	23.17389	0.635957	0.104437	0.020221	0.348332
0	0	3	0.333333	23.44376	0.701886	0.080234	0.018363	0.090244
0	0	3	0.333333	23.44276	0.706315	0.061946	0.013415	0.187883
0	0	3	0.333333	23.48893	0.652472	0.061878	0.015552	0.046736
0	0	3	0.333333	23.51402	0.642497	0.075017	0.019997	-0.04013
0	0	3	0.333333	23.70479	0.625026	0.082715	0.017831	0.035116
0	0	3	0.333333	23.72866	0.587013	0.099943	0.015967	0.335365
0	0	3	0.333333	23.98352	0.584588	0.134435	0.036506	0.307066
0	0	3	0.333333	24.10844	0.572256	0.116496	0.039912	0.152115
0	0	3	0.333333	24.19818	0.544827	0.09805	0.020463	0.045677
1	1	1	1	20.83568	0.274702	0.141051	0.013612	0.087706
0	0	1	1	20.99831	0.288973	0.151867	0.025293	0.268758
0	0	1	1	21.09227	0.271192	0.149147	0.032294	0.350169
0	0	1	1	21.17211	0.299452	0.083105	0.017302	0.00911
0	0	1	1	21.16085	0.233107	0.097074	0.012853	0.074129
0	0	1	1	21.16218	0.164208	0.087135	0.015508	-0.02839
0	0	1	1	21.20401	0.167122	0.050687	0.007691	0.010523
0	0	1	1	21.22913	0.15789	0.047694	0.006895	-0.20642
0	0	1	0	22.63941	0.62615	0.042428	0.010003	0.01124
0	0	1	0	22.69506	0.622121	0.061346	0.014306	0.316373
0	0	1	0	22.72413	0.614006	0.054651	0.021038	0.085097
1	1	1	0	22.65857	0.593824	0.03709	0.013974	-0.03062
1	1	1	0	22.66077	0.598033	0.047367	0.018983	-0.00611
1	1	1	0	22.77377	0.643289	0.025249	0.016973	0.01816
1	1	1	0	22.75959	0.609528	0.073863	0.020194	-0.07508
1	1	1	0	22.68684	0.552236	0.084957	0.022202	0.0457
1	1	1	0	22.72601	0.550772	0.095687	0.030026	0.102103
1	1	1	0	22.8579	0.595965	0.079122	0.024199	0.069035
1	1	1	0	23.03308	0.623708	0.062733	0.029815	-0.03252
1	1	3	0.333333	21.24541	0.509862	0.045323	0.001824	-0.01534
1	1	3	0.333333	21.33293	0.376709	0.03213	0.007051	0.039356
1	1	3	0.333333	21.34856	0.331693	0.03925	0.009073	0.041782
1	1	3	0.333333	21.61619	0.31334	0.065496	0.015664	0.159013
1	1	3	0.333333	21.77823	0.420893	0.027351	0.014698	0.074396
1	1	3	0.333333	21.8293	0.432317	0.062277	0.011288	0.154956
1	1	1	1	21.15686	0.393451	0.068017	0.013996	-0.06223
0	0	1	1	21.16622	0.321888	0.130924	0.032492	0.399014
1	1	1	1	21.66723	0.304124	0.141928	0.033909	1.07531
1	1	1	1	21.83178	0.374439	0.133444	0.008776	0.039988
1	1	1	1	22.16135	0.508525	0.120343	0.038932	0.112837
1	1	1	1	22.34505	0.524391	0.13166	0.03417	0.215886
1	1	1	1	22.90325	0.467554	0.136444	0.033985	0.532232
1	1	1	1	22.95205	0.380349	0.149342	0.033162	0.118455
1	1	1	1	23.03075	0.349837	0.127858	0.031779	0.072899
1	1	1	1	23.31799	0.489346	0.102223	0.032382	0.081343
1	1	1	1	23.36153	0.52385	0.08582	0.02193	-0.01975
1	1	1	1	21.21299	0.31894	0.046409	0.006378	-0.15456
1	1	1	1	21.35765	0.371113	0.05871	0.006303	0.317575

1	1	1	1	21.47719	0.396283	0.068728	0.006977	0.206096
1	1	1	1	21.57413	0.430153	0.068841	0.007505	-0.10113
0	0	1	1	21.64569	0.446131	0.045671	0.007542	-0.08999
0	0	1	1	21.69644	0.459295	0.039667	0.004098	-0.01788
0	0	1	1	22.04974	0.249994	0.031397	0.006322	0.115965
0	0	1	1	22.06954	0.248809	0.025689	0.002692	0.04759
0	0	1	1	22.12139	0.259009	0.039944	0.001658	0.102866
1	1	2	1	20.57909	0.354988	0.127737	0.031969	0.492524
0	0	2	1	21.03203	0.285524	0.131018	0.033873	0.233466
0	0	2	1	21.19743	0.306779	0.139965	0.015386	0.441414
0	0	2	1	21.36177	0.368753	0.084882	0.019118	0.040692
0	0	2	1	21.52685	0.439	0.058336	0.013883	0.007848
0	0	2	1	21.98726	0.471391	0.059995	0.019892	0.387214
0	0	2	1	22.30841	0.589945	0.057326	0.014446	0.52501
0	0	2	1	22.40511	0.62116	0.029047	0.003501	0.091058
0	0	2	1	22.23661	0.596614	-0.00815	0.009124	-0.15568
0	0	2	1	22.27717	0.718684	-0.08992	0.009485	-0.52245
1	3	1	0	20.57272	0.325691	0.151845	0.053885	0.274599
1	3	1	0	21.11388	0.328846	0.087954	0.043702	0.155853
1	3	1	0	21.34542	0.403164	0.074994	0.038796	0.132953
1	3	1	0	21.46645	0.397061	0.04464	0.024312	-0.13818
1	3	1	0	21.54599	0.437346	0.06288	0.036802	-0.48467
1	3	1	0	21.60324	0.456307	0.037574	0.019539	0.202264
1	3	1	0	21.76969	0.526065	0.02612	0.012957	0.080862
1	3	1	0	22.24325	0.376066	0.024294	0.016044	1.4742
1	3	1	0	22.26662	0.357618	0.04949	0.013379	0.231984
1	3	1	0	22.23123	0.300019	0.038043	0.017786	-0.35152
1	3	1	0	22.19886	0.24169	0.031771	0.010532	-0.46708
0	0	1	1	21.71941	0.513118	0.053804	0.00801	0.157109
0	0	1	1	22.19135	0.637859	0.065877	0.022739	0.95508
0	0	1	1	22.29435	0.631513	0.071076	0.023868	0.088626
0	0	1	1	22.3926	0.681005	0.026823	0.010434	-0.37296
0	0	1	1	22.45136	0.696368	0.03052	0.008813	0.090589
0	0	1	1	22.58421	0.620472	0.027113	0.010178	-0.12934
1	1	1	1	22.56685	0.61638	0.02244	0.006412	-0.2084
1	1	1	1	23.04295	0.690726	0.022757	0.007365	0.367674
1	1	1	1	23.23187	0.6121	0.039011	0.018352	0.98142
1	1	1	1	23.38264	0.654458	0.051487	0.017135	0.458496
1	1	1	1	20.71953	0.436109	0.049265	0.009722	-0.15957
1	1	1	1	20.85457	0.440474	0.101948	0.025548	0.20285
1	1	1	1	21.08452	0.486486	0.101099	0.011296	0.208311
1	1	1	1	21.37456	0.615204	0.028094	0.035731	-0.11788
1	3	1	1	21.46973	0.70003	-0.02198	0.005147	0.013372
1	3	2	0.5	22.15057	0.237252	0.029312	0.006554	0.136509
1	3	2	0.5	22.29827	0.260418	0.125884	0.045239	0.868265
1	3	2	0.5	22.6556	0.309207	0.041039	0.03298	-0.01243
1	3	2	0.5	22.73375	0.311362	0.072539	0.021239	-0.05133
1	3	2	0.5	22.67422	0.233793	0.091792	0.020257	0.177436
1	3	2	0.5	22.63239	0.151579	0.129622	0.035075	0.081955
0	0	3	1	20.99113	0.677748	0.029031	0.007098	0.23532
0	0	3	1	21.24062	0.646278	-0.03407	0.005462	-0.15634
0	0	3	1	21.26107	0.726185	-0.04182	0.032359	-0.03289
0	0	3	1	21.42322	0.660846	0.05002	0.008825	1.682897
0	0	3	1	22.21896	0.538907	0.025219	0.013282	0.003627
0	0	3	1	22.30671	0.402882	0.064998	0.009271	-0.29761
0	0	3	1	22.68615	0.355812	0.05275	0.011191	-0.32595
0	0	3	1	22.82872	0.349472	0.047025	0.007405	9.973616
1	1	2	1	20.19895	0.076737	0.17969	0.02832	0.134786

1	1	2	1	20.41435	0.133185	0.163088	0.029924	0.558861
1	1	2	1	20.56332	0.171082	0.156429	0.029303	0.169473
0	0	2	1	20.79782	0.249488	0.13576	0.027549	0.199918
1	1	2	1	20.92967	0.214915	0.19138	0.027531	0.356099
1	3	2	1	21.18286	0.236436	0.196491	0.033978	0.229794
1	3	2	1	21.37597	0.183717	0.215008	0.034189	0.174039
0	0	2	1	21.43328	0.181932	0.079048	0.031274	-0.25667
0	0	1	0	20.75387	0.474956	0.059686	0.012282	-0.2519
0	0	1	0	20.89943	0.520247	0.057163	0.016933	0.259261
0	0	1	0	21.06551	0.588885	0.039533	0.005708	0.021222
0	0	1	0	21.07692	0.601701	0.056948	0.005683	0.118142
0	0	1	0	21.08903	0.585843	0.036469	0.009914	-0.04778
0	0	1	0	21.11118	0.598889	0.02769	0.007151	0.10189
0	0	1	0	20.9958	0.607184	-0.03993	0.003972	-0.14901
0	0	2	0.5	21.05476	0.618203	0.02855	0.007841	0.017142
0	0	2	0.5	21.0976	0.606626	0.063001	0.005904	0.262954
0	0	2	0.5	21.2281	0.656327	0.011839	0.002692	-0.13876
0	0	2	0.5	21.21898	0.637188	0.042175	0.000507	0.308081
0	0	2	0.5	20.56371	0.483492	0.077194	0.025701	-0.11345
0	0	2	0.5	20.91095	0.535255	0.074346	0.01982	0.298551
0	0	2	0.5	21.1869	0.352433	0.067125	0.01599	0.247206
0	0	2	0.5	21.32212	0.395674	0.065531	0.012323	0.284832
0	0	2	0.5	21.46077	0.441549	0.059541	0.012397	0.091937
0	0	2	0.5	21.64186	0.499516	0.060158	0.013487	0.14662
0	0	2	0.5	21.92889	0.387091	0.067581	0.0134	0.303403
0	0	2	0.5	22.1482	0.331483	0.057105	0.017089	0.175495
0	0	2	0.5	22.22462	0.404806	0.037319	0.012839	0.159992
0	0	2	0.5	22.24438	0.383559	0.063765	0.013943	0.21233
0	0	2	0.5	22.29526	0.384065	0.088116	0.0162	0.166533
0	0	1	1	20.30044	0.342752	0.120626	0.018048	0.147562
0	0	1	1	21.66689	0.380453	0.078066	0.014616	1.506627
0	0	2	0.5	21.74901	0.351384	0.102735	0.028809	0.443951
1	3	1	1	21.8369	0.377605	0.052855	0.011923	-0.54885
1	3	1	1	22.05247	0.484937	0.038269	0.013274	0.134209
1	3	1	1	22.12258	0.5076	0.045187	0.009885	0.63423
1	3	1	1	22.33233	0.596811	0.036755	0.010013	-0.18364
1	3	1	1	22.61545	0.435522	0.053562	0.010402	0.294122
1	3	1	1	22.90701	0.534625	0.071459	0.012177	0.619531
1	3	1	1	23.01774	0.565447	0.0461	0.024409	-0.00171
1	3	1	1	23.08964	0.582794	0.038302	0.004771	-0.35146
0	0	1	0	20.80731	0.42129	0.059235	0.007219	-0.05366
0	0	1	0	21.07569	0.514831	0.076943	0.017362	0.641188
0	0	1	0	21.23298	0.610235	0.035919	0.011182	0.445297
0	0	1	0	21.18334	0.588809	0.03502	0.010035	-0.17498
0	0	1	0	21.20302	0.533166	0.040747	0.00798	0.024974
1	3	1	1	21.17331	0.517071	0.033274	0.006329	0.045595
0	0	1	1	22.48818	0.311177	0.118961	0.098333	0.027236
0	0	1	1	22.60075	0.282362	0.12611	0.026915	0.098412
0	0	1	1	22.68953	0.300827	0.077165	0.024637	-0.13512
0	0	1	1	22.70506	0.298933	0.042088	0.012472	-0.1919
0	0	1	1	22.74634	0.341421	0.013276	0.003798	-0.18866
0	0	1	1	22.73756	0.336168	0.018468	0.003774	0.070937
0	0	1	1	22.74549	0.339487	0.017865	0.006058	-0.03543
1	3	1	1	22.61764	0.296187	-0.02917	0.004529	0.028387
1	3	1	1	22.87529	0.529779	0.034575	0.006087	0.175708
1	1	1	1	20.87211	0.53441	0.07402	0.012899	-0.0885
1	1	1	1	21.22818	0.58747	0.111153	0.024011	0.644959
1	1	1	1	21.57285	0.458387	0.067443	0.023394	0.232758

1	1	1	1	21.63129	0.456921	0.033575	0.01143	-0.07274
1	1	1	1	21.87159	0.535541	0.0525	0.011611	0.136718
1	1	1	1	21.98024	0.534978	0.06868	0.017769	0.261187
1	1	1	1	22.09055	0.436793	0.072067	0.021114	0.127328
1	1	1	1	22.29066	0.466098	0.074193	0.018742	0.145714
1	1	1	1	22.6221	0.447673	0.068059	0.02188	0.386286
1	1	1	1	22.7849	0.478686	0.065686	0.021941	0.160985
1	1	1	1	22.85436	0.515216	0.055993	0.020554	0.099916
1	1	1	1	21.3251	0.495896	0.077173	0.011874	0.266898
1	1	1	1	21.6801	0.550508	0.063176	0.015287	0.45311
1	1	1	1	21.86013	0.618567	0.051769	0.029416	0.048673
1	1	1	1	21.91698	0.633991	0.044321	0.004772	-0.00676
1	1	1	1	21.92357	0.643923	0.039481	0.006248	-0.09961
1	1	1	1	21.95248	0.640321	0.046194	0.007841	0.072888
1	1	1	1	21.75781	0.338064	0.03844	0.008206	-0.07502
0	0	1	1	22.17703	0.330903	0.042748	0.010216	0.18934
0	0	1	1	22.58601	0.518877	0.023233	0.011081	0.475534
0	0	1	1	22.42593	0.486955	-0.02832	0.003145	-0.01045
0	0	2	1	22.29179	0.432923	0.036818	0.006909	0.123349
1	1	1	1	22.60101	0.726385	0.042513	0.002983	0.382967
0	0	1	1	22.66346	0.721646	0.052506	0.005815	0.645584
0	0	1	1	22.62642	0.680357	0.07096	0.00891	-0.11193
0	0	1	1	22.68428	0.675179	0.05215	0.000133	0.024997
1	1	1	1	22.80451	0.692151	0.045418	0.021006	-0.15519
1	1	1	1	22.87056	0.609817	0.027182	0.004494	0.01553
1	1	1	1	22.89061	0.634225	-0.00206	0.001064	0.021412
1	1	1	1	22.82535	0.611275	0.07287	-0.00048	1.438827
1	1	1	1	23.17056	0.693333	0.076888	0.067877	-0.08741
1	1	2	1	23.29405	0.709468	0.046076	0.000088	0.212894
1	1	1	1	19.88815	0.323652	0.032233	0.003112	-0.01378
1	1	1	1	21.12355	0.23922	0.031352	0.015133	0.372313
1	1	1	1	21.10025	0.243966	0.055657	0.010448	0.43548
1	1	1	1	21.15156	0.181328	0.090564	0.007997	0.069642
1	1	1	1	21.50233	0.146251	0.041501	0.011819	-0.06815
1	1	1	1	21.50778	0.215098	0.057945	0.009902	-0.06957
1	1	1	1	21.45142	0.176711	0.069963	0.056984	-0.03388
0	0	1	1	21.4434	0.173494	0.053367	0.009033	0.081731
0	0	1	1	21.42906	0.16658	0.054968	0.012036	0.114207
0	0	1	1	21.3877	0.141832	0.051768	0.012789	0.005844
0	0	1	1	21.38758	0.11189	0.046458	0.013729	-0.14902
1	3	1	0	20.251	0.284847	0.101064	0.014082	0.208472
1	3	1	0	20.42742	0.34868	0.105218	0.023785	0.352952
1	3	1	0	20.54205	0.393474	0.081101	0.023765	0.127847
1	3	1	0	20.59738	0.403682	0.076001	0.017295	0.071908
1	3	1	0	20.62108	0.380115	0.084756	0.01956	0.063001
1	3	1	0	20.61069	0.337644	0.094504	0.017965	0.045176
1	3	1	0	20.6226	0.294559	0.097403	0.017584	-0.05362
1	3	1	0	21.03941	0.215407	0.093084	0.023581	0.104986
1	3	1	0	21.091	0.196405	0.095555	0.02247	0.174863
1	3	1	0	21.17597	0.18881	0.109589	0.022626	0.105522
1	3	1	0	21.25111	0.174232	0.135691	0.023338	0.093591
1	3	1	0	21.41184	0.198297	0.114245	0.016493	0.164092
1	3	1	0	21.67054	0.276953	0.111378	0.013184	0.151947
1	3	1	0	22.13088	0.465023	0.10879	0.018911	0.61228
1	3	1	0	22.28551	0.447504	0.101244	0.019822	0.1113
1	1	1	0	22.24637	0.427594	0.047821	0.014269	-0.10458
1	1	1	0	22.19321	0.373062	0.05101	0.017071	0.127198
1	1	1	0	22.2348	0.383003	0.044452	0.022011	-0.01453

1	1	1	0	22.14918	0.423662	-0.08324	0.00522	-0.10408
0	0	1	0	22.17162	0.406285	0.015137	0.008924	0.29525
0	0	1	0	22.15739	0.334682	0.035877	0.014042	0.195451
0	0	1	0	22.20881	0.345359	0.066446	0.027415	0.05242
0	0	1	1	21.46317	0.4915	0.048829	0.004304	0.04099
0	0	1	1	22.01366	0.413268	0.055367	0.018262	0.395161
0	0	1	1	21.94416	0.356803	0.024515	0.007517	-0.06083
0	0	1	1	21.94185	0.347009	0.022275	0.003667	-0.01974
0	0	1	1	22.02786	0.387952	0.031858	0.005357	0.111506
0	0	1	1	22.09826	0.40216	0.042422	0.008292	0.182968
0	0	1	1	22.5969	0.425611	0.030586	0.01197	0.110594
0	0	1	1	23.2203	0.484765	0.030977	0.007123	0.419805
0	0	1	1	23.50563	0.597569	0.013057	-0.00047	0.144501
1	3	1	1	21.32318	0.64283	0.059113	0.023124	0.653528
1	3	1	1	21.76149	0.568581	0.052699	0.011685	0.665856
1	3	1	1	21.88568	0.589318	0.083695	0.013885	0.439303
1	3	1	1	22.21994	0.696112	0.045672	0.000974	0.274707
1	3	1	1	22.51231	0.749775	0.023555	0.005453	0.143524
1	3	1	1	22.71278	0.668851	0.034142	-0.00586	0.057825
1	2	1	1	23.00192	0.636224	0.057937	-0.00325	-0.00408
1	2	1	1	23.22952	0.492688	0.104029	0.030727	0.152524
1	2	1	1	23.53362	0.596666	0.047088	0.014336	0.089575
1	2	1	1	23.78318	0.680223	0.024957	0.007636	0.072669
1	2	1	1	24.15183	0.67649	0.06749	-0.01327	0.10875
1	1	1	1	20.40501	0.208484	0.111522	0.003592	-0.04697
1	1	1	1	20.59489	0.235321	0.174889	0.033873	0.689137
1	3	1	1	20.70918	0.228147	0.164831	0.035286	0.232922
1	3	1	1	20.72238	0.225823	0.127502	0.020495	-0.18636
1	3	1	1	20.86001	0.243182	0.152143	0.024093	0.213438
1	3	1	1	20.95757	0.235002	0.167754	0.029957	0.165054
1	3	1	1	21.54701	0.192609	0.079037	0.025509	-0.10171
1	3	1	1	21.79186	0.272544	0.083153	0.010373	0.401331
1	3	1	1	21.85835	0.426182	0.084571	0.010788	0.287826
1	3	1	1	21.90277	0.426741	0.074971	0.013176	0.079241
1	3	1	1	21.97564	0.412823	0.086462	0.010592	0.043509
0	0	1	1	20.45519	0.300752	0.189306	0.051127	0.037789
0	0	1	1	20.85903	0.465103	0.124923	0.049048	0.501904
0	0	1	1	21.15305	0.399305	0.089811	0.043166	0.225236
0	0	1	1	21.28955	0.433498	0.075004	0.023712	-0.0262
0	0	1	1	21.32977	0.458104	0.049073	0.021258	0.157848
0	0	2	1	21.30277	0.419675	0.087926	0.017755	0.111802
0	0	2	1	21.30687	0.390035	0.087988	0.022819	-0.14219
0	0	2	1	21.63895	0.36394	0.070982	0.025675	0.003481
0	0	2	1	21.79887	0.457836	0.02917	0.012434	0.183993
0	0	2	1	22.01412	0.585294	0.019574	0.003115	0.136282
0	0	2	1	22.06302	0.587764	0.042795	0.010886	-0.03419
1	1	1	1	20.53008	0.285565	0.104161	0.003914	0.301372
1	1	1	1	21.03096	0.224192	0.065393	0.006639	0.122914
1	1	1	1	21.04829	0.155095	0.060851	0.003841	0.04863
0	0	1	1	21.05783	0.1149	0.053171	0.00704	0.018437
1	1	1	1	21.09076	0.154084	0.022735	0.003549	0.148032
1	3	1	1	21.18597	0.204816	-0.07762	0.002251	-0.27629
1	1	2	0	21.80131	0.53815	0.104649	0.003191	0.431791
1	1	2	0	22.129	0.573416	0.052478	0.031306	0.62586
1	1	2	0	21.80449	0.733578	0.057444	0.003439	-0.07103
1	1	2	0	21.77273	0.707686	0.037836	0.01408	-0.07739
1	1	2	0	21.59533	0.716061	-0.02478	-0.00875	-0.10565
1	1	2	0	22.02503	0.678906	0.054205	0.002926	0.299408

1	1	2	0	22.13973	0.675708	0.085211	0.011293	0.280153
1	1	2	0	22.42862	0.692531	0.04148	0.01594	0.100634
0	0	2	0	22.5981	0.689727	0.009522	0.007577	-0.12221
0	0	2	0	22.57131	0.77283	-0.0689	-0.01058	-0.36696
0	0	1	1	22.26017	0.213806	0.049951	0.009085	0.186623
0	0	1	1	22.17377	0.152097	0.02961	0.01373	0.169861
0	0	2	1	19.66274	0.350169	0.041952	0.010474	-0.03165
0	0	2	1	19.93675	0.481931	0.04314	0.011814	0.241097
0	0	2	1	19.9455	0.495141	0.020834	-0.00434	-0.11423
1	1	2	1	20.3482	0.5689	0.024125	-0.00153	1.697785
1	1	2	1	20.20235	0.5499	-0.03218	0.000572	0.114259
1	1	1	0	19.80136	0.349042	0.030542	0.004672	-0.24467
1	1	1	0	19.75575	0.319374	0.033953	0.005102	0.167424
1	1	1	0	19.78475	0.318591	0.038409	0.0058	0.205171
1	1	1	0	19.84653	0.36103	0.038643	0.00456	0.155501
1	1	1	0	20.30849	0.200399	0.026999	0.007705	-0.05566
1	1	1	0	20.83572	0.282595	0.026985	0.004135	0.374048
1	1	1	0	21.41386	0.547272	0.046296	0.003001	0.664938
0	0	1	0	21.74316	0.424385	0.041185	0.005898	0.421233
1	1	2	1	20.99445	0.298905	0.067402	-0.00109	0.047056
0	0	2	1	21.27802	0.415327	0.078099	0.021251	0.493674
0	0	2	1	21.55923	0.372133	0.048582	0.018273	0.127177
1	1	2	1	21.66149	0.400243	0.062844	0.014603	0.240107
1	1	2	1	21.99275	0.445636	0.073186	0.017678	0.507615
1	1	2	1	22.14831	0.392776	0.090566	0.018109	0.35074
1	1	2	1	22.67894	0.252166	0.061671	0.019689	0.171998
1	1	2	1	22.76153	0.282894	0.06078	0.013371	0.413269
1	1	2	1	22.9604	0.359891	0.068054	0.018319	0.280338
1	1	2	1	23.2444	0.487656	0.039736	0.012319	0.015952
1	1	2	1	23.49866	0.3818	0.029789	0.004076	0.137858
1	1	1	0	22.29108	0.431173	0.038773	0.00459	0.112824
1	1	1	0	22.31021	0.432377	0.023567	0.005067	-0.13387
0	0	2	1	21.57045	0.157235	0.162898	0.034874	-0.04133
0	0	2	1	21.58119	0.12869	0.102803	0.025628	-0.26747
0	0	2	1	21.56098	0.101603	0.078221	0.020319	0.01081
0	0	2	1	21.52104	0.109881	0.01126	0.002373	0.038853
0	0	2	1	21.55593	0.155449	0.018191	0.004915	0.081755
0	0	2	1	21.65996	0.207128	0.057061	0.008828	0.576402
0	0	2	1	21.63809	0.181807	0.030663	0.009226	-0.00066
0	0	2	1	21.65957	0.216754	-0.0642	0.00666	-0.0541
1	1	2	1	21.61017	0.221169	0.10549	0.015204	0.433658
1	1	1	1	20.0313	0.493382	0.022959	0.012227	-0.09138
1	1	1	1	20.16204	0.529298	0.05731	0.019318	0.802013
1	1	1	1	20.35414	0.571161	0.075278	0.023151	0.120766
1	1	1	1	20.32831	0.55555	0.055273	0.025478	-0.17757
1	1	1	1	20.62136	0.317039	0.02035	0.013976	0.024295
1	1	1	1	20.96351	0.357469	0.01563	0.00304	0.27764
1	1	1	1	21.73881	0.187528	0.025019	0.007455	0.276219
1	1	1	1	21.7894	0.190106	0.049896	0.008722	0.320885
1	1	1	1	21.91485	0.248243	0.049785	0.009352	0.256114
0	0	1	1	20.48427	0.522116	0.038213	0.010602	-0.23789
0	0	1	1	20.7335	0.621146	0.03846	0.008132	0.39528
0	0	1	1	20.8286	0.638717	0.046174	0.009667	0.205357
0	0	1	1	20.8637	0.635726	0.050398	0.01163	0.004881
0	0	1	1	20.95394	0.665527	0.034216	0.009771	-0.03981
0	0	1	1	21.22084	0.738956	0.027323	0.007261	0.187464
0	0	1	1	21.48293	0.587795	0.024747	0.006272	-0.07509
0	0	1	1	21.60788	0.635863	0.020953	0.007557	0.153752

0	0	1	1	21.73156	0.678898	0.023506	0.006424	0.25257
1	2	1	1	21.56475	0.612589	0.033209	0.007897	0.077273
1	1	3	0.666667	22.23491	0.681725	0.054136	0.010815	-0.23447
1	1	2	0.5	22.41468	0.712349	0.0476	0.001235	0.495724
1	1	2	0.5	22.6603	0.63453	0.033996	-0.00254	0.312097
1	1	2	0.5	22.58514	0.581455	0.054287	0.014264	-0.12864
1	2	2	0.5	22.7134	0.605727	0.045574	0.011925	0.2652
1	2	2	0.5	22.82941	0.605384	0.075706	0.022	-0.07891
1	2	2	0.5	22.94461	0.551686	0.088941	0.01845	0.126813
0	0	2	0.5	23.26992	0.635792	0.07976	0.016731	0.318307
0	0	2	0.5	23.44981	0.638635	0.064231	0.019194	0.664123
0	0	2	0.5	23.67912	0.563383	0.081203	0.019762	0.36156
0	0	2	0.5	23.92285	0.601858	0.063111	0.017682	0.013698
1	1	1	1	20.99419	0.272621	0.12422	0.030497	-0.08091
1	1	1	1	21.33711	0.4205	0.10456	0.024595	0.701651
0	0	1	1	21.69298	0.219044	0.089711	0.023586	0.423575
0	0	1	1	21.84938	0.361597	0.03383	0.01674	-0.17199
0	0	1	1	21.90666	0.389594	0.036446	0.008811	0.101524
0	0	1	1	21.92857	0.3874	0.059619	0.013475	0.064024
0	0	1	1	22.01469	0.415804	0.059681	0.015693	-0.07224
0	0	1	1	22.15748	0.334701	0.068913	0.021079	0.210099
0	0	1	1	22.20122	0.343895	0.090969	0.019977	0.241388
0	0	2	1	22.29388	0.389873	0.084634	0.021181	0.120335
0	0	2	1	22.44079	0.493817	0.072618	0.020188	0.12443
0	0	2	0.5	21.12718	0.348944	0.037259	0.003267	-0.0284
0	0	2	0.5	21.62436	0.297295	0.047686	0.014395	0.517929
0	0	2	0.5	21.62609	0.3063	0.001687	0.01342	-0.00455
0	0	2	0.5	21.70724	0.356544	0.008372	-0.00337	-0.12184
0	0	2	0.5	20.95826	0.325825	0.027052	0.004616	-0.03638
1	3	2	0.5	20.7566	0.233539	-0.05161	0.004314	-0.13252
1	3	2	0.5	20.78886	0.247123	0.010963	-0.0027	0.017236
1	3	2	0.5	20.89999	0.307516	0.022367	0.011516	0.384027
1	3	2	0.5	20.8909	0.328185	0.007908	0.008074	0.142955
1	2	3	0.666667	21.06416	0.219541	0.127084	0.012544	-0.08281
1	2	3	0.666667	21.34808	0.124266	0.114833	0.014238	0.470574
0	0	3	0.666667	21.38582	0.105619	0.107103	0.002088	0.124171
0	0	3	0.666667	21.52658	0.23967	0.064964	0.014361	-0.15427
0	0	3	0.666667	21.78745	0.411159	0.033592	0.013563	0.038198
0	0	2	0.5	21.82486	0.418255	0.044061	0.009361	0.232601
0	0	2	0.5	21.87245	0.430142	0.043458	0.009366	0.010004
0	0	2	0.5	22.91432	0.382184	0.016481	0.009825	0.258262
1	1	2	0.5	22.9876	0.16523	0.054716	-0.00254	0.788095
0	0	3	0.666667	23.02059	0.174333	0.064656	-8.4E-05	0.227392
0	0	3	0.666667	23.13145	0.19866	0.044719	-0.00934	0.125506
1	2	2	1	21.2006	0.41871	0.057585	0.026487	0.044676
0	0	2	1	21.36829	0.46358	0.070621	0.005981	0.331489
0	0	2	1	21.74374	0.421859	0.053882	0.006445	0.365548
0	0	2	1	21.8625	0.44956	0.047319	0.020101	0.020594
0	0	2	1	22.03398	0.515288	0.042177	0.025984	0.344239
0	0	2	1	21.98677	0.473914	0.009948	0.009726	0.136274
0	0	2	1	22.0691	0.509265	0.002639	0.009699	0.034933
0	0	2	1	22.14688	0.538707	0.016449	0.008881	0.128289
0	0	2	1	22.1963	0.54072	0.022922	0.012832	0.099322
0	0	2	1	22.25143	0.552601	0.027951	0.01096	0.053929
0	0	2	1	22.3878	0.57816	0.045348	0.009113	0.080589
0	0	2	0	20.61992	0.218722	0.046757	0.016552	-0.22188
0	0	2	0	21.04056	0.424579	0.097857	0.023079	1.562294
0	0	2	0	21.42469	0.294683	-0.0512	0.010159	-0.18653

0	0	2	0	21.55793	0.38062	0.009584	0.004218	0.106692
0	0	2	0	21.66521	0.438763	0.019547	0.004563	1.011421
0	0	2	0	21.885	0.560839	0.017453	0.004346	-0.48715
1	1	2	0	22.16983	0.38627	0.019675	0.006159	0.333822
1	1	2	0	22.29269	0.437158	0.035968	0.00943	0.568097
1	1	2	0	22.47249	0.507217	0.042501	0.011829	0.327011
1	1	2	0	22.569	0.544704	0.028595	0.006598	-0.26017
1	1	2	0	22.55952	0.527921	0.029792	0.006616	-0.05965
0	0	1	0	21.08146	0.5961	0.03917	0.005853	0.439645
1	1	1	0	21.41683	0.690658	0.040228	-0.00116	-0.05022
1	1	1	0	22.07265	0.774975	0.046959	-0.00672	0.539856
1	1	1	0	22.30454	0.663968	0.053983	0.009584	0.5661
1	1	1	0	23.14799	0.705205	0.029931	0.00635	0.416779
1	2	1	1	20.2743	0.244056	0.185995	0.05094	0.340104
0	0	1	1	20.78483	0.173044	0.172437	0.064253	0.6433
0	0	1	1	20.98324	0.146175	0.198799	0.055886	0.324262
0	0	1	1	21.15493	0.148274	0.17705	0.044788	0.121054
0	0	1	1	21.31846	0.163491	0.163628	0.047886	0.085437
0	0	1	1	21.48328	0.176153	0.154719	0.054232	0.180773
0	0	1	1	21.79686	0.271255	0.140351	0.05502	0.250883
0	0	1	1	22.4824	0.150889	0.095518	0.053712	0.25139
0	0	1	1	22.61466	0.159571	0.110436	0.03495	0.345275
0	0	1	1	22.65644	0.197751	0.117989	0.040286	0.181228
0	0	1	1	22.79841	0.227117	0.107211	0.041492	0.108176
1	1	2	0	20.15805	0.303667	0.099338	0.006527	0.04859
0	0	2	0	20.21057	0.249045	0.124964	0.008229	0.243241
0	0	2	0	20.35175	0.220234	0.07983	0.009961	0.211569
0	0	2	0	20.5135	0.241865	0.103851	0.010287	0.013338
0	0	2	0	20.88658	0.086935	0.147336	0.009177	0.067325
0	0	2	0	21.0566	0.083129	0.161848	0.015933	0.026586
0	0	2	0	21.18094	0.078856	0.167712	0.0179	-0.03975
0	0	2	0	21.29529	0.085926	0.13794	0.016672	-0.10254
0	0	2	0	21.4041	0.124712	0.098125	0.02118	0.079435
0	0	2	0	21.69504	0.258106	0.034476	0.014572	0.018935
0	0	2	0	21.75376	0.175111	0.053835	0.016189	0.029408
1	1	3	0.666667	21.15003	0.415122	0.116251	0.027624	0.048651
1	1	3	0.666667	21.52167	0.439136	0.092752	0.029496	0.452894
1	1	3	0.666667	21.7904	0.541438	0.06786	0.015313	0.269074
1	1	3	0.666667	21.85663	0.553475	0.056294	0.014985	0.061937
1	1	3	0.666667	22.05384	0.493043	0.054071	0.015883	0.133654
1	1	3	0.666667	22.31239	0.502663	0.049674	0.015719	0.073396
1	1	3	0.666667	22.3632	0.488975	0.026967	0.013739	0.069983
1	1	3	0.666667	22.26654	0.48117	-0.03187	0.009101	-0.16293
1	1	3	0.666667	22.3078	0.477835	0.02573	0.011926	0.203582
1	1	3	0.666667	22.38939	0.475741	0.053228	0.020811	0.381378
1	1	3	0.666667	22.46792	0.478141	0.056292	0.015529	0.064829
0	0	2	0.5	20.84966	0.60538	0.071964	0.002595	0.389645
0	0	2	0.5	21.20935	0.489272	0.063004	0.004179	0.716823
0	0	2	0.5	21.31896	0.509794	0.06983	0.002847	0.261301
0	0	2	0.5	21.48484	0.5713	0.060709	0.014867	0.102413
0	0	2	0.5	21.51529	0.551012	0.081443	0.014863	0.154709
0	0	2	0.5	21.61889	0.555621	0.093124	0.01641	0.11236
0	0	2	0.5	21.81626	0.460042	0.069471	0.015851	0.050065
0	0	2	0.5	22.41503	0.624875	0.102523	0.016263	0.598652
0	0	2	0.5	22.58918	0.57134	0.060137	0.01571	0.918333
0	0	2	0.5	22.67112	0.568854	0.067777	0.017256	0.390762
0	0	1	1	20.53091	0.190964	0.080849	0.00289	0.911633
0	0	1	1	20.49736	0.133092	0.062849	0.005104	-0.24403

0	0	1	1	20.53103	0.204648	-0.07114	-0.01655	-0.14288
0	0	1	1	20.56873	0.228695	0.006664	-0.01019	0.286074
0	0	1	1	20.56011	0.220653	0.009699	-0.0063	-0.11105
0	0	1	1	20.58785	0.224911	0.008752	-0.00805	0.230034
1	1	1	0	22.16207	0.540422	0.086899	0.040121	0.164001
1	1	1	0	22.28867	0.514675	0.10869	0.037647	0.205855
1	1	1	0	22.37742	0.344706	0.174447	0.046101	0.322534
1	1	1	0	22.35615	0.322074	0.07046	0.055761	-0.15731
1	1	1	0	22.41802	0.298451	0.111854	0.016491	0.094698
1	1	1	0	22.41117	0.213263	0.159442	0.041515	0.167867
1	1	1	0	22.52083	0.274122	0.088216	0.026642	-0.13978
1	3	1	0	22.6304	0.304332	0.092406	0.007065	-0.05336
1	1	1	0	23.00486	0.163825	0.098237	0.028124	0.257612
1	1	2	0.5	23.11596	0.179125	0.208297	0.049617	0.452722
1	1	2	0.5	23.18729	0.166774	0.194362	0.052521	0.039268
0	0	1	1	20.75956	0.43747	-0.02292	0.003348	-0.10634
0	0	1	1	20.82588	0.428993	0.06444	-0.01095	0.382222
0	0	1	1	21.47232	0.240729	0.100589	0.03898	0.341018
0	0	1	1	21.44714	0.334521	-0.03612	0.008552	-0.11452
0	0	1	1	21.50199	0.52703	0.089918	0.039664	0.563836
0	0	1	1	21.68808	0.572745	0.17153	0.000697	0.70277
0	0	1	0	20.25035	0.085761	0.058732	0.019855	-0.51818
0	0	1	0	20.17168	0.07976	0.022031	0.008813	0.035312
0	0	1	0	20.21842	0.11183	0.014099	-0.00108	-0.00462
0	0	1	0	20.26641	0.145049	0.014594	0.002442	-0.01799
0	0	1	0	20.51272	0.195251	0.084775	0.062588	0.15992
0	0	1	0	20.39062	0.120857	0.015497	0.00012	-0.04946
1	1	1	0	22.62463	0.426269	0.039733	-0.00017	4.518998
1	3	1	0	22.84403	0.41283	0.065904	0.018405	1.105604
1	3	1	0	22.88205	0.400609	0.051565	0.014089	-0.00869
1	2	2	0	21.86409	0.47083	0.000085	-0.00341	0.193843
1	2	2	0	21.93055	0.495165	0.025217	0.008602	0.716845
0	0	2	0	21.90307	0.477437	0.028912	-0.00188	0.2353
1	1	2	0	21.83154	0.483506	-0.01729	-0.00219	-0.0806
1	1	1	0	21.74698	0.429297	0.023456	0.003312	0.019511
1	1	1	0	21.7096	0.403725	0.02213	0.00236	-0.0352
1	3	1	0	21.59958	0.417177	-0.06337	0.00243	-0.16066
1	3	1	0	21.64849	0.387819	0.024891	-0.01061	-0.02659
1	1	1	1	23.66828	0.779288	0.044571	0.003491	0.218282
1	1	1	1	24.11441	0.798511	0.070235	0.055956	1.1944
1	1	1	1	24.27284	0.805954	0.032929	0.010501	-0.42661
1	1	1	1	24.40121	0.785462	0.051107	0.005313	0.61936
1	1	1	1	24.3673	0.75918	0.035617	0.013005	0.132601
1	1	1	1	24.4664	0.741054	0.053033	0.01671	0.073052
1	1	1	1	24.62614	0.682451	0.059136	0.017097	0.541681
1	1	1	1	24.82167	0.728431	0.052075	0.011511	-0.29192
1	1	1	1	25.1955	0.779396	0.055951	0.012135	0.533028
1	1	1	1	25.58791	0.827101	0.048634	0.008651	0.181811
1	1	2	0	20.64673	0.367171	0.074264	-0.00997	-0.02626
1	1	2	0	20.90732	0.428265	0.127399	0.038249	0.607251
1	1	2	0	21.26851	0.505423	0.116536	0.035567	0.50345
1	1	2	0	21.27997	0.442567	0.123569	0.028444	-0.05028
1	1	2	0	21.39275	0.455659	0.106514	0.029114	-0.15809
1	1	2	0	21.64061	0.319755	0.105278	0.02433	0.084998
1	1	2	0	21.63466	0.230669	0.138472	0.029764	0.044142
1	1	2	0	22.33805	0.46355	0.066442	0.021107	0.21561
1	1	2	0	22.59791	0.443052	0.10969	0.023833	0.863157
1	1	2	0	22.72189	0.457983	0.070192	0.025397	0.185795

1	3	2	0	22.65625	0.574614	0.035978	0.015731	-0.18202
0	0	2	0.5	20.09196	0.192881	0.070883	0.009614	0.119944
1	1	2	0.5	20.11933	0.165298	0.073676	0.012315	0.515921
1	1	2	0.5	20.24069	0.217834	0.071395	0.01112	0.301012
1	1	2	0.5	20.36593	0.240179	0.095904	0.010478	0.292529
0	0	2	0.5	20.83538	0.182416	0.083101	0.008807	0.215031
0	0	2	0.5	20.93026	0.181827	0.090945	0.011034	0.156145
0	0	2	0.5	21.0113	0.190341	0.072403	0.011186	0.032449
0	0	4	0.5	22.29901	0.078169	0.05474	0.012063	0.607034
0	0	1	0	20.9657	0.430921	0.137674	0.030721	0.23564
0	0	1	0	21.25703	0.482223	0.150212	0.031436	0.472072
0	0	1	0	21.73933	0.34267	0.130968	0.04488	0.301298
0	0	1	0	22.07112	0.445568	0.121932	0.033814	0.150822
0	0	1	0	22.24994	0.412126	0.11997	0.028895	0.139742
0	0	1	0	22.48063	0.398435	0.117962	0.030561	0.184635
0	0	1	0	22.57096	0.340985	0.117576	0.030816	0.004391
0	0	1	0	22.5152	0.267792	0.055764	0.022483	-0.22668
1	1	1	0	22.88112	0.348399	0.029971	0.015242	0.325259
1	1	1	0	22.87786	0.336126	0.007989	0.011483	0.001641
1	1	1	0	22.98618	0.37875	0.033944	0.027848	0.041238
0	0	1	1	21.83575	0.486833	0.068184	0.012354	0.100741
0	0	1	1	22.06332	0.589983	0.05688	0.007711	0.18251
0	0	1	1	22.36822	0.663188	0.064492	0.034524	0.246507
0	0	1	1	22.52826	0.678836	0.076287	0.034452	0.185618
0	0	1	1	22.85626	0.604818	0.060041	0.028486	0.138146
0	0	1	1	22.89772	0.615598	0.051933	0.029052	0.079812
0	0	1	1	23.23119	0.592982	0.025709	0.019522	0.256556
0	0	1	1	23.40064	0.559716	0.019324	0.013729	0.001216
0	0	1	1	23.49707	0.595361	0.019099	0.01561	0.115059
0	0	1	1	23.7635	0.628955	0.019342	0.015871	0.066518
0	0	1	1	23.91694	0.678641	0.022493	0.014161	0.068677
1	1	2	1	20.53116	0.074991	0.179842	0.026997	0.250627
1	1	2	1	20.91245	0.156247	0.215194	0.02385	0.167948
1	1	2	1	21.17761	0.300524	0.11653	0.017522	-0.25098
1	1	2	1	22.96179	0.080512	0.065632	0.023958	1.658829
1	1	2	1	23.17047	0.073005	0.150238	0.046897	0.52555
1	3	2	1	23.30542	0.113061	0.146835	0.031625	0.155429
1	3	2	1	23.39434	0.136646	0.071962	0.019267	-0.17131
1	3	2	1	23.19585	0.030536	0.059829	0.022499	0.432731
1	2	1	1	19.92533	0.14065	0.064119	0.009795	0.082051
1	2	1	1	20.01778	0.183818	0.085491	0.002508	0.139919
1	1	1	1	20.58869	0.129123	0.060867	0.005371	0.196905
1	1	1	1	20.68752	0.162867	0.063315	0.003392	0.177128
1	1	1	1	20.70026	0.124186	0.074605	0.004197	0.261148
1	1	1	1	20.74585	0.175961	0.014411	0.005539	-0.13881
1	1	1	1	21.19768	0.181705	0.024826	0.004877	0.182296
1	1	1	1	21.13969	0.134268	0.028032	0.005018	0.209409
1	1	1	1	21.14715	0.112842	0.03302	0.003472	-0.15635
1	1	1	1	21.19594	0.12738	0.034449	0.004286	0.274814
1	1	1	1	21.26042	0.153886	0.037074	0.001886	0.007975
0	0	1	1	21.41189	0.441443	0.076426	0.013365	0.301343
0	0	1	1	21.49317	0.419011	0.072137	0.011631	0.071097
0	0	1	1	21.53804	0.395213	0.07845	0.01569	0.308598
0	0	1	1	21.65052	0.392677	0.075692	0.014315	0.021824
0	0	1	1	21.77681	0.436005	0.043338	0.012687	0.055804
0	0	1	1	21.89739	0.483799	0.026144	0.005434	-0.05191
0	0	1	1	22.02138	0.358462	0.034264	0.006111	0.067415
0	0	1	1	22.02898	0.339084	0.029527	0.005303	-0.2891

0	0	1	1	22.6238	0.486502	0.019234	0.007309	0.099525
0	0	1	1	22.74885	0.473721	0.003733	0.007043	0.665227
1	1	1	1	21.13788	0.66542	0.039843	0.001651	0.029202
1	1	1	1	21.41215	0.576424	0.045144	0.009727	0.219812
1	1	1	1	21.59781	0.657538	0.028158	0.001988	0.311011
0	0	1	1	21.61619	0.658094	0.042315	0.004553	-0.11965
0	0	1	1	21.68203	0.670508	0.035378	0.000412	-0.09719
0	0	1	1	21.41198	0.486588	0.042942	0.000335	0.045441
0	0	1	1	21.88039	0.616891	0.022128	-0.00186	-0.12159
0	0	1	1	22.03069	0.489309	0.064559	0.000014	1.367843
0	0	1	1	21.80086	0.334685	0.074878	0.004624	-0.2428
0	0	2	1	20.352	0.09736	0.131071	0.029558	0.175598
0	0	2	1	20.54839	0.109766	0.14217	0.028971	0.15754
0	0	2	1	20.65523	0.105248	0.086269	0.029286	0.040146
0	0	2	1	20.6938	0.107412	0.038708	0.0169	0.164484
0	0	2	1	20.76478	0.132283	0.044031	0.011943	0.292722
0	0	2	1	21.03771	0.188639	0.056286	0.022422	0.175817
1	3	2	1	22.1873	0.175296	0.052697	0.054162	0.168457
1	3	2	1	22.16859	0.10947	0.052319	0.018783	0.33801
1	3	2	1	22.19763	0.114822	0.01417	0.017653	0.087825
0	0	1	0	21.83751	0.180625	0.00188	0.002412	0.046191
0	0	1	1	21.48389	0.540015	0.059383	0.012209	-0.0784
0	0	1	1	21.69093	0.355794	0.066691	0.017602	0.307363
0	0	1	1	21.93441	0.112836	0.043242	0.014626	-0.12601
0	0	1	1	21.95848	0.193284	-0.01711	0.005312	-0.34244
0	0	1	1	21.98868	0.232716	0.011414	-0.00153	-0.07988
0	0	1	1	21.9745	0.256914	0.013874	0.001353	0.233524
0	0	1	1	22.16177	0.285456	0.01323	0.001766	-0.02149
0	0	1	1	22.10231	0.297207	-0.0325	0.000562	0.058474
0	0	1	0	22.66728	0.47951	0.053737	0.024825	-0.16083
0	0	1	0	22.66272	0.553672	-0.00227	0.012299	-0.04924
0	0	1	0	22.54575	0.492186	0.044837	0.013453	0.03565
0	0	1	0	22.6127	0.571115	-0.03535	0.007644	-0.00718
0	0	1	0	22.69859	0.600816	0.017938	0.009398	0.186178
1	3	2	1	21.43254	0.173641	0.118216	0.003351	1.005609
0	0	2	1	21.54528	0.174362	0.119353	0.01735	0.719496
0	0	2	1	21.87787	0.312621	0.123742	0.018035	0.744717
0	0	2	1	22.12191	0.420221	0.05963	0.012435	-0.35897
0	0	2	1	22.06933	0.372923	0.061298	0.014728	0.037124
1	3	2	0	19.95417	0.086986	0.135317	0.019777	0.018375
1	3	2	0	20.12757	0.130857	0.193309	0.046297	0.765505
1	3	2	0	20.78	0.079793	0.14086	0.055309	0.305146
1	3	2	0	20.89443	0.099345	0.149748	0.011309	0.368098
1	3	2	0	20.94404	0.084237	0.107921	0.02338	0.057193
1	3	2	0	21.31001	0.205425	0.103661	0.027896	0.560113
1	3	2	0	21.85011	0.120511	0.056589	0.022707	0.209099
1	3	2	0	21.94828	0.138659	0.08287	0.015892	0.422015
1	3	2	0	22.36313	0.311571	0.083244	0.0237	0.277107
1	3	2	0	22.45939	0.294704	0.097137	0.014605	0.083887
1	3	2	0	22.57822	0.246824	0.093585	0.013299	0.289814
1	1	2	1	21.44687	0.135315	0.184462	0.181426	0.1264
1	1	2	1	21.407	0.091594	0.155013	0.03062	-0.11466
1	1	2	1	21.51218	0.099126	0.157879	0.028964	0.181319
1	1	2	1	21.6336	0.163879	0.160091	0.031433	0.128911
1	1	2	1	21.7125	0.180766	0.188925	0.033251	0.107575
1	1	2	1	21.74702	0.166354	0.186081	0.029716	0.009442
1	1	2	1	21.73839	0.150954	0.155592	0.021415	-0.06447
1	1	2	1	21.77752	0.154802	0.162191	0.02945	0.111824

1	1	2	1	21.89561	0.178245	0.169469	0.030605	0.059527
1	1	2	1	21.94884	0.175996	0.141506	0.030196	-0.0194
1	1	2	1	21.95162	0.175717	0.129275	0.030684	0.035181
1	3	1	0	21.12179	0.334086	0.078573	0.070726	-0.0822
1	3	1	0	21.35009	0.425431	0.070699	0.007099	0.445591
1	3	1	0	21.58048	0.327231	0.062778	0.011586	0.21321
1	1	1	0	22.08812	0.406001	0.064657	0.008835	0.480201
1	3	1	0	22.1158	0.373443	0.07304	0.00983	0.260894
1	3	1	0	22.26613	0.416784	0.066965	0.009289	0.204159
1	3	1	0	22.34145	0.391922	0.070059	0.009211	0.171063
1	3	1	0	22.33908	0.387094	0.051236	0.008409	-0.06828
1	3	1	0	22.67533	0.433951	0.025253	-0.00445	0.161308
1	3	2	0	22.71712	0.450775	0.029313	-0.00044	0.179666
1	1	2	0	22.75114	0.436836	0.044055	0.001197	0.115056
1	1	1	1	21.69189	0.423203	0.075735	0.093973	0.2376
1	1	1	1	21.90478	0.462787	0.086118	0.042141	0.297422
1	1	1	1	22.314	0.592475	0.0816	0.053686	0.330472
1	1	1	1	22.36814	0.496785	0.099051	0.051157	0.205755
1	1	1	1	22.60144	0.528077	0.081045	0.043639	0.047102
1	1	1	1	22.79121	0.554804	0.058901	0.039303	-0.00572
1	1	1	1	23.07718	0.641219	0.042999	0.02391	0.038311
1	1	1	1	23.19214	0.520519	0.028712	0.014317	0.007754
1	1	1	1	23.284	0.527469	0.03448	0.020467	0.165239
1	1	1	1	23.4521	0.505125	0.042729	0.024074	-0.00528
1	1	1	1	23.49499	0.527707	0.031251	0.014269	-0.13436
0	0	2	1	20.94622	0.153272	0.07549	0.048836	-0.2184
0	0	2	1	20.96936	0.132776	0.063068	0.00913	0.038983
0	0	2	1	21.12286	0.209022	0.05737	0.054784	0.377377
0	0	2	1	21.16495	0.24166	0.033697	0.003499	0.228988
0	0	2	1	21.1863	0.242917	0.030827	0.005011	0.019718
1	1	2	1	21.36328	0.344916	0.03174	0.005363	-0.12199
1	1	2	1	20.55716	0.0872	0.083671	0.051714	-0.0991
1	1	2	1	20.5781	0.087233	0.089931	0.017215	0.169877
1	1	2	1	20.80703	0.25548	0.067533	0.005981	0.368137
1	1	2	1	20.90605	0.347289	0.015433	0.010197	-0.03523
1	1	2	1	20.8001	0.2778	0.037663	0.008135	0.045854
1	1	2	1	20.7368	0.212732	0.040845	0.007367	-0.03107
1	1	2	1	20.75856	0.245289	0.014158	0.006623	-0.20799
1	1	2	1	20.75287	0.221763	0.016265	0.005379	-0.01017
1	1	2	1	21.57925	0.253378	0.030406	0.008212	0.37687
0	0	2	1	21.6954	0.193515	0.039417	0.009953	0.78044
0	0	2	1	21.73131	0.182321	0.03878	0.010533	0.108325
0	0	2	1	21.74174	0.438472	0.030455	0.010845	-0.0314
0	0	2	1	21.88772	0.505519	0.022686	0.00623	-0.05176
0	0	3	1	21.89968	0.44619	0.08589	0.026238	0.202798
0	0	3	1	22.1367	0.358473	0.112784	0.018489	0.564492
0	0	3	1	22.29381	0.358148	0.119425	0.024149	0.065646
0	0	3	1	22.84747	0.415478	0.073676	0.014194	0.297971
0	0	3	1	22.8472	0.412868	0.020269	0.010635	0.083851
0	0	3	1	22.5549	0.344321	0.029759	0.015585	-0.03975
0	0	3	0.666667	21.16543	0.138343	0.125899	0.077005	0.2443
0	0	3	0.666667	21.35244	0.212972	0.140816	0.041328	0.588059
0	0	3	0.666667	21.49021	0.233462	0.194699	0.057663	0.309883
0	0	3	0.666667	21.57332	0.191279	0.180958	0.055001	0.143768
0	0	3	0.666667	21.69611	0.196572	0.140491	0.041201	-0.07367
0	0	3	0.666667	21.85945	0.201463	0.13812	0.039792	0.093967
0	0	3	0.666667	21.97169	0.237999	0.142754	0.042518	0.055853
0	0	3	0.666667	22.16032	0.315575	0.097961	0.037991	0.081143

0	0	3	0.666667	22.24675	0.312857	0.124572	0.031919	0.478912
0	0	3	0.666667	22.33416	0.226416	0.128064	0.040149	0.032381
0	0	3	0.666667	22.40419	0.220335	0.119009	0.033922	0.009846
0	0	2	0.5	22.79074	0.130756	0.21533	0.06877	0.119915
0	0	2	0.5	22.7688	0.137965	0.109905	0.047958	-0.039
1	1	1	1	21.43451	0.415813	0.052418	0.018413	-0.10976
1	1	1	1	20.43116	0.160881	0.122747	-0.00234	0.504506
1	1	1	1	20.70983	0.266382	0.10866	-0.01507	0.378238
1	1	1	1	20.84796	0.359742	0.036859	-0.00143	-0.14774
1	1	1	1	21.31704	0.171096	0.045483	0.006451	0.318254
1	1	1	1	21.37957	0.280007	0.059583	0.002984	0.356261
1	1	1	1	21.48546	0.322661	0.047405	0.003368	0.076867
1	1	1	1	21.5275	0.30994	0.051043	0.004476	-0.09084
1	1	1	1	21.5337	0.371909	-0.05091	0.006325	0.062734
1	1	1	1	21.44062	0.287727	0.03131	0.004835	-0.03325
1	1	1	1	21.34775	0.166928	0.041714	0.004322	-0.04596
0	0	1	0	21.50222	0.275048	0.110231	0.110231	0.1099
0	0	1	0	21.88776	0.443218	0.098005	0.013931	0.439477
0	0	1	0	22.34123	0.261725	0.105217	0.017412	0.503417
0	0	1	0	22.61478	0.476076	0.00889	0.014008	0.317532
0	0	1	0	22.8275	0.588067	-0.01018	-0.00767	0.148544
0	0	1	0	23.0297	0.637241	0.03173	-0.00839	0.366981
0	0	1	0	23.11759	0.490957	-0.01875	-0.0125	0.078272
0	0	1	0	23.27753	0.469251	0.080256	0.010415	0.320311
0	0	1	0	23.30364	0.504493	0.043516	0.004758	0.108708
0	0	1	0	23.4081	0.447834	0.123638	0.01538	0.136673
1	1	2	0.5	21.54564	0.411369	0.094855	0.094855	0.0609
1	1	2	0.5	22.02811	0.597501	0.068701	0.001818	0.126602
1	1	2	0.5	22.30189	0.642331	0.084164	0.000671	0.538536
1	1	2	0.5	22.59478	0.673737	0.091822	0.00319	1.170052
0	0	1	1	22.21889	0.196367	0.046525	0.008128	0.028162
0	0	1	1	21.6753	0.179276	0.093366	0.093366	0.026
1	3	1	1	21.90015	0.281881	0.071388	0.01879	0.721525
1	3	1	1	22.73944	0.640724	0.044701	0.014939	0.644145
1	1	1	1	23.2147	0.752493	0.057531	0.006551	0.31819
1	1	1	1	23.44658	0.789042	0.057557	0.000939	0.276542
1	1	1	1	23.56321	0.697025	0.056329	-0.00139	0.145017
1	1	1	1	23.78421	0.734534	0.054935	0.005231	0.312988
1	1	1	1	23.93904	0.764072	0.025592	0.008302	-0.06988
1	1	1	1	24.18366	0.70903	0.039184	-0.00385	0.719411
1	1	1	1	23.96508	0.650214	0.021723	0.008471	-0.13848
0	0	1	1	23.78722	0.595186	0.040727	0.004476	-0.29302
1	1	1	0	20.50205	0.248495	0.070192	0.070192	0.1816
1	1	1	0	20.60812	0.285107	0.061763	-0.00395	0.295584
1	1	1	0	20.76562	0.335831	0.057588	-7.7E-05	0.175421
1	1	1	0	20.96268	0.374616	0.062672	-0.01073	0.498727
1	1	1	0	21.28947	0.503302	0.069547	-0.00464	0.366492
1	1	1	0	21.77371	0.424074	0.045161	-0.00313	0.398513
1	1	1	0	22.65117	0.354844	0.005414	-0.01352	0.135249
1	3	1	0	21.99044	0.081199	-0.0874	-0.02484	-0.11005
1	3	1	0	22.77481	0.59146	0.010138	-0.01197	1.662038
0	0	2	0.5	20.64133	0.175474	0.043147	0.043147	0.0743
0	0	2	0.5	20.85332	0.301963	0.045801	0.001457	0.677517
0	0	2	0.5	20.96899	0.362342	0.044694	0.003394	0.338893
1	3	2	0.5	21.37236	0.562533	0.024977	0.007861	-0.17893
0	0	2	0	21.06231	0.077271	0.084575	0.084575	0.139
0	0	2	0	21.11678	0.064453	0.090763	0.014265	0.161551
1	1	2	0	21.17417	0.058951	0.099686	0.019509	0.171572

1	1	2	0	21.27549	0.086588	0.106831	0.023442	0.180897
1	1	2	0	21.40686	0.120161	0.100766	0.029596	0.229678
1	1	2	0	21.55894	0.176739	0.107777	0.029034	0.200443
1	1	2	0	21.92745	0.367266	0.104151	0.033308	0.205745
1	3	2	0	22.16438	0.189622	0.115261	0.031904	0.07273
1	3	2	0	22.3035	0.231822	0.101916	0.029757	0.160562
1	3	2	0	22.38041	0.246639	0.096692	0.02825	0.202253
0	0	1	1	20.59315	0.046918	0.083316	0.083316	-0.0314
0	0	1	1	20.60804	0.056619	0.0555	0.01705	-0.12407
0	0	1	1	20.66247	0.077405	0.052619	0.014702	0.126539
0	0	1	1	20.68749	0.108522	0.039547	0.012108	0.135445
0	0	1	1	20.72209	0.142506	0.031614	0.007694	0.048864
0	0	1	1	20.69882	0.125995	0.014007	0.00477	0.24848
0	0	1	1	21.12626	0.312542	0.059759	0.059759	0.247
0	0	1	1	21.24226	0.337245	0.06836	0.014144	0.150943
0	0	1	1	21.41592	0.348217	0.083107	0.016008	0.437219
0	0	1	1	21.63842	0.403428	0.107255	0.026201	0.310929
1	1	1	1	22.02587	0.501559	0.092396	0.026767	0.247705
1	1	1	1	22.41204	0.462029	0.067579	0.024952	-0.04315
1	1	1	1	22.63077	0.516833	0.064194	0.016697	-0.11386
1	1	1	1	22.94196	0.623138	0.022459	0.014351	-0.04298
1	1	1	1	23.13824	0.662474	0.029051	0.008685	0.15771
0	0	1	1	23.19841	0.68508	-0.01714	0.010531	0.178599
0	0	2	1	23.18198	0.684281	0.021201	0.012664	0.026716
0	0	2	1	20.95709	0.316628	0.062913	0.062913	0.2537
0	0	2	1	20.93477	0.286415	0.058746	0.01174	0.036979
0	0	2	1	21.23253	0.406678	0.056579	0.020946	0.420175
0	0	2	1	21.29036	0.40355	0.056057	0.017109	0.155858
0	0	2	1	21.38905	0.462521	0.034612	0.011625	-0.08255
0	0	2	1	21.6468	0.313191	0.051059	0.013601	0.379181
0	0	2	1	21.95788	0.443829	0.053995	0.017506	0.452743
0	0	2	1	22.21956	0.519081	0.082585	0.016012	0.133931
0	0	2	1	22.49207	0.523884	0.058814	0.03948	0.573386
0	0	2	1	22.61763	0.524331	0.09518	0.025598	0.197793
0	0	2	1	22.6499	0.510481	0.067448	0.024282	0.040837
0	0	2	0	20.9599	0.177355	0.08897	0.077166	0.1631
0	0	2	0	21.09535	0.229004	0.105605	0.035157	0.347968
0	0	2	0	21.24985	0.268861	0.15785	0.031767	0.361771
0	0	2	0	21.45294	0.271024	0.166541	0.046774	0.222613
0	0	2	0	21.59138	0.243683	0.174037	0.055121	0.048918
0	0	2	0	21.74915	0.224017	0.172923	0.055692	0.056801
0	0	2	0	21.79753	0.218471	0.161941	0.044419	0.062363
0	0	2	0	21.98406	0.245903	0.15698	0.037712	0.104638
0	0	2	0	22.1127	0.217126	0.14958	0.028618	0.131786
0	0	2	0	22.24322	0.221411	0.140586	0.02668	0.115543
0	0	2	0	22.27134	0.242053	0.128482	0.021838	-0.04442
0	0	2	1	21.60084	0.092833	0.103309	0.103309	-0.1455
1	1	2	1	21.59616	0.083702	0.068432	0.019516	0.08245
1	1	2	1	21.71685	0.109568	0.024055	0.008431	0.099388
1	1	1	1	21.81971	0.150413	0.010555	-0.00157	0.129836
1	1	1	1	22.03091	0.291029	0.034825	0.006867	0.674092
0	0	1	1	22.07751	0.314529	0.052421	0.013666	0.374014
0	0	1	0	21.2159	0.211586	0.060279	0.045651	-0.0708
1	1	1	0	21.08836	0.112865	0.041267	0.022949	0.293019
1	1	1	0	21.15009	0.112932	0.031986	0.016754	0.340172
1	1	1	0	21.16611	0.14123	0.033869	0.019037	0.025632
1	1	1	0	21.19211	0.143964	0.02896	0.018489	0.039051
1	1	1	0	21.21359	0.200159	0.027653	0.020174	-0.20926

1	1	1	0	21.27047	0.241436	0.022441	0.016168	-0.04401
1	1	1	0	21.30194	0.259781	0.01113	0.010329	0.015861
1	1	1	0	21.23488	0.20197	0.007671	0.004789	0.020985
1	1	1	0	21.30783	0.255917	0.005345	0.006623	0.254793
1	1	1	0	21.37574	0.306992	0.006902	0.009099	0.162464
1	1	2	0	21.0392	0.150067	0.086581	0.086581	0.15
1	1	2	0	21.0063	0.107176	0.052864	0.007209	0.031202
1	1	2	0	21.05876	0.100458	0.057462	0.010173	0.146495
1	1	2	0	21.09573	0.099833	0.059005	0.010724	0.030461
1	1	2	0	21.12292	0.104368	0.061623	0.009175	-0.06517
1	1	2	0	21.3169	0.260388	0.032423	0.0059	0.014913
1	1	2	0	21.20921	0.095869	0.144472	-0.0106	-0.00014
1	1	2	0	21.28985	0.114523	0.110615	-0.00305	-0.09535
1	1	2	0	21.21152	0.093196	-0.01768	-0.00737	0.0566
1	1	2	0	21.32221	0.272214	-0.08847	-0.01739	0.068276
0	0	1	1	21.92042	0.703912	0.08042	0.177675	0.1407
1	1	1	1	22.71479	0.565756	0.006496	0.003913	-0.01525
0	0	1	1	20.44027	0.600721	0.157111	0.157111	-0.0683
0	0	1	1	21.19779	0.213661	0.059498	0.015453	-0.17596
0	0	1	1	21.19572	0.208672	0.011892	0.003093	0.036254
1	1	1	1	21.23795	0.245301	-0.00145	0.0021	-0.14668
1	1	1	1	21.32491	0.261304	0.027318	0.002574	0.483125
1	1	1	1	21.28991	0.239868	0.030493	0.006483	0.072961
1	1	1	1	21.2478	0.203637	-0.06093	0.007603	-0.50893
1	1	1	1	21.26023	0.212617	0.008997	-0.00468	0.057629
1	1	1	1	21.22912	0.1954	0.021574	-0.00302	0.513411
1	1	1	1	21.14633	0.145606	-0.00112	-0.00325	-0.20617
1	1	1	1	21.17926	0.17096	0.003026	-0.01242	0.17191
0	0	1	1	21.89901	0.337324	0.076612	0.01494	0.081876
0	0	1	1	22.02429	0.389505	0.067006	0.016844	0.05726
0	0	1	1	22.14045	0.438836	0.049978	0.012705	0.081742
0	0	1	1	22.19368	0.45557	0.037399	0.011627	0.02672
0	0	1	1	22.11982	0.410612	0.028465	0.005479	-0.08472
0	0	1	1	22.0788	0.379184	0.02622	0.0064	-0.14107
0	0	1	1	22.12709	0.405616	0.020016	0.006458	0.022947
0	0	1	1	22.13631	0.416538	0.009601	0.006582	0.037235
0	0	1	1	22.10847	0.395573	0.018488	0.00526	0.19012
0	0	1	1	22.11235	0.394053	0.020672	0.006156	0.166641
0	0	3	0.333333	20.06055	0.320691	0.19652	0.189319	0.2674
0	0	3	0.333333	21.09501	0.071073	0.076722	0.026911	0.459761
0	0	3	0.333333	21.31259	0.215251	0.102028	0.030721	0.588785
0	0	3	0.333333	21.44672	0.279408	0.082937	0.03467	0.163553
0	0	3	0.333333	21.95167	0.286358	0.069397	0.028433	0.351784
0	0	3	0.333333	22.19709	0.44327	0.071194	0.0284	0.191095
0	0	3	0.333333	22.19942	0.412786	0.085535	0.025006	0.08919
0	0	3	0.333333	22.24352	0.404389	0.071317	0.024795	0.017828
0	0	3	0.333333	22.4099	0.359137	0.072665	0.026183	0.126885
0	0	3	0.333333	22.42152	0.388691	0.030627	0.02225	0.052441
0	0	3	0.333333	22.3871	0.35204	0.036387	0.022108	0.090748
0	0	2	0.5	20.78444	0.244961	0.052908	0.022167	-0.0988
0	0	2	0.5	20.88099	0.279388	0.066394	0.016371	0.431182
0	0	2	0.5	20.93387	0.307012	0.043768	0.01604	0.008678
0	0	2	0.5	21.225	0.438965	0.015182	0.014047	-0.1198
0	0	2	0.5	21.25731	0.292622	0.010926	0.009877	0.041962
0	0	2	0.5	21.27184	0.300083	0.014009	0.003026	0.007499
0	0	2	0.5	21.3111	0.321056	0.013479	0.002677	-0.02393
0	0	2	0.5	21.4146	0.431908	-0.04734	0.002687	0.194664
0	0	2	0.5	21.52253	0.483251	0.021958	0.003398	0.702768

0	0	2	0.5	21.50724	0.46303	0.017627	0.005032	0.096652
0	0	1	1	20.91165	0.066602	0.083591	0.022969	0.058906
0	0	1	1	20.89759	0.059336	0.053532	0.013927	0.112418
0	0	1	1	20.87943	0.007969	0.063917	0.017319	0.085501
0	0	1	1	20.89957	0.009063	0.037119	0.014427	-0.01982
0	0	1	1	20.93984	0.019687	0.035399	0.011291	-0.00624
0	0	1	1	20.99986	0.057472	0.02722	0.009051	-0.02636
0	0	1	1	21.27168	0.19919	0.047534	0.008114	0.638515
0	0	1	1	21.3441	0.237198	0.021271	0.00543	0.255758
0	0	1	0	19.72915	0.577115	0.148816	0.146729	0.0542
0	0	1	0	20.51359	0.116906	0.036575	0.011979	0.141697
0	0	1	0	20.7565	0.304932	0.05065	0.011007	0.461371
0	0	1	0	20.76064	0.314254	0.059799	0.015446	0.205886
0	0	1	0	20.86409	0.370317	0.050072	0.015799	-0.1337
0	0	1	0	20.68652	0.255236	0.040694	0.008973	0.125557
0	0	1	0	20.60156	0.200369	0.035351	0.007731	-0.17742
0	0	2	0.5	21.29738	0.336121	0.073294	0.022041	0.254724
1	3	1	1	19.73767	0.524508	0.147274	0.02534	0.3217
1	3	1	1	20.45102	0.328469	0.07403	0.022837	0.009999
1	3	1	1	20.5727	0.300058	0.12446	0.011096	0.258584
1	3	1	1	20.74715	0.352606	0.09672	0.034676	0.410959
1	3	1	1	20.80682	0.364365	0.084435	0.032431	0.142585
1	3	1	1	20.91806	0.349707	0.116902	0.039361	0.05118
1	3	1	1	21.77185	0.618331	0.082275	0.036705	2.300454
1	3	1	1	21.79358	0.608926	0.061205	0.016828	-0.07825
1	3	1	1	21.72172	0.556496	0.063222	0.022828	-0.09675
0	0	1	0	20.35574	0.286849	0.057246	0.019544	0.328
0	0	1	0	20.44068	0.297562	0.075288	0.011818	0.2844
0	0	1	0	20.7675	0.436209	0.072771	0.019484	0.185564
0	0	1	0	20.92128	0.490584	0.070515	0.016667	0.192838
0	0	1	0	21.32573	0.353297	0.069065	0.017939	0.271852
0	0	1	0	21.5768	0.265851	0.068165	0.016692	-0.05674
0	0	1	0	21.67894	0.288761	0.07659	0.016174	0.198923
0	0	1	0	21.87825	0.38388	0.049543	0.010606	0.293549
0	0	1	0	21.94273	0.452983	0.031715	0.008605	0.060111
0	0	1	0	22.01159	0.495199	0.051537	0.009481	0.072668
0	0	2	0.5	21.18447	0.438984	0.056732	0.015499	0.1301
0	0	2	0.5	21.30473	0.478419	0.052985	0.018193	0.122142
0	0	2	0.5	21.37921	0.50221	0.047244	0.014883	-0.02411
0	0	2	0.5	21.53348	0.406724	0.047731	0.013383	0.119265
0	0	2	0.5	21.55978	0.398035	0.035916	0.013114	0.093517
0	0	2	0.5	21.50568	0.347157	0.030383	0.0146	0.11992
0	0	2	0.5	21.71726	0.284221	0.024574	0.012813	0.061496
0	0	2	0.5	21.91588	0.300395	0.025007	0.012065	0.171808
0	0	2	0.5	21.96019	0.310636	0.024825	0.010251	0.101987
0	0	2	0.5	21.95809	0.294101	0.019277	0.00949	-0.14084
0	0	1	0	20.57821	0.167663	0.057066	0.021076	0.0129
1	1	1	0	20.58605	0.145086	0.054148	0.018795	-0.0342
1	1	1	0	20.5224	0.139456	-0.0442	0.002663	-0.24812
0	0	1	0	20.50245	0.114778	0.002546	-0.00796	-0.00762
0	0	2	0	20.55901	0.119281	0.052824	0.020325	0.197568
0	0	2	0	21.44643	0.615151	0.031376	0.018001	0.233076
0	0	2	0	21.61204	0.109242	0.05354	0.019762	0.646009
0	0	2	0	21.7698	0.200024	0.055494	0.02124	0.189583
0	0	2	0	21.98699	0.328497	0.052594	0.027122	0.248684
1	1	2	0.5	20.3303	0.203935	0.069628	0.023757	0.3018
1	1	2	0.5	20.53242	0.348989	0.040293	0.009069	0.470255
1	1	2	0.5	20.90016	0.306488	0.061946	0.022554	0.054339

1	1	2	0.5	20.96036	0.341898	0.055323	0.015434	0.069974
1	1	2	0.5	21.78497	0.345013	0.021945	0.012468	0.062551
1	1	2	0.5	22.05755	0.470239	0.035764	0.008654	0.933959
1	1	2	0.5	22.38728	0.404183	0.031805	0.009099	0.288353
1	1	2	0.5	22.57515	0.496163	0.014177	0.000026	0.062261
1	1	2	1	22.21888	0.459316	0.064761	0.016444	0.8044
1	1	2	1	22.63222	0.573745	0.079743	0.01516	0.615983
1	1	2	1	23.01464	0.639157	0.076741	0.018821	0.32042
1	3	2	1	23.26452	0.650426	0.086512	0.018623	0.267989
1	3	2	1	23.59909	0.619309	0.071015	0.02021	0.063754
1	3	2	1	23.71579	0.609442	0.023347	0.00594	0.014764
1	3	2	1	23.76378	0.611699	0.024837	0.005709	0.014409
1	1	2	1	23.79735	0.617735	0.029252	0.006068	0.172418
0	0	1	1	21.87546	0.347069	0.053475	0.011749	0.044231
0	0	1	1	21.86285	0.390272	-0.03769	0.001246	-0.34088
0	0	1	1	21.92091	0.410944	0.030464	-0.00357	-0.02415
0	0	1	1	22.00355	0.453375	0.033073	0.004763	0.395929
0	0	1	1	22.11076	0.500452	0.037788	0.009682	0.021113
0	0	1	1	22.02857	0.541482	-0.05676	0.008324	-0.02218
0	0	1	0	20.66379	0.096912	0.060372	0.009985	0.1764
0	0	1	0	20.67414	0.080662	0.040095	0.010719	0.02816
0	0	1	0	20.71391	0.100166	0.022689	0.004903	-0.02091
0	0	1	0	20.79204	0.146083	0.021813	0.003435	0.052881
0	0	1	0	20.83806	0.153503	0.034011	0.004153	0.294894
0	0	1	0	20.95092	0.211579	0.053145	0.001812	0.344535
0	0	1	0	21.53514	0.120763	0.038573	0.000603	0.213183
0	0	1	0	21.60425	0.142944	0.046413	0.002729	0.15265
0	0	1	0	21.6184	0.148033	0.045882	0.002013	0.030343
0	0	1	0	21.73596	0.179156	0.044022	0.005155	0.232019
0	0	2	0	20.82	0.199828	0.044219	0.007161	0.3153
1	1	2	0	20.97999	0.30003	0.035925	0.007226	0.389788
1	1	2	0	20.99752	0.251228	0.10037	0.017504	0.032754
1	1	2	0	21.05371	0.203009	0.129856	0.026865	-0.04296
1	1	2	0	21.13585	0.21046	0.105317	0.024809	-0.02695
1	1	2	0	21.1451	0.195914	0.07809	0.01612	-0.1341
1	1	2	0	21.60963	0.265439	0.019156	0.007872	0.123271
1	1	2	0	21.4529	0.182369	0.064037	0.004713	0.061506
1	1	2	0	21.47893	0.198059	0.081627	0.014265	0.193577
1	1	2	0	21.56829	0.23954	0.124286	0.030823	0.123062
0	0	2	1	21.25221	0.179473	0.064233	0.033038	0.4978
0	0	2	1	21.3822	0.24972	0.033382	0.020528	0.334167
0	0	2	1	21.73109	0.518023	-0.03371	0.005959	0.543343
0	0	2	1	21.91153	0.589591	0.020729	0.007268	0.454514
0	0	2	1	22.35526	0.704895	0.026558	0.00497	0.333703
0	0	2	1	22.65659	0.606763	0.018111	0.004604	0.133135
0	0	2	1	23.44053	0.818564	0.024189	0.006476	1.104574
0	0	2	1	23.81945	0.647614	0.042569	0.007527	0.831372
0	0	2	1	24.16162	0.729067	0.048894	0.011467	0.288238
0	0	2	1	24.17864	0.725376	0.046022	0.013713	0.188011
1	1	1	0	21.02854	0.096447	0.073963	0.058477	0.091
1	1	1	0	21.09093	0.089151	0.085098	0.012235	0.340328
1	1	1	0	21.18081	0.13732	0.04205	0.004865	-0.11005
0	0	1	0	21.31068	0.242602	0.018914	0.008213	-0.08614
1	1	1	0	21.50064	0.369426	0.015857	0.006438	-0.16325
1	1	2	1	20.27729	0.101902	0.054527	0.015964	0.3958
0	0	2	1	20.26087	0.083922	0.052178	0.006145	0.116969
0	0	2	1	20.24578	0.068741	0.034207	0.003095	-0.25239
0	0	2	1	20.21654	0.055117	0.027525	0.005753	-0.01399

0	0	2	1	20.53084	0.301807	0.024492	0.007643	0.232113
0	0	2	1	20.57864	0.325779	0.025392	0.004524	0.101003
0	0	2	1	20.95417	0.528441	0.022577	0.005707	0.430136
0	0	2	1	21.30378	0.301164	0.052528	0.013626	-0.00218
0	0	2	1	21.40271	0.352522	0.022278	0.008503	0.030811
1	1	4	0.25	21.4926	0.396035	0.029365	0.006657	0.808623
0	0	1	1	20.51702	0.078457	0.084041	0.050344	0.177
0	0	1	1	20.7191	0.148611	0.120112	0.025733	0.57625
0	0	1	1	21.26127	0.076363	0.036694	0.034117	-0.09722
0	0	1	1	21.30313	0.130892	0.017701	0.005442	0.232497
0	0	1	1	21.76668	0.236468	0.024686	0.006526	0.583044
0	0	1	1	22.02922	0.080374	0.041328	0.008885	0.319806
0	0	1	1	22.1055	0.117225	0.042939	0.011291	0.314502
0	0	1	1	21.46105	0.136073	0.051559	0.031024	0.299
0	0	1	1	21.58021	0.19274	0.047322	0.009565	0.29921
1	1	1	1	21.64617	0.210881	0.042608	0.01074	0.197053
1	1	1	1	22.00554	0.234671	0.056277	0.016488	0.345139
1	1	1	1	22.11936	0.27365	0.063644	0.019201	0.270992
1	1	1	1	22.27294	0.393112	-0.00276	0.013151	-0.06206
1	1	1	1	22.31396	0.386058	0.045188	0.007656	0.066807
1	1	1	1	22.39308	0.406529	0.045511	0.010741	0.214903
0	0	1	1	22.36759	0.334142	0.088286	0.016084	0.39738
0	0	1	1	22.44428	0.31781	0.085065	0.019436	0.170629
0	0	1	1	21.65068	0.244541	0.128475	0.0408	0.4579
0	0	1	1	21.69913	0.191539	0.160056	0.026069	0.234426
1	3	1	1	22.06036	0.380522	0.089091	0.017893	-0.00738
1	3	1	1	22.11335	0.364417	0.110911	0.019975	0.173651
1	3	1	1	22.17966	0.366455	0.111741	0.018938	0.058168
1	3	1	1	22.22111	0.397374	0.106935	0.020891	0.025176
1	3	1	1	22.27617	0.390043	0.117028	0.022689	0.075249
1	3	1	1	22.15531	0.266104	0.134057	0.021036	0.070885
1	3	1	1	22.16995	0.241799	0.121322	0.01894	0.041231
1	3	1	1	22.23004	0.239553	0.118895	0.024415	-0.06193
1	1	1	1	20.67504	0.185156	0.05577	0.028931	0.1061
1	1	1	1	20.68141	0.176651	0.041269	0.017168	0.001629
1	1	1	1	20.73884	0.173866	0.025794	0.007792	0.005961
1	1	1	1	20.83336	0.215896	0.013809	0.003914	0.079219
1	1	1	1	20.85737	0.240753	0.004532	-0.00243	0.462756
1	1	1	1	20.87901	0.238395	0.026432	-1.1E-05	0.233064
1	1	1	1	20.94583	0.247092	0.044859	0.011911	0.066194
1	1	1	1	20.98056	0.26026	0.034103	0.008963	0.205854
1	1	1	1	20.98784	0.341581	0.027766	0.012707	0.095969
1	1	1	1	21.00697	0.35818	0.024768	0.006347	0.015006
0	0	2	0.5	21.45352	0.246024	0.04447	0.019644	0.2896
0	0	2	0.5	21.46316	0.242126	0.046996	0.014644	0.074277
0	0	2	0.5	21.55983	0.297192	0.02058	0.010973	-0.12034
0	0	2	0.5	21.58866	0.296347	0.03488	0.010366	0.101703
0	0	2	0.5	21.71904	0.384104	0.041115	0.008276	0.033653
0	0	2	0.5	21.73092	0.391259	0.049635	0.016559	0.016083
0	0	2	0.5	21.96524	0.380318	0.044178	0.020878	0.136269
0	0	2	0.5	22.19005	0.48297	0.054887	0.018026	0.205692
0	0	2	0.5	22.26318	0.518268	0.037756	0.015784	0.060614
0	0	2	0.5	22.37574	0.584272	0.032913	0.01407	0.087305
0	0	1	1	21.23555	0.146162	0.039984	0.021725	0.0596
0	0	1	1	21.57257	0.337391	0.050511	0.019992	0.677127
0	0	1	1	21.60243	0.324189	0.035608	0.012598	0.052191
0	0	1	1	21.72128	0.396747	0.019952	0.006843	0.079661
0	0	1	1	21.87506	0.476776	0.01521	0.006932	0.366994

0	0	1	1	22.0649	0.385601	0.023523	0.005701	0.023551
0	0	1	1	22.38309	0.452753	0.113428	0.032405	0.3093
0	0	1	1	22.67431	0.54544	0.060284	0.017437	0.307597
0	0	1	1	22.82234	0.507444	0.033053	0.020139	0.047358
0	0	1	1	22.9252	0.616626	-0.03391	0.01092	-0.01585
0	0	1	1	21.72428	0.125438	0.020747	0.005863	0.201459
0	0	1	1	21.64758	0.033435	0.029531	0.005925	0.117059
0	0	1	1	21.92855	0.254698	0.019435	-0.00351	0.588894
0	0	1	1	22.12513	0.199447	0.021602	-0.00491	0.281468
0	0	1	1	22.14054	0.200234	0.016934	0.000144	0.254368
1	1	1	1	22.20557	0.234713	0.036993	0.002065	0.06608
1	1	1	1	22.18937	0.18439	0.050935	0.00284	0.511566
1	1	1	1	22.89687	0.197757	0.089067	0.028807	0.2404
1	1	1	1	23.07134	0.24217	0.107146	0.018607	0.278524
1	1	1	1	23.42669	0.392927	0.091132	0.019556	0.14325
1	1	1	1	23.61068	0.441467	0.080581	0.016709	0.160741
1	1	1	1	23.7774	0.482599	0.064219	0.017107	0.174512
0	0	1	1	23.84048	0.50566	0.050569	0.014646	-0.03242
0	0	1	1	23.87642	0.510192	0.043106	0.013751	0.103384
0	0	1	1	24.05505	0.571874	0.060551	0.013667	0.334932
0	0	1	1	24.10293	0.558456	0.066923	0.021194	0.429984
0	0	1	1	24.17279	0.558333	0.058083	0.018379	0.078553
0	0	1	1	20.71602	0.020963	0.066849	0.01856	0.0891
0	0	1	1	21.05538	0.27548	0.061804	0.013903	0.329045
0	0	1	1	21.50136	0.121255	0.060818	0.016331	0.240817
0	0	1	1	21.66161	0.201189	0.065225	0.012322	0.516125
0	0	1	1	22.22586	0.159962	0.050327	0.016719	0.563538
0	0	1	1	22.49293	0.231544	0.039142	0.0101	0.931881
0	0	1	1	22.65177	0.310655	0.05109	0.009438	0.296755
0	0	1	1	22.82189	0.383593	0.0547	0.01135	0.053161
0	0	1	1	22.88372	0.386909	0.053102	0.012028	0.023575
0	0	1	1	22.89858	0.387049	0.030959	0.010401	0.210496
0	0	3	0.666667	20.77627	0.30921	0.062461	0.028226	0.1216
0	0	3	0.666667	20.81701	0.306882	0.05728	0.00761	0.333834
0	0	3	0.666667	20.8333	0.291901	0.058753	0.009403	0.048905
0	0	3	0.666667	20.92682	0.323805	0.062319	0.018885	-0.01674
0	0	3	0.666667	21.03792	0.374223	0.052993	0.012602	0.063245
0	0	3	0.666667	21.07571	0.38065	0.048522	0.00767	-0.13289
0	0	3	0.666667	22.49594	0.139709	0.062128	0.008784	0.986463
0	0	3	0.666667	22.64267	0.216252	0.058257	0.012484	0.412266
0	0	3	0.666667	22.83277	0.314008	0.062538	0.014344	0.564727
0	0	4	0	22.11069	0.076196	0.11211	0.049032	0.0724
0	0	1	0	22.35019	0.183645	0.11255	0.022817	0.32299
0	0	1	0	22.38095	0.173941	0.074476	0.019686	0.156807
0	0	1	0	22.57796	0.207015	0.178466	0.02554	0.353638
1	1	1	0	22.77686	0.222278	0.219317	0.066319	0.113244
1	1	2	0	22.75014	0.185941	0.118912	0.053148	-0.15404
1	1	2	0	22.78243	0.171056	0.101857	0.009153	-0.03734
1	1	2	0	22.91816	0.19436	0.1312	0.026244	0.39146
1	1	2	0	23.0329	0.184671	0.157239	0.042355	0.067212
1	1	2	0	23.11301	0.175679	0.151271	0.030605	0.007594
0	0	2	1	21.56385	0.269147	0.085508	0.046823	0.304
0	0	2	1	21.68595	0.304808	0.099766	0.027642	0.235265
1	1	2	1	21.84216	0.350339	0.095808	0.028815	0.136013
1	1	2	1	21.984	0.402118	0.080081	0.026825	0.125791
1	1	2	1	22.05634	0.401351	0.089101	0.021738	0.091595
0	0	2	1	22.0667	0.375887	0.072286	0.022562	-0.06699
0	0	2	1	22.24765	0.239696	0.041762	0.010351	0.014725

0	0	2	1	22.37227	0.272564	0.051652	0.021118	0.117738
0	0	2	1	22.39633	0.277313	0.051947	0.01359	0.125476
0	0	2	1	22.40318	0.263112	0.055471	0.019169	0.160193
1	1	2	0.5	21.26851	0.386002	0.070133	0.044139	0.3777
1	1	2	0.5	21.51447	0.445648	0.095007	0.014336	0.532169
1	1	2	0.5	21.68946	0.543148	0.042921	0.012605	-0.05033
1	1	2	0.5	21.86444	0.635883	0.038429	0.009076	0.208189
0	0	2	0.5	21.87414	0.640169	0.031879	0.000619	0.04005
1	1	2	0.5	21.76366	0.627509	-0.00966	0.002531	-0.2876
1	1	2	0.5	21.68407	0.575604	0.036091	-0.00183	0.061154
1	3	2	0.5	21.81231	0.618515	0.047714	0.013139	0.465985
1	1	2	0.5	21.79255	0.565398	0.071375	0.021725	0.141915
1	1	2	0.5	21.70253	0.535317	0.028391	0.005005	-0.09068
0	0	1	0	21.35194	0.347783	0.053748	0.030143	0.1071
0	0	1	0	21.44888	0.38689	0.04038	0.005506	0.23748
0	0	1	0	21.56737	0.362529	0.056873	0.010407	0.077528
0	0	1	0	21.69001	0.225093	0.053382	0.013245	0.026941
0	0	1	0	21.6938	0.217479	0.039615	0.006326	-0.13049
0	0	1	0	21.72997	0.23129	0.044121	0.006918	-0.13184
0	0	1	0	21.90484	0.351621	0.030915	0.005917	-0.00142
0	0	2	0	21.79193	0.09293	0.135623	0.133486	0.2034
0	0	2	0	21.89061	0.170876	0.114577	0.033408	0.147589
0	0	2	0	22.05294	0.215351	0.09396	0.019743	0.067379
0	0	2	0	22.2521	0.216531	0.125303	0.028268	0.147711
0	0	2	0	22.41466	0.272365	0.119444	0.022469	0.082974
0	0	2	0	22.4327	0.255056	0.101861	0.018523	0.108308
0	0	2	0	22.68234	0.190723	0.100777	0.021738	0.134374
0	0	2	0	22.79288	0.230729	0.104321	0.020232	0.187962
0	0	2	0	22.95128	0.265044	0.079391	0.029453	0.386408
0	0	2	0	23.13313	0.315838	0.090375	0.024531	0.116399
1	1	2	0.5	20.56459	0.164031	0.085487	0.14754	0.2769
0	0	2	0.5	21.01611	0.407922	0.092709	-0.00476	0.065146
0	0	2	0.5	21.29996	0.46911	0.107322	0.001689	0.78615
0	0	2	0.5	21.59289	0.542534	0.091275	0.003853	0.431391
0	0	2	0.5	21.89063	0.344787	0.091721	0.00682	0.013753
0	0	2	0.5	21.90851	0.27541	0.095557	0.006788	0.072654
1	1	2	0.5	21.88485	0.138831	0.124067	0.003397	0.349361
1	1	2	0.5	21.73408	0.243818	0.136725	0.007177	-0.49863
1	1	1	1	20.85879	0.222815	0.083422	0.073001	0.4385
1	1	1	1	21.36468	0.445879	0.106309	0.020221	1.911137
1	1	1	1	22.47956	0.469912	0.095681	0.036242	0.464346
1	1	1	1	22.79804	0.523021	0.109644	0.029673	0.428743
1	1	1	1	23.10987	0.556232	0.133319	0.036194	0.631249
1	1	1	1	23.22697	0.724385	-0.0724	0.037864	-0.54344
1	1	1	1	23.53847	0.638248	-0.06646	0.029234	0.206724
1	1	1	1	23.70925	0.821621	-0.06985	0.026539	-0.10788
0	0	1	1	20.86118	0.146473	0.044873	0.039854	-0.2266
0	0	1	1	20.96988	0.203732	0.035874	0.002083	0.148574
0	0	1	1	20.95965	0.170484	0.040759	0.005383	0.174758
0	0	1	1	20.99465	0.164834	0.06242	0.011757	0.208797
0	0	1	1	21.06004	0.157738	0.068888	0.014318	-0.03612
0	0	1	1	21.32353	0.323951	0.03975	0.013672	0.387144
0	0	1	1	21.76963	0.534003	0.051542	0.015664	0.948564
0	0	1	1	21.78383	0.517493	0.038068	0.009128	0.094913
0	0	1	1	21.61413	0.53271	-0.09807	-0.0055	-0.25598
0	0	1	1	21.80429	0.569637	0.060955	0.002996	0.100447
0	0	2	0.5	20.4703	0.074594	0.071528	0.016262	0.4572
0	0	2	0.5	20.53687	0.108775	0.071914	0.018934	0.320403

0	0	2	0.5	20.77005	0.226444	0.076617	0.010487	0.322476
0	0	2	0.5	21.30877	0.253436	0.050665	0.019323	0.092525
0	0	2	0.5	21.39338	0.28994	0.055265	0.015163	0.266971
0	0	1	1	21.65052	0.254937	0.066713	0.016669	0.557166
0	0	1	1	22.06056	0.346108	0.144863	0.045104	1.100649
0	0	3	0.666667	23.32749	0.410004	0.108612	0.052992	0.141541
0	0	3	0.666667	23.37743	0.40827	0.038331	0.024962	0.067515
0	0	1	1	22.11221	0.244456	0.095579	0.067467	0.3112
0	0	1	1	22.16722	0.240176	0.086205	0.0173	0.047358
0	0	1	1	22.26772	0.267282	0.068495	0.01509	0.002333
0	0	1	1	22.29337	0.296767	0.044231	0.013234	-0.04036
0	0	2	1	22.51374	0.417886	0.053085	0.011877	0.220685
0	0	2	1	22.61004	0.392345	0.102846	0.014922	0.188068
0	0	2	1	22.83181	0.376648	0.164105	0.028349	0.296833
1	3	2	0.5	20.49439	0.071874	0.098156	0.08501	0.2875
1	3	2	0.5	20.4974	0.089888	0.06211	0.017125	-0.05803
1	3	2	0.5	20.53064	0.088318	0.053041	0.015159	0.172384
1	3	2	0.5	20.7266	0.102243	0.046662	0.015356	0.183167
1	1	1	0	20.77169	0.091271	0.046047	0.035308	-0.0356
0	0	1	0	20.83904	0.123362	0.037697	0.004946	0.368613
1	1	1	0	21.1735	0.355336	0.032163	0.007061	-0.01466
1	1	1	0	21.2415	0.484542	-0.06657	0.00548	0.045729
1	1	2	0	22.53622	0.225841	0.056617	-0.00268	2.42763
1	1	2	0	22.74034	0.458231	0.081484	0.0147	0.312498
1	1	2	0	23.13971	0.482906	0.201161	0.061951	1.091496
1	1	2	0	23.6047	0.403864	0.196577	0.055885	0.400935
1	1	2	0	24.52176	0.732553	0.091962	0.061204	0.141568
1	1	2	0	24.5648	0.808797	-0.05262	0.019937	-0.22481
1	3	1	1	22.93843	0.687385	0.042428	0.008008	0.192656
0	0	3	0	21.12629	0.179976	0.12083	0.091726	0.5921
0	0	3	0	21.28483	0.230447	0.109403	0.0287	0.101617
0	0	3	0	21.32326	0.233015	0.077535	0.020881	-0.14395
0	0	3	0	21.50165	0.335092	0.046565	0.016028	0.246342
0	0	3	0	21.59821	0.352501	0.070993	0.016235	0.278034
0	0	3	0	22.05986	0.250539	0.046724	0.015402	0.103368
0	0	3	0	22.08555	0.228187	0.053225	0.012662	0.247183
0	0	3	0	22.73646	0.544335	0.041984	0.014205	0.514308
0	0	3	0	22.79313	0.535288	0.040614	0.012548	0.193924
0	0	3	0	22.82247	0.548814	0.027484	0.009453	0.027017
1	1	1	0	19.98013	0.082331	0.083073	0.060591	0.5054
0	0	1	0	19.96669	0.063585	0.038996	0.010339	-0.02419
0	0	1	0	20.03413	0.112173	0.040405	0.007682	-0.10121
1	1	1	0	19.95908	0.070916	-0.00227	0.001063	-0.19012
1	1	1	0	19.90939	0.111356	-0.03103	-6.2E-05	-0.09125
1	1	3	0.666667	21.39933	0.071893	0.084891	0.056515	0.2933
1	3	3	0.666667	21.46716	0.093186	0.093334	0.018812	0.281477
1	3	3	0.666667	21.77458	0.254888	0.111235	0.027063	1.321689
1	3	3	0.666667	21.9992	0.357757	0.072298	0.025456	0.170588
1	3	3	0.666667	22.06293	0.352415	0.08049	0.01955	0.177512
1	3	3	0.666667	22.33027	0.371644	0.068708	0.014312	-0.22875
1	3	3	0.666667	22.36228	0.376705	0.08425	0.016595	-0.06172
1	3	3	0.666667	22.35258	0.374684	0.108507	0.024346	0.231297
1	3	3	0.666667	22.48892	0.313915	0.051452	0.015347	-0.09424
1	3	3	0.666667	22.93188	0.491554	0.057042	0.012119	0.367162
1	1	3	1	21.99288	0.293992	0.076304	0.061481	1.1886
1	1	3	1	22.40046	0.483481	0.063911	0.017892	0.288761
1	1	2	1	22.68855	0.557169	0.065251	0.009543	0.252722
1	1	2	1	22.90217	0.575593	0.079551	0.012566	0.282318

1	3	2	1	23.15047	0.621862	0.069804	0.012997	0.126177
1	3	2	1	23.33748	0.565035	0.038829	0.01135	-0.18165
1	3	2	1	23.48614	0.602219	0.038308	0.006391	0.262487
1	3	2	1	23.48734	0.566398	0.05713	0.005804	0.239573
1	3	2	1	23.7662	0.659778	0.037056	0.006952	0.148574
1	2	2	1	23.91966	0.711313	0.017922	0.007638	-0.09386
1	3	1	1	21.96971	0.114889	0.046032	0.01078	0.121261
1	3	1	1	22.0708	0.160756	0.065933	0.011118	0.362198
1	3	1	1	22.1237	0.152603	0.066876	0.012996	0.176317
1	3	1	1	22.22756	0.200866	0.066503	0.013104	0.082794
0	0	1	1	21.02201	0.351893	0.069197	0.066376	0.5533
0	0	1	1	21.20539	0.418976	0.065327	0.013708	0.312966
0	0	1	1	21.41134	0.524799	0.049563	0.013194	0.156225
0	0	1	1	21.66194	0.624352	0.039806	0.011438	0.300456
0	0	1	1	21.98363	0.541811	0.04013	0.008045	0.125981
0	0	1	1	22.03653	0.564477	0.024357	0.008349	0.160392
0	0	1	1	22.16821	0.661655	-0.0348	-0.00224	0.490977
0	0	1	1	22.69554	0.42267	0.008773	-0.00319	0.321002
0	0	1	1	22.7082	0.447862	0.036831	0.00647	-0.09746
0	0	1	1	22.62639	0.403268	0.01442	0.006258	-0.21966
0	0	3	1	22.14647	0.262695	0.079626	0.137096	0.3996
0	0	3	1	22.0954	0.218245	0.068481	0.038453	0.190433
0	0	3	1	22.18283	0.291651	0.044113	0.043392	-0.03685
0	0	3	1	22.31235	0.357277	0.059959	0.043453	0.095411
0	0	3	1	22.45278	0.417393	0.072337	0.022957	0.091776
0	0	3	1	22.48294	0.417818	0.103845	0.049372	0.072998
0	0	3	1	22.59929	0.482801	0.017547	0.042837	0.071095
0	0	3	1	22.8554	0.605005	0.040257	0.041526	0.196182
0	0	3	1	22.63706	0.521737	-0.00494	0.024456	-0.03312
0	0	3	1	22.64734	0.446583	0.092313	0.053554	0.068193
0	0	2	1	22.24636	0.401885	0.080417	0.080417	0.3027
0	0	2	1	22.26847	0.391551	0.045792	0.007006	0.012865
0	0	2	1	22.38828	0.441274	0.038401	0.006209	0.07791
0	0	2	1	22.48	0.527723	-0.02678	0.006369	-0.23036
0	0	2	1	22.52241	0.545	0.012051	0.003165	0.110064
0	0	2	1	22.48991	0.566227	-0.04261	-0.00164	-0.36009
0	0	2	1	22.47629	0.67331	-0.09476	-0.00818	-0.0513
1	3	2	1	22.61148	0.695172	0.040147	0.00899	0.501413
1	3	2	1	22.46998	0.688104	-0.01201	0.007641	-0.01418
1	1	2	0.5	20.79836	0.289367	0.076259	0.082589	0.0809
1	1	2	0.5	20.70983	0.223722	0.081237	0.016467	-0.07252
1	1	2	0.5	20.7289	0.186577	0.086564	0.02218	0.112652
1	1	2	0.5	20.81356	0.224256	0.060076	0.018367	-0.06478
0	0	2	0.5	20.93187	0.274417	0.0485	0.011861	0.045074
0	0	2	0.5	21.13906	0.251846	0.032629	0.005859	-0.12313
0	0	2	0.5	21.15335	0.23655	0.037306	0.012853	0.367463
0	0	2	0.5	21.17624	0.22346	0.050614	0.011798	0.082835
0	0	2	0.5	21.16688	0.194797	0.04969	0.009583	0.123934
0	0	2	0.5	21.21256	0.209473	0.048679	0.013179	0.079644
1	1	1	0	23.26217	0.428561	0.215157	0.10264	0.5529
1	1	1	0	23.58891	0.497553	0.144286	0.074467	0.511393
1	1	1	0	23.88951	0.622378	0.022697	0.039676	-0.00069
1	1	1	0	24.06853	0.667875	0.018527	0.002934	0.230495
1	1	1	0	24.1465	0.716301	-0.00545	-0.00187	0.083621
1	1	1	0	24.3469	0.656219	0.028786	0.003484	-0.09862
1	1	1	0	24.42805	0.636332	0.086003	0.007458	0.586856
1	1	1	0	24.85127	0.585103	0.044206	0.020497	0.550103
1	1	1	0	25.52248	0.705318	0.029887	0.010118	0.296227

1	1	1	0	25.93049	0.77234	0.022384	0.005942	-0.09762
0	0	1	1	20.74752	0.056007	0.061471	0.009594	0.137887
0	0	1	1	20.80723	0.056334	0.067455	0.007438	0.102962
0	0	1	1	21.07351	0.263956	0.069345	0.013876	0.231253
0	0	1	1	21.48422	0.340398	0.063371	0.010272	0.305562
0	0	1	1	21.55611	0.383494	0.014924	0.009872	-0.32016
0	0	1	1	21.64977	0.208337	0.012764	0.007483	-0.11503
0	0	1	1	21.70408	0.280791	-0.01777	0.007093	0.263622
0	0	1	1	21.66436	0.246708	0.003108	0.004916	-0.20914
0	0	1	1	21.65438	0.255568	0.007667	0.003983	-0.04825
0	0	3	1	20.77869	0.034865	0.061915	0.061915	0.1551
0	0	3	1	20.81172	0.031868	0.048485	0.010782	0.052689
0	0	3	1	20.81791	0.029785	0.035301	0.009394	-0.03759
0	0	3	1	20.82876	0.036613	0.028364	0.011786	0.005298
0	0	3	1	20.82726	0.027656	0.038468	0.011769	0.111133
0	0	3	1	20.88322	0.072293	0.037527	0.012409	0.088854
0	0	3	1	20.88365	0.050893	0.03039	0.010701	-0.10692
0	0	3	1	20.92606	0.072823	0.017892	0.00536	-0.03759
0	0	3	1	20.97632	0.09821	0.031311	0.009984	0.111241
0	0	3	1	20.96116	0.062371	0.024684	0.007799	-0.07012
0	0	3	0	21.34535	0.136311	0.065527	0.063914	0.1492
0	0	3	0	21.58731	0.273022	0.028871	0.005711	0.322424
0	0	3	0	21.84359	0.369855	0.052976	0.010878	0.590893
0	0	3	0	22.06134	0.469365	0.05719	0.016591	0.251139
0	0	3	0	22.25443	0.506842	0.063283	0.019042	0.164549
0	0	3	0	22.54334	0.425068	0.042348	0.012608	0.372158
1	1	3	0	22.73269	0.493807	0.033351	0.011806	0.752248
0	0	3	0	22.81759	0.482456	0.074216	0.024536	-0.32341
1	1	3	0	22.41241	0.457989	-0.09052	0.004388	-0.51317
0	0	1	0	21.32309	0.075828	0.10008	0.10008	0.1947
0	0	1	0	21.43713	0.073675	0.110782	0.08272	0.448826
0	0	1	0	21.65335	0.183704	0.096333	0.014416	-0.00559
1	1	1	0	21.75224	0.197639	0.102794	0.017992	0.168186
1	1	1	0	21.79174	0.187932	0.107244	0.017894	0.132793
1	1	1	0	21.78567	0.172517	0.063019	0.010191	-0.07665
1	1	1	0	21.89888	0.200868	0.063059	0.011068	0.175742
1	1	1	0	22.19172	0.348269	0.086479	0.015342	0.493255
1	1	1	0	22.2794	0.36916	0.077277	0.016424	0.300256
1	1	1	0	22.37538	0.394891	0.042228	0.012769	0.042421
1	3	1	1	22.13323	0.191923	0.115894	0.117516	0.0905
1	3	1	1	22.21748	0.213939	0.073564	0.02315	0.214906
1	3	1	1	22.27775	0.190109	0.056813	0.010565	-0.02376
1	3	1	1	22.34103	0.181692	0.105264	0.00804	0.308276
1	3	1	1	22.49676	0.283909	0.05467	0.006898	-0.03533
1	3	1	1	22.55021	0.334025	0.069364	0.009625	-0.10059
1	3	1	1	22.58532	0.310281	0.076719	0.010554	-0.0079
1	3	1	1	22.55179	0.255354	0.047944	0.013773	0.146647
1	3	1	1	22.61689	0.292313	0.042069	0.007867	0.173487
1	3	1	1	22.57315	0.205287	0.077895	0.011753	0.109807
0	0	2	1	21.30382	0.362499	0.080031	0.059506	0.3077
0	0	2	1	21.52568	0.438415	0.093865	0.021549	0.204095
0	0	2	1	22.00419	0.604421	0.093668	0.027065	0.233175
0	0	2	1	22.24428	0.324326	0.098934	0.024831	0.233165
0	0	2	1	22.62557	0.487214	0.089918	0.023234	0.028981
0	0	2	1	22.90027	0.564912	0.085725	0.021351	0.188554
0	0	2	1	23.21561	0.622357	0.075778	0.018793	0.113741
0	0	2	1	23.44625	0.474955	0.05708	0.012712	0.185037
0	0	1	0	22.21989	0.313118	0.073589	0.073589	1.0381

0	0	1	0	22.6233	0.564053	0.029698	0.011884	0.240423
0	0	1	1	21.24467	0.186028	0.090878	0.090878	0.3184
0	0	1	1	21.37528	0.213274	0.100964	0.021544	0.245446
0	0	1	1	21.57011	0.253579	0.119061	0.023433	0.279586
0	0	1	1	21.75388	0.271544	0.145593	0.026258	0.352094
0	0	1	1	22.01135	0.315632	0.171236	0.031929	0.352373
0	0	1	1	22.33802	0.370099	0.179613	0.035364	0.265755
0	0	1	1	22.58194	0.356958	0.206638	0.035257	0.275646
0	0	1	1	22.79349	0.336732	0.201952	0.04086	0.210961
0	0	1	1	22.96985	0.351601	0.16932	0.0408	0.058068
0	0	1	1	23.08901	0.345256	0.167868	0.038101	0.045212
0	0	2	1	21.89905	0.383824	0.053539	0.029402	0.099
1	1	2	1	21.76035	0.263549	0.041307	0.010642	0.043506
1	1	2	1	21.95459	0.356181	0.070242	0.015086	0.260307
1	1	2	1	22.22576	0.492011	0.042395	0.01528	0.06936
1	1	2	1	22.23839	0.483555	0.036659	0.00999	0.008019
0	0	2	1	22.23713	0.464388	0.048898	0.012678	0.173368
0	0	2	1	22.23011	0.402458	0.087315	0.017346	0.287387
0	0	2	1	22.47972	0.474443	0.080815	0.023895	0.217623
0	0	2	1	22.36147	0.356529	0.11005	0.027488	0.224254
0	0	2	1	22.51939	0.323462	0.123206	0.029815	0.168424
0	0	1	0	21.24031	0.124766	0.090983	0.090983	0.3244
0	0	1	0	21.33288	0.140293	0.078821	0.023045	0.307552
0	0	1	0	21.38688	0.143157	0.080802	0.016084	0.152556
0	0	1	0	21.77033	0.371557	0.067819	0.015899	0.209421
0	0	1	0	21.96166	0.447457	0.072016	0.017872	0.223623
0	0	1	0	22.60207	0.665797	0.05746	0.013386	0.06692
0	0	1	0	22.66666	0.471674	0.072329	0.013401	0.237148
1	1	1	0	22.31738	0.165389	0.117153	0.018083	0.006739
1	1	1	0	22.19076	0.118335	0.065222	0.018955	-0.23759
1	1	1	0	22.14358	0.128115	0.049074	0.012842	-0.10904
0	0	1	1	20.53505	0.275612	-0.05271	-0.01011	-0.03871
1	3	1	1	20.60804	0.190954	0.067606	0.067309	0.309
1	3	1	1	20.66977	0.203495	0.120169	0.038982	0.65958
1	3	1	1	20.6748	0.226784	0.070415	0.033914	-0.26569
1	3	1	1	20.68728	0.34822	0.011138	0.00919	-0.23545
1	3	1	1	21.02397	0.482261	0.052	0.018096	1.229522
1	3	1	1	21.6118	0.295359	0.024687	0.014008	0.39185
1	3	1	1	21.57825	0.265839	0.028034	0.009675	0.020829
1	3	1	1	21.69104	0.318323	0.032674	0.00666	0.282705
1	1	1	1	22.47085	0.486632	0.050386	0.01062	0.958354
1	1	1	1	22.48512	0.509299	0.052934	0.014604	0.097097
0	0	2	1	21.6632	0.230479	0.069248	0.069248	0.3701
0	0	2	1	21.66335	0.205802	0.030117	0.004355	0.166286
0	0	2	1	21.78698	0.269767	0.058903	0.001277	0.12145
0	0	2	1	21.9733	0.346976	0.068095	0.014153	0.191039
0	0	2	1	22.11643	0.362879	0.087211	0.023058	0.203895
0	0	2	1	22.13362	0.182096	0.079538	0.02315	-0.07185
0	0	2	1	22.16392	0.193883	0.041424	0.006158	0.149689
0	0	2	1	22.21854	0.234016	0.046865	0.012501	0.31943
0	0	2	1	22.19565	0.225591	0.017197	0.006793	0.029603
0	0	2	1	22.1916	0.197801	0.04058	0.001021	-0.11668
0	0	2	0.5	20.82137	0.099563	0.081943	0.081943	0.2194
0	0	2	0.5	20.93504	0.130076	0.084663	0.02106	0.15736
0	0	2	0.5	21.00169	0.144721	0.081284	0.020474	-0.01241
0	0	2	0.5	20.98548	0.179756	0.062171	0.015673	0.115788
0	0	2	0.5	21.27894	0.218017	0.042544	0.006324	0.069676
0	0	2	0.5	21.32766	0.244103	0.046089	0.006034	-0.10684

0	0	2	0.5	21.45814	0.237475	0.055775	0.012983	0.113755
0	0	2	0.5	21.79974	0.289623	0.019245	0.007743	0.178719
0	0	2	0.5	21.85743	0.362906	0.009262	0.006483	0.6514
0	0	2	0.5	21.91699	0.412237	0.018448	0.00568	0.187866
0	0	1	1	21.769	0.350377	0.06612	0.06612	0.2472
1	1	1	1	21.77326	0.321535	0.067538	0.013362	0.261242
1	1	1	1	21.83149	0.347914	0.02833	0.011941	-0.02885
1	1	1	1	21.95138	0.40265	0.027193	0.003734	-0.09839
1	1	1	1	22.05046	0.453896	0.022674	0.005027	-0.00072
1	1	1	1	21.80053	0.397984	-0.07229	0.00544	-0.25989
1	1	1	1	21.70294	0.333086	0.013375	0.00195	-0.2039
1	1	1	1	21.84191	0.23695	0.037851	-0.00255	0.61355
1	1	1	1	21.87848	0.214281	0.069411	0.016735	0.356046
1	1	1	1	21.947	0.212468	0.078985	0.018653	0.161996
1	1	2	0	21.00815	0.119728	0.073323	0.065282	0.2315
1	1	2	0	21.02454	0.066409	0.085686	0.005932	0.30944
1	1	2	0	21.21038	0.126062	0.088176	0.006237	0.278695
1	1	2	0	21.3003	0.127453	0.106005	0.008877	0.190474
0	0	2	0	21.62939	0.175205	0.089042	0.008806	0.304336
0	0	2	0	21.95088	0.343794	0.062288	0.007304	0.154748
1	1	2	0	22.21348	0.387199	0.054962	0.005661	0.809229
1	1	2	0	22.55775	0.524584	0.04069	0.01001	0.248314
0	0	2	0	22.61592	0.512042	-0.02375	0.004142	0.032725
0	0	2	0	22.57289	0.49186	0.028859	0.005206	0.005398
0	0	3	0.333333	21.04596	0.261375	0.059439	0.059439	0.3207
0	0	3	0.333333	20.87323	0.126422	0.041698	0.003485	0.079606
0	0	3	0.333333	20.80361	0.083725	0.002442	-0.00051	-0.33415
0	0	3	0.333333	20.84576	0.117471	0.005972	-9.9E-05	0.009732
0	0	3	0.333333	20.84985	0.122922	0.001845	-0.00698	-0.18153
0	0	3	0.333333	20.92268	0.181649	0.004932	-0.0075	0.113403
1	1	3	0.333333	21.36405	0.177871	-0.02353	-0.00382	-0.10832
1	1	3	0.333333	21.36453	0.174439	0.008431	0.007116	0.326641
1	1	3	0.333333	21.37639	0.274965	-0.0983	0.016138	-0.11634
0	0	2	1	21.55662	0.401952	0.05351	0.018725	0.149576
0	0	2	1	21.4083	0.266842	0.083137	0.030559	0.143542
0	0	2	1	21.40645	0.281878	0.079385	0.017141	-0.00554
0	0	2	1	21.41094	0.270566	0.084543	0.015753	0.103076
1	1	1	1	20.93475	0.636621	0.13307	0.131871	0.2414
1	1	1	1	21.76518	0.348491	0.080745	0.017449	0.297438
1	1	1	1	22.23748	0.516646	0.069051	0.016421	0.162308
1	1	1	1	22.41885	0.565151	0.066788	0.017987	0.578596
0	0	1	1	22.64687	0.657277	0.024767	0.016586	-0.07379
0	0	1	1	22.65984	0.570079	-0.03796	0.007968	-0.34227
0	0	1	1	22.61529	0.553429	0.014961	0.004245	0.041433
0	0	2	1	22.76587	0.620148	0.016507	0.004259	0.286625
0	0	1	1	22.74074	0.625386	0.004296	0.008315	0.24862
0	0	1	1	20.86042	0.710517	0.124834	0.149746	0.4872
0	0	1	1	21.24663	0.323084	0.056333	0.017961	-0.05558
0	0	1	1	21.40025	0.405708	0.028714	0.009835	-0.07884
0	0	1	1	21.51215	0.463906	0.026693	0.010373	0.430995
0	0	1	1	21.64765	0.523135	0.033158	0.009367	0.297974
0	0	1	1	21.81705	0.406436	0.030604	0.012083	0.185893
0	0	1	1	21.77698	0.354532	0.042481	0.012702	0.088771
0	0	1	1	21.87889	0.351052	0.090765	0.020576	0.278763
0	0	1	1	22.03796	0.40802	0.076066	0.027673	0.057978
0	0	1	1	22.08625	0.459798	0.02083	0.011652	-0.06464
0	0	2	1	20.69979	0.70539	0.140681	0.111215	0.53
0	0	2	1	21.27843	0.249298	0.06183	0.015254	-0.02617

0	0	2	1	21.65645	0.413404	0.060736	0.012919	0.185625
0	0	2	1	21.85744	0.486502	0.052945	0.014248	0.290428
0	0	2	1	21.71189	0.387566	0.045719	0.014114	-0.19701
0	0	2	1	21.74094	0.377158	0.054176	0.038666	-0.17597
0	0	2	1	23.37971	0.526422	0.021996	0.022875	0.275639
0	0	2	1	23.66564	0.630634	0.025786	0.009497	0.516932
0	0	2	1	22.96754	0.297865	0.01587	0.008929	0.254049
0	0	2	0.5	20.57852	0.587178	0.14206	0.14206	0.0958
0	0	2	0.5	21.4245	0.291154	0.090811	0.025727	0.327755
0	0	2	0.5	21.50549	0.281769	0.101623	0.031505	0.078624
0	0	2	0.5	21.70761	0.382567	0.06067	0.023501	0.19886
0	0	2	0.5	21.94097	0.4772	0.063499	0.019129	0.069473
0	0	2	0.5	22.13564	0.26641	0.076853	0.021117	0.25038
0	0	1	1	22.21431	0.474515	0.081703	0.023171	0.583363
0	0	1	1	22.34075	0.52942	0.054767	0.018524	0.056434
0	0	1	1	22.44028	0.569188	0.049816	0.018312	0.123794
0	0	1	1	22.62172	0.62507	0.057255	0.018666	0.212623
0	0	1	1	22.90546	0.651579	0.054562	0.018442	0.250841
0	0	1	1	22.92298	0.649958	0.042867	0.013968	0.040144
0	0	1	1	22.95331	0.660054	0.034504	0.017303	0.274943
0	0	3	0.666667	23.06239	0.693182	0.044511	0.014744	0.016372
0	0	3	0.666667	22.99723	0.660586	0.046943	0.010013	0.096358
1	1	3	0.666667	21.73814	0.20742	0.091451	0.019515	0.146829
1	1	3	0.666667	21.86314	0.249152	0.0789	0.01483	0.157878
1	1	3	0.666667	21.9924	0.287438	0.085842	0.025749	0.212659
1	1	3	0.666667	22.04667	0.313009	0.078743	0.028007	0.077248
1	2	3	0.666667	22.17159	0.337139	0.080017	0.03066	0.053926
1	2	3	0.666667	22.35063	0.425766	0.095247	0.029501	0.183028
1	2	3	0.666667	22.59661	0.512199	0.082568	0.0283	0.317055
1	2	3	0.666667	22.64007	0.493057	0.094618	0.031875	0.058472
1	2	3	0.666667	22.66391	0.438461	0.108189	0.034182	-0.1004
1	1	1	1	21.34017	0.046985	0.084451	0.025645	-0.35563
1	1	1	1	21.37942	0.030375	0.072564	0.013135	-0.01228
1	1	1	1	21.44902	0.046347	0.07305	0.018539	0.181431
1	1	1	1	21.56083	0.077059	0.087395	0.017978	-0.05364
1	1	1	1	21.76465	0.190132	0.071412	0.020298	-0.07066
1	1	1	1	21.83311	0.200844	0.052615	0.018125	0.025544
0	0	1	1	21.83654	0.194266	0.040923	0.019646	0.372335
0	0	1	1	21.87962	0.2014	0.04192	0.027025	0.24047
0	0	1	1	21.79973	0.205041	0.106559	0.025968	0.267444
0	0	2	1	21.00724	0.111258	0.025659	0.011479	0.290304
0	0	1	1	21.14423	0.199405	0.038679	0.012164	0.2092
0	0	1	1	21.42595	0.338865	0.039497	0.012333	0.734793
0	0	1	1	21.58043	0.381768	0.053558	0.013163	0.596355
0	0	1	1	21.80789	0.465433	0.059977	0.018212	0.428138
0	0	1	1	22.124	0.465051	0.044751	0.019223	0.167873
0	0	1	1	22.1843	0.379824	0.053852	0.017081	0.031051
0	0	1	1	22.13537	0.323289	0.037345	0.017366	-0.18742
0	0	1	1	22.27508	0.403373	0.014006	0.008829	-0.0611
0	0	2	0	20.40511	0.089247	0.076482	0.020403	0.094979
0	0	2	0	20.16269	0.072138	0.076633	0.018951	0.065518
0	0	2	0	20.21744	0.070718	0.062857	0.016017	0.025577
0	0	2	0	20.2906	0.076937	0.077404	0.018085	0.135643
0	0	2	0	20.32917	0.073132	0.108271	0.03227	0.125285
0	0	2	0	20.38045	0.076598	0.095485	0.028384	0.002665
1	1	2	0	20.43937	0.079651	0.10714	0.025388	0.059173
1	1	2	0	20.47763	0.102736	0.119956	0.035057	0.216572
1	1	2	0	20.50387	0.100356	0.131328	0.033684	0.009543

1	1	2	0	20.48236	0.126326	0.064949	0.020099	0.036576
0	0	2	0.5	20.5494	0.089482	0.050597	0.022982	0.0675
1	1	2	0.5	20.52559	0.056762	0.013	0.002939	-0.08613
0	0	2	0.5	20.5015	0.079346	-0.00072	-0.00037	-0.16797
0	0	2	0.5	21.95153	0.168127	0.015163	-0.00086	1.326676
1	1	2	0.5	22.25438	0.197345	0.19881	0.046987	6.784336
1	1	2	0.5	22.57741	0.259475	0.205648	0.055549	0.126934
1	1	2	0.5	22.93813	0.204525	0.212352	0.077328	0.179298
1	3	2	0.5	22.85099	0.271811	0.147267	0.053511	0.2333
0	0	1	0	21.86031	0.187976	0.077269	0.018147	0.2776
0	0	1	0	21.91583	0.196391	0.098358	0.020662	0.004019
0	0	1	0	22.07689	0.302872	0.077558	0.006352	0.086769
0	0	1	0	22.17737	0.34131	0.085411	0.018852	0.041755
0	0	1	0	22.24514	0.360102	0.101965	0.023942	0.063645
0	0	1	0	22.16313	0.281663	0.108588	0.02939	0.060893
0	0	1	0	22.26525	0.32996	0.091363	0.025775	0.025541
0	0	1	0	22.35801	0.344943	0.11056	0.02449	0.16496
0	0	1	0	22.41128	0.324238	0.1387	0.028521	0.152537
1	1	1	0	22.8846	0.123007	0.132187	-0.02864	4.069967
1	1	1	0	23.27563	0.290235	0.094407	0.038896	0.250964
1	1	1	0	23.09182	0.149575	0.103466	0.030163	0.30029
1	1	1	0	23.01985	0.188672	0.082279	0.030127	-0.31965
1	1	2	0.5	23.53391	0.290378	0.120725	0.023188	0.230142
0	0	1	1	21.71022	0.783194	0.069889	0.069889	0.1407
0	0	1	1	22.14146	0.50426	0.050829	0.008352	0.400352
0	0	1	1	22.37528	0.586366	0.042989	0.009878	0.216227
0	0	1	1	22.51247	0.525197	0.043638	0.010931	0.156577
0	0	1	1	22.65307	0.57238	0.031104	0.010963	0.12432
0	0	1	1	22.65192	0.575148	0.029034	0.008623	-0.15557
0	0	1	1	23.55676	0.83648	-0.00943	0.004353	-0.39251
1	1	2	1	23.84063	0.860102	0.03161	0.008093	7.704953
0	0	2	1	21.84074	0.299147	-0.06458	0.0173	-0.15597
0	0	1	1	21.44955	0.25805	0.076332	0.011244	0.3019
0	0	1	1	21.59063	0.309375	0.087979	0.017778	0.178413
0	0	1	1	21.70698	0.338231	0.067163	0.012918	0.059764
0	0	1	1	22.02073	0.457122	0.06718	0.011553	0.414091
0	0	1	1	22.11732	0.322432	0.062578	0.013017	-0.06523
0	0	1	1	22.27268	0.370483	0.064278	0.013187	0.155988
0	0	1	1	22.52015	0.349967	0.079544	0.014384	0.261153
0	0	1	1	22.5998	0.394781	0.044487	0.013401	0.122646
0	0	1	1	22.70852	0.478078	0.04874	0.005027	-0.00407
0	0	2	1	20.57795	0.32678	0.083726	0.020163	0.3298
0	0	2	1	20.60122	0.307434	0.069952	0.013926	-0.08563
0	0	2	1	20.61814	0.321892	0.025086	0.013915	-0.00343
0	0	2	1	20.5173	0.376597	-0.09385	0.007793	-0.25977
1	3	1	1	20.25691	0.162644	0.031763	0.003665	-0.08153
1	2	1	0	23.09628	0.551032	0.082363	0.018293	-0.00159
0	0	3	0.333333	22.0263	0.240245	0.102999	0.051588	0.4615
0	0	3	0.333333	22.1857	0.349003	0.037586	0.011299	0.130382
0	0	3	0.333333	22.30503	0.410074	0.038689	0.013126	0.285727
0	0	3	0.333333	22.33318	0.390445	0.060946	0.023411	-0.20048
0	0	3	0.333333	22.32283	0.383126	0.032889	0.017508	-0.23421
1	1	3	0.333333	22.31688	0.375877	0.026398	0.011323	-0.36057
1	1	3	0.333333	22.07975	0.191813	0.039473	0.013502	0.10032
0	0	1	1	22.12621	0.212806	0.034822	0.010893	0.110419
0	0	1	1	22.20329	0.2614	0.028981	0.014131	-0.16222
1	1	1	1	20.77125	0.168168	0.067245	0.038885	0.1153
1	1	1	1	20.74873	0.153369	0.014513	0.008549	-0.20814

1	1	1	1	20.87956	0.250512	0.008077	-0.00078	0.222834
1	1	1	1	20.85252	0.249911	-0.01	0.001131	0.146197
1	1	1	1	20.8276	0.201404	0.015552	0.004353	-0.18449
1	1	1	1	20.70511	0.129744	-0.00169	-0.0017	-0.04582
1	1	1	1	20.77364	0.190055	0.001997	0.017355	0.324625
1	1	1	1	20.84045	0.230247	0.01812	0.004867	0.065042
1	1	1	1	21.0021	0.327915	0.030443	0.007525	0.119694
0	0	2	1	20.75796	0.121695	0.064035	0.028452	0.1948
0	0	2	1	20.8667	0.20621	0.035381	0.008402	0.450884
0	0	2	1	20.92196	0.21155	0.039737	0.008843	0.120313
0	0	2	1	20.99161	0.237716	0.031104	0.00803	0.13894
0	0	2	1	20.96687	0.229672	0.01433	0.007853	-0.13155
0	0	2	1	21.25043	0.141215	0.010398	0.004669	-0.0887
0	0	2	1	21.25396	0.123543	0.02316	0.004732	0.178901
0	0	2	1	21.25879	0.11725	0.020079	0.00376	0.16072
0	0	2	1	21.25031	0.182398	0.029006	0.003341	0.026077
0	0	2	1	20.84929	0.116067	0.085785	0.043129	0.1004
0	0	2	1	21.05529	0.252654	0.051899	0.012588	0.205842
0	0	2	1	21.17946	0.347204	0.038556	0.00801	0.450031
0	0	2	1	21.23088	0.315011	0.066694	0.010826	-0.01873
0	0	2	1	21.59512	0.24828	0.055611	0.011632	0.143586
0	0	2	1	21.58723	0.236896	0.046035	0.008768	0.165048
0	0	2	1	21.63272	0.30631	0.043303	0.002537	0.159339
0	0	2	1	21.97866	0.482178	0.03539	0.004735	-0.01176
0	0	2	1	22.16569	0.527406	0.027606	-0.00209	-0.00519
0	0	1	1	22.14828	0.108455	0.11483	0.075012	0.3451
0	0	1	1	22.12213	0.106842	0.030095	0.016778	0.076101
0	0	1	1	22.15474	0.123926	0.023339	0.004989	0.166246
0	0	2	0.5	22.45019	0.088762	0.016131	0.008748	-0.07708
0	0	2	0.5	22.48313	0.100401	0.018755	0.004899	0.050788
0	0	2	0.5	22.62355	0.200516	0.022865	0.007685	0.072338
0	0	2	0.5	22.92606	0.183289	0.013579	0.006302	0.1842
0	0	2	0.5	22.95357	0.17215	0.042304	0.007855	0.276091
0	0	2	0.5	22.91998	0.150296	0.024386	0.005001	0.15936
0	0	1	1	21.28027	0.530237	0.066354	0.02455	0.2785
0	0	1	1	21.45698	0.562213	0.069101	0.012881	0.075706
0	0	1	1	21.68083	0.621845	0.067885	0.010132	0.158145
0	0	1	1	21.95242	0.527783	0.055935	0.009673	0.130937
0	0	1	1	22.21757	0.630997	0.035698	0.007698	0.034998
1	1	1	1	22.44501	0.694355	0.032787	0.004894	0.155914
1	1	1	1	22.88623	0.499252	0.043289	0.002189	0.309207
1	1	1	1	23.1478	0.510212	0.03989	0.008799	0.23312
0	0	1	1	21.97911	0.419643	0.083266	0.02691	0.373
0	0	1	1	22.12514	0.433021	0.079847	0.017283	0.106847
0	0	1	1	22.18035	0.377618	0.105328	0.022833	0.05711
0	0	1	1	22.35108	0.399645	0.106541	0.027366	0.057712
0	0	1	1	22.56625	0.393744	0.115319	0.029151	0.245258
0	0	1	1	22.64868	0.380996	0.084659	0.032346	-0.1153
0	0	1	1	22.72008	0.412814	0.043081	0.013253	-0.15527
0	0	1	1	22.71738	0.395204	0.031572	0.013516	0.080675
0	0	1	1	22.60969	0.313951	0.017726	0.006251	0.01579
0	0	2	1	21.20857	0.593327	0.049107	0.038736	0.1546
0	0	2	1	21.20264	0.58996	0.030564	0.009176	0.018909
0	0	2	1	21.32464	0.617278	0.036663	0.008183	0.257184
0	0	2	1	21.3306	0.582764	0.061975	0.009931	0.030472
0	0	2	1	21.45826	0.594567	0.060909	0.012619	0.27839
0	0	2	1	21.8852	0.44551	0.052015	0.009681	0.320286
0	0	2	1	21.97474	0.458477	0.053517	0.012939	0.019317

0	0	2	1	21.96774	0.471903	0.000839	0.013933	-0.00866
0	0	2	1	21.99974	0.449323	0.041962	0.004383	-0.00271
0	0	2	1	20.64905	0.187173	-0.06817	-0.00881	-0.25584
0	0	2	1	20.64308	0.180784	0.01351	0.004069	-0.20291
0	0	2	1	21.1336	0.130321	0.003251	-0.01974	0.755911
0	0	2	1	21.09982	0.169529	0.027587	0.005968	0.29323
0	0	2	1	21.14869	0.149861	0.066199	0.023487	0.128499
0	0	2	1	20.46514	0.23656	0.117859	0.117997	0.0799
0	0	2	1	20.56194	0.231877	0.096941	0.029535	0.089612
0	0	2	1	20.6413	0.224719	0.099295	0.028309	0.101988
0	0	2	1	21.21853	0.194595	0.061658	0.025978	0.058546
0	0	2	1	21.65733	0.209107	0.046445	0.020557	0.015823
0	0	2	1	21.62856	0.161903	0.051728	0.011942	0.35359
0	0	2	1	21.66901	0.170245	0.055561	0.015819	-0.11815
0	0	2	1	21.59751	0.223607	0.004483	0.001714	-0.08331
0	0	2	1	21.51543	0.471719	0.059177	0.018995	0.2061
0	0	2	1	21.5224	0.468839	0.037596	0.014771	-0.15458
0	0	2	1	21.66596	0.536239	0.036514	0.00892	0.126619
0	0	2	1	21.76249	0.569502	0.035934	0.007913	0.091367
0	0	2	1	21.81273	0.411487	0.03043	0.009145	-0.04376
0	0	2	1	21.97443	0.480659	0.032763	0.006115	0.102667
0	0	2	1	22.0549	0.511173	0.03641	0.006218	0.407221
0	0	2	1	22.094	0.517622	0.030468	0.009071	0.457527
0	0	2	1	22.09963	0.512399	0.03589	0.007996	-0.09159
1	1	1	1	21.63768	0.264064	0.085996	0.095515	0.4055
1	1	1	1	21.66747	0.251668	0.061491	0.02504	0.21782
1	1	1	1	21.84041	0.336871	0.063086	0.015806	0.069919
1	1	1	1	21.93852	0.366595	0.062452	0.012894	0.079764
1	1	1	1	22.01456	0.3964	0.070105	0.014799	0.035163
1	1	1	1	22.18011	0.382996	0.157203	0.045954	0.128414
1	1	1	1	22.46279	0.311651	0.191765	0.061312	-0.07748
1	1	1	1	22.53961	0.271114	0.149325	0.03713	-0.03894
0	0	2	1	20.59787	0.36788	0.060779	0.047409	0.06776
0	0	2	1	20.60436	0.360649	0.047986	0.011456	0.074809
0	0	2	1	20.71166	0.410378	0.024928	0.007297	0.034321
0	0	2	1	20.801	0.453887	0.015329	0.003834	0.16662
0	0	2	1	20.93603	0.475078	0.026568	0.002834	0.127042
0	0	2	1	21.05486	0.506495	0.036109	0.003017	0.202766
0	0	2	1	21.57717	0.344909	0.047126	0.002433	0.663983
0	0	2	1	21.60952	0.351414	0.067826	0.005414	0.314159
0	0	2	1	21.70509	0.337227	0.069367	0.006834	0.058712
0	0	1	1	21.2654	0.102636	0.098197	0.102568	0.0999
0	0	1	1	21.36231	0.160957	0.051894	0.016789	-0.06992
0	0	1	1	21.45107	0.204904	0.0473	0.011286	0.321382
0	0	1	1	22.15808	0.134009	0.05681	0.012342	0.39497
0	0	1	1	22.34677	0.21249	0.0947	0.015284	0.767185
0	0	1	1	22.42873	0.200676	0.11091	0.028748	0.142135
0	0	2	1	22.58171	0.20528	0.15229	0.027832	0.010104
0	0	2	1	23.54143	0.17088	0.062978	0.023804	1.327245
0	0	2	1	24.20016	0.231476	0.101178	0.01373	0.808188
1	1	2	0.5	22.16438	0.123958	0.12455	0.086932	0.1843
1	1	2	0.5	22.15016	0.076998	0.041462	0.020065	-0.15592
0	0	2	0.5	22.26186	0.085807	0.054804	0.016859	0.5104
0	0	2	0.5	22.35013	0.104917	0.079073	0.024338	0.173079
0	0	2	0.5	22.46495	0.15412	0.087562	0.027615	0.090237
0	0	2	0.5	22.55592	0.146842	0.102927	0.037327	0.199515
0	0	2	0.5	22.78853	0.098332	0.080636	0.03246	0.06835
0	0	2	0.5	22.87004	0.116108	0.079946	0.043043	0.179672

0	0	2	0.5	22.92817	0.123145	0.077947	0.039157	0.209933
0	0	3	0.666667	20.60505	0.148244	0.111762	0.06007	0.1555
0	0	3	0.666667	20.69105	0.170222	0.123893	0.039946	-0.01445
0	0	3	0.666667	20.82837	0.225396	0.137966	0.050643	0.100948
0	0	3	0.666667	21.09548	0.334607	0.116838	0.045924	0.058078
0	0	3	0.666667	21.09529	0.268005	0.092833	0.033372	0.08034
0	0	3	0.666667	21.4162	0.227047	0.071424	0.0283	-0.12229
0	0	3	0.666667	21.39069	0.155648	0.052633	0.017305	-0.07061
0	0	3	0.666667	21.70258	0.292608	0.086985	0.009213	0.424186
0	0	3	0.666667	21.82884	0.479907	0.173767	0.043	0.840169
0	0	1	0	20.97469	0.456399	0.061674	0.038414	0.0951
0	0	1	0	21.02526	0.462971	0.039702	-0.00172	-0.01717
1	1	1	0	21.05889	0.478155	0.038848	0.001419	-0.02572
1	1	1	0	21.00542	0.428603	0.03307	0.007025	-0.05088
1	3	1	0	21.03587	0.429619	0.037114	-0.00801	0.029182
1	1	2	0	21.05678	0.37251	0.079484	0.021796	0.236439
1	1	2	0	21.11091	0.39147	0.058997	0.0257	0.106211
1	1	3	0.333333	21.00344	0.331961	-0.01666	0.011762	-0.27486
0	0	2	0.5	21.23218	0.518551	0.092911	0.074847	0.1772
0	0	2	0.5	21.58747	0.630768	0.060867	0.020464	0.153352
0	0	2	0.5	21.81724	0.696603	0.045701	0.019135	0.222758
0	0	2	0.5	21.94954	0.75227	0.009537	0.013521	0.037892
0	0	2	0.5	22.02267	0.767387	0.030518	0.003871	-0.00309
0	0	2	0.5	22.16966	0.554862	0.031403	0.009669	0.256693
0	0	2	0.5	22.25536	0.581168	0.03405	0.009905	0.015771
0	0	2	0.5	22.69763	0.589847	0.185642	0.006264	-0.06413
0	0	2	0.5	22.70868	0.41166	0.055997	0.014524	0.131003
0	0	2	0.5	22.39077	0.304427	0.046648	0.01476	0.129889
0	0	2	1	21.17892	0.442662	0.087617	0.056777	0.0648
0	0	2	1	21.34421	0.516962	0.055634	0.014689	-0.095
0	0	2	1	21.50707	0.581721	0.054022	0.014512	0.050711
0	0	2	1	21.51939	0.609841	0.028524	0.007736	-0.02983
1	1	2	1	21.3454	0.490369	0.090904	0.019773	-0.33151
1	1	2	1	21.91732	0.307085	0.025592	0.004428	-0.21158
1	1	2	1	22.56477	0.593684	0.072681	0.017215	1.324758
1	1	2	1	22.82384	0.647724	0.027853	0.015694	-0.18804
0	0	2	1	20.51795	0.259348	0.091335	0.039436	0.0758
0	0	2	1	20.60566	0.30726	0.043034	0.007307	-0.10682
0	0	2	1	20.73116	0.354707	0.054306	0.008151	0.638868
0	0	2	1	20.75352	0.449747	0.028623	0.006058	-0.25619
0	0	2	1	22.02625	0.129431	0.050011	0.004925	0.302527
0	0	2	1	22.07795	0.118043	0.059084	0.009645	0.150013
1	3	2	1	22.65917	0.162067	0.063745	0.015499	0.501097
1	3	2	1	22.6891	0.096854	0.100521	0.019282	-0.01869
1	1	1	1	20.72221	0.045617	0.104595	0.078676	0.099
1	1	1	1	20.76937	0.049037	0.04676	0.020178	-0.2306
1	1	1	1	20.79717	0.035936	0.04249	0.014725	-0.11592
1	1	1	1	20.86076	0.047872	0.04063	0.015751	0.137007
1	1	1	1	20.87283	0.023457	0.042242	0.007902	-0.26458
0	0	2	0.5	20.90135	0.055876	0.085911	0.085911	-0.1143
0	0	2	0.5	20.88596	0.033222	0.031933	0.000243	-0.33035
0	0	2	0.5	20.94001	0.052957	0.016874	-0.00035	-0.24522
0	0	2	0.5	21.06345	0.132082	0.021186	-0.0009	-0.12799
1	3	2	0.5	21.02461	0.146318	0.062155	0.006588	-0.09023
0	0	1	1	20.36796	0.30635	0.097492	0.097492	0.1564
0	0	1	1	20.39786	0.320356	0.043011	0.018196	-0.03546
0	0	1	1	20.44759	0.331326	0.029076	0.009585	0.16786
0	0	1	0	19.89863	0.119881	0.118109	0.118109	0.1778

0	0	1	0	19.98053	0.133072	0.077775	0.010112	0.051751
0	0	1	0	20.07569	0.165296	0.067016	0.014456	0.240006
0	0	1	0	20.27053	0.254754	0.073802	0.01461	0.380027
0	0	1	0	20.37188	0.248195	0.071483	0.015897	0.00657
0	0	1	0	20.42189	0.185389	0.106653	0.014278	0.041839
0	0	1	1	20.71636	0.117247	0.091569	0.093579	0.2475
0	0	1	1	20.71915	0.101985	0.03606	0.008941	-0.11175
0	0	1	1	20.78049	0.148619	0.021753	0.003139	0.120677
0	0	1	1	20.78799	0.140122	0.027792	0.006139	0.139148
0	0	1	1	21.17089	0.09474	0.029892	0.011318	-0.07192
0	0	1	1	21.19823	0.095412	0.029562	0.007649	0.214889
0	0	1	1	21.5397	0.188886	0.072563	0.01385	0.546206
0	0	2	1	21.55209	0.124419	0.106015	0.028588	0.484082
0	0	2	1	21.77665	0.316984	0.082548	0.027355	0.162263
0	0	1	1	20.64573	0.375081	0.067663	0.064351	0.1583
0	0	1	1	20.66875	0.383093	0.023062	0.005249	0.085694
0	0	1	1	20.6208	0.384473	-0.00862	-0.00271	-0.06577
0	0	1	1	20.65949	0.451744	-0.02156	0.000954	0.071564
1	3	1	1	20.22431	0.151213	-0.03727	-0.00213	-0.39909
1	3	1	1	20.23405	0.15061	0.008018	-0.00165	0.257286
0	0	2	1	20.24549	0.153724	0.006046	-0.00641	-0.07053
0	0	2	1	20.233	0.190226	-0.04684	-0.00204	-0.2413
1	1	1	0	22.62227	0.519182	0.084228	0.012326	-0.06856
1	1	1	0	21.66017	0.282032	0.071087	0.014904	0.473848
1	1	1	0	21.65685	0.216197	0.083971	0.018109	0.128096
0	0	2	0.5	21.51935	0.51105	0.073129	0.060962	0.0336
0	0	2	0.5	21.74518	0.604224	0.032833	0.015573	-0.06053
0	0	2	0.5	21.69879	0.549706	0.066348	0.008526	-0.01034
1	3	2	0.5	21.70465	0.580125	-0.00723	0.001328	0.24548
1	3	2	0.5	21.47329	0.467896	0.051002	0.007436	-0.03871
0	0	2	1	21.45785	0.223189	0.108815	0.110872	0.2233
0	0	2	1	21.3475	0.089799	0.112802	0.029954	-0.0156
0	0	2	1	21.47494	0.176693	0.098018	0.027728	0.012954
0	0	2	1	21.55532	0.242608	0.078765	0.023876	0.00401
0	0	2	1	21.71543	0.315925	0.080093	0.032606	0.041702
0	0	2	1	21.77079	0.315597	0.071597	0.027573	0.075028
1	1	2	1	21.7798	0.284171	0.072191	0.026678	0.002401
1	1	2	1	21.68691	0.170487	0.058975	0.025803	-0.05436
1	3	2	1	21.72579	0.142494	0.071411	0.027192	0.140537
1	1	1	0	21.61003	0.217109	0.199958	0.20084	-0.1576
1	1	1	0	21.69303	0.217025	0.111463	0.032495	-0.34063
1	1	1	0	21.64914	0.252442	0.126119	0.024321	0.098491
1	1	1	0	21.56813	0.194529	0.116673	0.035677	-0.16288
1	1	1	0	21.63863	0.254135	0.056863	0.01689	-0.22464
1	1	1	0	21.63392	0.234461	0.039857	0.01066	-0.19388
1	1	1	0	21.62447	0.196859	0.055914	0.01507	0.136727
1	1	1	0	21.64384	0.18489	0.050685	0.016444	-0.01711
1	1	1	0	21.70295	0.173739	0.073984	0.0229	0.331459
0	0	2	0	19.87139	0.417852	0.15536	0.15536	0.601
0	0	2	0	20.29551	0.240038	0.06715	0.021914	0.037552
0	0	2	0	20.48371	0.321504	0.065515	0.019068	0.123241
0	0	2	0	20.6986	0.427008	0.047951	0.012569	0.199075
0	0	2	0	21.4591	0.365075	0.032714	0.003468	0.603429
0	0	2	0	21.91507	0.330314	0.051486	0.003205	0.871461
1	1	2	0	22.18356	0.387012	0.043449	0.00832	0.937448
0	0	1	1	20.5626	0.216681	0.125563	0.022299	0.1895
0	0	1	1	20.71308	0.27608	0.112339	0.027475	0.22203
0	0	1	1	20.77611	0.282488	0.090021	0.0341	0.246846

0	0	1	1	21.62178	0.185897	0.058068	0.031305	0.19411
0	0	1	1	21.62889	0.151418	0.07004	0.01712	0.186451
0	0	1	1	21.81964	0.279399	0.05493	0.017486	0.04893
0	0	1	1	22.09943	0.434023	0.067551	0.031954	0.258608
0	0	1	1	22.13932	0.454565	0.07573	0.019764	0.062218
0	0	2	1	21.12728	0.290457	0.082225	0.022897	0.2585
0	0	2	1	21.19642	0.264089	0.103712	0.020457	0.345083
0	0	2	1	21.34607	0.282047	0.121756	0.023866	0.172249
0	0	2	1	21.73178	0.441642	0.085961	0.026389	-0.02916
0	0	2	1	21.68109	0.333559	0.101958	0.0255	0.179833
1	1	1	1	21.97396	0.413087	0.094332	0.026224	0.359137
1	1	1	1	21.95899	0.335091	0.09904	0.02227	0.087699
1	1	1	1	22.16995	0.388744	0.09114	0.023256	0.129684
0	0	3	0.666667	21.16612	0.107368	0.121773	0.023829	0.1904
0	0	3	0.666667	21.29943	0.154559	0.112753	0.018533	0.22157
0	0	3	0.666667	21.41743	0.191418	0.103622	0.022704	0.056573
0	0	3	0.666667	21.89058	0.174488	0.072013	0.014489	0.149596
0	0	3	0.666667	21.94318	0.186871	0.055928	0.014074	0.197131
0	0	3	0.666667	21.98477	0.188792	0.058616	0.011975	0.326773
0	0	3	0.666667	22.05139	0.211332	0.055642	0.007766	0.185693
0	0	3	0.666667	22.12098	0.260388	0.069719	0.008608	0.032628
0	0	2	0	20.51556	0.180475	0.086884	0.019945	0.0042
0	0	2	0	20.6024	0.190677	0.067194	0.011919	-0.07768
0	0	2	0	20.59991	0.141606	0.052916	0.014253	-0.01312
0	0	2	0	20.56212	0.106055	-0.00224	0.010757	-0.31066
0	0	2	0	20.54429	0.119848	-0.03335	-0.00014	-0.43683
0	0	2	0	20.59695	0.13673	0.02273	0.009073	0.983288
0	0	2	0	20.65233	0.172999	0.013865	0.013353	0.025119
1	3	2	0	20.64468	0.159415	0.012455	0.015324	0.20082
0	0	1	0	20.22131	0.086246	0.059898	0.032466	-0.1858
0	0	1	0	20.29741	0.136138	0.06446	0.013147	0.300479
0	0	1	0	20.46915	0.197794	0.109709	0.014231	0.641737
1	3	1	0	20.62342	0.203583	0.14921	0.045104	0.378973
1	3	1	0	21.30863	0.103832	0.055109	0.038225	-0.18286
1	3	2	0	21.29245	0.073466	0.022361	0.013881	-0.07421
1	3	2	0	21.53853	0.153088	0.039796	0.004441	0.687981
0	0	2	1	20.81581	0.31407	0.097142	0.04714	-0.178
1	1	2	1	21.03594	0.419884	0.099006	0.023086	0.166772
1	1	2	1	21.02953	0.39159	0.105073	0.025608	0.006852
1	1	1	0	21.20541	0.132092	0.108653	0.050564	0.2046
1	1	1	0	21.37333	0.189959	0.102222	0.0186	0.153545
1	1	1	0	21.56436	0.302884	0.065778	0.017871	0.254728
1	1	1	0	21.65927	0.376356	0.030405	0.00464	0.055617
1	1	1	0	21.71138	0.124678	0.03449	0.002468	0.088081
1	1	1	0	21.69713	0.112278	0.015623	0.004498	-0.17659
0	0	2	0.5	21.80989	0.218842	0.037072	0.001375	-0.14481
0	0	2	0.5	21.81366	0.224947	0.0397	0.006766	-0.12159
0	0	2	0	20.80794	0.066931	0.109352	0.072868	-0.3146
0	0	2	0	20.92906	0.103522	0.104992	0.036266	0.31812
0	0	2	0	21.05498	0.150044	0.093916	0.023688	-0.0306
0	0	1	0	21.65854	0.122431	0.050347	0.019482	0.058446
0	0	1	0	22.1744	0.277405	0.056184	0.012634	0.457325
0	0	1	0	22.38589	0.376318	0.071434	0.016227	0.377358
0	0	1	0	22.6645	0.338254	0.07717	0.015186	0.462034
1	1	1	0	22.71045	0.344902	0.062115	0.017429	0.282892
0	0	2	0.5	20.72553	0.13953	0.097289	0.050206	0.0314
0	0	2	0.5	20.79378	0.142547	0.11501	0.023989	0.079623
0	0	2	0.5	20.84652	0.126719	0.13652	0.024659	0.201492

0	0	2	0.5	20.91545	0.13897	0.147361	0.024008	0.102787
0	0	2	0.5	21.05804	0.176424	0.167994	0.028236	0.285408
0	0	2	0.5	21.23949	0.219177	0.212867	0.032046	0.541812
1	3	2	1	22.03013	0.110346	0.093615	-0.00112	0.2777
0	0	2	1	22.11895	0.112712	0.121534	0.019089	0.279559
0	0	2	1	22.22962	0.140704	0.147339	0.025978	0.526043
1	1	1	0	20.77987	0.156348	0.082197	0.040383	-0.0274
1	1	1	0	20.91047	0.176084	0.127314	0.020491	0.345564
1	1	1	0	21.05305	0.21016	0.110378	0.018682	0.020064
1	1	1	0	21.86229	0.204058	0.099814	0.013686	0.596079
0	0	1	0	22.14544	0.314932	0.102154	0.021459	0.219825
0	0	1	0	22.89595	0.298252	0.0641	0.014352	0.525902
0	0	1	0	22.76316	0.319903	-0.08924	0.012053	0.042259
0	0	2	1	20.42172	0.275202	0.077497	0.044928	0.2028
0	0	2	1	20.5703	0.39905	-0.00903	-0.00494	-0.40563
0	0	2	1	20.72826	0.476393	0.024208	0.000544	0.695076
0	0	2	1	21.19253	0.456722	0.003057	0.002423	0.12727
0	0	2	1	22.54286	0.726163	0.026846	0.002811	2.693703
0	0	2	1	23.12403	0.74851	0.00933	0.004984	0.920677
0	0	1	1	20.63825	0.145591	0.060655	0.017267	-0.1395
0	0	1	1	20.72668	0.216081	0.010991	0.002056	-0.09208
0	0	1	1	20.81044	0.255779	0.006453	0.001243	0.293915
0	0	2	1	20.89996	0.293367	0.025482	0.002927	2.080451
0	0	1	1	21.36277	0.282286	0.034001	0.004241	0.392956
0	0	1	1	22.49834	0.249002	0.037725	0.008748	1.337089
0	0	1	1	22.50395	0.203415	0.066941	0.030877	0.98877
0	0	1	1	22.71739	0.312739	0.062573	0.010636	0.37593
0	0	2	0	20.85377	0.166965	0.099758	0.091316	0.0869
0	0	2	0	20.88756	0.180913	0.059565	0.008914	-0.09177
0	0	2	0	21.04562	0.2626	0.063335	0.01017	0.131091
0	0	2	0	21.04171	0.243275	0.060812	0.008218	0.019792
0	0	2	0	21.06602	0.240662	0.064297	0.00955	0.045997
0	0	2	0	21.09135	0.276161	0.070772	0.009923	0.120002
0	0	2	0	21.17057	0.290604	0.097642	0.012798	0.276997
1	1	2	0	21.22663	0.294354	0.111423	0.01681	0.115949
1	1	1	1	20.72663	0.139356	0.119431	0.100549	0.0689
1	1	1	1	20.74	0.114868	0.109394	0.022244	-0.03808
1	1	1	1	21.14107	0.370401	0.062784	0.023517	0.133232
1	1	1	1	21.3573	0.44063	0.077701	0.019778	0.488997
1	1	1	1	21.70247	0.404985	0.079996	0.031101	0.196101
1	1	1	1	21.80843	0.446835	0.07249	0.023385	-0.02531
1	1	1	1	21.92784	0.466506	0.065955	0.02375	0.117342
1	1	1	1	21.93052	0.459711	0.078916	0.021351	0.09422
1	3	1	1	20.59082	0.134323	0.098866	0.084814	0.2519
1	3	1	1	20.62954	0.132302	0.047882	0.011899	-0.1626
1	3	1	1	20.64628	0.11018	0.044419	0.012408	-0.06452
1	1	1	1	20.72787	0.156932	0.02872	0.00833	-0.08689
1	1	1	1	20.73852	0.136479	0.019082	0.002607	0.036747
1	1	1	1	20.82268	0.19215	0.021999	0.003585	-0.06087
0	0	1	1	20.60296	0.046018	0.083087	0.060181	-0.0288
0	0	1	1	20.63667	0.050623	0.077176	0.006543	0.153786
0	0	1	1	20.66515	0.052979	0.068337	0.005934	0.077492
0	0	1	1	21.16162	0.401607	0.049573	0.006793	0.432839
1	1	1	1	21.04326	0.446883	0.016649	-0.00324	0.700266
1	1	1	1	20.83681	0.402196	-0.06338	-0.0028	0.366631
1	1	1	1	20.67301	0.286778	0.021292	-0.01027	0.027891
1	1	2	0.5	21.30859	0.414769	0.090338	0.070938	0.0909
0	0	2	0.5	21.4221	0.473943	0.068003	0.008586	0.168667

0	0	1	1	21.59563	0.551783	0.029284	0.010117	0.0418
1	1	2	1	21.53098	0.565426	0.033887	0.004543	-0.03568
0	0	2	1	21.0691	0.068241	0.090847	0.06989	0.0039
0	0	2	1	21.11634	0.047825	0.085282	0.027202	0.004114
0	0	2	1	21.19326	0.073802	0.061356	0.026135	0.101563
0	0	2	1	21.3265	0.152932	0.039033	0.015447	0.169815
0	0	2	1	21.37243	0.157595	0.062549	0.013354	0.057986
0	0	2	1	21.4547	0.155882	0.088417	0.021879	0.213462
0	0	2	1	21.63837	0.225646	0.086159	0.026856	0.284074
0	0	2	1	21.74027	0.20588	0.095259	0.028825	0.115588
0	0	2	1	20.93558	0.2616	0.08157	0.061022	0.1559
0	0	2	1	21.01776	0.29783	0.058443	0.004048	0.152754
0	0	2	1	21.25827	0.417202	0.052078	0.007302	0.319488
0	0	2	1	21.35783	0.432883	0.042105	-0.00704	0.046565
0	0	2	1	21.37257	0.414065	0.046936	0.002857	0.109879
0	0	2	1	21.99882	0.357314	0.047937	0.003694	0.157409
0	0	2	1	22.0363	0.37934	0.026244	0.006112	0.308862
1	1	2	1	21.81021	0.526952	-0.08172	0.004032	-0.09237
0	0	1	1	21.69836	0.363885	0.078196	0.060498	0.1326
0	0	1	1	21.77456	0.377344	0.057516	0.019266	0.136202
0	0	1	1	21.85097	0.381467	0.065556	0.01846	0.073736
0	0	1	1	22.01579	0.441059	0.060283	0.017644	0.15154
0	0	1	1	22.10635	0.453322	0.045491	0.015696	0.152282
0	0	1	1	22.18381	0.461764	0.053633	0.013331	0.097652
0	0	1	1	22.29064	0.454629	0.079621	0.016406	0.040507
0	0	1	1	22.35403	0.401211	0.112592	0.019847	0.083485
1	1	1	1	22.28632	0.377695	0.128964	0.128964	0.2343
1	1	1	1	22.40435	0.381644	0.160262	0.041458	0.30247
1	1	1	1	22.63498	0.429455	0.167292	0.045758	0.194352
1	1	1	1	23.07287	0.569505	0.144051	0.051881	0.221412
1	1	1	1	23.42721	0.645846	0.121466	0.040359	0.140536
1	1	1	1	23.38688	0.591707	0.097037	0.02035	-0.03373
1	1	1	1	23.32175	0.582218	0.061906	0.028991	0.113453
1	1	1	1	23.41336	0.594529	0.091189	0.029515	0.14602
0	0	2	0.5	20.89068	0.379291	0.074491	0.074491	-0.1239
0	0	2	0.5	20.99646	0.409939	0.047347	0.014638	0.230152
0	0	2	0.5	21.46571	0.313629	0.028931	0.013461	0.202559
0	0	2	0.5	21.35762	0.224488	0.033841	0.011137	0.032638
0	0	2	0.5	21.4531	0.279974	0.03796	0.010111	0.356539
0	0	2	0.5	21.46731	0.292436	0.01511	0.009904	0.015714
0	0	2	0.5	21.4387	0.271166	0.000707	-0.00456	-0.01833
1	1	2	1	22.61829	0.437724	0.084163	0.014972	0.17708
1	1	2	1	22.68771	0.450662	0.077275	0.016638	0.027004
1	1	2	1	22.82137	0.469019	0.097375	0.018953	0.080551
0	0	2	0	20.49522	0.369931	0.090664	0.090664	0.0449
1	1	2	0	20.84468	0.201019	0.065722	0.020318	0.071681
1	1	2	0	20.83266	0.183614	0.037021	0.019359	-0.12576
1	1	2	0	21.59494	0.365928	0.042353	0.006321	1.044978
1	1	2	0	21.57623	0.357031	0.021974	0.011342	0.313126
1	1	2	0	21.50162	0.352432	-0.0305	0.00789	-0.07244
1	1	1	1	22.00672	0.506464	0.098376	0.024402	1.241511
0	0	2	1	19.99648	0.332684	0.137809	0.137809	-0.0385
0	0	2	1	20.48886	0.180214	0.060979	0.009258	-0.171
0	0	2	1	21.08767	0.220485	0.059416	0.00883	0.612336
0	0	2	1	21.0339	0.162873	0.025041	0.005777	0.080398
0	0	2	1	21.07762	0.191761	0.016959	0.003153	0.035327
0	0	2	1	20.92665	0.329362	-0.05669	0.003444	0.117235
0	0	3	0.666667	20.70516	0.332727	0.041408	0.014758	0.204605

1	1	3	0.666667	20.77115	0.38849	-0.00431	0.009687	0.095961
1	1	3	0.666667	20.76448	0.372515	0.019373	0.005455	0.281502
1	1	2	1	20.31591	0.282815	0.179106	0.179106	0.0374
0	0	2	1	20.77395	0.17231	0.100422	0.029162	-0.0097
0	0	2	1	20.73985	0.126697	0.045797	0.017314	-0.22648
0	0	2	1	20.76069	0.124187	0.035888	0.015573	-0.00147
0	0	2	1	20.8353	0.187302	0.027799	0.010464	0.216797
0	0	2	1	20.90731	0.205109	0.040438	0.011396	0.236099
0	0	2	1	20.85121	0.152414	0.033772	0.027105	-0.26093
1	1	1	1	21.2772	0.129539	0.066363	0.034138	0.1229
1	1	1	1	21.3811	0.157664	0.064462	0.020782	0.204137
1	1	1	1	21.55578	0.196462	0.104341	0.03018	0.276211
1	3	1	1	21.65688	0.201419	0.073438	0.033698	0.205976
1	3	1	1	22.04626	0.400881	0.05861	0.030157	0.335609
0	0	2	1	20.53162	0.108966	0.104666	0.088164	0.0059
0	0	2	1	21.19526	0.446993	0.076304	0.017043	0.269712
0	0	2	1	21.38026	0.459384	0.071091	0.016058	0.047399
0	0	2	1	21.44965	0.462563	0.104879	0.020742	0.28828
0	0	2	1	21.53722	0.489085	0.107249	0.030329	0.040845
1	3	1	0	19.93126	0.075642	0.065892	0.0111	0.102679
1	3	1	0	19.99667	0.103468	0.047904	0.013239	0.042977
1	3	1	0	20.01279	0.089105	0.0515	0.014058	0.006885
0	0	2	0.5	20.92336	0.353771	0.067711	0.055587	-0.1281
0	0	2	0.5	20.99152	0.343679	0.061705	0.007704	-0.08225
0	0	2	0.5	21.0842	0.371255	0.047701	0.007859	0.170731
0	0	2	0.5	21.17504	0.39178	0.062291	0.007876	0.312248
0	0	2	0.5	21.27133	0.41932	0.055771	0.008175	0.156922
0	0	2	0.5	21.18088	0.341819	0.053811	0.011846	0.067692
1	1	1	1	21.39523	0.472882	0.088898	0.088898	-0.1009
0	0	1	1	21.55072	0.527666	0.057451	0.024651	-0.09314
0	0	1	1	21.66051	0.562413	0.049813	0.004494	-0.26062
0	0	1	1	21.68849	0.558166	0.052359	-0.00054	0.238449
0	0	1	1	21.83918	0.613281	0.039624	0.008878	-0.01977
0	0	1	1	21.94765	0.645956	0.042816	0.012203	-0.15562
0	0	2	0.5	20.65135	0.454398	0.075996	0.075996	0.1381
1	1	2	0.5	20.86402	0.328379	0.071357	0.016629	0.10105
1	1	2	0.5	21.45493	0.234897	0.064854	0.030227	0.390489
1	1	2	0.5	21.59283	0.274974	0.081481	0.028181	0.226638
1	1	2	0.5	21.72951	0.313852	0.091393	0.033622	0.067276
1	1	2	0.5	22.28504	0.518523	0.08585	0.035338	0.864605
0	0	3	0.333333	22.04048	0.389319	0.117571	0.117571	0.2466
0	0	3	0.333333	22.04891	0.306579	0.106906	0.032281	0.116233
0	0	3	0.333333	22.16396	0.336016	0.101855	0.036211	0.108348
0	0	2	0	22.22069	0.292736	0.127951	0.028818	0.146136
1	1	2	0	22.34446	0.320656	0.142426	0.040863	0.159953
0	0	2	0	22.39272	0.354029	0.146227	0.038511	-0.02244
1	3	1	1	23.46143	0.410338	0.104868	0.068021	0.498548
1	3	1	1	23.67253	0.455688	0.104566	0.040964	0.401037
1	3	1	1	23.86492	0.49404	0.090992	0.031071	0.180212
1	3	1	1	23.86347	0.454359	0.081683	0.031807	0.064913
1	1	2	1	20.61825	0.190585	0.080847	0.008846	0.1154
1	1	2	1	21.48434	0.424907	0.080516	0.017257	0.110204
1	1	2	1	21.7527	0.516574	0.06438	0.01711	0.072261
1	1	2	1	22.16716	0.614301	0.067009	0.01611	0.25758
1	1	1	1	22.34016	0.651408	0.059707	0.01797	0.506063
0	0	1	1	22.33774	0.640404	0.06026	0.016449	0.109895
0	0	1	1	21.85415	0.197442	0.133048	0.011646	0.191219
0	0	1	1	20.6291	0.086213	0.168338	0.042153	0.0369

0	0	1	1	20.71797	0.099333	0.155836	0.040614	0.015277
0	0	1	1	20.70867	0.131201	0.216313	0.044223	0.200338
0	0	1	1	21.01746	0.169169	0.167751	0.046749	0.17201
0	0	3	0.333333	19.89221	0.344403	0.176269	0.176269	0.0478
0	0	3	0.333333	20.36496	0.142305	0.101074	0.025174	-0.03188
0	0	3	0.333333	20.44841	0.11745	0.131113	0.025954	0.232616
0	0	3	0.333333	20.48149	0.177896	0.050803	0.020323	0.361383
0	0	3	0.333333	20.48444	0.167122	0.006842	0.00947	0.103588
0	0	3	0.333333	20.5087	0.174748	-0.03084	0.005335	-0.18064
1	1	1	0	21.13079	0.694303	0.04883	0.044494	0.2371
1	1	1	0	21.3069	0.755722	0.02601	0.004109	0.19228
1	1	1	0	21.52582	0.789853	0.031197	0.004082	0.143585
0	0	1	0	21.82717	0.663354	0.04449	0.00438	0.53467
0	0	1	0	22.02714	0.548283	0.03765	0.005892	-0.06984
1	1	1	0	20.76326	0.450652	0.037071	0.025537	-0.2012
1	1	1	1	20.79069	0.466234	0.01563	0.01201	-0.26416
1	1	1	1	20.63587	0.473569	-0.0405	-0.00259	0.197472
0	0	1	1	20.62051	0.431662	0.035666	0.002528	0.27519
0	0	2	0	20.79176	0.082673	0.037091	0.020947	0.054923
1	1	2	0	20.79538	0.072319	0.045996	0.016093	-0.03414
1	1	1	0	21.35676	0.195693	0.128068	0.084399	0.1413
0	0	1	0	21.40523	0.162806	0.131832	0.053823	0.085415
0	0	1	0	21.51149	0.177339	0.146593	0.067357	0.042742
0	0	2	0	21.6097	0.233301	0.109392	0.065228	0.102249
0	0	2	0	21.6436	0.232579	0.106346	0.065549	0.097905
1	1	2	1	21.18038	0.353932	0.063673	0.038182	0.0034
1	1	2	1	21.51359	0.51359	0.071345	0.022523	0.481602
0	0	2	1	21.61653	0.494406	0.065834	0.015385	0.046913
0	0	2	1	21.64011	0.504536	0.008626	0.014327	-0.28629
0	0	2	1	21.97061	0.562657	-0.05508	-0.01041	0.310467
0	0	2	0.5	20.40281	0.152097	0.077258	0.03477	-0.0508
0	0	2	0.5	20.46189	0.134932	0.060755	0.013567	0.006063
0	0	2	0.5	20.5194	0.14772	0.056926	0.006184	0.057688
0	0	2	0.5	20.54346	0.14437	0.030922	0.006526	-0.0671
0	0	2	0.5	20.60751	0.169571	0.03413	0.002251	0.09939
1	1	2	1	21.78839	0.129668	0.15194	0.056834	0.2388
1	1	2	1	22.04719	0.227045	0.144663	0.036067	0.224494
1	1	2	1	22.24631	0.22736	0.159779	0.042013	0.097032
1	1	2	1	22.37094	0.21385	0.145031	0.050551	0.046994
0	0	2	1	22.48577	0.207047	0.138053	0.046113	0.116535
0	0	1	0	21.15204	0.310753	0.053826	0.008912	0.170473
0	0	1	0	21.20958	0.336005	0.012277	-0.01013	-0.06289
0	0	2	1	21.87741	0.250278	0.052702	0.023653	-0.08451
0	0	1	1	20.46698	0.185169	0.032553	0.010416	0.199012
0	0	1	1	20.50883	0.203432	0.028262	0.008121	0.042736
1	1	1	1	21.96131	0.557432	0.075145	0.075145	0.0419
1	1	1	1	22.00159	0.545652	0.05754	0.021131	-0.01513
1	1	1	1	22.19136	0.579692	0.059444	0.016932	0.190418
1	1	3	0.666667	22.32064	0.602633	0.067881	0.016303	0.276626
1	1	3	0.666667	22.28654	0.576227	0.047189	0.01573	-0.197
0	0	2	1	20.9835	0.298972	0.088951	0.088951	0.0936
0	0	2	1	20.91213	0.214322	0.110702	0.031927	0.106349
0	0	2	1	21.02609	0.257214	0.085464	0.022712	0.141896
1	1	2	1	21.56095	0.377341	0.03716	0.011479	0.203913
1	1	2	1	21.58376	0.349473	0.050518	0.00897	0.204108
0	0	1	0	21.29411	0.668941	0.079618	0.079618	0.0242
0	0	1	0	21.79414	0.629895	0.05273	0.017611	0.147526
0	0	1	0	22.01071	0.679348	0.052209	0.015413	0.149581

0	0	1	0	22.11218	0.690104	0.055589	0.014821	0.212087
0	0	1	0	22.24229	0.741345	0.033927	0.017481	0.017297
0	0	2	0.5	20.51611	0.140928	0.133284	0.07432	0.091
0	0	2	0.5	20.61671	0.161016	0.162866	0.03169	0.299102
0	0	2	0.5	21.06097	0.350885	0.086741	0.032953	0.129705
0	0	2	0	20.24475	0.215025	0.089266	0.041671	0.0751
0	0	2	0	20.36346	0.295805	0.0409	0.00918	0.160054
0	0	2	0	21.42356	0.286127	0.027467	0.006535	1.219393
0	0	2	0	21.61192	0.364407	0.050115	0.009531	0.462058
0	0	2	1	22.4848	0.442902	0.079253	0.012223	7.073093
0	0	2	1	22.66949	0.479807	0.097412	0.014398	0.292896
0	0	1	0	20.55345	0.114463	0.131938	0.065187	0.1109
0	0	1	0	20.70248	0.143578	0.130607	0.030411	0.289448
0	0	1	0	20.81751	0.172299	0.105648	0.024942	0.136986
0	0	1	0	20.92988	0.171084	0.153222	0.033014	0.228237
0	0	1	0	20.74184	0.130369	0.07485	0.060163	-0.0268
0	0	1	0	20.94579	0.236068	0.063554	0.016843	0.444158
0	0	1	0	21.07421	0.270749	0.054954	0.008976	0.476632
0	0	1	0	21.24736	0.313076	0.085382	0.012251	0.242569
0	0	1	1	20.62579	0.299928	0.070169	0.049182	0.1503
0	0	1	1	20.82642	0.387322	0.058948	0.020866	0.142468
0	0	1	1	21.00227	0.415887	0.068988	0.016056	0.401769
0	0	1	1	21.11661	0.369781	0.129081	0.029324	0.218735
0	0	1	0	21.35981	0.167939	0.104336	0.073586	0.013
0	0	1	0	21.43523	0.187911	0.086605	0.013657	0.06465
0	0	1	0	22.76481	0.471312	0.104249	0.013442	1.013893
0	0	1	0	23.22414	0.599684	0.095209	0.032183	0.285685
0	0	1	1	20.36567	0.097815	0.08766	0.08766	0.1168
0	0	1	1	20.42045	0.098316	0.072494	0.021298	0.008894
0	0	1	1	20.47485	0.106976	0.042136	0.013393	0.030612
0	0	1	1	20.53911	0.126807	0.044221	0.01004	0.104672
0	0	1	1	21.93746	0.425367	0.067293	0.067293	0.0358
0	0	1	1	22.14153	0.507279	0.059431	0.010669	0.178801
0	0	1	1	22.27979	0.542219	0.066956	0.012217	0.306555
0	0	1	1	22.52582	0.523819	0.064284	0.013763	0.172085
0	0	2	0.5	20.41688	0.367171	0.088451	0.088451	-0.0191
0	0	2	0.5	21.14142	0.432285	0.059183	0.019952	0.365258
0	0	2	0.5	21.41759	0.521628	0.079778	0.018505	0.716657
0	0	2	0.5	21.54448	0.568812	0.045885	0.01915	-0.11663
0	0	2	0.5	20.36533	0.230543	0.079471	0.023145	0.3481
0	0	2	0.5	20.83883	0.456664	0.049865	0.014093	0.788513
0	0	2	0.5	21.10303	0.480708	0.076198	0.026667	0.429125
1	1	1	1	21.40548	0.392911	0.028562	0.009423	-0.1723
1	1	1	1	21.64542	0.534495	0.015973	0.005983	0.449655
1	1	1	1	21.73556	0.533172	0.022165	0.00508	0.261482
0	0	1	0	21.06062	0.534193	0.042312	0.011511	-0.0058
0	0	1	0	21.09284	0.542689	0.026081	0.005706	0.012876
0	0	1	0	21.15699	0.58245	-0.01119	0.004685	-0.04809
0	0	2	0	19.9838	0.123232	0.116357	-0.02036	0.1715
0	0	2	0	20.19232	0.22652	0.087574	-0.01301	0.328692
0	0	2	0	20.42143	0.302673	0.074284	-0.00361	0.173408
0	0	1	0	21.16986	0.138504	0.156907	0.019414	0.3226
0	0	1	0	21.47199	0.263906	0.142754	0.029741	0.315826
0	0	1	0	21.46738	0.247066	0.059357	0.013762	-0.27655
0	0	1	1	20.51549	0.18194	0.126722	0.062107	0.2048
0	0	1	1	20.61376	0.172854	0.12275	0.0355	0.153271
0	0	1	1	21.03474	0.189138	0.162865	0.027185	0.73078
0	0	2	1	22.20053	0.228102	0.179008	0.088172	0.3114

0	0	2	1	22.49607	0.325917	0.184384	0.041893	0.279731
0	0	2	1	22.50195	0.199029	0.219485	0.042856	0.11691
0	0	1	0	21.04512	0.26195	0.067388	0.047403	-0.0689
0	0	1	0	21.04698	0.276874	0.006654	0.003262	-0.02986
1	1	1	1	22.32194	0.355242	0.067273	0.030902	0.0836
1	1	1	1	22.26524	0.319224	0.016534	0.017707	-0.03979
0	0	1	1	20.56825	0.273584	0.090224	0.044533	0.0766
0	0	1	1	20.70745	0.328884	0.086197	0.024231	0.018403
0	0	1	1	20.8036	0.345952	0.082233	0.019438	0.113903
1	1	1	1	20.38696	0.266969	0.123424	0.070604	0.1591
0	0	1	1	20.72897	0.36484	0.113264	0.033991	0.283569
0	0	1	1	21.00423	0.441876	0.116985	0.033801	0.49458
1	1	1	1	21.24655	0.17252	0.078123	0.047746	0.1287
1	1	1	1	21.32216	0.173654	0.067812	0.015874	0.126203
1	1	1	1	21.31961	0.196257	0.086715	0.013421	0.227465
0	0	2	0.5	21.2413	0.173042	0.160158	0.062675	0.3203
0	0	2	0.5	21.55565	0.305401	0.15057	0.034698	0.40278
0	0	2	1	21.09841	0.492875	0.051707	0.009379	0.023157
1	1	2	1	21.15481	0.493329	0.038597	0.01322	0.14277
0	0	2	1	20.00844	0.089987	0.058221	0.012434	0.010476
0	0	2	1	19.9727	0.107089	0.058032	0.008384	0.030742
0	0	1	1	20.32992	0.16002	0.124211	0.098382	0.1733
0	0	1	1	20.43281	0.163227	0.10165	0.019099	0.218472
0	0	1	1	20.54852	0.184643	0.117087	0.036377	0.180018
0	0	2	1	21.8499	0.616171	0.016975	0.009854	0.169254
0	0	2	1	21.50431	0.159725	0.117413	0.085637	0.5099
0	0	2	1	21.64496	0.21634	0.09401	0.033017	0.891068
0	0	2	1	21.70614	0.307036	0.084752	0.026473	-0.03243
0	0	1	1	20.44504	0.167006	0.119547	0.163541	-0.0374
0	0	2	0.5	20.39465	0.144341	0.053827	0.017647	-0.20184
0	0	2	0.5	20.32344	0.096106	0.006153	-0.01149	-0.64341
0	0	3	0.666667	21.83403	0.377924	0.109119	0.090008	0.0164
0	0	3	0.666667	21.79583	0.33275	0.078722	0.02783	-0.08239
0	0	3	0.666667	21.85895	0.362701	0.04934	0.017698	0.029073
0	0	1	1	20.43732	0.145636	0.107596	0.107596	-0.053
0	0	1	1	20.52288	0.129726	0.096063	0.014035	-0.0312
0	0	1	1	20.60874	0.135254	0.084849	0.013214	0.079095
0	0	2	1	21.98691	0.373243	0.105656	0.105656	0.2433
0	0	2	1	22.19644	0.415685	0.098805	0.017065	0.110221
0	0	2	1	22.36783	0.435805	0.098255	0.017172	0.185657
1	1	2	0	22.08774	0.327917	0.064295	0.0137	0.1631
1	1	2	0	22.32006	0.409241	0.05746	0.013291	0.150183
1	1	2	0	21.16384	0.069764	0.105583	0.070696	0.2342
1	1	2	0	21.23048	0.075425	0.095561	0.025022	-0.024
0	0	2	1	20.8303	0.327668	0.132771	0.132771	0.1962
0	0	2	1	20.99202	0.292874	0.135362	0.052669	0.123203
0	0	1	1	20.74958	0.241158	0.114651	0.114651	0.1482
0	0	1	1	20.78909	0.265695	0.019289	0.033061	-0.25551
0	0	2	0.5	20.89876	0.49085	0.133087	0.133087	0.5457
0	0	2	0.5	21.3542	0.373155	0.09048	0.003158	0.30406
0	0	1	0	20.63696	0.135386	0.096403	0.027962	-0.1256
1	1	3	0.666667	20.94384	0.245258	0.100784	0.049607	-0.0092
0	0	1	0	21.22676	0.189772	0.109769	0.055576	0.1433
0	0	2	1	21.55053	0.282807	0.111543	0.111543	0.255
0	0	1	0	21.91015	0.614091	0.069401	0.069401	0.393
0	0	2	1	20.56865	0.194991	0.07091	0.070863	0.4175
0	0	2	1	20.69588	0.236906	0.073181	0.015113	0.182934
0	0	2	1	20.80781	0.238476	0.091048	0.019139	0.32527

0	0	2	1	20.80795	0.219391	0.046176	0.015207	-0.22587
0	0	2	1	20.89655	0.246462	0.04859	0.0211	0.173207
0	0	2	1	22.0514	0.20033	0.037399	0.010627	1.008379
0	0	2	1	22.0433	0.1866	0.020527	0.005117	0.002035
0	0	2	1	22.06519	0.185856	0.031291	0.006653	0.091325
0	0	2	1	22.07222	0.185745	0.017932	-0.00451	-0.02168
1	3	2	1	21.73184	0.276513	0.010128	0.008574	-0.10822
1	1	1	1	20.41823	0.05577	0.093714	0.093714	0.2994
1	1	1	1	20.46799	0.062983	0.094454	-7.9E-05	0.349496
1	1	1	1	20.56179	0.07107	0.126813	0.001973	0.408059
1	1	1	1	20.68117	0.107086	0.140163	0.008468	0.230219
1	1	1	1	20.86469	0.143946	0.14992	0.018755	0.260487
1	1	1	1	21.18546	0.185966	0.108764	0.011961	0.191937
1	1	1	1	21.35902	0.227119	0.114709	0.006628	0.390683
0	0	2	1	21.86193	0.212056	0.051376	-0.00164	0.282975
0	0	1	1	21.9807	0.278296	0.055896	0.005624	0.28202
1	1	1	1	22.21263	0.276449	0.122662	0.016574	0.187392
1	1	1	0	21.70079	0.456035	0.04151	0.007027	0.157969
1	1	1	1	19.92766	0.039006	0.092033	0.128457	0.2234
1	1	1	1	19.97906	0.01576	0.093979	0.024829	-0.16032
0	0	1	1	20.07825	0.063253	0.088868	0.020512	0.080472
0	0	1	1	20.18472	0.10219	0.116556	0.022949	0.392865
1	1	1	1	20.2461	0.098561	0.117131	0.023679	0.210368
1	1	1	1	20.62566	0.265348	0.119487	0.026098	0.326777
1	1	1	1	20.77208	0.111796	0.047351	0.028437	0.116938
1	1	1	1	20.67837	0.056896	0.02246	0.022742	0.015118
1	1	1	1	20.85507	0.062802	0.122515	0.030739	0.047463
0	0	1	1	20.97691	0.064349	0.126098	0.028415	0.163654
1	1	2	0	20.4043	0.025415	0.061322	0.065955	0.2007
0	0	2	0	20.47169	0.051418	0.0419	0.008226	0.262248
0	0	2	0	20.54896	0.07226	0.062033	0.00915	0.496464
0	0	2	0	20.70547	0.113922	0.108356	0.019961	0.502926
0	0	2	0	21.05354	0.181921	0.178171	0.023615	0.47888
0	0	2	0	22.88229	0.150789	0.141784	0.038443	0.516726
0	0	2	0	23.05178	0.223136	0.07792	0.022492	0.208285
0	0	1	1	20.35096	0.063642	0.093095	0.075855	0.3272
0	0	1	1	20.35591	0.051499	0.057078	0.011955	-0.17445
0	0	1	1	20.46402	0.097326	0.045232	0.004842	0.380977
0	0	1	1	20.51474	0.110568	0.049457	0.000188	0.248191
0	0	1	1	20.51576	0.087203	0.038946	0.005565	-0.08099
0	0	1	1	20.56557	0.088027	0.050819	0.001807	0.096897
0	0	1	1	21.14376	0.094254	0.048371	0.001206	0.2037
0	0	1	1	21.1921	0.078578	0.069221	0.006213	0.324954
0	0	1	1	21.27766	0.102017	0.063211	0.006673	0.125426
1	1	2	0.5	20.70081	0.23454	0.060153	0.005508	0.116108
1	1	2	0.5	20.7403	0.253302	0.072019	-9E-06	0.195463
1	1	2	0.5	20.8602	0.221538	0.131753	0.017614	0.169385
1	1	1	1	20.18358	0.347957	0.059366	0.059366	0.2739
1	1	1	1	20.48041	0.477062	0.056906	0.009166	0.213691
1	1	1	1	20.83555	0.619069	0.049747	0.011473	0.221488
1	1	1	1	20.98748	0.648548	0.05193	0.003172	0.427257
1	1	1	1	21.07544	0.663526	0.043364	0.010431	0.265251
1	1	1	1	21.15098	0.687484	0.046645	0.009697	0.093706
1	1	1	1	21.25101	0.679858	0.078834	0.009886	0.470125
1	1	1	1	21.63399	0.44609	0.042299	0.013417	-0.11284
1	1	1	1	21.80604	0.507898	0.052761	0.013927	0.247988
1	1	1	1	22.06374	0.593344	0.056564	0.021896	0.385516
1	1	1	1	22.08812	0.58977	0.063414	0.019713	0.217052

0	0	2	0.5	23.63351	0.482152	-0.02981	0.025166	-0.0334
1	3	1	0	19.20553	0.314092	-0.06617	-0.01274	-0.42592
0	0	2	0.5	21.09292	0.149353	0.07298	0.010822	0.526261
1	1	2	0.5	21.30087	0.224713	0.054688	0.015569	0.86098
0	0	2	0.5	20.81681	0.399745	0.017904	-0.00462	-0.37168
0	0	1	0	20.59488	0.060187	0.047207	0.015136	-0.23818
0	0	1	0	20.60694	0.065469	0.043079	0.011148	0.106223
0	0	1	0	20.8561	0.225046	0.02127	0.008494	0.152125
0	0	1	0	20.97588	0.30059	0.018	0.016276	0.403675
0	0	1	0	21.88742	0.413738	0.042208	0.001325	1.750551
1	1	1	0	22.08612	0.454352	0.097996	0.035404	1.47326
1	1	1	0	22.58663	0.275168	0.024796	0.027031	0.112861
1	1	1	0	22.67753	0.430234	-0.04466	0.00476	0.097461
0	0	2	1	21.01132	0.204121	0.091197	0.026981	0.314013
0	0	2	1	21.29479	0.145789	0.055161	0.026556	0.155842
0	0	2	1	21.32551	0.134061	0.062441	0.024013	0.160359
0	0	2	1	21.53138	0.162213	0.057736	0.027804	0.339045
0	0	2	1	21.60226	0.267744	0.056289	0.029069	0.148104
0	0	2	1	21.7109	0.375107	0.071372	0.024706	0.080642
0	0	2	1	20.4336	0.069243	0.083435	0.014624	0.390987
0	0	2	1	20.66473	0.189944	0.096457	0.02052	0.369228
0	0	2	1	21.15455	0.39586	0.089426	0.019821	1.517206
0	0	2	1	21.32461	0.390721	0.096409	0.016402	0.984182
0	0	2	1	21.76802	0.319853	0.120112	0.009711	0.134512
0	0	2	1	21.91752	0.36619	0.15292	0.028747	-0.33564
0	0	2	1	22.35675	0.529919	0.104564	0.056935	0.430818
1	1	2	1	22.51163	0.562711	0.063054	0.02543	0.151348
1	1	2	1	22.5076	0.535756	0.062775	0.012564	0.02353
1	1	2	1	22.47073	0.486492	0.077049	0.012266	-0.0801
1	2	1	1	20.64393	0.081644	0.078315	0.009793	0.2242
1	2	1	1	20.78196	0.129163	0.05977	0.006929	0.216046
1	2	1	1	20.83901	0.114679	0.081633	0.008332	0.321939
1	2	1	1	20.93855	0.107303	0.100838	0.010518	0.103158
1	2	1	1	21.07114	0.134057	0.113653	0.012822	0.032125
1	2	1	1	21.19425	0.090979	0.096464	0.014802	-0.07291
1	2	1	1	21.29048	0.118178	0.083832	0.018153	0.074302
0	0	1	1	21.31359	0.104109	0.048558	0.012449	-0.12193
0	0	1	1	21.36174	0.10591	0.055111	0.010002	0.12586
0	0	1	1	21.50652	0.144478	0.102369	0.011368	0.437017
0	0	1	1	20.14058	0.175756	0.054312	0.03105	-0.1953
0	0	1	1	20.28665	0.256039	0.035663	0.01243	0.062441
0	0	1	1	20.35894	0.293579	0.022452	0.010469	0.004846
1	1	1	1	20.35843	0.303213	0.039188	0.016301	0.323947
0	0	1	1	20.30355	0.262997	0.030895	0.024143	0.081325
0	0	1	1	20.35474	0.292985	0.023105	0.0064	0.027506
0	0	1	1	20.52606	0.382332	0.042839	0.016744	0.577658
1	1	1	1	20.62149	0.406211	0.038475	0.010576	-0.22766
1	1	2	1	20.19326	0.351107	0.060529	0.003072	0.074273
0	0	2	0.5	20.5406	0.017699	0.053701	0.002248	0.0171
0	0	2	0.5	20.56814	0.031995	0.045604	-0.00076	0.222276
0	0	2	0.5	20.67982	0.049006	0.071226	-0.00109	0.577796
0	0	2	0.5	20.77354	0.046227	0.086319	0.005578	0.279724
0	0	2	0.5	20.78466	0.046125	0.024719	0.009238	-0.13458
1	1	2	0.5	21.04713	0.170999	0.055412	-0.01077	0.031778
1	1	2	0.5	21.18763	0.240708	0.014721	-0.00584	0.332998
0	0	2	0	20.88934	0.087562	0.008264	-0.00355	-0.20295
0	0	2	0	20.83678	0.049093	-0.01143	-0.00502	-0.23005
0	0	2	0	20.85152	0.067521	-0.03236	-0.0043	-0.15481

0	0	3	0.333333	20.82109	0.084701	0.025185	0.038184	0.471481
0	0	1	1	21.08123	0.183201	0.058523	0.029031	0.3347
0	0	1	1	21.19759	0.244877	0.042104	0.008204	0.397676
0	0	1	1	21.44737	0.366963	0.050793	0.006534	0.512571
0	0	1	1	21.72238	0.477321	0.054907	0.013165	0.615278
0	0	1	1	22.04833	0.603031	0.030669	0.012811	0.214308
0	0	1	1	22.89491	0.431855	-0.05361	-0.01465	-0.10396
0	0	1	1	22.98947	0.468883	0.024338	0.006898	0.440058
0	0	1	1	23.13799	0.498475	0.053107	0.017436	0.250276
1	1	1	0	20.57464	0.232144	0.06083	0.061775	-0.1359
1	1	1	0	20.58236	0.237004	0.029769	0.019219	-0.03359
1	1	1	0	20.63366	0.230165	0.042999	0.016658	0.056775
1	1	1	0	20.78275	0.233093	0.076361	0.011637	0.31599
1	1	1	0	20.84843	0.237044	0.05867	0.015272	0.603707
1	1	1	0	20.90044	0.277887	0.063256	0.012957	-0.16505
1	1	1	0	21.47482	0.382908	0.034814	0.013952	0.050054
1	1	1	0	21.69324	0.474965	0.035593	0.006793	0.144002
1	1	1	0	21.60468	0.467	-0.01296	0.013731	0.360857
1	1	1	0	21.65852	0.460192	0.065579	0.015301	0.160366
1	1	2	0.5	20.88038	0.297329	0.117715	0.094319	0.4278
1	1	2	0.5	21.02992	0.327808	0.130605	0.03481	0.257487
0	0	2	0.5	21.12479	0.345709	0.09588	0.01706	0.100927
0	0	1	1	21.33482	0.455769	0.075407	0.02254	0.122453
0	0	1	1	22.01583	0.475121	0.050219	0.023422	0.24183
0	0	1	1	22.12595	0.474464	0.080607	0.022162	0.651194
1	1	1	1	22.43234	0.529447	0.083523	0.023912	0.336334
1	1	1	1	22.60622	0.567995	0.05315	0.021134	0.295255
1	1	1	1	22.74208	0.637182	0.013009	0.012516	0.299288
1	1	2	0	20.66982	0.148435	0.04178	0.010583	-0.3806
1	1	2	0	20.73388	0.17031	0.022389	0.00013	-0.31409
1	1	2	0	20.72101	0.143736	0.016392	0.003902	-0.03136
0	0	2	0	20.74614	0.133449	0.041932	0.006524	0.336428
1	1	2	0	20.76408	0.119164	0.011987	0.011109	-0.20312
1	1	2	0	21.45891	0.415453	0.059727	0.000323	4.003129
1	1	1	0	20.5153	0.019471	0.072861	0.055223	0.3014
0	0	1	0	20.50459	0.020259	0.019079	0.011407	0.012464
0	0	1	0	20.50948	0.015349	0.011129	0.004224	0.150023
0	0	1	0	20.56348	0.029448	0.045732	0.004563	0.169462
1	1	1	0	20.63308	0.054588	0.048333	0.0199	0.133812
1	1	1	0	20.66664	0.050889	0.022219	0.007128	-0.10893
1	1	1	0	20.66498	0.035071	0.02212	0.004876	-0.20301
1	1	1	0	20.80382	0.093192	0.096592	0.005454	0.618044
1	1	1	0	21.34348	0.050781	0.184532	0.068812	-0.3477
0	0	1	1	19.69552	0.068748	0.079789	0.064307	0.2554
0	0	1	1	19.78099	0.099566	0.067807	0.017034	0.16792
0	0	1	1	19.83074	0.111846	0.053592	0.015533	0.115934
0	0	1	1	19.86204	0.107851	0.05837	0.014852	-0.01624
0	0	1	1	19.97804	0.156716	0.074766	0.014449	0.209912
1	2	1	1	20.93512	0.059151	0.053211	0.031308	0.147216
1	2	1	1	20.99322	0.063609	0.069566	0.015782	0.378404
1	2	1	1	21.39552	0.335041	0.056655	0.021297	0.219567
1	2	1	1	21.55752	0.445869	0.059613	0.018313	0.429205
0	0	1	1	22.15757	0.272619	0.048791	0.017704	0.323856
1	1	1	1	20.86454	0.041325	0.088621	0.057917	0.1724
1	1	1	1	20.94439	0.04471	0.078411	0.017528	0.198071
1	1	1	1	21.30265	0.249697	0.067953	0.014996	0.38667
1	1	1	1	21.58485	0.383437	0.069793	0.01888	0.542967
1	1	1	1	21.68705	0.435571	0.038586	0.017741	0.057151

1	1	1	1	21.73442	0.476141	0.030696	0.011007	0.206307
0	0	2	1	22.63481	0.797313	0.040666	0.006489	0.452134
0	0	2	1	22.53133	0.772147	0.04157	0.008687	-0.19211
0	0	3	0.333333	22.1528	0.403362	0.03689	0.00305	-0.11312
0	0	3	0.333333	22.13419	0.499978	0.036248	0.014539	1.129846
0	0	1	1	20.28554	0.114464	0.077092	0.055272	0.1888
1	1	1	1	20.2761	0.073073	0.068788	0.013651	0.000565
1	1	1	1	20.29272	0.085623	0.031552	0.012618	-0.23161
1	1	1	1	20.34183	0.095512	0.054226	0.016398	0.325342
1	1	1	1	20.37985	0.15179	0.007773	0.002706	0.028145
0	0	1	1	20.45859	0.133832	0.009793	0.002812	0.193121
0	0	1	1	20.4488	0.102933	0.027929	0.000718	0.242505
0	0	1	1	21.70759	0.298034	0.027633	0.009875	0.410328
1	1	1	1	21.77674	0.30317	0.063539	0.013189	1.321194
1	3	1	1	22.02314	0.311074	0.045268	0.009342	0.130738
0	0	1	1	21.51593	0.030592	0.134697	0.040744	0.2206
0	0	1	1	21.60052	0.060818	0.081985	0.012761	-0.14726
0	0	1	1	21.64096	0.052139	0.088497	0.003857	0.209286
0	0	1	1	21.6741	0.076745	0.049359	0.016442	0.026109
0	0	1	1	21.72186	0.094574	0.055505	0.016848	0.026609
0	0	1	1	21.70969	0.079206	0.075734	0.018148	-0.11012
0	0	1	1	21.72002	0.069465	0.006379	0.023865	-0.37432
0	0	1	1	22.13569	0.284558	0.119971	0.024924	2.0106
0	0	2	1	20.6976	0.195474	0.039704	0.016233	0.3419
0	0	2	1	20.93983	0.340097	0.037564	0.006855	0.626138
0	0	2	1	21.05074	0.379783	0.047994	0.008291	0.462748
0	0	2	1	21.02313	0.316491	0.059969	0.010037	0.172176
0	0	2	1	21.10004	0.349037	0.028617	0.013465	-0.43192
0	0	2	1	21.31274	0.466357	0.021207	-0.00085	0.143446
0	0	2	1	21.50263	0.45898	0.021549	-0.00189	0.329636
1	1	2	1	22.60295	0.28628	0.024144	0.001134	0.782975
1	1	2	1	22.70583	0.334609	0.035103	0.005431	0.348199
1	1	1	1	22.75656	0.336704	0.034488	0.007748	0.562777
0	0	3	1	21.10448	0.107747	0.099406	0.172048	0.1276
0	0	3	1	21.19417	0.159886	0.03339	0.007812	0.083993
0	0	3	1	21.38935	0.273881	0.023485	0.005949	0.090395
0	0	3	1	21.58965	0.311395	0.025971	0.002531	0.607656
0	0	1	1	21.74249	0.381791	0.046839	0.004422	0.332575
0	0	1	1	21.85792	0.368769	0.062466	0.0076	0.11041
0	0	3	1	21.83988	0.296026	0.085023	0.014647	-0.0572
1	1	3	1	21.89192	0.298424	0.061479	0.015729	0.056041
1	1	3	1	21.96493	0.361495	0.002325	0.006126	-0.07388
1	1	3	1	22.17609	0.456626	0.04171	0.00815	0.506952
1	3	1	1	20.67345	0.234788	0.121386	0.018085	0.455623
0	0	2	0	20.91472	0.019145	0.103703	0.094693	0.6981
0	0	2	0	21.11055	0.112144	0.129354	0.022955	0.430144
0	0	2	0	21.2927	0.136939	0.155877	0.003719	0.78856
0	0	2	0	21.46774	0.135541	0.163842	0.030797	0.276808
0	0	2	0	22.3374	0.327107	0.106544	0.025179	1.081634
0	0	2	0	22.82159	0.268908	0.082494	0.024035	0.386849
0	0	2	0	23.06352	0.373328	0.05986	0.01793	0.672739
0	0	2	0	23.2521	0.441192	0.063815	0.017631	0.180117
0	0	2	0	23.27838	0.45622	0.035623	0.004168	0.105159
1	1	1	1	21.78875	0.590858	0.028316	0.006721	-0.21254
1	1	1	1	21.84358	0.607442	0.026971	0.004548	0.304244
0	0	1	1	21.80753	0.581872	0.049058	0.008103	0.138941
0	0	1	1	21.7878	0.565307	0.040976	0.004165	0.185295
1	1	1	1	20.11588	0.071407	0.071137	0.071181	0.2345

1	1	1	1	20.44783	0.284129	0.044355	0.019219	0.182136
0	0	1	1	20.41074	0.200364	0.073037	0.015594	0.443828
0	0	1	1	20.49285	0.195629	0.080923	0.019021	0.238642
0	0	1	1	20.52353	0.160266	0.066787	0.018302	-0.05223
0	0	1	1	20.57485	0.184592	0.023901	0.011196	-0.07258
0	0	1	1	20.68457	0.248776	0.040845	0.001357	0.349435
0	0	1	1	20.90468	0.4089	0.015479	0.006405	0.33442
0	0	1	1	21.18247	0.539033	0.012038	0.007133	0.390607
0	0	1	1	21.01605	0.443858	0.016881	-0.00347	-0.30944
1	1	1	1	20.82846	0.165831	0.078423	0.078423	0.6014
1	1	1	1	20.96439	0.202641	0.089039	0.014754	0.360844
1	1	1	1	21.11542	0.218312	0.093702	0.016623	0.21242
1	1	1	1	21.29537	0.25022	0.104669	0.016646	0.281982
1	1	1	1	21.44209	0.263692	0.108811	0.01792	0.168817
1	1	1	1	22.40279	0.322853	0.061212	0.016489	0.239815
1	1	1	1	22.71182	0.432867	0.088735	0.011272	0.432005
1	1	1	1	23.01227	0.522116	0.081591	0.017434	0.367236
1	1	1	1	21.28496	0.07372	0.060667	0.055913	0.6858
1	1	1	1	21.36205	0.07288	0.096895	0.024073	0.901185
1	1	1	1	21.46745	0.084388	0.140463	0.063386	0.621202
1	1	1	1	21.62153	0.090639	0.190941	0.059122	0.389569
1	1	1	1	21.71695	0.09384	0.203012	0.075395	0.150271
1	1	1	1	22.3136	0.071666	0.140398	0.047149	0.329073
1	1	1	1	22.39631	0.117233	0.114596	0.04828	0.019006
1	1	1	1	22.53378	0.160568	0.137198	0.061484	0.347182
1	1	1	1	23.00467	0.296462	0.11315	0.076608	0.398606
1	1	1	1	22.84321	0.288009	-0.04972	0.061922	0.209393
1	1	1	0	20.09617	0.059532	0.071789	0.071789	0.6259
1	1	1	0	20.17427	0.085343	0.063841	0.007884	0.298198
1	1	1	0	20.21374	0.11178	0.037516	0.004875	0.008308
1	1	1	0	20.2748	0.145368	0.033368	0.0078	0.17163
1	1	1	0	21.431	0.125632	0.06811	0.006841	0.572674
1	1	1	0	21.45841	0.070324	0.102471	0.028979	0.055311
1	1	2	0	22.32161	0.102737	0.059614	0.004692	0.432968
1	1	2	0	22.2864	0.104601	0.033428	0.012829	0.200123
1	1	3	0	22.09756	0.172211	0.01299	0.013249	0.101181
0	0	2	1	20.22526	0.631637	0.137614	0.137614	0.7211
0	0	2	1	20.97224	0.207788	0.09399	0.023495	0.258716
0	0	2	1	20.98651	0.203852	0.040349	0.018395	-0.01005
0	0	2	1	21.10746	0.275864	0.026144	0.010614	-0.00106
0	0	2	1	21.03275	0.203871	0.027702	0.014434	0.064218
0	0	2	1	21.15198	0.266146	0.042551	0.017626	0.21827
0	0	2	1	21.26215	0.285469	0.082614	0.02773	0.124687
0	0	2	1	21.49565	0.38475	0.08022	0.038156	0.20094
0	0	2	1	21.26014	0.413882	0.058356	0.02595	-0.09398
0	0	1	1	20.59845	0.154538	0.014285	0.007142	0.199416
0	0	1	1	20.58639	0.156766	0.017309	0.005106	0.107773
0	0	1	1	20.63449	0.155384	0.034223	0.004893	0.155845
0	0	1	1	20.93615	0.120751	0.010821	0.004534	-0.0599
0	0	1	1	21.12846	0.238085	0.018188	0.009722	0.477188
0	0	1	1	21.02774	0.175604	0.030457	0.009403	0.145795
0	0	1	1	21.03441	0.226024	-0.03627	0.007433	0.13811
0	0	1	1	21.0564	0.324715	0.031181	0.015881	0.318157
0	0	1	0	21.64689	0.174567	0.083936	0.020647	0.350066
0	0	1	0	21.89001	0.306595	0.072636	0.019764	0.266608
1	1	1	0	22.0526	0.362728	0.071248	0.019867	0.242454
0	0	1	0	22.1507	0.357839	0.084927	0.022459	0.232659
0	0	1	0	22.12499	0.274288	0.094091	0.027061	0.23585

0	0	1	0	22.15788	0.285421	0.061461	0.024327	-0.14639
0	0	1	0	22.19718	0.293182	0.038879	0.017779	0.299015
0	0	1	0	22.26829	0.335085	0.017561	0.00878	0.111279
0	0	1	0	22.37357	0.377454	0.03519	0.004005	0.181124
0	0	2	0.5	19.79797	0.483118	0.154681	0.152735	0.3749
0	0	2	0.5	20.4821	0.12622	0.079633	0.012072	0.126154
0	0	2	0.5	20.53075	0.13977	0.028254	0.012883	-0.02703
0	0	2	0.5	20.83635	0.345953	0.023062	0.013011	0.313955
0	0	2	0.5	21.1173	0.39231	0.072796	0.008817	0.34398
0	0	2	0.5	21.17778	0.396675	0.046475	0.0075	0.04618
0	0	2	0.5	20.90631	0.335094	0.049424	0.010159	-0.30378
0	0	2	0.5	20.61634	0.077933	0.073432	0.091755	-0.35548
0	0	2	0.5	21.06703	0.299574	0.055041	-0.00335	-0.08119
1	1	2	0.5	21.13709	0.338301	0.032321	0.013038	0.624769
1	1	3	0.666667	19.99154	0.681495	0.12062	0.12062	0.3039
1	1	3	0.666667	20.53122	0.372002	0.058907	0.020952	0.375766
1	1	3	0.666667	20.73687	0.464279	0.065723	0.016115	0.425723
1	1	3	0.666667	21.12907	0.626684	0.046692	0.01626	0.397051
1	1	3	0.666667	21.32078	0.68893	0.035428	0.009863	0.167579
0	0	3	0.666667	21.43252	0.70563	0.045513	0.010433	0.159611
0	0	3	0.666667	21.31684	0.647774	0.053652	0.01425	-1.6E-05
0	0	1	1	20.55378	0.123362	0.075649	0.010435	0.257527
1	1	1	1	20.66236	0.120864	0.076113	0.012804	0.231767
1	1	1	1	20.85742	0.116968	0.099503	0.01005	0.411336
1	1	1	1	20.99097	0.147105	0.087846	0.00818	0.276703
1	1	1	1	21.36694	0.126345	-0.00318	0.001395	-0.07165
1	1	1	1	21.4448	0.18654	0.007438	-0.00444	0.200093
1	1	1	1	21.63728	0.232952	0.044103	0.00034	0.33325
0	0	1	1	21.74895	0.243108	0.060434	0.009458	0.262558
0	0	1	1	21.87853	0.35043	-0.04041	0.012318	0.256431
1	1	2	1	19.97748	0.249411	0.208008	0.208008	0.1981
1	1	2	1	20.95005	0.056418	0.064278	0.013542	0.086407
1	1	2	1	21.0124	0.065172	0.06877	0.007859	0.300998
1	1	2	1	21.052	0.048173	0.070979	0.010897	0.186658
1	1	2	1	21.29318	0.197377	0.072541	0.018142	0.335508
1	1	2	1	21.6955	0.42303	0.050917	0.020929	0.171743
1	1	2	1	22.06949	0.315054	0.029972	0.005734	0.265731
1	1	2	1	22.61352	0.264448	0.048647	0.009404	0.741105
1	1	2	1	22.74343	0.31881	0.054458	0.018305	0.223314
1	1	2	1	22.85031	0.374174	0.030598	0.014659	0.057165
1	1	2	1	19.66956	0.405779	0.149433	0.149433	0.2197
1	1	2	1	20.55355	0.189583	0.064465	0.01037	0.063476
1	1	2	1	20.5773	0.18314	0.078613	0.015163	0.230666
1	1	2	1	20.66341	0.179829	0.096498	0.018912	0.220085
1	1	2	1	20.89006	0.262166	0.105071	0.022859	0.227724
1	1	2	1	21.33875	0.224227	0.060936	0.024232	0.302822
1	1	2	1	21.43516	0.277743	0.055015	0.0162	0.252186
1	1	2	1	21.48986	0.307585	0.035458	0.008289	-0.05491
1	1	1	1	21.47175	0.343694	0.027467	0.007623	-0.08017
1	1	1	1	21.44142	0.31349	0.022777	0.007632	0.247721
1	1	1	0	20.84319	0.122555	0.094766	0.020294	0.593864
1	1	1	0	20.95926	0.088963	0.124691	0.020975	0.542138
1	1	1	0	21.37473	0.250662	0.111717	0.021132	0.285996
1	1	1	0	21.57355	0.261825	0.126902	0.025596	0.320371
1	1	1	0	22.51852	0.280169	0.102494	0.025738	0.791653
1	1	1	0	23.26266	0.286517	0.094283	0.014163	0.48292
1	1	1	0	23.44135	0.351469	0.082455	0.011748	0.331801
1	1	1	0	23.49439	0.385881	0.021763	0.016182	0.151715

1	1	2	1	21.75069	0.585225	0.096641	0.095841	0.0115
1	1	2	1	22.49754	0.388684	0.041841	0.015647	0.096368
1	1	2	1	22.56611	0.416562	0.034241	0.013536	0.121807
1	1	2	1	22.63835	0.449169	0.024613	0.008964	0.191559
1	1	2	1	22.65341	0.470371	0.033251	0.004907	0.244358
1	1	2	1	22.78896	0.510156	0.039975	0.007069	0.139623
1	1	2	1	22.93157	0.41757	0.042853	0.008171	0.037022
1	1	2	1	23.03466	0.463628	0.044063	0.005912	0.305033
1	1	2	1	23.0618	0.48555	0.048089	0.008827	0.114911
0	0	2	1	23.23248	0.552935	0.046231	0.009636	0.139299
0	0	2	0.5	20.8035	0.296761	0.029405	0.013083	0.190389
0	0	2	0.5	20.92159	0.344346	0.042174	0.001268	0.65331
0	0	2	0.5	21.04357	0.379845	0.04058	0.008722	0.12593
0	0	2	0.5	21.16582	0.415658	0.036625	0.004363	0.408665
0	0	2	0.5	21.56082	0.423356	0.052869	0.003753	-0.14184
0	0	2	0.5	21.82051	0.486124	0.06424	0.001677	0.986171
0	0	2	0.5	21.88402	0.471144	0.049471	0.007249	-0.19988
1	3	2	0.5	21.92647	0.538614	-0.04996	0.005884	-0.22609
1	3	2	0.5	21.73491	0.413976	0.027795	0.004821	-0.29385
0	0	2	0.5	20.52589	0.179309	0.073203	-0.00183	0.428305
0	0	2	0.5	20.64764	0.157354	0.07945	0.000492	0.303453
0	0	2	0.5	20.7677	0.186065	0.063478	-0.0063	0.115475
0	0	2	0.5	20.99668	0.271206	0.09902	0.000436	0.544761
1	1	2	0.5	21.51284	0.232075	0.075386	-0.00017	0.265406
1	1	2	0.5	21.68901	0.279485	0.074236	0.000044	0.3076
1	1	2	0.5	21.85851	0.244715	0.091467	-0.00064	0.339442
1	1	2	0.5	21.95994	0.2529	0.092414	0.000465	0.197468
0	0	1	1	20.77139	0.176189	0.050296	0.011272	0.2577
0	0	1	1	20.84739	0.158537	0.051852	0.009917	0.343895
0	0	1	1	20.88831	0.172131	0.017928	0.008994	-0.21498
0	0	1	1	21.40238	0.241932	0.055032	0.010259	1.337681
0	0	1	1	21.74484	0.420235	0.058038	0.007155	0.476983
1	1	1	1	22.22542	0.367879	0.025847	0.011333	-0.19506
1	1	1	1	22.53803	0.390372	0.031605	0.007431	0.832984
1	1	1	1	22.69911	0.483676	0.04435	0.010551	0.456407
1	1	1	1	22.81571	0.505312	0.05166	0.012846	0.322448
0	0	1	1	20.81097	0.022006	0.07101	0.021301	0.1447
0	0	1	1	20.86607	0.029514	0.066312	0.017257	0.155046
0	0	1	1	20.94433	0.036843	0.073279	0.016822	0.169849
0	0	1	1	20.94063	0.020836	0.010463	0.010769	-0.53968
0	0	1	1	20.95323	0.072853	-0.05439	-0.01029	-0.4219
0	0	1	1	20.96899	0.07869	-0.00702	0.004772	0.793066
0	0	1	1	20.96248	0.061808	-0.01133	-0.00888	0.021389
0	0	1	1	21.05977	0.098975	0.050514	0.008678	0.329449
0	0	1	1	21.29392	0.318036	0.018662	0.005905	0.598868
1	1	1	1	21.32052	0.068619	0.046931	0.024177	-0.2273
1	1	1	1	21.3137	0.046656	0.038607	0.01225	0.171439
1	1	1	1	21.34566	0.07431	0.010153	0.005665	-0.14818
1	1	1	1	21.32878	0.068052	0.018369	0.007046	0.25376
1	1	1	1	21.63116	0.13832	0.030527	0.003899	0.578564
1	1	1	1	22.20433	0.09869	0.060857	0.014959	0.831696
1	1	1	1	22.26844	0.110526	0.071099	0.023418	0.612012
1	1	1	1	22.18016	0.139453	-0.07721	0.021601	0.276682
1	1	1	1	22.27845	0.157643	0.065499	0.01913	0.041751
0	0	1	0	22.3955	0.251889	0.032578	0.008761	-0.01851
0	0	1	0	22.49561	0.353343	0.025214	0.010009	0.160544
0	0	2	1	20.67936	0.124313	0.071584	0.013464	0.1785
0	0	2	1	20.89152	0.234488	0.08819	0.015299	0.357089

0	0	2	1	21.0825	0.292448	0.089466	0.018074	0.527784
0	0	2	1	21.152	0.258058	0.117621	0.022178	0.209741
0	0	2	1	21.54194	0.332806	0.121326	0.028214	0.362617
0	0	2	1	21.83057	0.234999	0.105484	0.028961	0.166125
0	0	1	1	21.85595	0.209759	0.085537	0.024546	0.146653
0	0	1	1	21.86632	0.19438	0.092239	0.021339	0.08423
0	0	1	1	21.88193	0.142466	0.102592	0.022717	0.005353
1	1	1	1	20.71216	0.082188	0.082726	0.014415	0.437
1	1	1	1	20.84214	0.096001	0.096043	0.013359	0.440361
0	0	1	1	21.00204	0.170993	0.080183	0.017404	0.652406
0	0	1	1	21.16523	0.278211	0.044423	0.019696	0.52342
0	0	1	1	21.45419	0.406123	0.041651	0.005595	0.309789
0	0	2	1	20.39878	0.071319	0.100832	0.001256	0.9185
0	0	2	1	20.65614	0.146508	0.166024	0.005722	1.0209
0	0	2	1	21.32358	0.1286	0.154746	0.0028	-0.2176
0	0	2	1	21.69538	0.291012	0.123175	0.005546	0.048772
0	0	2	1	21.69683	0.312909	0.032316	0.006298	-0.26552
0	0	2	1	21.57822	0.309725	-0.05496	0.004286	-0.08839
0	0	2	1	21.50227	0.250293	0.030201	0.005526	0.074292
0	0	2	1	20.86251	0.041171	0.092267	0.0474	0.6053
1	1	2	1	21.08301	0.134091	0.115861	0.02917	1.702182
1	1	2	1	21.174	0.162776	0.070927	0.036098	0.605118
1	1	2	1	21.31765	0.164213	0.116705	0.023298	0.224391
1	1	2	1	21.45123	0.186908	0.108228	0.031507	0.141709
1	1	2	1	21.57793	0.18392	0.123144	0.031384	0.12455
1	1	2	1	21.68969	0.159459	0.112464	0.031356	-0.011
1	1	2	1	21.74289	0.113378	0.0525	0.024853	-0.4192
1	1	2	1	21.34514	0.121983	0.010299	0.011974	-0.17937
0	0	1	1	20.57185	0.26887	0.070793	0.035971	0.2171
0	0	1	1	20.77156	0.352192	0.065581	0.013132	0.111677
1	1	1	1	20.89629	0.416348	0.051248	0.006462	-0.31626
1	1	1	1	21.08583	0.437896	0.112835	0.010045	2.96564
1	1	1	1	21.48255	0.458394	0.19549	0.055311	0.735072
1	2	2	1	21.74401	0.419329	0.112344	0.050513	0.031035
0	0	1	1	21.29996	0.388231	0.063056	0.035135	0.3238
0	0	1	1	21.52051	0.469787	0.078973	0.021505	0.48175
0	0	1	1	21.7078	0.499781	0.08386	0.019972	0.361456
0	0	1	1	21.90727	0.583018	0.03826	0.007799	-0.11501
0	0	1	1	22.06387	0.642423	0.02098	0.000654	-0.01034
0	0	1	1	22.04231	0.489065	0.035654	0.004171	0.325844
0	0	1	1	22.05619	0.473772	0.050133	0.005192	0.161262
0	0	1	1	22.02895	0.444981	0.053704	0.006999	0.102721
0	0	1	1	22.01851	0.418245	0.064183	0.008514	0.137819
0	0	1	0	20.49437	0.134244	0.075519	0.042123	0.2499
0	0	1	0	20.67834	0.167796	0.090689	0.009893	0.382455
0	0	1	0	20.92824	0.281387	0.089803	0.013517	0.26324
0	0	1	0	21.25687	0.425894	0.103861	0.012065	0.284061
0	0	1	0	21.83883	0.663532	0.035566	0.015092	-0.08856
0	0	1	0	22.19028	0.742069	0.036047	0.015185	0.41762
0	0	1	0	22.26696	0.839223	-0.04355	-0.00873	-0.597
0	0	2	1	21.02514	0.265233	0.108393	0.05182	0.2087
0	0	2	1	21.0516	0.229098	0.069475	0.025935	-0.07674
0	0	2	1	21.17837	0.28049	0.063115	0.013906	0.25196
0	0	2	1	21.23638	0.275803	0.073137	0.019606	0.231466
0	0	2	1	21.56343	0.198743	0.039058	0.014939	-0.07541
0	0	2	1	21.73018	0.258778	0.02053	0.005221	-0.01325
0	0	2	1	21.81263	0.302913	0.044424	0.007177	0.861064
0	0	2	1	22.03198	0.399491	0.052545	0.011532	0.294288

0	0	2	1	21.99606	0.414304	-0.02554	0.01143	-0.00236
0	0	1	1	19.5891	0.152636	0.054009	0.03506	0.2283
0	0	1	1	19.76873	0.257807	0.036718	0.020104	0.020351
0	0	1	1	19.83074	0.283091	0.031402	0.011653	0.106785
0	0	1	1	19.86017	0.282993	0.024304	0.009636	0.215364
0	0	1	1	19.96918	0.275404	-0.02693	0.005528	-0.05002
0	0	1	1	19.95878	0.264359	0.017756	0.009231	0.024199
0	0	1	1	19.68849	0.274891	0.023174	0.01931	0.150518
0	0	1	1	19.76264	0.287359	0.03849	0.014875	0.030611
0	0	2	1	20.46118	0.11113	0.071235	0.050065	-0.0015
0	0	2	1	20.52122	0.135062	0.068713	0.016115	-0.01729
0	0	2	1	20.70271	0.231992	0.084633	0.018592	0.245465
0	0	2	1	20.74693	0.217301	0.093931	0.018465	0.095076
1	1	2	1	20.79233	0.202463	0.063889	0.017859	0.013183
1	3	2	1	20.86922	0.221156	0.065083	0.016337	0.124998
1	3	2	1	20.91451	0.244379	0.038755	0.010341	0.009437
1	1	2	0	22.24594	0.30522	0.100798	0.020468	0.117703
1	1	2	0	22.25069	0.285112	0.079351	0.019265	-0.05403
1	1	1	1	21.05845	0.31423	0.060929	0.040706	0.0382
1	1	1	1	21.10841	0.336271	0.039428	0.002539	0.013839
1	1	1	1	21.17803	0.351636	0.061584	0.015169	0.136841
1	1	1	1	21.30284	0.374511	0.075647	0.019029	0.156394
1	1	1	1	21.41456	0.34361	0.084718	0.025943	0.121552
1	1	1	1	21.53725	0.333504	0.073935	0.024751	0.205187
1	1	1	1	22.3133	0.197034	0.073138	0.01451	0.962874
1	1	1	1	22.38844	0.226715	0.069256	0.019736	0.078884
1	1	1	1	22.44467	0.269572	0.057456	0.023308	-0.07086
0	0	2	1	19.8132	0.115659	0.108892	0.055666	0.1426
1	1	2	1	19.88633	0.132112	0.083726	0.016386	-0.04567
1	1	2	1	20.67407	0.164421	0.046269	0.0159	0.456764
1	1	2	1	20.76332	0.176491	0.073244	0.010343	0.801395
1	1	2	1	20.87165	0.218024	0.042014	0.007956	-0.02044
1	1	2	1	21.1086	0.145854	0.041142	0.012855	0.123087
1	1	2	1	21.1379	0.138641	0.05404	0.014156	0.14114
1	1	2	1	21.15056	0.167743	-0.00081	0.012338	0.185039
1	1	2	1	21.34488	0.186049	0.14131	0.005489	0.145379
0	0	1	1	20.02222	0.127546	0.08923	0.038722	0.2523
0	0	1	1	20.11076	0.16215	0.062368	0.034036	0.144035
0	0	1	1	20.16556	0.129884	0.070494	0.014335	0.146115
0	0	1	1	20.24448	0.14192	0.084885	0.016341	0.1297
0	0	1	1	20.50886	0.23098	0.085904	0.014947	0.188151
0	0	1	1	21.21594	0.123126	0.040769	0.010937	0.382969
1	1	1	1	21.29897	0.318276	0.030823	0.00946	0.472544
1	1	3	0.666667	19.74941	0.216175	0.084846	0.042942	0.0789
1	1	3	0.666667	20.00019	0.332258	0.092774	0.01711	0.231393
1	1	3	0.666667	20.06863	0.323611	0.114816	0.040783	0.495107
1	1	3	0.666667	20.08946	0.358132	0.026524	0.010998	-0.34298
1	1	3	0.666667	20.47486	0.094222	0.018932	0.009906	0.162763
1	1	3	0.666667	20.5407	0.13716	0.026626	0.00458	0.084538
1	1	3	0.666667	20.69439	0.248864	0.027533	0.008019	0.125241
1	1	3	0.666667	20.90454	0.373611	0.034858	0.0086	0.270892
1	1	3	0.666667	20.99661	0.245356	0.030628	0.006838	0.086298
1	3	1	1	21.73809	0.164942	0.077681	0.024626	-0.04169
1	1	1	1	21.61242	0.570825	0.026244	0.021187	-0.01447
0	0	2	1	20.20454	0.041108	0.09003	0.059864	0.0546
0	0	2	1	20.27292	0.107739	0.029179	0.009441	0.183102
0	0	2	1	20.32283	0.125081	0.03175	0.011696	0.080421
0	0	2	1	20.49857	0.153819	0.034288	0.011138	0.071967

0	0	2	1	21.38158	0.235012	0.026691	0.011663	0.510692
0	0	2	1	21.74753	0.185809	0.041554	0.005643	0.398579
0	0	2	1	21.81844	0.205747	0.032333	0.007784	1.029088
0	0	1	0	20.12555	0.169568	0.07978	0.064888	0.206
0	0	1	0	20.17375	0.149583	0.065391	0.010092	0.266376
0	0	1	0	20.36443	0.263052	0.029465	0.008537	0.402322
0	0	1	0	21.08508	0.423664	0.023554	-0.01566	0.265716
0	0	1	0	21.18157	0.441469	0.025754	0.000029	0.793258
0	0	1	0	21.50276	0.577098	0.027541	0.003506	0.109333
0	0	1	0	21.64103	0.61637	0.046272	-0.00325	0.281487
0	0	1	0	21.80388	0.66055	0.024569	0.011054	0.09945
0	0	1	0	21.75693	0.651953	0.016127	0.004303	-0.1399
1	1	2	1	20.0717	0.082778	0.086596	0.062024	0.4133
1	1	2	1	20.24951	0.145031	0.078896	0.013294	0.564105
1	1	2	1	20.42393	0.149991	0.103986	0.008973	0.312512
1	1	2	1	20.9205	0.349691	0.099095	0.013496	0.408751
1	1	2	1	21.27419	0.396103	0.089017	0.013767	0.532641
1	1	2	1	21.93124	0.280864	0.176206	0.009515	0.267296
1	1	2	1	22.03492	0.283737	0.070079	0.006378	0.261173
1	1	2	1	22.1672	0.227216	0.074939	0.008686	0.195226
1	1	2	1	22.35621	0.236537	0.081235	0.009482	0.326146
1	1	2	0.5	20.89404	0.16757	0.081541	0.059215	-0.0926
1	1	2	0.5	21.07283	0.206403	0.080812	0.018449	0.359117
1	1	2	0.5	21.22119	0.21867	0.090049	0.018314	0.224103
1	1	2	0.5	21.43855	0.302172	0.089382	0.016677	0.151506
1	1	2	0.5	21.56839	0.325165	0.092544	0.018107	0.129058
0	0	2	0.5	21.86493	0.269029	0.074191	0.017895	0.220993
0	0	2	0.5	21.98458	0.310097	0.074884	0.017293	0.269799
0	0	2	0.5	22.07	0.343789	0.054669	0.018414	0.163694
0	0	2	0.5	22.0951	0.309522	0.077503	0.01994	0.253441
0	0	2	0	20.76728	0.144159	0.111095	0.079883	0.2407
0	0	2	0	20.89583	0.174056	0.111393	0.029554	0.145532
0	0	2	0	21.01589	0.194828	0.112134	0.026915	0.21165
0	0	2	0	21.18566	0.257724	0.101607	0.026197	0.180178
0	0	2	0	21.31353	0.284347	0.102911	0.0246	0.112972
0	0	2	0	21.439	0.288954	0.117725	0.030517	0.286579
0	0	2	0	21.5949	0.302796	0.131974	0.03525	0.256238
0	0	2	0	21.7475	0.326054	0.11435	0.037221	0.120977
0	0	2	0	21.89912	0.377148	0.071445	0.030054	-0.02862
0	0	1	1	20.74557	0.147986	0.086424	0.066088	0.3698
0	0	1	1	20.90924	0.229727	0.044033	0.008077	-0.06895
0	0	1	1	21.44275	0.302773	0.060357	0.010626	0.765626
1	1	1	1	21.70825	0.417123	0.06543	0.009874	0.707598
1	1	1	1	22.01003	0.32678	0.057787	0.010192	0.101029
1	1	1	1	21.92268	0.257089	0.00695	0.001687	-0.4075
1	1	1	1	22.0193	0.291168	0.019892	0.004097	0.315357
1	1	1	1	22.21443	0.364745	0.039392	0.004428	0.50818
1	1	1	1	22.2673	0.356425	0.055367	0.010116	0.162807
1	1	2	0	20.35457	0.279172	0.080461	0.071048	0.3053
1	1	2	0	20.39958	0.266749	0.056655	0.01758	-0.05847
1	1	2	0	20.39438	0.241624	0.030791	0.007039	0.095023
0	0	2	0	21.13451	0.306904	0.06271	0.007827	1.327837
0	0	2	0	21.35613	0.373216	0.06821	0.00963	0.179504
1	1	2	0	22.51982	0.504552	0.041976	0.012715	1.377743
0	0	2	0	22.97223	0.586155	0.049249	0.023756	0.441832
0	0	1	0	20.84386	0.058413	0.121724	0.094186	0.6718
0	0	1	0	20.93643	0.059241	0.133932	0.033206	0.372183
0	0	1	0	21.09276	0.078098	0.148241	0.027688	0.210789

0	0	1	0	21.52102	0.244374	0.152246	0.041872	0.355666
1	1	1	0	22.28886	0.113698	0.133722	0.041596	0.268238
1	1	1	0	22.4158	0.077297	0.149812	0.03729	0.556913
1	1	1	0	22.46725	0.05391	0.106702	0.039014	0.045111
1	1	1	0	22.52311	0.067683	0.040104	0.021709	-0.1674
1	1	2	0	22.5387	0.057085	0.025295	0.016167	0.16558
0	0	1	1	20.32473	0.266909	0.098645	0.093123	0.4533
0	0	1	1	20.3773	0.246752	0.066905	0.022872	-0.11812
1	1	1	1	20.43815	0.265145	0.029942	0.014699	0.151531
1	1	1	1	20.49127	0.268152	0.036756	0.011022	0.163848
1	1	1	1	20.59001	0.306927	0.028475	0.005284	0.031705
0	0	1	1	20.69348	0.362183	0.016582	0.002482	-0.04921
0	0	1	1	22.22712	0.469006	0.017435	0.002946	0.908898
0	0	1	1	22.33902	0.295132	0.053565	0.008539	1.865632
0	0	1	1	22.41771	0.327014	0.057316	0.014149	0.085674
0	0	1	1	20.52346	0.250494	0.079055	0.079055	0.39
0	0	1	1	20.42185	0.148508	0.035783	0.011952	-0.12894
0	0	1	1	20.41888	0.151444	-0.00233	0.000584	-0.16778
0	0	1	1	20.48436	0.198867	-0.00257	0.001635	0.091173
0	0	1	1	20.51253	0.216415	0.00904	-0.0026	-0.2452
1	1	2	1	20.53005	0.232108	0.007567	0.006715	0.018114
1	1	2	1	20.79061	0.404757	0.007192	0.006145	0.418113
1	1	2	1	21.50907	0.391281	0.068441	0.001772	0.848948
1	1	2	1	21.65034	0.202323	0.152913	0.011901	0.288333
1	1	2	0.5	20.42974	0.293688	0.090394	0.090394	0.3047
1	1	2	0.5	20.35037	0.166467	0.088965	0.005382	0.000439
1	1	2	0.5	20.6422	0.311849	0.073082	0.006651	0.04942
1	1	2	0.5	20.72498	0.303557	0.07924	0.007009	0.309267
0	0	2	0.5	20.76537	0.29374	0.043383	0.007326	-0.13186
0	0	2	0.5	20.86201	0.338537	0.039359	0.007418	0.132044
0	0	2	0.5	21.15337	0.22168	0.031537	0.007609	0.166761
0	0	1	1	21.10363	0.172417	0.016741	0.007813	-0.099
0	0	2	1	21.06306	0.580571	0.037965	0.016251	0.167164
0	0	2	1	21.09556	0.575006	0.042991	0.007962	0.650113
0	0	2	1	20.84565	0.326644	0.101898	0.023882	0.258604
0	0	2	1	20.94158	0.315808	0.097517	0.024579	0.083071
0	0	2	1	21.20332	0.323901	0.10977	0.015206	0.562641
0	0	2	1	21.22929	0.240409	0.135043	0.016396	0.269484
0	0	2	1	21.23179	0.196131	0.068981	0.017065	-0.21753
0	0	2	1	21.2851	0.18743	0.071767	0.015014	0.07984
0	0	1	1	21.31137	0.625987	0.009319	0.009193	-0.6288
0	0	1	1	20.90095	0.444733	0.019242	0.004316	-0.1977
0	0	1	1	21.64335	0.641709	0.01761	0.003698	6.431397
0	0	1	1	21.58724	0.601695	0.032678	0.01139	0.280902
0	0	1	1	21.57337	0.590957	0.023955	0.010895	-0.17208
0	0	1	0	19.12369	0.324005	0.218964	0.204108	0.2478
0	0	1	0	19.91615	0.115033	0.050595	0.004464	-0.02668
0	0	1	0	19.95123	0.120241	0.034044	0.002439	0.454063
0	0	1	0	20.13467	0.236827	0.04491	-0.00953	0.220872
0	0	1	0	21.20419	0.177656	0.049869	0.001702	0.697943
0	0	1	0	21.45434	0.314287	0.06543	0.01042	0.196078
0	0	1	0	21.59731	0.480471	-0.06183	0.028316	0.171712
0	0	1	0	21.51183	0.43053	0.041876	0.014818	0.046952
0	0	2	1	20.83762	0.36062	0.052526	0.018104	0.3508
0	0	2	1	20.99365	0.421998	0.028914	0.011077	0.420335
0	0	2	1	21.27734	0.547377	0.039879	0.009457	0.133205
0	0	2	1	21.84722	0.500972	0.052896	0.006592	0.539888
0	0	2	1	22.05661	0.378781	-0.00024	0.011027	0.129771

1	1	2	1	22.05966	0.351082	0.040024	0.005727	0.092218
0	0	1	1	20.72313	0.194534	0.096137	0.031584	0.4274
0	0	1	1	20.95369	0.268973	0.110076	0.029973	0.521903
0	0	1	1	21.08476	0.265531	0.139719	0.031159	0.31432
0	0	1	1	21.5238	0.234724	0.051012	0.034533	-0.03132
0	0	1	1	21.78851	0.357037	0.061885	0.014535	0.572823
0	0	1	1	21.92271	0.441311	0.018889	0.01498	0.361832
0	0	1	1	21.90246	0.402663	0.054478	0.013947	0.141053
0	0	1	1	22.14055	0.463453	0.084713	0.020752	0.069011
0	0	1	1	20.11589	0.043802	0.055051	0.031389	-0.2802
0	0	1	1	20.42216	0.284681	0.018257	0.028409	-0.22402
0	0	1	1	20.3013	0.168965	0.006691	0.006817	0.311734
0	0	1	1	20.27903	0.145178	0.005293	-0.00818	0.022432
0	0	1	1	20.26464	0.120811	0.01551	-0.01146	0.084099
1	1	1	1	22.81268	0.408866	0.09581	0.026572	1.187582
1	1	1	1	23.0738	0.339805	0.056596	0.013814	-0.07731
0	0	2	1	20.98625	0.085244	0.088075	0.016304	0.2408
0	0	2	1	21.08924	0.122989	0.058647	0.008875	0.089268
0	0	2	1	21.07411	0.062444	0.069868	0.006069	0.078977
1	1	2	1	21.64372	0.462107	0.032341	0.005331	0.141401
1	1	1	1	22.43781	0.654321	0.051329	0.01263	2.129145
0	0	2	1	20.43928	0.072894	0.060835	0.024328	-0.03413
0	0	2	1	20.54776	0.107844	0.085856	0.027294	0.207455
0	0	2	1	20.58092	0.102639	0.066938	0.03255	0.147035
0	0	2	1	20.60206	0.105136	0.040142	0.022233	-0.01187
0	0	2	1	20.68777	0.141202	0.068677	0.012535	0.186661
0	0	1	1	21.33801	0.091922	0.09943	0.074243	-0.3898
0	0	1	1	21.32094	0.06646	0.0117	0.007686	-0.65174
0	0	1	1	21.36187	0.068186	0.02303	0.006235	0.401724
0	0	1	1	21.58313	0.182908	0.050626	0.008355	1.412264
0	0	1	1	22.14182	0.165921	0.053815	0.011668	0.844389
0	0	1	1	22.51772	0.38503	0.070812	0.015181	0.785529
0	0	2	0.5	22.56936	0.332163	0.10206	0.025374	0.301133
0	0	2	0.5	22.78538	0.400142	0.091661	0.023867	0.226379
0	0	2	0	20.74518	0.442389	0.011621	0.009091	-0.2493
0	0	2	0	20.84163	0.444295	0.0284	0.016006	0.412249
0	0	2	0	21.0486	0.556395	0.010076	0.014308	0.061532
0	0	2	0	22.59152	0.636574	0.032953	0.0059	1.79685
0	0	1	0	22.9325	0.496088	0.05422	0.012366	0.535601
0	0	1	0	22.92318	0.450597	0.058491	0.014743	0.13317
0	0	2	0.5	20.58474	0.304637	0.090709	0.050979	-0.1204
0	0	2	0.5	20.5093	0.183623	0.105321	0.024287	0.172101
0	0	2	0.5	20.75904	0.292821	0.106655	0.026487	0.301727
0	0	2	0.5	20.80871	0.281451	0.082598	0.025917	-0.00978
0	0	2	0.5	20.91447	0.297453	0.084489	0.024321	0.032531
0	0	2	0.5	21.21766	0.429036	0.109676	0.022733	0.509385
0	0	2	0.5	21.69813	0.421898	0.085953	0.028928	0.649103
0	0	2	0.5	21.76828	0.39244	0.098465	0.029086	0.052172
0	0	2	1	20.28673	0.201618	0.056182	0.032606	0.0006
0	0	2	1	20.32637	0.215544	0.055174	0.01713	0.116579
0	0	2	1	20.32044	0.181439	0.046457	0.012467	0.04273
0	0	2	1	20.60619	0.125977	0.039817	0.014685	0.045256
0	0	2	1	20.64962	0.134202	0.035545	0.010301	0.054504
0	0	2	1	20.72606	0.163403	0.044739	0.011462	0.206797
0	0	2	1	20.84772	0.21036	0.046893	0.017096	0.122996
0	0	2	1	20.94631	0.221252	0.041975	0.012301	0.049498
0	0	1	1	21.40539	0.173936	0.063734	0.061534	-0.0858
0	0	1	1	21.54275	0.254827	0.036691	0.008484	0.115764

1	1	1	1	21.67896	0.33563	0.027275	0.010888	-0.09009
1	1	1	1	21.61823	0.377237	-0.07242	-0.00568	-0.0476
1	1	1	1	21.66116	0.399647	0.015458	-0.00061	0.150061
1	1	1	1	21.71428	0.428907	0.018079	0.011857	0.271075
1	1	1	1	21.75552	0.49019	-0.00627	0.009288	-0.03052
0	0	1	1	21.83932	0.521533	0.030804	-0.00618	0.050044
0	0	2	1	20.51218	0.107234	0.080453	0.061255	0.1161
0	0	2	1	20.54529	0.110018	0.056532	0.001834	-0.06443
0	0	2	1	20.59405	0.129103	0.058348	0.002176	0.088593
0	0	2	1	20.65185	0.183792	0.001306	0.001711	-0.08104
0	0	2	1	20.68704	0.209192	0.004839	0.00305	0.206905
0	0	2	1	21.82707	0.291087	0.076353	0.007276	1.876704
1	1	2	1	21.82459	0.251211	0.063079	0.010512	0.482453
0	0	1	1	20.50522	0.143466	0.117427	0.09312	0.1366
0	0	1	1	20.56498	0.121392	0.129313	0.035959	0.235357
0	0	1	1	20.62412	0.126256	0.110714	0.023105	-0.08376
0	0	1	1	20.77293	0.150612	0.139596	0.026715	0.304512
0	0	1	1	21.11062	0.160225	0.128545	0.036601	0.226139
0	0	1	1	21.29335	0.227079	0.126509	0.037974	0.18105
0	0	1	1	21.37103	0.297957	0.061926	0.027554	-0.03522
0	0	1	1	20.4965	0.186312	0.078256	0.062351	-0.0322
0	0	1	1	20.53432	0.22387	0.029884	0.014763	-0.19295
0	0	1	1	20.54327	0.228166	0.023149	0.002383	0.115286
1	1	1	1	20.79429	0.214447	0.030043	0.012601	0.08614
1	1	1	1	20.81994	0.231723	0.018079	0.01518	-0.14193
0	0	1	1	20.74084	0.236107	-0.05041	-0.00284	-0.2394
1	1	1	1	20.183	0.139129	0.093463	0.006583	0.178926
0	0	1	0	20.12666	0.304896	0.089918	0.089295	0.0782
0	0	1	0	20.34283	0.388924	0.067088	0.017682	-0.02376
0	0	1	0	20.48737	0.444895	0.067588	0.020021	0.678144
0	0	1	0	21.48447	0.185628	0.05458	0.014514	0.143238
0	0	1	0	21.69062	0.266649	0.062617	0.010702	-0.10473
0	0	1	0	21.78735	0.309268	0.042347	0.015925	0.385865
0	0	2	1	20.662	0.051767	0.062044	0.056585	-0.1697
0	0	2	1	20.73113	0.101006	0.062911	0.005024	0.420588
0	0	2	1	20.86478	0.110151	0.10639	0.01967	0.709607
0	0	2	1	20.97133	0.142306	0.095084	0.018531	0.33083
0	0	2	1	21.85667	0.131746	0.057108	0.020924	0.87576
0	0	2	1	21.93608	0.138212	0.059871	0.009149	0.325042
1	1	2	0	19.69331	0.069497	0.106708	0.051609	0.053
1	1	2	0	19.65167	0.032908	0.040704	-0.0082	-0.13379
1	1	2	0	19.70276	0.079054	0.013129	-0.0095	0.050231
1	1	2	0	19.71586	0.067888	0.019944	-0.00751	0.25174
1	1	2	0	19.75569	0.091644	0.006132	-0.0065	0.136714
0	0	2	0	19.80675	0.130056	0.011083	-0.01171	-0.00244
0	0	2	0	19.78023	0.081343	0.050366	-0.01319	0.036361
1	1	2	0	19.86759	0.096357	0.081088	-0.00646	0.324495
0	0	2	1	21.01405	0.746045	0.067329	0.067329	0.0444
0	0	2	1	21.18503	0.607799	0.054222	0.009222	0.121953
0	0	1	1	22.07076	0.568148	0.033046	0.008189	0.236566
0	0	1	1	22.16744	0.578754	0.057202	0.003486	1.174746
0	0	1	1	22.37522	0.642095	0.038994	0.006612	-0.12467
0	0	1	1	20.53931	0.285245	0.135784	0.135784	0.3194
0	0	1	1	20.88066	0.213175	0.122857	0.033091	0.095306
0	0	1	1	21.0736	0.204877	0.102003	0.030158	-0.10356
0	0	1	1	21.17247	0.1902	0.11318	0.024868	0.104044
0	0	1	1	21.34869	0.146661	0.071562	0.025276	0.050511
0	0	1	1	21.734	0.355422	0.053017	0.015575	0.27602

0	0	1	1	21.81503	0.249062	0.063494	0.017149	0.097909
0	0	2	1	21.64831	0.568037	0.079118	0.019351	0.460892
0	0	2	1	22.21328	0.695091	0.07323	0.010902	0.870099
0	0	2	1	22.93903	0.595094	0.062833	0.00946	0.424447
0	0	2	1	23.0982	0.591092	0.082363	0.008306	0.395057
1	1	2	1	23.20433	0.583318	0.066438	0.012095	-0.36426
1	1	2	1	23.26305	0.565386	0.055955	0.011055	-0.16736
1	1	1	1	19.50615	0.317796	0.175113	0.175113	0.1627
1	1	1	1	20.69359	0.195166	0.06705	-0.03511	0.077272
1	1	1	1	20.64302	0.088755	0.086643	-0.0043	0.222211
1	1	1	1	21.39837	0.096654	0.061509	0.005271	0.371382
1	1	1	1	23.0854	0.356729	0.054471	0.012588	0.7592
1	1	1	1	23.15573	0.340893	0.079734	0.018938	0.477291
1	1	1	1	23.22742	0.317799	0.084318	0.021626	0.178313
1	1	2	0	21.67267	0.694704	0.063725	0.051215	0.6024
1	1	2	0	22.10346	0.769542	0.048924	0.013642	0.023101
1	1	2	0	22.41277	0.695716	0.05181	0.009765	0.397971
1	3	2	0	22.81499	0.726086	0.047948	0.01044	0.796748
1	1	2	0	23.04025	0.747079	0.055126	0.012314	0.524446
1	1	2	0	23.39464	0.741021	0.04674	0.012352	0.040792
0	0	2	0	19.82625	0.189512	0.123149	0.089596	0.1279
0	0	2	0	20.69318	0.155342	0.056989	0.030783	0.179429
0	0	2	0	20.73802	0.198674	0.018669	0.009461	0.284396
0	0	2	0	20.75229	0.179886	0.050397	0.010152	0.245934
1	1	2	0	20.8492	0.219155	0.055104	0.015727	0.057076
1	1	2	0	20.86009	0.217259	0.067676	0.016648	-0.02598
1	1	3	0	20.44255	0.061204	0.055173	0.040524	-0.1383
1	1	3	0	20.4848	0.096229	0.011977	0.009711	-0.04797
1	1	3	0	20.59218	0.141447	0.0513	0.00639	1.176243
1	1	1	0	21.05604	0.081527	0.114274	0.029512	0.275833
1	1	1	0	21.22179	0.051395	0.215261	0.094299	0.572436
1	1	1	0	21.45545	0.161845	0.189102	0.086982	0.088104
0	0	1	1	21.06437	0.555829	0.063067	0.063067	0.1329
0	0	1	1	20.97311	0.545789	0.002449	0.003781	-0.29655
0	0	1	1	21.24079	0.447386	0.028193	0.007995	-0.02918
0	0	1	1	21.32697	0.480793	0.033546	0.005208	0.450868
0	0	1	1	21.28435	0.520285	-0.0393	0.007215	0.060364
0	0	1	1	21.22038	0.498182	0.024565	0.012095	-0.21019
0	0	2	0.5	20.02284	0.229418	0.071911	0.071911	0.1781
0	0	2	0.5	20.47217	0.306581	0.059078	0.004976	0.147903
0	0	2	0.5	21.16068	0.257639	0.053521	0.003418	0.681244
0	0	2	0.5	21.77182	0.287865	0.083573	0.010508	1.107754
0	0	2	0.5	21.88888	0.364493	0.020509	0.013101	0.129295
1	1	1	1	20.55724	0.395657	0.066255	0.066255	0.0483
1	1	1	1	20.54844	0.348509	0.063155	0.00778	0.086198
1	1	1	1	20.59756	0.340437	0.060921	0.009036	0.024661
1	1	1	1	22.06346	0.106863	0.023208	0.008401	0.352081
0	0	2	1	19.93619	0.214658	0.110023	0.110023	0.1916
0	0	2	1	20.10336	0.262975	0.106325	0.013414	0.182843
0	0	2	1	20.7322	0.243246	0.0668	0.015173	0.388074
0	0	2	1	20.91064	0.324312	0.046343	0.006105	0.474628
0	0	2	1	21.03198	0.385132	0.039275	0.000232	0.300482
0	0	2	1	21.14011	0.446987	0.051939	0.000668	0.214958
0	0	1	0	20.50009	0.152971	0.11193	0.06205	-0.2119
0	0	2	1	20.96665	0.69214	0.091126	0.091126	0.1262
0	0	2	1	21.0857	0.456288	0.068621	0.011733	0.0117
0	0	2	1	21.31445	0.523338	0.082209	0.013847	0.213498
0	0	2	1	21.5848	0.537062	0.14445	0.034194	0.392038

0	0	2	1	21.69292	0.559178	0.088682	0.027827	0.003314
0	0	2	1	21.77596	0.544205	0.087503	0.025724	0.049073
1	1	1	1	19.61269	0.239344	0.112173	0.112173	0.0283
1	1	1	1	21.07098	0.457014	0.017698	0.005738	0.088746
1	1	1	1	21.10257	0.221467	0.056766	0.003159	1.934641
0	0	1	1	21.40957	0.261013	0.043956	0.007621	0.243882
0	0	1	1	21.51292	0.284895	0.0679	0.006374	0.328005
1	1	1	1	21.75353	0.356502	0.065836	0.018234	0.288702
0	0	1	1	20.72759	0.185482	0.084412	0.034847	-0.1895
0	0	1	1	20.82805	0.212017	0.058185	0.002195	0.028781
1	1	1	1	20.92906	0.264621	0.061014	0.002456	0.115089
1	1	1	1	21.49328	0.557854	0.01691	0.003865	0.594044
1	1	1	1	21.63522	0.588496	0.038619	-0.0003	0.980712
0	0	2	1	20.44847	0.17007	0.114899	-0.00978	0.1407
0	0	2	1	20.61135	0.22827	0.11152	-0.00578	0.344967
0	0	2	1	21.88425	0.36725	0.048888	-0.00472	0.811833
0	0	2	1	22.01937	0.387411	0.088055	0.030156	0.763088
0	0	2	1	22.29043	0.510668	0.069205	0.021365	0.022097
1	1	2	1	20.30016	0.080063	0.150077	0.040124	0.212026
1	1	2	1	20.81027	0.118165	0.123056	0.040461	0.314385
1	1	2	1	20.97228	0.12968	0.120966	0.031455	0.455829
1	1	2	1	21.35585	0.126402	0.085265	0.032619	0.154899
1	1	2	1	21.4667	0.173309	0.084716	0.020331	0.168889
0	0	1	1	20.62649	0.32609	0.068501	0.01326	0.0783
0	0	1	1	20.66209	0.304008	0.064028	0.015003	0.082914
0	0	1	1	21.36438	0.192049	0.053229	0.014282	0.488049
0	0	1	1	21.47632	0.227823	0.056242	0.006949	0.241397
0	0	1	1	21.57635	0.264554	0.041793	0.008226	0.000091
1	3	1	1	21.01147	0.318504	0.147312	0.040836	0.2285
1	3	1	1	22.21937	0.219397	0.05763	0.046538	0.363306
1	3	1	1	22.4295	0.267918	0.081326	0.022217	0.987151
1	3	2	1	21.71676	0.352748	0.091568	0.015295	0.02243
0	0	3	0.333333	20.64808	0.309443	0.115642	0.017886	0.068613
0	0	3	0.333333	20.74247	0.314236	0.06514	0.020908	-0.27094
0	0	3	0.333333	20.99334	0.415714	0.055391	0.016303	0.440189
0	0	3	0.333333	21.07233	0.4257	0.055162	0.018025	0.224788
0	0	3	0.333333	21.78199	0.267328	0.035671	0.014543	0.739153
1	1	1	1	20.22633	0.254012	0.06633	0.049125	0.0416
0	0	1	1	21.06688	0.244024	0.06555	0.012933	1.200365
0	0	1	1	21.64954	0.359387	0.084383	0.021464	0.969184
0	0	1	1	21.79126	0.386636	0.07479	0.023342	0.350606
0	0	1	1	21.75398	0.431102	-0.01845	0.014772	0.036041
0	0	2	1	20.19949	0.101985	0.10143	0.051654	-0.321
0	0	2	1	20.25369	0.116188	0.066206	0.018135	0.019004
0	0	2	1	20.32359	0.132322	0.074395	0.012283	0.270939
0	0	2	0.5	20.53384	0.262395	0.057416	0.011663	0.315522
0	0	2	0.5	20.85928	0.373288	0.072372	0.007715	0.3583
1	1	1	0	20.59169	0.255237	0.071563	0.041922	0.0619
1	3	1	0	22.40255	0.065848	0.044827	0.033162	0.766363
1	3	1	0	23.09618	0.110476	0.041945	0.021218	0.560845
1	3	1	0	23.27582	0.472835	0.086405	0.0327	0.951733
0	0	2	1	20.25081	0.245564	0.062619	0.017649	-0.366
0	0	2	1	21.04861	0.193777	0.044086	0.006932	0.420888
0	0	2	1	21.30512	0.216634	0.018055	0.005144	0.378257
0	0	2	1	20.94932	0.153021	0.089315	0.047194	-0.1604
0	0	2	1	21.09578	0.212217	0.072328	0.020616	0.251818
0	0	2	1	21.14275	0.202541	0.061343	0.00928	-0.00923
0	0	2	1	21.11876	0.154613	0.014206	-0.00037	-0.35734

0	0	2	1	19.95387	0.066869	0.144206	0.073408	-0.0676
0	0	2	1	20.07302	0.122823	0.12871	0.031351	0.073054
0	0	2	1	20.26559	0.184991	0.148384	0.034714	0.439392
0	0	2	1	20.63282	0.158435	0.122437	0.037579	0.175485
1	1	2	1	20.6994	0.110484	0.161458	0.039493	0.069846
0	0	2	1	20.12769	0.293575	0.04518	0.0384	-0.0076
0	0	2	1	20.04433	0.216692	0.025375	0.028573	-0.16695
0	0	2	1	20.18769	0.318534	0.036947	0.024768	0.20038
0	0	2	1	22.58818	0.475818	0.008936	0.013015	-0.12028
0	0	2	1	22.68385	0.409437	0.061544	0.026234	3.520213
0	0	2	0.5	21.38843	0.031878	0.122569	0.06164	0.0293
0	0	2	0.5	21.51923	0.053518	0.134853	0.034109	0.014989
0	0	2	0.5	21.62074	0.054582	0.134622	0.02363	0.21704
0	0	2	0.5	21.7387	0.074287	0.119352	0.027652	0.903701
0	0	2	0.5	21.75485	0.033597	0.056353	0.018183	-0.16589
1	1	2	1	20.6677	0.273677	0.044675	0.033263	-0.0769
1	1	2	1	20.76784	0.400671	-0.05726	-0.01854	-0.45593
1	1	2	1	20.86371	0.425666	0.037592	0.01702	1.551898
1	1	2	1	21.40116	0.440541	0.039392	0.007432	0.375105
1	1	2	1	21.58352	0.405493	0.047366	0.006299	0.054744
0	0	2	1	20.77235	0.161718	0.111467	0.017552	0.549395
0	0	2	1	20.82932	0.159947	0.082439	0.02533	0.118661
0	0	2	0	20.90336	0.168529	0.105052	0.023387	0.1606
0	0	2	0	21.06142	0.22437	0.091715	0.028366	0.228921
0	0	2	0	21.04501	0.176695	0.018219	-0.00211	-0.13383
0	0	2	0	21.2332	0.202494	0.143179	0.02888	0.532829
1	1	3	0.333333	21.4002	0.568958	0.138346	0.138346	0.3167
1	1	3	0.333333	21.80521	0.404806	0.060544	0.020863	0.178409
1	1	3	0.333333	21.64262	0.277671	0.047543	0.022311	-0.22965
1	1	1	1	20.84108	0.485504	0.058318	0.026807	0.0129
1	1	1	1	20.94205	0.51501	0.05668	0.012001	0.102249
1	1	1	1	21.04554	0.539762	0.03666	0.009677	0.1119
1	1	1	1	21.09554	0.533706	0.036047	0.011801	0.243406
0	0	2	1	21.43613	0.344148	0.08872	0.047955	0.0846
0	0	2	1	21.46736	0.332968	0.102475	0.020557	0.078193
0	0	2	1	21.41489	0.333652	0.101615	0.022958	0.046937
0	0	2	1	21.37388	0.267932	0.06193	0.017979	0.19487
0	0	1	0	20.46867	0.372891	0.105691	0.048491	0.3138
0	0	1	0	20.60978	0.407786	0.065208	0.012915	0.160546
0	0	1	0	20.86023	0.501303	0.054579	-0.00821	0.118873
1	1	1	1	20.08277	0.150819	0.075237	0.032117	-0.0701
1	1	1	1	20.11934	0.174087	0.048956	0.01571	-0.01268
1	1	1	1	20.223	0.218844	0.062309	0.011643	0.232464
1	1	1	1	20.29461	0.224079	0.078301	0.016165	0.111704
0	0	2	1	20.33557	0.272395	0.074333	0.042475	0.0001
1	3	2	1	20.56399	0.384593	0.075544	0.017306	0.037218
0	0	2	1	20.77416	0.480256	0.052992	0.020115	0.042435
0	0	2	0	20.28335	0.130156	0.110566	0.069778	0.2214
0	0	2	0	20.55932	0.260451	0.071306	0.023875	0.331138
0	0	1	0	20.89953	0.374964	0.125633	0.038716	1.147781
0	0	1	0	21.3655	0.329801	0.035091	0.048917	-0.00066
1	1	1	1	20.48445	0.380157	0.073262	0.058543	0.0768
1	1	1	1	20.70704	0.461522	0.061102	0.004516	0.115012
1	1	1	1	20.91863	0.530887	0.054577	0.006023	0.083867
1	1	1	1	21.06326	0.564199	0.041147	0.004892	0.005529
0	0	1	1	20.06927	0.321263	0.092041	0.07158	0.0674
0	0	1	1	20.44495	0.4994	0.060581	0.026091	0.234204
0	0	1	1	20.67666	0.574593	0.024699	0.01187	0.126419

0	0	1	1	20.72672	0.510973	0.026392	0.009992	0.004454
0	0	1	1	20.39144	0.19551	0.091116	0.05445	0.1742
0	0	1	1	20.66435	0.32559	0.100845	0.008998	0.297719
0	0	1	1	20.81097	0.325507	0.109627	0.001364	0.579709
0	0	1	1	20.83009	0.229438	0.116332	0.013732	0.176088
0	0	2	0.5	20.52923	0.258248	0.121153	0.076313	0.1566
1	1	2	0.5	20.63906	0.238342	0.143507	0.029924	0.268308
1	1	2	0.5	20.69406	0.187458	0.154147	0.031649	-0.05775
1	1	2	0.5	20.69664	0.140898	0.125649	0.031472	-0.05537
0	0	1	0	20.19392	0.174304	0.098121	0.092615	0.0467
0	0	1	0	20.35643	0.257455	0.054411	0.000416	0.172372
1	1	1	0	20.66545	0.408966	0.047858	0.004195	0.416868
1	1	1	0	20.67521	0.338983	0.049434	0.008046	0.126561
0	0	2	0	20.23681	0.310197	0.069431	0.069431	0.185
0	0	2	0	20.28142	0.303332	0.076635	0.027082	0.038303
0	0	2	0	20.26774	0.236741	0.081634	0.027387	0.141158
0	0	2	0	20.37679	0.252843	0.08901	0.025544	0.273206
1	1	1	0	21.13285	0.167814	0.111272	0.025171	0.225223
1	1	1	0	21.25157	0.183843	0.105619	0.026524	0.110417
1	1	1	0	21.34049	0.180188	0.102448	0.022935	0.076669
0	0	1	1	20.31573	0.221195	0.095785	0.095785	0.0169
0	0	1	1	20.3616	0.19735	0.083326	0.004989	-0.12635
0	0	1	1	20.45493	0.181901	0.100715	-0.00341	-0.02212
0	0	1	1	20.51154	0.182984	0.068094	0.003088	0.371707
0	0	2	0.5	21.42863	0.156781	0.055144	0.00251	0.382441
1	3	2	0.5	21.67665	0.28238	0.066531	0.017215	0.339514
0	0	2	0.5	21.81762	0.368398	0.032067	0.015424	-0.08218
0	0	2	1	20.66446	0.166912	0.096161	0.096161	0.0061
0	0	2	1	20.71251	0.121803	0.087783	-0.00392	0.093953
0	0	2	1	21.29559	0.411057	0.059414	0.001206	0.544661
0	0	2	1	21.57605	0.482157	0.094473	0.00564	1.018789
0	0	2	1	20.34891	0.556965	0.06806	0.06806	0.3373
0	0	2	1	20.74376	0.627461	0.089613	0.002361	0.479125
0	0	2	1	20.80314	0.635896	0.016542	-0.00823	-0.18456
0	0	2	1	20.65631	0.553113	0.001109	-0.02378	-0.56823
0	0	1	1	20.74168	0.552056	0.054003	-0.00256	0.003752
0	0	1	1	20.82804	0.529112	0.057467	-0.0025	0.147739
0	0	1	1	20.96306	0.525272	0.04118	0.000665	-0.02576
0	0	3	0.666667	20.86301	0.423262	0.113862	0.113862	0.0928
0	0	3	0.666667	21.23816	0.256116	0.094521	0.016586	0.21513
1	1	3	0.666667	21.3035	0.209417	0.134502	0.02858	0.218854
1	1	3	0.666667	21.40976	0.195924	0.143875	0.039144	0.07906
0	0	1	1	19.56933	0.230893	0.148958	0.148958	0.0698
0	0	1	1	20.18657	0.150667	0.108241	0.034357	0.170885
0	0	1	1	20.36951	0.176673	0.103824	0.038137	0.466394
0	0	1	1	20.48823	0.177966	0.109708	0.027562	0.137286
0	0	1	1	21.97665	0.492649	0.004006	-0.02407	-0.36063
1	1	2	1	21.72374	0.637015	0.045676	0.01824	0.038209
0	0	1	1	21.03174	0.10335	0.123389	0.028243	0.2991
0	0	1	1	21.16831	0.134752	0.110051	0.029388	0.247591
0	0	1	1	21.62145	0.084828	0.081776	0.023001	0.254016
0	0	2	0.5	19.99907	0.27876	0.071402	0.009574	0.0226
1	1	2	1	20.29073	0.131532	0.121757	0.030403	0.1331
1	1	2	1	20.50081	0.123615	0.195466	0.049454	0.205061
1	1	2	1	20.81754	0.295314	0.10413	0.052982	-0.02715
1	2	1	1	20.79059	0.092313	0.100873	0.02083	0.2028
1	2	1	1	20.89575	0.091033	0.106169	0.010679	0.211376
1	2	1	1	20.9833	0.086608	0.119491	0.013481	0.256811

0	0	1	0	19.98724	0.137297	0.096199	0.049163	0.0316
0	0	1	0	20.06485	0.182882	0.058913	0.01068	-0.02063
0	0	1	0	20.62574	0.52632	0.024381	0.007779	0.463033
0	0	2	0.5	20.70855	0.308645	0.031183	0.007165	-0.0054
1	1	2	0.5	20.75501	0.3234	0.034342	0.007662	-0.06378
0	0	2	1	20.55758	0.277149	0.064351	0.01617	0.018
0	0	2	1	20.64309	0.296518	0.051518	0.004848	0.351635
0	0	2	1	20.61029	0.246788	0.061707	0.004089	0.101048
0	0	2	0	20.61441	0.26835	0.099232	0.049247	0.2593
0	0	2	0	21.07353	0.485514	0.066312	0.021354	-0.07471
0	0	2	0	21.10632	0.471221	0.05873	0.015085	0.00851
0	0	1	1	20.27288	0.332051	0.064632	0.038752	0.0035
0	0	1	1	20.28245	0.296465	0.065321	0.005533	0.001193
0	0	1	1	20.39368	0.334735	0.060087	0.00509	0.011204
0	0	1	1	19.91971	0.047692	0.105889	0.052286	0.0058
0	0	1	1	20.2425	0.246065	0.107188	0.021187	1.240926
0	0	1	1	20.29218	0.219411	0.073521	0.015078	0.112172
0	0	4	0.5	20.75121	0.266793	0.12122	0.061931	0.2318
0	0	4	0.5	20.93102	0.370369	0.041804	0.013847	0.038102
0	0	4	0.5	21.14811	0.445234	0.072443	0.014617	0.354757
0	0	2	1	21.77512	0.30473	0.098098	0.06018	0.2509
0	0	2	1	21.82724	0.296381	0.067877	0.027569	0.111776
0	0	2	1	21.89604	0.275186	0.075546	0.021929	0.000403
0	0	1	1	19.95085	0.265181	0.052647	0.05342	-0.2308
0	0	1	1	20.59549	0.368542	0.013379	0.005308	0.505726
0	0	1	1	20.72036	0.409544	0.043289	0.007442	0.794447
0	0	2	1	21.36972	0.139161	0.082594	0.071002	-0.0269
0	0	2	1	21.49122	0.20682	0.045601	0.013513	-0.21244
0	0	2	1	21.49777	0.188534	0.028263	0.015468	0.04957
0	0	2	1	20.54219	0.3341	0.127665	0.127665	1.8749
0	0	2	1	20.62246	0.246768	0.188488	0.028675	0.337848
0	0	2	1	20.7599	0.232747	0.141125	0.020096	0.016503
0	0	1	0	20.21612	0.112141	0.106586	0.083297	0.1156
0	0	1	0	20.30387	0.130072	0.093776	0.020035	0.205309
0	0	1	0	20.37542	0.144373	0.07637	0.013532	-0.01668
1	1	2	0	20.77928	0.11813	0.127886	0.106665	0.24
1	1	2	0	20.79101	0.147396	0.092586	0.021914	-0.0984
1	1	2	0	20.97734	0.202902	0.131759	0.037625	0.543358
0	0	1	0	21.4166	0.436743	0.06069	0.06069	0.2395
0	0	1	0	21.42469	0.395059	0.068255	0.02391	0.078007
1	3	1	0	20.15864	0.332496	0.057243	0.06092	-0.0792
1	3	1	0	20.26755	0.399669	0.013981	-0.00184	-0.12501
1	3	1	0	20.47171	0.432147	0.020736	-0.00065	0.57138
1	1	2	1	21.87891	0.183752	0.089331	0.074661	0.4987
1	1	2	1	22.00064	0.201999	0.118663	0.031456	0.436278
1	1	2	1	22.12507	0.200134	0.151451	0.049995	0.257705
0	0	1	0	21.18274	0.197331	0.125124	0.14605	0.3005
0	0	1	0	21.22535	0.23468	0.0194	0.005852	-0.04334
0	0	1	0	21.50808	0.347313	0.087669	0.00377	0.669477
0	0	1	0	20.30453	0.124378	0.086292	0.109531	0.0678
0	0	1	0	20.47528	0.241356	0.022869	0.002579	-0.17895
0	0	1	0	20.65404	0.27459	0.028448	0.013369	0.082433
1	1	1	0	20.31626	0.426373	0.090839	0.090839	0.4657
1	1	1	0	20.62881	0.523939	0.092148	0.023843	0.570938
1	1	1	0	21.44099	0.483489	0.087109	0.021035	0.881126
0	0	1	1	20.14951	0.102532	0.093233	0.093233	0.2459
0	0	1	1	20.20785	0.09666	0.086589	0.028292	0.080183
0	0	1	1	20.30282	0.109133	0.088279	0.027598	0.029645

1	1	1	0	20.8285	0.07708	0.131424	0.131424	0.4023
1	1	1	0	20.94948	0.090086	0.137604	0.030718	0.169569
1	1	1	0	21.01277	0.09619	0.112807	0.030216	-0.09317
0	0	1	1	20.8306	0.13611	0.095498	0.095498	0.1059
0	0	1	1	20.87458	0.18659	0.051894	-0.01018	0.135419
0	0	1	1	20.97725	0.240211	0.065632	-0.01103	0.106276
0	0	1	1	20.96234	0.298873	0.074093	0.074093	0.2097
0	0	1	1	21.05006	0.333023	0.039341	0.010967	-0.07049
0	0	1	1	21.2747	0.482561	0.010237	0.0043	-0.15331
1	1	2	0.5	22.21482	0.498779	0.073976	0.069583	0.2011
1	1	2	0.5	22.51678	0.574808	0.068831	0.023944	0.692969
0	0	2	1	21.08829	0.209822	0.057318	0.016131	-0.15406
0	0	2	1	21.15048	0.256143	0.019712	0.007951	0.007599
1	1	1	1	20.42375	0.305047	0.168416	0.168416	0.1053
0	0	1	1	21.09048	0.219313	0.081587	0.014099	0.136894
0	0	2	1	20.9736	0.323376	0.103201	0.008511	0.3701
1	1	1	1	20.91035	0.240048	0.093653	0.056339	-0.0073
0	0	2	0	20.37884	0.12124	0.08624	0.038419	-0.0727
0	0	2	0	20.56885	0.186176	0.105317	0.070388	0.1668
1	1	1	1	21.4248	0.407103	0.064848	0.05472	0.6758
0	0	2	0	20.40351	0.141531	0.14016	0.14016	0.6296
0	0	2	0	21.38829	0.108389	0.101582	0.101582	0.0004
0	0	2	0	20.65499	0.270947	0.09219	0.09219	0.2833
0	0	2	0	20.24926	0.208351	0.124609	0.124609	-0.0841
1	1	2	1	23.48561	0.476878	0.171597	0.028005	0.200114
1	1	2	1	24.88933	0.618167	0.127815	0.057026	-0.07771
1	1	2	1	24.88008	0.608428	0.059112	0.031579	-0.20292
1	1	2	1	24.86655	0.607347	0.035161	0.021234	-0.18654
1	1	2	1	24.83787	0.614052	0.023537	0.007702	-0.23046
1	1	2	1	24.8432	0.618996	0.015244	0.011246	-0.00372
1	1	2	1	24.7878	0.547146	0.07191	0.021394	0.646691
1	1	2	1	25.02428	0.559441	0.104181	0.035393	0.456147
1	1	2	1	25.22907	0.497172	0.148086	0.048989	0.355495
1	1	1	0	22.39794	0.655224	0.043958	0.013754	0.116018
1	1	1	0	22.41295	0.647677	0.068806	0.008173	0.180058
1	1	1	0	22.59302	0.692709	0.017751	0.007137	-0.26915
1	1	1	0	22.76836	0.756158	0.021182	0.003297	-0.06629
1	1	1	0	22.76106	0.730472	0.058522	0.008229	0.573445
1	1	1	0	22.81583	0.748192	0.042791	0.027456	-0.16751
1	1	1	0	22.8217	0.740725	0.051089	0.00474	0.13091
1	1	1	0	22.63829	0.602813	0.126849	0.004188	0.690061
1	1	1	0	22.6118	0.598246	0.081391	0.004854	-0.42849
1	1	1	0	22.79638	0.647223	0.044739	0.006429	-0.04099
1	1	2	1	22.8633	0.772454	0.082482	-0.00183	0.292518
1	3	2	1	22.79708	0.738768	0.044616	0.004387	-0.49009
1	3	2	1	22.84726	0.703628	0.070606	0.002831	-0.18983
1	3	2	1	22.96898	0.774066	0.038785	0.009018	-0.16119
1	3	2	1	23.04199	0.819536	0.041809	0.007411	0.263494
1	3	2	1	22.96454	0.786569	0.058415	0.002207	-0.08417
1	3	2	1	22.49317	0.778384	-0.05685	-0.00234	0.331378
1	3	2	1	22.22546	0.544208	0.083096	0.00181	-0.25711
1	3	2	1	22.38634	0.562183	0.034366	0.006839	-0.52778
1	3	2	1	22.66744	0.649181	0.016598	0.005556	-0.01592
1	1	1	1	21.00868	0.465378	0.039498	-0.0147	0.105887
1	1	1	1	21.51314	0.156845	0.028181	-0.00519	-0.00589
1	1	1	1	21.56558	0.180886	0.044849	-0.00073	0.086744
1	3	1	1	21.61502	0.172934	0.062767	0.001945	0.079835
1	3	1	1	21.66572	0.149086	0.066286	0.002453	0.029169

1	1	3	0	23.09684	0.809762	0.047422	0.008664	-0.02069
1	1	3	0	22.99176	0.73857	0.087819	0.020757	0.9172
1	1	3	0	22.96021	0.622476	0.131248	0.033092	0.106518
1	1	3	0	23.41756	0.659946	0.085088	0.046755	-0.32873
1	1	2	1	23.47806	0.612747	0.106937	0.049742	0.314758
1	1	2	1	23.70859	0.643503	0.07102	0.008744	-0.08039
1	1	2	1	23.71344	0.561085	0.049349	0.010038	-0.02142
1	1	2	1	23.71269	0.610859	0.039687	0.00392	-0.17198
1	1	2	1	23.76435	0.602584	0.060758	0.028579	0.125275
1	1	3	0	23.91744	0.628709	0.06348	0.002576	0.175663
1	1	3	0	23.95576	0.644595	0.04536	0.010225	-0.03961
0	0	2	1	22.0214	0.306333	0.090291	-0.00802	0.1376
0	0	2	1	22.01148	0.192468	0.121942	0.037099	0.268388
0	0	2	1	22.31052	0.297514	0.110874	0.032136	0.390505
0	0	2	1	22.49235	0.352168	0.102612	0.032672	0.259285
1	1	1	0	23.10356	0.716205	0.041216	0.008922	0.6053
1	1	1	0	23.09278	0.709556	0.060495	0.023649	0.161272
1	1	1	0	23.15025	0.758883	0.045685	-0.00065	-0.08858
1	1	1	0	23.33957	0.716159	0.007702	-0.00162	-0.16776
1	1	1	0	23.34561	0.714258	0.012511	0.001387	0.495241
0	0	1	0	22.97648	0.60602	0.01171	-0.00244	1.235185
0	0	1	0	23.59488	0.777517	0.022315	0.001706	-0.64589
0	0	1	0	23.90319	0.807159	0.03941	0.002691	0.670323
0	0	1	0	24.35268	0.853442	0.024467	-0.00143	-0.09259
1	1	1	0	22.44798	0.710889	0.034407	0.004933	-0.16614
1	1	1	0	22.5279	0.723751	0.032266	0.009063	-0.01804
1	1	1	0	22.64614	0.728816	0.038744	0.007187	0.016787
1	1	1	0	22.48184	0.679263	0.051261	0.01491	0.047024
1	1	1	0	22.30897	0.610864	0.038688	0.011474	-0.07564
1	1	1	0	22.35668	0.620034	0.034216	0.008231	0.055025
1	1	1	0	22.57744	0.687092	0.024789	0.00846	-0.08007
1	1	1	0	22.70579	0.719067	0.032524	0.009626	0.368773
1	1	1	0	22.79696	0.734872	0.038126	0.011858	-0.08636
1	3	1	0	22.81778	0.736718	0.036298	0.009007	0.053735
0	0	1	1	21.135	0.680193	0.042661	0.010481	0.523447
0	0	1	1	21.67253	0.784514	0.062053	0.010967	0.407586
0	0	1	1	21.87415	0.818015	0.069339	0.000183	0.983385
0	0	1	1	22.31931	0.850905	0.067521	0.038019	0.550079
0	0	1	1	22.36719	0.827508	0.064177	0.032284	0.228106
0	0	2	1	22.53605	0.834283	0.049027	0.014789	-0.23372
0	0	1	1	22.98201	0.709581	0.068555	0.035624	0.906549
1	1	3	0.666667	23.06368	0.659935	0.034406	0.016728	-0.4924
0	0	3	0.666667	23.43706	0.704569	0.04915	0.011872	0.753642
0	0	3	0.666667	23.79265	0.669438	0.043873	0.008163	0.829966
0	0	3	0.666667	24.31993	0.730328	0.030662	0.004157	-0.03947
0	0	3	0.666667	24.71514	0.791041	0.038159	0.006577	0.696136
0	0	3	0.666667	24.76585	0.777714	0.04526	0.00929	0.168806
0	0	3	0.666667	24.59118	0.72299	0.04395	0.004954	0.306365
0	0	3	0.666667	24.45981	0.659761	0.04849	0.009378	-0.02539
1	1	3	1	22.18403	0.706087	0.048227	0.009572	-0.06907
1	1	3	1	21.95803	0.560586	0.100841	0.021675	0.620346
1	1	3	1	22.03979	0.564598	0.033455	0.012764	-0.24602
1	1	3	1	22.06179	0.571064	0.024398	0.007567	-0.28337
1	1	3	1	21.72774	0.401144	0.039002	0.00559	-0.16924
1	1	3	1	21.68502	0.316702	0.103579	0.022207	1.197894
1	1	3	1	21.94135	0.329449	0.06236	0.013438	-0.09865
1	1	1	1	22.14018	0.310614	0.078174	0.015031	0.143177
1	1	1	1	22.27336	0.365988	0.087394	0.020057	0.112709

0	0	1	1	22.42147	0.423728	0.050865	0.014921	0.122698
0	0	1	1	22.5519	0.490873	0.018855	0.004504	0.046488
1	1	1	1	20.06266	0.298192	0.062225	0.022821	0.260747
0	0	1	1	20.18224	0.302866	0.082929	-0.00093	0.570898
0	0	1	1	20.31514	0.337462	0.062708	0.009006	0.202467
0	0	1	1	20.23901	0.268231	0.02127	0.007426	0.012683
0	0	1	1	20.20411	0.228505	0.016178	0.006038	0.055104
0	0	1	1	20.21189	0.231216	0.000577	0.00523	-0.24477
0	0	1	1	20.11639	0.192124	-0.0518	-0.00011	-0.37943
0	0	1	1	20.18871	0.225521	0.022922	0.008055	0.311408
0	0	1	1	20.29538	0.268668	0.038735	0.006878	0.276252
0	0	1	1	20.36181	0.282189	0.043907	0.008171	0.128565
0	0	1	1	20.35699	0.272914	0.023237	0.008891	-0.14585
1	1	2	0.5	22.80189	0.583241	0.058916	0.014533	-0.00779
1	1	2	0.5	22.95955	0.598128	0.088053	0.010212	0.440061
1	1	2	0.5	23.04198	0.53402	0.126401	0.048427	0.127378
1	1	2	0.5	23.26427	0.364913	0.086981	0.017201	0.060031
1	1	2	0.5	23.31104	0.395511	0.076176	0.02082	0.178579
1	1	2	0.5	23.34344	0.405166	0.057436	0.01779	0.1156
1	1	2	0.5	23.41203	0.428716	0.071582	0.02103	-0.02557
1	1	2	0.5	23.73186	0.519946	0.05736	0.021813	0.147222
1	1	2	0.5	23.75582	0.515825	0.046501	0.009829	0.286064
1	1	2	0.5	23.83502	0.539055	0.051886	0.015267	0.094187
1	1	2	0.5	24.09639	0.632967	0.070101	0.016044	0.156786
1	3	2	0	21.3622	0.921597	-0.04228	0.000462	4.71084
1	1	1	1	22.53492	0.710644	0.089029	-0.00049	0.100477
1	1	1	1	22.71458	0.75771	0.06084	0.000565	-0.11486
1	1	1	1	22.84008	0.781191	0.018472	0.002935	-0.32118
1	1	1	1	23.24871	0.851002	0.01302	-0.00125	1.064738
1	1	1	1	23.31179	0.835821	0.049655	0.001324	0.085603
1	1	1	1	23.34604	0.830379	0.040042	0.006609	0.509518
1	1	1	1	23.57006	0.717746	0.041617	0.011219	-0.04442
1	1	1	1	23.49463	0.668727	0.064049	0.010319	0.284538
1	1	1	1	23.63435	0.560031	0.068298	0.009841	-0.20263
1	1	1	1	23.80459	0.751673	0.044938	0.002725	-0.04136
0	0	1	1	23.86927	0.755876	0.051976	0.006133	0.191534
1	3	3	1	20.96214	0.592531	0.046229	-0.00046	0.275765
1	3	3	1	21.14356	0.623647	0.044785	-0.00069	0.221297
1	3	3	1	21.32534	0.641177	0.055584	0.038907	0.317405
1	3	3	1	21.5331	0.659483	0.059475	-0.0009	0.215551
1	3	3	1	21.72059	0.679011	0.062975	0.051874	0.178753
1	3	3	1	21.78559	0.657526	0.069048	0.05433	0.136554
1	3	3	1	21.89583	0.638597	0.068996	0.060042	0.14549
1	1	1	0	21.87481	0.565844	0.073562	0.026224	0.25477
1	1	1	0	22.02074	0.533879	0.157683	0.003148	1.106207
1	1	1	0	21.88026	0.461877	0.053796	0.015659	-0.54795
1	1	1	0	21.93798	0.542232	0.030906	0.012081	-0.22818
1	1	1	0	21.98234	0.554532	0.021601	0.009585	0.32588
1	1	1	0	22.21641	0.638998	0.087191	0.008938	0.910671
1	1	1	0	22.20527	0.629441	0.029852	0.007645	-0.21274
1	1	1	0	22.13789	0.592735	0.030952	0.007036	-0.08303
1	1	1	0	22.47457	0.645625	0.06878	0.010238	0.110686
1	1	1	0	22.53379	0.620585	0.128629	0.030932	0.816181
1	1	1	0	22.76346	0.640735	0.079643	0.029378	-0.32131
1	1	1	0	24.45936	0.628626	0.104897	0.034064	0.138986
1	1	1	0	24.59992	0.684915	0.084565	0.012905	0.182021
1	1	1	0	24.61371	0.726412	0.068796	0.015187	-0.20492
1	1	1	0	24.63248	0.713165	0.061408	0.012266	-0.06993

1	1	1	0	24.60165	0.705863	0.059158	0.013639	0.413172
1	1	1	0	24.5866	0.648413	0.095314	0.025258	0.048549
1	1	1	0	24.91711	0.692674	0.094096	0.040302	-0.08651
0	0	1	0	24.88077	0.641826	0.090101	0.057	0.025304
1	1	1	0	24.92675	0.634951	0.029547	0.029098	-0.33941
1	1	1	0	25.04888	0.624646	0.069103	0.013953	-0.02074
0	0	1	0	25.11353	0.652784	0.070118	0.01577	0.289108
1	3	2	1	20.39731	0.539005	0.013103	-0.01049	-0.70878
1	3	2	1	20.62672	0.487196	0.115576	-0.01137	0.111946
1	1	2	1	20.57567	0.509186	-0.02559	-0.00439	0.712386
1	1	2	1	20.80626	0.507433	0.136072	0.008372	-0.38864
1	1	2	1	20.92805	0.411838	0.103954	0.012512	-0.04818
1	3	2	1	19.81509	0.047771	0.174016	-0.01157	-0.63793
1	1	1	1	22.82539	0.552295	0.125491	0.012258	0.017366
1	1	1	1	22.98868	0.583703	0.112846	0.02289	0.247925
1	1	1	1	23.0388	0.585088	0.09069	0.028228	0.276441
1	1	1	1	23.05343	0.604857	0.056829	0.01446	-0.02077
1	1	1	1	23.08819	0.622632	0.051072	0.01361	0.070644
1	1	1	1	23.10237	0.63716	0.046635	0.013293	-0.01446
1	1	1	1	23.10243	0.59952	0.048077	0.010974	-0.01313
1	1	1	1	23.10585	0.584291	0.051208	0.015357	-0.00402
1	1	1	1	23.02964	0.551272	0.086317	0.049731	0.018143
1	1	1	1	23.0626	0.546549	0.070073	0.013461	0.059035
1	1	1	1	23.10107	0.520735	0.068825	0.011266	0.022321
1	1	2	1	21.77635	0.118612	0.038698	-0.00082	0.411596
1	1	2	1	21.769	0.09589	0.032954	0.021396	-0.68305
1	1	1	1	22.83334	0.198052	0.020719	-0.00167	-0.06946
1	1	1	1	22.89964	0.206764	0.058826	0.008643	0.174027
1	1	1	1	23.00498	0.205673	0.03332	0.003885	0.078303
1	1	1	1	23.03063	0.195445	0.045002	0.05603	0.204856
1	1	1	1	23.06145	0.203637	0.038035	0.01634	0.027
0	0	1	1	22.07091	0.500605	0.061635	0.011029	-0.17212
0	0	1	1	22.07841	0.50469	0.058504	0.010407	-0.0328
0	0	1	1	22.16849	0.517492	0.062314	0.008808	-0.0952
0	0	1	1	22.22792	0.510574	0.07127	0.019525	0.020793
0	0	2	1	22.27545	0.50906	0.055022	0.006787	0.028042
0	0	2	1	22.3073	0.499235	0.055179	0.006162	0.125282
0	0	2	1	22.33544	0.51282	0.054275	0.007277	-0.02945
1	3	3	1	22.70149	0.58189	-0.05163	-0.00056	-0.208
1	1	3	1	22.70476	0.579747	0.027935	-0.00659	-0.1598
1	1	3	1	22.65688	0.329257	0.016711	-0.00447	-0.38025
0	0	3	1	22.62073	0.271201	0.044527	0.009875	0.435833
1	3	1	0	21.43046	0.800425	0.058875	0.011332	0.249357
1	3	1	0	21.63204	0.831932	0.054849	-0.00876	0.357027
1	3	1	0	21.6087	0.864294	0.013072	0.0004	-0.18796
1	3	1	0	21.48093	0.357514	0.045369	0.004214	2.234602
1	3	1	0	21.85039	0.358434	0.048578	0.013694	0.624555
1	3	1	0	22.03981	0.389018	0.057042	0.007571	0.413751
0	0	1	1	21.13176	0.616548	0.123889	0.0107	0.134088
0	0	1	1	21.81097	0.549598	0.172934	0.041045	1.001271
0	0	1	1	22.08273	0.486293	0.132913	0.079274	-0.19299
0	0	1	1	22.26236	0.33369	0.191207	0.061375	0.691118
0	0	1	1	22.5246	0.391013	0.155281	0.031492	1.054099
0	0	1	1	22.53648	0.191736	0.109678	0.032703	-0.58217
1	1	1	1	20.90704	0.489089	0.087182	0.014943	0.083211
0	0	1	1	20.90404	0.459617	0.04358	0.009755	0.164409
0	0	1	1	20.98478	0.49287	0.03946	0.013932	0.103337
0	0	1	1	21.0366	0.408573	0.146309	0.016617	0.084569

1	1	1	1	21.07297	0.433665	0.022322	0.015568	0.084131
1	1	1	1	21.13111	0.469842	0.027208	0.009177	0.19581
1	1	1	1	21.24048	0.20341	0.021496	0.010668	0.144969
0	0	1	1	21.24355	0.205142	0.009778	0.004918	0.075985
0	0	1	1	21.31254	0.245769	0.017532	0.006597	0.07377
0	0	1	1	21.25682	0.302799	-0.08226	0.003863	0.039625
1	1	2	0.5	21.51182	0.509441	0.072783	0.015011	-0.07808
1	1	2	0.5	21.70366	0.553281	0.088713	0.025336	0.245155
1	1	2	0.5	21.87495	0.560508	0.09339	0.022222	0.08477
1	1	2	0.5	22.1321	0.402334	0.058878	0.017786	0.101264
1	1	2	0.5	22.19979	0.39572	0.066271	0.019108	0.222204
1	1	2	0.5	22.21474	0.361625	0.072728	0.02159	0.025936
1	1	1	1	22.34243	0.39723	0.064891	0.024526	0.309652
1	1	1	1	22.45326	0.41415	0.079045	0.024831	0.031752
1	1	1	1	22.59457	0.46281	0.083502	0.024999	0.146847
1	1	1	1	22.61078	0.434334	0.063058	0.020517	0.114713
1	1	1	1	22.57012	0.406954	0.09195	0.034784	-0.05342
1	3	1	1	22.10819	0.824725	0.036671	0.009783	-0.00032
1	3	1	1	22.62598	0.90267	0.020738	0.006018	0.173115
1	3	1	1	22.62814	0.902448	0.039582	0.008134	0.332368
1	1	1	1	22.88624	0.909504	0.032936	0.007639	-0.08378
1	1	1	1	23.03971	0.871188	0.09026	0.021238	0.214141
1	1	1	1	23.33068	0.875725	0.054596	0.015888	-0.06112
1	3	1	1	23.47404	0.894146	0.03534	0.011136	-0.03277
1	3	1	1	23.51836	0.891875	0.025602	0.008456	-0.03248
1	3	1	1	23.59452	0.893923	0.040848	0.008287	0.314601
1	3	1	1	23.48906	0.90639	0.012866	0.009184	-0.02407
1	1	1	1	23.44865	0.942555	-0.01059	0.008487	-0.01779
1	1	2	0.5	20.91226	0.433778	0.029366	0.011625	0.177158
1	1	2	0.5	20.98125	0.461519	0.043921	0.012977	0.253851
1	1	2	0.5	21.2229	0.402419	0.079478	0.054892	0.220853
1	2	2	0.5	21.62929	0.369253	0.038528	0.016438	0.202772
1	2	2	0.5	21.71971	0.363497	0.059937	0.018691	0.295898
1	2	2	0.5	21.8157	0.291579	0.140022	0.024102	0.344189
1	2	2	0.5	21.91948	0.333635	0.082926	0.026337	0.28431
1	2	2	0.5	21.85788	0.326526	0.093515	0.026862	0.110676
0	0	2	0.5	21.92196	0.330339	0.102293	0.034654	0.050625
1	1	2	1	21.30096	0.547522	0.072143	0.011938	0.545993
1	1	2	1	21.6179	0.614032	0.07041	0.014707	0.429515
1	1	2	1	22.13415	0.540462	0.025518	0.003841	-0.07877
1	1	2	1	22.04958	0.48827	0.026179	0.006685	-0.16421
1	1	2	1	22.2408	0.568845	0.022708	0.004158	0.161568
1	1	2	1	22.28155	0.566319	0.035983	0.011421	0.141154
1	1	2	1	22.71734	0.42384	0.022297	0.01124	0.177728
0	0	2	1	22.66724	0.404609	-0.00155	0.004064	-0.13957
0	0	2	1	22.74148	0.438952	0.018339	0.001337	0.228869
0	0	2	1	22.71983	0.440981	-0.00991	0.001652	-0.28068
0	0	1	0	24.38735	0.673638	0.058839	0.013207	0.029743
0	0	1	0	24.50681	0.699119	0.056685	0.015043	0.119207
0	0	1	0	24.51835	0.697933	0.044693	0.011822	-0.02109
0	0	1	0	24.53579	0.690336	0.052176	0.01123	0.088655
0	0	1	0	24.55323	0.699793	0.06046	0.012899	0.333654
0	0	1	0	24.58651	0.679633	0.072673	0.016507	0.078241
0	0	1	0	24.60175	0.64913	0.061525	0.013322	-0.04478
0	0	1	1	22.3584	0.716865	0.047033	0.015484	-0.06375
0	0	1	1	22.61851	0.678064	0.056182	0.012526	0.50944
0	0	1	1	22.76029	0.674383	0.039854	0.012805	0.022824
0	0	1	1	22.81258	0.667241	0.040648	0.000801	-0.10625

0	0	1	1	22.74905	0.695863	-0.02126	0.000166	-0.13393
0	0	1	1	22.84769	0.698007	0.02719	0.000963	-0.16124
0	0	1	1	23.03573	0.749556	0.026625	0.005072	0.092534
1	1	1	1	22.95467	0.681431	0.021362	0.016876	-0.15685
1	1	1	1	22.77201	0.578814	0.060405	0.056053	0.019399
1	3	1	1	22.5795	0.525514	-0.00457	0.006432	-0.23473
1	3	1	1	22.49784	0.478091	0.018635	-0.00201	-0.1606
1	3	1	0	21.21343	0.908105	0.090681	0.114593	-0.04608
1	3	1	0	21.15779	0.985499	-0.02961	-0.00286	0.080673
1	1	1	1	23.27819	0.506601	0.099587	0.008658	-0.02976
1	1	1	1	23.45345	0.582863	0.020723	-0.00074	0.221416
1	1	1	1	23.59309	0.632972	0.030585	0.00634	0.349932
0	0	1	1	23.55643	0.619783	0.040147	0.008394	-0.00429
0	0	1	1	23.5246	0.605539	0.036412	0.008991	0.018359
0	0	1	1	23.56035	0.617978	0.03821	0.01015	-0.0494
1	1	1	1	23.49786	0.591402	0.038391	0.008642	0.016735
1	1	1	1	23.42417	0.548808	0.043745	0.007295	0.147941
1	1	1	1	23.38155	0.484243	0.079163	0.013718	0.263555
1	1	1	1	23.36751	0.43902	0.078238	0.018373	0.080858
1	1	1	1	23.34081	0.387366	0.070306	0.012176	-0.08294
0	0	1	0	21.61836	0.512269	0.019543	0.00761	-0.02202
0	0	1	0	21.67847	0.510451	0.015241	0.00491	-0.14817
0	0	1	0	21.8206	0.307543	0.002292	0.011058	-0.37225
0	0	1	0	21.96726	0.397654	0.003891	0.003066	0.489381
0	0	1	0	21.90937	0.343636	0.040769	0.005465	0.574328
0	0	1	0	21.97202	0.385164	0.015522	0.000822	0.037843
0	0	1	0	21.95054	0.215984	-0.00875	0.000089	-0.26985
1	3	3	0.666667	21.67372	0.569946	0.127646	0.032866	0.305725
1	3	3	0.666667	21.87253	0.573074	0.130462	0.044485	0.141341
1	3	3	0.666667	22.10004	0.650325	0.103263	0.039407	0.118115
1	3	3	0.666667	22.27423	0.69753	0.060041	0.029135	0.057371
1	3	3	0.666667	22.24292	0.657899	0.077495	0.020785	0.186297
1	1	1	1	22.88247	0.73991	0.061739	0.010965	-0.01398
1	3	1	1	23.05647	0.802886	0.005286	0.008504	0.058335
1	3	1	1	22.95245	0.786381	0.056212	0.00446	0.059873
1	3	1	1	22.81231	0.798017	0.001204	0.007951	0.012617
1	3	1	1	22.78717	0.81094	0.060788	0.013113	-0.20918
1	3	1	1	23.07504	0.497908	0.005355	0.007146	0.070591
1	3	1	1	23.04919	0.481793	0.022973	0.007634	0.162174
1	3	1	1	23.05329	0.470834	0.032125	-0.00251	-0.06693
1	3	1	1	22.97704	0.391103	0.065245	0.013037	0.134062
1	3	1	1	22.26028	0.506401	0.038362	0.014998	-0.05381
0	0	1	0	20.09595	0.900279	0.064643	-0.02313	0.173119
0	0	1	0	20.02873	0.876357	0.052052	-0.0293	-0.01751
0	0	1	0	20.91168	0.444749	0.148481	-0.02965	-0.03253
1	1	1	0	22.28992	0.546562	0.218472	0.07711	-0.17037
1	1	1	0	22.15319	0.344296	0.204465	0.042528	0.091867
1	1	1	1	21.88715	0.47032	0.05493	0.018101	0.282897
0	0	1	1	21.88172	0.428606	0.074507	0.021662	0.16295
0	0	1	1	22.17061	0.370148	0.070288	0.028187	0.093123
0	0	1	1	22.15966	0.350768	0.013621	0.019777	-0.12564
0	0	1	1	22.1977	0.332865	0.060048	0.01114	0.416588
0	0	1	1	22.29734	0.375751	0.075436	0.016609	0.140544
0	0	1	1	22.18482	0.368274	0.022273	0.0203	0.042431
0	0	2	1	22.9607	0.54105	0.085847	0.024241	0.093245
0	0	2	1	23.31769	0.514447	0.075989	0.021558	0.445456
0	0	2	1	23.58221	0.587678	0.065874	0.026122	0.232478
0	0	2	1	23.56545	0.511931	0.058427	0.010893	-0.0705

0	0	2	1	23.66637	0.476021	0.10716	0.022572	0.84145
0	0	2	1	23.85983	0.469273	0.154797	0.048225	0.075549
0	0	2	1	24.00533	0.43405	0.133624	0.05984	-0.02032
0	0	2	1	24.41055	0.565124	0.085714	0.015786	-0.16754
0	0	2	1	24.55955	0.591677	0.07958	0.018635	0.222197
0	0	2	1	24.67866	0.579514	0.103707	0.023068	0.263221
0	0	2	1	24.66648	0.480077	0.12586	0.031255	0.120006
1	3	2	0.5	20.59038	0.288744	0.07887	-0.0026	1.451476
1	3	2	0.5	20.44165	0.279354	-0.06588	0.008328	-0.04291
1	3	1	1	22.07429	0.400904	0.054115	0.012451	0.002822
1	3	1	1	22.17458	0.409554	0.06728	0.016626	0.201625
0	0	1	1	21.84983	0.364706	0.093192	0.0231	0.934865
1	2	1	1	22.1197	0.254085	0.166373	0.041936	-0.25915
1	2	1	1	22.09475	0.217494	0.113584	0.025572	-0.26756
1	2	1	1	22.09397	0.187676	0.062972	0.033802	-0.32878
0	0	1	1	22.3015	0.252362	0.071485	0.02617	0.000148
0	0	1	1	22.46893	0.192427	0.172081	0.140781	0.086846
0	0	1	1	22.54715	0.222939	0.089595	0.017581	0.205676
0	0	1	1	22.43277	0.187512	0.021382	0.011003	0.077253
0	0	1	1	22.45069	0.233246	0.012619	0.026262	-0.03985
1	1	3	0.666667	21.14482	0.150101	0.027705	-5.8E-05	0.12115
1	1	3	0.666667	21.16845	0.146696	0.05029	0.000178	0.19478
1	1	3	0.666667	21.15566	0.131975	0.028355	0.016804	0.172292
1	3	3	0.666667	21.15847	0.120434	0.02336	0.003242	-0.02119
1	3	3	0.666667	21.1421	0.091442	0.023913	0.01036	-0.1139
1	3	3	0.666667	21.16413	0.101957	0.023374	0.009844	-0.15942
1	1	3	0.666667	21.20302	0.118318	0.026526	0.014113	-0.10548
1	1	3	0.666667	21.25221	0.126399	0.041933	0.01436	-0.01959
1	1	3	0.666667	21.28288	0.145664	0.052371	0.013664	0.091248
1	3	3	0.666667	21.29253	0.122	0.046333	0.011863	0.109011
1	3	3	0.666667	21.30583	0.117292	0.055737	0.014497	-0.11899
1	3	1	1	20.46902	0.542895	0.096579	0.021165	0.017867
1	3	1	1	20.45336	0.519036	0.08691	0.025708	-0.11741
1	3	1	1	20.73105	0.587803	0.091466	0.023743	-0.04269
1	3	1	1	20.75448	0.564832	0.097162	0.020743	0.175171
1	3	1	1	20.73631	0.617763	-0.03583	0.016992	-0.37481
1	3	1	1	20.4938	0.49663	0.023202	-0.00543	0.060977
1	3	1	1	20.32027	0.407269	0.009211	0.009629	-0.15172
1	3	1	1	20.4336	0.403054	0.070463	0.012688	-0.14316
1	3	1	1	20.4577	0.332032	0.075567	0.013412	-0.19067
1	3	1	1	20.48552	0.346324	0.069445	0.014693	0.02373
1	3	1	1	20.49712	0.315948	0.074831	0.015182	0.043529
1	1	2	1	22.53906	0.283789	0.150979	0.036979	0.25345
1	1	2	1	22.68445	0.251936	0.157171	0.002554	0.214379
1	1	2	1	22.89964	0.380577	0.066889	0.007066	0.071242
1	1	2	1	23.00096	0.426835	0.063385	0.018318	0.2209
1	1	2	1	23.09774	0.439355	0.070786	0.024168	0.063235
1	1	2	1	23.21517	0.458677	0.075538	0.023664	0.192611
1	1	1	1	21.89261	0.088008	0.058494	0.006634	0.609528
1	1	1	1	22.47986	0.123299	0.039123	0.008386	0.100438
1	1	1	1	22.62205	0.18213	0.082038	0.015525	1.675276
0	0	1	1	22.66263	0.155156	0.087803	0.019029	0.121086
0	0	1	1	22.77592	0.148965	0.10576	0.021517	0.650793
1	3	2	0.5	19.23907	0.701429	0.032618	0.011758	-0.68512
1	3	2	0.5	19.02187	0.570914	0.015814	0.001869	-0.54607
1	1	2	0.5	23.64268	0.616607	0.169442	0.053128	0.7256
1	1	2	0.5	23.87778	0.64364	0.166107	0.052185	0.282991
1	1	2	0.5	23.9169	0.585301	0.163457	0.051752	0.073877

1	1	2	0.5	23.94607	0.554658	0.170332	0.051041	0.070619
1	1	2	0.5	24.11075	0.557476	0.154571	0.055914	0.048881
1	1	2	0.5	24.08765	0.520631	0.146714	0.053317	0.150864
1	1	3	0.666667	22.17257	0.679407	0.023584	0.008844	-0.05786
1	1	3	0.666667	22.37645	0.694897	0.033581	0.006812	1.032034
1	1	3	0.666667	22.52891	0.732262	0.030039	0.006054	-0.17485
1	3	3	0.666667	22.6448	0.757514	0.024496	0.013861	-0.08972
1	1	3	0.666667	22.66867	0.759816	0.023753	0.007197	0.49003
1	1	3	0.666667	22.47918	0.699903	0.03542	0.007441	0.187095
1	1	3	0.666667	22.63357	0.623515	0.028167	0.008093	-0.06729
1	1	3	0.666667	23.01005	0.54585	0.028313	0.008258	0.147251
1	1	3	0.666667	22.41875	0.155767	0.148507	0.004914	-0.10763
1	1	3	0.666667	22.42496	0.261653	0.048133	0.011123	-0.08532
1	3	3	0.666667	22.42665	0.246639	0.041527	0.012586	0.023109
0	0	3	0.666667	22.62156	0.558313	-0.03824	-0.00353	-0.41358
0	0	3	0.666667	22.6417	0.56891	0.032303	0.000232	0.32158
0	0	3	0.666667	22.71174	0.565356	-0.02739	0.010937	0.113839
0	0	3	0.666667	22.71404	0.562795	0.044863	-0.00101	-0.05762
0	0	3	0.666667	22.74735	0.624988	-0.01389	0.008737	-0.13586
0	0	3	0.666667	22.77235	0.744106	-0.0665	-0.00235	-0.25124
1	3	3	0.666667	22.59074	0.845331	-0.04798	-0.00958	0.428435
1	3	3	0.666667	22.42192	0.866457	-0.00769	0.003131	0.866068
1	3	1	1	18.34322	0.801263	0.055331	0.007339	4.271049
1	3	1	1	18.18666	0.753993	0.028511	0.006787	-0.36861
1	3	1	1	20.94171	0.406538	0.07257	0.008586	0.094572
1	3	1	1	21.07109	0.420792	0.087599	0.01398	0.266448
1	3	1	1	21.21178	0.437067	0.113249	0.017708	0.3394
1	3	1	1	21.48955	0.508913	0.117947	0.026141	0.239117
1	3	1	1	21.78308	0.348826	0.105913	0.027553	0.188418
1	3	1	1	21.82987	0.322215	0.125097	0.023762	0.149601
1	3	1	1	22.3223	0.325854	0.105879	0.028199	0.19298
1	1	1	1	22.41008	0.33139	0.091717	0.020796	0.037617
0	0	1	1	22.56986	0.404608	0.068761	0.021824	0.147373
1	1	1	1	22.65357	0.427057	0.066274	0.016536	0.213539
1	1	1	1	22.72946	0.417056	0.078096	0.018756	0.143338
0	0	1	1	22.60049	0.757537	0.033494	0.007584	-0.01028
0	0	1	1	22.58315	0.689439	0.047435	0.008882	-0.03724
1	1	1	1	22.55986	0.636584	0.097866	0.011242	0.145805
1	1	1	1	22.79086	0.570881	0.091935	0.022595	0.336246
0	0	1	1	22.89605	0.582089	0.067501	0.015832	0.072554
1	1	1	0	21.14563	0.577123	0.032482	0.005319	-0.18941
1	1	1	0	21.48615	0.437568	0.021575	0.000624	0.127724
1	1	1	0	21.6687	0.497079	0.011976	-0.00142	0.531903
1	1	1	0	21.62282	0.507756	0.000987	0.001101	-0.29415
1	1	1	0	21.36867	0.368835	0.047396	0.02476	-0.32762
0	0	1	0	21.67527	0.550035	-0.00864	0.004129	-0.2441
0	0	1	0	22.0627	0.44153	0.026206	-0.00474	0.315995
0	0	1	0	22.37723	0.575834	-0.04239	-0.0078	0.511532
1	1	1	0	22.63109	0.489586	0.017067	-0.00625	-0.07946
1	1	1	0	22.57156	0.455677	0.014876	-0.00115	0.209304
1	1	1	0	22.58133	0.529494	-0.04972	-0.0078	0.09884
1	1	1	1	22.06685	0.411955	0.128908	0.005656	-0.05163
1	1	1	1	22.37068	0.529591	0.066158	0.012673	0.129145
1	1	1	1	22.39459	0.581373	0.053402	0.005456	0.224237
1	1	1	1	22.64548	0.664765	0.053716	0.004576	0.134706
1	1	1	1	22.71092	0.638025	0.117128	0.011589	0.126365
1	1	1	1	22.77242	0.688463	0.067972	0.011836	0.037882
1	1	1	1	22.21968	0.493966	0.110976	0.014902	-0.54222

1	1	1	1	22.21494	0.508463	0.057464	0.012799	-0.35364
1	1	1	1	22.07228	0.458716	0.041004	-0.0005	-0.14695
1	1	1	1	22.08133	0.414846	0.080647	-0.00486	0.457831
1	1	1	1	22.07283	0.403929	0.067453	0.006172	0.008889
0	0	1	1	22.78017	0.692302	0.041977	0.010168	-0.09787
0	0	1	1	23.00591	0.742828	0.03649	0.010585	0.497465
0	0	1	1	23.25532	0.782833	0.054511	0.009236	0.370931
0	0	1	1	23.36898	0.786704	0.045783	0.051749	0.036857
0	0	1	1	23.59143	0.820735	0.027101	0.005817	0.095382
0	0	3	0.333333	23.7603	0.839223	0.024829	0.00484	0.058868
0	0	3	0.333333	23.91714	0.850784	0.019336	0.005605	-0.01242
0	0	3	0.333333	24.03481	0.799793	0.021663	0.003976	-0.08986
0	0	1	1	24.34203	0.833367	0.025886	0.006722	0.22517
0	0	1	1	24.66455	0.793027	0.025774	0.009287	0.130887
0	0	1	1	24.80832	0.802801	0.025978	0.007785	0.060077
0	0	2	1	20.80428	0.487439	0.040202	-0.00051	-0.03956
0	0	2	1	21.03606	0.537472	0.097996	0.006805	0.343834
0	0	2	1	20.93315	0.507828	-0.00293	0.001899	0.018165
0	0	2	1	20.97157	0.516949	0.040221	-0.01568	-0.09992
0	0	2	1	20.88823	0.467702	0.034167	-0.0006	0.039088
0	0	2	1	20.80898	0.419601	0.034777	0.002284	-0.11732
0	0	3	0.666667	20.72302	0.359944	0.029937	0.004691	-0.07324
0	0	2	1	20.7355	0.31752	0.064463	0.0068	0.058622
0	0	2	1	20.80806	0.359973	0.039356	0.007034	0.077459
0	0	2	1	21.04016	0.476193	0.038993	0.010623	0.223622
0	0	2	1	21.24703	0.559957	0.031549	0.009053	0.018678
1	3	1	1	23.31208	0.631938	0.027244	0.007511	0.321121
0	0	2	0.5	21.81802	0.636761	0.029991	0.010523	0.066818
0	0	2	0.5	21.92133	0.662045	0.020419	0.009298	-0.21323
0	0	2	0.5	22.14982	0.72921	0.01672	0.004402	-0.16707
0	0	2	0.5	22.28303	0.775814	0.014522	0.004941	0.083055
1	1	2	0.5	22.43488	0.782031	0.039621	0.018207	0.348229
1	1	2	0.5	22.45691	0.793113	0.01636	0.00352	0.573582
1	1	2	0.5	22.41839	0.635504	0.012874	0.007481	0.002586
1	1	2	0.5	22.78007	0.412793	0.023757	0.005215	0.050394
1	1	2	0.5	23.12172	0.546566	0.023181	0.007801	0.169926
1	3	1	1	20.58394	0.435803	0.016865	-0.00218	0.463128
1	3	1	1	20.69003	0.485429	0.01507	-0.00817	-0.01733
1	3	1	1	20.67524	0.56077	-0.08128	-0.01375	-0.15884
0	0	1	1	22.0345	0.508313	0.136177	0.015166	0.278794
0	0	1	1	22.46134	0.501537	0.105338	0.029957	0.167281
0	0	1	1	22.65598	0.339115	0.073852	0.019476	0.121238
0	0	1	1	22.78684	0.394432	0.079057	0.012358	0.1925
1	1	1	1	22.91885	0.385845	0.083599	0.015686	0.165019
1	1	1	1	23.1913	0.311359	0.06724	0.018942	0.276165
1	1	1	1	23.47707	0.338337	0.080234	0.016287	0.912081
1	1	1	1	23.74137	0.408727	0.093976	0.024065	0.277493
1	1	1	1	24.01195	0.337321	0.08487	0.022715	0.283943
1	1	1	1	24.1892	0.391393	0.078939	0.022397	0.251727
1	1	1	1	24.41698	0.465351	0.061878	0.02015	0.142893
0	0	1	1	20.88798	0.314328	0.105476	0.018002	0.1272
0	0	1	1	21.01128	0.314711	0.123207	0.02016	0.369817
0	0	1	1	21.1655	0.050958	0.121576	0.025045	0.24157
0	0	1	1	21.27807	0.046664	0.124676	0.024642	-0.13064
0	0	1	1	21.37975	0.051771	0.135031	0.026682	0.073082
0	0	1	1	21.33976	0.035121	0.019773	0.027108	0.00617
0	0	1	1	21.29423	0.040835	-0.01108	0.002916	-0.09346
0	0	1	1	21.26797	0.057158	0.014121	0.002104	-0.20266

0	0	1	1	21.66651	0.032365	0.028377	0.009733	0.203012
0	0	1	1	21.63989	0.028886	0.0307	0.00583	-0.04048
0	0	1	1	21.63472	0.048438	0.035492	0.002599	-0.15953
1	1	1	1	22.11587	0.460641	0.109597	0.020187	0.162608
1	1	1	1	22.41938	0.371882	0.113191	0.025108	0.16504
1	1	1	1	22.6155	0.430823	0.12825	0.048139	0.412348
0	0	1	1	22.73629	0.438072	0.140589	0.028506	0.41563
0	0	1	1	23.04685	0.594261	0.153301	0.035485	0.193292
1	1	1	1	23.28215	0.607745	0.156654	0.042195	0.132371
1	1	1	1	23.45846	0.499962	0.133396	0.034623	0.052102
1	1	2	1	23.56388	0.520813	0.100688	0.02576	0.054746
1	1	2	1	23.7928	0.583199	0.095417	0.025564	0.154075
1	1	2	1	23.94899	0.55473	0.097334	0.02472	0.117769
1	1	2	1	23.90184	0.505989	0.073534	0.027247	0.056076
0	0	1	1	22.34152	0.619115	0.042089	0.002845	0.1471
0	0	1	1	22.29888	0.735912	0.036094	-0.00574	0.370077
0	0	1	1	22.40334	0.744291	0.039221	0.011286	0.207847
0	0	1	1	22.62916	0.590967	0.047009	0.006425	0.513837
0	0	1	1	22.68229	0.572383	0.062241	0.01707	0.050501
1	1	1	1	22.61005	0.454877	0.007583	0.001389	-0.19927
1	1	1	1	22.62231	0.453534	0.013417	0.001017	-0.14197
1	1	1	1	21.76752	0.373002	0.010204	0.009487	-0.35812
1	1	1	0	20.47128	0.527215	0.064821	0.007418	0.044719
1	1	1	0	20.46239	0.468737	0.083579	0.024507	0.06806
1	1	1	0	20.4844	0.368599	0.087895	0.030007	0.069675
1	1	1	0	20.6239	0.37949	0.091886	0.013824	0.049217
1	1	1	0	20.68801	0.343494	0.112728	0.014067	0.2976
1	1	1	0	20.92693	0.412451	0.112413	0.014381	0.190912
1	1	1	0	21.18033	0.299625	0.096859	0.014028	-0.02223
1	1	1	0	21.2641	0.283405	0.090564	0.012706	0.286998
1	3	1	0	21.20079	0.293872	0.01348	-0.00123	-0.07507
1	3	1	0	21.13416	0.329473	0.033252	-0.00042	-0.0189
1	1	1	1	21.37984	0.404922	0.075039	0.018365	0.284612
1	1	1	1	21.62483	0.303369	0.101056	0.012546	0.279327
1	1	1	1	21.8086	0.455444	0.125141	0.050168	0.179719
1	1	1	1	22.2359	0.462255	0.100875	0.024665	0.287766
0	0	1	1	22.21703	0.418139	0.138291	0.033586	0.069674
1	1	1	1	22.56364	0.464941	0.148857	0.040156	0.224836
1	1	1	1	22.98177	0.522894	0.077364	0.03473	0.480329
1	1	1	1	22.86277	0.471677	0.074819	0.022853	0.135497
1	1	1	1	22.96612	0.379669	0.10181	0.029462	-0.12066
1	1	1	1	23.09476	0.459314	0.101648	0.03424	0.281958
0	0	1	1	23.01179	0.498728	0.008375	0.025164	-0.0027
1	3	1	0	21.70455	0.463043	0.103027	0.02489	0.044605
1	3	1	0	22.12535	0.36158	0.07049	0.026082	0.300895
1	3	1	0	22.29706	0.436049	0.041222	0.005079	0.083441
1	3	1	0	22.28631	0.415746	0.045922	0.003859	-0.20311
1	3	1	0	22.90806	0.563304	0.055551	0.018473	1.284477
1	3	1	0	23.005	0.577223	0.059989	0.011044	0.203384
1	3	1	0	23.36696	0.646517	0.044947	0.012778	0.374472
1	2	1	0	23.50095	0.653374	0.030244	0.01065	-0.05908
1	2	1	0	23.54318	0.639548	0.068606	0.007079	0.131491
0	0	1	0	23.63858	0.649485	0.054442	0.011135	0.098155
0	0	1	0	23.70406	0.623445	0.072719	0.02199	0.120989
0	0	2	0.5	21.70561	0.358468	0.061158	0.022032	0.154354
0	0	2	0.5	21.788	0.368238	0.065356	0.013847	0.220637
0	0	2	0.5	21.82208	0.366066	0.048706	0.013288	-0.1741
0	0	2	0.5	21.85886	0.358006	0.058467	0.012714	-0.09101

0	0	2	0.5	21.95546	0.410884	0.038173	0.011269	-0.01405
0	0	2	0.5	22.03714	0.452526	0.030135	0.010114	0.003054
0	0	2	0.5	22.13146	0.499017	0.027099	0.008765	-0.16461
0	0	1	1	22.34362	0.409932	0.042835	0.010626	0.446036
0	0	1	1	22.49312	0.476114	0.045695	0.010923	0.143398
0	0	1	1	22.40901	0.410835	0.04241	0.011049	0.034522
0	0	1	0	20.97185	0.445529	0.125413	0.020043	0.13299
0	0	1	0	21.17107	0.440175	0.146319	0.033751	0.12243
0	0	1	0	21.3131	0.36897	0.192271	0.025336	0.297023
0	0	1	0	21.54607	0.321196	0.186527	0.018689	0.183489
0	0	1	0	21.88669	0.396271	0.172416	0.026404	0.236318
0	0	1	0	22.42723	0.376897	0.107803	0.019172	0.13364
1	1	1	0	22.44381	0.364657	0.049012	0.01577	0.046069
1	1	1	0	22.60865	0.363667	0.081901	0.020377	0.116367
1	1	1	0	22.69658	0.354848	0.077448	0.021998	0.032776
1	1	1	0	22.44924	0.337303	0.045096	0.013182	-0.1344
1	3	2	1	22.46269	0.269204	0.060107	0.016325	-0.46673
1	3	2	1	22.54081	0.312377	0.033159	0.010479	0.128953
1	3	2	1	22.67349	0.397221	0.012978	0.004353	0.116587
1	3	2	1	22.70322	0.391713	0.032656	-0.00159	0.259224
1	3	2	1	22.70274	0.341681	0.087809	0.022407	0.107072
1	3	2	1	22.7824	0.445308	-0.01732	0.00365	-0.04781
1	3	2	1	22.92717	0.458494	0.07453	0.010464	0.069678
1	3	2	1	23.03646	0.379615	0.143815	0.032137	0.511852
1	3	2	1	23.11149	0.412912	0.056319	0.018375	-0.00397
1	3	2	1	19.21298	0.128966	0.041838	0.018863	0.375002
1	1	2	1	19.30885	0.129889	0.09252	0.031193	0.400682
1	1	2	1	19.34063	0.096866	0.073712	0.019868	0.03878
1	1	2	1	21.53578	0.113418	0.095672	0.017015	0.392311
1	1	2	1	21.70565	0.144142	0.098272	0.022207	0.231272
1	1	2	1	21.82271	0.189031	0.072112	0.017288	0.003111
1	1	2	1	21.85334	0.18261	0.05104	0.00953	0.08627
1	1	2	1	21.86318	0.168798	0.042459	0.010524	0.067517
1	1	2	1	21.99194	0.251565	0.033126	0.008963	0.040308
0	0	1	1	21.35188	0.601227	0.053531	-0.0029	0.15934
0	0	1	1	21.36536	0.574671	0.053071	0.018956	0.073598
0	0	1	1	21.58553	0.57743	0.036136	-0.00216	0.440768
0	0	1	1	21.72953	0.605584	0.032205	0.007976	-0.04947
0	0	1	1	21.71718	0.606321	0.026091	0.008613	0.209329
0	0	1	1	22.56246	0.576849	0.027227	0.010706	-0.012
0	0	1	1	22.84012	0.43915	0.023297	0.012248	0.351624
1	1	2	1	21.06546	0.552917	0.04318	-0.00982	-0.64888
1	1	1	1	20.72149	0.294369	0.022662	-0.00925	-0.26187
1	1	1	1	20.66393	0.162718	0.132623	0.138647	-0.31989
1	1	1	1	20.57285	0.080902	0.086715	0.050104	0.015199
1	1	1	1	22.40869	0.222388	0.008903	0.062013	0.212507
1	1	1	1	21.92998	0.203635	0.01637	-0.00173	0.084259
1	1	1	1	21.81032	0.189452	0.019934	-0.00226	0.05805
0	0	1	1	21.77433	0.190456	0.023737	-0.00177	-0.04671
1	1	1	1	21.87435	0.171625	0.023171	0.001886	0.013046
1	3	3	1	22.3834	0.489957	0.080169	0.02673	-0.01588
1	3	3	1	22.61309	0.530617	0.067636	0.031398	0.058393
1	3	3	1	22.6352	0.615014	0.036935	0.011229	-0.33539
1	3	3	1	22.69079	0.627349	0.024363	0.003485	0.248101
1	3	3	1	22.80541	0.653719	0.043893	0.003193	0.519527
1	3	3	1	22.69745	0.472928	0.090957	0.020082	-0.00093
0	0	1	1	22.92617	0.515375	0.164466	0.020785	0.063391
0	0	1	1	23.081	0.447132	0.206726	0.05586	0.399494

0	0	2	0.5	23.2257	0.489718	0.16442	0.123446	0.138854
0	0	2	0.5	23.29137	0.465131	0.161615	0.006588	0.057587
0	0	2	0.5	23.4034	0.462144	0.179806	0.040941	0.122355
0	0	1	1	23.54913	0.478381	0.173285	0.042801	0.124071
1	1	1	1	23.9352	0.338821	0.110914	0.028176	0.049915
1	1	1	1	24.11998	0.396014	0.117867	0.028792	0.224544
1	1	1	1	24.17971	0.400541	0.129273	0.029212	0.125999
0	0	1	1	24.26395	0.414624	0.140669	0.02883	0.080648
0	0	1	1	24.38236	0.449623	0.083756	0.023039	0.043456
1	1	1	1	19.45166	0.776176	-0.05185	-0.00634	-0.36282
1	1	1	1	19.53973	0.783851	0.037896	-0.00915	0.305591
1	1	1	1	19.92574	0.786286	0.040364	-0.00384	0.425598
1	1	1	1	19.99058	0.83017	0.017205	0.001865	1.032346
1	1	1	1	20.08257	0.746974	0.113316	0.002017	-0.17128
1	3	1	1	21.9183	0.650694	0.031956	0.024877	-0.01537
1	3	1	1	22.13905	0.687844	0.04632	0.062499	-0.14258
1	3	1	1	22.38853	0.644886	0.012488	0.009877	-0.0688
1	3	1	1	22.48679	0.642092	0.048435	0.004144	0.850882
0	0	1	1	22.6414	0.634862	0.082	0.01042	0.281005
0	0	1	1	22.8533	0.684932	0.052957	0.01231	-0.22272
0	0	1	1	22.90254	0.679161	0.06582	0.007866	0.368076
0	0	2	1	21.44694	0.29143	0.106024	0.03819	1.206284
0	0	2	1	22.51813	0.184125	0.095032	0.073525	0.834196
0	0	2	1	22.96501	0.372243	0.147534	0.033495	1.025609
0	0	2	1	23.17798	0.459509	0.096261	0.027121	0.924761
0	0	2	1	23.31449	0.475279	0.107793	0.019478	0.109691
0	0	2	1	23.55746	0.326567	0.116234	0.019484	0.227112
0	0	2	1	23.75724	0.231038	0.100585	0.030439	0.060832
0	0	2	1	23.88338	0.260356	0.106475	0.023268	0.291123
0	0	2	1	23.95156	0.216523	0.155595	0.034999	0.338157
0	0	2	1	24.15043	0.309862	0.10729	0.042686	-0.0035
0	0	2	1	24.11376	0.267358	0.057445	0.023423	-0.10812
1	1	1	0	21.37025	0.585898	0.020122	-0.00298	-0.63312
1	1	1	0	20.83601	0.488718	0.022131	-0.00162	-0.67276
1	1	1	0	20.94829	0.515808	0.052282	-0.00076	0.757447
1	1	1	0	20.99252	0.555968	0.008282	-0.00134	-0.24085
0	0	1	0	20.67852	0.493079	-0.01627	0.01574	7.944525
1	1	1	0	20.8219	0.209802	0.15405	0.034201	0.039874
1	1	1	0	20.87063	0.190357	0.139998	0.034964	0.006309
1	1	1	0	20.98053	0.214734	0.123076	0.035165	0.037147
1	3	1	1	20.65414	0.545525	0.053068	0.01963	0.170529
1	3	1	1	20.66426	0.608776	0.061553	0.03293	0.263031
1	3	1	1	20.80334	0.616176	0.064848	0.033436	0.29024
1	3	1	1	20.92482	0.611286	0.068331	0.037429	0.11099
1	3	1	1	21.2895	0.415592	0.075478	0.026983	0.081775
1	3	1	1	21.35606	0.417626	0.072212	0.026388	-0.14927
1	3	1	1	21.42862	0.416657	0.059174	0.023429	-0.07753
1	3	1	1	21.60154	0.483343	0.042222	0.017908	-0.08399
1	3	1	1	22.33287	0.633539	0.053768	0.011526	0.331043
1	1	2	1	22.0368	0.570319	0.106697	0.02688	0.361656
1	1	3	0.333333	20.25493	0.529584	0.021897	-0.01458	0.888151
1	1	3	0.333333	20.22983	0.484437	0.050574	-0.00874	0.274487
1	1	3	0.333333	20.69035	0.322517	0.022849	-0.02	0.623
1	1	3	0.333333	20.84267	0.40517	0.020694	-0.01328	-0.1959
1	1	3	0.333333	20.8788	0.421126	0.014231	-0.01252	0.097323
1	1	3	0.333333	21.01333	0.521283	-0.01893	-0.0113	-0.10328
0	0	3	0.333333	21.34024	0.649421	0.015312	-0.01627	-0.0498
1	1	1	0	19.30154	0.11352	0.138781	-0.00169	0.36019

1	1	1	0	20.2844	0.125751	0.21279	0.05597	0.22187
0	0	1	0	20.49152	0.134571	0.182987	0.050826	0.260845
0	0	1	0	20.90347	0.365735	0.1575	0.047452	0.307491
0	0	1	0	20.97484	0.301239	0.139365	0.037751	0.151269
1	1	1	0	21.30801	0.387428	0.163413	0.045626	0.342486
1	1	2	1	20.55766	0.579533	0.048176	-0.0029	0.064025
1	1	2	1	21.24516	0.485245	0.059066	-0.00778	1.526922
1	1	2	1	21.43394	0.544745	0.055761	0.008959	0.014926
1	1	2	1	22.87227	0.511935	0.132599	0.014061	1.609171
1	1	2	1	22.97054	0.465229	0.133866	0.034011	0.03423
1	1	2	1	23.15472	0.567267	0.111706	0.012856	0.162355
1	1	2	1	23.63429	0.713722	0.062425	0.010883	0.13013
1	1	2	1	23.79386	0.726217	0.066415	0.019644	0.569148
1	1	2	1	23.88088	0.602939	0.098896	0.024235	0.358408
1	1	2	1	23.91591	0.58413	0.083498	0.020914	-0.00649
1	1	2	1	20.44154	0.536683	0.083657	0.015989	-0.14625
1	1	2	1	20.42636	0.503801	0.042043	0.004913	0.035782
1	1	2	1	20.40116	0.474936	0.052762	0.024456	0.068852
1	1	2	1	20.42233	0.450943	0.051245	0.009276	-0.2074
1	1	2	1	20.40902	0.444406	0.076281	0.007514	0.232496
1	1	2	1	20.41855	0.450462	0.039421	0.005603	0.003879
0	0	2	1	20.66656	0.421612	0.074934	0.007824	-0.00958
0	0	2	1	20.73311	0.364945	0.119025	0.008744	0.037705
0	0	2	1	20.77624	0.346101	0.033066	0.022739	-0.11257
0	0	2	1	20.75456	0.377145	0.014733	0.001892	-0.12515
0	0	2	1	20.63659	0.363094	-0.06469	0.002705	0.105762
0	0	2	0.5	23.49457	0.562223	0.022531	0.010356	1.223783
0	0	2	0.5	23.97927	0.607632	0.064079	-0.00157	3.305069
0	0	2	0.5	24.21997	0.608553	0.055338	-0.00141	0.245127
0	0	2	0.5	24.33646	0.623765	0.052985	-0.00103	0.192724
0	0	2	0.5	24.63704	0.676055	0.049659	0.007874	0.496242
0	0	2	0.5	24.79912	0.65985	0.060294	0.007259	0.250898
1	1	2	0.5	24.90299	0.622708	0.062764	0.008068	0.183596
1	1	2	0.5	25.10466	0.627074	0.048646	0.005889	-0.08813
1	1	2	0.5	25.24416	0.623284	0.05912	0.007177	0.36174
1	1	2	0.5	25.40836	0.586464	0.063244	0.006572	0.107542
1	1	2	0.5	25.57188	0.622118	0.043751	0.004857	0.037481
1	3	1	0	21.11768	0.650289	0.131345	0.032578	0.20815
1	3	1	0	21.20046	0.612365	0.129384	0.043285	-0.00672
1	3	1	0	21.46211	0.605628	0.139198	0.029604	0.192611
1	3	1	0	21.52619	0.571082	0.103648	0.033517	0.052076
1	3	1	0	21.5609	0.504803	0.133366	0.031442	0.030614
1	3	1	0	21.5603	0.451832	0.123595	0.032401	-0.06573
1	3	1	0	21.78246	0.543455	0.045281	0.022756	-0.0762
1	3	1	0	23.4857	0.718433	0.068866	0.029031	3.93089
1	3	1	0	23.6008	0.654438	0.104064	0.024997	0.249228
1	3	1	0	23.69174	0.658819	0.107518	0.027338	0.114345
1	3	1	0	23.67586	0.591967	0.110416	0.028797	-0.0665
1	1	2	1	21.42761	0.698097	0.043392	0.000067	0.647016
1	1	2	1	21.67811	0.796147	0.029214	0.004875	0.013235
1	1	2	1	21.95377	0.835035	0.018087	0.005366	-0.22209
1	1	2	1	21.977	0.832925	0.019888	-0.00121	0.281352
1	1	2	1	21.92276	0.823659	0.024749	0.003976	0.28752
1	1	2	1	21.86422	0.81632	0.036375	0.00482	0.035152
1	1	2	1	22.03064	0.744665	0.027434	0.008551	-0.22261
1	1	2	1	21.9483	0.731505	0.011209	0.004478	0.123254
1	1	2	1	21.96945	0.729298	0.02018	0.000699	-0.2302
1	1	2	1	21.95538	0.730876	0.011961	0.006674	0.020515

1	3	2	1	21.88299	0.745099	-0.01685	-0.00053	-0.19701
1	3	1	1	20.47195	0.216742	0.067764	0.019772	-0.0865
1	3	1	1	20.52767	0.226431	0.066547	0.022102	-0.15969
1	3	2	1	20.53027	0.754144	0.059649	0.018889	0.021053
1	3	2	1	21.0445	0.531756	0.038933	0.011706	0.011206
1	1	1	1	21.45524	0.148545	0.074771	0.019784	-0.15088
1	1	1	1	21.53646	0.157388	0.115113	0.035345	0.130343
1	1	2	1	21.53484	0.277487	0.050294	0.017451	0.041395
1	1	2	1	21.50549	0.207539	0.101266	0.025291	0.111429
1	1	2	1	21.58925	0.125928	0.185645	0.021087	0.230013
1	1	2	1	21.59042	0.163405	0.034164	0.024521	0.263899
1	1	2	1	21.71446	0.251713	0.085163	0.032046	0.21453
1	1	2	1	21.80718	0.269167	0.118619	0.037006	0.205191
1	1	2	1	22.05514	0.345582	0.155083	0.050345	0.150187
1	1	2	1	22.2655	0.156099	0.172335	0.051198	0.222296
1	1	2	1	22.28198	0.045299	0.207666	0.052575	0.247467
1	1	2	1	22.42132	0.133446	0.180818	0.063184	0.05799
1	3	2	1	22.40035	0.063758	0.180564	0.054198	0.031275
1	3	2	1	19.53807	0.749759	0.063668	-0.01466	0.020674
1	3	2	1	22.83891	0.820864	0.063331	0.008542	0.386
1	1	2	1	23.10835	0.699535	0.06131	0.01534	0.162308
1	1	2	1	22.94254	0.672675	0.009982	0.01429	-0.10142
1	1	2	1	23.02143	0.688303	0.052049	0.008185	0.171459
0	0	2	1	23.22823	0.7302	0.038834	0.008792	-0.01923
0	0	2	1	23.30063	0.669052	0.06534	0.015675	0.031646
0	0	2	1	23.3341	0.567824	0.038884	0.011032	0.045407
0	0	2	1	23.6606	0.68151	0.018333	0.00429	0.409832
0	0	2	1	23.634	0.72888	0.033771	0.009603	0.014561
0	0	2	1	23.59532	0.716488	0.031852	0.009171	-0.02034
0	0	1	1	22.19938	0.327891	0.143515	0.038913	0.138608
0	0	1	1	22.27985	0.26329	0.157122	0.030053	0.186149
0	0	2	1	20.31556	0.225479	0.018543	0.000279	-0.14398
0	0	2	1	20.27358	0.257784	-0.06396	0.002271	-0.04072
0	0	2	1	20.28237	0.24139	0.033967	0.005205	0.031719
0	0	2	1	20.36091	0.283591	0.038975	0.008074	0.096598
0	0	2	1	20.87351	0.236192	0.108564	0.039955	0.795446
0	0	2	1	20.93772	0.249104	0.079624	0.019779	0.071659
0	0	2	1	21.42455	0.192183	0.110488	0.024214	0.221378
0	0	2	1	21.67976	0.325349	0.099987	0.025249	0.020229
0	0	2	1	21.60356	0.230831	0.090935	0.014425	-0.00761
0	0	2	1	21.75768	0.262049	0.079181	0.02013	0.068473
0	0	2	1	21.73078	0.220253	0.076924	0.017145	-0.00473
1	3	1	1	20.98263	0.367091	0.040682	0.010126	0.836191
1	3	1	1	20.798	0.212311	0.079717	0.01245	0.024852
1	3	1	1	20.87878	0.249332	0.079622	0.015003	-0.10123
1	3	1	1	20.95517	0.283824	0.073894	0.018448	0.089816
1	3	1	1	21.09164	0.349334	0.073866	0.019119	0.293975
1	3	1	1	21.15947	0.352202	0.084261	0.020832	-0.11991
1	3	1	1	21.19457	0.37746	0.061063	0.0179	0.300961
1	3	1	1	21.31537	0.429502	0.040713	0.016718	0.036135
1	3	1	1	21.37775	0.424451	0.053848	0.011712	0.147166
1	3	1	1	21.26686	0.335104	0.051406	0.017399	-0.20285
1	1	1	1	21.38003	0.373664	0.052088	0.017371	0.035837
1	1	1	1	21.10772	0.538747	0.037016	0.005941	0.502753
1	1	1	1	21.39586	0.650603	0.009275	0.004596	0.334014
1	1	1	1	21.60205	0.718379	0.006411	0.000875	0.366609
1	1	1	1	21.75118	0.759488	0.016234	0.027903	0.154697
0	0	1	1	22.14108	0.820694	0.02645	0.00485	-0.39811

0	0	1	1	22.25827	0.829735	0.014399	0.00158	0.228541
0	0	1	1	22.90758	0.565006	0.022214	0.004914	1.166828
1	1	1	1	22.82295	0.479715	0.058611	0.001738	1.906716
1	1	1	1	23.08879	0.421524	0.054389	0.013217	0.347927
1	1	1	1	23.05653	0.393524	0.043062	0.011264	0.500512
1	1	1	1	22.81748	0.507479	0.073342	0.065855	0.121
1	1	1	1	23.19464	0.636483	0.056085	0.013327	0.168782
1	1	1	1	23.41816	0.673338	0.056621	0.013084	0.187972
1	1	1	1	23.64622	0.712883	0.052034	0.013456	0.133199
1	1	1	1	23.90652	0.664781	0.047549	0.011576	0.228194
1	1	1	1	24.20712	0.697847	0.044953	0.010165	0.207479
1	1	1	1	24.37984	0.689883	0.045654	0.008262	0.241334
1	1	1	1	24.67544	0.624627	0.050615	0.008802	0.201213
1	1	1	1	24.92308	0.694305	0.040468	0.007938	0.178428
1	1	1	1	24.98802	0.691238	0.049924	0.010464	0.141855
0	0	1	1	21.93151	0.456773	0.004029	0.00624	-0.08886
0	0	1	1	22.10765	0.501437	0.082094	0.012316	0.770338
0	0	1	1	22.08112	0.508017	0.037091	0.021246	-0.10615
0	0	1	1	22.14664	0.542321	0.013921	-0.00653	-0.03962
0	0	1	1	22.20347	0.433898	0.038836	-0.00358	0.052529
0	0	1	1	22.1124	0.364714	0.059842	0.015505	0.100771
0	0	1	1	22.10163	0.381362	0.00742	0.000472	-0.24268
0	0	1	1	22.16854	0.402198	0.031344	0.002565	0.002764
0	0	1	1	22.21565	0.391254	0.055638	0.012425	0.29876
0	0	1	1	22.27998	0.405239	0.058598	0.009859	0.170006
0	0	1	1	22.16647	0.301123	0.044802	0.023651	-0.15101
1	1	2	0	22.25715	0.377838	0.083987	0.01847	0.2326
1	1	2	0	22.35683	0.434047	0.044945	0.015303	-0.19965
0	0	2	0	22.39949	0.455622	0.029555	-0.00272	-0.16235
0	0	2	0	22.45793	0.473255	0.040484	0.011696	0.003228
0	0	2	0	22.80764	0.380015	0.024229	0.00136	-0.19771
1	1	2	0	22.92156	0.436261	0.024751	0.002128	0.180898
1	1	2	0	23.05112	0.373742	0.028938	0.005647	0.632242
1	1	2	0	23.1138	0.378119	0.04792	0.008677	0.212802
1	1	2	0	23.10575	0.368063	0.01404	0.009122	-0.23438
1	1	2	1	23.20844	0.600211	0.04274	0.04274	-0.1004
1	1	2	1	23.05521	0.590366	-0.04203	0.005741	-0.23008
1	1	2	1	23.08913	0.584959	0.042972	0.003247	0.065081
1	1	2	1	23.36388	0.490031	0.039287	0.006842	0.724133
1	1	2	1	23.39882	0.478645	0.053025	0.011155	0.092746
1	1	2	1	23.41374	0.499043	0.014426	0.007687	-0.10356
1	1	1	1	21.64531	0.359564	0.095506	-0.00032	0.1469
1	1	1	1	21.80869	0.365051	0.122272	0.011046	0.35384
0	0	1	1	22.08313	0.322345	0.071226	0.016262	0.265703
0	0	1	1	22.23576	0.378464	0.036925	0.006634	0.326891
1	1	2	1	23.14465	0.684474	0.116704	0.116704	0.4718
1	1	2	1	23.49766	0.592003	0.122185	0.033888	0.104544
0	0	2	1	23.70117	0.627238	0.07787	0.034075	0.041481
1	1	2	1	23.74867	0.589179	0.08946	0.025624	0.301467
1	1	2	1	24.00325	0.498608	0.078494	0.022189	0.195382
1	1	2	1	24.10313	0.48781	0.085628	0.024506	0.12883
0	0	3	0.333333	21.79285	0.347135	0.07959	0.07959	0.2041
0	0	3	0.333333	21.77044	0.31493	0.056055	0.011247	-0.10065
0	0	3	0.333333	21.87014	0.353417	0.064677	0.012152	0.101521
0	0	3	0.333333	22.10757	0.352663	0.038869	0.011074	-0.04622
0	0	3	0.333333	21.90483	0.184282	0.041921	0.013551	-0.13996
0	0	2	0.5	21.68999	0.320717	0.067803	0.019141	0.008581
0	0	2	0.5	21.74048	0.344174	0.056924	0.020041	-0.05512

1	1	2	1	21.95116	0.081601	0.111378	0.111378	0.3873
1	1	2	1	22.06213	0.110623	0.074373	0.024437	-0.07786
1	1	2	1	22.0724	0.099188	0.062046	0.018688	0.17662
1	1	2	1	22.54071	0.370841	0.086307	0.015056	1.040151
1	1	2	1	22.70721	0.370808	0.128291	0.035339	0.506496
1	1	2	1	22.85469	0.333779	0.173462	0.052128	0.285693
0	0	1	1	23.71075	0.769429	0.040818	0.020584	0.5947
0	0	1	1	23.88932	0.754404	0.061109	0.017178	0.354569
0	0	1	1	24.00282	0.729171	0.034254	0.021472	-0.07723
0	0	1	1	24.1223	0.737645	0.016132	0.001293	-0.10414
0	0	1	1	20.67698	0.640637	0.167408	0.167408	0.7217
0	0	1	1	21.47463	0.127952	0.071091	0.022023	0.180543
0	0	1	1	21.54886	0.162299	0.047215	0.011013	-0.00448
0	0	1	1	21.68085	0.24934	0.036977	0.010602	0.027787
0	0	1	1	21.74489	0.282454	0.037325	0.01048	0.171746
0	0	1	1	21.72577	0.250171	0.039223	0.011389	0.022991
0	0	1	1	22.39491	0.380796	0.054209	0.012067	0.761431
0	0	1	1	22.4893	0.386374	0.077207	0.014171	0.339612
0	0	1	1	22.40926	0.349884	0.076655	0.018653	-0.11952
0	0	1	1	22.75041	0.505558	0.068728	0.017597	0.251761
0	0	1	1	24.93986	0.795412	0.050467	0.050467	0.14
0	0	1	1	25.35965	0.841351	0.044218	-0.00312	0.186708
0	0	1	1	25.93563	0.858431	0.048044	0.001065	0.448935
0	0	1	1	26.52332	0.845734	0.050281	0.003113	0.335778
0	0	1	1	26.85907	0.865973	0.040477	0.000896	0.585845
0	0	1	1	23.30467	0.406709	0.074805	0.057141	0.491
1	1	1	1	23.40335	0.451019	0.046859	0.015007	0.180463
0	0	1	1	23.43631	0.33191	0.066069	0.013632	0.057213
1	3	2	0.5	21.84932	0.228447	0.02939	0.008825	0.189968
1	3	2	0.5	21.82665	0.223054	0.043463	0.020063	0.035956
1	3	2	0.5	21.87952	0.255596	0.016687	0.003241	-0.05163
1	3	2	0.5	21.89542	0.275862	0.029354	0.002439	0.055225
1	1	1	1	22.04219	0.400106	0.060791	0.035211	-0.213
0	0	1	1	22.03798	0.398579	0.013686	0.008416	-0.21503
0	0	1	1	22.06624	0.41011	0.020441	0.003438	0.126579
0	0	1	1	22.07633	0.38166	0.036855	0.003108	0.171431
0	0	1	1	22.09263	0.366583	0.043064	0.00741	0.080291
0	0	1	1	22.06258	0.32069	0.040916	0.010739	-0.12123
0	0	1	1	22.13894	0.369783	0.009257	0.007617	-0.1336
0	0	1	1	22.216	0.433649	-0.01301	-0.0129	-0.0595
0	0	1	1	22.22853	0.443016	0.02603	0.006175	0.179927
1	1	2	1	22.54818	0.667858	0.090293	0.092893	-0.073
1	1	2	1	22.81957	0.576792	0.095168	0.020687	0.049396
1	1	2	1	22.86079	0.55168	0.076158	0.026602	0.070269
1	1	2	1	23.23559	0.487084	0.064553	0.021765	0.112151
1	1	2	1	23.29063	0.477494	0.059065	0.01275	0.343355
1	1	2	1	23.45794	0.510722	0.061948	0.00894	0.124785
1	1	2	1	23.57763	0.502806	0.093372	0.025703	0.238569
1	3	2	1	23.72654	0.53304	0.066796	0.016614	0.129423
1	3	2	1	23.81029	0.529622	0.066585	0.015424	0.108909
1	1	1	1	24.93903	0.81897	0.026121	0.007708	-0.05737
0	0	1	1	24.87272	0.802759	0.02597	0.005152	-0.06532
0	0	1	1	24.98362	0.815241	0.0291	0.005142	0.170899
0	0	1	1	24.87479	0.78927	0.036549	0.00868	0.067805
0	0	2	0.5	22.08693	0.292118	0.105792	0.05302	0.1667
0	0	2	0.5	22.28367	0.333028	0.120983	0.0061	0.294839
0	0	2	0.5	22.38733	0.322823	0.124949	0.033231	0.162817
0	0	2	0.5	22.5038	0.300542	0.144993	0.03594	0.118533

0	0	2	0.5	22.6033	0.289333	0.111167	0.022353	0.040601
0	0	2	0.5	22.65938	0.274779	0.09904	0.020764	0.171871
0	0	2	0.5	22.97218	0.391448	0.072855	0.017588	0.208988
0	0	2	0.5	23.09372	0.419635	0.068863	0.0193	0.21079
1	1	2	0.5	23.16014	0.394594	0.066926	0.020572	-0.02172
0	0	1	1	20.98322	0.547845	0.123824	0.118406	0.0977
0	0	1	1	21.55247	0.413002	0.069527	0.011307	0.194667
0	0	1	1	21.63386	0.432417	0.086661	0.012589	0.21628
0	0	1	1	21.7615	0.445303	0.09694	0.015374	0.12832
0	0	1	1	21.75521	0.416056	0.09403	0.015629	-0.02657
0	0	1	1	21.75919	0.376995	0.066247	0.017189	-0.09169
0	0	1	1	21.68823	0.340422	0.02994	0.007824	-0.09628
0	0	2	0	22.78002	0.4198	0.080023	0.026043	0.1079
0	0	2	0	23.27761	0.644679	0.034796	0.027824	0.081501
0	0	2	0	23.2111	0.61087	0.047716	0.016666	0.090001
1	1	2	0	22.33047	0.484779	0.050172	0.017021	0.169483
1	1	2	0	22.32496	0.447609	0.043055	0.005591	-0.13469
1	1	3	0.666667	22.03607	0.318314	0.058539	0.043678	0.031
1	1	3	0.666667	22.11158	0.352332	0.043229	0.008905	0.120273
1	1	3	0.666667	22.21087	0.397353	0.038068	0.009816	0.021615
0	0	3	0.666667	22.6592	0.472127	0.020502	0.008041	-0.13294
1	1	3	0.666667	22.14108	0.19887	0.218387	0.071503	0.111305
1	1	3	0.666667	22.23352	0.162197	0.201044	0.065174	0.108587
1	1	3	0.666667	22.48386	0.371171	0.120629	0.049743	0.055057
1	1	3	0.666667	22.46154	0.288827	0.137822	0.041695	0.196747
1	1	3	0.666667	22.49934	0.269269	0.154769	0.043852	0.187594
1	1	1	1	21.50312	0.331714	0.075508	0.053211	0.0543
1	1	1	1	21.61108	0.371844	0.072827	0.033001	0.064905
1	1	1	1	22.03156	0.209527	0.055544	0.034061	0.146192
1	1	1	1	22.10843	0.236202	0.06559	0.030986	0.229089
1	1	1	1	22.19483	0.270892	0.056809	0.029708	-0.07365
1	3	1	1	22.2331	0.298223	0.052834	0.021785	-0.01919
0	0	1	1	23.82928	0.781111	0.028838	0.028838	0.3027
0	0	1	1	24.26988	0.795595	0.029234	0.003766	0.520274
0	0	2	0.5	21.32383	0.587549	0.134145	0.134145	-0.0652
1	1	2	0.5	22.01649	0.271564	0.06152	0.009752	0.076031
1	1	2	0.5	21.90296	0.191662	0.011206	0.000961	-0.25307
1	1	2	0.5	21.90622	0.213053	0.014238	0.000835	0.210193
1	1	2	0.5	21.85015	0.168839	0.013459	0.001651	-0.19386
0	0	2	0.5	21.82132	0.153631	0.002365	0.001156	-0.12915
1	3	2	0.5	21.74201	0.14372	-0.03623	-0.00476	-0.11579
1	3	2	0.5	21.73974	0.122199	0.008388	-8.7E-05	-0.06085
1	3	2	0.5	21.76292	0.104212	0.042908	0.000679	-0.07658
0	0	3	0.666667	21.96423	0.218199	0.038968	0.00446	0.173166
1	1	2	0.5	21.96611	0.255022	0.119318	0.095018	0.3174
1	1	2	0.5	21.90655	0.186587	0.027135	0.008421	-0.19927
1	1	2	0.5	21.94474	0.209116	0.035324	0.006683	0.073908
1	1	2	0.5	22.37734	0.457709	0.05008	0.004719	0.148523
1	1	2	0.5	22.44072	0.319717	0.037434	0.009597	-0.03242
1	1	2	0.5	22.50646	0.315097	0.057822	0.013333	0.190616
1	1	2	0.5	22.87989	0.361351	0.056817	0.016371	0.385637
1	1	2	0.5	22.95713	0.347351	0.076654	0.019511	0.285455
1	1	2	0.5	23.18865	0.382557	0.105459	0.01998	0.062008
0	0	2	0	22.14404	0.229091	0.124823	0.031801	0.3078
0	0	2	0	22.23257	0.161087	0.197533	0.052496	0.290446
0	0	2	0	22.44279	0.278083	0.094803	0.040592	-0.08722
0	0	2	0	22.58141	0.342707	0.057238	0.019223	-0.02616
0	0	2	0	22.67501	0.356852	0.08101	0.031473	0.192323

0	0	2	0	22.70704	0.338174	0.092619	0.026398	-0.01486
0	0	2	0	22.69475	0.297214	0.07586	0.013479	0.0096
0	0	2	0	22.79193	0.269054	0.150499	0.035146	0.329598
0	0	2	0	23.08972	0.41859	0.10356	0.035325	0.044299
0	0	2	0	23.12783	0.420911	0.069932	0.0216	-0.08697
0	0	2	0.5	21.71675	0.083794	0.04292	0.024126	-0.00786
0	0	2	0.5	21.87488	0.184189	0.047843	0.013408	0.29014
0	0	2	0.5	21.94155	0.184754	0.067109	0.01198	0.107879
0	0	2	0.5	22.13933	0.315421	0.060082	0.01479	0.285878
0	0	2	0.5	22.09924	0.287897	0.067517	0.019924	0.181821
0	0	2	0.5	22.32014	0.384125	0.056817	0.015375	-0.09613
0	0	2	0.5	22.2286	0.33906	0.049035	0.014058	-0.12216
1	1	2	0.5	22.26577	0.388995	0.014536	0.010746	-0.09656
1	1	2	0.5	22.25059	0.479792	-0.04217	0.012322	0.47473
1	1	3	0.333333	23.04069	0.529786	0.053067	0.070986	0.2705
1	1	3	0.333333	23.1018	0.539962	0.048792	0.013309	0.274648
1	1	3	0.333333	23.36585	0.636895	0.032793	0.010634	0.005606
1	1	3	0.333333	23.58994	0.666635	0.044885	0.01087	0.16068
1	1	3	0.333333	23.76021	0.738091	0.039672	0.011796	0.133374
1	1	3	0.333333	23.95331	0.727207	0.040067	0.011282	0.087097
1	1	3	0.333333	24.10359	0.767377	0.016204	0.009441	-0.10994
1	3	3	0.333333	24.12515	0.757179	0.027442	0.005922	0.140656
1	3	3	0.333333	24.05207	0.729386	0.055733	0.007059	-0.12598
0	0	1	0	21.94904	0.381957	0.098881	0.022393	0.223953
0	0	1	0	22.4043	0.301065	0.073905	0.026038	0.35638
0	0	1	0	22.58862	0.376736	0.087537	0.020494	0.271453
0	0	1	0	22.70406	0.397205	0.098996	0.022864	0.192379
0	0	1	0	22.86969	0.428908	0.111337	0.02703	0.200575
0	0	3	0.333333	25.29813	0.547161	0.073369	0.073369	0.1614
0	0	3	0.333333	25.43154	0.591417	0.068126	0.018878	0.299309
0	0	3	0.333333	25.5297	0.599451	0.069203	0.019391	0.156564
0	0	1	0	22.66252	0.472406	0.067269	0.067269	0.0242
0	0	1	0	22.96315	0.519511	0.097506	0.02084	0.568901
0	0	1	1	24.28873	0.602485	0.097602	0.034228	0.676681
0	0	1	1	24.51098	0.534136	0.088323	0.014549	0.161273
0	0	1	1	24.58573	0.532433	0.097353	0.020272	0.171002
0	0	1	1	24.73563	0.548611	0.089293	0.017009	0.102297
1	1	2	0.5	21.3736	0.434908	0.043107	0.043107	0.0168
1	1	2	0.5	21.47699	0.450761	0.062066	0.0081	0.270696
1	1	2	0.5	21.47852	0.418047	0.056326	0.009167	-0.00669
1	1	2	0.5	21.50962	0.414891	0.039423	0.007022	-0.08455
0	0	1	1	22.06957	0.256628	0.060829	0.045203	0.0114
0	0	1	1	22.05082	0.231185	0.023499	0.021519	0.092029
0	0	1	1	22.05464	0.226165	0.022043	0.007002	-0.03985
0	0	1	1	22.0373	0.226376	-0.0051	0.005288	-0.24428
0	0	1	1	22.05839	0.232809	0.006993	0.0075	0.084619
0	0	1	1	22.0692	0.205412	0.035321	0.008955	-0.0185
0	0	1	1	22.05824	0.199425	0.005605	0.008551	-0.31834
0	0	1	1	22.05973	0.200979	0.016023	0.005221	-0.00312
0	0	1	1	22.05189	0.206088	-0.00981	-0.00255	0.018026
0	0	1	1	22.08333	0.207552	0.024946	0.007907	0.237655
0	0	1	1	23.61152	0.553829	0.071926	0.040743	0.2043
0	0	1	1	23.68782	0.550893	0.075672	0.018629	0.323286
0	0	1	1	23.97339	0.611703	0.056753	0.01719	0.099404
0	0	1	1	24.00345	0.584978	0.074738	0.017026	0.121725
1	1	1	1	21.27543	0.123446	0.048979	0.007715	0.0665
1	1	1	1	21.25398	0.076897	0.035214	-0.0039	-0.0328
1	1	1	1	21.401	0.137401	0.045393	0.001844	0.053447

1	3	1	1	21.4327	0.135923	0.041353	0.006634	0.32809
1	3	1	1	21.48996	0.171353	0.025606	0.001595	-0.03605
1	3	1	1	21.5153	0.186942	0.038106	0.001562	0.080309
1	3	1	1	21.56278	0.178595	0.052254	0.010109	0.042112
1	3	1	1	22.04554	0.289464	0.042517	0.01278	0.227034
1	1	1	1	22.10325	0.293253	0.043892	0.008452	0.216317
0	0	1	1	22.35159	0.292313	0.124759	0.003647	2.0608
0	0	1	1	22.28743	0.221602	0.07465	0.03321	-0.1907
0	0	1	1	22.27909	0.179197	0.070648	0.027258	0.060472
0	0	1	1	22.45411	0.275893	0.088143	0.028728	0.119218
0	0	1	1	22.43616	0.261398	0.070183	0.02504	-0.02074
0	0	1	1	22.39734	0.235124	0.055433	0.026567	0.003602
0	0	1	1	22.30797	0.185325	0.037191	0.028428	-0.067
0	0	1	1	22.22393	0.155496	0.022383	0.020533	-0.10406
1	3	2	0.5	21.3418	0.374997	0.05053	0.00381	-0.09332
1	1	2	1	21.24318	0.496534	0.07528	0.057697	0.4284
1	1	2	1	20.92	0.355645	-0.01821	-0.00345	-0.42082
1	1	2	1	21.25762	0.526	0.01714	0.00619	0.172737
0	0	2	1	21.76794	0.748156	-0.04556	-0.01083	1.059229
0	0	2	1	22.40678	0.218867	0.012063	0.01393	0.461085
0	0	2	1	22.56622	0.314181	0.008113	0.00096	0.059787
0	0	2	1	22.45091	0.267118	0.022393	0.001867	-0.0473
0	0	2	1	22.50519	0.322923	0.009415	-6.8E-05	-0.15827
1	1	1	1	20.41653	0.225698	0.123529	0.063738	0.3033
1	1	1	1	20.65474	0.22786	0.138082	0.026853	0.295165
1	1	1	1	20.83328	0.256314	0.152077	0.044692	0.349516
1	1	1	1	21.22462	0.33735	0.091277	0.03317	0.10073
1	1	1	1	21.43992	0.366578	0.073654	0.021827	0.009844
1	1	1	1	21.43313	0.321603	0.068089	0.019675	0.010244
1	1	1	0	21.13338	0.447086	0.071528	0.056801	-0.0518
1	1	1	0	21.68283	0.59131	0.088592	0.022679	1.029393
1	1	1	0	21.95777	0.658543	0.049579	0.008802	0.219484
1	1	1	0	22.03892	0.663736	0.048697	0.011058	-0.02346
1	1	2	0.5	20.94511	0.187946	0.096948	0.066642	0.0702
1	1	2	0.5	21.13493	0.273159	0.092104	0.017247	0.139088
1	1	2	0.5	21.41026	0.421818	0.060009	0.017287	0.262903
1	1	2	0.5	21.89384	0.584451	0.083122	0.015796	0.30753
1	1	2	0.5	22.19951	0.44775	0.072969	0.012919	0.313895
1	1	2	0.5	22.47904	0.525719	0.061673	0.012652	0.437615
1	1	2	0.5	22.51374	0.592181	-0.05909	0.013244	0.321136
1	1	2	0.5	22.61902	0.567978	0.087433	0.009242	0.156845
0	0	2	0.5	20.64688	0.510328	0.081935	0.071017	0.1946
0	0	2	0.5	20.81975	0.519312	0.07141	0.021142	0.113766
0	0	2	0.5	21.29257	0.274173	0.043287	0.019791	0.132868
0	0	2	0.5	21.42944	0.331834	0.048688	0.013242	0.14048
0	0	2	0.5	21.8186	0.411117	0.028352	0.0126	0.333442
0	0	2	0.5	21.89395	0.484981	-0.0347	0.006035	0.070134
1	1	3	1	20.34093	0.250106	0.089966	0.061522	0.1579
1	1	3	1	20.82756	0.162052	0.084624	0.034881	0.178098
1	1	3	1	21.06382	0.250388	0.121239	0.0345	0.393858
1	1	3	1	21.31591	0.391944	0.074955	0.031117	0.201067
1	1	3	1	21.45433	0.453611	0.07152	0.011229	0.176299
1	1	3	1	21.44255	0.406764	0.091891	0.023039	0.113515
1	1	1	1	21.44188	0.365812	0.143258	0.143258	-0.1432
1	1	1	1	21.88836	0.249316	0.048513	0.015017	-0.26387
1	1	1	1	21.9646	0.282565	0.032865	0.009019	-0.10545
1	1	1	1	22.09416	0.3373	0.046698	0.009463	0.391505
1	1	1	1	22.23447	0.362609	0.059849	0.012191	0.282348

1	1	1	1	22.41362	0.476113	0.079449	0.009982	0.423543
0	0	1	1	20.64778	0.102264	0.085009	0.04504	-0.0439
0	0	1	1	20.67083	0.121559	0.057229	0.016413	0.005565
0	0	1	1	20.6871	0.117717	0.071678	0.017831	0.096451
0	0	1	1	20.68334	0.108989	0.07099	0.017197	0.064298
1	1	1	1	21.33169	0.587982	0.054285	0.011489	0.0295
1	1	1	1	21.71725	0.488418	0.037006	0.010679	-0.04076
1	1	1	1	21.8501	0.553147	0.025324	0.006741	0.126631
1	1	1	1	21.79331	0.531838	0.024293	0.004209	0.022248
0	0	2	0	20.3093	0.155312	0.06404	0.044979	-0.0228
0	0	2	0	20.38027	0.185825	0.054793	0.014647	-0.04157
0	0	2	0	20.48286	0.245162	0.043596	0.010865	0.241899
0	0	2	0	20.52431	0.246778	0.049607	0.012648	0.111795
0	0	2	0	20.56212	0.24952	0.047703	0.010979	0.029026
0	0	1	0	20.06199	0.114601	0.175666	0.078634	0.0027
0	0	1	0	20.18337	0.09109	0.181489	0.056637	0.040221
0	0	1	0	20.24078	0.088917	0.126711	0.02512	-0.05688
0	0	1	0	20.55528	0.229548	0.092479	0.031603	0.013468
0	0	1	0	20.52988	0.132087	0.047774	0.015502	-0.31633
1	3	2	0.5	20.02027	0.145683	0.109271	0.109271	-0.219
1	3	2	0.5	20.01264	0.114003	0.059277	0	-0.22929
1	3	2	0.5	20.53344	0.175478	0.008475	0.002319	-0.41884
1	1	2	1	21.86425	0.335077	0.085295	0.085295	0.407
1	1	2	1	21.87501	0.289459	0.079552	0.022946	-0.06784
1	1	2	1	22.24194	0.44916	0.090463	0.026497	0.092944
0	0	2	1	22.68032	0.58873	0.046991	0.007676	0.246239
0	0	3	0.333333	21.01226	0.424466	0.070434	0.034658	0.0562
0	0	3	0.333333	21.1098	0.439853	0.073894	0.016694	0.179781
1	1	1	1	22.18825	0.406757	0.098486	0.029214	0.090085
1	1	1	1	22.24758	0.421864	0.057401	0.014398	-0.0954
0	0	1	1	20.5575	0.081705	0.128649	0.128649	-0.0025
0	0	1	1	20.67457	0.097093	0.112259	0.03354	0.020348
0	0	1	1	20.73013	0.069261	0.12508	0.041119	0.29103
0	0	1	1	20.88443	0.080459	0.131759	0.025484	0.089053
1	1	2	0	21.15022	0.405119	0.034149	0.015783	0.053662
0	0	1	1	20.83514	0.141795	0.082427	0.082427	0.1611
0	0	1	1	21.0149	0.233575	0.074659	0.022325	0.078321
0	0	1	1	21.21808	0.306667	0.070089	0.025497	0.474711
0	0	2	1	21.91392	0.477411	0.08148	0.017493	0.051711
0	0	2	1	22.19006	0.527351	0.067833	0.020685	0.192468
0	0	2	1	20.08178	0.2349	0.072458	0.034025	-0.056
0	0	2	1	20.80931	0.153551	0.033604	0.011312	0.234031
0	0	2	1	21.14602	0.35676	0.048017	0.006677	1.168849
0	0	2	1	21.56659	0.571412	0.021076	0.008872	0.223313
1	1	2	1	20.19458	0.235045	0.07934	0.07934	0.0989
1	1	2	1	20.16007	0.190597	0.041882	0.016475	-0.18095
1	1	2	1	20.30199	0.281934	0.031305	0.002887	0.233485
1	1	2	1	20.40651	0.323062	0.05168	0.011637	0.314628
1	1	2	1	20.49156	0.335947	0.051148	0.018429	0.116825
1	1	2	1	20.53266	0.328755	0.084739	0.022726	0.097794
0	0	1	1	20.77391	0.324397	0.089208	0.030491	0.21863
0	0	2	1	21.19103	0.438604	0.069699	0.010161	0.284114
0	0	2	1	21.16424	0.377878	0.081114	0.034071	0.024194
1	1	1	0	20.06543	0.124861	0.072194	0.058658	-0.0299
1	1	1	0	20.21361	0.225713	0.056511	0.014329	0.600187
1	1	1	0	20.31354	0.263815	0.050347	0.017544	0.128254
0	0	1	0	20.43638	0.318842	0.048304	0.011553	0.182153
0	0	1	0	21.4798	0.431987	0.069869	0.028836	-0.0318

1	1	1	0	21.61441	0.4696	0.058923	0.020578	0.140562
1	1	1	0	22.14856	0.677999	0.033119	0.021488	0.197253
1	1	1	0	22.06108	0.637841	0.027037	0.007157	0.214881
0	0	2	1	21.6639	0.430328	0.034952	0.02713	-0.5897
0	0	2	1	21.70057	0.453807	0.033668	0.004069	0.007614
1	1	2	1	21.25025	0.4518	0.09409	0.041254	0.1989
1	1	2	1	22.13619	0.459059	0.056136	0.017647	0.329137
1	1	2	1	22.68692	0.614256	0.0693	0.016826	0.995118
1	1	2	1	22.74388	0.584933	0.103421	0.02228	0.381015
0	0	2	0	22.39129	0.273024	0.152956	0.086853	0.5696
1	1	2	0	22.63953	0.283937	0.219377	0.056196	0.324722
1	1	2	0	22.88232	0.319639	0.104321	0.026543	-0.07514
1	1	1	0	21.96177	0.605925	0.052023	0.02954	-0.1294
1	1	1	0	22.17391	0.479312	0.038271	0.012304	0.05405
0	0	2	0.5	22.25667	0.497002	0.063542	0.014137	0.1844
0	0	2	0.5	22.65879	0.381617	0.044247	0.021669	0.001622
0	0	2	0.5	22.70093	0.398601	0.014896	0.015608	0.154714
0	0	2	0.5	22.81506	0.437213	0.018361	0.00289	0.103055
1	1	2	1	20.66232	0.407158	0.098285	0.044261	0.246
1	1	2	1	20.85553	0.429196	0.109122	0.010885	0.356876
0	0	2	1	21.0724	0.416532	0.143786	0.013169	0.564047
0	0	3	0.666667	21.24343	0.465045	0.071485	0.067296	0.2975
0	0	3	0.666667	21.45686	0.51818	0.042955	0.014649	0.400278
0	0	3	0.666667	21.65444	0.577477	0.064665	0.015384	0.27373
0	0	2	1	20.48259	0.20901	0.0842	0.045521	0.0881
0	0	2	1	20.62702	0.269581	0.105585	0.027773	0.424843
0	0	2	1	21.02533	0.242094	0.054172	0.014101	0.212196
0	0	2	1	21.20483	0.237708	0.060673	0.01164	0.050576
0	0	2	0	20.67329	0.169804	0.122323	0.039651	0.1305
0	0	2	0	20.9374	0.266387	0.118993	0.033692	0.240555
0	0	2	0	21.14412	0.310163	0.109672	0.033407	0.166824
0	0	2	0	21.20192	0.339208	0.098193	0.022701	0.126498
0	0	2	0	21.62064	0.48425	0.065662	0.019104	0.014906
1	1	1	1	20.01447	0.226672	0.105144	0.071906	0.0524
1	1	1	1	20.1264	0.281494	0.101957	0.02203	0.217245
1	1	1	1	20.15527	0.252662	0.089503	0.024034	0.110652
1	1	1	1	20.16118	0.243397	0.045059	0.004463	-0.11492
1	1	1	1	21.46529	0.686789	0.070503	0.070503	0.2478
1	1	1	1	21.91849	0.62275	0.045422	0.016758	0.500919
1	1	1	1	21.94427	0.620276	0.036513	0.011894	0.179527
1	1	1	1	20.58669	0.125458	0.180333	0.082738	0.2188
1	1	1	1	20.9662	0.300683	0.163244	0.054882	0.203882
1	1	1	1	21.33418	0.134564	0.176895	0.045653	0.061911
1	1	1	1	21.34889	0.113472	0.098344	0.031441	-0.04072
1	1	1	1	21.22319	0.10155	-0.06838	0.026153	-0.35302
1	1	1	1	21.18657	0.063099	0.008962	0.013881	-0.1506
0	0	2	0.5	21.86067	0.570029	0.095658	0.027187	0.8091
0	0	2	0.5	22.08638	0.587382	0.088445	0.027536	0.100134
0	0	2	0.5	22.29384	0.639763	0.047861	0.016635	-0.10848
1	1	2	1	21.14657	0.161126	0.123786	0.092214	0.1211
1	1	2	1	21.35953	0.240275	0.121342	0.027379	0.021482
1	1	2	1	21.56325	0.311376	0.126103	0.034057	0.101961
0	0	2	1	21.2806	0.116624	0.135141	0.135141	0.0807
0	0	2	1	21.73942	0.381901	0.078433	0.026989	0.178437
1	1	1	0	22.16026	0.166475	0.182939	0.046975	-0.00122
1	1	1	0	22.22636	0.161437	0.139381	0.051745	0.064726
1	1	1	0	22.28452	0.345126	0.035157	0.028424	-0.14771
1	1	1	1	22.38123	0.303976	0.130697	0.077532	-0.0109

1	1	1	1	22.45505	0.303236	0.157908	0.066204	0.038105
1	1	1	1	22.49656	0.299499	0.14872	0.070225	0.03293
1	1	1	1	22.5829	0.323263	0.158781	0.076238	0.111656
1	1	1	1	22.66471	0.316328	0.178408	0.07654	0.082602
0	0	2	0	20.55368	0.110214	0.068623	0.016307	-0.08132
0	0	2	0	20.64252	0.12303	0.047855	0.009825	0.003975
0	0	2	0	21.17746	0.41058	0.054099	0.011833	0.338649
0	0	2	0	21.47696	0.507448	0.034415	0.013192	0.157461
0	0	2	0	21.45779	0.485338	0.059118	0.011703	0.105251
0	0	2	1	23.12578	0.406344	0.189312	0.035072	0.3139
0	0	2	1	23.56638	0.519927	0.114284	0.029194	0.422217
0	0	2	1	23.85443	0.490831	0.081647	0.020525	0.045606
1	3	1	1	20.70433	0.256133	0.096394	0.043549	0.1456
1	3	1	1	20.78645	0.224355	0.107067	0.020833	0.048328
1	3	1	1	20.85377	0.192073	0.105505	0.014717	-0.04586
0	0	1	1	22.48504	0.51867	0.113481	0.086685	0.1829
0	0	1	1	22.60075	0.527812	0.114971	0.036017	0.19378
1	1	1	1	22.88336	0.500491	0.109546	0.036516	0.257571
1	1	1	1	20.02594	0.226363	0.145669	0.145669	0.0185
1	1	1	1	21.03412	0.357942	0.049804	0.011246	0.175996
1	1	1	1	21.11888	0.370073	0.050515	0.01236	0.514434
1	1	1	1	21.1987	0.361048	0.07591	0.017246	0.172541
1	3	2	0.5	21.36271	0.247977	0.062632	0.051239	-0.1989
1	1	2	0.5	23.37394	0.61412	0.140358	0.140358	0.519
0	0	2	0.5	23.49969	0.500148	0.217838	0.053141	0.593719
0	0	2	0.5	23.57899	0.505269	0.087253	0.035941	-0.19299
1	1	1	0	21.58177	0.118557	0.141304	0.092961	0.1419
0	0	2	0	20.27977	0.165435	0.062335	0.024538	-0.0523
0	0	2	0	20.58453	0.313495	0.0515	0.018674	0.207931
0	0	2	0	20.92583	0.457853	0.067046	0.011829	0.6168
0	0	1	1	21.75876	0.516067	0.070901	0.070901	0.3452
0	0	1	1	21.88766	0.523481	0.080981	0.018239	0.294065
0	0	1	1	22.1249	0.5804	0.04825	0.015563	0.119149
0	0	2	1	22.26596	0.428643	0.10848	0.059644	0.1344
0	0	1	1	20.16156	0.258037	0.070602	0.036848	0.1529
0	0	1	1	20.26458	0.312162	0.041183	0.01576	0.121362
0	0	1	1	20.50847	0.418821	0.043644	0.009929	0.400127
1	1	1	1	20.87169	0.112531	0.115125	0.085882	0.0922
1	1	1	1	20.95913	0.108425	0.122474	0.028115	0.020944
0	0	1	0	20.53964	0.247003	0.082759	0.053085	0.0162
1	1	1	0	20.56305	0.184821	0.096521	0.02806	0.165752
1	1	1	0	20.61799	0.202486	0.108859	0.029872	0.069645
0	0	1	0	20.73434	0.254835	0.07524	0.028658	0.082617
0	0	1	0	20.91421	0.321942	0.081323	0.025285	0.147971
0	0	1	0	20.68117	0.376294	0.079405	0.079405	0.1989
0	0	1	0	20.83145	0.375024	0.127056	0.025464	0.459771
0	0	1	0	21.02413	0.444194	0.089812	0.030142	0.111366
0	0	1	0	21.314	0.549659	0.069418	0.028815	0.107555
0	0	1	0	20.40377	0.18401	0.088053	0.053428	0.2777
0	0	1	0	20.38442	0.129849	0.066209	0.019038	-0.02864
0	0	1	0	20.80398	0.362656	0.042738	0.018927	0.089359
0	0	2	0	21.15073	0.330976	0.035768	0.005734	0.007813
0	0	2	0	21.20286	0.34527	0.025518	0.003398	-0.0009
0	0	1	1	19.97602	0.234534	0.12872	0.12872	0.1236
0	0	1	1	20.52938	0.250131	0.049708	0.010172	0.153411
0	0	1	1	20.70098	0.346148	0.047928	0.012827	0.232369
0	0	1	1	20.79359	0.426319	0.032073	0.008658	0.175179
0	0	3	0.666667	20.75439	0.339752	0.087748	0.047779	0.3248

0	0	3	0.666667	20.80533	0.338362	0.092187	0.027202	0.064513
0	0	3	0.666667	21.13091	0.441636	0.067841	0.031278	0.100139
0	0	1	0	20.71376	0.204861	0.177031	0.177031	0.1442
0	0	1	0	21.18215	0.132948	0.11861	0.024853	-0.06862
0	0	1	0	20.59826	0.321499	0.082471	0.073144	0.0819
0	0	1	0	20.49996	0.249355	0.018284	0.022614	0.018944
0	0	1	0	20.56123	0.283432	0.028753	0.014667	0.026515
0	0	1	1	20.47842	0.053277	0.076042	0.076042	0.0705
0	0	1	1	20.50073	0.02807	0.089495	0.024958	0.011797
0	0	1	1	20.72262	0.163187	0.061177	0.024342	0.115544
0	0	1	1	20.80282	0.192391	0.075947	0.020744	0.116072
0	0	1	1	20.81396	0.157339	0.124649	0.028878	0.28
0	0	1	1	21.03902	0.208188	0.139792	0.0279	0.449767
0	0	1	1	21.7452	0.207321	0.128102	0.053824	0.639922
0	0	1	1	22.01349	0.284073	0.145711	0.029522	0.498914
0	0	1	1	22.30419	0.339948	0.157654	0.032422	0.399298
0	0	1	0	21.1561	0.149959	0.085912	0.038634	-0.0835
0	0	1	0	21.31903	0.191009	0.114411	0.028193	0.492638
0	0	1	0	21.4417	0.197929	0.088288	0.025468	0.219906
0	0	1	0	21.62544	0.291911	0.0542	0.017614	-0.04489
0	0	2	1	21.78142	0.291879	0.062372	0.022099	0.0405
0	0	2	1	22.09221	0.423098	0.029086	0.016391	-0.05089
0	0	2	1	20.96734	0.401982	0.073705	0.016489	0.478339
0	0	1	0	22.08779	0.35299	0.067597	0.02586	0.164262
0	0	1	0	22.27135	0.416608	0.048615	0.024532	0.277431
0	0	1	0	22.25806	0.399851	0.042409	0.011454	0.030872
1	1	1	1	22.07652	0.374032	0.073787	0.055076	0.2499
1	1	1	1	22.27568	0.436234	0.079666	0.020929	0.301719
1	1	1	1	22.50988	0.497126	0.072243	0.019312	0.265339
1	1	1	1	20.25545	0.268655	0.186455	0.186455	0.1873
1	1	1	1	20.47395	0.119216	0.167325	0.040017	0.053746
1	1	1	1	20.61499	0.110709	0.187167	0.044632	0.287078
0	0	1	1	21.69097	0.226556	0.11931	0.11931	0.2453
0	0	1	1	21.74104	0.20949	0.120709	0.034644	0.104388
0	0	1	1	21.76324	0.192318	0.128742	0.035061	0.104124
0	0	1	0	22.38119	0.208329	0.108349	0.029501	0.28542
0	0	1	0	22.43413	0.173476	0.104736	0.027079	0.080016
0	0	1	0	21.65666	0.484035	0.040103	-0.00862	0.226444
0	0	1	0	21.88024	0.570478	0.055453	-0.00178	0.458855
0	0	1	1	22.40184	0.075022	0.148037	0.054406	-0.1167
0	0	2	1	22.12743	0.227855	0.079511	0.014118	0.2067
0	0	2	1	22.20781	0.248913	0.068107	0.016824	0.055238
0	0	2	0	20.7459	0.16963	0.089322	0.089322	-0.0368
0	0	2	0	20.79502	0.174724	0.064325	-0.0036	0.019131
0	0	2	0	21.48459	0.180606	0.07718	0.07718	-0.1765
0	0	2	0	21.51546	0.283024	0.079179	0.052822	-0.0179
0	0	2	0	21.92781	0.2457	0.066217	0.019807	0.123078
1	1	2	0	21.82148	0.144061	0.040034	0.014246	0.144454
1	1	2	0	21.86829	0.146943	0.063558	0.016862	0.038873
1	1	2	0	21.91867	0.159402	0.054217	0.02473	0.084303
1	1	2	0	22.26449	0.387969	0.062865	0.019148	0.083014
1	1	1	1	22.16028	0.406642	0.126974	0.065404	0.2511
1	1	1	1	22.18932	0.426286	0.0344	0.028185	-0.067
0	0	3	0.666667	20.58126	0.094729	0.107602	0.081326	0.1423
0	0	3	0.666667	20.6379	0.082308	0.104231	0.015251	0.134303
0	0	3	0.666667	20.68251	0.066912	0.106836	0.027238	-0.01298
1	1	1	0	21.76174	0.578521	0.068555	0.039213	0.1135
1	1	1	0	22.24929	0.642515	0.038187	0.025148	0.647401

1	1	1	0	23.58411	0.544789	0.060593	0.008314	2.439293
1	1	1	0	21.65703	0.10056	0.121017	0.121017	0.146
1	1	1	0	21.70309	0.072781	0.077073	0.020631	-0.17013
1	1	1	0	21.73985	0.071777	0.048599	0.016735	-0.24608
1	1	1	0	21.77633	0.059511	0.071592	0.025348	0.176774
1	1	2	0	22.32729	0.111809	0.160932	0.15405	0.66658
0	0	1	1	20.42412	0.116299	0.093373	0.018303	0.067068
0	0	1	1	20.62424	0.240642	0.085055	0.025555	0.096659
0	0	1	1	20.66843	0.235983	0.109407	0.027065	0.417786
0	0	1	1	20.66883	0.24674	0.105996	0.028587	0.04859
0	0	1	1	20.91342	0.394971	0.120262	0.054843	0.001239
0	0	2	0.5	21.11416	0.690389	0.087968	0.087968	0.0565
0	0	2	0.5	21.3617	0.563239	0.063429	0.020685	-0.03265
0	0	2	0.5	21.42308	0.566677	0.070715	0.023137	0.056459
0	0	2	0.5	21.84389	0.699044	0.05629	0.019452	0.252528
0	0	2	0.5	21.97169	0.704805	0.090187	0.02261	0.469831
0	0	3	0.333333	21.26003	0.184805	0.086126	0.059596	0.2047
0	0	3	0.333333	21.35505	0.202242	0.082195	0.019878	0.078656
0	0	3	0.333333	21.45507	0.229249	0.068094	0.018594	0.070452
0	0	1	0	21.34112	0.336235	0.160731	0.160731	-0.0205
0	0	2	1	22.38583	0.206722	0.098927	0.098927	0.0902
0	0	2	1	22.58791	0.274455	0.109817	0.029441	0.387271
0	0	2	1	22.58096	0.235669	0.062218	0.014914	-0.15618
0	0	1	1	22.6381	0.228755	0.075746	0.013377	0.157183
0	0	1	1	20.90453	0.353852	0.117536	0.028632	0.108329
0	0	1	1	21.62207	0.250928	0.051093	0.029974	-0.07122
0	0	1	1	21.91215	0.155071	0.053944	0.017684	0.714372
0	0	1	1	22.00975	0.203785	0.066011	0.018779	0.386164
0	0	1	1	22.04839	0.189662	0.080311	0.023492	0.128624
0	0	2	1	22.5066	0.330793	0.123333	0.056083	0.3014
0	0	2	1	22.58253	0.32908	0.098585	0.017184	0.153481
0	0	2	1	22.65277	0.320489	0.10012	0.024109	0.311674
0	0	2	1	22.82519	0.390941	0.072886	0.028639	-0.11338
1	1	1	1	22.92256	0.417856	0.069064	0.026781	0.183733
0	0	1	1	20.21584	0.175724	0.101484	0.029714	0.0929
0	0	1	1	20.26956	0.150424	0.104627	0.032765	0.092873
0	0	1	1	20.65235	0.320232	0.079382	0.027211	0.040763
0	0	2	0.5	21.50586	0.36758	0.13294	0.040451	0.492555
0	0	2	0.5	21.49652	0.308596	0.13127	0.043767	0.196892
0	0	2	0.5	21.62399	0.337188	0.131848	0.030189	0.007956
0	0	3	0.666667	19.86721	0.106803	0.083942	0.042732	0.0377
0	0	3	0.666667	19.89907	0.100388	0.090626	0.01811	0.213479
0	0	3	0.666667	19.95297	0.077982	0.082506	0.014127	0.02864
0	0	3	0	21.36252	0.136989	0.151149	0.151149	0.2028
0	0	3	0	21.43608	0.077227	0.188998	0.042146	0.078523
0	0	3	0	21.62362	0.090927	0.201601	0.053701	0.129948
0	0	3	0	21.77207	0.111068	0.161789	0.052091	0.101286
0	0	1	1	21.75848	0.248042	0.087132	0.022218	0.1621
0	0	2	0.5	21.77642	0.252112	0.034725	0.008896	-0.06684
0	0	2	0.5	21.75547	0.22051	0.092265	0.02225	0.159114
0	0	1	0	21.99548	0.134732	0.20732	0.121185	0.0794
0	0	1	0	22.09052	0.156412	0.200814	0.067132	0.132334
0	0	2	1	21.79492	0.150496	0.090781	0.057188	0.7308
0	0	2	1	21.87821	0.140475	0.074618	0.013198	0.042811
0	0	1	1	20.93621	0.41057	0.174021	0.112955	1.19
0	0	1	1	21.09055	0.390495	0.108292	0.079006	0.200999
0	0	1	1	21.3572	0.437207	-0.06702	-0.01909	-0.24256
0	0	2	1	21.83536	0.266965	0.045076	0.034527	0.0916

0	0	2	1	21.94148	0.288775	0.051643	0.020332	0.133302
0	0	2	1	22.42661	0.248336	0.069977	0.024743	0.561839
0	0	2	1	22.49819	0.213768	0.085398	0.028248	0.213463
0	0	2	1	22.61014	0.229333	0.084923	0.026853	0.092519
0	0	2	0.5	21.7741	0.406194	0.131277	0.035647	0.324291
0	0	2	0.5	21.81498	0.305202	0.149947	0.04187	0.322826
0	0	2	1	21.22073	0.530899	0.066735	0.04097	0.1948
0	0	2	1	21.27959	0.53693	0.046948	0.008379	0.143307
0	0	2	1	21.44485	0.585123	0.035922	0.006521	-0.04392
0	0	2	1	21.80714	0.465153	0.029989	0.006902	0.183959
0	0	2	1	21.95082	0.491882	0.06787	0.010483	0.466668
0	0	2	1	22.09524	0.4566	0.137637	0.021766	0.220291
1	1	1	0	21.96983	0.181319	0.124381	0.124381	0.0578
0	0	1	1	20.99757	0.337196	0.090672	0.020121	-0.0471
0	0	1	1	21.63104	0.467704	0.063301	0.031468	0.139821
0	0	1	1	21.80987	0.452806	0.075197	0.018125	0.618318
0	0	1	1	21.98631	0.500779	0.065942	0.020721	0.2043
0	0	1	1	22.3437	0.678953	0.057493	0.020526	0.209437
0	0	1	1	20.42134	0.36463	0.100093	0.078189	0.1725
0	0	1	1	20.5538	0.378414	0.09266	0.039521	0.102501
0	0	1	1	20.63175	0.35166	0.092894	0.032362	0.055592
1	1	3	0.666667	21.91921	0.535256	0.058586	0.012964	0.1672
1	3	3	0.666667	22.10254	0.430347	0.047759	0.014572	0.101192
1	3	3	0.666667	22.29738	0.569577	0.040706	0.010727	0.213323
1	3	3	0.666667	22.46148	0.596405	0.037453	0.01348	0.051995
1	3	3	0.666667	22.61471	0.592772	0.031468	0.007567	0.12151
1	1	2	0	21.95892	0.489051	0.085446	0.010541	0.661799
1	1	2	0	22.32489	0.606706	0.036956	0.020018	0.11241
1	1	2	0	22.24888	0.630087	-0.02947	0.009857	-0.1167
0	0	2	0	21.35521	0.096827	0.078154	0.020878	-0.0647
0	0	2	0	21.41385	0.099463	0.070024	0.026605	-0.01731
0	0	2	0	21.38013	0.099647	0.019056	0.0245	0.000737
0	0	2	1	20.70218	0.140984	0.170863	0.038996	0.603079
0	0	2	1	21.2466	0.394086	0.160981	0.055483	0.591465
0	0	2	1	21.70234	0.248168	0.154848	0.055993	0.413257
0	0	2	1	21.55409	0.296285	0.15818	0.038454	0.54982
0	0	2	1	21.89562	0.394173	0.121709	0.056487	0.378781
0	0	2	1	21.9752	0.358608	0.099683	0.026543	0.125632
0	0	2	1	20.02543	0.073265	0.084726	0.084726	0.1991
0	0	2	1	20.0581	0.064922	0.066872	0.030213	-0.07019
0	0	2	1	20.07299	0.055124	0.048087	0.011078	-0.08223
0	0	1	1	20.68125	0.122801	0.115831	0.103622	0.1107
0	0	1	1	20.71943	0.124608	0.11217	0.02008	0.110005
0	0	2	1	21.69575	0.164375	0.082906	0.082906	0.1003
0	0	2	1	21.78348	0.200146	0.060672	0.01489	0.222543
0	0	2	1	22.04347	0.305523	0.074777	0.021752	0.252793
0	0	2	0.5	20.00162	0.123506	0.095831	0.076526	0.0014
0	0	2	0.5	20.16744	0.18483	0.110637	0.023227	0.468814
0	0	2	0.5	20.56076	0.316628	0.098627	0.041041	0.467549
0	0	2	0.5	20.75848	0.450068	0.136569	0.029953	0.271579
0	0	2	1	20.85992	0.183092	0.084188	0.022101	0.0715
0	0	2	1	21.01158	0.243354	0.06725	0.017973	0.180028
0	0	2	1	21.08257	0.271605	0.044456	0.016763	0.08994
0	0	1	1	21.2289	0.502714	0.072979	0.013428	0.346924
0	0	1	1	21.48155	0.55986	0.060932	0.017547	0.325455
0	0	1	1	21.72624	0.486572	0.039672	0.00901	0.612279
0	0	2	1	21.05478	0.36304	0.098255	0.02195	0.15629
0	0	2	1	21.41834	0.480529	0.051259	0.018196	0.0689

0	0	2	1	21.29703	0.394918	0.063263	0.017103	0.206396
0	0	2	0.5	21.22421	0.310918	0.120177	0.02119	0.21706
0	0	2	0.5	21.80101	0.196429	0.083533	0.025807	0.385675
0	0	2	0.5	21.92126	0.229235	0.088852	0.015594	0.256646
0	0	2	0.5	22.04656	0.246674	0.11876	0.021436	0.072142
0	0	2	0.5	22.20864	0.279781	0.112247	0.025342	0.269246
0	0	2	1	20.42046	0.299676	0.091897	0.031439	0.177
0	0	2	1	20.56177	0.313569	0.09441	0.021243	0.096229
0	0	2	1	20.93488	0.442559	0.093667	0.032813	0.352087
1	1	1	1	22.74889	0.496725	0.072559	0.01775	0.101057
1	1	1	1	22.94652	0.547361	0.053945	0.015596	0.162508
0	0	1	1	21.00577	0.389623	0.027941	0.005912	0.210797
0	0	1	1	21.13663	0.453383	0.007373	0.001507	0.05732
0	0	2	1	21.03485	0.269646	0.088613	0.022384	0.129089
0	0	2	1	21.1627	0.29855	0.095189	0.022756	0.273547
0	0	2	0.5	21.48754	0.320184	0.053908	0.04328	-0.0148
0	0	2	0.5	20.36985	0.043555	0.083458	0.083458	-0.0457
0	0	2	0.5	20.42831	0.062455	0.05627	0.013818	-0.02778
0	0	2	0.5	20.4549	0.057837	0.054604	0.014869	-0.01916
0	0	2	0.5	20.36071	0.070712	0.058292	0.016005	0.335811
0	0	2	0.5	20.27258	0.072348	0.0753	0.020571	0.242726
0	0	2	0	21.27927	0.102084	0.11574	0.062566	-0.0844
1	1	2	0	20.42502	0.263502	0.104417	0.036141	0.016
1	1	2	0	20.56039	0.302803	0.109553	0.028779	0.223685
1	1	2	0	20.62898	0.235484	0.140824	0.026713	-0.09837
1	1	2	0	20.69857	0.27727	0.065561	0.018896	0.037571
0	0	2	1	20.55284	0.384374	0.037565	0.013948	0.182195
0	0	2	1	20.79926	0.500013	0.048304	0.017596	1.018767
0	0	2	1	20.95429	0.574769	-0.06881	0.006832	-0.30742
0	0	2	1	21.05862	0.601958	0.030325	0.008769	0.401014
1	1	1	1	21.01827	0.175006	0.082381	0.070495	-0.0578
1	1	1	1	21.0716	0.178027	0.093419	0.017317	0.077011
0	0	1	1	21.71458	0.238424	0.130573	0.0893	0.9122
0	0	1	1	21.94772	0.288394	0.137379	0.047705	0.528077
0	0	1	1	22.34827	0.408459	0.137149	0.048986	0.452464
0	0	2	0	21.92912	0.336717	0.117482	0.013285	0.231294
0	0	2	0	22.08824	0.352817	0.112125	0.01977	0.223588
0	0	1	0	21.3423	0.318627	0.100367	0.087935	0.3816
0	0	1	0	21.66683	0.425562	0.103701	0.029588	0.3559
1	1	1	0	20.93863	0.223338	0.080622	0.056743	0.1097
1	1	1	0	21.11524	0.320662	0.052772	0.023413	0.084533
0	0	2	1	20.31065	0.214115	0.090366	0.090366	0.2897
0	0	2	1	20.33087	0.231707	0.046746	0.018458	0.452377
0	0	2	1	20.34744	0.211651	0.059883	0.013205	0.195396
0	0	2	1	20.82523	0.328171	0.074892	0.016384	0.072
0	0	2	1	20.96985	0.361118	0.118562	0.034693	0.685697
0	0	2	1	21.06681	0.374668	0.103166	0.030233	0.200384
0	0	2	1	21.21466	0.433848	0.080338	0.025098	0.014808
0	0	2	0.5	22.06111	0.188145	0.194273	0.127421	0.1836
0	0	2	0.5	22.36016	0.353593	0.114485	0.04831	0.316112
0	0	2	0.5	22.38562	0.290258	0.13385	0.041087	0.127419
1	1	3	0.333333	21.30323	0.204272	0.051778	0.030094	0.0705
1	1	3	0.333333	21.33148	0.230763	0.029281	0.011245	0.003857
1	1	3	0.333333	21.3676	0.241536	0.042494	0.009615	0.070521
0	0	2	1	21.74179	0.323016	0.069946	0.069946	0.035
0	0	2	1	21.82235	0.364764	0.049691	0.038771	0.123544
0	0	2	1	21.76753	0.347671	0.014589	0.022664	0.070091
0	0	2	1	21.77629	0.38476	0.009729	0.030655	0.028595

1	1	1	0	21.13405	0.481137	0.077608	0.032326	0.0598
1	1	1	0	21.35672	0.262489	0.062914	0.019329	0.062719
1	1	1	0	21.46853	0.331932	0.042876	0.017341	-0.05132
0	0	2	1	20.73697	0.279087	0.12921	0.035659	0.153703
0	0	2	1	20.89493	0.282483	0.153883	0.04308	0.287501
0	0	2	1	21.54481	0.177124	0.119669	0.056453	0.403603
0	0	2	1	21.53597	0.14097	0.110393	0.041546	0.067134
0	0	2	1	21.49904	0.146626	0.09026	0.021244	-0.26762
0	0	2	0	20.83981	0.082227	0.078395	0.049649	0.0157
0	0	2	0	21.16219	0.284978	0.033982	0.014984	-0.10969
0	0	2	0	21.1052	0.226373	0.028945	0.01051	0.224999
0	0	2	0	21.20313	0.320633	-0.02615	-0.00918	-0.06671
0	0	2	0	21.2652	0.351937	0.019769	0.003606	0.184077
0	0	1	0	20.95072	0.130219	0.122092	0.106799	0.0651
0	0	1	0	21.00762	0.10199	0.104694	0.039319	0.016567
1	1	2	0.5	21.7636	0.639255	0.042315	0.020533	-0.0829
1	1	2	0.5	22.05571	0.696504	0.039696	0.01021	0.361681
1	1	2	0.5	22.40834	0.71099	0.060847	0.014656	0.94113
0	0	1	0	22.98214	0.748023	-0.00029	0.01132	-0.07444
0	0	1	0	23.08462	0.587711	0.040553	0.004458	0.213469
0	0	1	0	23.53375	0.634299	0.152778	0.037533	0.974322
0	0	1	0	23.67085	0.558742	0.123767	0.048685	0.496988
0	0	1	0	23.8703	0.5663	0.025202	0.005465	0.304625
1	1	1	0	21.52625	0.299351	0.082483	0.082483	0.3083
1	1	1	0	21.61563	0.319746	0.082098	0.026374	0.104435
1	1	1	0	21.62955	0.307931	0.062727	0.019548	-0.00273
0	0	1	1	21.12289	0.256546	0.111677	0.074979	0.0455
0	0	1	1	21.1543	0.283932	0.02526	0.039244	-0.34241
1	1	2	1	22.37569	0.339238	0.141947	0.192441	0.3011
1	1	2	1	22.6044	0.386344	0.16015	0.044541	0.390217
1	1	1	1	23.07464	0.522792	0.127975	0.056961	0.37607
1	1	1	1	23.22961	0.486088	0.132671	0.040176	0.209491
0	0	1	1	20.47117	0.064421	0.064917	0.064917	-0.0318
0	0	1	1	20.61954	0.175066	0.040959	0.016714	0.293033
0	0	1	1	20.94168	0.373319	0.047458	0.008177	0.327841
0	0	1	1	20.85111	0.315702	0.108696	0.012116	0.156161
0	0	1	1	21.41202	0.347423	0.099601	0.099601	0.1754
0	0	1	1	21.44052	0.315621	0.088764	0.020184	0.112023
0	0	1	1	21.55989	0.302976	0.104394	0.025095	0.199996
0	0	1	1	21.74561	0.35457	0.107644	0.021685	0.092708
0	0	1	1	22.28415	0.743902	0.04021	0.014921	0.2377
0	0	1	1	22.54976	0.783835	0.032877	0.003159	0.308074
0	0	1	1	22.52751	0.721498	0.049355	0.003252	-0.02242
0	0	1	1	20.93725	0.078984	0.114418	0.038908	0.1492
0	0	1	1	21.10214	0.111091	0.137147	0.034657	0.250682
0	0	1	1	21.31119	0.218397	0.097347	0.028263	0.051521
1	1	1	1	21.55872	0.570055	0.029596	0.015444	-0.1174
1	1	1	1	21.79709	0.647133	0.022717	0.011011	0.00569
1	1	1	1	21.99199	0.696046	0.025607	0.012626	0.243151
1	1	1	1	22.10751	0.715735	0.029556	0.014773	0.172109
1	1	1	1	22.10786	0.706155	0.020389	0.014817	-0.04118
0	0	1	1	20.57671	0.290983	0.075352	0.08332	0.0977
0	0	1	1	20.66267	0.302429	0.07497	0.011075	0.152208
0	0	1	1	20.73397	0.321354	0.056334	0.012261	0.096782
0	0	4	1	20.4053	0.142886	0.07769	0.043091	-0.0211
0	0	4	1	20.47765	0.148349	0.067948	0.024698	-0.09577
0	0	4	1	20.48698	0.123153	0.072241	0.021031	0.00196
0	0	4	1	20.54464	0.137044	0.048421	0.029968	0.061119

0	0	4	1	20.62758	0.175813	0.048699	0.018487	-0.09397
0	0	1	0	21.87123	0.184031	0.099371	0.028643	0.177835
0	0	1	0	22.02883	0.244085	0.08665	0.034279	0.160901
0	0	1	0	22.09811	0.265556	0.096567	0.030737	0.478275
0	0	1	1	21.77453	0.464179	0.075648	0.017438	0.190812
0	0	2	0.5	21.26908	0.169808	0.086358	0.086358	0.3406
0	0	2	0.5	21.358	0.177894	0.096881	0.024321	0.086956
0	0	2	0.5	21.45951	0.183857	0.113241	0.025875	0.122807
0	0	1	0	23.65045	0.319152	0.120341	0.120341	0.0571
0	0	1	0	23.678	0.311408	0.104703	0.017767	0.125237
0	0	1	0	23.72025	0.307918	0.116098	0.015422	-0.01437
0	0	1	0	23.77657	0.349167	0.119514	0.020024	0.043228
0	0	1	1	21.74037	0.312826	0.069083	0.01202	0.0793
0	0	5	0.8	21.95675	0.397388	0.048003	0.011237	0.109678
0	0	5	0.8	22.37288	0.509197	0.045481	0.007765	0.701695
0	0	5	0.8	22.64077	0.590057	0.034125	0.006397	0.110704
0	0	2	1	22.36924	0.278226	0.169138	0.037599	0.16769
0	0	1	1	20.6471	0.166938	0.101803	0.007074	0.1083
0	0	1	1	22.36995	0.30455	0.045084	0.006903	1.524529
0	0	1	1	22.54081	0.38499	0.062271	0.004467	1.730125
1	1	1	1	22.65911	0.422517	0.075012	0.001521	0.206951
1	1	1	1	22.68926	0.440317	0.045449	0.007275	0.064757
0	0	1	0	22.8576	0.5931	0.054693	0.016214	0.1186
0	0	2	0	23.32873	0.712568	0.035624	0.011762	0.091822
0	0	2	0	21.56927	0.165535	0.064854	0.064854	-0.1788
0	0	2	0	21.66469	0.209737	0.059042	0.003585	0.101423
0	0	2	0	21.78234	0.249324	0.079388	0.013929	0.36879
0	0	2	0	21.86505	0.241901	0.118695	0.030353	0.161737
1	1	3	0.333333	20.16805	0.094701	0.088783	0.045213	0.0429
1	1	3	0.333333	20.21289	0.105467	0.071161	0.018452	0.087807
1	1	3	0.333333	20.18089	0.077048	0.047426	0.011167	-0.29511
0	0	2	1	21.30157	0.400479	0.095824	0.095824	0.0092
0	0	2	1	21.40145	0.381049	0.133707	-0.01019	0.116607
0	0	2	1	21.51362	0.386543	0.133411	-0.01482	0.101829
0	0	2	1	21.59571	0.376259	0.13143	-0.00077	0.135048
0	0	1	1	20.82106	0.429026	0.029491	0.012953	0.193715
0	0	1	1	20.95249	0.536675	-0.04152	0.010007	-0.07139
0	0	1	1	20.64676	0.061096	0.097554	0.048332	0.1749
0	0	1	1	20.8985	0.173549	0.091846	0.036434	0.38244
0	0	1	1	21.01652	0.125534	0.091675	0.033777	0.069032
0	0	1	1	20.82524	0.250776	0.092641	0.092641	0.1822
0	0	1	1	20.93661	0.263858	0.094285	0.014872	0.129958
0	0	1	1	20.99167	0.257643	0.069588	0.016129	0.002283
1	3	1	1	21.53786	0.273052	0.131673	0.057317	0.0802
1	3	1	1	21.57043	0.216729	0.138133	0.068601	0.043482
1	3	1	1	21.7167	0.259892	0.124051	0.066821	0.097235
1	3	1	1	21.87965	0.200428	0.107481	0.051167	0.117604
0	0	1	0	21.08263	0.180193	0.093603	0.047686	0.1766
0	0	1	0	21.21913	0.312803	0.058519	0.021638	0.133979
0	0	1	0	21.44807	0.442224	0.066901	0.018027	0.512007
1	1	1	1	21.07987	0.378269	0.140326	0.140326	0.8835
1	1	1	1	21.23183	0.400572	0.085856	0.043258	-0.1457
1	1	1	1	21.38316	0.42218	0.094747	0.025642	0.266764
1	1	1	1	21.47802	0.451164	0.05289	0.023964	-0.17124
0	0	2	1	20.23349	0.243709	0.087917	0.075231	-0.011
0	0	2	1	20.20325	0.219311	0.021849	0.016822	0.472041
0	0	2	1	20.14582	0.166936	0.030089	0.014741	-0.26691
0	0	1	1	21.23872	0.351936	0.072416	0.048199	-0.1272

0	0	1	1	21.27941	0.275797	0.093551	0.016546	0.070704
0	0	1	1	21.3167	0.263681	0.11305	0.030741	0.248958
0	0	1	1	21.39947	0.264846	0.115016	0.034859	0.221063
0	0	1	1	21.46008	0.246635	0.122192	0.031821	0.113093
0	0	3	1	21.19901	0.053638	0.099652	0.022364	-0.29568
0	0	3	1	21.28347	0.082909	0.092571	0.00405	0.06271
0	0	3	1	21.48327	0.232934	0.071291	0.022886	0.467914
0	0	3	1	21.71207	0.35353	0.073992	0.006145	0.073042
0	0	3	1	21.8632	0.411374	0.075061	0.012974	0.132181
0	0	2	1	20.78316	0.101521	0.089438	0.070635	0.0823
0	0	2	1	20.84235	0.123787	0.071612	0.02149	0.171226
0	0	2	1	20.93914	0.165172	0.046985	0.020559	-0.12538
0	0	2	1	22.28798	0.136662	0.107572	0.058152	0.137
0	0	2	1	22.3418	0.133125	0.165673	0.037985	0.18688
0	0	2	1	22.41903	0.156347	0.08751	0.035302	-0.09636
0	0	2	1	21.15992	0.206182	0.099261	0.099261	0.0666
1	3	2	1	21.19491	0.162973	0.111947	0.023668	0.109817
1	1	2	1	21.49996	0.25115	0.088435	0.027232	0.154134
1	1	2	1	22.05026	0.284465	0.075614	0.026289	0.25299
0	0	2	1	20.68218	0.299901	0.054474	0.011538	0.393356
0	0	2	0	21.43864	0.136344	0.12864	0.058816	0.1147
0	0	2	0	21.54941	0.204283	0.13387	0.030586	0.18735
0	0	2	0	21.56961	0.197663	0.149651	0.039158	0.153739
0	0	2	0	21.92136	0.367149	0.11026	0.020969	0.208128
0	0	2	0	22.06527	0.393265	0.109765	0.023949	0.041013
1	1	2	1	22.36998	0.390393	0.097831	0.097831	0.0113
1	1	2	1	23.5892	0.719956	0.043842	0.028568	1.4719
0	0	1	1	22.06552	0.419199	0.078867	0.083947	0.1836
0	0	1	1	21.43089	0.121802	0.049122	0.029237	0.0506
0	0	1	1	20.98816	0.060148	0.054701	0.015257	-0.0804
1	1	1	1	21.32412	0.139082	0.036087	0.036087	-0.148
0	0	1	0	21.66217	0.083699	0.116621	0.098619	0.0429
0	0	1	1	21.07484	0.076135	0.082372	0.082372	0.2347
0	0	3	1	20.844	0.113205	0.064436	0.064436	0.383
1	1	1	0	21.0744	0.158445	0.060617	0.060617	0.0323
0	0	3	0.333333	20.74672	0.124621	0.084988	0.084988	0.1337
1	1	1	0	20.86912	0.246869	0.092749	0.092749	0.0734
0	0	2	0	20.9783	0.092759	0.088593	0.088593	0.1309
0	0	3	0.666667	21.69924	0.041515	0.15089	0.130704	0.2538
0	0	2	0	20.26129	0.299173	0.114286	0.114286	-0.0954
0	0	2	0.5	20.80037	0.57678	0.204908	0.204908	0.255
0	0	2	1	20.6359	0.472456	0.116876	0.116876	0.0757
1	1	2	0	21.12233	0.59321	0.151761	0.151761	0.1949
0	0	2	1	20.31282	0.238967	0.142782	0.142782	0.2636
0	0	1	1	20.70697	0.533579	0.112355	0.112355	0.0434
0	0	2	1	20.62509	0.495838	0.140671	0.140671	-0.1132
0	0	2	0.5	20.71919	0.372556	0.178375	0.178375	0.1075
0	0	2	1	20.47605	0.323879	0.055214	0.030082	-0.0939
0	0	1	0	22.78814	0.71058	0.054871	0.054871	0.2142
0	0	1	0	21.30273	0.281467	0.154373	0.154373	-0.0153
0	0	3	1	20.95103	0.377377	0.151232	0.151232	0.0147
0	0	1	1	19.80039	0.123964	0.188367	0.188367	0.1929
1	1	1	1	21.61807	0.360099	0.097939	0.097939	0.1978
0	0	1	1	21.62254	0.586162	0.086158	0.086158	0.0536
0	0	1	1	20.0822	0.377939	0.162197	0.162197	0.3022

Board	Independe	Salary	Meet	Sex	Education	Establish	Industry	Year
2.197225	0.333333	13.89247	2.197225	0	1	3.178054	H61	2012
1.94591	0.428571	13.25689	1.94591	1	1	3.178054	H61	2013
1.94591	0.428571	13.25689	1.94591	1	1	3.218876	H61	2014
1.94591	0.428571	15.18033	2.197225	1	1	3.258097	C37	2015
1.791759	0.5	13.20486	2.079442	1	4	2.833213	C39	2009
1.94591	0.428571	12.06853	2.302585	1	4	2.890372	C39	2010
1.94591	0.428571	12.36308	2.079442	1	4	2.944439	C39	2011
1.94591	0.428571	13.41682	1.791759	1	4	2.995732	C39	2012
1.94591	0.428571	13.75236	1.386294	1	4	3.044522	C39	2013
1.94591	0.428571	13.80202	1.609438	1	4	3.091042	C39	2014
1.94591	0.428571	13.61535	2.302585	1	4	3.135494	C39	2015
1.791759	0.5	13.90132	1.791759	1	4	3.178054	C39	2016
1.791759	0.5	13.94654	2.302585	1	4	3.218876	C39	2017
1.791759	0.5	13.94654	1.791759	1	4	3.258097	C39	2018
1.791759	0.5	14.00199	1.098612	1	4	3.295837	C39	2019
2.639057	0.357143	15.16812	3.044522	1	4	2.995732	K70	2009
2.639057	0.357143	15.04237	2.70805	1	4	3.044522	K70	2010
2.639057	0.357143	15.01653	2.70805	1	4	3.091042	K70	2011
2.639057	0.357143	15.23259	2.833213	1	4	3.135494	K70	2012
2.70805	0.333333	15.9702	2.890372	1	4	3.178054	K70	2013
2.70805	0.333333	15.63403	3.295837	1	4	3.218876	K70	2014
2.833213	0.352941	16.52263	3.401197	1	4	3.258097	K70	2015
2.890372	0.333333	17.40641	2.944439	1	4	3.295837	K70	2016
2.890372	0.333333	16.82683	2.833213	1	4	3.332205	K70	2017
2.890372	0.333333	16.60505	2.70805	1	4	3.367296	K70	2018
2.564949	0.461538	16.27017	2.772589	1	4	3.401197	K70	2019
2.397895	0.363636	14.13759	2.70805	0	3	2.890372	C13	2012
2.397895	0.363636	14.58701	2.197225	0	3	2.944439	C13	2013
2.397895	0.363636	15.47395	2.197225	0	3	2.995732	C13	2014
2.397895	0.363636	15.29343	2.564949	0	3	3.044522	C13	2015
2.397895	0.363636	15.06656	2.564949	0	3	3.091042	C13	2016
2.397895	0.363636	15.90569	2.397895	0	3	3.135494	C13	2017
2.639057	0.357143	16.57552	2.197225	1	3	2.564949	C38	2013
2.564949	0.384615	16.65751	2.484907	1	3	2.639057	C38	2014
2.484907	0.416667	16.57425	2.397895	1	3	2.70805	C38	2015
2.302585	0.4	16.54467	2.70805	1	3	2.772589	C38	2016
2.302585	0.4	16.5679	2.302585	1	3	2.833213	C38	2017
2.197225	0.333333	16.66274	2.564949	1	3	2.890372	C38	2018
2.079442	0.5	16.9061	2.484907	1	3	2.944439	C38	2019
2.197225	0.444444	14.38128	2.197225	1	2	3.135494	D45	2017
2.197225	0.333333	12.65683	2.197225	1	2	2.833213	F51	2013
2.197225	0.333333	14.33627	2.079442	1	4	2.772589	C14	2009
2.197225	0.333333	14.24995	1.791759	1	4	2.833213	F52	2010
2.197225	0.333333	14.22921	2.197225	1	4	2.890372	F52	2011
2.197225	0.333333	14.43609	2.197225	1	4	2.944439	F52	2012
2.197225	0.333333	14.6751	1.098612	1	4	2.995732	F52	2013
2.197225	0.333333	14.79331	2.484907	1	4	3.044522	F52	2014
1.94591	0.428571	14.58851	2.302585	1	3	2.772589	C38	2009
2.079442	0.375	15.08841	1.791759	1	3	2.833213	C38	2010
2.197225	0.333333	15.24527	1.386294	1	3	2.890372	C38	2011
2.197225	0.333333	15.42574	1.94591	1	3	2.944439	C38	2012
2.197225	0.333333	16.16457	1.609438	1	3	2.995732	C38	2013
2.197225	0.333333	15.80414	2.302585	1	3	3.044522	C38	2014
2.197225	0.333333	15.46992	1.791759	1	3	3.091042	C38	2015
2.079442	0.375	14.79785	1.609438	1	3	3.135494	C38	2016
2.197225	0.333333	15.81813	1.609438	1	3	3.178054	C38	2017
2.197225	0.333333	15.98652	1.94591	1	3	3.218876	C38	2018

2.197225	0.333333	11.23585	2.397895	1	1	2.833213	B09	2011
2.197225	0.333333	13.48701	1.791759	1	1	2.890372	B09	2012
2.197225	0.333333	13.44835	2.197225	1	1	2.944439	B09	2013
2.197225	0.333333	13.57979	2.484907	1	1	2.995732	B09	2014
2.197225	0.333333	13.45626	1.609438	1	1	3.044522	B09	2015
2.197225	0.333333	13.29984	2.833213	1	1	3.091042	B09	2016
2.197225	0.333333	13.57408	2.197225	1	1	3.135494	B09	2017
2.197225	0.333333	13.54383	2.564949	1	1	3.178054	B09	2018
2.197225	0.333333	13.38519	2.197225	1	1	3.218876	B09	2019
2.079442	0.375	13.59174	2.197225	1	3	2.890372	K70	2009
2.197225	0.333333	13.79663	1.791759	1	3	3.091042	K70	2013
2.197225	0.333333	13.87378	1.791759	1	3	3.135494	K70	2014
2.197225	0.333333	14.07046	2.484907	1	3	3.178054	K70	2015
2.197225	0.333333	13.95857	2.564949	1	3	3.295837	K70	2018
2.079442	0.375	14.17179	1.791759	1	3	3.332205	K70	2019
1.94591	0.428571	13.64116	1.098612	0	1	2.890372	S90	2009
1.94591	0.428571	13.85473	1.94591	0	1	2.944439	S90	2010
1.609438	0.4	13.64116	2.302585	1	3	3.044522	K70	2009
1.609438	0.4	13.65956	1.94591	1	3	3.091042	K70	2010
1.609438	0.4	13.71015	2.079442	1	3	3.135494	K70	2011
1.609438	0.4	13.71015	2.397895	1	3	3.178054	K70	2012
1.609438	0.4	13.71015	2.772589	1	3	3.218876	K70	2013
2.197225	0.333333	14.58513	2.564949	1	3	2.833213	C27	2009
2.197225	0.333333	14.48973	2.079442	1	3	2.890372	C27	2010
2.197225	0.333333	14.48026	2.197225	1	3	2.944439	C27	2011
2.197225	0.333333	15.0117	1.94591	1	3	2.995732	C27	2012
2.397895	0.454545	14.77375	2.639057	1	3	3.044522	C27	2013
2.397895	0.454545	14.82617	2.564949	1	3	3.091042	C27	2014
2.197225	0.333333	13.79531	2.397895	1	5	3.135494	F52	2009
2.197225	0.333333	14.15198	2.079442	1	5	3.178054	F52	2010
2.197225	0.333333	13.97678	2.079442	1	5	3.218876	F52	2011
2.397895	0.363636	14.22098	2.197225	1	5	3.258097	F52	2012
2.397895	0.363636	14.22098	1.386294	1	5	3.295837	F52	2013
2.397895	0.363636	14.22098	2.197225	1	5	3.332205	F52	2014
2.397895	0.363636	14.18707	1.609438	1	5	3.367296	F52	2015
2.397895	0.363636	14.22098	2.079442	1	5	3.401197	F52	2016
2.397895	0.363636	14.22098	1.791759	1	5	3.433987	F52	2017
2.197225	0.333333	14.22098	1.609438	1	5	3.496508	Q83	2019
1.94591	0.428571	12.65076	2.397895	1	4	2.833213	S90	2009
1.94591	0.428571	12.63786	2.484907	1	4	2.890372	S90	2010
2.197225	0.333333	15.50929	2.302585	1	3	2.833213	C38	2009
2.197225	0.333333	16.08143	2.397895	1	3	2.890372	C38	2010
2.197225	0.333333	16.00493	1.94591	1	3	2.944439	C38	2011
2.197225	0.333333	15.96727	1.94591	1	3	2.995732	C38	2012
2.397895	0.363636	14.5059	2.484907	1	3	3.044522	C38	2013
2.397895	0.363636	14.46679	2.079442	1	3	3.091042	C38	2014
2.397895	0.363636	14.9036	2.302585	1	3	3.135494	C38	2015
2.484907	0.333333	14.65432	2.564949	1	4	2.772589	S90	2009
2.484907	0.333333	14.4669	2.079442	1	4	2.833213	K70	2010
2.397895	0.363636	14.43124	1.94591	1	4	2.890372	K70	2011
2.397895	0.363636	14.77379	1.94591	1	4	2.944439	K70	2012
2.484907	0.333333	15.10749	2.302585	1	4	2.995732	K70	2013
2.484907	0.333333	15.11144	2.079442	1	4	3.044522	K70	2014
2.484907	0.333333	15.35156	3.091042	1	4	3.091042	K70	2015
2.484907	0.333333	15.10518	2.70805	1	4	3.135494	K70	2016
2.484907	0.333333	15.07783	2.890372	1	4	3.178054	K70	2017
2.484907	0.333333	15.11734	2.564949	1	4	3.218876	K70	2018
2.397895	0.363636	15.42355	2.302585	1	4	3.258097	K70	2019

1.609438	0.4	11.83501	2.079442	1	2	2.833213	C27	2009
1.94591	0.428571	12.79968	2.397895	1	2	2.944439	K70	2012
1.94591	0.428571	13.55155	1.94591	1	2	2.995732	K70	2013
1.94591	0.428571	13.32187	2.397895	1	2	3.044522	C30	2014
1.94591	0.428571	14.27407	2.890372	1	2	3.091042	C30	2015
1.791759	0.5	14.26881	2.079442	1	2	3.135494	C30	2016
1.94591	0.428571	15.09419	2.772589	1	2	3.178054	C30	2017
1.94591	0.428571	14.61099	2.484907	1	2	3.218876	N77	2018
1.791759	0.5	15.06203	2.484907	1	2	3.258097	N77	2019
2.079442	0.375	13.30751	2.484907	1	3	3.044522	K70	2009
2.197225	0.333333	13.63075	2.70805	1	3	3.091042	K70	2010
2.197225	0.333333	13.86525	2.772589	1	3	3.135494	K70	2011
2.197225	0.333333	13.8545	2.995732	1	3	3.178054	K70	2012
2.079442	0.375	13.73787	2.772589	1	3	3.218876	K70	2013
2.079442	0.375	13.88158	2.944439	1	3	3.258097	K70	2014
2.197225	0.333333	13.80949	3.135494	1	3	3.332205	K70	2016
2.197225	0.333333	14.06705	2.484907	1	3	3.401197	K70	2018
2.197225	0.333333	13.79183	1.791759	1	4	2.772589	C36	2009
2.197225	0.333333	13.8643	2.302585	1	5	2.833213	C36	2010
2.197225	0.333333	13.84507	2.197225	1	5	2.890372	C36	2011
2.197225	0.333333	13.87971	2.397895	1	5	2.944439	C36	2012
2.197225	0.333333	14.13759	2.397895	1	5	2.995732	C36	2013
2.197225	0.333333	14.04662	1.609438	1	5	3.044522	C36	2014
2.197225	0.333333	14.07093	2.197225	1	5	3.091042	C36	2015
2.197225	0.333333	14.14481	1.791759	1	5	3.135494	C36	2016
2.079442	0.375	14.22861	2.397895	1	5	3.178054	C36	2017
2.197225	0.333333	14.25744	2.397895	1	5	3.218876	C36	2018
2.197225	0.333333	14.96659	1.791759	1	5	3.258097	C36	2019
2.197225	0.333333	15.21768	2.302585	1	2	3.044522	B06	2013
1.94591	0.428571	15.03105	2.302585	1	2	3.091042	B06	2014
1.94591	0.428571	14.85991	2.639057	1	2	3.135494	B06	2015
1.609438	0.4	13.16542	1.94591	1	1	2.833213	K70	2009
1.609438	0.4	13.85281	2.197225	1	1	2.890372	K70	2010
1.609438	0.4	13.95266	1.791759	1	1	2.944439	K70	2011
1.609438	0.4	13.95266	2.302585	1	1	2.995732	K70	2012
1.609438	0.4	13.98862	2.197225	1	1	3.044522	K70	2013
1.609438	0.4	14.00654	2.639057	1	1	3.091042	K70	2014
1.609438	0.4	14.02796	2.302585	1	1	3.135494	K70	2015
1.609438	0.4	14.13251	2.079442	1	1	3.178054	K70	2016
1.609438	0.4	14.13076	2.302585	1	1	3.218876	K70	2017
1.94591	0.428571	13.89478	2.302585	1	5	2.944439	C22	2013
1.94591	0.428571	14.01436	2.079442	1	5	2.995732	C22	2014
1.94591	0.428571	14.14481	2.197225	1	5	3.044522	C22	2015
1.94591	0.428571	14.14481	2.079442	1	5	3.091042	C22	2016
1.94591	0.428571	14.14481	2.639057	1	5	3.178054	C22	2018
1.94591	0.428571	14.46261	2.397895	1	5	3.218876	C22	2019
2.197225	0.333333	13.05622	2.302585	1	1	2.944439	S90	2009
2.197225	0.333333	13.41801	1.791759	1	1	2.995732	C28	2010
2.197225	0.333333	13.65534	2.197225	1	1	3.044522	C28	2011
2.197225	0.333333	13.65534	1.94591	1	1	3.091042	C28	2012
2.197225	0.333333	13.65534	1.609438	1	1	3.135494	C28	2013
2.197225	0.333333	13.64294	1.609438	1	1	3.178054	C28	2014
2.197225	0.333333	13.40453	2.70805	1	1	3.218876	C28	2015
2.197225	0.333333	13.79775	2.70805	1	4	3.091042	C41	2012
2.397895	0.363636	13.31875	2.302585	1	3	2.833213	C34	2013
2.397895	0.363636	13.13627	2.639057	1	3	2.890372	C34	2014
2.397895	0.363636	13.54028	2.564949	1	3	2.995732	C34	2016
2.197225	0.333333	13.50654	2.397895	1	3	3.091042	C34	2018

2.197225	0.333333	13.33747	2.639057	1	2	3.091042	B09	2016
2.197225	0.333333	14.19805	2.397895	1	2	3.135494	B09	2017
2.197225	0.333333	14.352	2.639057	1	2	3.178054	B09	2018
2.197225	0.333333	14.71973	2.079442	1	2	3.218876	B09	2019
1.791759	0.5	13.04115	2.302585	1	4	2.772589	C40	2009
1.94591	0.428571	13.24688	2.197225	1	4	2.833213	C40	2010
1.94591	0.428571	13.42804	2.079442	1	4	2.944439	C40	2012
1.94591	0.428571	13.64393	1.386294	1	4	2.995732	C40	2013
1.94591	0.428571	12.9808	2.397895	1	1	2.772589	C26	2009
1.94591	0.428571	13.11514	2.484907	1	1	2.833213	C26	2010
2.197225	0.333333	13.35205	2.079442	1	1	2.890372	C26	2011
2.197225	0.333333	13.63231	2.302585	1	1	2.944439	C26	2012
2.197225	0.333333	13.719	2.564949	1	1	2.995732	C26	2013
2.197225	0.333333	13.71337	2.302585	1	1	3.044522	C26	2014
2.197225	0.333333	13.77677	2.397895	1	1	3.091042	C26	2015
2.197225	0.333333	13.97559	2.833213	1	5	2.772589	K70	2009
2.197225	0.333333	13.71635	2.302585	1	5	2.833213	K70	2010
2.197225	0.333333	14.40668	1.94591	1	5	2.890372	K70	2011
2.197225	0.333333	14.51895	1.791759	1	5	2.944439	K70	2012
2.197225	0.333333	14.88665	1.791759	1	5	2.995732	K70	2013
2.197225	0.333333	13.79531	1.609438	1	5	3.044522	K70	2014
2.197225	0.333333	13.70982	2.302585	1	5	3.091042	K70	2015
2.197225	0.333333	13.70357	1.94591	1	5	3.135494	K70	2016
2.197225	0.333333	13.72274	1.386294	1	5	3.178054	K70	2017
2.197225	0.333333	12.83094	1.609438	1	1	3.091042	C33	2009
2.079442	0.375	13.50817	2.484907	1	1	3.135494	C33	2010
2.197225	0.333333	13.95952	2.197225	1	1	3.178054	C13	2011
2.197225	0.333333	13.91082	2.079442	1	1	3.218876	C13	2012
2.197225	0.333333	13.84507	1.386294	1	1	3.258097	C13	2013
2.197225	0.333333	13.28244	1.94591	1	1	3.295837	C13	2014
2.197225	0.333333	13.71015	2.639057	1	1	3.332205	C13	2015
2.197225	0.333333	13.45884	2.944439	1	1	3.367296	C13	2016
2.197225	0.333333	13.81551	2.564949	1	1	3.401197	C13	2017
2.197225	0.333333	13.81551	2.484907	1	1	3.433987	C13	2018
2.197225	0.333333	13.20781	2.833213	1	4	2.564949	C27	2009
2.197225	0.333333	13.62785	2.302585	1	4	2.639057	C27	2010
2.079442	0.375	14.03825	2.639057	1	4	2.772589	C27	2012
2.079442	0.375	13.95926	2.833213	1	4	2.833213	C27	2013
2.197225	0.333333	13.85569	2.197225	1	4	2.890372	C27	2014
2.197225	0.333333	14.08485	2.302585	1	4	2.944439	C27	2015
2.197225	0.333333	14.27091	2.397895	1	4	2.995732	C27	2016
2.197225	0.333333	14.33001	1.791759	1	4	3.044522	C27	2017
2.197225	0.333333	14.74448	1.609438	1	4	3.091042	C27	2018
2.197225	0.333333	14.73484	2.079442	1	4	3.135494	C27	2019
2.197225	0.333333	15.67367	2.079442	1	3	3.178054	K70	2011
2.197225	0.333333	16.21124	2.639057	1	3	3.218876	K70	2012
2.197225	0.333333	16.53875	2.890372	1	3	3.258097	K70	2013
2.079442	0.375	16.36913	2.944439	1	3	3.295837	K70	2014
2.079442	0.375	16.64126	2.564949	1	3	3.332205	K70	2015
2.197225	0.333333	17.01048	2.70805	1	3	3.367296	K70	2016
2.197225	0.333333	16.68315	2.70805	1	3	3.401197	K70	2017
2.197225	0.333333	17.41975	2.772589	1	3	3.433987	K70	2018
2.197225	0.333333	17.89956	2.70805	1	3	3.465736	K70	2019
2.197225	0.555556	14.51245	2.944439	1	4	2.995732	K70	2009
2.197225	0.555556	14.89914	2.639057	1	4	3.044522	K70	2010
2.197225	0.555556	14.83403	2.995732	1	4	3.091042	K70	2011
2.197225	0.555556	14.93693	3.367296	1	4	3.135494	K70	2012
2.197225	0.555556	14.94907	2.639057	1	4	3.178054	K70	2013

1.94591	0.428571	14.92529	2.70805	1	4	3.218876	K70	2014
1.94591	0.428571	14.79968	2.302585	1	4	3.258097	K70	2015
1.94591	0.428571	15.60515	3.044522	1	4	3.295837	K70	2016
1.94591	0.428571	16.30177	2.772589	1	4	3.332205	K70	2017
1.94591	0.428571	16.00753	2.639057	1	4	3.367296	K70	2018
1.94591	0.428571	15.97667	2.197225	1	4	3.401197	K70	2019
2.484907	0.333333	13.01967	1.098612	1	3	3.091042	K70	2010
2.484907	0.333333	13.90836	1.386294	1	3	3.135494	K70	2011
2.397895	0.363636	14.00676	1.791759	1	3	3.178054	K70	2012
2.484907	0.416667	14.21099	1.791759	1	3	3.218876	K70	2013
1.94591	0.428571	14.66438	2.197225	1	3	3.295837	K70	2015
1.94591	0.428571	14.74318	1.609438	1	3	3.401197	K70	2018
1.94591	0.428571	14.86563	1.791759	1	3	3.433987	K70	2019
2.197225	0.333333	13.69898	2.197225	1	4	3.367296	C28	2017
2.197225	0.333333	13.69898	1.609438	1	4	3.401197	C28	2018
1.94591	0.428571	15.25274	2.772589	1	3	3.135494	F52	2016
1.94591	0.428571	14.80435	2.397895	1	3	3.178054	F52	2017
1.94591	0.428571	15.09658	2.079442	1	3	3.218876	F52	2018
1.94591	0.428571	14.82245	1.94591	1	3	3.258097	F52	2019
2.197225	0.333333	14.48355	2.079442	1	3	3.044522	C18	2014
2.079442	0.375	14.30059	2.70805	1	3	3.091042	R87	2015
2.079442	0.375	14.29115	2.564949	1	3	3.135494	R87	2016
2.079442	0.375	14.31264	2.302585	1	3	3.178054	R87	2017
2.079442	0.375	14.57431	2.197225	1	3	3.218876	R87	2018
2.079442	0.375	14.6167	1.609438	1	3	3.258097	R87	2019
2.197225	0.333333	14.256	2.079442	1	3	3.258097	B09	2015
2.197225	0.333333	14.5615	2.484907	1	3	3.295837	B09	2016
2.197225	0.333333	14.63466	2.079442	1	3	3.332205	B09	2017
1.791759	0.333333	12.61154	2.197225	1	2	3.044522	C36	2009
1.609438	0.4	12.89922	2.302585	1	2	3.091042	C36	2010
1.609438	0.4	12.67608	2.079442	1	2	3.135494	C36	2011
1.609438	0.4	13.58232	2.484907	1	2	3.178054	C36	2012
1.609438	0.4	13.58861	2.302585	1	2	3.218876	C36	2013
1.609438	0.4	13.91082	2.079442	1	2	3.258097	C36	2014
1.609438	0.4	14.18707	2.564949	1	2	3.295837	C36	2015
1.609438	0.4	14.80876	2.484907	1	2	3.332205	C36	2016
1.609438	0.4	15.17649	2.639057	1	2	3.367296	C36	2017
1.609438	0.4	14.82777	2.564949	1	2	3.401197	C36	2018
1.609438	0.4	16.51699	2.079442	1	2	3.433987	C36	2019
2.197225	0.333333	14.56839	2.397895	1	4	3.044522	C28	2011
2.197225	0.333333	14.95267	2.564949	1	4	3.091042	C28	2012
2.197225	0.333333	14.60805	2.079442	1	4	3.135494	C28	2013
2.197225	0.333333	14.64533	2.197225	1	4	3.178054	C28	2014
2.197225	0.333333	14.65163	2.772589	1	4	3.218876	C28	2015
2.197225	0.333333	14.76175	2.397895	1	4	3.258097	C28	2016
2.197225	0.333333	14.8758	2.484907	1	4	3.295837	C28	2017
2.197225	0.333333	14.92832	2.70805	1	4	3.332205	C28	2018
1.94591	0.428571	15.29939	2.639057	1	4	3.367296	C28	2019
1.94591	0.428571	12.93796	1.609438	1	4	2.772589	K70	2009
1.94591	0.428571	12.73346	1.791759	1	4	2.833213	K70	2010
1.94591	0.428571	13.4813	1.609438	1	4	2.890372	K70	2011
1.94591	0.428571	13.74798	1.791759	1	4	2.944439	K70	2012
1.94591	0.428571	13.61058	2.197225	1	4	2.995732	K70	2013
2.197225	0.333333	14.36109	1.94591	1	4	2.772589	C22	2009
1.94591	0.428571	15.05868	2.484907	1	4	2.833213	K70	2010
2.397895	0.454545	14.98222	1.791759	1	4	2.890372	K70	2011
2.302585	0.5	14.83436	1.791759	1	4	2.944439	K70	2012
2.302585	0.5	15.27663	1.609438	1	4	2.995732	K70	2013

1.94591	0.428571	15.33079	2.302585	1	3	3.044522	K70	2014
1.94591	0.428571	15.0985	1.94591	1	3	3.091042	K70	2015
1.94591	0.428571	15.44183	2.70805	1	3	3.135494	K70	2016
1.94591	0.428571	15.55241	2.079442	1	3	3.178054	K70	2017
1.94591	0.428571	15.48348	2.302585	1	3	3.218876	K70	2018
1.94591	0.428571	15.30832	2.564949	1	3	3.258097	K70	2019
1.94591	0.428571	13.83138	1.098612	1	2	2.833213	C25	2009
1.94591	0.428571	13.83138	1.386294	1	2	2.890372	C25	2010
2.197225	0.333333	13.94654	1.386294	1	2	2.944439	C25	2011
2.197225	0.333333	13.94654	1.94591	1	2	2.995732	C25	2012
2.197225	0.333333	13.94654	1.94591	1	2	3.044522	C25	2013
2.302585	0.4	14.25377	2.079442	1	2	3.091042	C25	2014
2.197225	0.333333	14.05634	2.564949	1	2	3.295837	C25	2019
2.197225	0.333333	14.04662	3.091042	1	3	3.178054	C27	2017
2.197225	0.333333	13.87255	3.178054	1	3	3.218876	C27	2018
1.94591	0.571429	13.85847	2.197225	1	4	2.772589	C35	2009
1.94591	0.571429	13.91109	1.386294	1	4	2.833213	C35	2010
1.94591	0.571429	13.25637	1.386294	1	4	2.890372	C35	2011
2.197225	0.444444	13.3322	1.609438	1	4	2.944439	C35	2012
2.197225	0.333333	13.23211	2.079442	1	2	2.70805	C25	2009
2.197225	0.333333	13.40468	1.791759	1	2	2.772589	C25	2010
2.197225	0.333333	13.37	1.791759	1	2	2.833213	C25	2011
2.197225	0.333333	13.50224	2.197225	1	2	2.890372	C25	2012
2.197225	0.333333	13.45448	1.94591	1	2	2.944439	C25	2013
2.484907	0.333333	13.25742	2.70805	1	2	2.995732	C25	2014
2.484907	0.333333	13.92741	2.564949	1	1	3.044522	C25	2015
2.484907	0.333333	13.97175	2.995732	1	1	3.091042	C25	2016
2.484907	0.333333	13.298	2.197225	1	2	3.135494	I64	2017
2.197225	0.333333	13.79531	2.995732	1	3	2.484907	I64	2009
2.197225	0.333333	13.79531	2.944439	1	3	2.564949	K70	2010
2.197225	0.333333	13.89247	2.772589	1	3	2.639057	C39	2011
2.197225	0.333333	13.98102	3.091042	1	3	2.70805	C38	2012
2.079442	0.375	14.09314	2.772589	1	3	2.772589	C38	2013
2.197225	0.333333	14.123	2.890372	1	3	2.833213	C38	2014
2.397895	0.363636	14.62199	3.044522	1	3	2.890372	C38	2015
2.197225	0.333333	15.36322	2.70805	1	3	3.178054	K70	2013
2.197225	0.333333	15.47166	2.564949	1	3	3.218876	K70	2014
2.197225	0.333333	15.5323	2.833213	1	3	3.258097	K70	2015
2.197225	0.333333	15.45813	2.890372	1	3	3.295837	K70	2016
2.197225	0.333333	14.94132	3.044522	1	3	3.332205	K70	2017
2.079442	0.375	15.57364	2.833213	1	3	3.367296	K70	2018
2.197225	0.333333	16.07856	3.258097	1	3	3.401197	K70	2019
2.197225	0.333333	14.20179	1.386294	1	5	2.484907	C15	2009
2.197225	0.333333	14.86633	1.609438	1	5	2.564949	C15	2010
2.197225	0.333333	14.96848	1.386294	1	5	2.639057	C15	2011
2.197225	0.333333	15.06458	1.609438	1	5	2.70805	C15	2012
2.197225	0.333333	15.02447	1.94591	1	5	2.944439	C15	2016
2.197225	0.333333	14.26659	1.098612	1	5	2.995732	C15	2017
2.197225	0.333333	14.77102	2.197225	1	5	3.044522	C15	2018
2.197225	0.333333	14.75552	1.609438	1	5	3.091042	C15	2019
2.197225	0.333333	13.39071	1.94591	1	2	2.397895	C13	2009
2.197225	0.333333	14.72385	2.397895	1	2	2.564949	C13	2011
2.197225	0.333333	14.72385	2.079442	1	2	2.639057	C13	2012
2.197225	0.333333	14.65146	1.609438	1	2	2.70805	C13	2013
2.197225	0.333333	14.76755	2.197225	1	2	2.772589	C13	2014
2.197225	0.333333	14.8664	2.564949	1	2	2.833213	C13	2015
2.079442	0.375	14.50395	2.772589	1	2	2.890372	C13	2016
1.94591	0.428571	14.86532	2.397895	1	2	2.944439	C13	2017

1.94591	0.428571	15.33334	2.833213	1	2	2.995732	C13	2018
2.079442	0.375	15.84556	2.70805	1	2	3.044522	C13	2019
1.94591	0.428571	13.03244	2.772589	1	1	2.397895	C37	2009
1.94591	0.428571	14.11294	2.302585	1	1	2.484907	C29	2010
1.94591	0.428571	14.13214	2.397895	1	1	2.564949	C29	2011
1.94591	0.428571	14.1274	2.484907	1	1	2.639057	C29	2012
1.94591	0.428571	14.2003	2.079442	1	1	2.70805	C29	2013
1.609438	0.6	14.43366	2.079442	1	1	2.772589	C29	2014
1.609438	0.6	14.47572	1.94591	1	1	2.833213	C29	2015
1.609438	0.6	14.68814	1.94591	1	1	2.890372	C29	2016
1.94591	0.428571	14.80876	2.397895	1	1	2.944439	C29	2017
1.94591	0.428571	14.88022	2.197225	1	1	2.995732	C29	2018
1.94591	0.428571	14.88022	2.197225	1	1	3.044522	C29	2019
1.609438	0.4	15.14235	2.302585	0	1	3.044522	R86	2018
2.484907	0.333333	13.3487	2.197225	1	4	2.772589	C34	2014
2.484907	0.333333	14.43221	2.197225	1	4	2.833213	C27	2015
2.397895	0.363636	14.55621	2.079442	1	4	2.890372	C27	2016
2.197225	0.444444	14.4694	2.197225	1	4	2.944439	C27	2017
2.079442	0.375	14.46314	1.94591	1	4	2.995732	C27	2018
2.197225	0.333333	14.25377	1.94591	1	3	2.302585	C22	2009
2.197225	0.333333	14.37284	2.079442	1	3	2.397895	C20	2010
2.197225	0.333333	14.44252	1.791759	1	3	2.484907	C20	2011
2.197225	0.333333	14.43517	2.197225	1	3	2.564949	C20	2012
2.197225	0.333333	14.42054	1.609438	1	3	2.639057	C20	2013
2.197225	0.333333	14.59616	1.94591	1	3	2.70805	C20	2014
2.079442	0.375	13.70937	2.197225	1	3	2.772589	C20	2015
2.079442	0.375	13.81681	2.302585	1	3	2.833213	C20	2016
1.94591	0.428571	14.07734	2.397895	1	3	2.890372	C20	2017
1.791759	0.5	13.42039	2.564949	1	4	2.944439	C20	2018
1.94591	0.428571	14.24352	1.791759	1	4	2.995732	C20	2019
2.197225	0.333333	14.7847	2.302585	1	4	2.890372	C37	2017
2.197225	0.333333	14.92156	2.079442	1	4	2.944439	C37	2018
2.079442	0.375	15.08	2.079442	1	4	2.995732	C37	2019
2.197225	0.333333	14.13178	2.079442	1	4	2.397895	E47	2009
2.197225	0.333333	14.82493	2.397895	1	4	2.484907	E47	2010
2.197225	0.333333	14.82493	2.397895	1	4	2.564949	E47	2011
2.197225	0.333333	14.92901	2.484907	1	4	2.639057	E48	2012
2.197225	0.333333	14.92901	3.135494	1	4	2.70805	E48	2013
2.197225	0.333333	14.96132	2.833213	1	4	2.772589	E48	2014
2.079442	0.375	14.96132	2.772589	1	4	2.833213	E48	2015
2.197225	0.333333	15.60727	3.091042	1	4	2.890372	E48	2016
2.484907	0.333333	15.51296	2.890372	1	4	2.944439	E48	2017
2.484907	0.333333	17.31649	3.218876	1	4	2.995732	K70	2018
2.397895	0.363636	17.5264	2.995732	1	4	3.044522	K70	2019
2.197225	0.333333	14.90407	2.079442	1	4	2.484907	F51	2011
2.197225	0.333333	14.98489	2.079442	1	4	2.564949	F52	2012
2.197225	0.333333	14.95654	1.791759	1	4	2.639057	F52	2013
2.197225	0.333333	15.05678	2.079442	1	4	2.70805	F52	2014
2.197225	0.333333	15.07113	2.197225	1	4	2.772589	F52	2015
2.197225	0.333333	14.99108	2.079442	1	4	2.833213	F52	2016
2.197225	0.333333	15.09644	1.94591	1	4	2.890372	F52	2017
2.197225	0.333333	15.2018	2.079442	1	4	2.944439	F52	2018
2.197225	0.333333	15.31959	2.079442	1	4	2.995732	F52	2019
2.197225	0.333333	15.58024	2.079442	1	3	3.135494	C28	2016
2.197225	0.333333	15.53828	2.397895	1	3	3.178054	C37	2017
2.079442	0.375	15.50929	2.397895	1	3	3.218876	C37	2018
1.94591	0.428571	15.55598	2.197225	1	3	3.258097	C37	2019
2.197225	0.333333	15.5564	2.944439	1	4	2.833213	K70	2015

2.197225	0.333333	15.59796	2.944439	1	4	2.890372	K70	2016
2.302585	0.4	12.79386	2.079442	1	2	2.397895	C17	2009
2.197225	0.444444	12.79386	1.609438	1	2	2.484907	C17	2010
2.197225	0.444444	13.43425	2.484907	1	2	2.564949	C17	2011
2.197225	0.444444	13.95443	1.94591	1	2	2.639057	C17	2012
2.197225	0.444444	13.96272	1.609438	1	2	2.70805	C17	2013
2.197225	0.333333	13.4	2.079442	1	3	2.772589	C27	2015
2.079442	0.375	13.78505	1.94591	1	3	2.833213	C27	2016
2.197225	0.333333	13.78505	2.302585	1	3	2.890372	C27	2017
2.197225	0.333333	13.77885	2.397895	1	3	2.944439	C27	2018
2.079442	0.375	14.2157	1.609438	1	3	2.995732	C27	2019
2.197225	0.333333	13.35043	1.386294	1	1	2.995732	C37	2009
2.397895	0.363636	13.84351	1.609438	1	1	3.044522	C37	2010
2.397895	0.363636	13.77385	2.302585	1	1	3.091042	C37	2011
2.397895	0.363636	14.24287	2.079442	1	1	3.135494	C37	2012
2.397895	0.363636	14.19764	2.302585	1	1	3.178054	C37	2013
2.197225	0.333333	14.3634	1.94591	1	1	3.218876	C37	2014
2.197225	0.333333	14.3544	1.94591	1	1	3.258097	C37	2015
2.197225	0.333333	14.55968	2.079442	1	1	3.295837	C37	2016
2.197225	0.333333	14.92325	2.197225	1	1	3.332205	C37	2017
2.197225	0.333333	15.07536	2.197225	1	1	3.367296	C37	2018
2.197225	0.333333	15.14484	1.94591	1	1	3.401197	C37	2019
2.197225	0.333333	13.41099	2.197225	1	5	2.079442	C27	2009
2.197225	0.333333	13.44053	2.302585	1	5	2.197225	C27	2010
1.94591	0.428571	13.44053	2.564949	1	5	2.302585	C27	2011
2.397895	0.363636	13.71602	2.639057	1	5	2.397895	C27	2012
2.302585	0.4	13.99783	2.564949	1	5	2.484907	C26	2013
2.197225	0.333333	14.12388	2.70805	1	5	2.564949	C26	2014
2.484907	0.333333	14.69015	2.639057	1	5	2.639057	C26	2015
2.484907	0.333333	14.69762	1.94591	1	5	2.70805	C26	2016
2.484907	0.333333	14.69762	1.94591	1	5	2.772589	C26	2017
2.197225	0.333333	14.7116	2.079442	1	5	2.833213	C26	2018
2.197225	0.333333	14.66464	1.94591	1	5	2.890372	C26	2019
2.079442	0.375	12.90246	2.639057	1	2	2.564949	C38	2009
2.079442	0.375	13.28142	2.772589	1	2	2.639057	C38	2010
2.197225	0.333333	13.42336	2.484907	1	2	2.70805	C38	2011
2.197225	0.333333	13.85184	2.484907	1	2	2.772589	C38	2012
2.079442	0.375	14.45915	2.302585	1	2	2.833213	C38	2013
2.197225	0.333333	14.59474	2.302585	1	2	2.890372	C38	2014
2.197225	0.333333	14.68286	2.079442	1	2	2.944439	C38	2015
2.197225	0.333333	14.7442	2.079442	1	2	2.995732	C38	2016
2.197225	0.333333	13.98787	1.791759	1	2	3.044522	C38	2017
2.197225	0.333333	13.66539	1.94591	1	2	2.197225	C35	2009
2.197225	0.333333	13.92517	2.397895	1	2	2.302585	C35	2010
2.197225	0.333333	14.05412	2.197225	1	2	2.397895	C35	2011
2.197225	0.333333	13.77427	2.302585	1	2	2.484907	C35	2012
2.197225	0.333333	14.1271	2.079442	1	2	2.70805	C35	2015
2.197225	0.333333	14.31598	2.079442	1	2	2.772589	C35	2016
2.197225	0.333333	14.40407	2.564949	1	2	2.833213	C35	2017
2.197225	0.333333	14.38942	2.079442	1	2	2.890372	C35	2018
2.197225	0.333333	14.58724	2.079442	1	2	2.944439	C35	2019
2.197225	0.333333	14.4033	1.791759	1	3	2.397895	C27	2011
1.94591	0.428571	14.3694	1.791759	1	3	2.484907	C27	2012
1.94591	0.428571	14.42528	2.079442	1	3	2.564949	C27	2013
1.94591	0.428571	14.46261	2.197225	1	3	2.639057	C27	2014
1.94591	0.428571	14.50866	1.791759	1	3	2.70805	C27	2015
1.94591	0.428571	14.57163	1.609438	1	3	2.772589	C27	2016
1.94591	0.428571	14.61302	1.386294	1	3	2.833213	C27	2017

1.94591	0.428571	14.63969	1.791759	1	3	2.890372	C27	2018
1.94591	0.428571	14.63969	1.791759	1	3	2.944439	C27	2019
2.197225	0.333333	13.10216	2.197225	1	2	2.197225	C35	2009
2.197225	0.333333	13.10216	2.397895	1	2	2.302585	C35	2010
2.197225	0.333333	13.10216	2.197225	1	2	2.397895	C35	2011
2.197225	0.333333	13.52783	2.197225	1	2	2.484907	C34	2012
2.197225	0.333333	13.71015	2.302585	1	2	2.564949	C34	2013
2.197225	0.333333	13.91082	2.564949	1	2	2.639057	C34	2014
2.197225	0.333333	13.71015	2.564949	1	2	2.70805	C34	2015
2.197225	0.333333	13.71015	2.639057	1	2	2.772589	C34	2016
2.197225	0.333333	14.37513	2.397895	1	2	2.833213	C34	2017
2.079442	0.375	13.79531	2.70805	1	2	2.890372	C34	2018
2.197225	0.333333	14.04296	2.484907	1	2	2.944439	C34	2019
2.197225	0.333333	14.46481	2.079442	1	1	2.079442	C26	2009
2.197225	0.333333	14.58775	2.079442	1	4	2.197225	C26	2010
2.197225	0.333333	14.74444	2.639057	1	4	2.302585	C26	2011
2.197225	0.333333	14.81275	2.397895	1	1	2.397895	C26	2012
2.197225	0.333333	14.98098	1.94591	1	4	2.484907	C26	2013
2.197225	0.333333	15.02447	1.386294	1	1	2.564949	C26	2014
2.197225	0.333333	15.36307	2.70805	1	1	2.639057	C26	2015
2.197225	0.333333	15.54539	2.302585	1	2	2.70805	C26	2016
2.197225	0.333333	15.56297	2.772589	1	1	2.772589	C26	2017
2.197225	0.333333	15.58877	2.302585	1	2	2.833213	L72	2018
2.197225	0.333333	15.74993	2.833213	1	4	2.890372	L72	2019
2.197225	0.333333	14.42305	2.197225	1	5	2.890372	C17	2018
2.197225	0.333333	14.99312	2.772589	1	5	2.944439	D44	2019
2.197225	0.333333	14.98719	2.564949	1	1	2.639057	K70	2014
2.197225	0.333333	13.69898	1.94591	1	2	2.397895	C26	2009
2.197225	0.333333	13.768	1.94591	1	2	2.484907	C26	2010
1.791759	0.333333	13.92005	1.94591	1	4	2.197225	C26	2009
1.791759	0.333333	13.95048	2.197225	1	4	2.564949	C27	2013
2.197225	0.333333	13.20302	2.197225	1	4	2.70805	C35	2009
2.197225	0.333333	13.104	2.302585	1	4	2.772589	C35	2010
2.484907	0.333333	13.35775	2.484907	1	4	2.833213	C35	2011
2.484907	0.333333	13.59922	2.197225	1	4	2.890372	C35	2012
2.484907	0.333333	13.72219	2.197225	1	4	2.944439	C35	2013
1.791759	0.333333	14.44054	2.639057	1	4	2.995732	C35	2014
2.197225	0.444444	12.92098	2.079442	1	3	2.197225	G58	2009
2.197225	0.444444	13.05708	2.197225	1	3	2.302585	G58	2010
2.079442	0.5	13.14217	1.94591	1	3	2.397895	G58	2011
2.197225	0.444444	13.20799	2.484907	1	3	2.484907	C37	2012
2.197225	0.444444	13.63423	2.397895	1	3	2.564949	C37	2013
2.197225	0.444444	14.79335	2.197225	1	4	2.890372	C37	2018
2.197225	0.333333	13.8643	1.609438	1	2	2.397895	C33	2009
2.197225	0.333333	13.81551	2.079442	1	2	2.484907	C33	2010
2.197225	0.333333	13.8643	1.94591	1	2	2.564949	C33	2011
2.079442	0.375	13.90169	1.791759	1	2	2.639057	C33	2012
2.197225	0.333333	13.92884	2.397895	1	2	2.70805	C33	2013
2.197225	0.333333	14.04662	2.197225	1	2	2.772589	C33	2014
2.197225	0.333333	14.01436	2.302585	1	2	2.833213	C33	2015
2.197225	0.333333	14.05058	2.079442	1	2	2.890372	C33	2016
2.197225	0.333333	14.27243	1.94591	1	2	2.944439	C33	2017
2.197225	0.333333	14.44965	1.386294	1	2	2.995732	C33	2018
2.197225	0.333333	14.25377	1.791759	1	4	3.044522	C33	2019
1.94591	0.571429	16.34793	1.791759	1	3	2.890372	L72	2019
2.197225	0.444444	13.51441	2.079442	0	1	2.079442	C18	2009
2.197225	0.444444	13.88317	2.079442	0	1	2.197225	C18	2010
2.197225	0.444444	14.28176	2.484907	0	1	2.302585	C18	2011

2.197225	0.444444	14.31629	2.197225	1	1	2.397895	C18	2012
2.197225	0.444444	14.4033	2.079442	1	2	2.484907	C18	2013
2.197225	0.444444	14.38082	2.197225	1	4	2.564949	C18	2014
2.197225	0.444444	14.28551	2.484907	1	4	2.639057	C18	2015
2.197225	0.444444	14.18015	2.079442	1	4	2.70805	C18	2016
2.197225	0.444444	14.32233	1.791759	1	4	2.772589	C18	2017
2.197225	0.444444	14.35783	1.791759	1	4	2.833213	C18	2018
2.197225	0.444444	14.54547	1.94591	1	4	2.890372	C18	2019
2.197225	0.333333	13.61657	1.609438	1	5	2.772589	N78	2017
2.197225	0.333333	12.41876	1.94591	1	4	2.397895	C17	2009
2.197225	0.333333	12.64013	1.098612	1	4	2.484907	C17	2010
2.197225	0.333333	13.21804	2.197225	1	4	2.564949	C17	2011
2.197225	0.333333	13.48701	1.609438	1	4	2.639057	C17	2012
2.197225	0.333333	13.69898	2.564949	1	4	2.70805	C17	2013
2.197225	0.333333	13.74294	2.079442	1	4	2.772589	C17	2014
2.197225	0.333333	13.82546	2.079442	1	4	2.833213	C17	2015
2.197225	0.333333	13.94654	2.079442	1	4	2.890372	C17	2016
2.197225	0.333333	13.42985	2.639057	1	4	2.944439	C17	2017
2.079442	0.375	13.42985	2.70805	1	4	2.995732	N77	2018
2.197225	0.333333	13.65299	2.397895	1	4	3.044522	N77	2019
2.397895	0.363636	12.6885	1.609438	1	2	2.197225	A01	2009
2.197225	0.444444	12.73552	1.609438	1	2	2.302585	A01	2010
2.197225	0.444444	12.87493	2.197225	1	2	2.397895	A01	2011
2.197225	0.444444	12.87493	1.386294	1	2	2.484907	A01	2012
2.484907	0.333333	12.88131	1.386294	1	2	2.564949	A01	2013
2.484907	0.333333	12.71168	1.609438	1	2	2.639057	A01	2014
2.484907	0.333333	13.01922	1.609438	1	2	2.70805	A01	2015
2.484907	0.333333	13.59237	2.197225	1	2	2.772589	A01	2016
2.484907	0.333333	13.59237	1.791759	1	2	2.833213	A01	2017
2.484907	0.333333	13.59237	1.609438	1	2	2.890372	A01	2018
2.197225	0.333333	12.85579	1.94591	1	2	2.944439	A01	2019
2.197225	0.333333	13.56628	2.302585	1	4	2.772589	C39	2009
2.197225	0.333333	13.56628	2.564949	1	4	2.833213	C39	2010
2.197225	0.333333	14.57012	2.397895	1	4	2.890372	C39	2011
2.079442	0.375	14.1263	2.397895	1	4	2.944439	C39	2012
2.197225	0.333333	14.6136	2.302585	1	4	2.995732	C39	2013
2.197225	0.333333	14.63203	2.197225	1	4	3.044522	C39	2014
2.197225	0.333333	14.6076	2.397895	1	4	3.091042	C39	2015
2.197225	0.333333	14.88201	1.791759	1	4	3.135494	C39	2016
2.197225	0.333333	14.97944	2.079442	1	4	3.178054	C39	2017
2.197225	0.333333	14.79699	2.70805	1	4	3.218876	C39	2018
2.397895	0.363636	14.94388	2.397895	1	4	3.258097	C39	2019
2.079442	0.375	13.13528	2.197225	1	3	2.079442	C39	2009
2.197225	0.333333	12.943	1.94591	1	4	2.079442	C34	2009
2.197225	0.333333	13.71004	1.791759	1	4	2.197225	C34	2010
2.197225	0.333333	13.89561	2.564949	1	4	2.302585	C34	2011
2.197225	0.333333	13.99131	2.197225	1	4	2.397895	C34	2012
2.197225	0.333333	14.11294	2.197225	1	4	2.484907	C34	2013
2.197225	0.333333	14.35696	1.609438	1	4	2.564949	C34	2014
2.197225	0.333333	14.34949	2.197225	1	4	2.639057	C34	2015
2.197225	0.333333	14.55157	1.94591	1	4	2.70805	C34	2016
2.197225	0.333333	14.86223	2.197225	1	4	2.772589	C34	2017
2.197225	0.333333	14.9079	1.94591	1	4	2.833213	C34	2018
2.197225	0.333333	14.94229	1.609438	1	4	2.890372	C34	2019
2.197225	0.333333	14.4782	1.94591	1	2	2.70805	E47	2009
2.197225	0.333333	14.57632	2.484907	1	2	2.772589	E47	2010
2.197225	0.333333	14.62644	2.484907	1	2	2.833213	E47	2011
2.197225	0.333333	14.65276	1.791759	1	2	2.890372	E48	2012

2.079442	0.375	14.7034	2.397895	1	2	2.944439	E48	2013
2.079442	0.375	14.7116	1.791759	1	2	2.995732	E48	2014
2.197225	0.333333	14.72779	2.302585	1	2	3.044522	E48	2015
2.197225	0.333333	14.74373	2.197225	1	2	3.091042	E48	2016
2.197225	0.333333	14.7318	2.079442	1	2	3.135494	E48	2017
2.197225	0.333333	14.80133	2.484907	1	2	3.178054	E48	2018
2.197225	0.333333	14.94691	2.079442	1	2	3.218876	E48	2019
2.197225	0.333333	14.40922	1.386294	1	2	2.302585	C28	2009
2.197225	0.333333	14.5403	1.609438	1	2	2.397895	C28	2010
2.079442	0.375	14.48477	1.791759	1	2	2.484907	C28	2011
2.197225	0.333333	14.5262	1.94591	1	2	2.564949	C28	2012
2.197225	0.333333	14.74893	1.386294	1	2	2.639057	C28	2013
2.197225	0.333333	14.70344	2.079442	1	2	2.70805	C28	2014
2.197225	0.333333	14.83987	2.079442	1	2	2.772589	C28	2015
2.197225	0.333333	14.94624	1.609438	1	2	2.833213	C28	2016
2.197225	0.333333	15.23618	1.386294	1	2	2.890372	C28	2017
2.197225	0.333333	15.56185	1.791759	1	2	2.944439	C28	2018
2.197225	0.333333	15.94744	2.302585	1	2	2.995732	C28	2019
2.302585	0.4	14.34142	2.564949	1	3	2.397895	I65	2013
2.302585	0.4	14.28301	2.772589	1	3	2.484907	I65	2014
2.302585	0.4	14.36767	2.833213	1	3	2.564949	I65	2015
2.302585	0.4	14.38394	2.833213	1	3	2.639057	I65	2016
2.302585	0.4	14.41654	2.564949	1	3	2.70805	I65	2017
2.397895	0.454545	14.44625	2.197225	1	3	2.772589	I65	2018
2.397895	0.454545	14.53967	2.484907	1	3	2.833213	I65	2019
2.079442	0.375	12.97154	1.791759	1	2	1.94591	C17	2009
1.94591	0.428571	12.89922	2.197225	1	2	2.079442	C17	2010
1.609438	0.6	12.9146	2.079442	1	2	2.197225	C17	2011
1.609438	0.6	12.94801	2.079442	1	2	2.302585	C17	2012
1.609438	0.6	12.94801	2.079442	1	2	2.397895	C17	2013
1.609438	0.6	12.94801	2.484907	1	2	2.484907	C17	2014
1.609438	0.6	12.94801	1.94591	1	2	2.564949	C17	2015
1.609438	0.6	12.94801	2.197225	1	2	2.639057	C17	2016
2.197225	0.333333	13.86373	2.197225	1	1	2.079442	C33	2009
2.197225	0.333333	14.31629	1.94591	1	1	2.197225	C33	2010
2.197225	0.333333	14.09314	1.94591	1	1	2.302585	C33	2011
2.197225	0.333333	13.91771	1.609438	1	1	2.397895	C33	2012
2.079442	0.375	13.80647	1.94591	1	1	2.484907	C33	2013
2.197225	0.333333	13.40724	2.397895	1	2	2.564949	R86	2014
2.079442	0.375	13.8439	2.302585	1	2	2.639057	R86	2015
2.079442	0.375	13.73939	1.791759	1	2	2.70805	R86	2016
2.079442	0.375	13.51549	3.044522	1	2	2.772589	R86	2017
2.079442	0.375	13.60922	2.564949	1	1	2.833213	R86	2018
2.197225	0.333333	11.65269	2.397895	1	3	2.564949	C17	2013
2.197225	0.333333	12.34583	2.564949	1	3	2.639057	C17	2014
2.079442	0.375	13.91635	2.484907	1	4	2.833213	C38	2015
1.94591	0.428571	14.34012	2.484907	1	4	2.890372	C38	2016
2.197225	0.333333	14.87122	2.397895	1	4	2.944439	C38	2017
2.197225	0.333333	14.96956	2.197225	1	4	2.995732	C38	2018
2.197225	0.333333	15.22777	2.397895	1	4	3.044522	C38	2019
1.94591	0.428571	14.07402	2.397895	1	1	2.484907	C31	2011
1.94591	0.428571	13.98102	1.609438	1	1	2.564949	C31	2012
1.94591	0.428571	13.92436	1.609438	1	1	2.639057	C31	2013
1.94591	0.428571	13.78402	2.079442	1	1	2.70805	C31	2014
1.94591	0.428571	13.87623	1.791759	1	1	2.772589	C31	2015
1.94591	0.428571	14.53754	1.94591	1	1	2.833213	C31	2016
1.94591	0.428571	14.58876	1.791759	1	1	2.890372	C31	2017
1.94591	0.428571	14.59758	1.791759	1	1	2.995732	C31	2019

1.94591	0.428571	13.88317	1.94591	1	3	1.609438	C39	2009
1.94591	0.428571	13.86715	1.94591	1	3	1.791759	C39	2010
1.791759	0.5	13.91445	1.791759	1	3	1.94591	C38	2011
1.94591	0.428571	13.91445	1.94591	1	3	2.079442	C38	2012
1.94591	0.428571	14.00613	2.079442	1	3	2.197225	C38	2013
1.94591	0.428571	14.00613	2.079442	1	3	2.302585	C38	2014
1.94591	0.428571	14.15198	2.70805	1	3	2.397895	C38	2015
1.94591	0.428571	13.9544	2.564949	1	3	2.484907	C38	2016
1.94591	0.428571	13.65181	2.639057	1	3	2.564949	C38	2017
1.94591	0.428571	13.54028	2.079442	1	2	2.197225	C22	2009
1.94591	0.428571	13.56435	2.197225	1	2	2.302585	C22	2010
1.94591	0.428571	13.55818	2.079442	1	2	2.397895	C22	2011
1.94591	0.428571	13.55894	2.079442	1	2	2.484907	C22	2012
1.94591	0.428571	13.83217	2.079442	1	2	2.564949	C22	2013
1.94591	0.428571	13.88885	2.079442	1	2	2.639057	C22	2014
1.94591	0.428571	13.91816	2.302585	1	2	2.70805	C22	2015
1.94591	0.428571	13.98787	2.197225	1	2	2.772589	C22	2016
1.94591	0.428571	14.00984	2.397895	1	2	2.833213	C22	2017
1.94591	0.428571	14.01919	2.197225	1	2	2.890372	C22	2018
1.94591	0.428571	13.97286	2.197225	1	2	2.944439	C22	2019
2.197225	0.333333	13.91082	1.609438	1	2	2.302585	C32	2009
2.197225	0.333333	14.03865	2.079442	1	2	2.397895	C32	2010
2.079442	0.375	14.2602	2.079442	1	2	2.484907	C32	2011
2.197225	0.333333	13.84183	1.791759	1	2	2.564949	C32	2012
2.197225	0.333333	14.12666	1.791759	1	2	2.639057	C32	2013
2.197225	0.333333	14.12666	1.791759	1	2	2.70805	C32	2014
2.079442	0.375	14.19395	2.079442	1	2	2.772589	C32	2015
2.079442	0.375	14.2602	1.791759	1	2	2.833213	C32	2016
2.397895	0.363636	12.87108	1.609438	1	1	1.94591	C17	2009
2.397895	0.363636	12.79386	1.791759	1	1	2.079442	C17	2010
2.197225	0.333333	12.65396	1.94591	1	1	2.197225	C17	2011
2.197225	0.333333	11.79056	1.791759	1	1	2.302585	C17	2012
2.197225	0.333333	11.8706	1.94591	1	1	2.397895	C17	2013
2.197225	0.333333	12.91411	2.079442	1	1	2.484907	C17	2014
2.197225	0.333333	12.53177	1.609438	1	1	2.564949	C17	2015
2.197225	0.333333	12.5532	2.079442	1	1	2.639057	C17	2016
2.197225	0.333333	13.61645	1.94591	1	1	2.70805	C17	2017
2.197225	0.333333	14.02155	2.302585	1	1	2.772589	C17	2018
2.197225	0.333333	13.72065	2.302585	1	1	2.833213	C17	2019
2.197225	0.333333	14.29769	2.079442	1	3	2.397895	C33	2014
2.197225	0.333333	14.25943	2.484907	1	3	2.484907	C33	2015
2.079442	0.375	14.1591	2.639057	1	3	2.564949	C33	2016
2.079442	0.375	14.44225	2.197225	1	3	2.639057	C33	2017
2.197225	0.333333	14.20125	2.564949	1	3	2.70805	C33	2018
2.197225	0.333333	14.02973	2.302585	1	3	2.772589	C33	2019
2.197225	0.333333	13.98289	1.94591	0	4	2.079442	C36	2009
2.197225	0.333333	14.06705	2.397895	0	4	2.197225	C36	2010
2.197225	0.333333	13.88485	2.302585	0	4	2.302585	C36	2011
2.197225	0.333333	13.97708	2.197225	0	4	2.397895	C36	2012
2.197225	0.333333	14.19072	2.079442	0	4	2.484907	C36	2013
2.197225	0.333333	14.41764	1.791759	0	4	2.564949	C36	2014
2.197225	0.333333	14.39122	2.484907	0	4	2.639057	C36	2015
2.197225	0.333333	14.73779	2.197225	0	4	2.70805	C36	2016
2.197225	0.333333	14.44262	2.197225	0	4	2.772589	C36	2017
2.197225	0.333333	14.62337	2.833213	0	4	2.833213	C36	2018
2.197225	0.333333	14.35031	2.079442	0	4	2.890372	C36	2019
2.197225	0.333333	12.67608	1.791759	1	3	2.079442	A04	2009
2.197225	0.333333	12.67608	2.079442	1	3	2.197225	A04	2010

2.197225	0.333333	12.67608	1.609438	1	3	2.302585	A04	2011
2.079442	0.375	13.30468	1.609438	1	3	2.397895	A04	2012
2.197225	0.333333	13.4	1.94591	1	3	2.484907	A04	2013
2.197225	0.333333	13.30468	1.386294	1	3	2.564949	A04	2014
2.197225	0.333333	13.30468	2.079442	1	3	2.639057	A04	2015
2.197225	0.333333	13.30468	2.302585	1	3	2.70805	A04	2016
2.197225	0.333333	13.48701	2.197225	1	3	2.772589	A04	2017
2.197225	0.333333	13.2894	1.94591	0	4	2.484907	C39	2009
2.079442	0.375	13.67395	2.197225	0	4	2.564949	C39	2010
2.197225	0.333333	14.02666	2.484907	0	4	2.639057	C39	2011
2.197225	0.333333	14.01052	2.564949	0	4	2.70805	C39	2012
2.197225	0.333333	13.97831	2.564949	0	4	2.772589	C39	2013
2.197225	0.333333	13.99675	2.397895	0	4	2.833213	C39	2014
2.197225	0.333333	13.97746	2.484907	0	4	2.890372	C39	2015
2.197225	0.333333	13.99173	2.484907	0	4	2.944439	C39	2016
2.197225	0.333333	14.15576	2.397895	0	4	2.995732	C39	2017
2.197225	0.333333	14.45647	2.484907	1	2	3.044522	C39	2018
1.94591	0.428571	14.77788	2.197225	1	3	2.197225	I63	2009
1.94591	0.428571	14.69895	1.609438	1	3	2.302585	I63	2010
1.791759	0.5	14.38292	2.079442	1	3	2.397895	I63	2011
1.94591	0.428571	14.38485	1.94591	1	3	2.484907	I65	2012
1.94591	0.428571	13.78226	2.197225	1	3	2.564949	I65	2013
1.94591	0.428571	14.0837	1.94591	1	3	2.639057	I65	2014
1.94591	0.428571	13.96995	1.94591	1	3	2.70805	I65	2015
1.94591	0.428571	13.94689	2.197225	1	3	2.772589	I65	2016
1.94591	0.428571	14.11004	1.791759	1	3	2.833213	I65	2017
1.94591	0.428571	14.40424	2.079442	1	3	2.890372	I65	2018
1.94591	0.428571	14.27471	1.94591	1	3	2.944439	I65	2019
2.397895	0.363636	13.87284	1.791759	1	4	2.079442	C35	2009
2.397895	0.363636	14.13149	2.302585	1	4	2.197225	C35	2010
2.302585	0.4	13.97635	1.94591	1	4	2.302585	C35	2011
2.302585	0.4	14.15198	2.197225	1	4	2.397895	C35	2012
1.94591	0.428571	14.46784	2.197225	1	4	2.484907	C35	2013
1.94591	0.428571	14.28207	1.791759	1	4	2.564949	C35	2014
1.94591	0.428571	14.02569	2.197225	1	4	2.639057	C35	2015
1.94591	0.428571	14.37272	2.564949	1	4	2.70805	C35	2016
1.791759	0.5	14.75868	2.302585	1	4	2.772589	C35	2017
1.791759	0.5	14.76283	2.197225	1	4	2.833213	C35	2018
2.079442	0.375	14.12402	1.386294	1	3	1.609438	C27	2009
2.197225	0.333333	14.60397	2.079442	1	3	1.791759	C27	2010
2.197225	0.333333	14.37513	1.791759	1	3	1.94591	C27	2011
2.197225	0.333333	14.30409	1.609438	1	3	2.079442	C27	2012
2.079442	0.375	14.5112	1.94591	1	3	2.197225	C27	2013
2.197225	0.333333	14.49689	1.94591	1	4	2.302585	C27	2014
2.079442	0.375	14.38626	2.484907	1	4	2.397895	C27	2015
2.197225	0.333333	14.49962	2.484907	1	4	2.484907	C27	2016
2.197225	0.333333	14.46025	1.94591	1	4	2.564949	C27	2017
2.197225	0.333333	14.61446	1.94591	1	4	2.639057	C27	2018
2.197225	0.333333	14.45894	1.791759	1	4	2.70805	C27	2019
2.197225	0.333333	13.00313	2.197225	1	4	1.94591	C30	2009
2.197225	0.333333	13.00313	2.397895	1	4	2.079442	C30	2010
2.197225	0.333333	13.0056	2.302585	1	4	2.197225	C30	2011
2.197225	0.333333	13.09664	2.484907	1	4	2.397895	C30	2013
2.197225	0.333333	13.13725	2.639057	1	4	2.484907	C30	2014
2.197225	0.333333	13.13804	2.639057	1	4	2.564949	C30	2015
2.197225	0.333333	13.13804	2.564949	1	2	2.639057	C27	2016
2.197225	0.333333	13.98102	2.564949	1	1	2.70805	C27	2017
2.197225	0.333333	13.53381	1.791759	1	2	2.564949	C41	2009

2.197225	0.333333	13.43571	2.079442	1	2	2.639057	C41	2010
2.079442	0.375	14.00646	1.791759	1	2	2.70805	C39	2011
2.079442	0.375	14.13124	2.197225	1	2	2.772589	C39	2012
2.197225	0.333333	14.6189	2.302585	1	2	2.833213	C39	2013
2.197225	0.333333	14.31822	1.791759	1	2	2.890372	C39	2014
2.197225	0.333333	14.59612	1.94591	1	2	2.944439	C39	2015
1.94591	0.428571	14.37353	2.397895	1	2	2.995732	C39	2016
2.397895	0.363636	13.9168	1.94591	1	3	1.791759	C24	2009
2.397895	0.363636	13.98952	1.94591	1	3	1.94591	C24	2010
2.397895	0.363636	14.23685	1.94591	1	3	2.079442	C24	2011
2.397895	0.363636	14.08531	1.94591	1	3	2.197225	C37	2012
2.397895	0.363636	14.4818	1.94591	1	3	2.302585	C37	2013
2.397895	0.363636	14.45087	1.94591	1	3	2.397895	C37	2014
2.397895	0.363636	14.38332	1.94591	1	3	2.484907	C37	2015
2.397895	0.363636	14.38451	1.94591	1	3	2.564949	C37	2016
2.397895	0.363636	14.39644	1.94591	1	3	2.639057	C37	2017
2.397895	0.363636	14.48759	1.94591	1	3	2.70805	C37	2018
2.397895	0.363636	14.78276	1.94591	1	3	2.772589	C37	2019
2.397895	0.363636	13.16158	2.302585	1	3	1.94591	C35	2009
2.397895	0.363636	13.25339	2.079442	1	3	2.079442	C35	2010
2.197225	0.333333	13.40378	2.484907	1	3	2.197225	C35	2011
2.197225	0.333333	13.54278	2.079442	1	3	2.302585	C35	2012
2.079442	0.375	13.30934	2.302585	1	3	2.397895	C35	2013
2.079442	0.375	13.5163	2.564949	1	3	2.484907	C35	2014
2.197225	0.333333	13.68426	2.772589	1	3	2.564949	C35	2015
2.197225	0.333333	13.99382	2.397895	1	3	2.639057	C35	2016
2.197225	0.333333	14.21777	2.564949	1	3	2.70805	C35	2017
2.197225	0.333333	14.17577	2.484907	1	3	2.772589	C35	2018
2.197225	0.333333	14.31526	2.197225	1	3	2.833213	C35	2019
1.94591	0.428571	12.3371	1.791759	1	4	2.079442	C27	2009
1.94591	0.428571	12.28303	2.397895	1	4	2.197225	C27	2010
1.791759	0.5	13.13033	2.197225	1	4	2.302585	C27	2011
1.94591	0.428571	13.13033	2.302585	1	4	2.397895	C27	2012
1.94591	0.428571	13.08154	2.484907	1	4	2.484907	C27	2013
1.94591	0.428571	13.08154	2.564949	1	4	2.564949	C27	2014
1.609438	0.6	13.12974	2.70805	1	4	2.639057	C27	2015
1.94591	0.428571	13.1587	3.091042	1	4	2.70805	C27	2016
1.94591	0.428571	13.15734	2.772589	1	4	2.772589	C27	2017
1.94591	0.428571	13.19932	2.833213	1	4	2.833213	C27	2018
1.94591	0.428571	13.83512	2.995732	1	4	2.890372	C27	2019
2.197225	0.333333	13.56705	1.609438	1	1	1.94591	C39	2009
2.197225	0.333333	13.91987	1.791759	1	1	2.079442	C39	2010
1.94591	0.428571	13.64579	2.197225	1	1	2.197225	C39	2011
1.94591	0.428571	13.66979	1.791759	1	1	2.302585	C39	2012
1.94591	0.428571	13.66979	1.609438	1	1	2.397895	C39	2013
1.94591	0.428571	13.66283	1.791759	1	4	2.484907	C39	2014
2.079442	0.375	14.34614	1.94591	1	3	1.94591	C34	2009
2.079442	0.375	14.42528	1.609438	1	3	2.079442	C34	2010
2.197225	0.333333	14.42528	2.079442	1	3	2.197225	C34	2011
2.197225	0.333333	14.29174	1.791759	1	3	2.302585	C34	2012
2.197225	0.333333	14.2602	1.609438	1	3	2.397895	C34	2013
2.197225	0.333333	14.09314	1.791759	1	3	2.484907	C34	2014
2.197225	0.333333	14.09314	2.484907	1	3	2.564949	C34	2015
2.197225	0.333333	14.09314	2.397895	1	3	2.639057	C34	2016
2.197225	0.333333	13.9447	2.995732	1	3	2.70805	C34	2017
2.197225	0.444444	12.91238	1.386294	1	2	2.302585	C36	2009
2.197225	0.444444	13.34005	2.079442	1	2	2.397895	C36	2010
2.197225	0.444444	13.8883	2.484907	1	2	2.484907	C36	2011

2.397895	0.363636	14.26071	2.302585	1	2	2.564949	C36	2012
2.397895	0.363636	14.17472	2.197225	1	2	2.639057	C36	2013
2.197225	0.333333	14.72534	2.197225	1	2	2.70805	C36	2014
2.197225	0.333333	14.93164	2.079442	1	2	2.772589	C36	2015
2.197225	0.333333	15.0081	2.079442	1	2	2.833213	C36	2016
2.197225	0.333333	15.00664	2.197225	1	2	2.890372	C36	2017
2.197225	0.333333	15.19629	2.197225	1	2	2.944439	C36	2018
2.197225	0.333333	15.55768	1.609438	1	2	2.995732	C36	2019
2.197225	0.333333	12.76569	2.484907	1	4	2.639057	C33	2009
2.197225	0.333333	12.70685	2.70805	1	4	2.70805	C33	2010
2.197225	0.333333	12.68973	2.397895	1	4	2.772589	C33	2011
2.197225	0.333333	12.70685	2.484907	1	4	2.833213	C33	2012
2.397895	0.363636	12.75823	2.484907	1	4	2.890372	C33	2013
2.397895	0.363636	13.04115	2.197225	1	4	2.944439	C33	2014
2.397895	0.363636	12.95678	2.70805	1	4	2.995732	C33	2015
2.197225	0.333333	12.7367	2.890372	1	4	3.044522	C33	2016
2.197225	0.333333	13.775	2.772589	1	4	3.091042	C33	2017
2.197225	0.333333	13.77469	2.944439	1	4	3.135494	C33	2018
2.197225	0.333333	13.71026	2.484907	1	4	3.178054	C33	2019
2.197225	0.333333	14.53822	2.995732	1	4	1.609438	K70	2009
2.197225	0.333333	14.31021	2.70805	1	4	1.791759	K70	2010
2.197225	0.333333	14.89732	2.564949	1	4	1.94591	K70	2011
2.197225	0.333333	14.61751	2.944439	1	4	2.079442	K70	2012
2.197225	0.333333	14.50866	3.178054	1	4	2.197225	K70	2013
2.197225	0.333333	14.60397	2.833213	1	4	2.302585	K70	2014
2.197225	0.333333	14.59484	2.639057	1	4	2.397895	K70	2015
2.197225	0.333333	14.55267	3.465736	1	4	2.484907	K70	2016
2.197225	0.333333	14.90009	3.433987	1	4	2.639057	K70	2018
2.197225	0.333333	15.07153	3.496508	1	4	2.70805	K70	2019
2.079442	0.375	12.93241	2.397895	1	2	2.484907	C17	2009
2.197225	0.333333	12.9473	2.397895	1	2	2.564949	C17	2010
2.197225	0.333333	12.81312	1.386294	1	2	2.639057	C17	2011
2.197225	0.333333	12.90917	2.079442	1	2	2.70805	C17	2012
2.197225	0.333333	13.55024	2.302585	1	2	2.772589	C17	2013
2.197225	0.333333	13.62059	1.94591	1	2	2.833213	C17	2014
2.197225	0.333333	13.76074	1.609438	1	2	2.890372	C17	2015
2.197225	0.333333	14.19216	1.791759	1	2	2.944439	C17	2016
2.197225	0.333333	14.33144	1.386294	1	2	2.995732	C17	2017
2.197225	0.333333	14.38451	1.386294	1	2	3.044522	C17	2018
2.197225	0.333333	14.50781	1.791759	1	2	3.091042	C17	2019
2.484907	0.333333	13.79531	1.609438	1	1	1.94591	C33	2009
2.484907	0.333333	13.91987	1.791759	1	1	2.079442	C33	2010
2.397895	0.363636	13.95527	1.791759	1	1	2.197225	C33	2011
2.197225	0.333333	14.15198	2.079442	1	1	2.302585	C33	2012
2.197225	0.333333	14.06237	1.386294	1	1	2.397895	C33	2013
2.197225	0.333333	14.2173	1.609438	1	1	2.484907	C33	2014
1.94591	0.428571	14.37912	1.609438	1	1	2.564949	C33	2015
1.94591	0.428571	14.94724	2.197225	1	1	2.639057	C33	2016
1.94591	0.428571	15.12384	2.397895	1	1	2.70805	C33	2017
1.94591	0.428571	15.94136	1.94591	1	1	2.772589	C33	2018
1.94591	0.428571	16.06533	1.386294	1	1	2.833213	C33	2019
2.197225	0.333333	13.86706	1.94591	1	4	2.079442	C18	2009
2.197225	0.333333	14.20661	2.564949	1	4	2.197225	C18	2010
2.197225	0.333333	15.4854	2.890372	1	4	2.302585	C18	2011
2.197225	0.333333	15.71517	1.94591	1	4	2.397895	C18	2012
1.94591	0.428571	15.6272	2.484907	1	4	2.484907	C18	2013
1.94591	0.428571	15.60289	1.791759	1	4	2.564949	C18	2014
1.94591	0.428571	14.84513	1.94591	1	4	2.639057	C18	2015

1.94591	0.428571	14.58488	1.94591	1	4	2.70805	C18	2016
1.94591	0.428571	14.54658	2.397895	1	4	2.772589	C18	2017
1.791759	0.5	14.99831	2.079442	1	4	2.833213	C18	2018
1.94591	0.428571	15.1006	1.94591	1	4	2.890372	C18	2019
2.397895	0.363636	13.89303	2.197225	1	3	2.70805	C39	2009
2.397895	0.363636	14.41209	2.079442	1	3	2.772589	C39	2010
2.397895	0.363636	14.26958	2.079442	1	3	2.833213	C39	2011
2.302585	0.4	14.17305	1.94591	1	3	2.890372	C39	2012
2.397895	0.363636	14.55463	1.791759	1	3	2.944439	C39	2013
2.397895	0.363636	14.69493	2.079442	1	3	2.995732	C39	2014
2.397895	0.363636	14.89484	2.397895	1	3	3.044522	C39	2015
2.397895	0.363636	15.20702	2.079442	1	3	3.135494	C39	2017
2.079442	0.375	15.1027	1.791759	1	3	3.218876	C39	2019
1.609438	0.4	12.83628	2.397895	1	4	2.564949	C13	2009
1.609438	0.4	12.0137	1.791759	1	4	2.639057	C13	2010
1.609438	0.4	13.03462	2.302585	1	4	2.70805	C13	2011
1.609438	0.4	13.11836	2.197225	1	4	2.772589	C13	2012
1.609438	0.4	13.33424	2.484907	1	4	2.833213	C13	2013
1.609438	0.4	12.30592	2.197225	1	4	2.890372	C13	2014
1.609438	0.4	13.67326	2.70805	1	4	2.944439	C13	2015
1.609438	0.4	13.45023	2.944439	1	4	2.995732	C13	2016
1.609438	0.4	13.7212	2.833213	1	4	3.044522	C13	2017
1.609438	0.4	13.79019	2.995732	1	4	3.091042	C13	2018
1.609438	0.4	13.76422	3.091042	1	4	3.135494	C13	2019
2.197225	0.333333	14.26429	3.091042	1	3	2.397895	C34	2009
2.197225	0.333333	14.3257	3.091042	1	3	2.484907	C34	2010
2.197225	0.333333	14.78451	3.091042	1	3	2.564949	C34	2011
2.197225	0.333333	15.07525	3.091042	1	3	2.639057	C34	2012
2.197225	0.333333	14.8837	3.091042	1	3	2.70805	C34	2013
2.197225	0.333333	14.98166	3.091042	1	3	2.772589	C34	2014
2.197225	0.333333	14.98275	3.091042	1	3	2.833213	C34	2015
2.197225	0.333333	15.00904	3.091042	1	3	2.890372	C34	2016
2.197225	0.333333	15.20796	3.091042	1	3	2.944439	C34	2017
2.197225	0.333333	15.25333	3.091042	1	3	2.995732	C34	2018
2.197225	0.333333	15.222	3.091042	1	3	3.044522	C34	2019
2.197225	0.333333	14.96323	1.386294	1	4	2.70805	C26	2009
2.197225	0.333333	14.60351	1.791759	1	4	2.772589	C26	2010
2.197225	0.333333	14.50841	1.609438	1	4	2.833213	C26	2011
2.197225	0.333333	14.45931	2.197225	1	4	2.890372	C26	2012
2.197225	0.333333	14.65319	1.791759	1	4	2.944439	C26	2013
2.197225	0.333333	14.39666	1.609438	1	4	2.995732	C26	2014
2.079442	0.375	14.82095	1.791759	1	4	3.044522	C26	2015
2.197225	0.333333	14.84916	2.302585	1	4	3.091042	C26	2016
2.197225	0.333333	14.92292	1.94591	1	4	3.135494	C26	2017
2.197225	0.333333	14.73627	1.94591	1	4	3.178054	C26	2018
2.197225	0.333333	14.93337	1.94591	1	4	3.218876	C26	2019
1.94591	0.428571	13.41965	2.302585	1	4	1.791759	C38	2009
1.94591	0.428571	13.67395	2.302585	1	4	1.94591	C38	2010
1.94591	0.428571	13.67395	2.079442	1	4	2.079442	C38	2011
2.197225	0.333333	13.67188	1.791759	0	3	2.197225	C38	2012
1.94591	0.428571	13.58986	2.302585	0	3	2.302585	C38	2013
1.94591	0.428571	14.07787	2.833213	0	3	2.397895	C38	2014
2.197225	0.333333	12.95323	2.079442	1	2	2.079442	C28	2009
2.197225	0.333333	13.13033	2.197225	1	2	2.197225	C28	2010
2.197225	0.333333	13.43571	2.079442	1	2	2.397895	C28	2012
2.197225	0.333333	13.67279	2.302585	1	2	2.484907	C28	2013
2.197225	0.333333	13.6517	2.302585	1	2	2.564949	C28	2014
2.197225	0.333333	13.65475	1.791759	1	2	2.639057	C28	2015

2.197225	0.333333	13.84507	2.484907	1	2	2.70805	C28	2016
2.197225	0.333333	13.84507	2.079442	1	2	2.772589	C28	2017
2.197225	0.333333	14.41873	2.890372	1	2	2.833213	C28	2018
2.079442	0.375	14.11146	2.197225	1	2	2.890372	C28	2019
2.197225	0.333333	14.27786	2.70805	0	1	2.639057	Q83	2017
2.197225	0.333333	14.69514	2.639057	1	1	2.70805	Q83	2018
2.197225	0.333333	12.79386	2.079442	1	4	2.639057	C41	2009
2.197225	0.333333	12.79386	2.079442	1	4	2.70805	C41	2010
2.197225	0.333333	12.79386	2.484907	1	4	2.772589	C41	2011
2.197225	0.333333	12.79386	2.197225	1	4	2.833213	C21	2012
2.197225	0.333333	12.79386	2.079442	1	4	2.890372	C21	2013
1.609438	0.4	12.34583	1.94591	1	2	2.079442	C40	2009
1.609438	0.4	12.82126	1.386294	1	2	2.197225	C40	2010
1.609438	0.4	13.10216	2.197225	1	2	2.302585	C40	2011
1.609438	0.4	13.08154	2.197225	1	2	2.397895	C40	2012
1.609438	0.4	13.41503	2.079442	1	2	2.484907	C40	2013
2.197225	0.333333	14.00116	2.397895	1	2	2.564949	C40	2014
2.197225	0.333333	13.91987	2.772589	1	2	2.639057	C40	2015
2.079442	0.375	13.8116	2.564949	1	2	2.70805	I64	2016
2.197225	0.333333	14.2805	1.609438	1	3	1.791759	C39	2009
2.197225	0.333333	14.62002	1.94591	1	3	1.94591	C39	2010
2.197225	0.333333	14.53174	2.302585	1	3	2.079442	C39	2011
2.197225	0.333333	14.70003	2.079442	1	3	2.197225	C39	2012
2.197225	0.333333	15.1065	2.397895	1	3	2.302585	C39	2013
2.197225	0.333333	15.18918	2.197225	1	3	2.397895	C39	2014
2.197225	0.333333	15.08521	2.079442	1	3	2.484907	C39	2015
2.197225	0.333333	15.22864	1.791759	1	3	2.564949	C39	2016
2.197225	0.333333	15.34764	2.079442	1	3	2.639057	C39	2017
2.197225	0.333333	15.33261	2.079442	1	3	2.70805	C39	2018
2.197225	0.333333	15.50339	2.079442	1	3	2.772589	C39	2019
1.94591	0.428571	13.09909	2.197225	1	4	3.218876	C17	2018
1.94591	0.428571	13.17439	2.302585	1	4	3.258097	C17	2019
2.197225	0.333333	13.40302	1.94591	1	2	1.94591	C39	2009
2.197225	0.333333	13.44835	1.791759	1	2	2.079442	C39	2010
2.197225	0.333333	13.40181	1.94591	1	2	2.197225	C39	2011
2.197225	0.333333	13.37685	1.94591	1	2	2.302585	C39	2012
2.197225	0.333333	13.30801	1.791759	1	2	2.397895	C39	2013
1.94591	0.428571	13.87858	1.791759	1	2	2.484907	C39	2014
2.197225	0.333333	14.07626	2.197225	1	2	2.564949	C39	2015
2.079442	0.375	14.19182	1.791759	1	2	2.639057	C39	2016
2.079442	0.375	15.02721	2.197225	1	2	2.833213	C39	2019
2.197225	0.333333	12.79885	1.609438	1	4	2.079442	C35	2009
2.197225	0.333333	13.03681	1.791759	1	4	2.197225	C35	2010
2.197225	0.333333	13.03681	1.94591	1	4	2.302585	C35	2011
2.197225	0.333333	12.80573	2.079442	1	4	2.397895	C35	2012
2.079442	0.375	12.59405	1.94591	1	4	2.484907	C35	2013
2.197225	0.333333	12.22734	2.484907	1	4	2.564949	C35	2014
2.079442	0.375	12.12378	2.564949	1	4	2.639057	C35	2015
2.197225	0.333333	12.77875	1.94591	1	4	2.70805	C35	2016
2.197225	0.333333	12.77875	1.791759	1	4	2.772589	C35	2017
1.94591	0.428571	12.97618	2.079442	1	2	2.70805	C30	2009
1.94591	0.428571	13.00897	1.94591	1	2	2.772589	C30	2010
2.079442	0.375	13.50626	2.302585	1	2	2.833213	C30	2011
2.197225	0.333333	13.82268	2.197225	1	2	2.890372	C30	2012
2.197225	0.333333	13.99783	2.197225	1	2	2.944439	C30	2013
2.197225	0.333333	13.99783	2.197225	1	2	2.995732	C30	2014
2.197225	0.333333	14.36213	1.94591	1	2	3.044522	C30	2015
2.197225	0.333333	14.30163	2.70805	1	2	3.091042	C30	2016

2.197225	0.333333	14.47282	2.302585	1	2	3.135494	C30	2017
2.197225	0.333333	14.63604	1.94591	1	2	3.178054	C30	2018
2.197225	0.333333	13.89617	1.94591	1	2	2.079442	C32	2009
2.197225	0.333333	13.90105	2.079442	1	2	2.197225	C32	2010
2.197225	0.333333	14.06666	1.791759	1	2	2.302585	C32	2011
2.197225	0.333333	14.09178	1.791759	1	2	2.397895	C32	2012
2.197225	0.333333	13.76569	1.791759	1	2	2.484907	C32	2013
2.197225	0.333333	14.08881	1.609438	1	2	2.564949	C32	2014
2.197225	0.333333	14.1901	2.302585	1	2	2.639057	C32	2015
2.197225	0.333333	14.25028	2.484907	1	4	2.70805	C32	2016
2.197225	0.333333	14.51215	2.302585	1	2	2.772589	C32	2017
2.197225	0.333333	14.82704	2.639057	1	4	2.833213	C32	2018
2.197225	0.333333	14.8709	2.484907	1	2	2.890372	C32	2019
2.397895	0.363636	15.1538	1.791759	1	1	2.079442	C28	2009
2.397895	0.363636	15.05918	2.197225	1	1	2.197225	C28	2010
2.397895	0.363636	15.17971	2.197225	1	1	2.302585	C28	2011
2.397895	0.363636	15.1469	1.94591	1	1	2.397895	C28	2012
2.197225	0.333333	15.16926	2.197225	1	1	2.484907	C28	2013
2.197225	0.444444	15.28631	2.484907	1	1	2.564949	C28	2014
2.197225	0.444444	15.38413	2.484907	1	1	2.639057	C28	2015
1.94591	0.428571	15.32892	2.079442	1	1	2.70805	C28	2016
1.94591	0.428571	15.44475	2.302585	1	1	2.772589	C28	2017
1.94591	0.428571	15.43292	2.079442	1	1	2.833213	C28	2018
1.94591	0.428571	15.47944	2.197225	1	1	2.890372	C28	2019
2.197225	0.333333	13.35348	1.609438	1	1	1.94591	C26	2009
2.197225	0.333333	13.71015	2.197225	1	1	2.079442	C26	2010
2.197225	0.333333	13.45884	2.302585	1	1	2.197225	C26	2011
2.197225	0.333333	12.99453	2.772589	1	1	2.302585	C26	2012
1.94591	0.428571	13.30801	2.397895	1	1	2.484907	C26	2014
1.94591	0.428571	13.08154	2.639057	1	1	2.564949	C26	2015
1.791759	0.5	13.14803	2.564949	1	1	2.639057	C26	2016
1.791759	0.5	13.10623	1.94591	1	1	2.70805	C26	2017
1.609438	0.4	12.94801	2.397895	1	1	2.772589	C26	2018
2.197225	0.333333	13.39254	2.639057	1	2	3.178054	C38	2009
2.197225	0.333333	13.41518	2.484907	1	2	3.218876	C38	2010
2.197225	0.333333	13.75385	2.302585	1	2	3.258097	C38	2011
2.197225	0.333333	13.78608	2.197225	1	2	3.295837	C38	2012
2.197225	0.333333	13.86115	2.484907	1	2	3.332205	C38	2013
2.197225	0.333333	14.00149	2.197225	1	2	3.367296	C38	2014
2.197225	0.333333	14.09026	2.079442	1	2	3.401197	C38	2015
2.197225	0.333333	14.14919	2.302585	1	2	3.433987	C38	2016
2.197225	0.333333	14.40496	2.564949	1	2	3.465736	C38	2017
2.197225	0.333333	14.35329	2.397895	1	2	3.496508	C38	2018
2.197225	0.333333	14.35364	2.484907	1	2	3.526361	C38	2019
2.197225	0.333333	12.57072	1.94591	1	4	2.079442	C14	2009
2.197225	0.333333	12.57072	2.079442	1	4	2.197225	C14	2010
2.197225	0.333333	13.26542	1.94591	1	4	2.302585	C14	2011
2.197225	0.333333	13.38473	1.791759	1	4	2.397895	C14	2012
2.197225	0.333333	13.38473	1.791759	1	4	2.484907	C14	2013
2.197225	0.333333	13.38473	2.302585	1	4	2.564949	C14	2014
2.302585	0.4	13.38473	2.079442	1	3	2.639057	C14	2015
2.197225	0.333333	13.38473	2.079442	1	4	2.70805	C14	2016
2.197225	0.333333	13.38473	2.302585	1	3	2.772589	C14	2017
2.197225	0.333333	13.38473	2.302585	1	3	2.833213	C14	2018
2.197225	0.333333	14.03865	2.197225	1	3	2.890372	C14	2019
2.197225	0.333333	13.46397	2.397895	0	4	0.693147	C39	2009
2.197225	0.333333	13.89229	2.302585	0	4	1.098612	C39	2010
2.197225	0.333333	13.77469	2.197225	0	4	1.386294	C39	2011

2.197225	0.333333	13.82546	2.772589	1	4	1.609438	C39	2012
2.197225	0.333333	13.92095	2.772589	1	4	1.791759	C39	2013
2.079442	0.375	14.00456	2.772589	0	4	1.94591	C38	2014
2.197225	0.333333	14.27887	2.397895	0	4	2.079442	C38	2015
2.197225	0.333333	14.37518	2.197225	0	4	2.197225	C38	2016
2.197225	0.333333	14.43705	2.639057	0	3	2.302585	C38	2017
2.197225	0.333333	14.50052	2.397895	0	4	2.397895	C38	2018
2.197225	0.333333	14.43893	2.397895	0	4	2.484907	C38	2019
1.94591	0.428571	15.33769	2.564949	1	1	2.564949	F51	2009
1.94591	0.428571	13.73538	2.302585	0	1	2.639057	F51	2010
1.94591	0.428571	13.78855	2.639057	0	1	2.70805	F51	2011
1.94591	0.428571	13.77423	2.079442	1	1	2.772589	F51	2012
1.94591	0.428571	13.92607	2.197225	1	1	2.890372	F51	2014
2.197225	0.333333	13.79531	1.94591	1	4	0.693147	C35	2009
2.197225	0.333333	13.83531	1.791759	1	4	1.098612	C35	2010
2.197225	0.333333	14.24078	2.197225	1	4	1.386294	C35	2011
2.197225	0.333333	14.41435	1.94591	1	4	1.609438	C35	2012
2.197225	0.333333	14.40884	2.079442	1	4	1.791759	C35	2013
2.197225	0.333333	14.2933	1.791759	1	4	1.94591	C35	2014
2.197225	0.333333	14.76597	2.639057	1	4	2.079442	C35	2015
2.197225	0.333333	14.96383	2.397895	1	4	2.197225	C35	2016
2.079442	0.375	15.10843	2.397895	1	4	2.302585	C35	2017
2.079442	0.375	15.1382	1.94591	1	4	2.397895	C35	2018
2.079442	0.375	15.71004	1.94591	1	4	2.484907	C35	2019
1.609438	0.4	13.21767	2.197225	1	2	1.94591	C29	2009
1.609438	0.4	13.48506	2.197225	1	2	2.079442	C29	2010
1.609438	0.4	13.4931	2.397895	1	2	2.197225	C29	2011
1.609438	0.4	13.55253	2.302585	1	2	2.302585	C29	2012
1.609438	0.4	14.32046	2.564949	1	2	2.397895	C29	2013
1.609438	0.4	13.62556	2.079442	1	4	2.484907	C29	2014
2.079442	0.375	13.61278	2.639057	1	4	2.564949	C29	2015
2.079442	0.375	13.61718	2.70805	1	2	2.639057	C29	2016
1.94591	0.428571	13.92158	2.639057	1	2	2.70805	C29	2017
1.94591	0.428571	14.4363	2.564949	0	4	2.772589	C29	2018
1.94591	0.428571	14.47757	2.484907	0	2	2.833213	C29	2019
2.397895	0.363636	14.25473	2.197225	0	2	1.94591	C30	2009
2.397895	0.363636	14.57804	2.079442	0	2	2.079442	C30	2010
2.397895	0.363636	14.35426	2.197225	0	2	2.197225	C30	2011
2.397895	0.363636	14.62644	2.079442	0	2	2.302585	C30	2012
2.302585	0.4	14.47878	2.772589	0	2	2.397895	C30	2013
2.302585	0.4	14.39229	2.197225	0	2	2.484907	C30	2014
2.197225	0.333333	14.32299	2.397895	1	3	2.564949	C30	2015
2.197225	0.333333	14.14776	1.94591	1	3	2.639057	C30	2016
2.079442	0.375	14.24091	1.94591	1	3	2.70805	C30	2017
2.079442	0.375	14.36381	2.397895	1	3	2.772589	C30	2018
2.197225	0.333333	14.49446	2.772589	1	3	2.833213	C30	2019
1.94591	0.428571	13.43498	2.197225	1	3	0.693147	C22	2009
1.94591	0.428571	13.49462	2.397895	1	3	1.098612	C22	2010
1.94591	0.428571	13.66213	2.197225	1	3	1.386294	C22	2011
2.397895	0.363636	13.80202	1.94591	1	3	1.609438	C22	2012
2.397895	0.363636	13.89792	2.197225	1	3	1.791759	C22	2013
2.397895	0.363636	13.8993	2.079442	1	3	1.94591	C22	2014
2.397895	0.363636	13.83884	1.791759	1	3	2.079442	C22	2015
2.397895	0.363636	13.8813	2.70805	0	3	2.197225	C22	2016
2.397895	0.363636	13.94943	2.079442	0	3	2.302585	C22	2017
2.397895	0.363636	14.38847	2.302585	0	3	2.397895	C22	2018
2.197225	0.333333	13.82268	1.94591	1	3	2.397895	C39	2011
2.197225	0.333333	14.2456	1.94591	1	3	2.484907	C39	2012

2.197225	0.333333	13.95205	2.302585	1	3	2.564949	C39	2013
2.197225	0.333333	13.98356	2.079442	1	3	2.639057	C39	2014
2.197225	0.333333	14.02739	2.564949	1	3	2.70805	C39	2015
2.197225	0.333333	14.10684	2.397895	1	3	2.772589	C39	2016
2.197225	0.333333	13.86601	2.484907	1	3	0.693147	C30	2009
2.197225	0.333333	13.83717	2.772589	1	3	1.098612	C30	2010
2.197225	0.333333	14.08935	2.197225	1	3	1.386294	C30	2011
2.197225	0.333333	15.07594	2.302585	1	3	1.609438	C30	2012
1.94591	0.428571	13.99449	2.302585	1	3	1.791759	C30	2013
1.94591	0.428571	14.73894	1.94591	1	3	1.94591	C30	2014
1.94591	0.428571	15.27543	2.397895	1	3	2.079442	C30	2015
1.609438	0.4	14.68055	2.639057	1	3	2.197225	C30	2016
1.609438	0.4	15.59806	1.94591	1	3	2.302585	C30	2017
1.609438	0.4	16.13704	2.197225	1	3	2.397895	C30	2018
1.609438	0.4	14.97716	2.397895	1	3	2.484907	C30	2019
2.197225	0.333333	14.2602	2.079442	1	2	2.484907	A03	2009
2.197225	0.333333	14.23078	1.609438	1	2	2.564949	A03	2010
2.197225	0.333333	14.10422	2.079442	1	2	2.639057	A03	2011
2.197225	0.333333	14.50085	2.197225	1	2	2.70805	A03	2012
2.197225	0.333333	16.01274	1.791759	1	2	2.944439	A03	2016
2.197225	0.333333	16.01135	1.94591	1	2	3.044522	A03	2018
2.197225	0.333333	12.48824	1.94591	1	2	1.94591	C18	2009
2.197225	0.333333	12.4232	1.386294	1	2	2.079442	C18	2010
2.197225	0.333333	12.498	1.609438	1	2	2.197225	C18	2011
2.197225	0.333333	12.50284	2.079442	1	2	2.302585	C18	2012
2.079442	0.375	12.61918	1.94591	1	2	2.397895	C18	2013
2.079442	0.375	12.71379	1.94591	1	2	2.484907	C18	2014
2.197225	0.333333	14.33834	2.484907	1	2	2.564949	C18	2015
2.079442	0.375	15.00024	2.197225	1	2	2.639057	C36	2016
2.197225	0.333333	14.13687	2.079442	1	2	2.70805	C36	2017
2.197225	0.444444	14.18942	2.197225	1	4	2.079442	C20	2009
2.197225	0.444444	14.50264	2.079442	1	4	2.197225	C20	2010
2.197225	0.444444	14.77616	1.791759	1	4	2.302585	C20	2011
2.197225	0.444444	14.05594	1.94591	1	4	2.397895	C20	2012
2.079442	0.5	14.78807	1.94591	1	4	2.484907	C20	2013
2.197225	0.444444	14.68797	2.302585	1	4	2.564949	C20	2014
1.94591	0.428571	15.04471	2.302585	1	4	2.639057	C20	2015
1.94591	0.428571	15.13213	2.890372	1	4	2.70805	C20	2016
1.94591	0.428571	14.77868	2.890372	1	4	1.098612	K70	2009
1.94591	0.428571	14.92077	3.091042	1	4	1.386294	K70	2010
1.94591	0.428571	14.92077	2.70805	1	4	1.609438	K70	2011
1.94591	0.428571	14.92077	2.995732	1	4	1.94591	K70	2013
1.94591	0.428571	14.92077	2.302585	1	4	2.079442	K70	2014
1.94591	0.428571	14.92077	3.332205	1	4	2.197225	K70	2015
1.94591	0.428571	14.92077	3.367296	1	4	2.302585	K70	2016
1.94591	0.428571	14.92077	3.044522	1	4	2.397895	K70	2017
1.94591	0.428571	14.92077	3.091042	1	4	2.484907	K70	2018
1.94591	0.428571	14.92077	3.178054	1	4	2.564949	K70	2019
2.197225	0.333333	12.9057	2.302585	1	2	0.693147	O81	2009
2.197225	0.333333	13.54501	2.302585	1	2	1.098612	O81	2010
2.197225	0.333333	14.40663	1.94591	1	2	1.386294	O81	2011
2.197225	0.333333	14.4044	1.609438	1	2	1.609438	G58	2012
2.197225	0.333333	13.999	1.94591	1	2	1.791759	G58	2013
2.197225	0.333333	13.81511	2.197225	1	2	1.94591	G58	2014
2.197225	0.333333	13.8006	1.609438	1	2	2.079442	G58	2015
2.197225	0.333333	13.81331	2.397895	1	2	2.197225	G58	2016
2.197225	0.333333	13.88895	2.079442	1	2	2.302585	G58	2017
2.197225	0.333333	14.12966	1.791759	1	2	2.397895	G58	2018

2.197225	0.333333	14.26032	2.079442	1	2	2.484907	G58	2019
1.791759	0.333333	13.66655	2.197225	1	2	0.693147	C41	2009
1.791759	0.333333	13.90114	2.197225	1	2	1.098612	C41	2010
1.791759	0.333333	14.07787	2.302585	1	2	1.386294	C41	2011
1.791759	0.333333	14.08679	2.484907	1	2	1.609438	C41	2012
1.791759	0.333333	14.3761	2.484907	1	2	1.791759	C41	2013
1.791759	0.333333	14.40413	2.484907	1	2	1.94591	C41	2014
1.791759	0.333333	14.40391	2.484907	1	2	2.079442	C41	2015
1.791759	0.333333	14.45736	2.564949	1	2	2.197225	C41	2016
1.94591	0.428571	14.41654	1.609438	0	1	2.079442	C26	2009
1.94591	0.428571	14.70085	2.197225	1	1	2.197225	C26	2010
1.94591	0.428571	14.84273	2.302585	0	1	2.302585	C26	2011
1.94591	0.428571	14.85352	2.079442	1	1	2.397895	C26	2012
1.94591	0.428571	14.85695	1.94591	1	1	2.484907	C26	2013
1.94591	0.428571	14.91609	2.397895	1	1	2.564949	C26	2014
1.791759	0.5	15.00476	2.079442	1	1	2.639057	C26	2015
1.94591	0.428571	15.36116	2.302585	1	1	2.70805	C26	2016
1.94591	0.428571	15.64615	2.70805	1	1	2.772589	C26	2017
1.94591	0.428571	15.69386	2.564949	1	1	2.833213	C26	2018
1.94591	0.428571	15.88327	2.564949	1	1	2.890372	C26	2019
1.94591	0.428571	13.47583	2.079442	1	3	1.609438	F52	2009
1.791759	0.5	13.59237	1.791759	1	3	1.791759	F52	2010
1.94591	0.428571	13.89709	2.397895	1	3	1.94591	F52	2011
1.94591	0.428571	14.13759	2.197225	1	3	2.079442	F52	2012
1.94591	0.428571	14.21362	2.079442	1	3	2.197225	F52	2013
1.94591	0.428571	14.21362	2.484907	1	3	2.302585	F52	2014
1.94591	0.428571	14.38592	2.197225	1	3	2.397895	F52	2015
1.94591	0.428571	14.38592	2.197225	1	3	2.484907	F52	2016
1.94591	0.428571	14.38592	2.197225	1	3	2.564949	F52	2017
2.197225	0.333333	14.38592	2.484907	1	3	2.639057	F52	2018
2.197225	0.333333	14.38592	2.397895	1	3	2.70805	F52	2019
2.197225	0.333333	15.43806	1.94591	1	5	3.044522	C27	2009
2.197225	0.333333	16.02322	1.791759	1	5	3.178054	C27	2012
2.197225	0.333333	16.18644	2.302585	1	5	3.218876	C27	2013
2.197225	0.333333	16.03206	2.564949	1	5	3.258097	C27	2014
2.197225	0.333333	15.93155	2.197225	1	5	3.295837	C27	2015
2.079442	0.375	15.82766	2.833213	1	3	3.332205	C27	2016
2.079442	0.375	16.03593	2.197225	1	5	3.367296	C27	2017
2.079442	0.375	16.03597	2.197225	1	3	3.433987	C27	2019
2.079442	0.375	13.8112	1.609438	1	3	2.197225	I65	2009
2.197225	0.333333	13.81391	1.609438	1	4	2.302585	I65	2010
2.197225	0.333333	13.90736	2.079442	1	3	2.397895	I65	2011
2.197225	0.333333	13.78557	1.386294	1	4	2.484907	I65	2012
2.197225	0.333333	13.88876	1.791759	1	3	2.564949	I65	2013
2.197225	0.333333	13.87453	1.386294	1	4	2.639057	I65	2014
2.079442	0.375	13.83757	2.197225	1	3	2.70805	I65	2015
2.079442	0.375	14.05098	1.94591	1	4	2.772589	I65	2016
2.197225	0.333333	13.97328	1.94591	1	3	2.833213	I65	2017
2.197225	0.333333	14.07263	1.94591	1	4	2.890372	I65	2018
2.197225	0.333333	14.33787	2.079442	1	3	2.944439	I65	2019
2.197225	0.333333	13.76422	1.609438	1	4	0.693147	C34	2009
2.197225	0.333333	14.3257	2.079442	1	4	1.098612	C34	2010
2.079442	0.375	14.1532	2.197225	1	4	1.386294	C34	2011
2.197225	0.333333	14.36479	1.94591	1	4	1.609438	C34	2012
2.197225	0.333333	14.4231	1.94591	1	4	1.791759	C34	2013
2.197225	0.333333	14.4307	1.791759	1	4	1.94591	C34	2014
2.197225	0.333333	14.30593	2.079442	1	4	2.079442	C34	2015
2.197225	0.333333	14.41983	1.791759	1	4	2.197225	C34	2016

2.197225	0.333333	14.46981	2.197225	1	4	2.302585	C34	2017
2.197225	0.333333	14.4407	2.397895	1	4	2.397895	C34	2018
2.197225	0.333333	13.78195	1.791759	1	3	1.386294	C20	2009
2.197225	0.333333	13.94777	2.302585	1	3	1.609438	C20	2010
2.197225	0.333333	13.89043	2.079442	0	3	1.791759	C20	2011
2.197225	0.333333	13.94285	2.639057	1	4	1.94591	C20	2012
2.079442	0.375	13.81821	2.397895	1	4	2.079442	C20	2013
2.079442	0.375	13.77458	2.302585	1	4	2.197225	C20	2014
1.94591	0.428571	13.91563	2.564949	1	4	2.302585	C20	2015
1.94591	0.428571	13.79459	2.302585	1	4	2.397895	C20	2016
1.94591	0.428571	13.90435	2.302585	1	3	2.484907	D45	2017
2.197225	0.333333	14.48789	2.079442	1	4	2.079442	I63	2009
2.197225	0.333333	14.29794	2.484907	1	4	2.197225	I63	2010
2.197225	0.333333	14.58144	2.197225	1	4	2.302585	I63	2011
2.197225	0.333333	14.4106	1.94591	1	4	2.397895	I65	2012
2.197225	0.333333	14.39353	1.94591	1	4	2.484907	I65	2013
2.197225	0.333333	14.34614	2.639057	1	4	2.564949	I65	2014
1.791759	0.333333	14.30409	2.564949	1	4	2.639057	I65	2015
2.397895	0.363636	14.59079	2.639057	1	4	2.70805	I65	2016
2.397895	0.363636	14.80423	1.94591	1	4	2.772589	I65	2017
2.197225	0.333333	15.43873	1.791759	1	4	2.890372	I65	2019
2.079442	0.375	12.89922	2.484907	1	2	2.197225	C29	2009
1.94591	0.428571	12.7189	2.833213	1	2	2.302585	C29	2010
2.079442	0.375	12.9808	2.564949	1	2	2.397895	C29	2011
1.94591	0.428571	13.79531	2.564949	1	2	2.484907	C29	2012
1.94591	0.428571	13.79531	2.397895	1	2	2.564949	C29	2013
1.94591	0.428571	13.79531	2.484907	1	2	2.639057	C29	2014
1.94591	0.428571	14.1474	2.484907	1	2	2.70805	C29	2015
1.94591	0.428571	14.11413	2.302585	1	2	2.772589	C29	2016
1.609438	0.4	15.05519	1.94591	1	4	2.639057	C18	2014
1.609438	0.4	15.00943	2.079442	1	4	2.70805	C18	2015
1.609438	0.4	15.18227	2.197225	1	4	2.772589	C18	2016
1.94591	0.428571	15.33288	2.302585	1	4	2.833213	C18	2017
1.791759	0.5	15.3458	1.609438	1	4	2.890372	C18	2018
2.079442	0.375	13.58886	2.197225	1	3	2.564949	C34	2015
2.197225	0.333333	14.23547	2.484907	1	3	2.639057	C38	2016
2.197225	0.333333	14.41605	2.397895	1	3	2.70805	C38	2017
2.079442	0.375	14.3664	2.484907	1	3	2.772589	C38	2018
2.197225	0.333333	14.3251	2.484907	1	3	2.833213	C38	2019
2.197225	0.333333	14.37798	2.079442	1	2	1.098612	C39	2009
2.197225	0.333333	14.39038	1.791759	1	2	1.386294	C39	2010
2.197225	0.333333	14.6669	2.484907	1	2	1.609438	C39	2011
2.197225	0.333333	14.78772	2.484907	1	2	1.791759	C39	2012
2.197225	0.333333	14.21032	2.079442	1	2	1.94591	C39	2013
2.197225	0.333333	14.65146	2.302585	1	2	2.079442	C39	2014
2.197225	0.333333	14.17883	2.197225	1	2	2.197225	C39	2015
2.197225	0.333333	14.89823	2.833213	1	2	2.302585	C39	2016
2.197225	0.333333	14.85118	2.890372	1	2	2.397895	C39	2017
2.197225	0.333333	15.41065	2.995732	1	2	2.484907	C39	2018
2.197225	0.333333	15.6535	2.772589	1	2	2.564949	C39	2019
2.079442	0.375	14.47236	2.397895	1	3	2.079442	C27	2009
2.197225	0.333333	14.5779	1.94591	1	3	2.197225	C27	2010
2.197225	0.333333	14.08767	1.94591	1	3	2.302585	C27	2011
2.197225	0.333333	15.15136	1.609438	1	3	2.397895	C27	2012
2.197225	0.333333	15.28391	2.079442	1	3	2.484907	C27	2013
2.197225	0.333333	15.21736	1.609438	1	3	2.564949	C27	2014
2.197225	0.333333	14.73667	1.609438	1	3	2.639057	C27	2015
2.197225	0.333333	14.82369	1.791759	1	3	2.70805	C27	2016

2.197225	0.333333	14.78276	1.098612	1	3	2.772589	C27	2017
2.197225	0.333333	14.72663	1.098612	1	3	2.833213	C27	2018
2.197225	0.333333	14.6172	1.791759	1	3	2.890372	C27	2019
2.197225	0.333333	12.84793	1.791759	1	2	2.564949	C38	2009
2.197225	0.333333	13.16158	2.079442	1	2	2.639057	C38	2010
2.197225	0.333333	13.20854	1.386294	1	2	2.70805	C38	2011
2.197225	0.333333	13.27078	2.397895	1	2	2.772589	C38	2012
2.197225	0.333333	13.76632	2.397895	1	2	2.833213	C38	2013
2.197225	0.333333	13.69221	2.397895	1	2	2.890372	C38	2014
2.197225	0.333333	14.18721	2.484907	1	2	2.944439	C38	2015
2.197225	0.333333	14.30391	2.833213	1	2	2.995732	C38	2016
2.197225	0.333333	14.20091	2.639057	1	2	3.044522	C38	2017
2.197225	0.333333	14.24404	2.302585	1	2	3.091042	C38	2018
2.197225	0.333333	14.4523	1.791759	1	2	3.135494	C38	2019
2.197225	0.333333	14.3737	1.791759	1	4	1.609438	F52	2009
2.197225	0.333333	14.24976	1.94591	1	4	1.791759	F52	2010
2.397895	0.363636	14.56082	2.302585	1	4	1.94591	F52	2011
2.397895	0.363636	14.70278	2.079442	1	4	2.079442	F52	2012
2.397895	0.363636	14.76512	2.484907	1	4	2.197225	F52	2013
2.397895	0.363636	14.68	2.564949	1	4	2.302585	F52	2014
2.397895	0.363636	14.64358	2.484907	1	4	2.397895	F52	2015
2.397895	0.363636	14.63034	2.302585	1	4	2.484907	F52	2016
2.397895	0.363636	14.65552	2.197225	1	4	2.564949	F52	2017
2.397895	0.363636	14.48881	2.302585	1	4	2.639057	F52	2018
2.397895	0.363636	14.3757	2.197225	1	4	2.70805	F52	2019
2.079442	0.375	13.71015	1.098612	1	4	2.772589	C35	2009
2.197225	0.333333	14.10706	2.079442	1	4	2.833213	C35	2010
2.079442	0.375	14.07687	1.94591	1	4	2.890372	C35	2011
2.079442	0.375	14.14862	1.791759	0	4	2.944439	C35	2012
2.197225	0.333333	14.27294	2.197225	0	4	2.995732	C35	2013
2.197225	0.333333	14.20077	1.791759	0	3	3.044522	C35	2014
2.197225	0.333333	13.98009	1.386294	1	3	2.397895	C41	2009
2.197225	0.333333	13.4398	1.94591	1	3	2.484907	C41	2010
2.197225	0.333333	13.7057	1.94591	1	3	2.564949	C34	2011
2.197225	0.333333	13.72482	2.079442	1	3	2.639057	C34	2012
2.197225	0.333333	13.56961	1.609438	1	3	2.70805	C34	2013
2.197225	0.333333	13.66991	1.098612	1	3	2.772589	C34	2014
2.197225	0.333333	13.87	1.791759	1	3	2.833213	C34	2015
2.197225	0.333333	14.00124	1.94591	1	3	2.890372	C34	2016
2.197225	0.333333	14.69953	2.397895	1	3	2.944439	C34	2017
2.484907	0.333333	14.85924	1.791759	1	3	2.995732	C34	2018
2.197225	0.333333	14.9501	1.94591	1	3	3.044522	C34	2019
1.94591	0.428571	14.61644	2.302585	1	5	2.639057	C39	2018
2.079442	0.375	14.94843	2.302585	1	5	2.70805	C39	2019
1.94591	0.428571	14.45441	2.639057	1	4	1.791759	C24	2013
1.94591	0.428571	15.05145	2.639057	1	4	1.94591	C24	2014
1.94591	0.428571	14.63419	2.564949	1	4	2.079442	C24	2015
1.94591	0.428571	15.23797	2.564949	1	4	2.197225	C24	2016
1.94591	0.428571	15.75549	2.564949	1	4	2.302585	C24	2017
1.94591	0.428571	15.29584	1.94591	1	4	2.484907	C24	2019
2.397895	0.363636	14.38179	1.609438	1	1	0.693147	C17	2009
2.484907	0.333333	14.68639	2.397895	1	1	1.098612	C17	2010
2.397895	0.363636	14.66352	2.197225	1	1	1.386294	C17	2011
2.302585	0.4	14.71957	2.197225	1	1	1.609438	C17	2012
2.197225	0.333333	14.73898	1.94591	1	1	1.791759	C17	2013
2.197225	0.333333	14.7716	1.386294	1	1	1.94591	C17	2014
2.197225	0.333333	14.72852	1.609438	1	1	2.079442	C17	2015
2.197225	0.333333	15.11131	1.94591	1	1	2.197225	C17	2016

2.197225	0.333333	15.4782	2.079442	1	1	2.302585	C17	2017
2.197225	0.333333	15.00752	2.079442	1	1	2.397895	C17	2018
2.197225	0.333333	15.07056	2.302585	1	1	2.484907	C17	2019
2.197225	0.333333	15.09191	2.302585	1	3	2.397895	C27	2018
2.197225	0.333333	15.18877	2.302585	1	3	2.484907	C27	2019
2.197225	0.333333	14.55959	1.94591	1	4	2.639057	C32	2019
1.94591	0.428571	13.01098	1.94591	1	3	2.197225	I65	2010
1.94591	0.428571	14.71924	2.079442	1	3	2.302585	I65	2011
1.94591	0.428571	14.33132	1.791759	1	3	2.397895	C39	2012
1.94591	0.428571	14.05193	1.94591	1	3	2.484907	C39	2013
1.609438	0.6	14.03937	2.197225	1	3	2.564949	C39	2014
1.609438	0.6	14.18652	1.94591	1	3	2.639057	C39	2015
1.609438	0.6	14.49851	2.484907	1	3	2.70805	C39	2016
1.609438	0.6	14.18652	2.397895	1	3	2.772589	C39	2017
1.609438	0.6	14.29559	1.609438	1	3	2.833213	C39	2018
1.609438	0.6	15.0469	1.609438	1	3	2.890372	C39	2019
2.197225	0.333333	14.03865	1.791759	0	3	1.098612	A03	2009
2.197225	0.333333	14.11968	1.94591	0	3	1.386294	A03	2010
2.197225	0.333333	14.18707	2.397895	0	3	1.609438	A03	2011
2.197225	0.333333	14.50163	2.639057	1	3	1.791759	A03	2012
2.197225	0.333333	14.52551	2.484907	1	3	1.94591	A03	2013
2.197225	0.333333	14.52551	2.302585	1	3	2.079442	A03	2014
2.197225	0.333333	14.52551	2.197225	1	3	2.197225	A03	2015
2.197225	0.333333	14.52551	1.791759	1	3	2.302585	A03	2016
2.197225	0.333333	14.56386	2.397895	1	3	2.397895	A03	2017
2.197225	0.333333	14.31834	2.079442	1	3	2.484907	A03	2018
2.197225	0.333333	14.11562	1.609438	1	4	2.397895	K70	2009
2.197225	0.333333	13.97251	2.484907	1	4	2.484907	K70	2010
2.197225	0.333333	13.95892	2.197225	1	4	2.564949	K70	2011
2.079442	0.375	14.8815	2.197225	1	4	2.639057	K70	2012
2.079442	0.375	15.69038	2.564949	1	3	2.772589	C39	2018
2.197225	0.333333	14.55124	1.386294	1	2	0.693147	C38	2009
2.197225	0.333333	13.86715	2.397895	1	2	1.098612	C38	2010
2.197225	0.333333	14.05492	2.484907	1	2	1.386294	C38	2011
2.079442	0.375	13.93303	2.484907	1	2	1.609438	C38	2012
2.197225	0.333333	14.10197	1.94591	1	2	1.791759	C38	2013
2.197225	0.333333	14.03545	2.564949	1	2	1.94591	C38	2014
2.197225	0.333333	14.4709	2.564949	1	2	2.079442	C38	2015
2.197225	0.333333	14.58562	2.833213	1	2	2.197225	C38	2016
2.197225	0.333333	14.61504	2.564949	1	2	2.302585	C38	2017
2.197225	0.333333	14.70644	2.833213	1	2	2.397895	C38	2018
2.197225	0.333333	14.75524	2.639057	1	2	2.484907	C38	2019
2.197225	0.333333	13.08445	1.791759	1	4	2.484907	C39	2009
2.197225	0.333333	13.13567	2.197225	1	4	2.564949	C39	2010
2.197225	0.333333	13.42069	2.397895	1	4	2.639057	C39	2011
2.197225	0.333333	13.66128	2.772589	1	4	2.70805	C39	2012
2.197225	0.333333	13.70021	2.564949	1	4	2.772589	C39	2013
2.197225	0.333333	13.59287	2.484907	1	4	2.833213	C39	2014
2.197225	0.333333	13.92544	2.944439	1	4	2.890372	C39	2015
2.197225	0.333333	14.40867	2.995732	1	4	3.044522	C39	2018
1.94591	0.428571	14.75813	2.772589	1	4	3.091042	C26	2019
2.197225	0.333333	13.47302	1.791759	0	3	2.302585	I63	2009
2.079442	0.375	13.47302	2.772589	0	3	2.397895	I63	2010
2.197225	0.333333	13.84972	2.397895	0	3	2.484907	I63	2011
2.197225	0.333333	13.56474	2.890372	0	3	2.70805	I65	2014
1.94591	0.428571	13.74659	2.197225	1	2	2.079442	C27	2009
1.94591	0.428571	13.81381	1.94591	1	2	2.197225	C27	2010
2.197225	0.333333	13.6662	2.079442	1	2	2.302585	C27	2011

2.197225	0.333333	14.12424	1.791759	1	2	2.397895	C27	2012
2.197225	0.333333	14.21764	2.397895	1	2	2.484907	C27	2013
2.197225	0.333333	13.92248	2.484907	1	2	2.564949	C27	2014
2.197225	0.333333	13.92481	2.564949	1	2	2.639057	C27	2015
2.197225	0.333333	13.92248	2.397895	1	2	2.70805	C27	2016
2.197225	0.333333	13.92248	2.302585	1	2	2.772589	C27	2017
2.197225	0.333333	14.06992	2.484907	1	2	2.833213	C27	2018
2.197225	0.333333	12.65715	1.386294	1	4	1.94591	C35	2009
2.197225	0.333333	12.88156	2.639057	1	4	2.079442	C35	2010
2.079442	0.375	13.07737	1.791759	1	4	2.197225	C35	2011
2.079442	0.375	13.46526	2.079442	1	4	2.302585	C35	2012
2.197225	0.333333	13.46453	1.098612	1	4	2.397895	C35	2013
2.197225	0.333333	13.52007	1.791759	1	4	2.484907	C35	2014
2.197225	0.444444	13.69589	0.693147	1	4	0.693147	E50	2009
2.197225	0.444444	13.96182	2.484907	1	4	1.098612	E50	2010
2.197225	0.444444	14.43066	1.94591	1	4	1.386294	E50	2011
2.197225	0.444444	14.59024	2.079442	1	4	1.609438	E50	2012
1.94591	0.428571	14.7652	2.079442	1	4	1.791759	E50	2013
1.94591	0.428571	14.6868	2.197225	1	4	1.94591	E50	2014
1.94591	0.428571	14.62644	2.302585	1	4	2.079442	E50	2015
1.94591	0.428571	14.70751	1.94591	1	4	2.197225	E50	2016
1.94591	0.428571	14.86353	2.484907	1	4	2.302585	E50	2017
1.94591	0.428571	14.96861	2.484907	1	4	2.397895	E50	2018
1.94591	0.428571	15.04903	2.397895	1	4	2.484907	E50	2019
2.197225	0.333333	13.27285	1.609438	1	2	0.693147	C26	2009
2.197225	0.333333	13.40965	2.484907	1	2	1.098612	C26	2010
2.079442	0.375	13.54147	2.302585	1	2	1.386294	C26	2011
1.94591	0.428571	13.58936	2.079442	1	2	1.609438	C26	2012
2.197225	0.333333	13.5861	1.94591	1	2	1.791759	C26	2013
2.197225	0.333333	13.61925	2.890372	1	2	1.94591	C26	2014
2.197225	0.333333	13.63014	3.044522	1	2	2.079442	C26	2015
2.197225	0.333333	13.60392	2.772589	1	2	2.197225	C26	2016
2.197225	0.333333	13.59424	2.197225	0	2	2.302585	C26	2017
2.197225	0.333333	13.56807	2.639057	0	2	2.397895	C26	2018
2.197225	0.333333	14.4033	2.079442	0	2	2.484907	C26	2019
1.791759	0.5	14.46565	1.791759	0	2	2.70805	C17	2009
1.94591	0.571429	14.60981	2.302585	0	2	2.772589	C17	2010
1.791759	0.666667	14.55167	2.397895	0	2	2.833213	C17	2011
1.791759	0.666667	14.65016	2.197225	0	2	2.890372	C17	2012
1.94591	0.571429	14.4942	2.197225	0	2	2.944439	C17	2013
1.94591	0.571429	14.57337	1.94591	0	1	2.995732	C17	2014
1.94591	0.571429	14.52478	2.302585	0	1	3.044522	C17	2015
1.94591	0.571429	14.56073	2.302585	0	1	3.091042	C17	2016
1.94591	0.571429	14.6085	2.302585	0	1	3.135494	C17	2017
1.94591	0.571429	14.73344	2.397895	0	2	3.178054	C17	2018
1.94591	0.571429	14.68806	2.484907	1	1	3.218876	C17	2019
2.197225	0.333333	16.03912	1.098612	0	1	0.693147	C33	2009
2.197225	0.333333	16.16641	2.484907	0	1	1.098612	C33	2010
2.197225	0.333333	14.95264	2.302585	0	1	1.386294	C33	2011
2.197225	0.333333	15.40532	1.94591	1	1	1.609438	C33	2012
2.197225	0.333333	15.39092	2.302585	1	1	1.791759	C33	2013
2.197225	0.333333	14.97641	2.484907	1	1	1.94591	C36	2014
1.609438	0.4	13.12396	1.791759	1	2	0.693147	C13	2009
1.609438	0.4	13.0002	1.94591	1	2	1.098612	C13	2010
1.609438	0.4	13.49379	2.302585	1	2	1.386294	C13	2011
1.609438	0.4	13.43234	2.079442	1	2	1.609438	C13	2012
1.94591	0.428571	13.65299	1.94591	1	2	1.791759	C13	2013
1.94591	0.428571	13.56705	1.609438	1	2	1.94591	C13	2014

1.94591	0.428571	13.74294	2.302585	1	2	2.079442	C13	2015
1.94591	0.428571	13.55415	1.791759	1	2	2.197225	C13	2016
1.94591	0.428571	13.85473	1.791759	1	2	2.302585	C13	2017
1.94591	0.428571	13.72963	1.609438	1	2	2.397895	C13	2018
1.94591	0.428571	13.64116	1.791759	1	2	2.484907	C13	2019
1.609438	0.4	13.47302	1.386294	1	1	2.772589	C32	2009
1.609438	0.4	13.95179	2.079442	1	1	2.833213	C32	2010
1.609438	0.4	14.21435	2.397895	1	1	2.890372	C32	2011
1.609438	0.4	14.48483	1.609438	1	1	2.944439	C32	2012
1.609438	0.4	14.65552	1.098612	1	1	2.995732	C32	2013
1.609438	0.4	15.09068	1.386294	1	1	3.044522	C32	2014
1.609438	0.4	15.00152	1.609438	1	1	3.091042	C32	2015
1.609438	0.4	15.02375	1.609438	1	1	3.135494	C32	2016
1.609438	0.4	15.00858	1.386294	1	1	3.178054	C32	2017
1.609438	0.4	14.78519	1.609438	1	1	3.218876	C32	2018
2.197225	0.333333	15.02148	1.609438	1	2	0.693147	F52	2009
2.197225	0.333333	14.87	2.302585	1	2	1.791759	F52	2013
1.609438	0.4	13.46055	2.079442	1	4	2.197225	C35	2009
1.609438	0.4	13.5505	2.197225	1	4	2.302585	C35	2010
1.609438	0.4	13.63531	2.197225	1	4	2.397895	C35	2011
1.609438	0.4	13.67012	2.079442	1	4	2.484907	C35	2012
1.609438	0.4	13.66805	1.791759	1	4	2.564949	C35	2013
1.609438	0.4	14.00778	2.639057	1	4	2.639057	C35	2014
1.609438	0.4	13.72515	1.791759	1	4	2.70805	C35	2015
1.609438	0.4	13.58358	1.94591	1	4	2.772589	C35	2016
1.609438	0.4	13.75576	2.079442	1	4	2.833213	C35	2017
1.609438	0.4	14.35626	2.079442	1	4	2.890372	C35	2018
1.609438	0.4	14.33526	2.302585	1	4	2.944439	C35	2019
2.397895	0.363636	14.38082	2.302585	1	3	1.791759	C41	2010
2.397895	0.363636	14.123	2.484907	0	3	1.94591	C41	2011
2.197225	0.333333	14.07787	2.197225	1	3	2.079442	C33	2012
2.197225	0.333333	14.06081	2.197225	1	3	2.197225	C33	2013
1.94591	0.428571	14.06002	1.94591	1	3	2.302585	C33	2014
1.94591	0.428571	13.68084	1.609438	1	3	2.397895	C33	2015
1.94591	0.428571	13.78505	1.791759	1	3	2.484907	C33	2016
1.94591	0.428571	13.81551	2.302585	1	3	2.564949	C33	2017
1.94591	0.428571	13.80344	1.791759	1	4	2.639057	C33	2018
1.94591	0.428571	14.0209	2.564949	1	4	2.70805	C33	2019
2.197225	0.333333	13.67751	1.791759	1	1	1.098612	C41	2009
2.197225	0.333333	14.34467	2.079442	1	1	1.386294	C41	2010
2.197225	0.333333	14.56735	2.302585	1	4	1.609438	C41	2011
2.197225	0.333333	14.68387	2.197225	1	1	1.791759	C41	2012
2.197225	0.333333	14.363	1.94591	1	1	1.94591	C41	2013
2.197225	0.333333	14.33293	2.302585	1	1	2.079442	C41	2014
2.197225	0.333333	14.27344	2.397895	1	4	2.197225	C41	2015
2.197225	0.333333	14.486	2.197225	1	4	2.302585	C41	2016
2.197225	0.333333	14.66382	1.609438	1	4	2.397895	C41	2017
2.197225	0.333333	14.7674	2.484907	1	4	2.484907	C41	2018
2.197225	0.333333	14.81035	2.079442	1	4	2.564949	C41	2019
2.197225	0.444444	13.77469	2.397895	0	2	1.098612	C34	2010
2.197225	0.555556	14.27924	2.197225	0	2	1.386294	C34	2011
2.197225	0.555556	14.40668	2.197225	1	2	1.609438	C34	2012
2.079442	0.375	14.49334	2.197225	1	2	1.791759	C34	2013
2.079442	0.375	14.48431	1.791759	1	4	1.94591	C34	2014
2.079442	0.375	14.48585	1.791759	1	4	2.079442	C34	2015
2.079442	0.375	14.32191	2.197225	1	4	2.197225	C34	2016
2.197225	0.333333	14.19607	2.639057	1	4	2.302585	C34	2017
2.197225	0.333333	14.32275	2.079442	1	4	2.397895	C34	2018

2.197225	0.333333	14.48431	2.079442	1	4	2.484907	C34	2019
2.079442	0.375	13.97763	2.079442	1	3	3.091042	C24	2011
2.079442	0.375	14.13032	1.609438	0	3	3.135494	C24	2012
2.079442	0.375	14.13032	2.197225	0	3	3.178054	C24	2013
2.079442	0.375	14.4307	1.609438	1	3	3.218876	C24	2014
2.079442	0.375	14.60101	1.791759	1	4	3.258097	C24	2015
2.079442	0.375	14.64122	1.791759	1	1	3.295837	C24	2016
2.079442	0.375	14.64122	1.609438	0	3	3.332205	C24	2017
2.079442	0.375	14.69298	1.94591	0	3	3.367296	C24	2018
2.197225	0.333333	12.18587	1.098612	1	3	0.693147	C33	2009
2.197225	0.333333	12.09514	2.197225	1	3	1.098612	C33	2010
2.197225	0.333333	12.3371	2.079442	1	3	1.386294	C33	2011
2.197225	0.333333	12.15373	1.94591	1	3	1.609438	C33	2012
2.197225	0.333333	12.27326	1.609438	1	3	1.791759	C33	2013
1.94591	0.428571	12.14153	1.791759	1	3	1.94591	C33	2014
1.94591	0.428571	12.23077	1.386294	1	3	2.079442	C33	2015
2.197225	0.333333	13.33747	2.302585	1	2	2.397895	C36	2010
2.197225	0.333333	13.16963	2.197225	1	3	0.693147	G54	2009
2.197225	0.333333	13.88699	2.833213	1	3	1.098612	G54	2010
2.197225	0.333333	13.82209	2.639057	1	3	1.386294	G54	2011
2.197225	0.333333	13.96969	2.484907	1	3	1.609438	G54	2012
2.079442	0.375	14.17066	2.397895	1	3	1.791759	G54	2013
2.079442	0.375	13.92131	2.079442	1	3	1.94591	G54	2014
2.079442	0.375	14.13578	2.772589	1	3	2.079442	G54	2015
2.197225	0.333333	13.98348	2.70805	1	3	2.197225	G54	2016
2.197225	0.333333	13.73235	2.079442	1	3	2.302585	G54	2017
2.079442	0.5	13.07191	2.079442	1	2	1.098612	C26	2010
2.302585	0.4	13.33844	1.791759	1	2	1.386294	C26	2011
2.302585	0.4	13.3675	1.94591	1	2	1.609438	C26	2012
2.302585	0.4	13.70591	1.791759	1	2	1.791759	C26	2013
1.94591	0.428571	13.73766	2.397895	1	2	1.94591	C26	2014
1.94591	0.428571	14.14034	2.197225	1	2	2.079442	C26	2015
1.791759	0.5	14.17144	1.609438	1	2	2.197225	C26	2016
1.791759	0.5	14.19634	1.791759	1	2	2.302585	C26	2017
1.791759	0.5	14.38592	1.791759	1	2	2.397895	C26	2018
1.791759	0.5	14.38241	1.609438	1	2	2.484907	C26	2019
1.94591	0.428571	12.42922	1.94591	1	3	2.772589	C36	2010
1.94591	0.428571	12.62938	2.197225	1	3	2.833213	C36	2011
1.94591	0.428571	12.77422	2.079442	1	3	2.890372	C36	2012
1.94591	0.428571	12.77987	2.302585	0	3	2.944439	C36	2013
1.94591	0.428571	12.92708	2.397895	1	3	2.995732	C36	2014
1.94591	0.428571	13.09807	2.197225	1	3	3.044522	C36	2015
1.94591	0.428571	13.09807	1.791759	1	3	3.091042	C36	2016
1.94591	0.428571	13.27078	2.197225	0	1	3.135494	C36	2017
1.94591	0.428571	13.4	2.079442	0	1	3.178054	C36	2018
1.94591	0.428571	13.4	2.079442	0	3	3.218876	C36	2019
2.197225	0.333333	13.39924	2.484907	1	2	2.197225	C27	2010
2.197225	0.333333	13.38657	1.94591	1	2	2.302585	C27	2011
2.197225	0.333333	13.49116	1.609438	1	2	2.397895	C27	2012
2.197225	0.333333	13.30468	1.94591	1	2	2.484907	C27	2013
2.197225	0.333333	13.52516	1.609438	1	2	2.564949	C27	2014
2.197225	0.333333	13.74014	1.609438	1	2	2.639057	C27	2015
2.197225	0.333333	13.8662	1.94591	1	2	2.70805	C27	2016
2.197225	0.333333	14.10324	2.197225	1	2	2.772589	C27	2017
2.197225	0.333333	13.99307	1.609438	1	2	2.833213	C27	2018
2.197225	0.333333	12.4837	1.791759	1	1	1.098612	C33	2010
2.197225	0.333333	13.19319	2.079442	1	1	1.386294	C33	2011
2.197225	0.333333	13.19375	1.791759	1	1	1.609438	C33	2012

2.197225	0.333333	13.33149	1.791759	1	1	1.791759	C33	2013
2.197225	0.333333	13.54737	1.94591	1	1	1.94591	C33	2014
2.197225	0.333333	13.55102	2.197225	1	2	2.079442	C33	2015
2.197225	0.333333	13.52543	2.564949	1	1	2.197225	C33	2016
2.197225	0.333333	13.52382	2.079442	1	1	2.302585	E48	2017
2.197225	0.444444	14.24729	2.639057	1	2	1.098612	E50	2010
2.197225	0.444444	14.24729	2.639057	1	2	1.386294	E50	2011
2.197225	0.444444	14.24729	2.639057	1	2	1.609438	E50	2012
2.197225	0.333333	13.99783	1.609438	1	2	1.791759	E50	2013
2.197225	0.333333	13.99783	2.564949	1	2	1.94591	E50	2014
2.397895	0.363636	14.31629	1.791759	1	2	2.302585	E50	2017
2.397895	0.363636	14.31629	1.609438	1	2	2.397895	E50	2018
2.197225	0.333333	14.65276	2.397895	1	2	2.484907	E50	2019
2.397895	0.363636	14.27924	1.94591	1	1	2.639057	C32	2014
2.397895	0.363636	14.27924	1.791759	1	1	2.70805	C32	2015
2.397895	0.363636	14.13105	1.94591	1	1	2.772589	C32	2016
2.197225	0.333333	14.27924	1.94591	1	1	2.833213	C32	2017
2.197225	0.333333	14.3694	2.079442	1	1	2.890372	C32	2018
2.197225	0.333333	14.44145	2.197225	1	1	2.944439	C32	2019
1.94591	0.428571	14.04503	2.397895	0	4	2.833213	C38	2010
2.079442	0.375	14.07147	1.94591	0	4	2.890372	C38	2011
1.94591	0.428571	14.06619	1.386294	1	4	2.944439	C38	2012
1.94591	0.428571	14.17946	1.94591	1	4	2.995732	C38	2013
1.94591	0.428571	14.29174	1.609438	1	4	3.044522	C38	2014
1.94591	0.428571	14.36836	1.609438	1	4	3.091042	C38	2015
1.94591	0.428571	14.43318	1.386294	1	4	3.135494	C38	2016
1.94591	0.428571	14.53442	1.098612	1	4	3.178054	C38	2017
1.94591	0.428571	14.50821	1.386294	1	4	3.218876	C38	2018
1.94591	0.428571	14.66335	1.791759	1	4	3.258097	I65	2019
2.397895	0.363636	13.31513	1.791759	1	2	2.197225	C29	2010
2.397895	0.363636	13.70458	2.197225	1	2	2.302585	C29	2011
2.397895	0.363636	13.69323	1.94591	1	2	2.397895	C29	2012
2.397895	0.363636	13.84682	1.791759	1	2	2.484907	C29	2013
2.397895	0.363636	13.88326	2.079442	1	2	2.564949	C29	2014
2.397895	0.363636	13.77009	2.197225	1	4	2.639057	C29	2015
2.397895	0.363636	13.86163	1.791759	1	4	2.70805	C29	2016
2.197225	0.333333	13.85646	2.079442	1	2	2.772589	C29	2017
2.197225	0.333333	13.93294	1.791759	1	4	2.833213	C29	2018
2.197225	0.333333	14.25357	1.386294	1	2	2.890372	C29	2019
2.197225	0.333333	13.8503	2.397895	1	1	1.098612	C33	2010
2.197225	0.333333	14.45462	2.397895	1	1	1.386294	C33	2011
2.197225	0.333333	14.51004	2.079442	1	1	1.609438	L72	2012
2.197225	0.333333	14.50966	2.302585	1	1	1.791759	C33	2013
2.197225	0.333333	14.51061	2.197225	1	1	1.94591	C33	2014
2.197225	0.333333	14.66391	2.397895	1	3	2.079442	C33	2015
2.197225	0.333333	14.95709	2.833213	1	3	2.197225	C33	2016
2.197225	0.333333	15.10485	2.890372	1	1	2.302585	C33	2017
2.197225	0.333333	15.12625	2.995732	1	3	2.397895	C39	2018
2.197225	0.333333	15.8	2.564949	1	3	2.484907	C39	2019
2.197225	0.333333	13.24635	2.079442	1	2	1.098612	C15	2010
2.197225	0.333333	13.75353	2.484907	1	2	1.386294	C15	2011
2.197225	0.333333	13.71384	2.197225	1	2	1.609438	C15	2012
2.079442	0.375	13.99081	2.302585	1	2	1.791759	C15	2013
2.197225	0.333333	13.96393	2.397895	1	2	1.94591	C15	2014
2.197225	0.333333	13.60145	2.079442	0	4	1.94591	C39	2010
2.197225	0.333333	13.72493	1.94591	0	4	2.079442	C39	2011
2.197225	0.333333	13.78773	2.079442	1	4	2.197225	C39	2012
2.079442	0.375	13.78226	2.484907	1	4	2.302585	C39	2013

2.079442	0.375	14.0058	2.079442	0	4	2.397895	C39	2014
2.197225	0.333333	13.89247	2.302585	1	4	2.484907	C39	2015
2.197225	0.333333	13.94654	2.639057	1	4	2.564949	C39	2016
2.197225	0.333333	14.13273	2.197225	1	4	2.639057	C39	2017
2.197225	0.333333	14.15924	2.484907	1	4	2.70805	C39	2018
2.197225	0.333333	14.13324	2.302585	1	4	2.772589	C39	2019
2.197225	0.333333	13.91064	2.302585	1	4	1.386294	C39	2010
2.197225	0.333333	14.02147	2.302585	1	4	1.609438	C39	2011
2.397895	0.363636	14.07847	2.639057	1	4	1.791759	C39	2012
2.302585	0.4	13.78464	2.397895	1	4	1.94591	C39	2013
2.302585	0.4	13.79234	2.70805	1	4	2.079442	C39	2014
2.197225	0.333333	13.75204	2.484907	1	4	2.197225	C39	2015
2.197225	0.333333	13.88876	1.94591	1	4	2.302585	C39	2016
2.079442	0.375	14.07787	1.94591	1	3	1.098612	C30	2010
2.197225	0.333333	14.07787	2.70805	1	3	1.386294	C30	2011
2.197225	0.333333	14.03865	2.302585	1	3	1.609438	C30	2012
2.197225	0.333333	14.07787	2.639057	1	3	1.791759	C30	2013
2.397895	0.363636	14.28551	2.079442	1	3	1.94591	C30	2014
2.397895	0.363636	13.77469	2.484907	1	3	2.079442	C30	2015
2.397895	0.363636	14.28551	2.079442	1	4	2.197225	C30	2016
2.397895	0.363636	14.28551	1.609438	1	3	2.302585	C30	2017
2.197225	0.333333	14.25377	2.302585	1	3	2.397895	C30	2018
2.197225	0.333333	14.25377	2.079442	1	4	2.484907	C30	2019
2.197225	0.333333	14.94164	2.484907	1	1	2.079442	C17	2010
2.197225	0.333333	15.03149	2.079442	1	1	2.197225	C17	2011
2.197225	0.333333	14.78814	1.94591	1	1	2.302585	C17	2012
2.197225	0.333333	14.96953	1.098612	0	1	2.397895	C17	2013
2.197225	0.333333	14.5353	1.94591	0	1	2.484907	C17	2014
2.197225	0.333333	14.49253	1.609438	0	1	2.564949	C17	2015
2.197225	0.333333	14.79387	1.791759	0	1	2.639057	C17	2016
2.197225	0.333333	14.96126	1.386294	1	1	2.70805	C17	2017
2.197225	0.333333	15.01122	1.609438	1	1	2.772589	C17	2018
2.197225	0.333333	15.39986	1.609438	1	3	2.833213	C17	2019
2.197225	0.333333	13.31859	1.94591	1	2	2.079442	C29	2010
2.079442	0.375	13.47752	1.94591	1	2	2.197225	C29	2011
2.197225	0.333333	13.75364	1.791759	1	2	2.302585	C29	2012
2.197225	0.333333	13.95527	1.94591	1	2	2.397895	C29	2013
2.197225	0.333333	14.04662	1.609438	1	2	2.484907	C29	2014
2.197225	0.333333	14.04662	1.386294	1	2	2.564949	C29	2015
2.197225	0.333333	14.04662	1.94591	1	2	2.639057	C29	2016
2.197225	0.333333	14.04662	1.609438	1	2	2.70805	C29	2017
2.197225	0.333333	14.00613	1.609438	1	2	2.772589	C29	2018
1.94591	0.428571	14.00613	1.94591	1	2	2.833213	C29	2019
1.94591	0.428571	13.37855	2.397895	1	1	2.833213	C33	2010
1.94591	0.428571	13.88382	2.397895	1	1	2.890372	C33	2011
1.94591	0.428571	13.87745	2.302585	1	1	2.944439	C33	2012
1.94591	0.428571	14.00501	1.94591	1	1	2.995732	C33	2013
1.94591	0.428571	14.02358	2.079442	1	1	3.044522	C33	2014
1.94591	0.428571	14.09109	2.302585	1	1	3.091042	C33	2015
1.94591	0.428571	14.07841	2.079442	1	1	3.135494	C33	2016
1.94591	0.428571	14.17897	2.302585	1	1	3.178054	C33	2017
1.94591	0.428571	14.07964	2.079442	1	1	3.218876	C33	2018
1.94591	0.428571	14.11887	1.94591	1	1	3.258097	C33	2019
2.197225	0.333333	12.96501	2.302585	1	4	1.791759	C26	2010
2.197225	0.333333	14.39414	2.70805	1	4	1.94591	C26	2011
2.079442	0.375	13.30201	2.079442	1	4	2.079442	C26	2012
2.197225	0.333333	13.3878	1.791759	1	4	2.197225	C26	2013
2.197225	0.333333	13.12755	2.484907	1	3	2.302585	C26	2014

2.079442	0.375	13.87764	2.70805	1	3	2.397895	C26	2015
2.197225	0.333333	15.13783	2.079442	1	4	2.484907	C26	2016
2.197225	0.333333	14.86346	2.772589	1	3	2.564949	C26	2017
2.197225	0.333333	14.3257	2.564949	1	4	2.639057	C26	2018
2.197225	0.333333	14.20789	2.302585	1	4	2.70805	C26	2019
1.94591	0.428571	14.00074	2.197225	1	3	2.197225	C39	2013
1.94591	0.428571	14.24892	2.079442	1	3	2.302585	C39	2014
1.94591	0.428571	14.12652	2.197225	1	3	2.397895	C39	2015
1.94591	0.428571	14.40091	2.079442	1	3	2.484907	C39	2016
1.94591	0.428571	14.46601	2.197225	1	3	2.564949	C39	2017
1.94591	0.428571	14.68379	2.197225	1	3	2.639057	C39	2018
1.94591	0.428571	14.54672	1.94591	1	3	2.70805	C39	2019
2.197225	0.333333	14.60397	2.079442	1	4	2.079442	C27	2010
2.197225	0.333333	14.60397	1.94591	1	4	2.197225	C27	2011
2.197225	0.333333	14.60397	2.564949	1	4	2.302585	C27	2012
2.197225	0.333333	14.77102	2.302585	1	4	2.397895	C27	2013
2.197225	0.333333	14.77102	2.397895	1	4	2.484907	C27	2014
2.197225	0.333333	14.90854	2.397895	1	4	2.564949	C27	2015
2.197225	0.333333	15.29712	2.639057	1	4	2.639057	C27	2016
2.197225	0.333333	15.29712	1.94591	1	4	2.70805	C27	2017
2.079442	0.375	15.29712	2.397895	1	4	2.772589	C27	2018
2.197225	0.333333	16.19506	2.564949	1	4	2.833213	C27	2019
2.197225	0.333333	13.45683	2.197225	1	4	1.098612	C27	2010
2.197225	0.333333	13.9817	1.94591	1	4	1.386294	C27	2011
2.197225	0.333333	13.9817	2.079442	1	4	1.609438	C27	2012
2.197225	0.333333	13.9817	1.386294	1	4	1.791759	C27	2013
2.197225	0.333333	14.62199	2.197225	1	4	1.94591	C27	2014
2.197225	0.333333	14.62199	2.197225	1	4	2.079442	C27	2015
2.197225	0.333333	14.62199	2.564949	1	4	2.197225	C27	2016
2.197225	0.333333	14.69928	2.484907	1	4	2.302585	C27	2017
2.197225	0.333333	14.83313	2.484907	1	4	2.397895	C27	2018
2.197225	0.333333	14.7926	2.397895	1	4	2.484907	C27	2019
2.197225	0.333333	13.29934	1.609438	1	4	1.098612	C35	2010
2.197225	0.333333	13.39392	2.079442	1	4	1.386294	C35	2011
2.197225	0.333333	13.33056	1.94591	1	4	1.609438	C35	2012
2.197225	0.333333	13.33343	1.94591	1	4	1.791759	C35	2013
2.197225	0.333333	13.40844	1.386294	1	4	1.94591	C35	2014
2.197225	0.333333	13.40799	2.079442	1	4	2.079442	C35	2015
2.197225	0.333333	13.00493	2.302585	1	4	2.197225	C35	2016
2.197225	0.333333	14.80002	1.791759	1	4	2.302585	C35	2017
2.197225	0.333333	14.89613	1.791759	1	4	2.397895	C27	2018
2.197225	0.333333	13.93773	2.397895	0	2	1.791759	C26	2010
2.197225	0.333333	14.4033	2.397895	0	2	1.94591	C26	2011
2.197225	0.333333	14.4033	2.484907	1	2	2.079442	C26	2012
2.197225	0.333333	14.63088	2.079442	1	2	2.197225	C26	2013
2.079442	0.375	14.63088	2.079442	1	2	2.302585	C26	2014
2.079442	0.375	14.42528	1.791759	0	2	2.397895	C26	2015
2.197225	0.333333	14.42528	1.791759	0	2	2.484907	C26	2016
2.197225	0.333333	14.58098	1.791759	1	2	2.564949	C26	2017
2.197225	0.333333	14.60397	1.609438	1	2	2.639057	C26	2018
2.197225	0.333333	14.69098	1.609438	1	2	2.70805	C26	2019
2.197225	0.333333	13.7726	1.609438	1	3	0.693147	F51	2010
2.197225	0.333333	13.78742	2.564949	1	3	1.098612	F51	2011
2.197225	0.333333	13.79326	2.484907	1	3	1.386294	F51	2012
2.197225	0.333333	13.79326	2.397895	1	3	1.609438	F51	2013
2.079442	0.375	14.4033	2.302585	1	3	1.791759	F51	2014
2.197225	0.333333	15.01367	2.564949	1	3	1.94591	F51	2015
2.197225	0.333333	14.99834	2.397895	1	3	2.079442	F51	2016

2.197225	0.333333	14.97785	2.302585	1	3	2.197225	F51	2017
2.197225	0.333333	15.21952	2.70805	1	3	2.302585	F51	2018
2.197225	0.333333	15.2178	2.079442	1	3	2.397895	F51	2019
2.079442	0.375	13.86058	1.609438	1	1	0.693147	C26	2010
2.197225	0.333333	13.90818	2.302585	1	1	1.098612	C26	2011
2.197225	0.333333	14.06814	1.609438	1	1	1.386294	C26	2012
2.079442	0.375	14.08164	1.386294	1	1	1.609438	C26	2013
2.197225	0.333333	14.41775	1.791759	1	1	1.791759	C26	2014
2.302585	0.4	13.95814	1.791759	1	1	1.94591	C26	2015
2.079442	0.375	13.92795	2.079442	1	1	2.079442	C26	2016
1.94591	0.428571	13.52583	2.079442	1	1	2.197225	C26	2017
1.94591	0.428571	14.64977	1.94591	1	1	2.302585	C26	2018
1.94591	0.428571	14.74901	1.386294	1	1	2.397895	C26	2019
2.197225	0.333333	13.32121	2.302585	1	1	2.079442	C33	2010
2.197225	0.333333	13.5008	2.70805	1	1	2.197225	C33	2011
2.197225	0.333333	13.81551	2.197225	1	1	2.302585	C33	2012
2.079442	0.375	13.78505	2.397895	1	3	2.397895	C33	2013
2.197225	0.333333	13.97251	2.079442	1	3	2.484907	C33	2014
2.197225	0.333333	13.97251	1.791759	1	3	2.564949	C33	2015
2.197225	0.333333	14.01436	2.302585	1	3	2.639057	C33	2016
2.197225	0.333333	14.07325	2.197225	1	3	0.693147	C33	2010
2.197225	0.333333	14.37752	2.397895	1	3	1.098612	C33	2011
2.197225	0.333333	14.44268	2.302585	1	3	1.386294	C33	2012
2.197225	0.333333	14.37215	2.079442	1	3	1.609438	C33	2013
2.197225	0.333333	14.39577	2.564949	1	3	1.791759	C33	2014
2.197225	0.333333	14.43679	1.791759	1	3	1.94591	C33	2015
2.197225	0.333333	14.49197	2.302585	1	3	2.079442	C33	2016
2.197225	0.333333	14.71384	2.197225	1	3	2.197225	C33	2017
2.197225	0.333333	14.7328	2.564949	1	3	2.302585	C33	2018
2.197225	0.333333	14.73268	2.397895	1	3	2.397895	C33	2019
1.94591	0.428571	13.54107	2.079442	1	3	0.693147	A04	2010
1.94591	0.428571	14.09246	2.639057	1	3	1.098612	A04	2011
1.94591	0.428571	14.37393	2.197225	1	3	1.386294	A04	2012
1.94591	0.428571	14.50168	1.94591	1	3	1.609438	A04	2013
1.94591	0.428571	15.46853	2.639057	1	3	1.791759	A04	2014
1.94591	0.428571	15.724	1.94591	1	3	1.94591	A04	2015
1.94591	0.428571	14.99908	2.302585	1	3	2.079442	A04	2016
1.791759	0.5	13.12236	2.890372	1	3	2.197225	A04	2017
2.197225	0.333333	13.81321	2.397895	1	3	2.197225	C26	2010
1.94591	0.428571	14.06767	2.772589	1	3	2.302585	C26	2011
1.94591	0.428571	14.17602	2.484907	1	3	2.397895	C29	2012
1.94591	0.428571	15.1566	2.197225	1	3	2.484907	C29	2013
1.94591	0.428571	15.36175	2.397895	1	3	2.564949	C29	2014
1.94591	0.428571	15.38022	2.484907	1	3	2.639057	C29	2015
1.94591	0.428571	15.65937	2.833213	1	3	2.70805	C29	2016
1.94591	0.428571	15.8064	2.079442	1	3	2.772589	C29	2017
2.197225	0.333333	13.13033	1.386294	1	3	1.386294	C38	2010
2.197225	0.333333	13.13033	1.791759	1	3	1.609438	C38	2011
1.94591	0.428571	12.97154	1.94591	1	3	1.791759	C38	2012
1.94591	0.428571	12.97154	2.197225	1	3	1.94591	C38	2013
1.94591	0.428571	12.97154	2.079442	1	3	2.079442	C38	2014
1.791759	0.5	13.12236	2.484907	1	3	2.197225	C38	2015
2.197225	0.333333	13.25339	2.564949	1	3	2.302585	C38	2016
2.197225	0.333333	13.5008	1.791759	1	3	2.397895	C38	2017
2.197225	0.333333	13.61339	1.791759	1	3	2.484907	C38	2018
2.197225	0.333333	14.0209	2.197225	1	3	2.564949	C38	2019
2.197225	0.333333	13.76884	2.197225	1	2	1.098612	C32	2010
2.197225	0.333333	13.92176	2.197225	1	2	1.386294	C32	2011

2.197225	0.333333	13.83732	2.302585	1	2	1.609438	C32	2012
2.197225	0.333333	13.90352	2.564949	1	2	1.791759	C32	2013
2.197225	0.333333	13.92786	2.079442	1	2	1.94591	C32	2014
2.197225	0.333333	13.9489	2.079442	1	2	2.079442	C32	2015
2.079442	0.375	14.1213	2.079442	1	2	2.197225	C32	2016
2.302585	0.4	14.49116	2.772589	1	2	2.397895	C32	2018
2.197225	0.333333	15.06742	2.302585	1	2	2.484907	C32	2019
2.197225	0.333333	13.85406	2.302585	1	3	1.94591	C39	2010
2.197225	0.333333	13.9622	2.302585	1	3	2.079442	C39	2011
2.197225	0.333333	14.04185	2.302585	1	3	2.197225	C39	2012
2.197225	0.333333	14.79474	2.302585	1	3	2.302585	C39	2013
2.197225	0.333333	14.74539	2.302585	1	3	2.639057	C39	2017
2.197225	0.333333	14.7427	2.302585	1	3	2.70805	C39	2018
2.197225	0.333333	15.17461	2.302585	1	3	2.772589	C39	2019
2.197225	0.333333	14.35446	2.197225	1	1	2.833213	C29	2010
2.197225	0.333333	14.38559	2.302585	1	1	2.890372	C29	2011
2.079442	0.375	14.16905	1.609438	1	1	2.944439	C29	2012
2.197225	0.333333	14.26109	1.386294	1	1	2.995732	C29	2013
1.94591	0.428571	12.95984	2.079442	1	3	1.098612	C26	2010
2.197225	0.333333	13.017	2.197225	1	3	1.386294	C26	2011
2.197225	0.333333	13.36922	2.197225	1	3	1.609438	C26	2012
2.197225	0.333333	13.49144	2.197225	1	3	1.791759	C26	2013
1.94591	0.428571	13.85184	2.639057	1	3	1.94591	C26	2014
1.94591	0.428571	13.93773	2.484907	1	3	2.079442	C26	2015
1.94591	0.428571	14.48129	2.197225	1	3	2.197225	C32	2016
1.94591	0.428571	14.99724	2.564949	1	3	2.302585	C32	2017
1.94591	0.428571	15.16085	2.639057	1	3	2.397895	C32	2018
1.94591	0.428571	15.3259	2.484907	1	3	2.484907	C32	2019
1.94591	0.428571	13.30868	2.197225	1	2	2.197225	C38	2017
2.197225	0.333333	14.03865	1.609438	1	4	1.609438	C34	2010
2.197225	0.333333	14.06401	1.94591	1	4	1.791759	C34	2011
2.197225	0.333333	14.04742	1.94591	1	4	1.94591	C34	2012
2.197225	0.333333	14.05524	1.609438	1	4	2.079442	C34	2013
2.079442	0.375	14.34243	1.94591	1	2	2.197225	C34	2014
2.197225	0.333333	14.37507	1.94591	1	2	2.302585	C34	2015
2.079442	0.375	14.4149	2.197225	1	4	2.397895	C34	2016
2.197225	0.333333	14.6886	2.484907	1	4	2.484907	C34	2017
2.197225	0.333333	14.70636	2.197225	1	2	2.564949	C34	2018
2.197225	0.333333	14.83976	2.079442	1	4	2.639057	C34	2019
2.197225	0.333333	14.14926	1.94591	1	4	1.791759	C39	2010
2.197225	0.333333	14.12307	1.94591	1	4	1.94591	C39	2011
2.197225	0.333333	14.35574	1.94591	1	4	2.079442	C38	2012
2.197225	0.333333	14.7318	1.791759	1	4	2.197225	C38	2013
2.197225	0.333333	14.7318	1.94591	1	4	2.302585	C38	2014
2.197225	0.333333	13.27834	1.791759	1	2	0.693147	D44	2010
2.197225	0.333333	13.35284	2.302585	1	2	1.098612	D44	2011
2.197225	0.333333	13.99364	2.397895	1	2	1.386294	D44	2012
2.197225	0.333333	13.59934	2.484907	1	2	1.609438	D44	2013
2.197225	0.333333	13.775	2.079442	1	2	1.791759	D44	2014
2.079442	0.375	13.69447	2.079442	1	2	1.94591	D44	2015
2.197225	0.333333	13.65897	1.94591	1	2	2.079442	D44	2016
2.197225	0.333333	14.123	2.302585	1	2	2.197225	D44	2017
2.197225	0.333333	14.13759	2.079442	1	2	2.302585	D44	2018
2.197225	0.333333	14.08554	2.079442	1	2	2.397895	D44	2019
2.197225	0.333333	14.25377	2.079442	1	3	0.693147	E50	2010
2.197225	0.333333	14.49861	2.639057	1	3	1.098612	E50	2011
2.197225	0.333333	14.57594	2.302585	1	4	1.386294	E50	2012
2.197225	0.333333	14.72236	2.197225	1	4	1.609438	E50	2013

2.197225	0.333333	14.75043	2.397895	1	4	1.791759	E50	2014
2.197225	0.333333	14.85678	2.772589	1	4	1.94591	E50	2015
2.197225	0.333333	14.92737	2.70805	1	4	2.079442	E50	2016
2.197225	0.333333	14.94701	2.302585	1	3	2.197225	E50	2017
2.197225	0.333333	14.83299	2.302585	1	3	2.302585	E50	2018
2.079442	0.375	14.74767	2.639057	1	4	2.397895	E50	2019
2.197225	0.333333	13.3858	2.302585	1	5	2.639057	C39	2016
2.302585	0.4	13.53699	2.302585	1	5	2.70805	C39	2017
2.197225	0.333333	13.8103	2.079442	1	5	2.772589	C39	2018
2.197225	0.333333	13.92068	2.302585	1	5	2.833213	C39	2019
2.197225	0.333333	13.2056	2.079442	1	1	1.098612	C36	2010
2.197225	0.333333	13.18063	2.302585	1	1	1.386294	C36	2011
2.197225	0.333333	13.21585	2.302585	1	1	1.609438	C36	2012
1.94591	0.428571	13.22726	2.302585	1	1	1.791759	C36	2013
1.94591	0.428571	13.35173	2.639057	1	1	1.94591	C36	2014
1.94591	0.428571	13.58181	2.302585	1	1	2.079442	C36	2015
1.94591	0.428571	14.05814	2.70805	1	1	2.197225	C36	2016
2.197225	0.333333	14.24241	2.833213	1	1	2.302585	C36	2017
2.197225	0.333333	14.49567	2.484907	1	1	2.397895	C36	2018
2.197225	0.333333	14.4487	2.302585	1	1	2.484907	C36	2019
2.397895	0.454545	14.50118	1.609438	1	2	1.098612	C21	2010
2.397895	0.454545	14.55588	1.94591	1	2	1.386294	C21	2011
2.302585	0.5	14.54515	2.484907	1	2	1.609438	C21	2012
2.197225	0.333333	14.50595	2.079442	1	2	1.791759	C21	2013
2.197225	0.333333	14.55066	1.94591	1	2	1.94591	C21	2014
2.197225	0.333333	14.56942	2.397895	1	2	2.079442	C21	2015
2.197225	0.333333	14.54749	2.302585	1	2	2.197225	C21	2016
2.197225	0.333333	14.53017	1.791759	1	2	2.302585	C21	2017
2.197225	0.333333	14.55157	2.079442	1	2	2.397895	C21	2018
2.197225	0.333333	15.0453	2.397895	1	2	2.484907	C21	2019
2.197225	0.333333	14.13039	1.791759	1	2	2.197225	C35	2010
2.197225	0.333333	14.16321	2.197225	1	2	2.302585	C35	2011
2.197225	0.333333	14.14637	2.079442	1	2	2.397895	C35	2012
2.197225	0.333333	13.9666	1.609438	1	2	2.484907	C35	2013
2.197225	0.333333	14.11421	1.386294	1	2	2.564949	C35	2014
2.079442	0.375	14.12871	1.609438	1	2	2.639057	C35	2015
2.197225	0.333333	14.12893	1.94591	1	2	2.70805	C35	2016
2.197225	0.333333	14.12945	1.791759	1	2	2.833213	C35	2018
2.197225	0.333333	14.30685	1.609438	1	2	2.890372	C35	2019
1.94591	0.428571	13.71148	1.609438	0	2	0.693147	G59	2010
1.94591	0.428571	13.89561	2.302585	1	2	1.098612	G59	2011
1.94591	0.428571	13.78927	1.791759	1	2	1.386294	G59	2012
1.94591	0.428571	14.07834	2.302585	1	2	1.609438	G59	2013
1.94591	0.428571	13.99742	2.397895	1	2	1.791759	G59	2014
1.94591	0.428571	14.01157	1.791759	1	2	1.94591	G59	2015
1.94591	0.428571	14.06385	2.397895	1	2	2.079442	G59	2016
1.94591	0.428571	14.09828	2.302585	1	2	2.197225	G59	2017
1.94591	0.428571	14.22204	1.94591	1	2	2.302585	G59	2018
1.94591	0.428571	14.26601	1.791759	1	2	2.397895	G59	2019
2.197225	0.333333	14.21837	2.079442	1	2	1.098612	C28	2010
2.197225	0.333333	14.39717	2.197225	1	2	1.386294	C28	2011
2.197225	0.333333	14.62666	2.079442	1	2	1.609438	C28	2012
2.079442	0.375	14.66	1.609438	1	2	1.791759	C28	2013
2.079442	0.375	15.0149	2.079442	1	2	1.94591	C28	2014
2.197225	0.333333	15.1267	2.197225	1	2	2.079442	C28	2015
2.197225	0.333333	15.15892	1.94591	1	2	2.197225	C28	2016
2.197225	0.333333	14.95225	2.079442	1	2	2.302585	C28	2017
2.197225	0.333333	14.97161	1.94591	1	2	2.397895	C28	2018

2.197225	0.333333	15.21331	2.397895	1	2	2.484907	C28	2019
2.197225	0.333333	12.23271	2.397895	1	1	0.693147	C19	2011
2.197225	0.333333	12.23854	2.397895	1	1	1.098612	C19	2012
2.197225	0.333333	12.67357	2.302585	1	1	1.386294	C19	2013
2.197225	0.333333	12.67357	1.609438	1	1	1.609438	C19	2014
2.197225	0.333333	12.67357	2.197225	1	1	1.791759	C19	2015
2.197225	0.333333	12.67357	2.197225	1	1	1.94591	C19	2016
1.94591	0.428571	12.67357	2.079442	1	1	2.079442	C19	2017
1.94591	0.428571	13.24917	2.302585	1	1	2.197225	C19	2018
1.94591	0.428571	13.24917	2.079442	1	1	2.302585	C19	2019
2.197225	0.333333	12.38839	1.791759	1	2	2.079442	C14	2010
2.197225	0.333333	13.10216	1.94591	1	2	2.197225	C14	2011
2.197225	0.333333	13.3253	2.197225	1	2	2.302585	C14	2012
2.197225	0.333333	13.3253	1.609438	1	2	2.397895	C14	2013
2.197225	0.333333	13.43352	2.079442	1	2	2.484907	C14	2014
2.197225	0.333333	13.89893	2.302585	1	2	2.564949	C14	2015
2.197225	0.333333	14.04464	1.609438	1	2	2.639057	C14	2016
2.197225	0.333333	14.18154	1.791759	1	2	2.70805	C14	2017
2.197225	0.333333	14.28545	1.94591	1	2	2.772589	C14	2018
2.197225	0.333333	14.28689	1.609438	1	2	2.833213	C14	2019
2.197225	0.333333	14.66061	0.693147	1	2	2.397895	C26	2010
2.079442	0.375	14.65228	2.397895	1	2	2.484907	C26	2011
2.197225	0.333333	14.045	2.079442	1	2	2.564949	C26	2012
2.197225	0.333333	14.19052	2.079442	1	2	2.639057	C26	2013
2.197225	0.333333	14.21435	1.94591	1	2	2.70805	C26	2014
2.197225	0.333333	14.1921	2.397895	1	2	2.772589	C26	2015
2.197225	0.333333	14.22231	2.197225	1	2	2.833213	C26	2016
2.197225	0.333333	14.21496	2.079442	1	2	2.890372	C26	2017
2.197225	0.333333	14.71868	1.94591	1	2	2.995732	C26	2019
2.197225	0.333333	14.15251	1.386294	0	3	2.197225	C26	2010
2.197225	0.333333	14.47201	2.197225	1	3	2.302585	C26	2011
2.197225	0.333333	14.37231	2.397895	1	3	2.397895	C26	2012
2.197225	0.333333	14.5132	2.197225	1	3	2.484907	C26	2013
2.197225	0.333333	14.66511	2.397895	1	3	2.564949	C26	2014
2.197225	0.333333	14.30151	2.564949	1	3	2.639057	C26	2015
2.197225	0.333333	14.74752	2.639057	1	3	2.70805	C26	2016
2.197225	0.333333	14.96792	2.484907	1	3	2.772589	C26	2017
2.197225	0.333333	14.91705	2.302585	1	3	2.833213	C26	2018
2.197225	0.333333	14.61581	2.197225	1	3	2.890372	C26	2019
2.197225	0.333333	13.26195	1.609438	1	3	1.098612	C38	2010
2.197225	0.333333	13.3284	2.079442	1	3	1.386294	C38	2011
2.197225	0.333333	12.86359	2.484907	1	3	1.609438	C38	2012
2.197225	0.333333	13.4	2.484907	1	3	1.791759	C38	2013
2.197225	0.333333	13.33747	2.197225	1	3	1.94591	C38	2014
2.079442	0.375	13.46453	1.94591	1	3	2.079442	C38	2015
2.197225	0.333333	13.55415	2.639057	1	3	2.197225	C38	2016
2.197225	0.333333	13.54107	2.302585	1	3	2.302585	C38	2017
2.197225	0.333333	13.61706	1.791759	1	3	2.397895	C38	2018
2.197225	0.333333	14.17319	2.197225	1	3	2.484907	C38	2019
2.197225	0.333333	13.27937	1.791759	1	3	0.693147	C32	2010
2.197225	0.333333	13.07464	2.302585	1	3	1.098612	C32	2011
2.197225	0.333333	14.11835	2.079442	1	3	1.386294	C32	2012
2.079442	0.375	14.02893	2.302585	1	3	1.609438	C32	2013
1.791759	0.5	13.94382	1.609438	1	3	1.791759	C32	2014
2.197225	0.333333	13.98179	1.94591	1	3	1.94591	C32	2015
2.197225	0.333333	14.1381	2.079442	1	3	2.079442	C32	2016
2.197225	0.333333	14.62577	2.397895	1	3	2.197225	C32	2017
2.197225	0.333333	13.25374	2.302585	1	4	1.94591	C39	2010

2.197225	0.333333	13.49806	2.944439	1	4	2.079442	C39	2011
2.197225	0.333333	13.87802	1.791759	1	1	2.302585	C38	2010
2.197225	0.333333	13.9749	2.302585	1	1	2.397895	C38	2011
2.197225	0.333333	14.14527	1.94591	1	1	2.484907	C38	2012
2.197225	0.333333	14.31937	1.791759	1	1	2.564949	C38	2013
2.079442	0.375	14.34319	1.94591	1	1	2.639057	C38	2014
2.197225	0.333333	14.40001	1.94591	1	1	2.70805	C38	2015
2.197225	0.333333	14.66609	1.94591	1	1	2.772589	C38	2016
2.197225	0.333333	14.70886	1.94591	1	1	2.833213	C38	2017
2.197225	0.333333	14.59088	1.791759	1	1	2.890372	C38	2018
2.197225	0.333333	14.68435	1.609438	1	1	2.944439	C38	2019
2.397895	0.363636	14.22098	2.564949	1	3	2.397895	C22	2010
2.397895	0.363636	13.95779	2.302585	1	3	2.484907	C22	2011
2.397895	0.363636	15.44348	2.302585	1	3	2.564949	C22	2012
2.397895	0.363636	14.08912	2.397895	1	3	2.639057	C22	2013
2.197225	0.444444	14.02114	2.197225	1	3	2.70805	C22	2014
2.197225	0.333333	14.4308	2.197225	1	3	2.772589	C22	2015
2.197225	0.333333	14.61778	2.197225	1	3	2.833213	C22	2016
2.197225	0.333333	15.16101	2.197225	1	3	2.890372	C22	2017
2.197225	0.333333	15.60602	2.302585	1	3	2.944439	C22	2018
2.197225	0.333333	16.21169	2.302585	1	3	2.995732	C22	2019
2.197225	0.333333	14.15277	1.791759	1	2	2.302585	C17	2010
2.197225	0.333333	14.02269	2.484907	1	2	2.397895	C17	2011
2.197225	0.333333	14.15198	2.197225	1	2	2.484907	C17	2012
2.197225	0.333333	14.26257	2.302585	1	2	2.564949	C17	2013
2.197225	0.333333	14.32185	2.302585	1	2	2.639057	C17	2014
2.197225	0.333333	14.34614	2.70805	1	2	2.70805	C17	2015
2.197225	0.333333	14.39342	2.302585	1	2	2.772589	C17	2016
2.197225	0.333333	14.53452	1.94591	1	2	2.833213	C17	2017
2.197225	0.333333	14.53715	1.94591	1	2	2.890372	C17	2018
2.197225	0.333333	14.48211	1.791759	1	2	2.944439	C17	2019
1.609438	0.6	13.42395	2.484907	1	3	2.639057	C19	2014
1.94591	0.428571	12.91608	1.098612	1	4	2.397895	C34	2010
1.94591	0.428571	13.73928	2.197225	1	4	2.484907	C34	2011
1.94591	0.428571	13.43952	2.302585	1	4	2.564949	C34	2012
1.94591	0.428571	13.47794	2.302585	1	4	2.639057	C34	2013
1.94591	0.428571	13.68597	1.94591	1	4	2.70805	C34	2014
1.94591	0.428571	13.64068	2.302585	1	4	2.772589	C34	2015
1.94591	0.428571	13.59199	2.302585	1	4	2.833213	C34	2016
1.94591	0.428571	13.7601	1.791759	1	4	2.890372	C34	2017
1.94591	0.428571	13.87548	2.484907	1	4	2.944439	C34	2018
1.94591	0.428571	14.55754	2.197225	1	4	2.995732	C34	2019
2.079442	0.375	13.95527	1.791759	1	4	2.197225	C22	2010
2.197225	0.333333	14.01025	2.079442	1	4	2.302585	C22	2011
2.197225	0.333333	13.84897	1.791759	1	4	2.397895	C22	2012
2.197225	0.333333	14.14121	2.197225	1	4	2.484907	C22	2013
1.94591	0.428571	14.19989	2.302585	1	4	2.564949	C22	2014
1.94591	0.428571	14.17744	1.609438	1	4	2.639057	C22	2015
1.94591	0.428571	14.1912	1.94591	1	4	2.70805	C22	2016
1.94591	0.428571	14.4307	1.94591	1	4	2.772589	C22	2017
1.94591	0.428571	14.12769	1.386294	1	4	2.833213	C22	2018
1.94591	0.428571	14.30917	1.609438	1	4	2.890372	C22	2019
1.94591	0.428571	13.90535	1.609438	1	3	2.197225	C29	2010
1.94591	0.428571	13.91363	2.197225	1	3	2.302585	C29	2011
1.94591	0.428571	13.93767	2.197225	1	3	2.397895	C29	2012
1.94591	0.428571	13.94109	1.791759	1	3	2.484907	C29	2013
1.94591	0.428571	13.93799	1.609438	1	3	2.564949	C29	2014
1.94591	0.428571	13.69447	2.564949	1	3	2.639057	C29	2015

1.94591	0.428571	13.8112	1.791759	1	3	2.70805	C29	2016
1.94591	0.428571	14.18804	2.079442	1	3	2.772589	C29	2017
1.94591	0.428571	14.63039	2.197225	1	3	2.833213	C29	2018
1.94591	0.428571	15.02286	1.791759	1	3	2.890372	C29	2019
2.197225	0.333333	12.46844	1.791759	1	2	2.397895	C35	2010
2.197225	0.333333	14.45736	2.484907	1	2	2.484907	C35	2011
2.197225	0.333333	13.73213	2.70805	1	2	2.564949	C35	2012
2.197225	0.333333	12.99453	2.079442	1	2	2.639057	C35	2013
2.079442	0.375	13.0444	1.791759	1	2	2.70805	C35	2014
2.079442	0.375	13.06049	2.197225	1	2	2.772589	C35	2015
2.197225	0.333333	13.0919	2.639057	1	2	2.833213	C35	2016
2.197225	0.333333	13.31298	2.197225	1	2	2.890372	C35	2017
2.197225	0.333333	13.94487	1.94591	1	2	2.944439	C35	2018
2.197225	0.333333	14.05484	1.94591	1	2	2.995732	C35	2019
2.197225	0.333333	14.48231	1.386294	1	4	2.70805	C38	2010
2.197225	0.333333	14.88915	2.397895	1	4	2.772589	C38	2011
2.197225	0.333333	14.99417	2.079442	1	4	2.833213	C38	2012
2.079442	0.375	15.11516	2.397895	1	4	2.890372	C38	2013
2.197225	0.333333	14.96132	2.833213	1	4	2.944439	C38	2014
2.197225	0.333333	14.70225	2.639057	1	4	2.995732	C38	2015
2.197225	0.333333	15.18862	2.564949	1	4	3.044522	C38	2016
2.197225	0.333333	14.82413	2.639057	1	4	3.091042	C38	2017
2.197225	0.333333	14.79541	1.94591	0	4	3.135494	C38	2018
2.197225	0.333333	14.82274	2.302585	0	4	3.178054	C38	2019
2.197225	0.333333	13.83708	1.791759	1	4	1.94591	C35	2010
2.197225	0.333333	14.33864	2.302585	1	4	2.079442	C35	2011
2.197225	0.333333	14.10751	1.791759	1	4	2.197225	C34	2012
1.94591	0.428571	14.11724	1.94591	1	4	2.302585	C34	2013
1.94591	0.428571	14.11724	1.791759	1	2	2.397895	C34	2014
1.94591	0.428571	14.11724	1.791759	1	4	2.484907	C34	2015
1.94591	0.428571	14.11724	1.94591	1	4	2.564949	C34	2016
1.94591	0.428571	14.11012	1.94591	1	4	2.639057	C34	2017
1.94591	0.428571	14.11012	2.484907	1	2	2.70805	C34	2018
2.197225	0.333333	14.44305	2.639057	1	4	2.944439	C34	2016
2.197225	0.333333	14.32329	2.197225	1	4	2.995732	C34	2017
1.94591	0.428571	14.44257	2.70805	1	4	3.044522	C34	2018
1.94591	0.428571	14.50274	2.197225	1	4	3.091042	C34	2019
2.197225	0.333333	13.19932	1.386294	1	4	2.079442	C35	2010
2.197225	0.333333	13.47302	2.70805	1	4	2.197225	C35	2011
2.197225	0.333333	13.62918	2.639057	1	4	2.302585	C35	2012
2.197225	0.333333	13.48144	2.302585	1	4	2.397895	C35	2013
2.197225	0.333333	13.57178	2.944439	1	4	2.484907	C35	2014
2.197225	0.333333	13.56294	2.639057	1	2	2.564949	C35	2015
2.079442	0.375	13.56948	2.397895	1	2	2.639057	C35	2016
2.197225	0.333333	13.59549	2.639057	1	4	2.70805	C35	2017
2.197225	0.333333	13.64354	2.079442	1	2	2.772589	C35	2018
2.197225	0.333333	12.92732	1.098612	1	3	2.079442	C36	2010
2.197225	0.333333	13.24847	2.397895	1	3	2.197225	C36	2011
2.079442	0.375	13.30468	1.791759	1	3	2.302585	C36	2012
2.197225	0.333333	13.30468	1.609438	1	3	2.397895	C36	2013
2.197225	0.333333	13.30468	1.386294	1	2	2.484907	C36	2014
2.197225	0.333333	13.30468	2.079442	1	2	2.564949	C36	2015
2.197225	0.333333	13.64116	1.609438	1	2	2.639057	C36	2016
2.197225	0.333333	13.80546	1.386294	1	3	2.70805	C36	2017
2.197225	0.333333	13.96393	1.609438	1	3	2.772589	C36	2018
2.197225	0.333333	13.97848	1.386294	1	2	2.833213	C36	2019
2.197225	0.333333	13.06219	1.098612	1	2	1.791759	C34	2010
2.079442	0.375	13.24458	1.94591	1	2	1.94591	C34	2011

2.197225	0.333333	13.57486	2.302585	0	2	2.079442	C34	2012
2.079442	0.375	13.53646	2.197225	0	2	2.197225	C34	2013
2.079442	0.375	13.56345	1.791759	0	2	2.302585	C34	2014
2.197225	0.333333	13.35427	2.197225	0	2	2.397895	C34	2015
2.197225	0.333333	14.03143	2.564949	0	2	2.484907	C34	2016
2.197225	0.333333	14.65841	2.302585	0	2	2.564949	C34	2017
2.484907	0.333333	14.49172	2.302585	0	2	2.639057	C34	2018
2.197225	0.333333	14.123	2.564949	1	3	2.564949	C26	2010
2.197225	0.333333	14.46533	2.197225	0	3	2.639057	C26	2011
2.079442	0.375	14.66363	2.197225	1	3	2.70805	C26	2012
2.197225	0.333333	13.8751	2.197225	1	3	2.772589	C26	2013
2.197225	0.333333	13.99775	1.609438	1	3	2.833213	C26	2014
2.197225	0.333333	14.03151	2.302585	1	3	2.890372	C26	2015
2.197225	0.333333	13.76422	2.302585	0	4	2.772589	C26	2011
2.197225	0.333333	13.74937	2.302585	0	4	2.833213	C26	2012
2.197225	0.333333	13.86801	2.197225	0	4	2.890372	C26	2013
2.197225	0.333333	13.99833	2.302585	0	4	2.944439	C26	2014
2.197225	0.333333	14.10542	2.772589	0	4	2.995732	C26	2015
2.197225	0.333333	13.98913	2.484907	0	4	3.044522	C26	2016
2.197225	0.333333	14.31671	2.197225	0	4	3.091042	C26	2017
2.197225	0.333333	14.02106	2.302585	0	4	3.135494	C26	2018
2.197225	0.333333	14.6421	2.564949	0	4	3.178054	C26	2019
1.94591	0.428571	13.75257	2.197225	1	4	2.079442	C38	2011
1.94591	0.428571	13.8622	2.484907	1	4	2.197225	C38	2012
1.94591	0.428571	14.19127	2.079442	1	4	2.302585	C38	2013
1.94591	0.428571	14.22018	2.079442	1	5	2.397895	C38	2014
1.94591	0.428571	14.1789	2.302585	1	5	2.484907	C38	2015
1.94591	0.428571	14.76732	2.197225	1	1	2.564949	C38	2016
1.791759	0.333333	14.85903	1.94591	1	1	2.639057	C38	2017
1.791759	0.333333	14.88596	2.197225	1	4	2.70805	C38	2018
1.94591	0.428571	14.85582	1.94591	1	1	2.772589	C38	2019
2.197225	0.444444	14.3343	2.079442	1	3	2.079442	C27	2011
2.197225	0.444444	14.83933	1.791759	1	3	2.197225	C27	2012
2.197225	0.444444	14.79661	1.386294	1	3	2.302585	C27	2013
2.079442	0.375	14.96672	2.079442	1	3	2.397895	C27	2014
2.197225	0.333333	14.87822	1.386294	1	3	2.484907	C27	2015
2.197225	0.333333	14.86724	1.609438	1	3	2.564949	C27	2016
2.079442	0.375	14.82907	1.94591	1	3	2.639057	C27	2017
2.079442	0.375	14.92381	1.94591	1	3	2.70805	C27	2018
2.079442	0.375	14.88949	1.609438	1	3	2.772589	C27	2019
2.197225	0.333333	13.74937	2.079442	0	4	2.564949	C35	2011
2.197225	0.333333	13.84991	2.197225	1	4	2.639057	C35	2012
2.197225	0.333333	13.94126	2.397895	1	4	2.70805	C35	2013
2.197225	0.333333	14.01272	2.639057	1	4	2.772589	C35	2014
2.197225	0.333333	14.05728	2.397895	1	4	2.833213	C35	2015
2.197225	0.333333	14.12666	2.639057	1	4	2.890372	C35	2016
2.197225	0.333333	14.14733	2.397895	1	4	2.944439	C35	2017
2.197225	0.333333	14.196	2.397895	1	4	2.995732	C35	2018
2.197225	0.333333	14.18015	2.302585	1	4	3.044522	C35	2019
1.94591	0.428571	14.00778	1.94591	1	4	2.995732	C34	2019
2.197225	0.333333	13.49365	1.94591	1	4	2.564949	C34	2011
2.197225	0.333333	13.45502	1.791759	1	4	2.639057	C34	2012
2.197225	0.333333	13.48074	1.791759	1	4	2.70805	C34	2013
2.079442	0.375	13.59011	1.791759	1	4	2.772589	C34	2014
2.197225	0.333333	13.72285	1.94591	1	4	2.833213	C34	2015
2.197225	0.333333	13.67395	2.197225	1	4	2.890372	C34	2016
2.197225	0.333333	13.96669	1.609438	1	4	2.944439	C34	2017
2.197225	0.333333	14.00149	1.791759	1	4	2.995732	C34	2018

2.197225	0.333333	13.976	1.791759	1	4	3.044522	C34	2019
2.079442	0.5	15.31925	2.079442	1	2	2.772589	C36	2011
2.197225	0.444444	15.39183	1.94591	1	2	2.833213	C36	2012
2.197225	0.444444	14.90252	2.397895	1	2	2.890372	C36	2013
2.197225	0.444444	14.65102	2.197225	1	2	2.944439	C36	2014
2.197225	0.444444	14.85579	2.564949	1	2	2.995732	C36	2015
2.197225	0.444444	15.05687	2.772589	1	2	3.044522	I64	2016
2.197225	0.444444	15.36859	2.397895	1	2	3.091042	I64	2017
2.079442	0.5	15.45256	2.397895	1	2	3.135494	I64	2018
2.197225	0.333333	14.47303	2.197225	1	4	2.302585	C13	2011
2.197225	0.333333	14.63088	1.94591	1	4	2.397895	C13	2012
2.197225	0.333333	14.7034	2.197225	1	4	2.484907	C13	2013
1.94591	0.428571	14.69098	2.302585	1	4	2.564949	C13	2014
1.94591	0.428571	14.69098	2.197225	1	4	2.639057	C13	2015
1.94591	0.428571	14.69098	2.079442	1	4	2.70805	C13	2016
1.94591	0.428571	14.44885	2.397895	1	4	2.772589	C13	2017
1.94591	0.428571	14.66472	1.94591	1	4	2.833213	C13	2018
1.94591	0.428571	14.82391	1.94591	1	4	2.890372	C13	2019
1.94591	0.428571	15.62416	2.197225	1	3	2.944439	I64	2016
1.94591	0.428571	15.79476	2.397895	1	3	2.995732	I64	2017
1.94591	0.428571	15.5441	2.833213	1	3	3.044522	I64	2018
1.94591	0.428571	16.02793	2.197225	1	3	3.091042	I64	2019
2.197225	0.333333	15.05475	2.197225	1	3	2.833213	C18	2019
2.197225	0.333333	14.34319	1.386294	1	2	2.197225	C35	2010
2.197225	0.333333	14.55138	2.484907	1	2	2.302585	C35	2011
2.197225	0.333333	14.22563	2.397895	1	2	2.397895	C35	2012
2.197225	0.333333	14.61329	2.197225	1	2	2.484907	C35	2013
2.197225	0.333333	14.44129	2.302585	1	2	2.564949	C35	2014
2.197225	0.333333	14.32233	2.397895	1	2	2.639057	C35	2015
2.197225	0.333333	14.37513	2.639057	1	2	2.70805	C35	2016
2.197225	0.333333	14.91755	2.995732	1	2	2.772589	C35	2017
1.94591	0.428571	14.975	2.772589	1	3	2.70805	C22	2019
2.197225	0.333333	13.99783	2.70805	1	5	2.944439	C13	2011
2.197225	0.333333	14.24404	2.639057	1	5	2.995732	C13	2012
2.197225	0.333333	14.23422	2.639057	1	5	3.044522	C13	2013
2.079442	0.375	14.30409	2.397895	1	5	3.091042	C13	2014
2.197225	0.333333	14.27294	2.639057	1	5	3.135494	C13	2015
2.197225	0.333333	14.27924	2.772589	1	5	3.178054	C13	2016
2.197225	0.333333	14.59941	2.833213	1	5	3.218876	C13	2017
2.197225	0.333333	14.7034	2.833213	1	5	3.258097	C13	2018
2.197225	0.333333	14.63088	3.044522	1	5	3.295837	C13	2019
2.197225	0.333333	13.60479	2.564949	1	1	1.791759	C18	2011
2.197225	0.333333	13.73213	1.791759	0	1	1.94591	C18	2012
2.197225	0.333333	13.62918	1.94591	0	1	2.079442	C18	2013
2.197225	0.333333	13.45884	2.302585	1	2	2.197225	C18	2014
2.197225	0.333333	13.39239	2.397895	1	3	2.397895	C18	2016
2.079442	0.375	14.92196	2.397895	1	3	2.833213	N77	2018
1.94591	0.428571	13.57153	2.639057	1	4	2.197225	C41	2011
1.94591	0.428571	13.74421	2.197225	1	4	2.302585	C41	2012
1.94591	0.428571	14.08653	1.791759	1	4	2.397895	C41	2013
1.94591	0.428571	14.14905	2.302585	1	4	2.484907	C41	2014
1.94591	0.428571	14.13622	2.197225	1	1	2.564949	C41	2015
1.94591	0.428571	14.13244	2.302585	1	1	2.639057	C41	2016
1.94591	0.428571	14.27332	1.609438	1	1	2.70805	C41	2017
1.94591	0.428571	14.30746	1.609438	1	4	2.772589	C41	2018
1.94591	0.428571	14.39828	2.079442	1	1	2.833213	C41	2019
1.94591	0.428571	13.166	1.94591	1	2	3.135494	C38	2011
1.94591	0.428571	13.15943	1.791759	1	2	3.178054	C38	2012

1.94591	0.428571	13.1527	1.609438	1	2	3.218876	C38	2013
1.94591	0.428571	13.23819	1.609438	1	4	3.258097	C38	2014
1.94591	0.428571	13.35538	1.94591	1	4	3.295837	C38	2015
1.94591	0.428571	13.45655	2.197225	1	4	3.332205	C38	2016
1.94591	0.428571	13.52302	2.397895	1	4	3.367296	C38	2017
1.94591	0.428571	13.61657	1.791759	1	4	3.401197	C38	2018
1.94591	0.428571	13.95684	1.386294	1	4	3.433987	C38	2019
2.197225	0.333333	12.7367	2.079442	1	2	2.197225	C32	2011
2.197225	0.333333	12.67608	2.079442	1	2	2.302585	C32	2012
2.197225	0.333333	13.21767	1.94591	1	2	2.397895	C32	2013
2.197225	0.333333	13.21767	1.791759	1	2	2.484907	C32	2014
2.197225	0.333333	13.12236	2.079442	1	2	2.564949	C32	2015
2.197225	0.333333	13.12236	2.079442	1	2	2.639057	C32	2016
2.197225	0.333333	13.73213	1.791759	1	2	2.70805	C32	2017
2.197225	0.333333	13.73213	1.609438	1	2	2.772589	C32	2018
2.079442	0.375	13.73213	1.791759	1	2	2.833213	C32	2019
2.197225	0.333333	13.73722	2.397895	1	3	2.302585	C26	2011
2.197225	0.333333	13.9778	2.397895	1	3	2.397895	C26	2012
2.197225	0.333333	14.14805	2.772589	1	3	2.484907	C26	2013
2.197225	0.333333	14.16243	2.484907	1	4	2.564949	C26	2014
2.197225	0.333333	14.71567	2.397895	1	3	2.639057	C26	2015
2.197225	0.333333	14.84877	2.639057	1	3	2.70805	C26	2016
2.197225	0.333333	14.89647	2.079442	1	3	2.772589	C26	2017
2.197225	0.333333	14.70689	2.484907	1	4	2.833213	C26	2018
2.197225	0.333333	14.32833	1.94591	1	3	2.890372	C26	2019
1.609438	0.4	13.93658	2.079442	1	2	2.639057	C29	2011
1.94591	0.428571	13.68609	2.079442	1	2	2.70805	C29	2012
1.94591	0.428571	13.77365	1.791759	1	2	2.772589	C29	2013
1.94591	0.428571	13.57535	2.197225	1	2	2.833213	C29	2014
1.94591	0.428571	13.67164	1.94591	1	2	2.890372	C29	2015
1.94591	0.428571	13.82348	1.94591	1	2	2.944439	C29	2016
1.94591	0.428571	14.02658	2.397895	1	2	2.995732	C29	2017
1.94591	0.428571	14.28757	1.386294	1	2	3.044522	C29	2018
1.94591	0.428571	14.28757	1.791759	1	2	3.091042	C29	2019
1.94591	0.428571	14.23888	2.302585	1	2	3.135494	E48	2011
1.94591	0.428571	14.32052	2.197225	1	2	3.178054	E48	2012
1.94591	0.428571	14.0976	2.302585	1	2	3.218876	E48	2013
1.94591	0.428571	14.29862	2.079442	1	4	3.258097	E48	2014
1.94591	0.428571	14.30274	2.397895	1	4	3.295837	E48	2015
1.94591	0.428571	14.42891	2.484907	1	4	3.332205	E48	2016
1.94591	0.428571	14.69609	2.639057	1	4	3.367296	E48	2017
1.791759	0.5	14.68253	2.70805	1	4	3.401197	E48	2018
1.609438	0.4	12.92756	2.484907	1	4	2.564949	C26	2011
1.609438	0.4	14.4033	1.791759	1	4	2.639057	C26	2012
1.609438	0.4	14.4033	1.609438	1	4	2.70805	C26	2013
1.609438	0.4	14.6056	2.079442	1	4	2.772589	C26	2014
1.609438	0.4	14.5682	2.079442	1	4	2.833213	C26	2015
1.609438	0.4	14.60397	1.791759	1	4	2.890372	C26	2016
1.609438	0.4	14.60397	1.609438	1	4	2.944439	C26	2017
1.609438	0.4	14.60397	1.791759	1	4	2.995732	C26	2018
1.609438	0.4	14.60397	1.791759	1	4	3.044522	C26	2019
2.197225	0.333333	14.02269	2.397895	1	3	2.484907	C36	2011
2.197225	0.333333	13.94302	2.079442	1	3	2.564949	C36	2012
2.197225	0.333333	14.16511	2.397895	1	3	2.639057	C36	2013
2.197225	0.333333	14.48947	2.197225	1	3	2.70805	C36	2014
2.197225	0.333333	14.37798	2.302585	1	4	2.772589	C36	2015
2.197225	0.333333	14.55267	2.397895	1	4	2.833213	C36	2016
2.197225	0.333333	14.64406	2.079442	1	3	2.890372	C36	2017

2.197225	0.333333	14.64668	2.302585	1	3	2.944439	C36	2018
2.197225	0.333333	14.57538	2.197225	1	3	2.995732	C36	2019
1.94591	0.428571	13.00493	2.079442	0	4	3.044522	C26	2015
1.94591	0.428571	12.88892	2.484907	0	4	3.091042	C26	2016
1.94591	0.428571	13.14471	2.944439	1	4	3.135494	C26	2017
1.94591	0.428571	13.13922	2.639057	1	4	3.178054	C26	2018
1.94591	0.428571	13.50844	2.302585	1	4	3.218876	C26	2019
1.94591	0.428571	14.31021	2.397895	0	2	1.94591	C37	2011
1.94591	0.428571	14.44678	2.772589	1	2	2.079442	C36	2012
1.94591	0.428571	14.4782	2.302585	1	2	2.197225	C36	2013
1.94591	0.428571	14.4782	2.639057	1	2	2.302585	C36	2014
1.94591	0.428571	14.63529	2.70805	1	2	2.397895	C36	2015
1.94591	0.428571	14.69514	2.995732	1	2	2.484907	C36	2016
1.94591	0.428571	14.74373	2.397895	1	2	2.564949	C36	2017
1.94591	0.428571	14.67383	2.70805	1	3	2.639057	C36	2018
1.94591	0.428571	13.44445	1.94591	0	1	2.772589	C37	2011
1.94591	0.428571	13.43498	2.302585	0	1	2.833213	C36	2012
1.94591	0.428571	13.6293	2.197225	0	1	2.890372	C36	2013
1.94591	0.428571	13.73874	1.94591	0	2	2.944439	C36	2014
1.94591	0.428571	13.65217	2.197225	0	1	2.995732	C36	2015
1.94591	0.428571	13.83972	1.791759	0	2	3.044522	C36	2016
1.94591	0.428571	13.74176	2.302585	1	2	3.091042	C36	2017
1.94591	0.428571	14.04734	2.197225	0	2	3.135494	C36	2018
1.94591	0.428571	14.097	2.197225	1	2	3.178054	C36	2019
2.197225	0.333333	14.05885	2.197225	1	4	1.609438	C26	2011
2.197225	0.333333	14.02577	1.609438	1	4	1.791759	C26	2012
2.197225	0.333333	14.44678	1.609438	1	4	1.94591	C26	2013
2.197225	0.333333	14.45209	2.197225	1	4	2.079442	C26	2014
2.197225	0.333333	14.50866	2.397895	1	4	2.197225	C26	2015
2.197225	0.333333	14.53335	2.833213	1	4	2.302585	C26	2016
2.197225	0.333333	14.38406	2.197225	1	4	2.484907	C26	2018
2.197225	0.333333	14.52364	2.197225	1	4	2.564949	C26	2019
2.197225	0.333333	14.0072	1.609438	0	1	2.397895	C23	2011
2.197225	0.333333	13.8662	1.94591	0	1	2.484907	C23	2012
2.197225	0.333333	13.81551	2.079442	0	1	2.564949	C23	2013
2.197225	0.333333	13.8643	1.609438	0	1	2.639057	C23	2014
2.197225	0.333333	13.59237	2.484907	0	1	2.70805	C23	2015
2.079442	0.375	13.94654	2.397895	0	1	2.772589	C23	2016
2.197225	0.333333	14.2329	2.397895	0	1	2.833213	C23	2017
2.197225	0.333333	14.44038	2.302585	1	1	2.890372	C23	2018
2.197225	0.333333	14.51821	2.302585	1	1	2.944439	C23	2019
1.94591	0.428571	14.95013	2.079442	1	4	1.098612	C37	2011
1.94591	0.428571	14.89392	2.197225	1	4	1.386294	C36	2012
1.94591	0.428571	14.91412	1.791759	1	4	1.609438	C36	2013
2.197225	0.333333	14.91412	1.94591	1	4	1.791759	C36	2014
2.197225	0.333333	14.91412	1.94591	1	4	1.94591	C36	2015
2.197225	0.333333	14.83796	2.397895	1	3	2.079442	C36	2016
2.197225	0.333333	14.73579	2.484907	1	4	2.197225	C36	2017
2.197225	0.333333	14.97154	2.995732	1	4	2.302585	C36	2018
2.197225	0.333333	15.12923	2.639057	1	4	2.397895	C36	2019
2.397895	0.363636	14.63912	2.484907	1	4	2.302585	C27	2011
2.397895	0.363636	14.67076	2.079442	1	4	2.397895	C27	2012
2.397895	0.363636	14.64838	2.564949	1	4	2.484907	C27	2013
2.397895	0.363636	14.65233	2.397895	1	4	2.564949	C27	2014
2.397895	0.363636	14.67688	2.302585	1	4	2.639057	C27	2015
2.197225	0.333333	14.69746	2.484907	1	4	2.70805	C27	2016
2.079442	0.375	14.39409	2.197225	1	4	2.772589	C27	2017
2.197225	0.333333	14.35556	2.079442	1	5	2.833213	C27	2018

2.197225	0.333333	14.90549	2.302585	1	4	2.890372	C27	2019
1.94591	0.428571	14.43107	2.079442	1	4	2.70805	C24	2011
1.791759	0.5	14.29513	1.791759	1	4	2.772589	C24	2012
1.94591	0.428571	14.4307	2.397895	1	4	2.833213	C24	2013
1.94591	0.428571	14.35783	2.197225	1	4	2.890372	C24	2014
1.94591	0.428571	14.28551	2.397895	1	4	2.944439	C24	2015
1.94591	0.428571	14.28501	2.079442	1	1	2.995732	C24	2016
1.94591	0.428571	14.28501	2.564949	1	1	3.044522	C24	2017
1.94591	0.428571	14.57641	2.484907	1	4	3.091042	C24	2018
1.94591	0.428571	15.27413	2.772589	1	1	3.135494	C24	2019
2.484907	0.333333	14.3125	2.302585	1	4	2.079442	C38	2011
2.484907	0.333333	14.39655	2.484907	1	4	2.197225	C38	2012
2.079442	0.5	14.5439	2.079442	1	4	2.302585	C38	2013
2.302585	0.4	14.12439	1.609438	1	4	2.397895	C38	2014
2.197225	0.333333	14.22603	2.197225	1	4	2.484907	C38	2015
1.94591	0.428571	14.51195	1.791759	1	2	2.564949	C38	2016
1.94591	0.428571	14.44954	2.564949	1	2	2.639057	C38	2017
1.94591	0.428571	14.6219	2.302585	1	2	2.70805	C38	2018
2.197225	0.333333	14.02374	1.94591	1	4	2.484907	F52	2011
2.197225	0.333333	13.9402	2.484907	1	4	2.564949	F52	2012
2.197225	0.333333	13.98407	1.94591	1	4	2.639057	F52	2013
2.197225	0.333333	14.00811	1.791759	1	4	2.70805	F52	2014
2.197225	0.333333	13.54619	2.484907	1	4	2.772589	F52	2015
2.197225	0.333333	13.6195	2.302585	1	4	2.833213	F52	2016
2.197225	0.333333	13.61596	1.94591	1	4	2.890372	F52	2017
2.197225	0.333333	13.60898	2.484907	1	4	2.944439	F52	2018
1.609438	0.4	14.58483	2.197225	1	2	2.484907	C18	2018
1.609438	0.4	14.58483	2.397895	1	2	2.564949	C18	2019
2.197225	0.333333	13.28957	1.94591	1	1	3.091042	C32	2011
2.197225	0.333333	13.52707	2.302585	1	1	3.135494	C38	2012
1.94591	0.428571	13.72131	2.197225	1	1	3.178054	C38	2013
1.791759	0.5	14.43748	1.94591	1	1	3.218876	C38	2014
1.94591	0.428571	13.71292	2.564949	1	3	3.258097	C38	2015
1.94591	0.428571	13.77988	2.564949	1	3	3.295837	C38	2016
1.94591	0.428571	14.10354	2.944439	1	1	3.332205	C38	2017
2.197225	0.333333	13.98745	2.944439	1	1	3.401197	C38	2019
2.197225	0.333333	13.14217	1.94591	1	2	2.302585	C30	2011
2.197225	0.333333	13.42985	1.098612	1	2	2.397895	C30	2012
2.197225	0.333333	13.62918	2.079442	1	2	2.484907	C30	2013
2.197225	0.333333	13.80546	2.397895	1	2	2.564949	C30	2014
2.197225	0.333333	13.80546	1.791759	1	2	2.639057	C30	2015
2.197225	0.333333	13.80546	2.639057	1	2	2.70805	C30	2016
2.197225	0.333333	13.97882	2.944439	1	2	2.772589	I64	2017
2.197225	0.333333	13.43454	2.639057	1	2	2.833213	I64	2018
1.94591	0.428571	12.68355	1.94591	1	2	2.079442	C35	2011
1.94591	0.428571	12.84212	2.197225	1	2	2.197225	C35	2012
1.94591	0.428571	13.06854	1.791759	1	2	2.302585	C35	2013
1.94591	0.428571	13.09971	1.94591	1	2	2.397895	C35	2014
1.94591	0.428571	13.06537	1.609438	1	2	2.484907	C35	2015
2.197225	0.333333	13.41533	1.94591	1	3	2.564949	C38	2011
2.197225	0.333333	13.64981	1.609438	1	3	2.639057	C38	2012
2.197225	0.333333	13.51441	1.609438	1	3	2.70805	C38	2013
2.079442	0.375	13.50531	1.791759	1	3	2.772589	C38	2014
1.94591	0.428571	13.91264	2.70805	1	3	2.833213	C38	2015
1.94591	0.428571	13.70536	1.609438	1	2	2.484907	C30	2011
1.94591	0.428571	13.75788	2.079442	1	2	2.564949	C30	2012
1.94591	0.428571	13.85252	2.484907	1	2	2.639057	C30	2013
1.94591	0.428571	13.7424	1.386294	1	4	2.302585	C36	2011

1.94591	0.428571	13.77453	2.197225	1	4	2.397895	C36	2012
1.94591	0.428571	13.77874	2.079442	1	4	2.484907	C36	2013
1.94591	0.428571	13.65252	2.302585	1	4	2.564949	C36	2014
1.94591	0.428571	13.82241	1.94591	1	4	2.639057	C36	2015
1.94591	0.428571	13.84827	1.94591	1	4	2.70805	C36	2016
2.197225	0.333333	13.54107	1.609438	1	1	1.386294	C26	2011
2.197225	0.333333	13.56705	2.302585	1	1	1.609438	C26	2012
2.197225	0.333333	13.61864	2.564949	1	1	1.791759	C26	2013
2.197225	0.333333	13.55674	2.302585	1	1	1.94591	C26	2014
2.197225	0.333333	13.48088	2.079442	1	1	2.079442	C26	2015
2.079442	0.375	13.4806	2.197225	1	1	2.197225	C26	2016
2.197225	0.333333	13.60479	2.564949	1	1	2.302585	C26	2017
2.197225	0.333333	13.95684	1.609438	1	1	2.397895	C26	2018
2.197225	0.333333	14.3343	1.94591	1	1	2.484907	C26	2019
2.197225	0.333333	13.45884	2.079442	1	3	2.70805	C34	2011
2.197225	0.333333	13.45884	2.302585	1	3	2.772589	C34	2012
1.94591	0.428571	13.45884	1.94591	1	3	2.833213	C34	2013
1.94591	0.428571	13.45884	1.94591	1	2	2.890372	C34	2014
1.94591	0.428571	13.45884	2.397895	1	2	2.995732	C34	2016
1.94591	0.428571	13.45884	2.079442	1	2	3.044522	C34	2017
1.94591	0.428571	13.45884	2.197225	1	3	3.091042	C34	2018
1.94591	0.428571	13.71015	1.791759	1	2	3.135494	C34	2019
2.197225	0.333333	14.50349	2.564949	0	4	2.944439	C26	2019
2.197225	0.333333	14.20077	1.94591	1	1	2.639057	C35	2018
2.197225	0.333333	14.3576	2.302585	1	1	2.70805	C35	2019
2.197225	0.444444	13.41503	1.386294	1	2	2.564949	C38	2011
2.197225	0.444444	13.41503	2.079442	1	2	2.639057	C38	2012
2.197225	0.444444	13.62918	1.791759	1	2	2.70805	C38	2013
2.079442	0.5	13.42985	2.302585	1	2	2.772589	C38	2014
1.94591	0.428571	13.59237	2.302585	1	2	2.833213	C38	2015
2.197225	0.333333	14.41069	2.197225	1	1	2.70805	C14	2011
2.197225	0.333333	14.30064	2.397895	1	1	2.772589	C14	2012
2.197225	0.333333	14.20789	2.197225	1	1	2.833213	C14	2013
2.197225	0.333333	14.26116	2.079442	1	3	2.890372	C14	2014
2.197225	0.333333	14.51469	2.484907	1	1	2.944439	C14	2015
2.197225	0.333333	14.5235	2.197225	0	1	2.995732	C14	2016
2.197225	0.333333	14.65276	2.639057	1	3	3.044522	C14	2017
2.079442	0.375	14.73826	2.397895	1	3	3.091042	C14	2018
1.94591	0.571429	13.97854	2.484907	1	3	3.135494	C14	2019
2.197225	0.333333	14.56693	1.386294	1	4	2.484907	C35	2011
2.197225	0.333333	14.56693	2.484907	1	4	2.564949	C35	2012
2.197225	0.333333	14.56693	1.609438	0	4	2.639057	C35	2013
2.197225	0.333333	14.56693	2.197225	1	2	2.70805	C35	2014
2.197225	0.333333	14.65276	1.791759	1	4	2.772589	C35	2015
2.197225	0.333333	14.70915	1.94591	1	4	2.833213	C35	2016
2.197225	0.333333	14.63088	2.079442	1	2	2.890372	C35	2017
2.197225	0.333333	14.71567	2.639057	1	2	2.944439	C35	2018
2.197225	0.333333	14.66733	2.197225	1	2	2.995732	C35	2019
2.197225	0.333333	13.11836	1.791759	0	2	2.197225	C39	2011
2.197225	0.333333	13.50354	2.197225	1	2	2.302585	C39	2012
2.197225	0.333333	14.77421	2.079442	1	2	2.397895	C39	2013
2.197225	0.333333	14.04781	2.197225	0	2	2.484907	C39	2014
2.197225	0.333333	14.84452	2.484907	0	3	2.564949	C39	2015
2.197225	0.333333	15.46774	2.484907	0	3	2.639057	C39	2016
2.197225	0.333333	15.37842	2.302585	0	3	2.70805	C39	2017
2.302585	0.4	12.64433	2.079442	1	1	2.70805	C14	2012
2.197225	0.444444	12.94801	2.197225	1	1	2.772589	C14	2013
2.197225	0.444444	13.27491	1.94591	1	1	2.833213	C14	2014

2.197225	0.333333	13.29194	1.94591	1	1	2.890372	C14	2015
2.197225	0.333333	13.60787	2.302585	1	1	2.944439	C14	2016
2.197225	0.333333	13.93169	2.397895	1	1	2.995732	C14	2017
2.197225	0.333333	14.0209	2.484907	1	1	3.044522	C14	2018
2.397895	0.363636	14.05074	2.079442	1	1	3.091042	C14	2019
2.197225	0.333333	13.87378	2.302585	1	4	3.091042	C36	2012
2.197225	0.333333	13.83531	1.94591	1	4	3.135494	C36	2013
2.197225	0.333333	14.00265	1.94591	1	4	3.178054	C36	2014
2.197225	0.333333	14.15903	2.397895	1	4	3.218876	C36	2015
2.197225	0.333333	14.26531	2.079442	1	4	3.258097	C36	2016
2.197225	0.333333	14.21012	2.302585	1	3	3.295837	C36	2017
2.197225	0.333333	14.25415	2.079442	1	3	3.332205	C36	2018
2.197225	0.333333	14.78818	2.197225	1	3	3.367296	C36	2019
2.397895	0.363636	14.74712	2.397895	1	1	2.995732	C26	2012
2.397895	0.363636	14.7034	1.94591	1	1	3.044522	C26	2013
2.302585	0.4	14.76214	2.079442	1	1	3.091042	C26	2014
2.197225	0.333333	14.46293	1.791759	1	1	3.135494	C26	2015
2.197225	0.333333	14.32707	1.609438	1	1	3.178054	C26	2016
2.079442	0.375	14.45341	1.386294	1	1	3.218876	C26	2017
2.079442	0.375	14.72175	1.609438	1	1	3.258097	C26	2018
2.079442	0.375	14.71819	1.94591	1	1	3.295837	C26	2019
2.197225	0.333333	15.04725	1.098612	1	4	2.890372	C35	2012
2.197225	0.333333	14.8153	1.609438	1	4	2.944439	C35	2013
2.197225	0.333333	14.57702	1.098612	1	4	2.995732	C35	2014
2.197225	0.333333	14.60032	2.079442	1	4	3.044522	C35	2015
2.197225	0.333333	13.99833	2.197225	1	4	3.091042	C35	2016
2.197225	0.333333	13.71193	2.079442	1	4	3.135494	C35	2017
2.197225	0.333333	14.70657	1.94591	1	4	3.178054	C35	2018
2.197225	0.333333	13.00987	2.302585	1	4	3.218876	C35	2019
2.197225	0.333333	13.99783	1.386294	1	3	3.178054	C26	2012
2.197225	0.333333	14.00182	2.079442	1	3	3.218876	C26	2013
2.197225	0.333333	14.52231	1.791759	1	3	3.258097	C26	2014
2.197225	0.333333	15.0933	1.609438	1	3	3.295837	C26	2015
2.197225	0.333333	14.25454	2.302585	1	3	3.332205	C26	2016
2.197225	0.333333	14.36635	2.484907	1	3	3.367296	C26	2017
2.197225	0.333333	14.6803	2.833213	1	3	3.401197	C26	2018
2.197225	0.333333	13.59686	1.609438	0	4	2.833213	C38	2012
2.197225	0.333333	13.55842	1.386294	0	4	2.890372	C38	2013
2.197225	0.333333	13.77469	1.386294	0	4	2.944439	C38	2014
2.302585	0.4	14.02755	1.94591	1	1	2.995732	C19	2012
2.197225	0.333333	14.12513	2.302585	1	1	3.044522	C19	2013
2.197225	0.333333	14.5218	2.302585	1	1	3.091042	C19	2014
2.197225	0.333333	14.45573	2.397895	1	1	3.135494	C19	2015
1.94591	0.428571	14.44933	2.639057	1	1	3.178054	C19	2016
1.94591	0.428571	14.33001	1.791759	1	1	3.218876	C19	2017
1.94591	0.428571	14.41435	2.079442	1	1	3.258097	C19	2018
1.94591	0.428571	15.15838	2.079442	1	1	3.295837	C19	2019
1.609438	0.4	13.52783	1.609438	1	3	2.639057	C27	2012
1.609438	0.4	13.77469	1.609438	1	3	2.70805	C27	2013
1.609438	0.4	13.79623	1.609438	1	3	2.772589	C27	2014
1.609438	0.4	13.89525	1.94591	1	3	2.833213	C27	2015
1.609438	0.4	13.90581	2.302585	1	3	2.890372	C27	2016
1.609438	0.4	14.06549	2.197225	1	3	2.944439	C27	2017
1.609438	0.4	14.30194	2.302585	1	3	2.995732	C27	2018
1.609438	0.4	15.01548	2.079442	1	3	3.044522	C27	2019
2.197225	0.333333	14.06627	1.609438	1	3	2.397895	C38	2012
2.197225	0.333333	13.9761	2.302585	1	3	2.484907	C38	2013
2.197225	0.333333	14.16518	1.94591	1	3	2.564949	C38	2014

2.197225	0.333333	14.28464	1.609438	1	3	2.639057	C38	2015
2.197225	0.333333	14.47127	2.079442	1	3	2.70805	C38	2016
2.197225	0.333333	14.62893	1.791759	1	3	2.772589	C38	2017
2.079442	0.375	12.94825	2.079442	0	4	2.197225	C35	2015
2.197225	0.333333	14.06471	2.079442	1	4	2.302585	C27	2016
2.197225	0.333333	14.07495	2.197225	1	4	2.397895	C27	2017
1.94591	0.428571	14.1328	2.484907	1	3	2.944439	C39	2012
1.94591	0.428571	14.27931	1.791759	1	3	2.995732	C39	2013
1.94591	0.428571	14.26951	2.197225	1	3	3.044522	C39	2014
1.94591	0.428571	14.39845	1.94591	1	3	3.091042	C39	2015
1.94591	0.428571	14.50605	2.397895	1	3	3.135494	C39	2016
2.197225	0.333333	14.77647	2.639057	1	3	3.178054	C39	2017
2.197225	0.333333	14.86012	2.484907	1	3	3.218876	C39	2018
1.94591	0.428571	13.05878	2.079442	1	1	2.397895	C38	2012
1.94591	0.428571	13.82109	2.302585	1	1	2.484907	C38	2013
1.94591	0.428571	13.82109	2.564949	1	1	2.564949	C38	2014
1.94591	0.428571	14.10069	2.890372	1	1	2.639057	C38	2015
1.94591	0.428571	14.87677	2.995732	1	1	2.70805	C38	2016
1.94591	0.428571	14.8848	2.995732	1	1	2.772589	C38	2017
2.197225	0.333333	13.90435	1.94591	1	3	2.079442	C34	2012
2.197225	0.333333	13.96229	1.791759	1	3	2.197225	C34	2013
2.079442	0.375	14.10031	2.079442	1	3	2.302585	C34	2014
2.197225	0.333333	14.13759	2.484907	1	3	2.397895	C34	2015
2.197225	0.333333	14.30409	2.079442	1	3	2.484907	C34	2016
2.197225	0.333333	14.72377	2.302585	1	3	2.564949	C34	2017
2.197225	0.333333	14.6085	1.791759	1	3	2.639057	C34	2018
2.197225	0.333333	14.88366	1.791759	1	3	2.70805	C34	2019
2.197225	0.333333	13.94126	1.609438	0	4	2.397895	C18	2012
2.197225	0.333333	13.94126	2.397895	0	1	2.484907	C18	2013
2.197225	0.333333	13.94126	2.397895	0	4	2.564949	C18	2014
2.197225	0.333333	13.97286	2.197225	1	3	2.639057	C18	2015
2.197225	0.333333	13.99483	1.791759	1	1	2.70805	C18	2016
2.197225	0.333333	14.39571	1.609438	1	1	2.772589	C18	2017
2.197225	0.333333	14.39571	2.197225	1	3	2.833213	C18	2018
2.197225	0.333333	14.24313	1.94591	1	1	2.890372	C18	2019
2.197225	0.333333	14.09366	2.302585	1	4	3.091042	C27	2012
2.197225	0.333333	14.3315	2.564949	1	4	3.135494	C27	2013
2.197225	0.333333	14.2683	2.564949	1	4	3.178054	C27	2014
2.197225	0.333333	14.18154	2.564949	1	4	3.218876	C27	2015
2.197225	0.333333	14.38711	2.484907	1	4	3.258097	C27	2016
2.197225	0.333333	14.35661	2.564949	1	4	3.295837	C27	2017
2.197225	0.333333	14.60646	2.564949	1	4	3.332205	C27	2018
2.197225	0.333333	14.35778	2.197225	1	4	3.367296	C27	2019
2.197225	0.333333	13.21385	1.791759	1	3	2.197225	C35	2012
2.197225	0.333333	13.66352	1.94591	1	3	2.302585	C35	2013
2.197225	0.333333	13.07191	2.302585	1	3	2.397895	C35	2014
2.197225	0.333333	13.17134	2.484907	1	3	2.484907	C35	2015
2.197225	0.333333	13.87236	2.079442	1	3	2.70805	C35	2018
2.197225	0.333333	13.93808	1.609438	1	3	2.772589	C35	2019
2.484907	0.333333	13.88371	1.791759	1	4	2.484907	C27	2012
1.94591	0.428571	14.38099	2.397895	1	4	2.564949	C27	2013
1.94591	0.428571	14.43963	2.564949	1	4	2.639057	C27	2014
1.94591	0.428571	14.41495	2.484907	1	4	2.70805	C27	2015
1.609438	0.4	14.47308	2.772589	1	3	2.833213	C27	2017
1.609438	0.4	14.18804	2.079442	1	4	2.890372	C27	2018
1.609438	0.4	14.20342	2.302585	1	3	2.944439	C27	2019
2.197225	0.333333	14.22941	1.791759	1	1	2.564949	C29	2012
2.197225	0.333333	14.8487	2.397895	1	1	2.639057	C29	2013

2.079442	0.375	14.28551	2.484907	1	1	2.70805	C29	2014
2.197225	0.333333	15.34113	2.079442	1	3	2.772589	C29	2015
2.197225	0.333333	13.24299	2.079442	1	3	2.564949	C13	2012
2.197225	0.333333	13.67256	2.302585	1	3	2.639057	C13	2013
2.197225	0.333333	14.12666	1.609438	1	3	2.70805	C13	2014
2.197225	0.333333	14.54243	1.609438	1	3	2.772589	C13	2015
2.197225	0.333333	14.54374	1.386294	1	3	2.833213	C13	2016
2.197225	0.333333	14.96943	2.079442	1	3	2.890372	C13	2017
2.197225	0.333333	15.09731	2.079442	1	3	2.944439	C13	2018
2.197225	0.333333	15.22217	1.94591	1	3	2.995732	C13	2019
2.197225	0.333333	14.05367	2.639057	1	1	2.484907	A04	2012
1.609438	0.4	14.34313	2.890372	1	1	2.564949	A04	2013
1.609438	0.4	14.25499	2.639057	1	2	2.639057	A04	2014
1.609438	0.4	14.22217	2.302585	1	1	2.70805	A04	2015
1.609438	0.6	14.06821	2.70805	1	2	2.772589	A04	2016
1.609438	0.6	14.30642	2.833213	1	2	2.833213	A04	2017
1.94591	0.428571	14.30372	2.484907	1	1	2.890372	A04	2018
1.609438	0.6	14.66763	2.70805	1	2	2.944439	A04	2019
2.397895	0.363636	13.15386	2.079442	1	1	2.484907	F52	2012
2.197225	0.333333	13.19468	2.772589	1	1	2.564949	F52	2013
2.197225	0.333333	13.14373	2.484907	1	1	2.639057	F52	2014
2.197225	0.333333	13.14373	2.70805	1	1	2.70805	F52	2015
2.197225	0.333333	13.18703	2.70805	1	1	2.772589	F52	2016
2.197225	0.333333	13.22852	1.94591	1	1	2.833213	F52	2017
2.079442	0.375	13.22852	2.397895	1	1	2.890372	F52	2018
2.197225	0.333333	13.69988	2.484907	1	1	2.944439	F52	2019
2.197225	0.333333	15.13727	1.94591	1	4	2.70805	C33	2012
2.197225	0.333333	15.13727	1.791759	1	4	2.772589	C33	2013
2.197225	0.333333	15.00943	1.791759	1	4	2.833213	C33	2014
2.197225	0.333333	15.53828	2.302585	1	4	2.890372	C33	2015
2.079442	0.375	15.42495	2.484907	1	4	2.944439	C33	2016
2.197225	0.333333	15.42495	2.079442	1	4	2.995732	C33	2017
2.197225	0.333333	15.2506	1.94591	1	4	3.044522	C33	2018
2.197225	0.333333	15.64006	2.197225	1	4	3.091042	C33	2019
2.397895	0.363636	13.83921	1.791759	1	5	2.944439	C36	2012
2.397895	0.363636	13.83948	2.197225	1	5	2.995732	C36	2013
2.397895	0.363636	14.03785	2.197225	1	5	3.044522	C36	2014
2.197225	0.333333	14.27395	2.397895	1	5	3.091042	C36	2015
2.197225	0.333333	14.33823	2.564949	1	5	3.135494	C36	2016
2.197225	0.333333	14.33484	1.94591	1	5	3.178054	C36	2017
2.197225	0.333333	15.08864	2.079442	1	5	3.218876	C36	2018
2.197225	0.333333	14.90505	1.791759	1	3	3.091042	C38	2017
2.197225	0.333333	15.03711	2.079442	1	3	3.135494	C38	2018
2.197225	0.333333	15.28163	1.386294	1	3	3.178054	C38	2019
2.197225	0.333333	13.60479	1.609438	1	1	2.944439	C34	2013
2.197225	0.333333	14.41435	2.079442	1	1	2.995732	C36	2014
2.197225	0.333333	14.29794	1.791759	1	1	3.044522	C36	2015
2.397895	0.363636	14.29794	1.386294	1	1	3.091042	C36	2016
2.397895	0.363636	13.92194	1.94591	1	1	3.135494	C36	2017
2.397895	0.363636	14.34726	1.386294	1	1	3.178054	C36	2018
2.197225	0.333333	14.80665	2.564949	1	1	2.70805	G59	2017
1.94591	0.428571	13.71015	1.94591	1	1	2.397895	C14	2013
1.94591	0.428571	13.77469	1.94591	1	1	2.484907	C14	2014
1.94591	0.428571	13.48701	2.397895	1	1	2.564949	C14	2015
1.94591	0.428571	13.75364	1.791759	1	1	2.639057	C14	2016
1.94591	0.428571	14.04662	2.197225	1	1	2.70805	C14	2017
1.609438	0.6	13.82546	2.397895	1	1	2.833213	C14	2019
1.94591	0.428571	13.083	2.079442	0	3	2.079442	C38	2015

1.94591	0.428571	13.71015	2.484907	1	3	2.197225	C38	2016
1.94591	0.428571	13.71015	2.079442	1	3	2.302585	C38	2017
2.197225	0.333333	14.75805	1.791759	1	5	2.70805	C36	2013
2.197225	0.333333	15.0978	2.079442	1	1	2.772589	C36	2014
2.079442	0.375	14.86062	1.94591	1	5	2.833213	C36	2015
2.197225	0.333333	14.5048	2.397895	1	5	2.890372	C36	2016
2.197225	0.333333	14.46057	2.302585	1	5	2.944439	C36	2017
2.197225	0.333333	14.70894	2.197225	1	1	2.995732	C36	2018
2.197225	0.333333	14.65914	2.197225	1	1	3.044522	C36	2019
2.197225	0.333333	13.99324	1.791759	1	4	2.397895	C13	2014
2.197225	0.333333	14.3924	1.791759	1	4	2.484907	C13	2015
2.197225	0.333333	14.38739	2.302585	1	4	2.564949	C13	2016
2.197225	0.333333	14.35702	1.94591	1	4	2.639057	C13	2017
2.079442	0.375	14.31082	2.302585	1	4	2.70805	C13	2018
2.197225	0.333333	13.06748	2.197225	1	4	2.484907	C27	2014
1.94591	0.428571	13.9666	2.079442	1	4	2.639057	C27	2016
1.94591	0.428571	13.9789	2.302585	1	4	2.70805	C27	2017
1.94591	0.428571	13.96943	2.302585	1	4	2.772589	C27	2018
1.94591	0.428571	14.17856	1.94591	1	4	2.833213	C27	2019
1.94591	0.428571	13.62459	1.94591	0	4	3.218876	C39	2017
1.94591	0.428571	14.22683	1.94591	0	3	3.258097	C39	2018
1.94591	0.428571	14.46742	1.94591	0	3	3.295837	C39	2019
2.197225	0.333333	13.54213	1.791759	1	1	2.772589	C35	2014
2.197225	0.333333	13.53381	1.94591	1	1	2.833213	C35	2015
2.197225	0.333333	13.55428	2.484907	1	1	2.890372	C35	2016
2.197225	0.333333	13.71015	1.791759	1	1	2.944439	C35	2017
2.197225	0.333333	13.98102	1.94591	1	1	2.995732	C35	2018
2.197225	0.333333	14.20755	2.197225	1	1	3.044522	C35	2019
2.079442	0.375	13.12636	2.302585	1	1	2.302585	C18	2014
2.079442	0.375	13.60108	1.386294	1	1	2.397895	C41	2015
2.197225	0.333333	13.48701	1.791759	1	1	2.484907	C41	2016
2.197225	0.333333	13.56705	2.197225	1	1	2.564949	C41	2017
2.079442	0.375	13.73484	2.302585	1	1	2.639057	C41	2018
2.197225	0.333333	13.74337	2.397895	1	1	2.70805	C41	2019
2.197225	0.333333	13.98736	2.397895	1	3	2.890372	C26	2014
2.197225	0.333333	14.3207	2.70805	1	3	2.944439	C26	2015
2.079442	0.375	14.79725	2.397895	1	3	2.995732	C26	2016
2.197225	0.333333	14.8467	1.94591	1	3	3.044522	C26	2017
2.397895	0.363636	14.7962	2.197225	1	3	3.091042	C26	2018
2.397895	0.363636	15.04123	2.197225	1	3	3.135494	C26	2019
2.197225	0.333333	14.46136	2.197225	1	2	2.197225	C27	2014
2.197225	0.333333	14.62822	2.564949	1	2	2.302585	C27	2015
2.197225	0.333333	14.44476	1.94591	1	2	2.397895	C27	2016
2.197225	0.333333	14.75552	1.098612	1	2	2.484907	C27	2017
2.079442	0.375	14.45947	1.94591	1	2	2.564949	C27	2018
2.197225	0.333333	14.40635	2.079442	1	2	2.639057	C27	2019
1.609438	0.4	16.07971	2.639057	1	3	2.302585	R86	2015
1.609438	0.4	16.83384	2.302585	1	3	2.397895	R86	2016
1.791759	0.333333	16.8987	1.791759	1	3	2.484907	R86	2017
1.791759	0.333333	16.84487	2.197225	1	3	2.564949	R86	2018
2.197225	0.333333	14.26659	2.302585	1	4	3.555348	C26	2015
2.197225	0.333333	13.99005	2.302585	1	2	2.564949	C30	2015
2.197225	0.333333	14.03143	2.639057	1	2	2.639057	C30	2016
2.197225	0.333333	14.66292	2.197225	1	2	2.70805	C30	2017
2.197225	0.333333	14.75677	1.609438	1	2	2.772589	C30	2018
2.197225	0.333333	14.38542	2.197225	1	2	2.833213	C30	2019
1.94591	0.428571	13.12136	2.302585	1	2	2.833213	A03	2018
2.197225	0.333333	14.13171	1.94591	1	2	2.70805	C26	2015

2.197225	0.333333	13.92964	1.609438	1	2	2.772589	C26	2016
2.197225	0.333333	14.09519	1.609438	1	2	2.833213	C26	2017
2.197225	0.444444	14.39196	2.397895	1	2	2.944439	C26	2019
2.079442	0.375	14.32774	1.609438	1	4	2.890372	C27	2014
2.197225	0.333333	14.46726	1.791759	1	4	2.944439	C27	2015
2.197225	0.333333	14.47028	1.94591	1	4	2.995732	C27	2016
2.197225	0.333333	14.52537	1.94591	1	4	3.044522	C27	2017
2.197225	0.333333	14.51379	1.94591	1	4	3.091042	C27	2018
2.197225	0.333333	14.49075	1.791759	1	4	3.135494	C27	2019
2.079442	0.375	13.80243	2.484907	1	2	2.397895	C21	2015
2.197225	0.333333	14.31143	2.197225	1	2	2.484907	C21	2016
2.197225	0.333333	14.39308	2.397895	1	2	2.564949	C21	2017
2.197225	0.333333	14.27597	2.079442	1	2	2.639057	C21	2018
2.197225	0.333333	14.44529	2.302585	1	2	2.70805	C21	2019
2.197225	0.333333	13.67774	2.079442	1	2	2.890372	C33	2015
2.197225	0.333333	13.84633	2.079442	1	2	2.944439	C33	2016
2.197225	0.333333	14.25312	2.397895	1	2	2.995732	C33	2017
2.197225	0.333333	14.57501	1.94591	1	2	3.044522	C33	2018
2.079442	0.375	14.34496	1.791759	1	2	3.091042	C18	2018
1.94591	0.428571	14.70677	2.079442	1	2	3.135494	C18	2019
2.197225	0.333333	15.46378	2.197225	1	3	2.079442	C18	2015
2.079442	0.375	15.45101	1.609438	1	3	2.197225	C18	2016
1.94591	0.428571	15.63644	1.791759	1	3	2.302585	C18	2017
1.94591	0.428571	15.89979	1.791759	1	3	2.397895	C18	2018
1.94591	0.428571	16.15589	1.609438	1	3	2.484907	C18	2019
2.197225	0.333333	13.67648	2.079442	1	4	2.944439	C36	2015
2.197225	0.333333	13.71922	2.564949	1	3	2.995732	C36	2016
2.197225	0.333333	13.88634	2.639057	1	4	3.044522	C36	2017
2.197225	0.333333	14.28107	2.70805	1	3	3.091042	C36	2018
2.197225	0.333333	14.51732	2.302585	1	4	3.135494	C36	2019
1.94591	0.428571	13.90169	1.791759	1	3	3.178054	C40	2015
1.94591	0.428571	13.88214	2.079442	1	3	3.218876	C40	2016
1.94591	0.428571	14.03062	1.791759	1	3	3.258097	C40	2017
1.94591	0.428571	13.98946	2.079442	1	3	3.295837	C40	2018
1.94591	0.428571	13.89247	1.791759	1	3	3.332205	C40	2019
2.197225	0.333333	15.28846	1.94591	1	3	2.944439	C27	2015
2.197225	0.333333	15.31422	2.564949	1	3	2.995732	C27	2016
2.197225	0.333333	15.64939	2.302585	1	3	3.044522	C27	2017
2.197225	0.333333	15.69796	1.609438	1	3	3.091042	C27	2018
2.197225	0.333333	15.95695	2.079442	1	3	3.135494	C27	2019
1.609438	0.4	14.12498	2.302585	1	2	2.995732	C34	2018
1.609438	0.4	14.25222	2.397895	1	2	3.044522	C34	2019
1.94591	0.428571	13.565	1.94591	1	3	2.564949	M74	2019
2.197225	0.333333	13.73863	1.94591	1	4	2.995732	C25	2018
1.94591	0.428571	13.85396	1.791759	1	4	3.044522	C25	2019
2.197225	0.333333	14.18604	2.484907	1	2	2.995732	E50	2015
1.94591	0.428571	14.36045	2.70805	1	2	3.044522	E50	2016
1.94591	0.428571	14.27021	3.218876	1	2	3.091042	E50	2017
1.94591	0.428571	14.28044	3.218876	1	2	3.135494	E50	2018
1.94591	0.428571	14.24664	3.135494	1	2	3.178054	E50	2019
1.94591	0.428571	13.8109	1.386294	1	4	2.833213	C33	2015
1.791759	0.5	14.42528	2.302585	1	3	2.890372	C33	2016
1.791759	0.5	14.33817	2.639057	1	4	2.944439	C33	2017
2.079442	0.375	14.00431	2.564949	1	3	2.995732	C33	2018
2.079442	0.375	14.00638	2.079442	1	4	3.044522	C33	2019
1.791759	0.333333	13.71059	2.302585	1	2	3.044522	E50	2015
1.791759	0.333333	13.94285	2.197225	1	2	3.091042	E50	2016
1.791759	0.333333	14.02244	2.302585	1	2	3.135494	E50	2017

1.791759	0.333333	14.07054	2.772589	1	2	3.178054	E50	2018
1.791759	0.333333	14.212	2.397895	1	2	3.218876	E50	2019
1.94591	0.428571	13.80192	1.94591	1	4	2.890372	C34	2016
1.94591	0.428571	13.78588	1.94591	1	4	2.944439	C34	2017
1.94591	0.428571	13.80828	2.079442	1	4	2.995732	C34	2018
2.079442	0.375	13.88186	1.609438	1	3	3.258097	C33	2016
2.079442	0.375	13.98584	2.397895	1	3	3.295837	C33	2017
1.609438	0.4	14.15034	2.397895	1	3	3.332205	C33	2018
1.609438	0.4	14.24658	2.079442	1	3	3.367296	C33	2019
2.484907	0.333333	13.96806	2.484907	1	3	3.178054	C21	2018
2.197225	0.333333	13.36107	2.197225	1	3	3.218876	C21	2019
2.197225	0.333333	13.81501	1.94591	1	4	1.94591	C38	2016
2.197225	0.333333	13.73863	1.94591	1	4	2.079442	C38	2017
2.197225	0.333333	13.81751	2.197225	1	4	2.197225	C38	2018
2.197225	0.333333	14.02252	1.791759	1	4	2.302585	C38	2019
2.197225	0.333333	14.55983	1.791759	1	3	2.397895	C26	2016
2.079442	0.375	14.58992	2.70805	1	3	2.484907	C26	2017
2.079442	0.375	14.64104	2.197225	1	3	2.564949	C26	2018
2.079442	0.375	15.57777	2.639057	1	3	2.639057	C26	2019
1.94591	0.428571	13.83825	2.079442	0	2	3.178054	C26	2016
1.94591	0.428571	13.85175	1.791759	0	2	3.218876	C26	2017
1.94591	0.428571	14.21637	1.386294	0	2	3.258097	C26	2018
1.94591	0.428571	14.65945	1.791759	0	3	3.295837	C26	2019
2.079442	0.375	14.36045	1.791759	1	4	2.302585	C29	2016
2.197225	0.333333	14.23416	2.079442	1	4	2.397895	C29	2017
2.197225	0.333333	14.80835	2.772589	1	4	2.484907	C29	2018
2.197225	0.333333	14.43636	2.772589	1	4	2.564949	C29	2019
1.94591	0.428571	12.94682	1.94591	1	1	2.995732	C27	2016
1.94591	0.428571	13.23426	1.791759	1	1	3.044522	C27	2017
1.94591	0.428571	13.75576	1.791759	1	1	3.091042	C27	2018
1.94591	0.428571	14.23757	1.386294	1	2	3.135494	C27	2019
1.609438	0.4	14.19367	1.791759	1	4	3.091042	E50	2016
1.609438	0.4	14.50012	1.94591	1	4	3.135494	E50	2017
1.609438	0.4	14.52305	1.94591	1	4	3.178054	E50	2018
1.609438	0.4	14.56101	2.197225	1	4	3.218876	E50	2019
1.94591	0.428571	13.48283	1.791759	1	3	2.944439	C32	2016
1.94591	0.428571	13.37359	2.197225	1	3	2.995732	C32	2017
1.94591	0.428571	13.55415	2.484907	1	4	3.044522	C32	2018
1.94591	0.428571	13.55415	1.94591	1	4	3.091042	C32	2019
1.94591	0.428571	13.40075	2.079442	0	3	2.397895	C33	2017
1.94591	0.428571	13.28261	2.302585	0	4	2.484907	C33	2018
1.94591	0.428571	14.13904	2.564949	0	4	2.564949	C33	2019
2.197225	0.333333	15.18923	2.302585	1	2	2.302585	C39	2017
2.197225	0.333333	15.06973	2.302585	1	2	2.397895	C39	2018
2.197225	0.333333	15.22801	2.302585	1	2	2.484907	C39	2019
1.94591	0.428571	14.14258	2.484907	1	3	3.496508	E50	2017
1.94591	0.428571	14.19593	2.079442	1	3	3.526361	E50	2018
1.94591	0.428571	14.04344	2.197225	1	3	3.555348	E50	2019
2.197225	0.333333	13.71015	1.94591	1	4	2.70805	R88	2017
2.197225	0.333333	13.64116	2.397895	1	4	2.772589	R88	2018
2.079442	0.375	13.63399	2.079442	1	4	2.833213	R88	2019
1.94591	0.428571	14.24306	2.079442	1	4	2.772589	C39	2017
1.94591	0.428571	14.3416	2.079442	1	4	2.833213	C39	2018
1.94591	0.428571	14.31871	2.302585	1	4	2.890372	C39	2019
1.94591	0.428571	14.65202	2.197225	1	2	2.995732	C38	2017
1.94591	0.428571	14.00671	1.791759	1	2	3.044522	C38	2018
1.94591	0.428571	14.94407	2.197225	1	2	3.091042	C38	2019
2.302585	0.4	14.80118	1.94591	1	3	2.302585	F52	2017

2.397895	0.363636	14.95568	2.397895	1	1	2.397895	F52	2018
2.397895	0.363636	14.91645	1.94591	1	3	2.484907	F52	2019
1.94591	0.428571	15.20632	2.484907	0	4	2.564949	C39	2017
1.791759	0.5	15.36399	2.484907	0	3	2.639057	C39	2018
2.397895	0.363636	14.43555	2.197225	1	4	2.772589	F51	2017
2.197225	0.444444	13.69718	2.772589	1	4	2.833213	F51	2018
2.197225	0.333333	13.77469	2.302585	1	3	3.091042	C27	2017
2.197225	0.333333	13.96393	2.079442	1	4	3.135494	C27	2018
2.197225	0.333333	14.16743	2.397895	1	3	3.178054	C27	2019
1.94591	0.428571	14.78002	2.197225	1	3	2.944439	L72	2017
1.94591	0.428571	14.95354	2.302585	1	3	2.995732	L72	2018
1.94591	0.428571	14.93052	2.079442	1	3	3.044522	L72	2019
2.079442	0.375	14.45283	2.079442	1	3	2.995732	C38	2017
2.079442	0.375	14.42517	1.609438	1	3	3.044522	C38	2018
2.079442	0.375	14.51692	1.609438	1	3	3.091042	C38	2019
2.197225	0.333333	13.64863	2.079442	1	1	3.044522	C38	2017
2.197225	0.333333	14.06268	2.484907	1	1	3.091042	C38	2018
2.197225	0.333333	14.30636	2.079442	0	3	3.091042	C39	2018
2.197225	0.333333	14.31762	2.197225	0	4	3.135494	C39	2019
1.94591	0.428571	13.93169	2.197225	1	2	3.044522	C39	2018
1.94591	0.428571	13.94452	2.197225	1	4	3.091042	C39	2019
1.609438	0.4	14.54016	2.079442	1	3	1.94591	C38	2017
1.609438	0.4	14.23369	1.94591	1	3	2.079442	C38	2018
1.609438	0.4	14.50716	2.079442	1	3	2.197225	C38	2019
2.197225	0.333333	14.11776	2.079442	1	2	3.178054	C39	2019
2.197225	0.333333	14.07787	2.079442	1	3	3.044522	C27	2017
2.197225	0.333333	14.24729	2.302585	1	3	3.091042	C27	2018
2.197225	0.333333	14.67417	2.079442	1	3	3.135494	C27	2019
1.94591	0.428571	14.08416	1.386294	1	2	2.564949	C34	2017
1.94591	0.428571	14.04153	1.94591	1	2	2.639057	C34	2018
1.94591	0.428571	13.60664	1.386294	1	2	2.70805	C34	2019
2.197225	0.333333	14.54778	2.197225	1	4	2.564949	R86	2017
2.197225	0.333333	14.54759	1.609438	1	4	2.639057	R86	2018
2.197225	0.333333	14.67962	1.94591	1	4	2.70805	R86	2019
2.079442	0.375	14.82303	1.609438	1	4	3.135494	C26	2017
2.079442	0.375	14.82729	2.197225	1	4	3.178054	C26	2018
2.079442	0.375	14.67747	1.94591	1	3	3.218876	C26	2019
2.197225	0.333333	15.29716	2.564949	1	3	2.944439	C30	2017
2.197225	0.333333	15.73299	2.70805	1	2	2.995732	C30	2018
2.197225	0.333333	16.0861	2.484907	1	2	3.044522	C30	2019
2.197225	0.333333	13.9038	2.197225	0	3	2.70805	I65	2018
2.197225	0.333333	13.71026	2.197225	0	3	2.772589	I65	2019
2.197225	0.333333	14.85667	1.94591	1	2	3.044522	C37	2018
2.197225	0.333333	14.98225	2.639057	1	2	3.091042	C37	2019
2.197225	0.333333	13.90782	1.791759	1	4	2.944439	C26	2018
2.197225	0.333333	14.25853	2.197225	1	4	2.995732	C26	2019
2.079442	0.375	13.4651	2.302585	1	2	2.995732	C34	2018
2.079442	0.375	13.21367	1.94591	1	2	3.044522	C34	2019
2.197225	0.444444	15.38635	1.94591	1	4	3.218876	M74	2018
2.197225	0.444444	15.38818	2.079442	1	4	3.258097	M74	2019
1.94591	0.428571	14.51145	2.564949	1	4	1.94591	C39	2019
1.609438	0.4	13.70982	2.079442	1	1	2.302585	C38	2019
1.609438	0.4	14.42647	1.791759	0	1	2.890372	C14	2019
2.197225	0.333333	15.13727	2.079442	1	3	2.944439	E50	2019
1.94591	0.428571	14.88617	2.772589	0	1	2.890372	N78	2019
2.197225	0.333333	12.72487	2.397895	1	3	2.302585	C35	2009
2.197225	0.333333	12.72487	2.302585	1	3	2.397895	C35	2010
2.197225	0.333333	13.21767	2.397895	1	3	2.484907	C35	2011

2.197225	0.333333	13.61706	1.791759	1	3	2.564949	C34	2012
2.197225	0.333333	13.61706	2.079442	1	3	2.639057	C34	2013
2.079442	0.375	13.44922	2.397895	1	3	2.70805	C34	2014
2.079442	0.375	14.5195	1.791759	1	3	2.772589	C34	2015
2.079442	0.375	14.41819	1.94591	1	3	2.833213	C34	2016
2.197225	0.333333	14.29	1.94591	1	3	2.890372	C34	2017
2.197225	0.333333	14.21643	1.94591	1	3	2.995732	C34	2019
2.079442	0.375	13.94346	2.079442	1	4	1.791759	M74	2009
2.079442	0.375	14.42021	2.302585	1	4	1.94591	M74	2010
2.079442	0.375	14.20288	1.94591	1	4	2.079442	M74	2011
2.079442	0.375	14.63701	2.397895	1	4	2.197225	M74	2012
2.197225	0.333333	14.91032	2.302585	1	4	2.302585	M74	2013
2.197225	0.333333	15.42739	2.302585	1	1	2.397895	M74	2014
2.197225	0.333333	15.69088	1.94591	1	1	2.484907	M74	2015
1.94591	0.428571	15.22835	2.484907	1	1	2.564949	M74	2016
1.94591	0.428571	14.85752	1.791759	1	4	2.639057	M74	2017
1.94591	0.428571	15.22244	2.564949	1	4	2.772589	M74	2019
2.197225	0.333333	14.67629	2.772589	1	3	3.044522	G59	2018
2.197225	0.333333	13.80748	2.079442	1	1	2.833213	C27	2009
2.197225	0.333333	13.93773	1.791759	1	1	2.890372	C27	2010
2.197225	0.333333	14.43318	1.386294	1	1	2.944439	C27	2011
2.197225	0.333333	14.79965	1.609438	1	1	2.995732	C27	2012
2.197225	0.333333	14.93173	2.079442	1	1	3.044522	C27	2013
2.197225	0.333333	14.7516	2.079442	1	1	3.091042	C27	2014
2.197225	0.333333	14.95973	2.197225	1	1	3.135494	C27	2015
2.197225	0.333333	14.75513	1.94591	1	1	3.178054	C27	2016
2.197225	0.333333	14.66275	1.791759	1	1	3.218876	C27	2017
2.197225	0.333333	14.74704	2.079442	1	1	3.258097	C27	2018
2.197225	0.333333	13.73971	2.079442	0	3	2.197225	I63	2009
2.197225	0.444444	13.98652	1.94591	0	3	2.302585	I63	2010
2.197225	0.333333	14.25176	1.94591	0	3	2.397895	I63	2011
2.197225	0.333333	14.6936	2.197225	0	3	2.484907	I65	2012
2.197225	0.333333	14.81945	2.079442	1	3	2.564949	I65	2013
1.94591	0.428571	15.11695	2.302585	1	3	2.772589	I65	2016
1.94591	0.428571	15.47383	2.944439	1	3	2.833213	I65	2017
2.397895	0.363636	14.48462	2.197225	1	5	2.079442	C38	2009
2.397895	0.363636	14.21429	1.791759	1	5	2.197225	C38	2010
1.609438	0.4	14.12277	2.079442	1	5	2.302585	C38	2011
1.609438	0.4	14.10219	1.94591	1	5	2.397895	C38	2012
1.609438	0.4	13.95405	1.94591	1	5	2.484907	C38	2013
1.609438	0.4	14.18293	2.079442	1	4	2.564949	C38	2014
1.609438	0.4	14.20809	2.079442	1	4	2.639057	C38	2015
1.609438	0.4	14.25131	1.791759	1	4	2.70805	C38	2016
1.609438	0.4	14.27805	2.397895	1	4	2.772589	C38	2017
2.197225	0.333333	14.41545	2.302585	1	3	2.944439	C26	2017
2.197225	0.333333	14.41545	1.94591	1	4	2.995732	C26	2018
2.197225	0.333333	14.53763	2.079442	1	4	3.044522	C26	2019
2.197225	0.333333	12.03172	2.302585	1	4	1.386294	C35	2009
2.197225	0.333333	12.83575	2.302585	1	4	1.609438	C35	2010
2.197225	0.333333	13.00628	2.079442	1	4	1.791759	C35	2011
2.197225	0.333333	12.96045	1.791759	1	4	1.94591	C29	2012
2.197225	0.333333	13.01433	2.079442	1	4	2.079442	C29	2013
2.197225	0.333333	13.22058	2.079442	1	4	2.197225	C29	2014
2.197225	0.333333	13.26282	2.302585	1	4	2.302585	C29	2015
2.079442	0.375	13.32661	2.484907	1	4	2.397895	C29	2016
2.197225	0.333333	13.8623	2.484907	1	3	2.484907	C29	2017
2.197225	0.333333	14.4095	2.70805	1	3	2.564949	C29	2018
2.197225	0.333333	14.5648	2.302585	1	3	2.639057	C29	2019

2.197225	0.333333	15.49105	2.302585	1	4	2.639057	R86	2018
1.609438	0.4	13.04721	2.079442	1	3	2.944439	C39	2018
2.197225	0.333333	14.11769	2.197225	0	1	0.693147	C35	2010
2.302585	0.4	13.96393	1.94591	0	1	1.098612	C35	2011
2.079442	0.5	13.85184	2.302585	0	3	1.791759	C35	2014
2.197225	0.333333	13.64116	2.197225	1	2	0.693147	C39	2010
2.197225	0.333333	13.64116	2.197225	1	2	1.098612	C39	2011
2.197225	0.333333	13.64116	2.197225	1	2	1.386294	C38	2012
2.197225	0.333333	13.64116	1.609438	1	2	1.609438	C38	2013
2.197225	0.333333	13.64116	2.079442	1	2	1.791759	C38	2014
2.197225	0.333333	13.66469	2.484907	1	2	1.94591	C38	2015
1.94591	0.428571	13.64116	2.484907	1	2	2.079442	C38	2016
1.94591	0.428571	13.64116	2.302585	1	2	2.197225	C38	2017
2.197225	0.333333	13.88317	2.302585	1	3	2.772589	C26	2014
2.197225	0.333333	14.09941	2.484907	1	4	2.833213	C26	2015
2.197225	0.333333	13.87708	1.791759	1	4	2.890372	C26	2016
2.197225	0.333333	14.13244	1.94591	1	4	2.944439	C26	2017
2.197225	0.333333	14.10609	1.94591	1	4	2.995732	C26	2018
2.197225	0.333333	14.21462	1.791759	1	2	3.044522	C26	2019
1.94591	0.428571	12.69863	2.197225	0	1	2.302585	C24	2010
1.94591	0.428571	12.91189	2.302585	0	1	2.397895	C24	2011
1.94591	0.428571	13.00852	1.94591	0	1	2.484907	C24	2012
1.94591	0.428571	13.17477	2.564949	1	1	2.564949	C24	2013
1.94591	0.428571	13.4	2.564949	1	4	2.639057	C24	2014
1.94591	0.428571	13.5198	2.564949	1	4	2.70805	C24	2015
1.94591	0.428571	13.46453	2.397895	1	4	2.772589	C24	2016
1.94591	0.428571	14.5883	2.70805	1	4	2.833213	I64	2017
1.94591	0.428571	14.59822	2.197225	1	1	2.890372	I64	2018
1.94591	0.428571	14.66292	1.609438	1	4	2.944439	I64	2019
2.197225	0.333333	13.00897	2.397895	1	1	1.791759	C40	2010
2.197225	0.333333	13.2269	2.397895	1	1	1.94591	C40	2011
2.197225	0.333333	13.28686	1.94591	1	1	2.079442	C40	2012
2.197225	0.333333	13.37359	2.197225	1	1	2.197225	C40	2013
2.197225	0.333333	13.52636	2.079442	1	2	2.302585	C40	2014
2.197225	0.333333	13.70167	2.564949	1	2	2.397895	C40	2015
2.197225	0.333333	13.75576	2.197225	1	1	2.484907	C40	2016
2.197225	0.333333	13.80273	2.079442	1	1	2.564949	C40	2017
2.197225	0.333333	13.77604	2.397895	1	1	2.639057	C40	2018
2.197225	0.333333	14.29787	2.302585	1	2	2.70805	C40	2019
1.609438	0.6	12.68788	2.197225	1	4	1.098612	C38	2010
1.609438	0.6	13.18101	1.94591	1	4	1.386294	C38	2011
1.609438	0.6	13.16158	1.94591	1	4	1.609438	C38	2012
1.609438	0.6	12.84635	1.94591	1	4	1.791759	C38	2013
1.609438	0.6	13.12236	1.609438	1	4	1.94591	C38	2014
1.609438	0.6	13.33893	1.609438	1	4	2.079442	C38	2015
1.609438	0.6	13.2616	2.302585	1	4	2.197225	C38	2016
1.94591	0.428571	13.79101	2.302585	1	4	2.302585	C38	2017
1.791759	0.5	14.17319	1.609438	1	4	2.484907	C38	2019
2.197225	0.333333	13.56242	1.94591	1	3	1.94591	I63	2010
2.197225	0.333333	13.4931	2.397895	0	3	2.079442	I63	2011
2.197225	0.333333	13.58004	1.94591	0	3	2.197225	I65	2012
2.079442	0.375	13.82348	1.791759	0	3	2.302585	I65	2013
2.197225	0.333333	14.06081	1.94591	0	3	2.397895	I65	2014
2.197225	0.333333	13.9929	2.079442	0	3	2.564949	I65	2016
2.197225	0.333333	14.24274	1.94591	0	2	2.639057	I65	2017
2.197225	0.333333	14.61877	2.397895	1	2	3.091042	C39	2014
2.197225	0.333333	14.54778	2.197225	1	2	3.135494	C39	2015
2.197225	0.333333	14.60397	2.197225	1	2	3.178054	C39	2016

2.197225	0.333333	14.45736	2.079442	1	2	3.258097	C39	2018
1.94591	0.428571	14.30446	1.386294	1	2	0.693147	C39	2010
1.94591	0.428571	14.5574	2.079442	1	2	1.098612	C39	2011
1.94591	0.428571	14.56546	2.079442	1	2	1.386294	C39	2012
1.94591	0.428571	14.71653	2.079442	1	2	1.609438	C39	2013
1.94591	0.428571	14.56881	2.397895	1	2	1.791759	C39	2014
1.94591	0.428571	14.69098	2.302585	1	2	1.94591	C39	2015
2.197225	0.333333	14.60087	2.197225	1	2	2.079442	C39	2016
2.197225	0.333333	14.60891	2.397895	1	2	2.197225	C39	2017
2.197225	0.333333	12.46844	1.791759	1	3	2.197225	C34	2010
2.197225	0.333333	12.46844	2.197225	1	3	2.302585	C34	2011
1.94591	0.428571	12.56375	2.397895	1	3	2.397895	C35	2012
2.197225	0.333333	12.87927	2.302585	1	3	2.484907	C35	2013
2.197225	0.333333	13.14823	2.302585	1	3	2.564949	C35	2014
2.197225	0.333333	13.29564	2.302585	1	3	2.639057	C35	2015
2.197225	0.333333	13.27285	2.197225	1	3	2.70805	C35	2016
2.197225	0.333333	13.49558	2.197225	1	3	2.772589	C35	2017
2.197225	0.333333	13.5535	2.944439	1	3	2.833213	C35	2018
2.197225	0.333333	13.66829	2.079442	0	3	2.890372	C35	2019
2.197225	0.333333	13.18665	1.791759	1	1	1.386294	C36	2010
2.197225	0.333333	12.76569	2.079442	1	1	1.609438	C36	2011
2.197225	0.333333	13.30468	1.94591	1	1	1.791759	C36	2012
2.197225	0.333333	13.3587	2.302585	1	4	1.94591	C36	2013
2.197225	0.333333	13.54107	2.772589	1	4	2.079442	C36	2014
2.197225	0.333333	13.50354	2.302585	1	3	2.197225	C36	2015
2.197225	0.333333	14.18029	2.639057	1	3	2.302585	C36	2016
2.197225	0.333333	14.86752	2.833213	1	3	2.397895	C36	2017
2.197225	0.333333	14.76574	2.397895	1	3	2.484907	C36	2018
2.197225	0.333333	13.48422	2.397895	1	2	2.484907	C35	2014
2.197225	0.444444	13.93684	1.386294	1	2	2.564949	C35	2015
2.079442	0.375	14.04853	1.609438	1	2	2.639057	C35	2016
2.197225	0.444444	14.02836	2.079442	1	2	2.70805	C35	2017
2.197225	0.444444	14.00836	2.397895	1	3	2.772589	C35	2018
2.197225	0.444444	13.83629	2.564949	1	3	2.833213	N77	2019
2.197225	0.333333	12.87313	2.079442	1	3	1.94591	C26	2010
2.079442	0.375	12.67888	2.079442	1	3	2.079442	C26	2011
2.079442	0.375	12.75996	1.609438	1	3	2.197225	C26	2012
2.197225	0.333333	13.11594	2.397895	1	3	2.302585	C26	2013
2.197225	0.333333	13.20781	2.302585	1	3	2.397895	C26	2014
2.197225	0.333333	13.29967	1.94591	1	3	2.484907	C26	2015
2.197225	0.333333	13.33068	2.079442	1	3	2.564949	C26	2016
2.197225	0.333333	13.80327	1.791759	1	3	2.639057	C26	2017
2.197225	0.333333	14.37832	2.079442	1	3	2.772589	C26	2019
1.94591	0.428571	12.6346	2.397895	1	4	1.94591	C26	2010
1.94591	0.428571	13.06091	1.791759	1	4	2.079442	C26	2011
1.94591	0.428571	13.18853	1.94591	1	4	2.197225	C26	2012
1.791759	0.5	13.11272	1.609438	1	4	2.302585	C26	2013
1.791759	0.5	13.12056	2.079442	1	4	2.397895	C26	2014
1.791759	0.5	13.12795	2.197225	1	4	2.484907	C26	2015
1.791759	0.5	13.00987	2.70805	1	4	2.564949	C26	2016
1.94591	0.428571	13.17647	2.397895	1	1	2.639057	C26	2017
1.791759	0.5	13.49544	2.70805	1	1	2.70805	C26	2018
1.791759	0.5	13.84507	2.70805	1	4	2.772589	C26	2019
2.397895	0.363636	14.13759	1.386294	1	4	2.197225	C27	2010
2.397895	0.363636	14.32233	2.302585	1	4	2.302585	C27	2011
2.397895	0.363636	14.30935	2.397895	1	4	2.397895	C27	2012
2.397895	0.454545	14.20417	2.397895	1	4	2.484907	C27	2013
2.197225	0.333333	13.61266	2.079442	1	4	2.564949	C27	2014

2.197225	0.333333	14.02147	2.197225	1	4	2.639057	C27	2015
1.94591	0.428571	14.18914	2.197225	1	3	3.433987	E50	2018
1.94591	0.428571	14.26729	2.397895	1	3	3.465736	E50	2019
2.197225	0.333333	14.27256	2.079442	0	1	0.693147	C39	2011
2.197225	0.333333	13.90178	2.639057	0	1	1.386294	C39	2013
2.197225	0.333333	13.0431	2.484907	1	3	2.397895	C38	2010
2.079442	0.375	12.61487	2.484907	1	3	2.484907	C38	2011
2.197225	0.333333	13.58522	1.94591	1	3	2.564949	C38	2012
2.197225	0.333333	13.42602	2.079442	1	3	2.639057	C38	2013
1.94591	0.428571	13.17723	2.197225	1	3	2.70805	C38	2014
1.94591	0.428571	13.10359	1.94591	1	3	2.772589	C38	2015
1.94591	0.428571	13.13686	2.302585	1	3	2.833213	C38	2016
2.197225	0.333333	13.06367	1.94591	1	3	2.890372	C38	2017
2.197225	0.333333	15.15351	2.639057	1	3	2.944439	C39	2018
2.197225	0.333333	15.28513	2.484907	1	3	2.995732	C39	2019
2.197225	0.333333	13.46737	2.079442	1	4	2.70805	C27	2010
2.197225	0.333333	13.51007	2.833213	1	4	2.772589	C27	2011
2.197225	0.333333	13.89247	2.564949	1	4	2.833213	C27	2012
2.197225	0.333333	13.89247	2.397895	1	4	2.890372	C27	2013
2.197225	0.333333	14.31629	1.94591	1	2	2.944439	C27	2014
1.94591	0.428571	14.31629	2.302585	1	4	2.995732	C27	2015
1.791759	0.5	14.22098	2.197225	1	4	3.044522	C27	2016
1.94591	0.428571	14.25724	2.197225	1	3	3.091042	C27	2017
2.197225	0.333333	13.19189	1.94591	1	3	1.94591	C37	2010
2.197225	0.333333	13.56512	1.94591	1	3	2.079442	C37	2011
2.197225	0.333333	13.87566	2.079442	1	3	2.197225	C37	2012
2.079442	0.375	13.94917	2.302585	1	3	2.302585	C37	2013
2.079442	0.375	14.04257	2.397895	1	3	2.397895	C37	2014
1.791759	0.5	13.91536	2.397895	1	3	2.484907	C37	2015
1.791759	0.5	14.16441	2.397895	1	3	2.564949	C37	2016
1.791759	0.5	14.12204	2.302585	1	3	2.639057	C37	2017
1.791759	0.5	14.09458	2.302585	1	3	2.70805	C37	2018
2.079442	0.375	14.01264	2.079442	1	3	2.772589	C37	2019
2.397895	0.363636	14.17003	2.302585	1	2	2.197225	C34	2010
2.397895	0.363636	14.15555	2.397895	1	2	2.302585	C34	2011
2.397895	0.363636	14.17807	2.197225	1	2	2.397895	C38	2012
2.397895	0.363636	14.17807	2.079442	1	2	2.484907	C38	2013
2.397895	0.363636	14.17807	2.197225	1	1	2.564949	C38	2014
2.397895	0.363636	14.17807	2.639057	1	1	2.639057	C38	2015
2.397895	0.363636	14.17807	2.079442	1	1	2.70805	C38	2016
2.564949	0.384615	14.03062	1.791759	1	1	2.772589	C38	2017
2.564949	0.384615	14.2241	2.397895	1	1	2.833213	C38	2018
2.397895	0.454545	14.23888	1.94591	1	1	2.890372	C38	2019
2.197225	0.333333	14.5497	1.609438	1	2	2.772589	C26	2017
1.94591	0.428571	14.09932	2.197225	1	2	1.609438	R86	2010
1.94591	0.428571	14.32678	2.397895	1	2	1.791759	R86	2011
1.94591	0.428571	14.72809	2.397895	1	2	1.94591	R86	2012
1.94591	0.428571	14.36917	2.639057	1	2	2.079442	R86	2013
1.94591	0.428571	14.5806	2.484907	1	2	2.197225	R86	2014
1.94591	0.428571	14.74369	2.564949	1	2	2.302585	R86	2015
1.94591	0.428571	14.55664	2.564949	1	2	2.397895	R86	2016
1.94591	0.428571	14.62479	2.197225	1	2	2.484907	R86	2017
1.94591	0.428571	14.92027	2.302585	1	2	2.564949	R86	2018
2.197225	0.333333	13.35791	2.302585	1	2	2.639057	C26	2016
2.197225	0.333333	13.30801	1.791759	1	2	2.70805	C26	2017
2.197225	0.333333	13.6043	1.791759	1	1	2.772589	C26	2018
1.94591	0.428571	13.72919	2.302585	1	2	2.833213	C26	2019
1.94591	0.428571	12.77592	1.791759	1	2	2.484907	C38	2010

1.94591	0.428571	12.84265	1.791759	1	2	2.564949	C38	2011
1.94591	0.428571	12.96604	2.197225	1	2	2.639057	C38	2012
1.94591	0.428571	13.01322	1.791759	1	2	2.70805	C38	2013
1.94591	0.428571	13.1904	2.079442	1	2	2.772589	C38	2014
1.94591	0.428571	13.35316	1.94591	1	2	2.833213	C38	2015
1.94591	0.428571	13.88718	2.302585	1	2	2.890372	C38	2016
1.94591	0.428571	13.9454	2.302585	1	2	2.944439	C38	2017
1.94591	0.428571	13.90745	1.94591	1	2	2.995732	C38	2018
1.94591	0.428571	13.90049	2.079442	1	2	3.044522	C38	2019
1.94591	0.428571	13.24334	1.609438	1	2	2.944439	C34	2010
1.94591	0.428571	13.31464	2.302585	1	4	2.995732	C34	2011
1.94591	0.428571	13.66956	2.197225	1	2	3.044522	C34	2012
1.94591	0.428571	13.74884	2.197225	1	4	3.091042	C34	2013
1.94591	0.428571	14.14107	2.079442	1	2	3.135494	C34	2014
2.197225	0.333333	13.57408	2.197225	1	4	3.178054	C34	2015
2.197225	0.333333	14.27761	2.197225	1	2	3.218876	C34	2016
2.197225	0.333333	14.12703	2.772589	1	4	3.258097	C34	2017
1.609438	0.4	14.30151	1.94591	1	4	1.609438	C14	2010
1.94591	0.428571	14.72928	2.197225	1	4	1.791759	C14	2011
1.94591	0.428571	14.52108	2.079442	1	4	1.94591	C14	2012
1.94591	0.428571	14.52797	2.484907	1	4	2.079442	C14	2013
1.94591	0.428571	14.51265	2.639057	1	4	2.197225	C14	2014
1.94591	0.428571	14.99417	2.302585	1	4	2.302585	C14	2015
1.94591	0.428571	15.6318	2.302585	1	4	2.397895	C14	2016
1.94591	0.428571	16.19875	2.484907	1	4	2.484907	C14	2017
1.94591	0.428571	16.48394	2.772589	1	4	2.564949	C14	2018
1.94591	0.428571	16.33376	2.484907	1	4	2.639057	C14	2019
1.609438	0.4	13.1558	1.609438	1	2	1.94591	R85	2010
1.609438	0.4	13.52783	2.484907	1	2	2.079442	R85	2011
1.609438	0.4	13.78144	1.609438	1	2	2.197225	R85	2012
1.609438	0.4	13.83531	1.94591	1	2	2.302585	R85	2013
1.609438	0.4	13.71015	2.197225	1	2	2.397895	R85	2014
1.609438	0.4	14.16617	2.397895	1	2	2.484907	R85	2015
1.609438	0.4	14.36363	2.302585	1	2	2.564949	R85	2016
1.609438	0.4	14.50866	2.639057	1	2	2.639057	R85	2017
1.609438	0.4	14.59024	2.302585	1	2	2.772589	R85	2019
2.197225	0.333333	13.68768	1.609438	1	1	2.197225	C30	2010
2.197225	0.333333	13.68768	1.94591	1	1	2.302585	C30	2011
2.197225	0.333333	14.1373	2.397895	1	1	2.397895	C30	2012
2.197225	0.333333	14.044	1.609438	1	1	2.484907	C30	2013
2.197225	0.333333	14.10639	1.791759	1	1	2.564949	C30	2014
2.197225	0.333333	14.2085	2.302585	1	3	2.639057	C30	2015
2.197225	0.333333	14.43269	1.94591	1	3	2.70805	C30	2016
2.197225	0.333333	14.65501	1.791759	1	3	2.772589	C30	2017
2.197225	0.333333	15.10215	1.609438	1	3	2.890372	C30	2019
1.94591	0.428571	13.92741	2.302585	0	4	2.079442	C39	2012
1.94591	0.428571	14.02212	1.609438	0	4	2.197225	C39	2013
1.791759	0.5	14.09722	1.791759	0	4	2.302585	C39	2014
1.94591	0.428571	14.22795	2.079442	0	4	2.397895	C39	2015
1.94591	0.428571	14.21583	2.484907	0	4	2.484907	C39	2016
1.791759	0.333333	13.92095	2.079442	0	4	2.564949	C39	2017
1.94591	0.428571	13.97712	2.397895	0	4	2.639057	C39	2018
1.94591	0.428571	14.03961	1.791759	0	4	2.70805	C39	2019
2.079442	0.375	14.06572	2.564949	1	2	2.890372	C35	2011
2.197225	0.333333	14.14128	2.079442	1	2	2.944439	C35	2012
2.197225	0.333333	14.3513	1.94591	1	2	2.995732	C35	2013
2.197225	0.333333	14.00597	2.397895	1	2	3.044522	C35	2014
2.197225	0.333333	14.06284	2.079442	1	2	3.091042	C35	2015

2.197225	0.333333	13.68995	1.94591	1	2	3.135494	C35	2016
2.197225	0.333333	14.26538	2.302585	1	2	3.178054	C35	2017
2.079442	0.375	14.38643	2.079442	1	2	3.218876	C35	2018
2.079442	0.375	14.40313	1.609438	0	2	3.258097	C35	2019
2.197225	0.333333	12.12269	1.098612	1	1	2.079442	C13	2010
2.197225	0.333333	12.53069	1.94591	1	1	2.197225	C13	2011
2.197225	0.333333	12.6261	1.94591	1	1	2.302585	C13	2012
2.197225	0.333333	12.76311	1.94591	1	1	2.397895	C13	2013
1.94591	0.428571	12.75823	1.386294	1	1	2.484907	C13	2014
1.94591	0.428571	13.20154	1.791759	1	1	2.564949	C13	2015
1.94591	0.428571	13.33457	2.302585	1	1	2.639057	C13	2016
1.791759	0.5	13.40619	1.94591	1	1	2.70805	C13	2017
1.609438	0.6	13.50354	2.397895	1	1	2.772589	C13	2018
1.609438	0.6	14.15612	2.197225	1	1	2.833213	C13	2019
2.197225	0.333333	13.8622	2.079442	1	5	1.94591	C34	2010
2.197225	0.333333	14.56509	2.484907	1	5	2.079442	C34	2011
2.197225	0.333333	13.96048	2.564949	1	5	2.197225	C34	2012
2.197225	0.333333	14.01919	2.484907	1	5	2.302585	C36	2013
2.197225	0.333333	13.91009	2.302585	1	5	2.397895	C36	2014
2.197225	0.333333	14.13933	2.484907	1	5	2.484907	C36	2015
2.197225	0.333333	14.26932	2.564949	1	5	2.564949	C36	2016
1.94591	0.428571	13.68426	2.302585	1	3	1.609438	C39	2011
1.94591	0.428571	13.61901	2.197225	1	3	1.791759	C39	2012
1.94591	0.428571	13.88448	2.397895	1	3	1.94591	C39	2013
1.94591	0.428571	14.05193	2.197225	1	3	2.079442	C39	2014
1.609438	0.4	13.7057	2.890372	1	3	2.197225	C39	2015
1.609438	0.4	14.01796	2.397895	1	3	2.302585	C39	2016
1.609438	0.4	14.18473	2.70805	1	3	2.397895	C39	2017
1.94591	0.428571	14.93865	2.833213	1	3	2.484907	C39	2018
1.94591	0.428571	14.43845	2.302585	1	3	2.564949	C39	2019
2.397895	0.363636	14.60832	1.791759	1	1	2.079442	C29	2010
2.397895	0.363636	14.57916	2.079442	1	1	2.197225	C29	2011
2.397895	0.363636	14.59475	2.079442	1	1	2.302585	C29	2012
2.079442	0.5	14.48559	1.386294	1	1	2.397895	C29	2013
1.94591	0.428571	14.75454	2.197225	1	2	2.484907	C29	2014
1.94591	0.428571	14.91981	2.079442	1	1	2.564949	C29	2015
1.94591	0.428571	15.01617	2.302585	1	1	2.639057	C29	2016
2.302585	0.4	15.17147	2.079442	1	2	2.70805	C29	2017
2.079442	0.375	15.14901	2.484907	1	1	2.772589	C29	2018
2.197225	0.444444	15.34664	2.079442	1	1	2.833213	C29	2019
2.197225	0.333333	13.05558	1.791759	1	3	2.302585	C27	2010
2.197225	0.333333	13.21294	2.484907	1	3	2.397895	C27	2011
2.197225	0.333333	13.51603	1.791759	1	3	2.484907	C27	2012
2.197225	0.333333	13.77469	1.386294	1	3	2.564949	C27	2013
2.197225	0.333333	13.88858	2.484907	1	3	2.639057	C27	2014
1.791759	0.333333	14.27344	2.397895	1	3	2.70805	C27	2015
1.791759	0.333333	14.58116	2.302585	1	3	2.772589	C27	2016
2.197225	0.333333	14.44401	2.302585	1	3	2.833213	C27	2017
2.197225	0.333333	14.69593	2.197225	1	3	2.890372	C27	2018
2.197225	0.333333	14.76062	1.791759	1	3	2.944439	C27	2019
2.197225	0.333333	13.31464	2.079442	1	3	1.609438	I65	2011
2.197225	0.333333	13.62459	2.564949	1	3	1.791759	I65	2012
2.197225	0.333333	13.8774	2.833213	1	3	1.94591	I65	2013
2.197225	0.333333	13.87736	2.564949	1	3	2.079442	I65	2014
2.197225	0.333333	14.06821	2.944439	1	3	2.197225	I65	2015
2.079442	0.375	14.49273	3.044522	1	3	2.302585	I65	2016
2.197225	0.333333	14.60837	2.772589	1	3	2.397895	I65	2017
2.197225	0.333333	14.61594	2.890372	1	3	2.484907	R86	2018

2.197225	0.333333	14.22649	1.791759	1	2	2.079442	C35	2010
2.197225	0.333333	14.6827	2.302585	1	2	2.197225	C35	2011
2.197225	0.333333	14.68814	2.564949	1	2	2.302585	C34	2012
2.079442	0.375	14.33359	2.639057	1	2	2.397895	C34	2013
2.197225	0.333333	14.48708	2.397895	1	2	2.484907	C34	2014
2.197225	0.333333	14.76971	2.397895	1	2	2.564949	C34	2015
2.197225	0.333333	15.08294	2.302585	1	2	2.639057	C34	2016
2.197225	0.333333	15.43373	1.386294	1	2	2.70805	C34	2017
2.079442	0.375	15.41835	1.609438	1	2	2.772589	C34	2018
2.197225	0.333333	15.44395	1.94591	1	2	2.833213	C34	2019
1.791759	0.333333	12.95631	2.079442	1	5	1.94591	O81	2011
1.791759	0.333333	14.18251	2.302585	1	5	2.079442	N77	2012
2.079442	0.375	13.96048	1.94591	1	5	2.197225	N77	2013
2.197225	0.333333	13.96402	2.397895	1	5	2.302585	N77	2014
2.197225	0.333333	14.54248	2.397895	1	5	2.397895	N77	2015
2.197225	0.333333	14.37353	2.639057	1	5	2.484907	N77	2016
2.197225	0.333333	14.53437	2.302585	1	5	2.564949	N77	2017
2.079442	0.375	14.31562	2.833213	1	5	2.639057	N77	2018
1.94591	0.428571	14.33502	2.397895	1	5	2.70805	N77	2019
1.94591	0.428571	14.28551	1.94591	0	4	2.484907	I64	2011
1.94591	0.428571	14.03865	2.397895	0	4	2.564949	I65	2012
1.94591	0.428571	14.22098	2.197225	1	4	2.639057	I65	2013
1.94591	0.428571	14.44678	1.791759	1	4	2.70805	I65	2014
1.94591	0.428571	14.46261	2.70805	1	3	2.772589	I65	2015
2.197225	0.333333	14.49365	1.791759	1	3	2.833213	I65	2016
2.197225	0.333333	14.60606	2.302585	1	3	2.890372	I65	2017
2.079442	0.375	14.77849	2.079442	1	4	2.944439	I65	2018
2.197225	0.333333	14.35539	2.079442	1	5	2.079442	N78	2011
2.197225	0.333333	14.42951	2.70805	1	5	2.197225	N77	2012
2.197225	0.333333	14.36461	2.484907	1	5	2.302585	N77	2013
2.197225	0.333333	14.42283	1.791759	1	5	2.397895	N77	2014
2.197225	0.333333	14.51519	2.564949	1	5	2.484907	N77	2015
2.197225	0.333333	14.5969	2.484907	1	5	2.564949	N77	2016
2.197225	0.333333	14.58506	2.639057	1	5	2.639057	N77	2017
2.197225	0.333333	14.67612	2.564949	1	5	2.70805	N77	2018
2.197225	0.333333	15.19208	2.397895	1	5	2.772589	N77	2019
2.197225	0.444444	14.60972	2.197225	1	3	2.079442	B11	2011
2.197225	0.444444	14.82281	2.079442	1	3	2.197225	B07	2012
2.197225	0.444444	14.63551	2.079442	1	3	2.302585	B11	2013
1.94591	0.428571	14.58307	1.94591	1	3	2.397895	B11	2014
1.94591	0.428571	14.30323	2.197225	1	3	2.484907	B11	2015
1.94591	0.428571	14.06331	2.079442	1	3	2.564949	B11	2016
1.94591	0.428571	14.1559	2.197225	1	3	2.639057	B11	2017
1.94591	0.428571	14.53257	2.302585	1	3	2.70805	B11	2018
1.94591	0.428571	14.71551	2.197225	1	3	2.772589	B11	2019
2.197225	0.333333	13.64187	2.397895	1	1	1.94591	C27	2011
2.197225	0.333333	13.67152	2.302585	1	1	2.079442	C27	2012
2.197225	0.333333	13.82397	1.94591	1	1	2.197225	C27	2013
2.197225	0.333333	13.83962	2.197225	1	1	2.302585	C27	2014
2.197225	0.333333	13.90918	2.564949	1	1	2.397895	C27	2015
2.197225	0.333333	13.9196	2.302585	1	1	2.484907	C27	2016
2.197225	0.333333	13.91309	1.609438	1	1	2.564949	C27	2017
2.197225	0.333333	14.08233	2.197225	1	1	2.639057	C27	2018
2.197225	0.333333	14.08355	2.302585	1	1	2.70805	C27	2019
1.94591	0.428571	15.3103	2.484907	1	3	3.091042	C35	2017
1.94591	0.428571	15.72704	2.772589	1	3	3.135494	C35	2018
2.197225	0.333333	14.04105	1.94591	0	2	2.197225	C30	2011
2.197225	0.333333	14.08294	2.302585	0	2	2.302585	C30	2012

2.197225	0.333333	14.09011	1.791759	0	2	2.397895	C30	2013
2.197225	0.333333	14.14301	1.94591	0	3	2.484907	C30	2014
2.197225	0.333333	14.22636	1.609438	0	3	2.564949	C30	2015
2.197225	0.333333	14.2022	2.197225	0	3	2.639057	C30	2016
2.197225	0.333333	14.06237	2.197225	1	3	2.70805	C30	2017
2.197225	0.333333	14.25377	2.302585	1	3	2.772589	C30	2018
2.197225	0.333333	14.41435	1.94591	1	3	2.833213	C30	2019
2.197225	0.333333	13.14315	2.302585	1	4	2.079442	C29	2011
2.079442	0.375	13.52355	2.564949	1	4	2.197225	C29	2012
2.197225	0.333333	13.54449	2.397895	1	3	2.302585	C29	2013
2.197225	0.333333	13.54278	2.302585	1	4	2.397895	C29	2014
2.197225	0.333333	13.70503	2.302585	1	4	2.484907	C29	2015
2.197225	0.333333	13.0954	2.397895	1	4	1.94591	C35	2011
2.197225	0.333333	13.34011	2.302585	1	3	2.079442	C35	2012
2.197225	0.333333	13.75053	2.197225	1	3	2.397895	C34	2015
2.197225	0.333333	13.7726	2.079442	1	3	2.484907	C34	2016
2.197225	0.333333	13.7235	1.94591	1	3	2.564949	C34	2017
2.197225	0.333333	13.53791	1.94591	1	3	2.639057	C34	2018
2.197225	0.333333	13.58798	2.197225	1	3	2.70805	C34	2019
2.197225	0.333333	13.57649	1.791759	1	4	2.197225	C27	2011
2.197225	0.333333	14.18057	2.302585	1	4	2.302585	C27	2012
2.079442	0.375	14.12996	1.791759	0	4	2.397895	C27	2013
2.197225	0.333333	14.32233	1.386294	0	4	2.484907	C27	2014
2.197225	0.333333	14.10219	2.197225	0	4	2.564949	C27	2015
2.197225	0.333333	14.40878	2.484907	0	4	2.639057	C27	2016
2.197225	0.333333	14.4941	1.791759	0	4	2.70805	C27	2017
2.197225	0.333333	14.59722	2.197225	0	4	2.772589	C27	2018
2.197225	0.333333	14.89962	1.791759	0	4	2.833213	C27	2019
1.94591	0.428571	13.28159	2.302585	1	3	2.564949	C38	2011
1.94591	0.428571	13.0549	2.302585	1	4	2.639057	C38	2012
1.94591	0.428571	13.04732	2.484907	1	3	2.70805	C38	2013
1.94591	0.428571	13.86797	2.484907	1	4	2.772589	C38	2014
2.197225	0.444444	13.70402	2.484907	1	3	2.833213	C38	2015
2.397895	0.363636	13.73798	2.564949	1	4	2.890372	C38	2016
2.197225	0.444444	14.61868	1.791759	1	3	2.639057	M74	2011
2.197225	0.333333	14.63914	1.94591	1	3	2.70805	M74	2012
2.197225	0.333333	14.67857	1.791759	1	3	2.772589	M74	2013
2.197225	0.333333	14.76311	1.609438	1	2	2.833213	M74	2014
2.197225	0.333333	14.76489	2.079442	1	2	2.890372	M74	2015
2.079442	0.375	14.70775	2.079442	1	2	2.944439	M74	2016
2.197225	0.333333	14.71197	1.94591	1	2	2.995732	M74	2017
2.197225	0.333333	15.08175	1.94591	1	2	3.044522	M74	2018
2.197225	0.333333	15.05666	1.94591	1	3	3.091042	M74	2019
2.197225	0.333333	12.84133	1.94591	1	3	2.197225	C35	2011
2.197225	0.333333	13.75693	2.484907	1	3	2.302585	C35	2012
2.197225	0.333333	13.97567	2.564949	1	3	2.397895	C35	2013
2.197225	0.333333	13.99491	2.302585	1	3	2.484907	C35	2014
2.197225	0.333333	14.04615	2.70805	1	2	2.564949	C35	2015
2.197225	0.333333	14.17911	2.484907	1	2	2.639057	C35	2016
2.197225	0.333333	14.26767	2.564949	1	3	2.70805	C35	2017
1.94591	0.428571	13.33747	2.079442	1	2	2.397895	C38	2011
1.791759	0.5	13.32121	1.94591	1	2	2.484907	C38	2012
1.94591	0.428571	13.33747	2.197225	1	2	2.564949	C38	2013
1.94591	0.428571	13.34086	1.386294	1	2	2.639057	C38	2014
1.791759	0.5	13.47541	1.609438	1	2	2.70805	C38	2015
1.791759	0.5	13.40784	1.791759	1	2	2.772589	C38	2016
1.94591	0.428571	13.81751	2.079442	1	2	2.833213	C38	2017
1.94591	0.428571	13.87302	2.079442	1	2	2.890372	C38	2018

1.94591	0.428571	13.88289	2.197225	1	2	2.944439	C38	2019
1.609438	0.4	13.08549	2.079442	1	3	1.791759	C39	2011
1.609438	0.4	13.1337	1.94591	1	3	1.94591	C39	2012
1.609438	0.4	13.28056	2.079442	1	3	2.079442	C39	2013
1.609438	0.4	13.36248	2.079442	1	3	2.197225	C39	2014
1.609438	0.4	12.83734	2.397895	1	3	2.302585	C39	2015
1.609438	0.4	12.8474	2.397895	1	3	2.397895	C39	2016
1.609438	0.4	13.20006	2.079442	1	3	2.564949	C39	2018
1.609438	0.4	13.46737	1.94591	1	3	2.639057	C39	2019
2.197225	0.333333	13.97414	1.94591	0	2	2.890372	C26	2011
2.197225	0.333333	14.34861	1.791759	0	2	2.944439	C26	2012
1.791759	0.5	13.55115	1.94591	0	2	2.995732	C26	2013
2.197225	0.333333	14.25351	1.791759	0	2	3.044522	C26	2014
2.197225	0.333333	14.52507	1.791759	0	2	3.091042	C26	2015
2.197225	0.333333	14.54054	2.197225	0	2	3.135494	C26	2016
1.94591	0.428571	13.9761	2.302585	0	2	3.178054	C26	2017
2.197225	0.333333	15.33248	2.079442	1	1	2.772589	C29	2018
2.079442	0.375	16.4505	1.609438	0	2	2.833213	C29	2019
2.197225	0.333333	14.42696	2.397895	1	2	1.94591	C26	2011
2.197225	0.333333	14.59539	2.079442	1	4	2.079442	C27	2012
2.197225	0.333333	14.6462	1.791759	1	2	2.197225	C27	2013
2.197225	0.333333	14.59502	2.397895	1	4	2.302585	C27	2014
2.197225	0.333333	14.43807	2.484907	1	2	2.397895	C27	2015
2.197225	0.333333	14.67718	2.639057	1	4	2.484907	C27	2016
2.397895	0.363636	14.69551	2.639057	1	2	2.564949	C27	2017
2.397895	0.363636	14.79007	1.94591	1	4	2.639057	C27	2018
2.397895	0.363636	14.83688	2.079442	1	2	2.70805	C27	2019
2.197225	0.333333	14.24163	1.791759	0	3	1.94591	C26	2011
2.197225	0.333333	14.20674	2.397895	0	3	2.079442	C26	2012
2.197225	0.333333	14.29143	2.197225	0	3	2.197225	C26	2013
2.197225	0.333333	13.96324	1.94591	1	3	2.302585	C26	2014
2.197225	0.333333	14.04089	1.791759	1	3	2.397895	C26	2015
2.197225	0.333333	14.14481	2.302585	1	3	2.484907	C26	2016
2.197225	0.333333	14.47277	2.079442	1	3	2.564949	C26	2017
2.197225	0.333333	14.4462	2.079442	1	3	2.639057	C26	2018
2.197225	0.333333	14.46627	1.791759	1	3	2.70805	C26	2019
2.197225	0.333333	14.69115	1.94591	1	4	2.484907	C35	2011
2.079442	0.375	14.85886	1.609438	1	4	2.564949	C35	2012
1.94591	0.428571	14.25021	2.197225	1	4	2.639057	C35	2013
1.94591	0.428571	14.29522	2.079442	1	4	2.70805	C35	2014
1.94591	0.428571	14.33114	1.609438	1	4	2.772589	C35	2015
1.94591	0.428571	14.61845	2.302585	1	4	2.833213	C35	2016
1.791759	0.333333	14.38207	2.302585	1	4	2.890372	C35	2017
1.94591	0.428571	13.43674	2.302585	1	5	2.639057	C27	2011
1.94591	0.428571	13.79122	2.079442	1	5	2.70805	C27	2012
1.94591	0.428571	13.88205	1.609438	1	5	2.772589	C27	2013
1.94591	0.428571	13.96125	2.079442	1	5	2.833213	C27	2014
1.94591	0.428571	13.98119	2.197225	1	5	2.890372	C27	2015
1.94591	0.428571	14.09602	1.609438	1	5	2.944439	C27	2016
1.94591	0.428571	14.19764	1.791759	1	5	2.995732	C27	2017
1.94591	0.428571	14.24742	2.302585	1	5	3.044522	C27	2018
1.94591	0.428571	14.12042	2.197225	1	5	3.091042	C27	2019
1.94591	0.428571	14.52251	2.564949	1	4	2.70805	I64	2017
2.197225	0.333333	13.82951	2.564949	1	4	2.833213	I64	2019
2.197225	0.333333	12.97386	1.098612	1	2	2.772589	C38	2011
2.197225	0.333333	13.30201	2.079442	1	2	2.833213	C38	2012
2.197225	0.333333	13.31628	1.94591	1	2	2.890372	C38	2013
2.197225	0.333333	13.53844	1.791759	1	2	2.944439	C38	2014

2.197225	0.333333	14.02634	2.079442	1	2	2.995732	C38	2015
2.197225	0.333333	14.04662	2.484907	1	2	3.044522	C38	2016
2.197225	0.333333	14.37621	2.484907	1	2	3.091042	C38	2017
1.609438	0.4	12.6885	1.94591	1	3	2.197225	C35	2011
1.609438	0.4	13.26906	2.772589	1	3	2.302585	C35	2012
1.609438	0.4	13.35348	2.397895	1	3	2.397895	C35	2013
1.609438	0.4	13.35348	1.94591	1	3	2.484907	C35	2014
1.609438	0.4	13.35348	2.302585	1	3	2.564949	C35	2015
1.609438	0.4	13.56705	2.639057	1	3	2.639057	C35	2016
1.609438	0.4	13.82546	2.944439	1	3	2.70805	C35	2017
1.609438	0.4	13.82546	2.564949	0	3	2.772589	I65	2018
1.609438	0.4	13.85011	2.484907	0	4	2.833213	I65	2019
1.94591	0.428571	13.20597	1.94591	0	4	1.94591	I65	2011
1.94591	0.428571	13.21585	2.302585	0	4	2.079442	I65	2012
1.94591	0.428571	13.66805	1.791759	1	4	2.197225	I65	2013
1.94591	0.428571	13.87764	2.397895	1	4	2.302585	I65	2014
1.94591	0.428571	14.34496	2.397895	0	4	2.397895	I65	2015
1.94591	0.428571	14.33977	2.639057	0	4	2.484907	I65	2016
1.94591	0.428571	14.37769	2.079442	0	4	2.564949	I65	2017
1.94591	0.428571	14.56168	2.197225	0	4	2.639057	I65	2018
1.94591	0.428571	14.70751	2.397895	0	4	2.70805	I65	2019
2.197225	0.444444	13.78536	1.791759	1	2	2.397895	C27	2011
2.197225	0.444444	14.09405	2.397895	1	2	2.484907	C27	2012
2.197225	0.444444	14.08401	1.94591	1	2	2.564949	C27	2013
2.197225	0.333333	14.16532	1.609438	1	2	2.639057	C27	2014
2.197225	0.333333	14.38082	2.079442	1	2	2.70805	C27	2015
2.079442	0.375	14.36802	2.197225	1	2	2.772589	C27	2016
2.079442	0.375	14.46986	2.197225	1	2	2.833213	C27	2017
1.609438	0.4	14.46403	1.791759	1	2	2.890372	C27	2018
1.609438	0.4	14.19709	2.079442	1	2	2.944439	C27	2019
2.079442	0.375	13.86525	2.197225	0	4	2.944439	C36	2011
2.079442	0.375	13.97217	2.197225	0	4	2.995732	C36	2012
2.079442	0.375	14.03705	1.94591	0	4	3.044522	C36	2013
2.079442	0.375	14.04987	1.609438	0	4	3.091042	C36	2014
2.079442	0.375	14.0619	1.098612	0	4	3.135494	C36	2015
2.079442	0.375	14.13643	1.386294	0	4	3.178054	C36	2016
1.94591	0.428571	14.21603	1.609438	0	4	3.218876	C36	2017
1.94591	0.428571	14.30776	1.609438	0	4	3.258097	C36	2018
1.94591	0.428571	14.27729	1.609438	0	4	3.295837	C36	2019
2.197225	0.333333	13.56062	1.098612	1	2	2.772589	C34	2011
2.197225	0.333333	13.56062	2.484907	1	1	2.833213	C34	2012
2.197225	0.333333	13.56062	2.302585	1	1	2.890372	C34	2013
2.197225	0.333333	14.29423	2.079442	1	2	2.944439	C34	2014
2.197225	0.333333	15.09644	2.302585	1	2	2.995732	C34	2015
2.197225	0.333333	15.1465	2.484907	1	2	3.044522	C34	2016
2.197225	0.333333	15.15051	1.94591	1	2	3.091042	C34	2017
2.197225	0.333333	15.2265	2.079442	1	2	3.135494	C34	2018
2.197225	0.333333	15.24103	2.197225	1	2	3.178054	C34	2019
2.197225	0.333333	13.32121	2.079442	0	4	2.944439	C34	2011
2.197225	0.333333	13.52518	2.197225	0	4	2.995732	C34	2012
2.197225	0.333333	13.65299	2.197225	0	4	3.044522	C34	2013
2.197225	0.333333	13.77385	2.302585	0	4	3.091042	C34	2014
1.94591	0.428571	13.5277	2.397895	0	4	3.135494	C34	2015
2.197225	0.333333	13.83482	2.197225	0	4	3.178054	C34	2016
2.197225	0.333333	14.59286	2.079442	0	4	3.218876	N77	2017
2.197225	0.333333	13.15966	1.791759	1	3	2.079442	C27	2011
2.197225	0.333333	13.62773	2.079442	1	3	2.197225	C27	2012
2.197225	0.333333	13.91996	2.564949	1	3	2.302585	C27	2013

2.197225	0.333333	13.9789	3.044522	1	3	2.397895	C27	2014
2.197225	0.333333	15.8587	2.564949	1	3	2.484907	C27	2015
2.197225	0.333333	13.94654	2.484907	1	3	2.564949	C27	2016
2.197225	0.333333	14.15341	2.079442	1	3	2.639057	C27	2017
2.197225	0.333333	14.17932	2.079442	1	3	2.70805	C27	2018
2.197225	0.333333	14.03841	1.94591	1	3	2.772589	C27	2019
2.197225	0.333333	13.26819	1.94591	1	2	2.484907	C35	2011
2.079442	0.375	13.35045	2.197225	1	2	2.564949	C35	2012
2.197225	0.333333	13.35585	1.94591	1	2	2.639057	C35	2013
2.197225	0.333333	13.54265	2.079442	1	2	2.70805	C35	2014
2.197225	0.333333	13.59237	1.609438	1	2	2.772589	C35	2015
2.197225	0.333333	13.49599	2.197225	1	2	2.833213	C35	2016
2.197225	0.333333	13.85444	2.197225	1	2	2.890372	C35	2017
2.197225	0.333333	13.8208	1.94591	1	2	2.944439	C35	2018
2.197225	0.333333	14.14826	2.079442	1	2	2.995732	C35	2019
2.197225	0.333333	13.91581	1.791759	1	2	2.197225	C34	2011
2.197225	0.333333	14.28126	2.302585	1	2	2.302585	C34	2012
2.197225	0.333333	14.01412	2.079442	1	2	2.397895	C34	2013
2.197225	0.333333	14.01009	2.079442	1	2	2.484907	C34	2014
1.791759	0.5	13.75332	1.94591	1	2	2.564949	C34	2015
2.197225	0.333333	13.90462	2.484907	0	3	2.639057	C34	2016
2.197225	0.333333	13.95814	2.639057	0	3	2.70805	C34	2017
2.484907	0.333333	13.96712	2.397895	0	3	2.772589	C34	2018
2.397895	0.363636	13.70503	1.94591	0	3	2.833213	C34	2019
1.94591	0.428571	12.94801	2.197225	1	4	3.178054	C35	2011
1.94591	0.428571	13.07107	1.94591	1	4	3.218876	C35	2012
1.94591	0.428571	12.9109	2.639057	1	4	3.258097	C35	2013
1.94591	0.428571	13.43014	2.484907	1	4	3.295837	C35	2014
1.94591	0.428571	13.71104	2.079442	1	4	3.332205	C35	2015
1.94591	0.428571	14.2007	2.302585	1	4	3.367296	C35	2016
1.94591	0.428571	14.00348	2.197225	1	4	3.401197	C35	2017
1.94591	0.428571	14.14898	2.197225	1	4	3.433987	C35	2018
2.197225	0.333333	13.92964	1.791759	1	1	3.044522	C38	2018
2.197225	0.333333	13.96729	2.079442	1	1	3.091042	C38	2019
2.197225	0.333333	15.47944	2.197225	1	3	2.70805	C27	2012
2.197225	0.333333	15.50284	1.609438	1	3	2.772589	C27	2013
2.197225	0.333333	15.51296	2.639057	1	2	2.833213	C27	2014
2.197225	0.333333	15.50561	2.70805	0	2	2.890372	C27	2015
1.791759	0.5	14.71681	2.302585	1	3	2.944439	C27	2016
1.94591	0.428571	14.90205	1.94591	1	2	2.995732	C27	2017
1.94591	0.428571	13.67567	1.791759	1	1	2.397895	C35	2015
1.791759	0.5	13.1904	2.484907	1	1	2.484907	C35	2016
1.609438	0.6	12.50395	2.079442	1	3	2.564949	C35	2017
1.609438	0.6	12.58245	2.079442	1	3	2.639057	C35	2018
1.609438	0.6	12.83974	1.386294	1	3	2.70805	C35	2019
2.197225	0.333333	13.98102	1.94591	1	4	2.302585	I63	2011
2.197225	0.333333	13.69425	2.197225	1	4	2.397895	I65	2012
2.197225	0.333333	13.76432	2.484907	1	4	2.484907	I65	2013
2.197225	0.333333	13.88055	2.302585	1	4	2.564949	I65	2014
2.197225	0.333333	14.27899	2.772589	1	4	2.639057	I65	2015
2.197225	0.333333	14.53179	2.890372	1	4	2.70805	I65	2016
2.079442	0.375	14.67773	2.197225	1	4	2.772589	I65	2017
2.197225	0.333333	14.72582	2.079442	1	4	2.833213	I65	2018
2.197225	0.555556	13.71015	2.397895	1	4	1.94591	C39	2012
2.197225	0.555556	13.73647	2.397895	1	4	2.079442	C39	2013
2.197225	0.555556	13.67854	2.197225	1	4	2.197225	C39	2014
1.94591	0.571429	13.78711	2.397895	1	4	2.302585	C39	2015
1.94591	0.571429	13.50449	2.302585	1	4	2.397895	C39	2016

1.94591	0.428571	13.33699	2.564949	1	4	2.484907	C39	2017
1.94591	0.428571	14.31421	2.302585	1	4	1.94591	C39	2012
1.94591	0.428571	14.55431	1.791759	1	4	2.079442	C39	2013
1.94591	0.428571	14.5936	1.791759	1	4	2.197225	C39	2014
1.609438	0.4	14.67107	2.397895	1	4	2.302585	C39	2015
1.609438	0.4	14.59484	2.197225	1	4	2.397895	C39	2016
1.609438	0.4	14.81357	1.94591	1	4	2.484907	C39	2017
1.609438	0.4	14.48442	2.197225	1	4	2.564949	C39	2018
1.609438	0.4	14.89664	2.302585	1	4	2.639057	C39	2019
1.94591	0.428571	13.2122	1.791759	1	2	1.94591	C35	2012
1.791759	0.5	13.23783	2.197225	1	2	2.079442	C35	2013
1.791759	0.5	13.38103	1.94591	1	2	2.197225	C35	2014
1.94591	0.428571	13.32285	2.197225	1	3	2.302585	C35	2015
1.94591	0.428571	13.35268	2.302585	1	3	2.397895	C35	2016
2.197225	0.444444	15.5678	2.564949	1	3	2.564949	C39	2018
2.197225	0.444444	15.55599	2.484907	1	2	2.639057	C39	2019
1.609438	0.4	14.16186	2.197225	0	3	1.791759	C35	2012
1.609438	0.4	13.95527	2.484907	1	3	1.94591	C40	2013
1.609438	0.4	14.22098	2.302585	1	3	2.079442	C40	2014
1.609438	0.4	14.40978	2.70805	1	3	2.197225	C40	2015
1.609438	0.4	14.03793	3.091042	1	3	2.397895	C40	2017
2.197225	0.333333	13.64318	1.791759	1	3	3.135494	C35	2015
2.197225	0.333333	13.71414	1.791759	1	3	3.178054	C35	2016
2.197225	0.333333	13.84381	1.386294	1	3	3.218876	C35	2017
2.197225	0.333333	13.93852	1.609438	1	3	3.258097	C35	2018
2.197225	0.333333	14.29979	1.791759	1	3	3.295837	C35	2019
2.079442	0.375	14.96291	1.791759	1	5	1.791759	C35	2012
2.197225	0.333333	14.88022	1.791759	1	5	1.94591	C35	2013
2.197225	0.333333	14.89494	2.079442	1	5	2.079442	C35	2014
2.079442	0.375	14.89945	2.397895	1	5	2.197225	C35	2015
2.197225	0.333333	15.13278	2.397895	1	3	2.302585	C35	2016
2.197225	0.333333	15.09366	2.397895	1	3	2.397895	C35	2017
2.197225	0.333333	15.3669	2.484907	1	3	2.484907	C35	2018
2.197225	0.333333	15.40025	2.397895	1	4	2.564949	C35	2019
2.197225	0.333333	14.17661	2.197225	0	3	2.944439	C38	2012
2.197225	0.333333	14.21382	2.70805	0	3	2.995732	C38	2013
1.791759	0.333333	14.2648	2.564949	0	3	3.044522	C38	2014
1.791759	0.333333	14.30789	3.091042	1	3	3.091042	C38	2015
1.791759	0.333333	14.32672	2.944439	1	3	3.135494	C38	2016
1.791759	0.333333	14.50173	2.772589	0	3	3.178054	C38	2017
2.197225	0.333333	13.71015	1.791759	1	4	2.639057	C29	2012
2.197225	0.333333	13.71015	1.609438	1	4	2.70805	C29	2013
2.197225	0.333333	13.81751	1.94591	1	4	2.772589	C29	2014
2.197225	0.333333	13.88746	1.791759	1	4	2.833213	C29	2015
2.197225	0.333333	13.88876	1.609438	1	4	2.890372	C29	2016
2.197225	0.333333	13.85377	2.397895	1	4	2.944439	C29	2017
2.197225	0.333333	14.2239	2.079442	1	4	2.995732	C29	2018
2.197225	0.333333	14.33204	1.791759	1	4	3.044522	C29	2019
2.079442	0.375	13.47302	1.94591	1	1	2.397895	C24	2012
1.94591	0.428571	13.69334	1.609438	1	1	2.484907	C24	2013
2.079442	0.375	13.78865	2.079442	1	1	2.564949	C24	2014
2.079442	0.375	13.78865	1.791759	1	1	2.639057	C24	2015
2.079442	0.375	13.78865	1.386294	1	1	2.70805	C24	2016
2.079442	0.375	13.88401	1.609438	1	1	2.772589	C24	2017
2.079442	0.375	13.96082	2.079442	1	1	2.833213	C24	2018
2.079442	0.375	13.96082	1.94591	1	1	2.890372	C24	2019
1.94591	0.428571	13.55415	1.94591	1	3	2.639057	C32	2012
1.94591	0.428571	13.57979	2.079442	1	3	2.70805	C32	2013

1.94591	0.428571	13.78505	2.079442	1	3	2.772589	C32	2014
1.94591	0.428571	14.19395	1.609438	1	2	2.833213	C32	2015
1.94591	0.428571	14.18707	1.94591	1	2	2.890372	C32	2016
1.94591	0.428571	13.99783	1.94591	1	2	2.944439	C32	2017
1.94591	0.428571	14.25377	1.94591	1	3	2.995732	C32	2018
1.94591	0.428571	14.23665	2.197225	1	3	3.044522	C32	2019
2.197225	0.333333	13.52649	1.94591	1	1	2.484907	C40	2012
2.079442	0.375	13.28872	2.079442	1	1	2.564949	C40	2013
1.609438	0.4	13.83551	1.94591	1	1	2.639057	C40	2014
1.609438	0.4	14.07841	1.609438	1	1	2.70805	C40	2015
1.609438	0.4	14.1484	2.564949	1	1	2.772589	C40	2016
2.197225	0.333333	14.40452	2.484907	1	3	2.833213	C40	2017
2.397895	0.363636	14.57744	2.639057	1	3	2.890372	C40	2018
2.197225	0.333333	13.44879	1.098612	1	1	2.302585	C38	2012
2.079442	0.375	13.56846	1.94591	1	1	2.397895	C38	2013
2.079442	0.375	13.68995	2.079442	1	1	2.484907	C38	2014
2.197225	0.333333	13.6195	1.94591	1	1	2.564949	C38	2015
2.197225	0.333333	13.7343	1.94591	1	1	2.639057	C38	2016
2.197225	0.333333	13.76632	2.197225	1	1	2.70805	C38	2017
2.197225	0.333333	14.12005	2.197225	1	1	2.772589	C38	2018
2.197225	0.333333	13.58628	1.791759	1	3	2.833213	C33	2012
1.791759	0.5	13.98854	1.791759	1	3	2.890372	C33	2013
1.94591	0.428571	13.87999	1.94591	0	3	2.944439	C33	2014
1.94591	0.428571	13.93329	2.397895	1	3	2.995732	C33	2015
1.94591	0.428571	13.41652	2.484907	1	3	3.044522	C33	2016
1.791759	0.5	13.99783	2.397895	1	3	3.091042	C33	2017
1.94591	0.428571	13.83825	2.197225	1	3	3.178054	C33	2019
1.94591	0.428571	12.80875	1.098612	1	4	2.484907	G59	2012
1.94591	0.428571	13.62677	2.079442	1	4	2.564949	G59	2013
2.079442	0.5	13.62677	2.079442	1	4	2.639057	G59	2014
2.197225	0.333333	13.62677	1.791759	1	4	2.70805	G59	2015
2.197225	0.333333	13.62677	1.609438	1	4	2.772589	G59	2016
2.197225	0.333333	13.62677	1.94591	1	4	2.833213	I65	2017
1.94591	0.428571	13.65299	1.386294	1	1	3.091042	C39	2012
1.94591	0.428571	13.65299	1.791759	1	1	3.135494	C39	2013
1.94591	0.428571	13.68768	2.079442	1	1	3.178054	C39	2014
1.94591	0.428571	13.68768	1.94591	1	4	3.218876	C39	2015
2.079442	0.375	13.75364	1.94591	1	4	3.258097	C39	2016
2.079442	0.375	13.94654	2.079442	1	1	3.295837	C39	2017
1.94591	0.428571	13.78865	1.386294	1	3	2.944439	C40	2012
1.94591	0.428571	13.48241	1.609438	1	3	2.995732	C40	2013
1.94591	0.428571	13.49544	1.791759	1	3	3.044522	C40	2014
1.94591	0.428571	13.79714	1.94591	1	3	3.091042	C40	2015
1.94591	0.428571	13.65323	1.609438	1	3	3.135494	C40	2016
1.791759	0.5	13.68335	1.609438	1	3	3.178054	C40	2017
1.94591	0.428571	13.81551	1.609438	1	3	3.218876	C40	2018
1.94591	0.428571	14.11547	1.098612	1	3	3.258097	C40	2019
2.397895	0.363636	13.33747	2.484907	1	4	2.484907	C34	2013
2.079442	0.375	13.32775	2.639057	1	4	2.564949	C34	2014
2.079442	0.375	13.52916	2.890372	1	2	2.639057	C34	2015
2.079442	0.375	13.77469	2.772589	1	2	2.70805	C34	2016
2.197225	0.333333	13.89247	3.091042	1	2	2.772589	C34	2017
1.94591	0.428571	14.58205	1.609438	1	4	3.218876	C29	2013
1.94591	0.428571	14.91011	2.397895	1	4	3.258097	C29	2014
1.94591	0.428571	14.28782	2.564949	1	4	3.295837	C29	2015
1.94591	0.428571	14.33305	1.94591	1	4	3.332205	C29	2016
1.94591	0.428571	14.34543	2.197225	1	4	3.367296	C29	2017
1.94591	0.428571	14.64637	2.397895	1	4	3.401197	C29	2018

1.94591	0.428571	14.36253	2.302585	1	4	3.433987	C29	2019
1.94591	0.428571	14.22994	2.484907	1	5	2.564949	C38	2014
1.94591	0.428571	14.24012	2.484907	1	5	2.639057	C38	2015
1.94591	0.428571	14.35311	2.70805	1	5	2.70805	C38	2016
1.94591	0.428571	14.17458	3.044522	1	5	2.772589	C38	2017
1.94591	0.428571	13.86325	2.890372	1	5	2.833213	C38	2018
1.94591	0.428571	14.00249	2.70805	1	5	2.890372	C38	2019
2.397895	0.363636	14.43393	1.94591	0	2	2.772589	I65	2013
2.197225	0.333333	14.07054	2.397895	0	3	2.833213	I65	2014
2.197225	0.333333	14.61432	2.197225	0	3	2.890372	I65	2015
1.94591	0.428571	14.52748	2.079442	0	3	2.944439	I65	2016
1.94591	0.428571	14.48088	2.079442	1	4	2.890372	I65	2017
1.94591	0.428571	14.60201	2.397895	1	4	2.944439	I65	2018
1.94591	0.428571	14.6916	2.079442	1	4	2.995732	I65	2019
2.397895	0.363636	13.87284	2.772589	1	3	2.833213	N77	2014
2.397895	0.363636	14.18008	2.890372	1	3	2.890372	N77	2015
2.079442	0.375	14.01272	2.995732	1	3	2.944439	N77	2016
2.197225	0.333333	14.88205	2.995732	1	3	2.995732	N77	2017
2.079442	0.375	14.19723	3.044522	1	3	3.044522	N77	2018
2.197225	0.333333	14.08958	2.564949	1	3	3.091042	N77	2019
1.609438	0.4	13.64116	2.197225	1	4	2.833213	C39	2014
1.609438	0.4	13.97251	2.302585	1	4	2.890372	C39	2015
1.791759	0.333333	13.83923	1.94591	1	4	2.944439	C39	2016
1.791759	0.333333	13.82941	1.609438	1	4	2.995732	C39	2017
1.791759	0.333333	13.87802	1.609438	1	1	3.044522	C39	2018
1.791759	0.333333	14.14948	1.609438	1	1	3.091042	C39	2019
2.197225	0.333333	13.58936	1.791759	1	2	2.639057	C14	2014
2.197225	0.333333	13.62277	1.609438	1	2	2.70805	C14	2015
2.197225	0.333333	13.6504	2.079442	1	2	2.772589	C14	2016
2.197225	0.333333	13.66317	2.197225	1	2	2.833213	C14	2017
2.197225	0.333333	14.04424	1.94591	1	2	2.890372	C14	2018
2.197225	0.333333	14.33555	1.94591	1	2	2.944439	C14	2019
2.197225	0.333333	13.10032	2.079442	1	3	2.484907	C26	2014
2.197225	0.333333	13.05194	2.079442	1	3	2.564949	C26	2015
2.197225	0.333333	12.84212	1.791759	1	3	2.639057	C26	2016
2.197225	0.333333	12.97433	1.609438	1	3	2.70805	C26	2017
2.197225	0.333333	13.0641	1.94591	1	3	2.772589	C26	2018
2.197225	0.333333	13.17039	1.386294	1	3	2.833213	C26	2019
2.197225	0.333333	14.25306	1.386294	1	3	2.833213	C40	2014
2.197225	0.333333	14.24508	2.564949	1	3	2.890372	C40	2015
2.197225	0.333333	14.4635	2.397895	1	3	2.944439	C40	2016
2.197225	0.333333	14.70229	2.197225	1	1	2.995732	C40	2017
2.197225	0.333333	15.05658	2.484907	1	4	3.044522	C35	2018
1.94591	0.428571	13.49833	2.079442	1	2	2.197225	C34	2014
1.94591	0.428571	13.79694	2.197225	1	2	2.302585	C34	2015
1.94591	0.428571	13.81551	2.302585	1	4	2.397895	C34	2016
1.94591	0.428571	13.8439	2.302585	1	2	2.484907	C34	2017
2.197225	0.333333	13.0782	1.098612	1	2	1.791759	C35	2014
2.197225	0.333333	13.14138	2.302585	1	2	1.94591	C35	2015
2.197225	0.333333	13.3962	2.484907	1	2	2.079442	C35	2016
2.197225	0.333333	13.49475	2.397895	1	2	2.197225	C35	2017
2.197225	0.333333	13.4778	2.197225	1	2	2.302585	C35	2018
2.197225	0.333333	13.8503	1.609438	1	2	2.397895	C35	2019
2.079442	0.375	14.04105	2.197225	1	3	2.397895	C39	2015
2.197225	0.333333	15.06076	2.079442	1	4	2.484907	C35	2014
2.197225	0.333333	15.11954	1.791759	1	4	2.564949	C35	2015
2.197225	0.333333	14.99223	2.197225	1	4	2.639057	C35	2016
2.397895	0.363636	15.47366	1.94591	1	4	2.70805	C35	2017

2.397895	0.363636	15.79005	1.609438	1	3	2.772589	C35	2018
2.397895	0.363636	15.45106	1.791759	1	4	2.833213	C35	2019
2.197225	0.333333	13.06917	1.791759	1	5	2.564949	C34	2014
2.197225	0.333333	12.87262	2.302585	1	5	2.639057	C34	2015
2.079442	0.375	12.85892	2.079442	1	5	2.70805	C34	2016
2.197225	0.333333	12.91902	2.833213	1	5	2.772589	C34	2017
2.197225	0.333333	13.79551	2.197225	1	3	2.833213	C34	2018
2.197225	0.333333	13.98568	1.94591	0	5	2.890372	C34	2019
2.079442	0.5	14.87974	2.397895	1	3	2.302585	C37	2015
2.197225	0.333333	14.40928	1.94591	1	3	2.397895	C37	2016
2.484907	0.333333	14.29379	2.397895	1	3	2.484907	C37	2017
2.484907	0.333333	14.54345	2.995732	1	3	2.564949	C37	2018
2.079442	0.375	14.58075	2.484907	1	3	2.639057	C37	2019
1.94591	0.428571	14.66792	2.484907	1	3	2.302585	C38	2015
1.94591	0.428571	14.6585	2.079442	1	3	2.397895	C38	2016
1.94591	0.428571	14.54898	2.70805	1	3	2.484907	C38	2017
1.94591	0.428571	14.58668	2.197225	1	4	2.564949	C38	2018
2.079442	0.375	14.61162	2.302585	1	3	2.639057	C38	2019
1.94591	0.571429	14.56641	2.302585	1	3	2.890372	C26	2015
1.94591	0.571429	14.49567	1.791759	1	3	2.944439	C26	2016
1.94591	0.571429	14.68181	2.079442	0	3	2.995732	C26	2017
1.94591	0.571429	14.86787	2.079442	0	3	3.044522	C26	2018
1.94591	0.571429	15.17912	1.609438	0	3	3.091042	C26	2019
2.197225	0.333333	13.24476	1.94591	1	3	2.484907	C40	2015
2.197225	0.333333	13.4198	1.791759	1	3	2.564949	C40	2016
2.197225	0.333333	13.56692	2.397895	1	3	2.639057	C40	2017
2.197225	0.333333	13.7548	1.94591	1	3	2.70805	C40	2018
2.197225	0.333333	13.72318	1.94591	1	3	2.772589	C40	2019
2.197225	0.333333	14.90934	2.197225	1	3	2.890372	C36	2015
2.079442	0.375	14.87031	2.484907	1	3	2.944439	C36	2016
2.197225	0.333333	14.72458	2.079442	1	3	2.995732	C36	2017
2.197225	0.333333	14.57243	2.197225	1	3	3.091042	C36	2019
2.197225	0.333333	14.07015	1.94591	1	2	2.197225	C34	2015
2.079442	0.375	14.07015	1.386294	1	2	2.302585	C34	2016
2.197225	0.333333	14.07248	1.94591	1	2	2.397895	C34	2017
2.197225	0.333333	14.08401	1.94591	1	4	2.484907	C34	2018
2.197225	0.333333	14.08401	2.079442	1	4	2.564949	C34	2019
1.94591	0.428571	13.32449	2.197225	1	4	1.94591	C34	2015
1.94591	0.428571	13.10623	2.833213	1	4	2.079442	C34	2016
1.94591	0.428571	13.71015	2.484907	1	4	2.197225	C34	2017
1.94591	0.428571	13.86905	2.197225	1	4	2.302585	C34	2018
1.94591	0.428571	13.99783	2.197225	1	4	2.397895	C34	2019
1.94591	0.428571	13.25951	1.94591	1	3	2.890372	C35	2015
1.94591	0.428571	13.57293	1.94591	1	3	2.944439	C35	2016
1.94591	0.428571	13.74884	1.791759	0	3	2.995732	C35	2017
1.94591	0.428571	13.76957	2.079442	0	3	3.044522	C35	2018
1.94591	0.428571	14.22098	2.079442	0	4	3.091042	C35	2019
2.197225	0.333333	13.69898	2.484907	1	4	2.079442	C26	2015
2.397895	0.363636	13.93782	2.639057	1	4	2.197225	C26	2016
2.397895	0.363636	14.14345	2.70805	1	4	2.302585	C26	2017
2.397895	0.363636	14.36905	2.890372	1	4	2.397895	C26	2018
2.197225	0.333333	13.86468	2.197225	1	2	2.944439	C40	2015
2.197225	0.333333	13.95022	2.70805	1	2	2.995732	C40	2016
2.197225	0.333333	13.90306	2.70805	1	2	3.044522	C40	2017
1.94591	0.428571	14.75934	1.609438	1	2	2.833213	C38	2015
1.94591	0.428571	13.972	1.609438	1	2	2.890372	C38	2016
1.94591	0.428571	14.2996	1.94591	1	2	2.944439	C38	2017
1.94591	0.428571	13.43571	1.609438	1	2	2.995732	C38	2018

1.94591	0.428571	12.77394	1.94591	1	3	2.564949	C26	2015
1.94591	0.428571	12.55849	1.94591	1	3	2.639057	C26	2016
1.94591	0.428571	13.19729	2.079442	1	3	2.70805	C26	2017
1.94591	0.428571	13.68859	1.791759	1	3	2.772589	C26	2018
1.94591	0.428571	13.74176	2.197225	1	3	2.833213	C26	2019
1.94591	0.428571	13.49255	1.609438	1	1	2.484907	C41	2015
1.94591	0.428571	14.11562	1.791759	1	1	2.564949	C41	2016
1.791759	0.5	13.95527	2.079442	1	1	2.639057	C41	2017
1.609438	0.4	13.96393	2.397895	1	1	2.70805	C41	2018
1.94591	0.428571	14.2302	1.94591	1	1	2.772589	B07	2019
2.079442	0.375	14.50163	1.791759	1	5	2.772589	C27	2015
2.079442	0.375	14.53008	1.609438	1	5	2.833213	C27	2016
2.079442	0.375	14.53481	1.386294	1	3	2.890372	C27	2017
2.079442	0.5	14.5852	2.079442	1	5	2.944439	C27	2018
1.94591	0.428571	14.14056	2.197225	1	5	2.995732	C27	2019
2.197225	0.333333	13.05622	2.197225	1	3	2.995732	C35	2015
1.94591	0.428571	13.90818	2.70805	1	3	3.044522	C35	2016
1.94591	0.428571	13.35348	2.397895	1	3	3.091042	C35	2017
1.94591	0.428571	14.16884	2.397895	1	3	3.135494	C35	2018
1.94591	0.428571	14.46789	2.397895	1	3	3.178054	C35	2019
1.791759	0.5	14.12674	1.94591	1	3	2.995732	C33	2017
1.791759	0.5	14.16898	1.609438	1	3	3.044522	C33	2018
2.197225	0.333333	14.56139	2.197225	1	1	2.079442	C39	2016
1.94591	0.428571	14.53306	2.397895	1	1	2.197225	C39	2017
1.94591	0.428571	14.5543	1.791759	1	4	2.302585	C39	2018
1.94591	0.428571	15.24038	1.386294	1	2	2.397895	C39	2019
2.197225	0.333333	14.32022	2.079442	1	1	2.772589	C39	2017
2.197225	0.333333	14.22044	1.791759	1	1	2.833213	C39	2018
2.197225	0.333333	14.35842	1.609438	1	1	2.890372	C39	2019
1.94591	0.428571	14.59932	2.079442	1	4	2.564949	C35	2016
1.94591	0.428571	14.59909	1.609438	1	1	2.639057	C35	2017
1.94591	0.428571	14.60515	2.397895	1	4	2.70805	C35	2018
1.94591	0.428571	14.73388	1.609438	1	1	2.772589	C35	2019
2.197225	0.333333	13.85953	2.397895	1	1	2.833213	C34	2016
2.197225	0.333333	13.8643	1.791759	1	1	2.890372	C34	2017
2.197225	0.333333	13.87849	2.772589	1	4	2.944439	C34	2018
2.197225	0.333333	13.93773	2.302585	1	1	2.995732	C34	2019
1.609438	0.4	14.85253	2.079442	1	2	2.70805	C39	2017
1.609438	0.4	14.40845	2.079442	1	2	2.772589	C39	2018
1.609438	0.4	14.50686	2.079442	1	3	2.833213	C39	2019
1.94591	0.428571	13.95388	1.94591	1	3	2.484907	C40	2016
1.94591	0.428571	14.00022	1.791759	1	3	2.564949	C40	2017
1.609438	0.4	14.03348	2.197225	1	3	2.639057	C40	2018
1.609438	0.4	14.1569	1.791759	1	3	2.70805	C40	2019
1.609438	0.4	13.8108	2.484907	1	3	2.564949	C35	2016
1.609438	0.4	14.14424	2.302585	1	3	2.639057	C35	2017
1.609438	0.4	14.03167	2.564949	1	3	2.70805	C35	2018
2.197225	0.333333	13.91554	2.564949	1	4	2.302585	C39	2016
2.197225	0.333333	14.11004	2.564949	1	4	2.397895	C39	2017
2.197225	0.333333	14.41165	2.564949	1	4	2.484907	C39	2018
2.197225	0.333333	14.40674	2.564949	1	4	2.564949	C39	2019
2.197225	0.333333	13.41414	1.791759	1	2	2.772589	E48	2016
2.197225	0.333333	14.01624	2.302585	1	2	2.833213	E48	2017
2.197225	0.333333	14.25808	2.484907	1	2	2.890372	E48	2018
2.197225	0.333333	14.20077	2.397895	1	2	2.944439	E48	2019
1.94591	0.428571	13.18628	2.197225	1	1	2.70805	C29	2016
1.94591	0.428571	13.72723	1.791759	1	1	2.772589	C29	2017
1.94591	0.428571	13.73169	2.079442	1	1	2.833213	C29	2018

1.94591	0.428571	13.76264	1.791759	1	1	2.890372	C29	2019
2.197225	0.333333	14.30905	1.791759	1	3	3.044522	C39	2016
2.197225	0.333333	14.51861	2.302585	1	3	3.091042	C39	2017
2.197225	0.333333	14.62252	2.302585	1	3	3.135494	C39	2018
2.197225	0.333333	14.69634	2.079442	1	4	3.178054	C39	2019
2.079442	0.375	12.90644	1.609438	1	4	2.639057	C29	2016
2.197225	0.333333	13.2752	2.197225	1	4	2.70805	C29	2017
2.197225	0.333333	13.33035	2.079442	1	4	2.772589	C29	2018
2.197225	0.333333	13.42867	1.609438	1	4	2.833213	C29	2019
2.197225	0.333333	13.76	1.609438	1	3	1.791759	I65	2016
2.197225	0.333333	13.84313	1.386294	1	5	1.94591	I65	2017
2.197225	0.333333	14.18431	2.079442	1	3	2.079442	I65	2018
1.94591	0.428571	14.61706	2.397895	1	5	2.197225	I65	2019
1.94591	0.428571	14.06448	1.94591	1	3	2.564949	C39	2016
1.94591	0.428571	14.04662	1.94591	1	3	2.639057	C39	2017
1.94591	0.428571	14.02252	1.609438	1	3	2.70805	C39	2018
1.94591	0.428571	14.4307	2.197225	1	3	2.772589	C39	2019
1.609438	0.4	14.35265	2.397895	1	2	2.995732	C36	2017
1.609438	0.4	14.40485	2.302585	1	2	3.044522	C36	2018
1.609438	0.4	14.35935	1.791759	1	2	3.091042	C36	2019
1.94591	0.428571	14.50641	1.94591	1	3	2.772589	C39	2016
1.94591	0.428571	14.54243	1.791759	1	3	2.833213	C39	2017
1.94591	0.428571	14.61239	2.079442	1	3	2.890372	C39	2018
1.94591	0.428571	14.67781	1.791759	1	3	2.944439	C39	2019
1.94591	0.428571	14.6557	2.564949	1	3	1.94591	C27	2017
1.94591	0.428571	13.83962	2.079442	1	3	2.079442	C27	2018
1.94591	0.428571	14.63591	1.94591	1	3	2.197225	C27	2019
1.94591	0.428571	13.99783	1.098612	0	3	2.564949	C29	2016
1.94591	0.428571	13.97251	2.079442	0	3	2.639057	C29	2017
1.94591	0.428571	14.02252	2.397895	1	3	2.70805	C29	2018
1.94591	0.428571	14.09102	2.197225	1	3	2.772589	C29	2019
1.94591	0.428571	13.87773	2.197225	1	3	2.833213	I65	2016
1.94591	0.428571	13.79306	2.197225	1	2	2.890372	I65	2017
1.94591	0.428571	14.20661	2.397895	1	3	2.944439	I65	2018
1.94591	0.428571	14.3088	2.302585	1	3	2.995732	I65	2019
1.94591	0.428571	13.4531	1.94591	1	1	2.70805	C19	2017
1.94591	0.428571	13.4531	2.197225	1	1	2.772589	C19	2018
1.94591	0.428571	13.67625	2.197225	1	1	2.833213	C19	2019
2.197225	0.333333	14.15084	1.098612	1	1	2.564949	C29	2016
2.197225	0.333333	14.57416	1.791759	1	1	2.639057	C29	2017
2.197225	0.333333	14.96924	1.609438	1	1	2.70805	C29	2018
2.197225	0.333333	14.7387	1.94591	1	1	2.772589	C29	2019
2.197225	0.333333	13.6218	1.791759	1	3	2.484907	C30	2016
2.197225	0.333333	13.88755	2.079442	1	3	2.564949	C30	2017
2.197225	0.333333	13.80243	1.791759	1	3	2.639057	C30	2018
2.197225	0.333333	13.84856	1.609438	1	3	2.70805	C30	2019
2.197225	0.333333	14.12688	2.197225	1	3	3.091042	C32	2019
2.079442	0.375	14.35731	1.791759	1	3	3.218876	E50	2019
2.197225	0.333333	14.506	2.302585	1	1	3.091042	C39	2017
2.197225	0.333333	14.59015	2.079442	1	1	3.135494	C39	2018
2.197225	0.333333	14.61509	2.484907	1	3	3.178054	C39	2019
2.197225	0.333333	13.86173	1.791759	0	3	2.944439	C33	2017
1.94591	0.428571	14.71225	2.397895	1	4	2.70805	C26	2017
1.94591	0.428571	14.91619	2.079442	1	4	2.772589	C26	2018
1.94591	0.428571	14.62341	2.197225	1	4	2.833213	C26	2019
2.397895	0.363636	15.25537	1.94591	1	4	2.639057	C27	2017
2.397895	0.363636	15.33448	1.94591	1	4	2.70805	C27	2018
2.197225	0.333333	15.47248	1.94591	1	4	2.772589	C27	2019

2.197225	0.333333	14.0723	1.609438	1	2	3.178054	C36	2017
1.94591	0.428571	14.10204	1.791759	1	2	3.218876	C36	2018
1.94591	0.428571	14.10474	1.94591	1	2	3.258097	C36	2019
1.94591	0.428571	14.03905	2.484907	1	4	2.944439	C29	2018
1.94591	0.428571	14.12674	2.302585	1	4	2.995732	C29	2019
2.079442	0.375	13.92142	2.197225	1	2	2.564949	C24	2017
1.94591	0.428571	13.92248	2.397895	1	3	2.639057	C24	2018
2.197225	0.333333	13.92607	2.397895	1	3	2.70805	C24	2019
1.94591	0.428571	14.28695	2.079442	1	1	2.70805	C36	2017
1.94591	0.428571	14.25441	1.94591	1	1	2.772589	C36	2018
1.94591	0.428571	14.43026	1.94591	1	1	2.833213	C36	2019
2.197225	0.333333	13.80232	1.791759	1	3	2.564949	R85	2017
2.197225	0.333333	13.99883	1.609438	1	3	2.639057	R85	2018
2.197225	0.333333	14.08607	1.386294	1	3	2.70805	R85	2019
1.94591	0.428571	13.6118	2.302585	1	3	2.564949	C39	2017
1.94591	0.428571	13.64151	2.302585	1	3	2.639057	C39	2018
1.94591	0.428571	13.65546	2.079442	1	3	2.70805	C39	2019
2.197225	0.333333	15.57175	1.94591	0	4	2.833213	C17	2017
2.197225	0.333333	15.73442	1.94591	0	3	2.890372	C17	2018
2.197225	0.333333	15.72812	2.197225	0	4	2.944439	C17	2019
2.197225	0.333333	14.45277	2.397895	1	1	2.397895	C38	2017
2.197225	0.333333	14.6906	2.302585	1	4	2.484907	C38	2018
2.197225	0.333333	14.72381	2.079442	1	4	2.564949	C38	2019
1.94591	0.428571	13.48116	1.94591	1	2	2.564949	C36	2017
1.94591	0.428571	13.48839	2.397895	1	2	2.639057	C36	2018
1.94591	0.428571	13.65064	2.079442	1	3	2.70805	C36	2019
2.197225	0.333333	14.07155	1.609438	1	4	3.218876	C27	2017
2.197225	0.333333	14.07124	2.302585	1	4	3.258097	C27	2018
2.197225	0.333333	14.2243	1.791759	1	4	3.295837	C27	2019
2.197225	0.333333	15.05493	1.791759	1	3	2.995732	C26	2017
2.197225	0.333333	14.97738	1.94591	1	3	3.044522	C26	2018
2.197225	0.333333	14.72417	2.564949	1	3	3.091042	C26	2019
1.609438	0.4	13.57776	1.609438	1	1	2.397895	C39	2017
1.609438	0.4	13.69176	1.94591	1	1	2.484907	C39	2018
1.609438	0.4	13.57636	1.386294	1	1	2.564949	C39	2019
2.197225	0.333333	14.65785	1.609438	1	2	2.833213	C30	2017
2.197225	0.333333	14.58302	1.94591	1	2	2.890372	C30	2018
2.197225	0.333333	14.6853	1.791759	1	2	2.944439	C30	2019
2.197225	0.333333	15.00033	1.791759	1	2	3.178054	C34	2018
2.197225	0.333333	15.14035	2.079442	1	3	3.218876	C34	2019
2.197225	0.333333	13.98102	0.693147	1	3	2.995732	C39	2017
2.197225	0.333333	13.57089	2.079442	1	3	3.044522	C39	2018
2.197225	0.333333	14.10232	1.609438	1	3	3.091042	C39	2019
2.197225	0.333333	14.05893	2.197225	0	1	3.218876	C28	2017
2.197225	0.333333	14.28075	2.079442	0	1	3.258097	C28	2018
2.197225	0.333333	14.84584	1.791759	0	1	3.295837	C28	2019
1.94591	0.428571	14.85427	1.609438	1	3	2.564949	C39	2017
1.94591	0.428571	14.49729	1.791759	1	3	2.639057	C39	2018
1.94591	0.428571	15.15921	2.079442	1	4	2.70805	C39	2019
2.197225	0.333333	13.8856	1.791759	1	1	2.772589	C39	2017
2.197225	0.333333	13.88494	2.197225	1	1	2.833213	C39	2018
2.197225	0.333333	13.77156	2.197225	1	1	2.890372	C39	2019
1.94591	0.428571	13.7356	1.386294	1	4	1.791759	C30	2017
1.94591	0.428571	14.30764	2.302585	1	4	1.94591	C30	2018
1.94591	0.428571	14.31349	2.484907	1	4	2.079442	C30	2019
2.197225	0.333333	13.4198	1.791759	1	3	2.890372	C29	2017
1.94591	0.428571	13.52114	2.079442	1	3	2.944439	C29	2018
2.197225	0.444444	13.63243	1.791759	1	3	2.995732	C29	2019

2.197225	0.444444	13.83805	1.94591	1	4	3.091042	C34	2017
2.197225	0.444444	14.08554	1.94591	1	4	3.135494	C34	2018
1.94591	0.428571	14.08577	1.386294	1	4	3.178054	C34	2019
2.079442	0.375	14.85512	2.484907	1	3	2.772589	C37	2017
2.079442	0.375	14.72191	2.302585	1	3	2.833213	C37	2018
2.079442	0.375	14.53554	2.484907	1	3	2.890372	C37	2019
1.94591	0.428571	14.38914	1.791759	1	1	3.044522	C26	2017
1.94591	0.428571	14.08813	2.197225	1	4	3.091042	C26	2018
1.94591	0.428571	13.96729	2.197225	1	1	3.135494	C26	2019
2.197225	0.333333	15.18084	2.079442	0	3	2.397895	C35	2018
2.197225	0.333333	15.32899	1.94591	0	3	2.484907	C38	2019
2.197225	0.333333	14.58691	2.302585	1	1	2.944439	C36	2018
2.197225	0.333333	14.51081	2.197225	1	1	2.995732	C36	2019
2.197225	0.333333	14.10129	2.302585	1	3	2.484907	C26	2018
2.197225	0.333333	14.55354	1.94591	1	4	2.564949	C26	2019
1.94591	0.428571	14.22908	2.302585	1	4	2.639057	C38	2019
1.609438	0.4	15.01086	2.302585	1	3	2.833213	M74	2019
2.197225	0.333333	14.96738	2.639057	1	3	2.833213	L72	2019
1.94591	0.428571	14.78652	2.397895	0	2	2.197225	I65	2019
2.197225	0.333333	14.17675	2.302585	1	2	1.791759	C39	2019
1.94591	0.428571	14.52507	2.302585	1	4	2.484907	I65	2019
1.609438	0.4	16.08305	1.791759	1	2	2.564949	C26	2019
1.94591	0.428571	14.20383	1.94591	0	3	2.70805	C39	2019
2.197225	0.333333	14.39302	0.693147	1	1	2.944439	C34	2019
2.197225	0.444444	13.7223	2.302585	1	3	2.197225	C35	2009
2.397895	0.363636	15.74674	2.397895	1	3	2.484907	C35	2012
2.197225	0.333333	16.15635	1.94591	1	3	2.564949	C35	2013
2.197225	0.333333	16.24684	2.302585	1	3	2.639057	C35	2014
2.197225	0.333333	15.62341	1.94591	1	3	2.70805	C35	2015
2.197225	0.333333	15.68605	2.833213	1	3	2.772589	C35	2016
2.302585	0.4	15.41441	2.70805	1	3	2.833213	C35	2017
2.302585	0.4	17.0205	2.302585	1	3	2.890372	C35	2018
2.302585	0.4	17.74573	2.564949	1	3	2.944439	C35	2019
2.197225	0.333333	14.59488	2.079442	1	3	2.772589	S90	2010
1.609438	0.4	14.90995	1.609438	1	3	2.833213	S90	2011
1.609438	0.4	15.09017	1.94591	1	3	2.890372	F51	2012
1.609438	0.4	14.48139	1.609438	1	3	2.944439	F51	2013
1.609438	0.4	14.70048	2.079442	1	3	2.995732	F51	2014
1.609438	0.4	15.00695	1.791759	1	3	3.044522	F51	2015
1.609438	0.4	15.01505	1.609438	1	3	3.091042	F51	2016
1.609438	0.4	15.10873	1.791759	1	3	3.135494	F51	2017
1.94591	0.428571	15.14613	2.197225	1	3	3.178054	F51	2018
1.94591	0.428571	15.37656	1.609438	1	3	3.218876	F51	2019
1.94591	0.428571	12.99271	2.079442	1	5	2.772589	K70	2009
2.197225	0.333333	12.88993	2.079442	1	5	2.833213	K70	2010
2.197225	0.333333	13.32121	2.564949	1	5	2.890372	K70	2011
2.197225	0.333333	12.78549	2.484907	1	5	2.944439	K70	2012
2.197225	0.333333	13.38626	2.302585	1	5	2.995732	K70	2013
2.197225	0.333333	13.3487	2.484907	1	5	3.044522	K70	2014
2.079442	0.375	13.68095	2.079442	1	5	3.091042	K70	2015
2.197225	0.333333	13.72208	2.079442	1	5	3.135494	K70	2016
2.197225	0.333333	13.61462	2.197225	1	5	3.178054	K70	2017
2.197225	0.333333	13.57573	2.397895	1	5	3.218876	K70	2018
2.197225	0.333333	14.90458	2.564949	1	4	2.890372	C35	2015
2.197225	0.333333	14.90053	2.079442	1	4	2.944439	C35	2016
2.197225	0.333333	14.62986	1.791759	1	4	2.995732	C35	2017
2.197225	0.333333	14.62243	1.94591	1	4	3.044522	C35	2018
2.197225	0.333333	14.72232	1.609438	1	4	3.091042	C35	2019

2.197225	0.333333	13.68015	2.833213	1	3	3.044522	C38	2009
2.197225	0.333333	14.29206	3.044522	1	3	3.091042	C38	2010
2.197225	0.333333	14.37227	2.772589	1	3	3.135494	C38	2011
2.197225	0.333333	14.37227	2.564949	1	3	3.178054	K70	2012
2.197225	0.333333	14.37227	3.091042	1	3	3.218876	K70	2013
2.197225	0.333333	14.37227	2.772589	1	3	3.258097	K70	2014
2.197225	0.333333	14.37227	3.178054	1	3	3.295837	K70	2015
2.079442	0.375	14.3694	2.70805	1	3	3.332205	K70	2016
2.197225	0.333333	14.48298	2.484907	1	3	3.367296	K70	2017
2.197225	0.333333	14.81603	2.484907	1	3	3.401197	K70	2018
2.197225	0.333333	15.06916	2.639057	1	3	3.433987	K70	2019
1.94591	0.428571	13.5176	2.564949	0	3	3.091042	C39	2015
1.94591	0.428571	13.73647	2.079442	0	3	3.135494	C20	2016
1.94591	0.428571	13.9622	2.302585	0	3	3.178054	C20	2017
1.94591	0.428571	13.98102	2.484907	0	3	3.218876	C20	2018
1.94591	0.428571	14.32642	2.197225	1	4	2.890372	C39	2011
1.94591	0.428571	15.11842	2.772589	1	4	2.944439	K70	2012
1.94591	0.428571	15.11842	2.944439	1	4	2.995732	K70	2013
1.94591	0.428571	15.11842	2.564949	1	4	3.044522	K70	2014
1.791759	0.5	14.91412	2.302585	1	4	3.091042	K70	2015
1.94591	0.428571	15.17135	2.564949	1	4	3.135494	K70	2016
1.94591	0.428571	15.17135	2.772589	1	4	3.178054	K70	2017
1.94591	0.428571	15.17135	2.70805	1	4	3.218876	K70	2018
1.94591	0.428571	15.17135	2.197225	1	4	3.258097	K70	2019
2.197225	0.333333	12.67608	2.564949	1	2	2.70805	C26	2009
2.197225	0.333333	12.67608	2.833213	1	2	2.772589	C26	2010
2.197225	0.333333	12.79386	2.197225	1	2	2.833213	C26	2011
2.197225	0.333333	13.27078	2.397895	1	2	2.890372	C26	2012
2.197225	0.333333	13.32121	1.791759	1	2	2.944439	C26	2013
2.197225	0.333333	13.30468	2.079442	1	2	2.995732	C26	2014
2.197225	0.333333	13.30468	2.397895	1	2	3.044522	C26	2015
2.197225	0.333333	14.05932	2.197225	1	2	3.091042	C26	2016
2.197225	0.333333	14.13839	2.079442	1	2	3.135494	C26	2017
2.197225	0.333333	13.5391	2.639057	1	2	3.178054	C26	2018
1.609438	0.4	14.42853	1.94591	1	3	2.772589	C41	2009
1.791759	0.333333	14.60687	1.609438	1	3	2.833213	C41	2010
1.791759	0.333333	14.96637	2.079442	1	3	2.890372	C41	2011
1.609438	0.4	13.48701	1.94591	1	3	2.944439	C41	2012
1.609438	0.4	13.75119	2.197225	1	3	2.995732	C41	2013
1.609438	0.4	13.99408	2.197225	1	3	3.044522	C41	2014
1.609438	0.4	14.36056	2.70805	1	3	3.091042	C41	2015
2.197225	0.444444	14.79725	2.484907	1	3	2.772589	K70	2012
2.197225	0.444444	15.13513	2.484907	1	3	2.833213	K70	2013
2.197225	0.444444	15.62596	2.484907	1	3	2.890372	K70	2014
2.197225	0.444444	15.59873	2.484907	1	3	2.944439	K70	2015
2.079442	0.375	15.10887	2.484907	1	3	2.995732	K70	2016
2.197225	0.333333	15.29848	2.484907	1	3	3.044522	K70	2017
2.197225	0.333333	15.42295	2.484907	1	3	3.091042	K70	2018
2.197225	0.333333	15.64354	2.484907	1	3	3.135494	K70	2019
1.791759	0.5	13.65299	1.609438	1	2	2.70805	C39	2009
1.791759	0.5	14.0286	1.94591	1	2	2.772589	C39	2010
1.791759	0.5	14.28551	1.94591	1	2	2.833213	C39	2011
1.791759	0.5	14.28551	2.079442	1	2	2.890372	C38	2012
1.609438	0.4	14.50866	2.302585	1	2	2.944439	C38	2013
1.791759	0.333333	14.45999	2.397895	1	2	2.995732	C38	2014
1.791759	0.333333	14.49861	2.197225	1	2	3.044522	C38	2015
1.791759	0.333333	14.49861	2.397895	1	3	3.091042	C38	2016
1.791759	0.333333	14.352	2.197225	1	3	3.135494	C38	2017

1.791759	0.333333	14.42712	2.564949	1	3	3.178054	C38		2018
1.791759	0.333333	14.53252	2.397895	1	3	3.218876	C38		2019
1.609438	0.4	12.3519	1.386294	1	3	2.564949	C18		2009
1.609438	0.4	12.70806	1.791759	1	3	2.639057	C18		2010
1.609438	0.4	12.77931	1.791759	1	3	2.70805	C18		2011
1.609438	0.4	12.878	1.791759	1	3	2.772589	C18		2012
1.609438	0.4	13.03898	2.079442	1	3	2.833213	C18		2013
1.609438	0.4	13.06049	1.386294	1	3	2.890372	C18		2014
1.609438	0.4	13.15192	1.791759	1	3	2.944439	C18		2015
1.94591	0.428571	13.16158	2.079442	1	3	2.995732	C18		2016
1.94591	0.428571	13.19932	1.609438	1	3	3.044522	C18		2017
1.94591	0.428571	13.19932	1.791759	1	3	3.091042	C18		2018
1.94591	0.428571	13.38473	1.791759	1	3	3.135494	C18		2019
2.197225	0.444444	14.55878	1.94591	1	4	2.079442	C29		2009
2.397895	0.363636	14.54311	2.302585	1	4	2.197225	C29		2010
2.397895	0.363636	14.60397	2.302585	1	4	2.302585	C29		2011
2.397895	0.363636	14.60397	1.94591	1	4	2.397895	C29		2012
2.397895	0.363636	14.60397	2.197225	1	4	2.484907	C29		2013
2.397895	0.363636	14.69928	1.94591	1	4	2.564949	C29		2014
2.397895	0.363636	14.97487	2.079442	1	4	2.639057	C29		2015
2.397895	0.363636	15.37903	2.079442	1	4	2.70805	C29		2016
2.397895	0.363636	15.17258	2.397895	1	4	2.772589	C29		2017
2.397895	0.363636	15.19433	2.079442	1	4	2.833213	C29		2018
2.397895	0.363636	15.27361	2.197225	1	4	2.890372	C29		2019
2.079442	0.375	12.93241	2.484907	1	2	2.772589	C29		2014
2.079442	0.375	15.09728	2.397895	1	4	2.70805	K70		2009
1.94591	0.428571	15.18135	2.397895	1	4	2.772589	K70		2010
1.94591	0.428571	15.40297	2.197225	1	4	2.833213	K70		2011
1.94591	0.428571	15.21937	2.079442	1	4	2.890372	K70		2012
1.94591	0.428571	15.25842	2.397895	1	4	2.944439	K70		2013
1.94591	0.428571	15.23088	2.079442	1	4	2.995732	K70		2014
2.079442	0.375	14.91987	2.564949	1	4	3.044522	K70		2015
2.197225	0.333333	15.0605	2.397895	1	4	3.091042	K70		2016
2.197225	0.333333	15.11013	2.079442	1	4	3.135494	K70		2017
2.197225	0.333333	15.07986	2.302585	1	4	3.178054	K70		2018
2.197225	0.333333	15.3285	2.197225	1	4	3.218876	K70		2019
1.94591	0.428571	12.59473	1.386294	0	1	2.302585	D44		2009
1.94591	0.428571	12.96055	1.098612	0	1	2.397895	D44		2010
1.94591	0.428571	12.99839	1.386294	0	1	2.484907	D44		2011
1.94591	0.428571	12.94252	1.609438	0	1	2.564949	D44		2012
1.94591	0.428571	12.97942	1.098612	0	1	2.639057	D44		2013
1.94591	0.428571	12.97872	1.609438	0	1	2.70805	D44		2014
1.94591	0.428571	13.00628	1.94591	0	1	2.772589	D44		2015
2.197225	0.333333	13.91987	2.70805	1	4	2.772589	C30		2009
2.197225	0.333333	13.91987	2.772589	1	4	2.833213	K70		2010
2.197225	0.333333	14.07015	2.944439	1	4	2.890372	K70		2011
2.197225	0.333333	14.07015	2.397895	1	4	2.944439	K70		2012
2.197225	0.333333	14.11562	2.302585	1	4	2.995732	K70		2013
2.197225	0.333333	14.18015	2.197225	1	4	3.044522	K70		2014
2.197225	0.333333	14.18015	2.079442	1	4	3.091042	K70		2015
2.197225	0.333333	14.20077	2.197225	1	4	3.135494	K70		2016
2.197225	0.333333	14.22098	2.564949	1	4	3.178054	K70		2017
2.197225	0.333333	14.76609	2.70805	1	4	3.218876	K70		2018
2.197225	0.333333	14.8622	2.079442	1	4	3.258097	K70		2019
2.70805	0.333333	15.19206	2.197225	1	2	2.772589	C18		2009
2.70805	0.333333	15.63271	2.079442	1	2	2.833213	C18		2010
2.484907	0.416667	15.71614	2.397895	1	2	2.890372	C18		2011
2.484907	0.416667	15.69754	1.791759	1	2	2.944439	C18		2012

2.484907	0.416667	15.67345	2.302585	1	2	2.995732	C18	2013
2.197225	0.333333	16.12647	2.397895	1	2	3.044522	C18	2014
1.94591	0.428571	16.02107	2.484907	1	2	3.091042	K70	2015
1.94591	0.428571	15.9811	2.197225	1	2	3.135494	K70	2016
2.197225	0.333333	15.94538	2.197225	1	2	3.178054	K70	2017
2.197225	0.333333	16.24531	2.302585	1	2	3.218876	K70	2018
2.197225	0.333333	16.14746	1.791759	1	2	3.258097	C18	2019
2.197225	0.333333	14.35127	2.079442	1	3	2.564949	S90	2009
2.079442	0.375	13.73452	1.94591	1	3	2.639057	S90	2010
1.94591	0.428571	13.65886	2.302585	1	3	2.70805	S90	2011
1.94591	0.428571	13.27044	1.94591	1	3	2.772589	K70	2012
1.94591	0.428571	13.60516	1.94591	1	3	2.833213	B08	2013
1.609438	0.4	13.50926	2.484907	1	3	2.944439	B08	2015
2.197225	0.333333	15.10254	2.079442	1	2	2.302585	C41	2009
2.197225	0.333333	15.33086	2.302585	1	2	2.397895	C41	2010
2.197225	0.333333	15.32866	2.484907	1	2	2.484907	C41	2011
2.197225	0.333333	15.19955	1.791759	1	2	2.564949	C29	2012
2.197225	0.333333	15.41792	2.079442	1	2	2.639057	C29	2013
2.197225	0.333333	15.44318	1.94591	1	2	2.70805	C29	2014
2.197225	0.333333	15.39304	2.079442	1	2	2.772589	C29	2015
2.197225	0.333333	15.51442	1.94591	1	2	2.833213	C29	2016
2.197225	0.333333	16.05366	2.197225	1	2	2.890372	C29	2017
2.197225	0.333333	16.09541	1.609438	1	2	2.944439	C29	2018
2.197225	0.333333	15.91776	1.386294	1	2	2.995732	C29	2019
2.197225	0.333333	13.85713	2.302585	0	1	3.218876	K70	2018
2.197225	0.333333	14.21944	1.791759	0	1	3.258097	K70	2019
2.397895	0.363636	14.35247	1.791759	1	3	2.890372	C27	2015
2.397895	0.363636	14.60474	1.94591	1	3	2.944439	C27	2016
2.397895	0.363636	14.84752	1.94591	1	3	2.995732	C27	2017
2.397895	0.363636	14.88384	1.791759	1	3	3.044522	C27	2018
2.397895	0.363636	15.01557	1.791759	1	3	3.091042	C27	2019
2.197225	0.333333	13.83531	1.791759	1	1	2.639057	C17	2013
2.197225	0.333333	13.83531	2.079442	1	1	2.70805	C17	2014
2.197225	0.333333	14.64842	1.791759	1	1	2.772589	C17	2015
2.197225	0.333333	14.93471	2.079442	1	1	2.833213	C17	2016
2.079442	0.375	14.75759	1.791759	1	1	2.890372	C17	2017
1.791759	0.5	14.63322	1.791759	1	1	2.944439	C17	2018
1.94591	0.428571	14.63701	1.94591	1	1	2.995732	C17	2019
2.197225	0.333333	13.20615	2.70805	1	3	2.772589	C26	2014
2.197225	0.333333	13.33473	1.94591	1	3	2.833213	C26	2015
2.197225	0.333333	13.34391	2.70805	1	3	2.944439	C27	2017
2.079442	0.375	13.51441	2.772589	1	3	2.995732	C27	2018
2.197225	0.333333	14.19127	2.564949	1	3	2.890372	G55	2011
2.197225	0.333333	14.13257	2.302585	1	3	2.944439	G55	2012
2.197225	0.333333	14.07502	2.079442	1	3	2.995732	G55	2013
2.197225	0.333333	14.47665	2.639057	1	3	3.135494	G55	2016
2.079442	0.375	14.37918	2.833213	1	3	3.178054	I64	2017
2.197225	0.333333	14.85893	2.484907	1	3	3.218876	I64	2018
2.197225	0.333333	15.2668	2.302585	1	3	2.772589	C27	2009
2.197225	0.333333	15.05822	2.302585	1	3	2.833213	C27	2010
2.197225	0.333333	15.04515	2.079442	1	3	2.890372	C27	2011
2.197225	0.333333	15.04515	2.079442	1	3	2.944439	C27	2012
2.197225	0.333333	15.15978	2.079442	1	3	2.995732	C27	2013
2.197225	0.333333	15.49401	2.484907	1	3	3.091042	C27	2015
1.609438	0.4	13.7212	1.791759	1	4	2.397895	A05	2010
1.609438	0.4	13.65299	2.639057	1	4	2.484907	A05	2011
1.609438	0.4	13.88317	2.484907	1	4	2.564949	A04	2012
1.609438	0.4	13.88317	2.079442	1	4	2.639057	A04	2013

1.609438	0.4	13.45884	2.564949	1	4	2.70805	A04	2014
1.609438	0.4	13.51441	2.484907	1	4	2.772589	A04	2015
1.609438	0.4	13.48701	1.94591	1	4	2.833213	A04	2016
1.609438	0.4	13.90471	2.70805	1	4	2.890372	A04	2017
1.609438	0.4	13.81631	1.94591	1	4	2.944439	A04	2018
1.609438	0.4	13.84507	1.609438	1	4	2.995732	A04	2019
2.079442	0.375	14.63969	2.197225	1	3	2.484907	C38	2009
2.079442	0.375	14.81981	2.302585	1	3	2.564949	C38	2010
2.197225	0.333333	14.77773	2.302585	1	3	2.639057	C38	2011
2.197225	0.333333	14.70134	2.079442	1	3	2.70805	C38	2012
2.197225	0.333333	14.5773	1.609438	1	3	2.772589	C38	2013
2.197225	0.333333	14.94778	1.791759	1	3	2.833213	C38	2014
2.197225	0.333333	15.49378	1.386294	1	3	2.890372	C38	2015
2.197225	0.333333	15.07647	1.791759	1	3	2.944439	C38	2016
2.197225	0.333333	15.21183	1.609438	1	3	2.995732	C38	2017
2.197225	0.333333	15.13588	1.609438	1	3	3.044522	C38	2018
2.079442	0.375	15.20796	1.609438	1	3	3.091042	C38	2019
2.397895	0.363636	14.07278	2.079442	1	3	2.890372	F52	2009
2.397895	0.363636	14.46303	2.302585	1	3	2.944439	F52	2010
2.397895	0.363636	14.26442	2.079442	1	3	2.995732	F52	2011
2.302585	0.4	14.65996	2.397895	1	3	3.044522	F52	2012
2.302585	0.4	14.63969	2.397895	1	3	3.091042	F52	2013
2.397895	0.363636	14.59024	2.484907	1	3	3.135494	F52	2014
2.302585	0.4	14.73579	2.197225	1	3	3.178054	F52	2015
2.079442	0.375	15.33714	1.94591	1	3	3.218876	F52	2016
2.197225	0.333333	15.36037	2.639057	1	3	3.258097	F52	2017
2.079442	0.375	15.94076	2.639057	1	3	3.295837	F52	2018
2.079442	0.375	15.32389	2.197225	1	3	3.332205	F52	2019
2.197225	0.333333	13.83188	1.94591	1	4	2.484907	C27	2011
2.197225	0.333333	13.99173	2.079442	1	4	2.564949	C27	2012
2.197225	0.333333	14.09125	2.197225	1	4	2.639057	C27	2013
2.197225	0.333333	13.9893	1.791759	1	4	2.70805	C27	2014
2.197225	0.333333	14.30311	1.609438	1	4	2.772589	C27	2015
2.197225	0.333333	14.7253	2.079442	1	4	2.833213	C27	2016
2.197225	0.333333	14.80286	1.609438	1	4	2.890372	C27	2017
2.197225	0.333333	14.91336	1.94591	1	4	2.944439	C27	2018
2.197225	0.333333	15.01128	1.791759	1	4	2.995732	C27	2019
2.197225	0.333333	13.52756	2.079442	1	3	2.397895	C35	2009
2.197225	0.333333	13.37312	2.484907	1	3	2.484907	C35	2010
2.197225	0.333333	13.34934	2.564949	1	3	2.564949	C35	2011
2.197225	0.333333	13.26577	2.484907	1	3	2.639057	C38	2012
2.197225	0.333333	13.3739	2.197225	1	3	2.70805	C38	2013
2.197225	0.333333	13.3495	2.564949	1	3	2.772589	C38	2014
2.197225	0.333333	13.48185	2.197225	1	3	2.833213	C38	2015
2.197225	0.333333	14.08095	2.564949	1	3	2.890372	C38	2016
2.079442	0.375	14.47815	2.484907	1	3	2.944439	C38	2017
2.197225	0.333333	14.32269	2.484907	1	3	2.995732	C38	2018
2.197225	0.333333	14.33192	2.564949	1	2	2.890372	C31	2013
2.197225	0.333333	14.33192	2.70805	1	2	2.944439	C31	2014
2.197225	0.333333	14.04607	2.484907	1	2	2.995732	C31	2015
2.197225	0.333333	14.33114	2.484907	1	2	3.044522	C31	2016
2.197225	0.333333	14.15198	2.772589	1	2	3.091042	C31	2017
2.197225	0.333333	14.77987	2.197225	1	2	3.135494	C31	2018
2.079442	0.375	15.57225	2.079442	1	2	3.178054	C31	2019
2.197225	0.333333	14.01436	2.079442	1	4	2.639057	C36	2009
2.197225	0.333333	14.01436	2.197225	1	4	2.70805	C36	2010
2.079442	0.375	14.71282	2.197225	1	4	2.772589	C36	2011
2.197225	0.333333	14.07787	2.484907	1	4	2.833213	C36	2012

2.197225	0.333333	13.78577	1.791759	1	4	2.890372	C36	2013
2.079442	0.375	14.08874	2.079442	1	4	2.944439	C36	2014
2.079442	0.375	14.19627	2.639057	1	4	2.995732	C36	2015
2.197225	0.333333	14.20607	2.833213	1	4	3.044522	C36	2016
2.197225	0.333333	14.46052	2.079442	1	4	3.091042	C36	2017
2.197225	0.333333	14.60169	1.94591	1	3	3.135494	C36	2018
2.197225	0.333333	14.75766	2.397895	1	3	3.178054	C36	2019
2.197225	0.333333	14.2089	2.197225	1	4	2.890372	F52	2017
2.197225	0.333333	13.79623	2.564949	1	4	2.944439	F52	2018
2.197225	0.333333	13.44445	2.397895	1	3	2.772589	C22	2009
2.197225	0.333333	13.35348	2.079442	1	3	2.833213	C22	2010
2.197225	0.333333	13.35348	2.079442	1	3	2.890372	C22	2011
2.197225	0.333333	13.25339	2.079442	1	3	2.944439	C22	2012
2.197225	0.333333	13.25339	1.791759	1	3	2.995732	C22	2013
2.197225	0.333333	13.28788	1.791759	1	3	3.044522	C22	2014
2.197225	0.333333	13.28788	1.94591	1	3	3.091042	C22	2015
2.197225	0.333333	13.44445	1.94591	1	3	3.135494	C22	2016
2.197225	0.333333	13.56705	1.609438	1	3	3.178054	C22	2017
2.197225	0.333333	13.56705	1.94591	1	3	3.218876	C22	2018
2.197225	0.333333	13.78557	2.079442	1	3	3.258097	C22	2019
2.197225	0.333333	12.99861	1.609438	1	4	2.772589	C20	2009
2.197225	0.333333	13.41159	2.197225	1	4	2.833213	C20	2010
2.197225	0.333333	12.93555	2.639057	1	4	2.890372	C20	2011
2.197225	0.333333	13.05258	1.94591	1	4	2.944439	C20	2012
2.197225	0.333333	13.12496	2.079442	1	4	2.995732	C20	2013
2.079442	0.375	13.19431	2.197225	1	4	3.044522	C20	2014
2.197225	0.333333	13.51832	2.079442	1	4	3.091042	C20	2015
2.197225	0.333333	14.64284	1.609438	1	1	2.302585	F52	2009
2.079442	0.375	14.77248	1.94591	1	1	2.397895	F52	2010
2.197225	0.333333	15.09163	1.94591	1	1	2.484907	F52	2011
2.197225	0.333333	14.91795	1.609438	1	1	2.564949	F52	2012
2.197225	0.333333	14.89463	1.609438	1	1	2.639057	F52	2013
2.197225	0.333333	14.26161	2.70805	1	4	2.70805	C32	2009
2.197225	0.333333	13.87208	2.302585	1	4	2.772589	C32	2010
2.197225	0.333333	14.6768	2.397895	1	4	2.833213	C32	2011
2.197225	0.333333	14.7318	1.94591	1	4	2.890372	C32	2012
2.197225	0.333333	14.77102	2.70805	1	4	2.944439	C32	2013
2.197225	0.333333	14.60032	2.397895	1	4	2.995732	C32	2014
2.197225	0.333333	14.7318	2.302585	1	4	3.044522	C32	2015
2.197225	0.333333	14.7318	1.791759	1	4	3.091042	C32	2016
2.197225	0.333333	14.7318	2.302585	1	4	3.135494	C32	2017
2.197225	0.333333	14.7318	1.098612	1	4	3.218876	C32	2019
2.197225	0.333333	13.95527	2.302585	1	1	2.70805	C32	2013
1.94591	0.428571	13.32775	2.079442	1	1	2.772589	C32	2014
1.94591	0.428571	13.95527	2.197225	1	1	2.833213	C32	2015
1.94591	0.428571	14.72377	2.079442	1	1	2.995732	B09	2018
1.94591	0.428571	14.90743	1.098612	1	1	3.044522	B09	2019
2.197225	0.444444	15.00213	1.386294	1	4	2.639057	C27	2013
2.197225	0.444444	15.34958	1.609438	1	4	2.70805	C27	2014
2.197225	0.444444	15.47672	1.791759	1	4	2.772589	C27	2015
2.197225	0.444444	14.74436	1.609438	1	4	2.833213	C27	2016
2.197225	0.444444	15.3197	1.386294	1	4	2.890372	C27	2017
2.197225	0.444444	15.31335	1.609438	1	4	2.944439	C27	2018
2.197225	0.444444	16.04268	1.098612	1	4	2.995732	C27	2019
2.197225	0.333333	14.48334	2.772589	1	1	2.397895	C26	2009
2.197225	0.333333	14.48334	2.70805	1	1	2.484907	C26	2010
2.197225	0.333333	14.61536	2.302585	1	1	2.564949	C26	2011
2.197225	0.333333	14.77018	2.079442	1	1	2.639057	C26	2012

2.397895	0.363636	15.35313	2.302585	1	1	2.70805	C26	2013
2.197225	0.333333	15.9011	2.484907	1	1	2.772589	C26	2014
2.197225	0.333333	17.18809	2.197225	1	1	2.833213	C26	2015
2.197225	0.333333	16.54484	2.302585	1	1	2.890372	C26	2016
2.197225	0.333333	16.68521	1.791759	1	1	2.944439	C26	2017
2.197225	0.333333	15.20178	2.079442	1	1	2.995732	C26	2018
2.197225	0.333333	15.45683	1.609438	1	1	3.044522	C26	2019
2.197225	0.333333	14.28332	2.639057	1	1	2.944439	I64	2017
1.94591	0.428571	14.68076	2.70805	1	1	2.995732	I64	2018
2.197225	0.333333	14.43904	2.639057	1	4	2.833213	C39	2016
2.197225	0.333333	14.45283	1.94591	1	4	2.890372	C39	2017
2.197225	0.333333	14.64842	1.609438	1	3	2.772589	C39	2010
2.079442	0.375	14.88022	1.791759	1	3	2.890372	C39	2012
2.079442	0.375	14.6784	1.609438	1	3	2.944439	C39	2013
2.197225	0.333333	14.60397	1.386294	1	3	2.995732	C39	2014
2.197225	0.333333	14.60397	2.079442	1	3	3.044522	C39	2015
2.079442	0.375	14.61751	2.197225	1	3	3.091042	C39	2016
2.197225	0.333333	14.80133	1.791759	1	3	3.135494	C39	2017
2.079442	0.375	14.50365	2.302585	1	3	3.178054	C39	2018
2.079442	0.375	14.58098	1.609438	1	3	3.218876	C39	2019
1.94591	0.428571	13.09293	1.609438	1	1	2.70805	C17	2009
1.94591	0.428571	13.38442	1.386294	1	1	2.772589	C17	2010
1.94591	0.428571	13.49915	1.609438	1	1	2.833213	C17	2011
1.94591	0.428571	13.65252	1.94591	1	1	2.890372	C17	2012
1.94591	0.428571	13.66178	1.94591	1	1	2.944439	C17	2013
1.94591	0.428571	13.69153	1.386294	1	1	2.995732	C17	2014
1.94591	0.428571	13.56718	1.791759	1	1	3.044522	C17	2015
1.94591	0.428571	13.9877	1.94591	1	1	3.091042	C17	2016
1.94591	0.428571	13.99616	1.386294	1	1	3.135494	C17	2017
1.94591	0.428571	14.2827	1.94591	1	1	3.178054	C17	2018
1.94591	0.428571	14.13832	2.079442	1	1	3.218876	C17	2019
2.197225	0.333333	13.80566	1.791759	1	1	2.639057	A01	2009
2.079442	0.375	13.66143	1.791759	1	1	2.70805	A01	2010
2.197225	0.333333	14.03191	2.197225	1	1	2.772589	A01	2011
2.079442	0.375	14.85069	1.386294	1	1	2.833213	A01	2012
2.079442	0.375	14.02439	1.609438	1	1	2.890372	A01	2013
2.197225	0.333333	11.82041	2.079442	1	1	2.944439	A01	2014
2.197225	0.333333	12.7762	2.079442	1	1	2.995732	A01	2015
2.197225	0.333333	13.07632	1.386294	1	1	3.044522	A01	2016
1.609438	0.4	12.13672	1.386294	1	4	3.091042	A01	2017
1.609438	0.4	13.79326	1.386294	1	4	3.135494	A01	2018
1.609438	0.4	13.83972	1.386294	1	4	3.178054	A01	2019
1.94591	0.428571	14.32833	2.484907	1	2	2.833213	C27	2009
1.94591	0.428571	14.36242	2.079442	1	2	2.890372	C27	2010
1.94591	0.428571	14.24091	1.791759	1	2	2.944439	C27	2011
1.94591	0.428571	13.7382	2.197225	1	2	2.995732	C27	2012
1.94591	0.428571	13.7382	2.197225	1	2	3.044522	C27	2013
1.94591	0.428571	13.7382	2.079442	1	3	3.091042	C27	2014
2.197225	0.333333	12.70594	2.484907	1	2	3.044522	F51	2015
2.197225	0.333333	13.59437	2.639057	1	2	3.091042	F51	2016
2.197225	0.333333	13.8474	2.302585	1	2	3.135494	F51	2017
2.197225	0.333333	14.26932	2.772589	1	2	3.178054	F51	2018
2.197225	0.333333	14.72478	2.197225	1	2	3.218876	F51	2019
1.94591	0.428571	12.85265	2.197225	1	1	3.135494	C27	2015
2.197225	0.333333	13.07987	2.302585	1	1	3.178054	C27	2016
1.94591	0.428571	14.64842	2.079442	1	2	2.833213	C18	2014
2.197225	0.333333	14.64842	1.609438	1	2	2.890372	C18	2015
2.197225	0.333333	14.64842	1.609438	1	2	2.944439	C18	2016

2.197225	0.333333	14.7318	2.397895	1	2	2.995732	C18	2017
2.197225	0.333333	14.7318	2.564949	1	2	3.044522	C18	2018
2.079442	0.375	14.7318	1.94591	1	2	3.091042	C18	2019
2.197225	0.333333	12.68788	2.079442	1	1	2.639057	C18	2009
2.197225	0.333333	12.86307	2.302585	1	1	2.70805	C18	2010
2.197225	0.333333	13.02805	2.302585	1	1	2.772589	C18	2011
2.197225	0.333333	13.19932	2.484907	1	1	2.833213	C18	2012
2.079442	0.375	13.17685	2.397895	1	1	2.890372	C18	2013
2.197225	0.333333	13.38626	2.484907	1	1	2.944439	C18	2014
2.197225	0.333333	13.5198	2.484907	1	1	2.995732	C18	2015
2.197225	0.333333	13.72875	2.484907	1	1	3.044522	C18	2016
2.197225	0.333333	14.39151	2.639057	1	1	3.091042	C18	2017
2.197225	0.333333	14.66459	2.772589	1	1	3.135494	C18	2018
2.197225	0.333333	14.65803	2.890372	1	1	3.178054	C18	2019
2.197225	0.444444	12.65715	2.302585	1	2	2.772589	C25	2009
2.197225	0.444444	13.28108	1.791759	1	2	2.833213	C25	2010
2.197225	0.444444	13.28448	2.484907	1	2	2.890372	C25	2011
2.197225	0.444444	13.07778	1.94591	1	2	2.944439	C31	2012
2.197225	0.444444	13.08217	1.386294	1	2	2.995732	C31	2013
2.079442	0.375	12.6072	1.94591	1	2	3.044522	C31	2014
1.94591	0.428571	12.59031	2.197225	1	2	3.135494	C31	2016
1.94591	0.428571	13.06643	1.94591	1	2	3.178054	C31	2017
1.94591	0.428571	12.79386	1.609438	1	3	2.833213	C27	2014
1.94591	0.428571	12.65076	1.386294	1	3	2.890372	C27	2015
2.197225	0.444444	13.32449	2.079442	1	4	2.639057	C27	2009
2.197225	0.444444	14.04057	2.079442	1	4	2.70805	C27	2010
2.197225	0.444444	14.03865	2.833213	1	4	2.772589	C27	2011
2.197225	0.444444	14.25377	2.70805	1	4	2.833213	C27	2012
2.197225	0.444444	14.25377	2.484907	1	4	2.890372	C27	2013
2.197225	0.444444	14.25377	2.890372	1	4	2.944439	C27	2014
2.197225	0.444444	14.25377	2.995732	1	4	2.995732	C27	2015
2.197225	0.444444	14.98349	2.944439	1	4	3.044522	C27	2016
2.197225	0.444444	14.42837	3.091042	1	4	3.091042	C27	2017
2.197225	0.444444	14.71307	2.833213	1	4	3.135494	C27	2018
2.197225	0.444444	14.57524	2.639057	1	4	3.178054	C27	2019
1.94591	0.428571	14.83016	2.397895	1	4	2.70805	E50	2014
1.94591	0.428571	15.03125	3.178054	1	4	2.772589	E50	2015
1.94591	0.428571	14.94219	3.496508	1	4	2.833213	E50	2016
1.94591	0.428571	14.89215	3.332205	1	4	2.944439	E50	2018
1.94591	0.428571	14.74882	2.564949	1	4	2.995732	E50	2019
2.197225	0.333333	13.16158	2.70805	1	4	2.197225	C39	2009
2.197225	0.333333	13.61706	2.302585	1	4	2.302585	C39	2010
2.079442	0.375	14.49461	2.197225	1	4	2.397895	C39	2011
2.197225	0.333333	14.67629	2.302585	1	4	2.484907	C38	2012
2.079442	0.375	14.64231	2.639057	1	4	2.564949	C38	2013
2.197225	0.333333	14.51663	2.772589	1	4	2.639057	C38	2014
2.197225	0.333333	14.74405	2.772589	1	4	2.70805	C38	2015
2.197225	0.333333	14.78135	2.70805	1	4	2.772589	C38	2016
2.197225	0.333333	14.91559	2.833213	1	4	2.833213	C38	2017
2.197225	0.333333	14.53622	2.772589	1	4	2.890372	C38	2018
2.197225	0.333333	14.72397	2.302585	1	4	2.944439	C38	2019
2.302585	0.4	12.89672	2.484907	1	1	2.639057	C38	2009
2.397895	0.363636	12.91164	2.484907	1	1	2.70805	C38	2010
2.397895	0.363636	12.91164	2.302585	1	1	2.772589	C38	2011
2.397895	0.363636	12.91164	2.197225	1	1	2.833213	C26	2012
2.197225	0.333333	13.33747	2.079442	1	1	2.890372	C26	2013
2.197225	0.333333	13.33747	1.94591	1	1	2.944439	C26	2014
2.197225	0.333333	13.73213	2.197225	1	1	2.995732	C26	2015

2.197225	0.333333	13.73213	2.079442	1	1	3.044522	C38	2016
2.302585	0.4	13.73213	1.791759	1	1	3.091042	C38	2017
2.197225	0.333333	13.76422	2.197225	1	1	3.135494	C38	2018
2.197225	0.333333	13.57216	2.197225	1	1	3.178054	C38	2019
1.94591	0.428571	13.24281	2.197225	1	2	2.639057	E47	2009
1.94591	0.428571	13.69221	1.609438	1	2	2.70805	E47	2010
1.94591	0.428571	13.69221	1.94591	1	2	2.772589	E47	2011
1.94591	0.428571	13.69221	1.94591	1	2	2.833213	E48	2012
1.94591	0.428571	13.94952	1.94591	1	2	2.890372	E48	2013
1.94591	0.428571	14.07787	1.791759	1	2	2.944439	E48	2014
1.94591	0.428571	14.28551	2.079442	1	2	2.995732	E48	2015
1.94591	0.428571	14.49354	2.079442	1	2	3.044522	E48	2016
1.94591	0.428571	14.49354	2.302585	1	2	3.091042	E48	2017
1.94591	0.428571	14.49354	1.94591	1	2	3.135494	E48	2018
1.94591	0.428571	14.85136	2.197225	1	2	3.178054	E48	2019
2.302585	0.4	12.89922	1.94591	1	4	2.890372	C17	2009
2.397895	0.363636	13.56705	1.791759	1	4	2.944439	C17	2010
2.302585	0.4	13.67625	2.302585	1	4	2.995732	C17	2011
2.302585	0.4	13.9834	2.079442	1	4	3.044522	C17	2012
2.397895	0.363636	13.98618	1.94591	1	4	3.091042	C17	2013
2.302585	0.4	13.762	2.079442	1	4	3.135494	C17	2014
2.302585	0.4	13.90763	1.791759	1	4	3.178054	C17	2015
2.397895	0.363636	14.15005	1.94591	1	4	3.218876	C17	2016
2.397895	0.363636	14.12871	1.386294	1	4	3.258097	C17	2017
2.397895	0.363636	14.33419	1.609438	1	4	3.295837	C17	2018
2.397895	0.363636	14.34837	1.609438	1	4	3.332205	C17	2019
2.197225	0.333333	14.6445	3.044522	1	2	2.944439	E50	2018
2.197225	0.333333	14.75942	2.484907	1	1	2.639057	E48	2009
2.197225	0.333333	14.75942	2.564949	1	1	2.70805	E48	2010
2.197225	0.333333	14.73786	2.302585	1	1	2.772589	E48	2011
2.197225	0.333333	14.72151	2.397895	1	1	2.833213	E48	2012
2.197225	0.333333	14.66681	2.079442	1	1	2.890372	E48	2013
2.197225	0.333333	14.60637	1.94591	1	1	2.944439	E48	2014
2.079442	0.375	14.26378	2.302585	1	4	2.995732	E48	2015
1.94591	0.428571	14.47531	2.197225	1	4	3.044522	E48	2016
1.94591	0.428571	14.60755	2.397895	1	4	3.091042	E48	2017
2.197225	0.333333	13.55648	1.791759	0	4	2.833213	C38	2017
2.197225	0.333333	13.60726	1.94591	0	4	2.890372	C38	2018
2.197225	0.333333	13.6012	1.791759	0	4	2.944439	C38	2019
2.197225	0.333333	14.24594	2.302585	1	3	2.302585	C39	2009
2.079442	0.375	14.36686	2.397895	1	3	2.397895	C39	2010
2.197225	0.333333	14.70752	2.302585	1	3	2.484907	C39	2011
2.197225	0.333333	14.82998	2.397895	1	3	2.564949	C38	2012
2.197225	0.333333	14.83403	2.564949	1	3	2.639057	C38	2013
2.197225	0.333333	14.91509	1.94591	1	3	2.70805	C38	2014
2.197225	0.333333	15.0663	2.079442	1	3	2.772589	C38	2015
2.197225	0.333333	15.17882	2.397895	1	3	2.833213	C38	2016
2.197225	0.333333	15.36571	2.079442	1	3	2.890372	C38	2017
2.197225	0.333333	15.39451	1.791759	1	3	2.944439	C38	2018
2.197225	0.333333	15.43248	2.197225	1	3	2.995732	C38	2019
2.197225	0.333333	13.16158	1.609438	1	2	2.564949	C28	2009
2.079442	0.375	13.21949	1.609438	1	2	2.639057	C28	2010
2.197225	0.333333	13.31513	2.079442	1	2	2.70805	C28	2011
2.197225	0.333333	13.42054	1.94591	1	2	2.772589	C28	2012
2.197225	0.333333	13.45511	1.386294	1	2	2.833213	C28	2013
2.197225	0.333333	13.48701	1.098612	1	2	2.890372	C28	2014
2.197225	0.333333	13.55713	1.386294	1	2	2.944439	C28	2015
2.197225	0.333333	13.55415	1.791759	1	2	2.995732	C28	2016

2.197225	0.333333	13.90169	1.94591	1	2	3.044522	C28	2017
2.197225	0.333333	14.13178	1.386294	1	2	3.091042	C28	2018
1.94591	0.428571	14.24586	1.609438	1	2	3.135494	C28	2019
2.079442	0.375	14.51696	1.609438	1	5	2.397895	C27	2009
2.079442	0.375	14.79135	1.609438	1	5	2.484907	C27	2010
2.079442	0.375	15.11695	2.302585	1	5	2.564949	C27	2011
2.079442	0.375	15.60462	1.94591	1	5	2.639057	C27	2012
2.079442	0.375	15.59095	1.94591	1	5	2.70805	C27	2013
2.079442	0.375	15.67029	2.197225	1	5	2.772589	C27	2014
2.197225	0.333333	16.76207	1.609438	1	5	2.833213	C27	2015
2.197225	0.333333	15.77974	2.302585	0	5	2.890372	C27	2016
2.197225	0.333333	16.02575	2.079442	0	5	2.944439	C27	2017
2.197225	0.333333	16.17631	1.94591	0	5	2.995732	C27	2018
2.197225	0.333333	15.51543	1.791759	0	5	3.044522	C27	2019
2.197225	0.333333	14.9626	2.302585	1	1	2.397895	C13	2011
2.197225	0.333333	14.65063	1.791759	1	1	2.564949	C38	2013
2.197225	0.333333	14.71376	2.397895	1	1	2.639057	C38	2014
2.197225	0.333333	14.6968	2.302585	1	1	2.70805	C38	2015
2.197225	0.333333	14.8642	2.302585	1	1	2.772589	C38	2016
1.94591	0.428571	14.76054	2.079442	1	1	2.833213	C38	2017
2.197225	0.333333	14.9274	2.197225	1	1	2.890372	C38	2018
2.197225	0.333333	13.7293	2.397895	1	1	2.397895	L72	2010
2.197225	0.333333	13.68768	1.94591	1	4	2.197225	I64	2009
2.197225	0.333333	13.54107	1.791759	1	4	2.302585	I64	2010
2.197225	0.333333	13.54107	2.397895	1	4	2.397895	I64	2011
2.639057	0.357143	13.54107	2.079442	1	4	2.484907	I65	2012
2.397895	0.363636	13.54107	1.791759	1	4	2.564949	I65	2013
2.397895	0.363636	13.74294	1.791759	1	4	2.639057	I65	2014
2.397895	0.363636	13.74294	1.94591	1	4	2.70805	I65	2015
2.397895	0.363636	13.81551	2.484907	1	4	2.772589	I65	2016
2.302585	0.4	13.81551	1.94591	1	4	2.890372	I65	2018
2.197225	0.333333	14.24729	1.791759	1	4	2.944439	I65	2019
2.197225	0.333333	13.89321	2.484907	1	5	2.772589	C27	2009
2.197225	0.333333	14.21395	2.397895	1	5	2.833213	C27	2010
2.197225	0.333333	14.39235	2.397895	1	5	2.890372	C27	2011
2.197225	0.333333	14.43404	2.70805	1	5	2.944439	C27	2012
2.197225	0.333333	14.68307	2.397895	1	5	2.995732	C27	2013
2.079442	0.375	14.67214	2.397895	1	5	3.044522	C27	2014
2.197225	0.333333	14.52674	2.833213	1	5	3.091042	C27	2015
2.197225	0.333333	14.45066	2.484907	1	5	3.135494	C27	2016
2.197225	0.333333	14.57374	2.079442	1	5	3.178054	C27	2017
2.197225	0.333333	14.83954	2.772589	1	5	3.218876	C27	2018
2.197225	0.333333	14.85681	2.772589	1	5	3.258097	C27	2019
2.197225	0.333333	13.8643	2.302585	1	4	2.397895	C38	2009
2.197225	0.333333	13.8643	2.197225	1	4	2.484907	C38	2010
2.197225	0.333333	14.02252	2.564949	1	4	2.564949	C38	2011
2.197225	0.333333	14.02252	2.397895	1	4	2.639057	C38	2012
2.197225	0.333333	14.07015	2.079442	1	4	2.70805	C38	2013
2.197225	0.333333	14.11562	2.302585	1	4	2.772589	C38	2014
2.197225	0.333333	14.18015	2.302585	1	4	2.833213	C38	2015
2.197225	0.333333	14.96908	2.302585	1	4	2.890372	C38	2016
2.197225	0.333333	15.43073	2.484907	1	4	2.944439	C38	2017
2.197225	0.333333	15.3074	2.484907	1	4	2.995732	C38	2018
2.197225	0.333333	15.3482	2.197225	1	4	3.044522	C38	2019
1.94591	0.428571	12.46844	2.197225	0	3	2.484907	C29	2009
1.94591	0.428571	12.76569	1.609438	0	3	2.564949	C29	2010
1.94591	0.428571	12.92391	1.791759	0	3	2.639057	C29	2011
1.94591	0.428571	13.30468	2.302585	0	3	2.70805	C26	2012

1.94591	0.428571	13.35348	1.609438	0	3	2.772589	C26	2013
1.791759	0.5	13.32326	1.791759	0	3	2.833213	C26	2014
1.94591	0.428571	13.35348	2.079442	0	3	2.890372	C26	2015
2.197225	0.333333	13.35348	1.94591	0	3	2.944439	C26	2016
2.197225	0.333333	13.35348	2.079442	1	3	2.995732	C26	2017
2.197225	0.333333	13.35348	1.94591	1	3	3.044522	C26	2018
2.564949	0.384615	13.41801	1.609438	1	4	2.197225	C27	2009
2.197225	0.333333	13.41801	1.791759	1	4	2.302585	C27	2010
2.197225	0.333333	13.40362	1.94591	1	4	2.397895	C27	2011
2.197225	0.333333	13.40362	2.079442	1	4	2.484907	C27	2012
2.197225	0.333333	13.40362	2.302585	1	4	2.564949	C27	2013
2.079442	0.375	13.4531	2.079442	1	4	2.639057	C27	2014
2.079442	0.375	13.40362	2.197225	1	4	2.70805	C27	2015
2.197225	0.333333	13.44792	1.94591	1	4	2.772589	C27	2016
2.197225	0.333333	13.44879	1.609438	1	4	2.833213	C27	2017
2.197225	0.333333	15.4053	1.94591	1	4	2.944439	C27	2019
2.197225	0.333333	14.64406	2.70805	1	1	2.772589	C26	2009
2.197225	0.333333	14.57958	2.564949	1	1	2.833213	C26	2010
2.197225	0.333333	14.40773	2.564949	1	1	2.890372	C26	2011
2.197225	0.333333	14.6076	2.197225	1	1	2.944439	C26	2012
2.079442	0.375	14.92572	2.079442	1	1	2.995732	C26	2013
2.197225	0.333333	14.33073	2.484907	1	1	3.091042	C26	2015
2.197225	0.333333	14.86703	2.564949	1	1	3.178054	C26	2017
2.197225	0.333333	14.92605	2.639057	1	1	3.218876	C26	2018
2.197225	0.333333	14.91078	2.197225	1	1	3.258097	C26	2019
2.197225	0.333333	13.19932	1.609438	1	2	2.890372	I65	2010
2.197225	0.333333	12.02754	1.791759	1	2	2.944439	I65	2011
2.197225	0.333333	13.17496	1.609438	1	2	2.995732	C27	2012
2.197225	0.333333	13.14491	2.079442	1	2	3.091042	C27	2014
2.197225	0.333333	13.41533	1.94591	1	2	3.135494	C27	2015
2.197225	0.333333	13.46695	2.302585	1	2	3.178054	C27	2016
2.197225	0.333333	13.42941	1.791759	1	2	3.218876	C27	2017
2.197225	0.333333	13.87895	1.386294	1	2	3.258097	C27	2018
2.197225	0.333333	13.91146	2.197225	1	2	3.295837	C27	2019
1.791759	0.333333	13.05408	2.564949	1	3	2.833213	C29	2009
1.791759	0.333333	13.05408	2.302585	1	3	2.890372	C29	2010
1.791759	0.333333	13.23569	2.079442	1	3	2.944439	C29	2011
1.94591	0.428571	13.28788	2.484907	1	3	2.995732	C27	2012
1.94591	0.428571	13.86601	1.94591	1	3	3.044522	C27	2013
2.079442	0.375	13.98373	2.302585	1	3	3.091042	C27	2014
2.079442	0.375	13.56935	2.397895	1	3	3.135494	C32	2015
2.079442	0.375	13.73755	2.564949	1	3	2.833213	S90	2009
2.397895	0.363636	14.41906	1.791759	1	3	2.995732	S90	2012
2.397895	0.363636	14.64393	1.609438	1	3	3.044522	S90	2013
2.397895	0.363636	14.62626	2.484907	1	3	3.091042	S90	2014
2.397895	0.363636	14.58037	2.484907	1	3	3.135494	S90	2015
2.397895	0.363636	15.40597	2.197225	1	3	3.178054	S90	2016
2.397895	0.363636	15.0677	2.079442	1	3	3.218876	S90	2017
2.397895	0.363636	14.97553	2.079442	1	3	3.258097	S90	2018
2.302585	0.4	15.00943	2.079442	1	3	3.295837	S90	2019
2.197225	0.333333	14.45663	2.079442	1	1	2.890372	K70	2009
2.197225	0.333333	14.48185	2.079442	1	1	2.944439	K70	2010
2.197225	0.333333	14.52591	2.197225	1	1	2.995732	K70	2011
2.197225	0.333333	14.57182	1.791759	1	1	3.044522	K70	2012
2.197225	0.333333	14.53291	2.079442	1	1	3.091042	K70	2013
2.197225	0.333333	14.59987	1.791759	1	1	3.135494	K70	2014
2.197225	0.333333	14.53115	1.94591	1	1	2.833213	C30	2009
2.197225	0.333333	15.15757	1.94591	1	1	2.890372	C30	2010

2.197225	0.333333	15.48845	1.94591	1	1	2.944439	C30	2011
2.197225	0.333333	15.49796	2.197225	1	1	2.995732	C30	2012
2.197225	0.333333	15.65947	2.079442	1	1	3.044522	C30	2013
2.197225	0.333333	15.7511	1.94591	1	1	3.091042	C30	2014
2.197225	0.333333	16.15557	1.609438	1	1	3.135494	C30	2015
2.079442	0.375	16.01332	1.609438	1	1	3.178054	C30	2016
2.197225	0.333333	16.00498	1.609438	1	1	3.218876	C30	2017
2.197225	0.333333	16.00616	2.079442	1	1	3.258097	C30	2018
2.197225	0.333333	16.17619	1.386294	1	1	3.295837	C30	2019
2.197225	0.333333	13.02915	2.944439	1	1	3.091042	C27	2015
2.197225	0.333333	13.75364	2.397895	1	1	3.135494	C27	2016
2.197225	0.333333	13.79531	2.833213	1	1	3.178054	C27	2017
2.079442	0.375	13.58194	2.079442	1	1	3.218876	C27	2018
2.197225	0.333333	13.66701	1.94591	1	1	3.258097	C27	2019
2.197225	0.444444	14.7173	1.94591	0	1	3.135494	F52	2013
2.197225	0.333333	14.69085	2.197225	0	1	3.178054	F52	2014
2.197225	0.333333	14.91951	1.386294	0	1	3.218876	F52	2015
2.197225	0.333333	14.91005	2.484907	0	1	3.258097	F52	2016
2.197225	0.333333	15.316	2.302585	0	1	3.295837	F52	2017
2.197225	0.333333	15.50372	2.484907	0	1	3.332205	F52	2018
2.197225	0.333333	15.72671	2.639057	0	1	3.367296	F52	2019
1.94591	0.428571	13.72799	2.564949	1	2	2.772589	C39	2009
1.94591	0.428571	13.76957	2.70805	1	2	2.833213	C39	2010
1.94591	0.428571	14.15255	2.70805	1	2	2.890372	C39	2011
1.94591	0.428571	14.15205	2.484907	1	2	2.944439	C39	2012
1.94591	0.428571	14.18707	2.833213	1	2	2.995732	C39	2013
1.94591	0.428571	14.60051	2.833213	1	2	3.044522	C39	2014
1.94591	0.428571	14.50796	2.890372	1	2	3.091042	C39	2015
2.079442	0.375	14.53608	2.397895	1	2	3.135494	C39	2016
2.079442	0.375	15.14605	2.397895	1	3	3.178054	C39	2017
2.079442	0.375	15.03929	2.302585	1	3	3.218876	C39	2018
2.079442	0.375	15.03929	2.397895	1	2	3.258097	C39	2019
1.609438	0.4	13.07107	1.791759	1	3	2.564949	K70	2009
1.791759	0.333333	12.91902	2.484907	1	3	2.639057	K70	2010
1.791759	0.333333	12.97618	2.079442	1	3	2.70805	K70	2011
1.791759	0.333333	13.25864	1.791759	1	3	2.772589	K70	2012
1.791759	0.333333	13.85953	2.302585	1	3	2.890372	K70	2014
1.94591	0.428571	13.60058	2.302585	1	3	3.178054	C19	2017
1.94591	0.428571	13.56987	1.791759	1	3	3.218876	C19	2018
1.94591	0.428571	13.53844	2.302585	1	3	3.258097	C19	2019
2.197225	0.333333	13.38427	1.609438	1	4	2.833213	F52	2009
2.197225	0.333333	13.87792	2.197225	1	4	2.890372	F52	2010
2.197225	0.333333	13.92436	1.609438	1	4	2.944439	F52	2011
2.197225	0.333333	14.15981	2.197225	1	4	2.995732	F52	2012
2.079442	0.375	14.20485	2.197225	1	4	3.044522	F52	2013
2.197225	0.333333	14.24091	1.791759	1	4	3.091042	F52	2014
2.197225	0.333333	14.17179	1.94591	1	4	3.135494	F52	2015
2.197225	0.333333	14.17179	2.197225	1	4	3.178054	F52	2016
2.197225	0.333333	14.28882	2.302585	1	4	3.218876	F52	2017
2.197225	0.333333	14.55745	2.639057	1	4	3.295837	F52	2019
2.197225	0.333333	12.97618	1.791759	1	3	2.564949	L72	2009
2.197225	0.333333	13.00358	1.94591	1	3	2.639057	L72	2010
2.197225	0.333333	13.15289	2.302585	1	3	2.70805	L72	2011
2.197225	0.333333	13.13033	2.079442	1	3	2.772589	N78	2012
2.397895	0.363636	12.97154	2.397895	1	3	2.833213	N78	2013
2.397895	0.363636	13.35348	2.197225	1	3	2.890372	N78	2014
2.397895	0.363636	13.24245	2.397895	1	3	2.944439	N78	2015
1.94591	0.428571	14.43824	2.197225	1	4	2.639057	Q83	2009

1.609438	0.6	14.79496	2.197225	1	4	2.890372	Q83	2013
1.94591	0.428571	14.44838	2.197225	1	4	2.944439	Q83	2014
1.94591	0.428571	14.42397	2.197225	1	4	2.995732	Q83	2015
1.94591	0.428571	15.19947	2.197225	1	4	3.044522	Q83	2016
1.94591	0.428571	15.46232	2.197225	1	4	3.091042	Q83	2017
1.94591	0.428571	13.92257	2.302585	1	5	2.890372	C26	2010
1.791759	0.333333	13.9995	1.791759	1	5	2.944439	C26	2011
1.791759	0.333333	14.09109	2.397895	1	5	2.995732	C26	2012
2.197225	0.333333	14.55816	2.197225	1	5	3.044522	C26	2013
2.197225	0.333333	15.17387	1.94591	1	5	3.091042	C26	2014
2.197225	0.333333	15.20741	2.302585	1	5	3.135494	C26	2015
2.197225	0.333333	15.11183	2.397895	1	5	3.178054	C26	2016
2.079442	0.375	15.28108	2.564949	1	5	3.218876	C26	2017
2.197225	0.333333	15.24486	2.890372	1	5	3.258097	C26	2018
2.197225	0.333333	15.38083	2.890372	1	5	3.295837	C26	2019
1.609438	0.4	12.95678	1.94591	1	4	2.772589	C37	2009
1.609438	0.4	12.88055	2.197225	1	4	2.833213	C37	2010
1.609438	0.4	13.46921	2.197225	1	4	2.890372	C37	2011
1.609438	0.4	13.45884	2.302585	1	4	2.944439	C37	2012
1.609438	0.4	13.45855	2.197225	1	4	2.995732	C37	2013
1.609438	0.4	13.45597	2.564949	1	4	3.044522	C37	2014
1.609438	0.4	13.23265	2.397895	1	4	3.091042	C37	2015
1.791759	0.333333	13.2444	2.197225	1	4	3.135494	C37	2016
1.791759	0.333333	13.5579	2.302585	1	4	3.178054	C37	2017
1.791759	0.333333	13.61401	2.397895	1	4	3.218876	C37	2018
1.791759	0.333333	13.50449	2.484907	1	4	3.258097	C37	2019
2.197225	0.444444	14.53184	2.890372	1	4	2.833213	K70	2009
2.197225	0.444444	15.29135	2.197225	1	4	2.890372	K70	2010
2.197225	0.444444	15.57182	2.302585	1	4	2.944439	K70	2011
2.197225	0.444444	15.45645	2.197225	1	4	2.995732	K70	2012
2.197225	0.444444	15.58525	2.397895	1	4	3.044522	K70	2013
2.197225	0.444444	15.59046	2.197225	1	4	3.091042	K70	2014
2.197225	0.444444	15.46663	2.564949	1	4	3.135494	K70	2015
2.197225	0.444444	15.51223	2.079442	1	4	3.178054	K70	2016
2.197225	0.444444	16.02489	2.302585	1	4	3.218876	K70	2017
2.197225	0.444444	15.94888	2.079442	1	4	3.258097	K70	2018
2.197225	0.444444	15.86444	2.397895	1	4	3.295837	K70	2019
2.197225	0.333333	14.01436	2.890372	1	4	2.772589	F52	2009
2.079442	0.375	14.55368	2.197225	1	4	2.833213	F52	2010
2.197225	0.333333	14.48031	2.564949	1	4	2.890372	F52	2011
2.197225	0.333333	14.48544	2.890372	1	4	2.944439	F52	2012
2.197225	0.333333	14.6544	2.639057	1	4	2.995732	F52	2013
2.079442	0.375	14.6318	2.70805	1	4	3.044522	F52	2014
2.079442	0.375	14.70759	3.135494	1	4	3.091042	F52	2015
2.197225	0.333333	14.54277	3.295837	1	4	3.135494	F52	2016
2.197225	0.333333	14.18217	2.944439	1	4	3.178054	F52	2017
2.079442	0.375	14.25163	3.044522	1	4	3.218876	F52	2018
2.197225	0.333333	14.67836	3.218876	1	4	3.258097	F52	2019
2.197225	0.333333	14.12762	1.94591	1	2	2.70805	C23	2009
2.197225	0.333333	14.35001	1.94591	1	2	2.772589	C23	2010
2.197225	0.333333	14.37312	1.94591	1	2	2.833213	C23	2011
2.197225	0.333333	14.39016	1.94591	1	2	2.890372	C23	2012
2.197225	0.333333	14.40207	1.609438	1	2	2.944439	C23	2013
2.197225	0.333333	14.3694	1.791759	1	2	2.995732	C23	2014
2.197225	0.333333	14.43555	1.94591	1	2	3.044522	C23	2015
2.197225	0.333333	14.43124	1.791759	1	2	3.091042	C23	2016
2.197225	0.333333	14.56409	1.791759	1	2	3.135494	C23	2017
2.302585	0.4	14.85225	1.791759	1	2	3.178054	C23	2018

2.079442	0.375	14.60669	2.079442	1	2	3.218876	C23	2019
2.197225	0.333333	15.31665	1.94591	0	1	3.135494	F52	2015
2.197225	0.333333	15.46715	1.791759	0	1	3.178054	F52	2016
2.302585	0.4	15.66709	2.079442	0	1	3.258097	F52	2018
2.197225	0.333333	15.39226	1.386294	0	1	3.295837	F52	2019
2.197225	0.333333	14.58098	1.94591	1	4	3.258097	F52	2018
2.197225	0.333333	14.59024	1.791759	1	4	3.295837	F52	2019
2.197225	0.333333	13.05494	1.386294	1	3	2.833213	C27	2009
2.197225	0.333333	13.31661	1.791759	1	3	2.890372	C27	2010
2.197225	0.333333	13.39726	2.079442	1	3	2.944439	C27	2011
2.197225	0.333333	13.48004	1.94591	1	3	2.995732	C27	2012
2.197225	0.333333	13.5019	1.94591	1	3	3.044522	C27	2013
2.197225	0.333333	13.56935	2.079442	1	3	3.091042	C27	2014
2.197225	0.333333	13.57395	2.397895	1	3	3.135494	C27	2015
2.197225	0.333333	13.57852	2.484907	1	3	3.178054	C27	2016
2.197225	0.333333	15.07386	1.94591	1	3	3.218876	C27	2017
2.079442	0.375	14.80802	2.197225	1	3	3.258097	C27	2018
2.197225	0.333333	15.39352	2.197225	1	3	3.295837	C27	2019
2.197225	0.333333	12.93555	2.079442	1	1	2.639057	C27	2009
2.079442	0.375	13.13508	2.302585	1	1	2.70805	C27	2010
2.197225	0.333333	13.82259	2.564949	1	1	2.772589	C27	2011
2.197225	0.333333	15.17618	2.197225	1	1	2.833213	C38	2012
2.197225	0.333333	15.24398	2.197225	1	1	2.890372	C38	2013
2.197225	0.333333	14.93777	2.639057	1	1	2.944439	C38	2014
2.197225	0.333333	15.28856	2.944439	1	1	2.995732	C38	2015
2.197225	0.333333	15.28828	2.772589	1	1	3.044522	C38	2016
2.197225	0.333333	14.87943	2.564949	1	1	3.091042	C38	2017
2.197225	0.333333	14.58668	2.197225	1	1	3.135494	C38	2018
2.197225	0.333333	14.85459	2.197225	1	1	3.178054	C38	2019
2.197225	0.333333	14.75078	2.079442	1	1	3.135494	C38	2013
2.302585	0.4	14.83128	1.791759	1	1	3.178054	C38	2014
2.197225	0.333333	12.35449	1.386294	1	1	2.397895	A03	2009
2.197225	0.333333	12.8739	1.386294	1	1	2.484907	A03	2010
2.079442	0.375	12.72487	1.791759	1	1	2.564949	A03	2011
1.94591	0.428571	12.72487	1.609438	1	1	2.639057	A03	2012
1.94591	0.428571	13.89247	1.94591	1	1	2.70805	A03	2013
1.791759	0.5	13.89247	1.609438	1	1	2.772589	A03	2014
1.94591	0.428571	13.89247	1.791759	1	1	2.833213	A03	2015
1.94591	0.428571	13.89247	2.302585	1	1	2.890372	A03	2016
1.94591	0.428571	13.89247	1.94591	1	1	2.944439	A03	2017
1.791759	0.5	14.18015	1.609438	1	1	2.995732	A03	2018
1.94591	0.428571	14.18015	1.94591	1	1	3.044522	A03	2019
2.397895	0.363636	13.21767	2.197225	1	4	2.772589	C27	2009
2.397895	0.363636	14.33977	2.302585	1	4	2.833213	C27	2010
2.397895	0.363636	14.07787	2.397895	1	4	2.890372	C27	2011
2.397895	0.363636	14.18707	2.302585	1	4	2.944439	C27	2012
2.197225	0.333333	14.18707	2.484907	1	4	2.995732	F52	2013
2.197225	0.333333	14.17179	2.564949	1	4	3.044522	F52	2014
2.197225	0.333333	14.13251	2.484907	1	4	3.091042	F52	2015
2.197225	0.333333	14.36629	1.94591	1	4	3.135494	F52	2016
2.197225	0.333333	14.26595	2.079442	1	4	3.178054	F52	2017
2.197225	0.333333	14.12432	2.197225	1	4	3.218876	F52	2018
2.197225	0.333333	14.44193	2.302585	1	4	3.258097	F52	2019
1.94591	0.428571	12.52743	1.791759	1	3	2.772589	E47	2009
1.94591	0.428571	12.2634	2.397895	1	3	2.833213	E47	2010
1.94591	0.428571	12.6152	2.302585	1	3	2.890372	E47	2011
1.791759	0.5	13.03222	2.079442	1	3	2.944439	E48	2012
1.94591	0.428571	12.88131	2.197225	1	3	2.995732	E48	2013

1.94591	0.428571	12.90545	2.484907	1	3	3.044522	E48	2014
1.791759	0.333333	13.43922	2.484907	1	3	3.091042	E48	2015
1.791759	0.333333	13.226	2.995732	1	3	3.135494	I64	2016
2.197225	0.333333	14.56191	3.218876	1	3	3.178054	I64	2017
2.197225	0.333333	15.14906	3.135494	1	3	3.218876	I64	2018
2.397895	0.363636	14.05106	1.386294	1	2	2.397895	F51	2010
2.397895	0.363636	13.67981	2.397895	1	2	2.484907	F51	2011
2.397895	0.363636	13.95805	1.94591	1	2	2.564949	F51	2012
2.397895	0.363636	14.22071	2.302585	1	2	2.639057	F51	2013
2.397895	0.363636	14.21227	2.484907	1	2	2.70805	F51	2014
2.397895	0.363636	14.31495	1.94591	1	2	2.772589	F51	2015
2.397895	0.363636	14.5351	1.94591	1	2	2.833213	F51	2016
2.397895	0.363636	14.65686	2.564949	1	2	2.890372	F51	2017
2.397895	0.363636	14.97697	1.94591	1	2	2.944439	F51	2018
2.397895	0.363636	15.04161	1.94591	1	2	2.995732	F51	2019
2.197225	0.333333	15.0469	1.94591	0	2	2.639057	C34	2009
2.197225	0.333333	15.1915	1.94591	0	2	2.70805	C34	2010
2.197225	0.333333	15.27645	1.94591	0	2	2.772589	C34	2011
2.197225	0.333333	15.25535	1.94591	0	2	2.833213	C34	2012
2.197225	0.333333	15.26478	1.94591	0	2	2.890372	C34	2013
2.197225	0.333333	15.37747	1.94591	1	2	2.944439	C34	2014
2.197225	0.333333	15.36055	1.94591	1	2	2.995732	C34	2015
2.197225	0.333333	15.36055	1.94591	1	2	3.044522	C34	2016
2.079442	0.375	15.37155	1.94591	1	2	3.091042	C34	2017
2.079442	0.375	15.40066	1.94591	1	2	3.135494	C34	2018
2.197225	0.333333	15.38413	1.94591	1	2	3.178054	C34	2019
2.197225	0.333333	14.64153	2.70805	1	3	2.079442	C25	2011
2.079442	0.375	14.32588	2.302585	1	3	2.197225	C25	2012
2.197225	0.333333	14.48206	2.302585	1	3	2.302585	C25	2013
2.197225	0.333333	14.62431	2.397895	1	3	2.397895	C25	2014
2.197225	0.333333	14.58762	2.772589	1	3	2.484907	C25	2015
2.197225	0.333333	14.63203	2.639057	1	3	2.564949	C25	2016
2.197225	0.333333	14.49997	2.833213	1	3	2.639057	C25	2017
2.197225	0.333333	14.59502	2.564949	1	3	2.70805	C25	2018
2.197225	0.333333	14.67629	2.833213	1	3	2.772589	C25	2019
2.197225	0.333333	12.82126	2.484907	1	1	2.397895	C25	2014
2.197225	0.333333	12.76569	2.484907	1	1	2.484907	C25	2015
2.197225	0.333333	12.82126	2.397895	1	1	2.564949	C25	2016
2.197225	0.333333	12.82126	2.302585	1	1	2.639057	C25	2017
2.197225	0.333333	12.67608	2.772589	1	1	2.70805	C25	2018
2.197225	0.333333	13.12236	2.484907	1	1	2.772589	C25	2019
2.079442	0.375	14.9007	2.397895	1	1	2.639057	B09	2016
1.94591	0.428571	14.86926	2.079442	1	1	2.70805	B09	2017
1.94591	0.428571	15.37285	2.639057	1	1	2.772589	B09	2018
2.079442	0.375	14.94057	2.079442	1	1	2.833213	B09	2019
2.079442	0.375	15.37846	1.609438	1	1	2.302585	G56	2014
2.079442	0.375	15.78248	2.302585	1	1	2.397895	G56	2015
1.94591	0.428571	16.01335	2.302585	1	1	2.484907	G56	2016
2.079442	0.375	15.90495	2.079442	1	1	2.564949	G56	2017
2.079442	0.375	15.89261	1.791759	1	1	2.639057	G56	2018
2.079442	0.375	15.90421	1.791759	1	1	2.70805	G56	2019
2.197225	0.444444	14.1591	1.791759	1	3	2.484907	C31	2011
2.197225	0.444444	14.07787	2.397895	1	3	2.564949	C33	2012
2.197225	0.444444	14.03062	2.302585	1	3	2.639057	C33	2013
1.94591	0.428571	13.98946	1.94591	1	3	2.70805	C33	2014
1.94591	0.428571	13.91716	1.94591	1	3	2.772589	C33	2015
1.94591	0.428571	14.39145	2.079442	1	1	3.091042	F52	2018
1.94591	0.428571	14.58959	2.302585	1	1	3.135494	F52	2019

1.791759	0.5	13.9439	1.609438	1	2	1.791759	C35	2011
1.791759	0.5	14.28282	2.079442	1	2	1.94591	C35	2012
1.94591	0.428571	14.24078	2.079442	1	2	2.079442	C35	2013
1.94591	0.428571	14.17535	1.386294	1	2	2.484907	C35	2017
1.94591	0.428571	14.27338	1.94591	1	2	2.564949	C35	2018
1.94591	0.428571	14.48718	1.386294	1	2	2.639057	C35	2019
2.197225	0.333333	15.65447	2.079442	1	4	2.197225	C36	2016
2.197225	0.333333	16.00381	2.639057	1	4	2.302585	C36	2017
2.197225	0.333333	16.20336	2.484907	1	4	2.397895	C36	2018
2.197225	0.333333	16.14588	2.564949	1	4	2.484907	C36	2019
2.197225	0.333333	13.26213	1.94591	1	3	2.833213	C32	2010
2.197225	0.333333	13.74294	2.302585	1	3	2.890372	C32	2011
2.197225	0.333333	13.89247	2.079442	1	3	2.944439	C32	2012
2.197225	0.333333	14.08554	1.791759	1	3	2.995732	C32	2013
2.197225	0.333333	14.13032	1.609438	1	3	3.044522	C32	2014
2.197225	0.333333	14.31021	1.791759	1	3	3.091042	C32	2015
2.197225	0.333333	14.40274	2.079442	1	3	3.135494	C32	2016
2.197225	0.333333	14.57332	1.609438	1	3	3.178054	C32	2017
2.197225	0.333333	14.28182	2.197225	1	3	3.218876	C32	2018
2.197225	0.333333	14.81651	2.397895	1	3	3.258097	C32	2019
1.609438	0.4	16.31284	2.197225	1	2	2.944439	K70	2015
2.197225	0.333333	16.46946	2.639057	1	2	2.995732	K70	2016
2.197225	0.333333	16.70588	2.302585	1	2	3.044522	K70	2017
2.197225	0.333333	16.70588	2.564949	1	2	3.091042	K70	2018
2.197225	0.333333	16.70588	2.564949	1	2	3.135494	K70	2019
2.197225	0.333333	14.29806	2.397895	1	3	2.70805	C29	2016
2.197225	0.333333	15.55324	2.197225	1	3	2.772589	C29	2017
2.197225	0.333333	16.03602	1.94591	1	3	2.890372	C29	2019
1.94591	0.428571	13.74884	1.94591	1	4	3.091042	C26	2016
1.94591	0.428571	14.01853	1.609438	1	4	3.135494	C26	2017
1.94591	0.428571	14.33389	1.386294	1	4	3.178054	C26	2018
1.94591	0.428571	14.70151	1.791759	1	4	3.218876	C26	2019
2.397895	0.363636	14.123	2.397895	1	3	2.079442	C35	2011
2.397895	0.363636	13.97251	2.197225	1	3	2.197225	C35	2012
2.079442	0.5	13.98102	1.94591	1	3	2.302585	C35	2013
1.94591	0.428571	13.98102	2.197225	1	3	2.397895	C35	2014
1.94591	0.428571	13.98102	1.609438	1	3	2.484907	C35	2015
1.791759	0.5	13.98102	1.94591	1	3	2.564949	C35	2016
1.94591	0.428571	13.98102	1.94591	1	3	2.639057	C35	2017
1.791759	0.5	13.68881	1.609438	1	3	2.70805	C35	2018
1.94591	0.428571	13.81551	1.791759	1	3	2.772589	C35	2019
2.197225	0.333333	14.25377	2.197225	1	3	2.079442	C39	2011
2.079442	0.375	14.25377	2.197225	1	4	2.197225	C39	2012
2.197225	0.333333	14.31629	2.197225	1	4	2.302585	C39	2013
2.197225	0.333333	14.25377	2.197225	1	4	2.397895	C39	2014
2.197225	0.333333	14.31629	2.197225	1	4	2.484907	C39	2015
2.197225	0.333333	14.31629	2.197225	1	3	2.564949	C39	2016
2.197225	0.333333	14.31629	2.197225	1	3	2.639057	C39	2017
2.197225	0.333333	14.31629	2.197225	1	3	2.70805	C39	2018
2.197225	0.333333	14.62644	2.197225	1	3	2.772589	C39	2019
2.639057	0.357143	14.20863	2.397895	1	3	2.397895	F52	2014
2.564949	0.384615	14.38372	2.079442	1	3	2.484907	F52	2015
2.639057	0.357143	14.37209	2.302585	1	3	2.564949	F52	2016
2.639057	0.357143	14.42239	2.079442	1	3	2.639057	F52	2017
2.197225	0.333333	13.14236	1.791759	1	2	2.833213	C38	2011
2.197225	0.333333	13.89145	2.197225	1	2	2.890372	C38	2012
2.197225	0.333333	13.77281	2.079442	1	2	2.944439	C38	2013
2.197225	0.333333	13.8108	2.302585	1	2	2.995732	C38	2014

2.197225	0.333333	13.858	2.564949	1	2	3.044522	C38	2015
2.197225	0.333333	14.09534	2.70805	1	2	3.091042	C38	2016
2.197225	0.333333	14.09276	2.484907	1	2	3.135494	C38	2017
2.197225	0.333333	14.12652	2.772589	1	2	3.178054	C38	2018
2.197225	0.333333	14.30679	2.397895	1	2	3.218876	C38	2019
2.079442	0.375	13.8643	1.94591	1	3	2.944439	G58	2011
2.079442	0.375	13.94126	2.197225	1	3	2.995732	C34	2012
2.079442	0.375	14.00282	2.397895	1	3	3.044522	C34	2013
2.079442	0.375	14.28626	2.079442	1	3	3.091042	C34	2014
2.079442	0.375	14.32173	2.197225	1	3	3.135494	C34	2015
2.079442	0.375	14.32425	2.079442	1	3	3.178054	C34	2016
2.079442	0.375	14.33472	2.197225	1	3	3.218876	C34	2017
2.197225	0.333333	14.98647	2.197225	1	3	2.944439	F52	2017
2.197225	0.333333	14.91486	2.302585	1	3	2.995732	F52	2018
2.197225	0.333333	15.12279	2.197225	1	3	3.044522	F52	2019
2.079442	0.375	13.99933	2.197225	1	1	2.833213	C32	2018
2.079442	0.375	14.09201	2.302585	1	1	2.890372	C32	2019
1.94591	0.428571	13.3739	2.302585	1	1	2.639057	C29	2016
1.94591	0.428571	14.39912	2.197225	1	1	2.70805	C29	2017
1.94591	0.428571	14.44822	2.302585	1	1	2.772589	C29	2018
1.94591	0.428571	14.84638	2.772589	1	1	2.833213	C29	2019
2.197225	0.333333	14.76632	2.772589	1	2	3.433987	C23	2014
1.94591	0.428571	14.51076	2.772589	1	2	3.465736	C23	2015
1.94591	0.428571	14.50264	2.772589	1	2	3.496508	C23	2016
1.94591	0.428571	14.57608	2.772589	1	2	3.526361	C23	2017
1.94591	0.428571	14.68948	2.772589	1	2	3.555348	C23	2018
2.197225	0.333333	14.18707	2.197225	1	2	3.044522	C15	2014
2.197225	0.333333	14.49861	2.079442	1	2	3.091042	C15	2015
2.197225	0.333333	14.51861	2.302585	1	2	3.135494	C15	2016
2.197225	0.333333	14.4782	1.609438	1	2	3.178054	C15	2017
2.197225	0.333333	14.43609	1.386294	1	2	3.218876	C15	2018
2.197225	0.333333	14.45736	2.397895	1	2	3.258097	C15	2019
2.397895	0.363636	15.97271	2.639057	1	3	2.484907	C34	2018
2.397895	0.363636	15.92962	2.079442	1	3	2.564949	C34	2019
2.397895	0.363636	15.11922	2.079442	1	3	1.098612	C38	2010
2.397895	0.363636	14.68219	2.079442	1	3	1.386294	C38	2011
2.397895	0.363636	14.66267	1.609438	0	1	1.609438	C38	2012
2.397895	0.363636	14.66566	2.079442	0	1	1.791759	C38	2013
2.079442	0.375	15.23258	2.197225	0	1	1.94591	C38	2014
2.197225	0.333333	14.78818	1.94591	0	1	2.079442	C38	2015
2.197225	0.333333	15.00758	2.397895	0	1	2.197225	C38	2016
2.197225	0.333333	14.87434	1.609438	0	1	2.302585	C38	2017
2.079442	0.375	14.93882	1.386294	0	1	2.397895	C38	2018
1.94591	0.428571	15.08808	2.484907	0	1	2.484907	C38	2019
2.079442	0.375	13.30468	1.791759	1	2	2.639057	C32	2011
2.197225	0.444444	13.54107	1.94591	1	2	2.70805	C32	2012
2.079442	0.375	13.54107	1.94591	1	2	2.772589	C32	2013
1.94591	0.428571	13.61706	2.484907	1	2	2.833213	C32	2014
1.94591	0.428571	13.68859	2.70805	1	2	2.890372	C32	2015
1.791759	0.333333	13.70368	2.70805	1	2	2.944439	C32	2016
1.94591	0.428571	13.66956	2.564949	1	2	2.995732	C32	2017
1.94591	0.428571	13.75491	2.484907	1	2	3.044522	C32	2018
1.94591	0.428571	13.70077	2.484907	1	2	3.091042	C32	2019
2.197225	0.333333	12.91425	2.302585	1	3	1.098612	S90	2010
2.484907	0.333333	12.90744	2.197225	1	3	1.386294	S90	2011
2.484907	0.333333	13.17477	2.302585	1	3	1.609438	C26	2012
2.484907	0.333333	13.39954	1.94591	1	3	1.791759	C26	2013
2.484907	0.333333	13.42587	1.609438	1	3	1.94591	C26	2014

2.484907	0.333333	13.71392	1.791759	1	3	2.079442	C26	2015
2.397895	0.363636	13.68244	1.94591	1	3	2.197225	C26	2016
2.484907	0.333333	13.95048	1.791759	1	3	2.302585	C26	2017
2.397895	0.363636	14.0299	2.197225	1	3	2.397895	C26	2018
2.197225	0.444444	13.98576	1.791759	1	3	2.484907	C26	2019
2.197225	0.333333	13.93649	2.079442	1	2	2.890372	C33	2011
2.197225	0.333333	14.04185	2.302585	1	2	2.944439	C33	2012
1.94591	0.428571	14.17179	2.079442	1	2	2.995732	C33	2013
1.94591	0.428571	14.18293	1.94591	1	2	3.044522	C33	2014
1.94591	0.428571	14.22616	1.791759	1	2	3.091042	C33	2015
1.94591	0.428571	14.30409	1.791759	1	2	3.135494	C33	2016
1.94591	0.428571	14.27767	1.94591	1	2	3.178054	C33	2017
1.94591	0.428571	14.25377	1.94591	1	2	3.218876	C33	2018
1.94591	0.428571	14.31629	1.791759	1	2	3.258097	C33	2019
2.484907	0.333333	14.21964	1.609438	1	1	2.564949	C36	2010
2.484907	0.333333	14.7516	2.302585	1	1	2.639057	C36	2011
2.484907	0.333333	14.48846	2.484907	1	1	2.70805	C36	2012
2.484907	0.333333	14.56693	2.484907	1	1	2.772589	C36	2013
2.484907	0.333333	14.56693	2.484907	1	1	2.833213	C36	2014
2.772589	0.375	14.56006	2.70805	1	1	2.890372	C36	2015
2.772589	0.375	14.97584	2.70805	1	1	2.944439	C36	2016
2.833213	0.352941	15.10593	2.772589	1	1	2.995732	C36	2017
2.833213	0.352941	14.64406	2.639057	1	1	3.044522	C36	2018
1.94591	0.428571	14.7529	1.94591	1	4	2.70805	C36	2015
1.94591	0.428571	14.52866	2.197225	1	4	2.772589	C36	2016
1.94591	0.428571	14.57632	1.386294	1	4	2.833213	C36	2017
1.94591	0.428571	14.57098	1.386294	1	4	2.890372	C36	2018
1.94591	0.428571	14.47768	1.609438	1	4	2.944439	C36	2019
2.484907	0.333333	16.82934	3.135494	1	4	2.302585	L72	2017
2.484907	0.333333	16.46898	3.258097	1	4	2.397895	L72	2018
2.484907	0.333333	16.25569	3.178054	1	4	2.484907	L72	2019
1.94591	0.428571	14.66797	1.94591	1	1	2.995732	C30	2018
1.94591	0.428571	14.7767	2.302585	1	1	3.044522	C30	2019
1.791759	0.333333	14.86913	2.302585	1	4	2.944439	C38	2016
1.791759	0.333333	14.86982	2.079442	1	4	2.995732	C38	2017
2.197225	0.333333	14.86982	2.079442	1	4	3.044522	C38	2018
2.197225	0.333333	14.89732	2.302585	1	4	3.091042	C38	2019
2.197225	0.333333	14.29794	1.609438	1	4	2.639057	C34	2016
2.197225	0.333333	14.39212	1.609438	1	4	2.70805	C34	2017
2.197225	0.333333	14.18707	1.791759	1	4	2.772589	C34	2018
2.197225	0.333333	14.3664	1.386294	1	4	2.833213	C34	2019
2.197225	0.333333	13.54107	2.197225	1	2	2.302585	C37	2010
2.197225	0.333333	13.54107	2.197225	1	2	2.397895	C37	2011
2.197225	0.333333	13.43571	1.94591	1	2	2.484907	C37	2012
2.197225	0.333333	13.43571	1.386294	1	2	2.564949	C37	2013
2.197225	0.333333	13.43571	1.386294	1	2	2.639057	C37	2014
2.197225	0.333333	13.43571	1.386294	1	2	2.70805	C37	2015
2.197225	0.333333	13.27319	1.386294	1	2	2.772589	C37	2016
2.197225	0.333333	13.27319	1.386294	1	2	2.833213	C37	2017
2.197225	0.333333	13.27319	1.098612	1	2	2.890372	C37	2018
2.197225	0.333333	13.61008	1.386294	1	2	2.944439	C37	2019
2.197225	0.333333	14.76944	2.397895	1	2	3.091042	C29	2016
2.197225	0.333333	14.41435	2.079442	1	2	3.135494	C29	2017
2.197225	0.333333	15.1054	2.564949	1	2	3.178054	C29	2018
2.197225	0.333333	15.20652	2.397895	1	2	3.218876	C29	2019
2.079442	0.375	14.09964	1.791759	1	3	2.397895	C20	2011
2.197225	0.333333	14.62644	2.079442	1	3	2.484907	C20	2012
2.079442	0.375	14.33466	2.302585	1	3	2.564949	C20	2013

2.197225	0.333333	14.48334	1.94591	1	3	2.639057	C20	2014
2.079442	0.375	14.69098	2.079442	1	3	2.70805	C20	2015
2.197225	0.333333	14.83994	2.564949	1	3	2.772589	C20	2016
2.197225	0.333333	14.79271	2.639057	1	3	2.833213	C20	2017
2.197225	0.333333	15.58019	2.079442	1	3	2.890372	C20	2018
2.197225	0.333333	15.48122	2.197225	1	3	2.944439	C20	2019
2.197225	0.444444	13.85924	2.197225	1	2	2.397895	C19	2012
2.197225	0.444444	13.36547	1.609438	1	4	2.484907	C19	2013
2.079442	0.375	13.52596	1.386294	1	2	2.564949	C19	2014
2.079442	0.375	14.30929	1.791759	1	4	2.639057	C19	2015
2.197225	0.444444	14.30984	1.609438	1	2	2.70805	C19	2016
2.197225	0.444444	14.15725	1.098612	1	4	2.772589	C19	2017
2.197225	0.444444	14.24215	1.609438	1	2	2.833213	C19	2018
2.197225	0.333333	14.30776	1.791759	1	4	2.890372	C19	2019
2.197225	0.333333	15.18509	1.791759	1	4	3.178054	C26	2019
2.197225	0.333333	14.29292	1.94591	1	2	2.70805	F51	2012
2.197225	0.333333	14.37775	2.302585	1	2	2.772589	F51	2013
2.197225	0.333333	14.50123	2.079442	1	2	2.833213	F51	2014
2.197225	0.333333	14.33953	2.484907	1	2	2.890372	F51	2015
2.197225	0.333333	14.37604	2.564949	1	2	2.944439	F51	2016
1.94591	0.428571	14.35807	2.302585	1	2	2.995732	F51	2017
1.94591	0.428571	15.11975	2.639057	1	2	3.044522	F51	2018
1.94591	0.428571	15.30326	2.484907	1	2	3.091042	F51	2019
2.197225	0.333333	14.352	2.397895	1	4	2.397895	C36	2014
2.197225	0.333333	14.33882	1.94591	1	4	2.484907	C36	2015
2.197225	0.333333	14.35364	2.079442	1	4	2.564949	C36	2016
2.197225	0.333333	14.10534	2.197225	1	4	2.639057	C36	2017
2.197225	0.333333	14.28376	2.302585	1	4	2.70805	C36	2018
2.079442	0.375	14.34219	1.791759	1	4	2.772589	C36	2019
1.94591	0.428571	13.81621	2.197225	1	1	2.564949	E48	2016
1.94591	0.428571	14.17807	2.890372	1	1	2.639057	E48	2017
1.94591	0.428571	14.04487	2.564949	1	1	2.70805	E48	2018
1.94591	0.428571	13.9622	1.791759	1	1	2.772589	E48	2019
1.94591	0.428571	13.68995	1.791759	1	3	2.772589	C21	2012
1.94591	0.428571	14.00733	2.079442	1	3	2.833213	C21	2013
1.94591	0.428571	13.83531	1.609438	1	3	2.890372	C21	2014
1.94591	0.428571	13.58257	2.397895	1	3	2.944439	C21	2015
1.94591	0.428571	13.49351	2.397895	1	3	2.995732	C21	2016
1.94591	0.428571	13.58924	2.079442	1	3	3.044522	C21	2017
1.94591	0.428571	13.63806	2.397895	1	3	3.091042	C21	2018
2.197225	0.333333	13.73213	1.791759	1	3	3.135494	C21	2019
1.609438	0.4	14.64406	2.397895	1	2	2.484907	C36	2014
1.609438	0.4	14.92077	2.302585	1	2	2.564949	C36	2015
1.791759	0.5	14.9007	2.302585	1	2	2.639057	C36	2016
1.609438	0.4	14.94339	2.197225	1	2	2.70805	C36	2017
1.609438	0.4	14.97553	2.484907	1	2	2.772589	C36	2018
1.609438	0.4	15.14553	2.302585	1	2	2.833213	C36	2019
2.397895	0.363636	14.36375	2.079442	1	2	2.639057	C26	2014
2.302585	0.4	14.51324	1.94591	1	2	2.70805	C26	2015
1.94591	0.428571	15.1478	2.484907	1	2	2.772589	C26	2016
1.94591	0.428571	14.70369	2.079442	1	2	2.833213	C26	2017
1.94591	0.428571	15.07363	2.484907	1	2	2.890372	C26	2018
1.94591	0.428571	15.15484	2.197225	1	2	2.944439	C26	2019
1.94591	0.428571	14.62319	2.302585	1	4	2.397895	C35	2014
1.94591	0.428571	14.39403	2.197225	1	4	2.484907	C35	2015
1.94591	0.428571	14.34908	1.94591	1	4	2.564949	C35	2016
1.791759	0.333333	14.17542	2.079442	1	4	2.639057	C35	2017
1.791759	0.333333	14.26097	2.079442	1	4	2.70805	C35	2018

1.791759	0.333333	14.57445	1.94591	1	4	2.772589	C35	2019
2.197225	0.333333	14.33347	1.609438	1	2	2.079442	C38	2016
2.197225	0.333333	14.73208	2.70805	1	2	2.197225	C38	2017
2.197225	0.333333	14.62279	2.197225	1	2	2.302585	C38	2018
2.197225	0.333333	14.67574	1.609438	1	2	2.397895	C38	2019
1.791759	0.5	13.12236	1.94591	1	2	2.833213	C30	2015
1.609438	0.6	13.54107	2.564949	1	2	2.890372	C30	2016
1.609438	0.6	13.54107	1.94591	1	2	2.944439	C30	2017
1.94591	0.428571	13.54107	2.397895	1	2	2.995732	C30	2018
2.197225	0.333333	14.01436	1.609438	1	3	2.772589	C22	2015
2.197225	0.333333	14.30709	1.609438	1	3	2.833213	C22	2016
2.197225	0.333333	14.36062	1.386294	1	3	2.890372	C22	2017
2.197225	0.333333	14.62261	1.609438	1	3	2.944439	C22	2018
2.079442	0.375	14.63573	1.386294	1	3	2.995732	C22	2019
1.94591	0.428571	13.98323	2.197225	1	2	2.70805	C39	2015
1.94591	0.428571	13.79429	1.94591	1	2	2.772589	C39	2016
1.94591	0.428571	13.68904	2.197225	1	2	2.833213	C39	2017
1.94591	0.428571	13.80708	1.791759	1	2	2.890372	C39	2018
1.94591	0.428571	13.91608	1.94591	1	2	2.944439	C39	2019
2.197225	0.333333	13.019	2.397895	1	4	2.564949	G54	2016
2.197225	0.333333	13.36075	2.302585	1	4	2.639057	G54	2017
2.197225	0.333333	14.60401	2.484907	1	4	2.772589	G54	2019
2.197225	0.333333	15.08572	1.386294	1	2	2.995732	C36	2016
2.197225	0.333333	15.125	2.197225	1	2	3.044522	C36	2017
2.197225	0.333333	15.14523	2.397895	1	2	3.091042	C36	2018
2.197225	0.333333	15.26408	2.484907	1	2	3.135494	C36	2019
2.197225	0.333333	14.94984	2.397895	1	2	2.944439	C38	2018
2.197225	0.333333	15.11333	2.302585	1	2	2.995732	C38	2019
2.197225	0.333333	15.16465	2.197225	1	2	2.833213	C17	2018
2.197225	0.333333	15.2127	2.197225	1	2	2.890372	C17	2019
1.94591	0.428571	14.2245	1.386294	1	2	2.944439	C23	2016
1.94591	0.428571	14.25647	2.079442	1	2	2.995732	C23	2017
1.94591	0.428571	14.2557	2.397895	1	3	3.044522	C23	2018
1.609438	0.6	14.25576	1.94591	1	3	3.091042	C23	2019
1.94591	0.428571	15.04026	2.397895	1	1	2.995732	C14	2019
2.197225	0.333333	14.13105	2.079442	1	2	2.639057	D45	2017
2.197225	0.333333	14.10061	2.397895	1	2	2.70805	D45	2018
2.197225	0.333333	13.98643	2.302585	1	2	2.772589	D45	2019
2.197225	0.333333	14.44572	1.791759	1	3	2.772589	C35	2018
1.94591	0.428571	14.49405	2.302585	1	3	2.833213	C35	2019
2.197225	0.333333	13.8472	2.302585	1	2	2.70805	C35	2015
2.197225	0.333333	14.0077	1.94591	1	2	2.772589	C35	2016
2.197225	0.333333	14.14164	2.302585	1	2	2.833213	C35	2017
2.197225	0.333333	14.28795	2.397895	1	2	2.890372	C35	2018
1.94591	0.428571	14.1839	1.94591	1	2	2.484907	C35	2014
1.94591	0.428571	13.64721	1.791759	1	2	2.564949	C35	2015
1.94591	0.428571	13.93649	1.791759	1	2	2.639057	C35	2016
1.94591	0.428571	13.83423	1.94591	1	2	2.70805	C35	2017
1.94591	0.428571	14.19388	1.791759	1	2	2.772589	C35	2018
1.94591	0.428571	14.48242	1.386294	1	2	2.833213	C35	2019
1.94591	0.428571	14.0133	1.609438	1	3	2.944439	C36	2017
1.94591	0.428571	14.46622	1.94591	1	3	2.995732	C36	2018
1.94591	0.428571	14.48036	2.197225	1	3	3.044522	C36	2019
1.609438	0.4	13.68995	1.386294	1	1	2.772589	C34	2016
1.609438	0.4	13.68026	1.791759	1	1	2.833213	C34	2017
1.609438	0.4	14.55745	2.079442	1	1	2.890372	C34	2018
1.609438	0.4	14.46533	2.079442	1	1	2.944439	C34	2019
2.079442	0.375	13.08237	2.302585	1	3	2.079442	F52	2016

2.197225	0.333333	13.69171	2.302585	1	3	2.197225	F52	2017
2.197225	0.333333	14.03119	2.944439	1	3	2.302585	F52	2018
2.197225	0.333333	14.05327	2.833213	1	3	2.397895	F52	2019
1.94591	0.428571	14.61094	2.397895	1	4	2.302585	C38	2018
1.94591	0.428571	15.02483	1.386294	1	4	2.397895	C38	2019
2.079442	0.375	14.94965	2.833213	1	4	2.772589	F51	2015
2.197225	0.333333	14.95334	3.218876	1	4	2.833213	F51	2016
2.197225	0.333333	15.17797	2.564949	1	4	2.890372	F51	2017
2.197225	0.333333	14.47773	2.197225	1	4	2.944439	F51	2018
2.397895	0.363636	14.17305	2.197225	1	3	2.564949	C25	2017
2.397895	0.363636	14.32161	2.564949	1	3	2.639057	C25	2018
2.397895	0.363636	14.65007	2.197225	1	3	2.70805	C25	2019
2.397895	0.363636	14.53291	1.94591	1	4	2.079442	L72	2015
2.302585	0.4	14.83038	2.197225	1	4	2.197225	L72	2016
2.397895	0.363636	15.04979	2.890372	1	3	2.833213	C39	2015
2.397895	0.363636	14.80327	2.484907	1	3	2.890372	C39	2016
2.197225	0.444444	14.86304	2.564949	1	3	2.944439	C39	2017
2.197225	0.444444	14.85476	2.639057	1	3	2.995732	C39	2018
2.197225	0.333333	14.63392	2.197225	1	4	3.091042	M73	2017
2.197225	0.333333	14.57907	1.791759	1	4	3.135494	M73	2018
2.197225	0.333333	14.83233	2.70805	1	4	3.178054	M73	2019
2.197225	0.333333	14.48743	1.94591	1	2	2.639057	C37	2017
2.197225	0.333333	14.73543	2.484907	1	2	2.70805	C37	2018
2.079442	0.375	14.70538	2.397895	1	2	2.772589	C37	2019
1.94591	0.428571	14.25377	1.098612	1	4	3.044522	C34	2016
1.94591	0.428571	14.25377	2.302585	1	4	3.091042	C34	2017
1.94591	0.428571	14.4307	2.079442	1	4	3.135494	C34	2018
1.94591	0.428571	14.38649	2.197225	1	4	3.178054	C34	2019
2.197225	0.333333	13.70793	1.791759	1	4	2.302585	C36	2015
2.197225	0.333333	13.80222	1.791759	1	2	2.397895	C36	2016
1.791759	0.5	13.85146	2.484907	1	2	2.484907	C36	2017
1.94591	0.428571	13.81311	2.302585	1	2	2.564949	C36	2018
1.791759	0.5	13.84196	2.397895	1	2	2.639057	C36	2019
2.197225	0.333333	13.99783	1.609438	1	2	2.944439	C35	2016
2.197225	0.333333	14.08554	1.386294	1	2	2.995732	C35	2017
2.197225	0.333333	14.08554	1.94591	1	2	3.044522	C35	2018
2.197225	0.333333	13.90626	1.098612	1	2	3.091042	C35	2019
1.94591	0.428571	14.57477	2.079442	1	3	2.70805	C36	2017
1.94591	0.428571	14.6809	2.079442	1	3	2.772589	C36	2018
1.94591	0.428571	14.59015	1.386294	1	3	2.833213	C36	2019
2.197225	0.333333	12.97849	1.94591	1	2	2.639057	C27	2014
2.197225	0.333333	13.09087	1.94591	1	2	2.70805	C27	2015
2.197225	0.333333	13.30868	2.079442	1	2	2.772589	C27	2016
2.197225	0.333333	13.59586	1.386294	1	2	2.833213	C27	2017
2.197225	0.333333	13.66213	1.609438	1	2	2.890372	C27	2018
2.197225	0.333333	13.66771	1.386294	1	2	2.944439	C27	2019
2.197225	0.333333	13.73517	2.484907	1	1	2.772589	C36	2017
2.197225	0.333333	13.94899	2.890372	1	1	2.833213	C36	2018
2.197225	0.333333	14.13564	2.197225	1	1	2.890372	C36	2019
2.197225	0.333333	14.81879	1.609438	1	3	2.639057	C26	2017
2.197225	0.333333	14.82853	2.079442	1	1	2.70805	C26	2018
2.197225	0.333333	14.76748	2.079442	1	1	2.772589	C26	2019
1.94591	0.428571	14.00984	1.609438	1	2	2.772589	C35	2018
1.94591	0.428571	14.03633	2.70805	1	2	2.833213	C35	2019
2.197225	0.333333	14.42549	2.079442	1	1	2.302585	C26	2016
1.94591	0.428571	14.46062	2.397895	1	1	2.397895	C26	2017
2.197225	0.333333	14.39167	2.484907	1	1	2.484907	C26	2018
2.397895	0.363636	13.73982	1.386294	1	1	2.484907	C15	2015

2.397895	0.363636	13.91164	1.386294	1	1	2.564949	C15	2016
2.197225	0.333333	14.04073	1.609438	1	1	2.639057	C15	2017
2.197225	0.333333	13.99842	1.609438	1	1	2.70805	C15	2018
2.197225	0.333333	14.09125	1.098612	1	1	2.772589	C15	2019
2.079442	0.375	13.7001	2.639057	1	4	2.944439	C27	2015
2.197225	0.333333	13.65581	2.302585	1	4	2.995732	C27	2016
2.197225	0.333333	14.14546	1.94591	1	4	3.044522	C27	2017
2.197225	0.333333	14.14955	2.564949	1	4	3.091042	C27	2018
1.94591	0.428571	14.13564	2.639057	1	4	3.135494	C27	2019
2.079442	0.375	14.22224	2.772589	1	2	2.833213	C28	2017
2.079442	0.375	14.47681	2.833213	1	2	2.890372	C28	2018
2.079442	0.375	14.72735	2.484907	1	2	2.944439	C28	2019
2.197225	0.333333	14.73547	2.484907	1	2	3.091042	C20	2017
2.197225	0.333333	14.74175	2.484907	1	2	3.135494	C20	2018
2.197225	0.333333	14.71847	2.484907	1	2	3.178054	C20	2019
1.94591	0.428571	15.59772	2.302585	1	4	2.890372	F52	2017
1.94591	0.428571	15.59182	2.639057	1	4	2.944439	F52	2018
1.94591	0.428571	15.51789	2.639057	1	4	2.995732	F52	2019
2.197225	0.333333	14.32851	1.94591	1	1	2.639057	C17	2016
2.197225	0.333333	13.42158	2.302585	1	1	2.70805	C17	2017
2.197225	0.333333	14.40757	1.609438	1	1	2.772589	C17	2018
2.197225	0.333333	14.53627	1.94591	1	1	2.833213	C17	2019
2.197225	0.333333	15.34928	1.609438	1	4	3.044522	C30	2019
1.94591	0.428571	13.97933	2.302585	1	2	2.484907	C26	2017
1.94591	0.428571	14.49856	2.197225	1	2	2.564949	C26	2018
2.079442	0.375	14.17779	1.791759	1	2	2.639057	C26	2019
2.197225	0.333333	14.85561	2.70805	1	2	2.890372	C39	2019
1.791759	0.5	13.86544	1.609438	1	4	2.564949	C30	2015
1.791759	0.5	14.01288	2.079442	1	4	2.639057	C30	2016
1.94591	0.428571	14.16441	2.833213	1	4	2.70805	C30	2017
2.197225	0.333333	14.23823	1.609438	1	3	2.639057	C33	2017
2.197225	0.333333	14.15676	2.302585	1	3	2.70805	C33	2018
2.197225	0.333333	14.22397	2.302585	1	3	2.772589	C33	2019
2.079442	0.375	14.51419	2.397895	1	3	2.197225	C34	2019
2.197225	0.333333	14.10369	2.079442	1	3	2.944439	C36	2017
2.079442	0.375	14.15697	1.609438	1	3	2.995732	C36	2018
2.079442	0.375	14.17179	2.302585	1	3	3.044522	C36	2019
2.197225	0.333333	13.99207	2.833213	1	4	3.044522	C40	2018
2.197225	0.333333	14.22118	2.833213	1	4	3.091042	C40	2019
2.197225	0.333333	14.38613	2.302585	1	4	2.995732	C35	2015
2.197225	0.333333	14.60518	1.791759	1	4	3.044522	C35	2016
2.197225	0.333333	14.48294	2.197225	1	4	3.091042	C35	2017
2.079442	0.375	14.56212	1.94591	1	4	3.135494	C35	2018
2.197225	0.333333	14.70988	1.609438	1	4	3.178054	C35	2019
2.197225	0.333333	12.89747	1.609438	1	4	3.091042	C36	2016
2.197225	0.333333	13.66376	2.079442	1	4	3.135494	C36	2017
2.197225	0.333333	13.85608	2.079442	1	4	3.178054	C36	2018
2.197225	0.333333	14.05311	1.791759	1	4	3.218876	C36	2019
1.94591	0.428571	12.75564	2.079442	1	2	3.178054	C34	2017
1.94591	0.428571	12.98195	1.791759	1	2	3.218876	C34	2018
1.94591	0.428571	13.43366	1.791759	1	4	3.258097	C34	2019
1.94591	0.428571	14.32263	1.386294	1	2	2.890372	C34	2018
1.94591	0.428571	14.31707	1.386294	1	2	2.944439	C34	2019
1.94591	0.428571	14.48859	1.791759	1	3	2.639057	C26	2016
1.94591	0.428571	14.44992	2.302585	1	3	2.70805	C26	2017
1.94591	0.428571	13.98946	1.609438	1	3	2.772589	C26	2018
1.94591	0.428571	14.14222	2.484907	1	3	2.833213	C26	2019
1.94591	0.428571	13.92005	1.791759	1	4	2.833213	C38	2017

2.397895	0.545455	14.29032	2.197225	1	4	2.890372	C38	2018
2.302585	0.5	14.37255	1.94591	1	4	2.944439	C38	2019
2.197225	0.444444	14.39588	1.609438	1	4	2.70805	C28	2018
2.197225	0.333333	14.51548	2.079442	1	4	2.772589	C28	2019
1.94591	0.428571	14.45435	1.791759	1	2	2.772589	C36	2017
1.94591	0.428571	15.4343	2.564949	1	2	2.833213	C36	2018
1.94591	0.428571	14.84349	2.302585	1	2	2.890372	C36	2019
1.94591	0.428571	13.34503	2.302585	1	3	3.178054	C13	2016
1.94591	0.428571	13.50626	2.079442	1	3	3.218876	C13	2017
1.94591	0.428571	13.55609	2.302585	1	3	3.258097	C13	2018
1.94591	0.428571	13.60405	2.079442	1	3	3.295837	C13	2019
2.197225	0.333333	13.72777	2.079442	1	3	2.302585	C35	2015
2.197225	0.333333	13.91897	2.397895	1	3	2.397895	C35	2016
2.197225	0.333333	13.96134	2.197225	1	3	2.484907	C35	2017
2.197225	0.333333	14.3318	2.397895	1	3	2.564949	C35	2018
2.197225	0.333333	14.34849	1.791759	1	3	2.639057	C35	2019
2.197225	0.333333	13.98719	2.302585	1	1	3.258097	C34	2016
2.197225	0.333333	13.97277	2.302585	1	1	3.295837	C34	2017
2.197225	0.333333	14.13556	2.833213	1	1	3.332205	C34	2018
2.197225	0.333333	13.96591	1.609438	1	1	3.367296	C34	2019
1.94591	0.428571	15.17849	2.302585	1	2	2.995732	C33	2018
1.94591	0.428571	15.16161	2.302585	1	2	3.044522	C33	2019
1.94591	0.428571	14.4859	2.484907	1	3	2.639057	C34	2019
2.197225	0.333333	14.67065	1.791759	1	2	2.70805	C36	2017
2.197225	0.333333	14.84152	1.386294	1	2	2.772589	C36	2018
2.197225	0.333333	14.81065	2.397895	1	2	2.833213	C36	2019
2.197225	0.333333	13.60479	2.302585	1	2	2.772589	E48	2017
2.197225	0.333333	13.68768	2.995732	1	2	2.833213	E48	2018
2.197225	0.333333	13.76422	2.197225	1	2	2.890372	E48	2019
2.197225	0.333333	14.76004	2.079442	1	4	2.564949	C26	2016
2.197225	0.333333	14.77394	2.397895	1	4	2.639057	C26	2017
2.197225	0.333333	14.77394	2.302585	1	4	2.70805	C26	2018
2.197225	0.333333	14.68261	1.94591	1	4	2.833213	C17	2017
2.197225	0.333333	14.81393	1.94591	1	4	2.890372	C17	2018
2.197225	0.333333	14.71973	2.197225	1	4	2.944439	C17	2019
1.94591	0.428571	14.5542	1.94591	1	3	2.995732	C27	2018
1.94591	0.428571	14.74223	1.94591	1	3	3.044522	C27	2019
1.791759	0.5	14.37318	2.302585	1	4	2.197225	C26	2018
1.791759	0.5	15.27422	1.94591	1	4	2.302585	C26	2019
2.197225	0.333333	14.59217	1.609438	1	1	2.890372	C26	2019
2.079442	0.375	15.94126	2.079442	1	2	3.526361	C30	2017
2.197225	0.333333	15.88399	2.397895	1	2	3.555348	C30	2018
2.197225	0.333333	14.68358	1.94591	1	2	2.772589	C21	2016
2.197225	0.333333	14.86483	1.791759	1	2	2.833213	C21	2017
2.197225	0.333333	14.91898	2.484907	1	3	3.218876	C39	2019
2.197225	0.333333	14.47495	2.302585	1	3	2.772589	C27	2014
2.197225	0.333333	14.55745	2.397895	1	3	2.833213	C27	2015
2.197225	0.333333	14.59474	1.94591	1	3	2.890372	C27	2016
2.197225	0.333333	14.77102	2.079442	1	3	2.944439	C27	2017
2.197225	0.333333	14.86353	2.397895	1	3	2.995732	C27	2018
2.197225	0.333333	15.06613	2.079442	1	3	3.044522	C27	2019
2.197225	0.333333	15.20088	1.94591	1	4	2.995732	C38	2018
2.197225	0.333333	15.47078	1.94591	1	4	3.044522	C38	2019
2.197225	0.333333	14.13309	2.079442	1	2	2.995732	C37	2017
2.197225	0.333333	14.52998	1.94591	1	2	3.044522	C37	2018
2.197225	0.333333	14.52104	1.94591	1	2	3.091042	C37	2019
2.197225	0.333333	14.41972	2.564949	1	3	2.302585	C39	2017
2.197225	0.333333	14.19162	2.70805	1	3	2.397895	C39	2018

2.197225	0.333333	14.18126	2.564949	1	3	2.484907	C39		2019
2.197225	0.333333	14.80717	1.609438	1	2	2.833213	I65		2015
2.197225	0.333333	14.82795	1.791759	1	2	2.890372	C37		2016
2.197225	0.333333	14.20498	1.94591	1	2	2.944439	C37		2017
2.197225	0.333333	14.32118	2.302585	1	2	2.995732	C37		2018
2.197225	0.333333	14.42103	2.397895	1	2	3.044522	C37		2019
1.94591	0.428571	13.62749	1.94591	0	1	3.044522	C38		2015
1.94591	0.428571	13.79836	1.94591	0	1	3.091042	C38		2016
1.94591	0.428571	14.1484	1.791759	0	1	3.135494	C38		2017
1.94591	0.428571	14.14805	2.079442	0	1	3.178054	C38		2018
1.94591	0.428571	14.46898	2.397895	0	1	3.218876	C38		2019
2.197225	0.333333	15.13655	1.609438	1	2	2.890372	C27		2015
2.079442	0.375	15.10469	2.197225	1	2	2.944439	C27		2016
1.94591	0.428571	15.00646	2.197225	1	2	2.995732	C27		2017
1.94591	0.428571	15.1006	2.564949	1	2	3.044522	C27		2018
1.94591	0.428571	14.94865	2.302585	1	2	3.091042	C27		2019
2.397895	0.545455	14.11665	1.94591	1	1	2.833213	G59		2017
2.302585	0.5	14.20721	2.079442	1	1	2.890372	G59		2018
2.197225	0.444444	14.46908	2.484907	1	1	2.944439	G59		2019
1.94591	0.428571	15.36307	2.397895	1	3	2.70805	C38		2019
2.197225	0.333333	14.65807	2.079442	1	3	2.70805	C40		2016
1.94591	0.428571	14.83486	2.079442	1	3	2.772589	C40		2017
1.94591	0.428571	14.81967	2.197225	1	3	2.833213	C40		2018
2.197225	0.333333	14.97994	2.302585	1	3	2.890372	C40		2019
2.197225	0.333333	13.97269	2.302585	1	3	3.091042	C17		2015
2.197225	0.333333	14.28489	1.609438	1	3	3.135494	C17		2016
1.94591	0.428571	14.27572	2.197225	1	3	3.178054	C17		2017
1.94591	0.428571	14.38852	2.079442	1	3	3.218876	C17		2018
1.94591	0.428571	14.86556	1.94591	1	3	3.258097	C17		2019
1.609438	0.4	14.26525	2.397895	1	4	2.944439	C27		2015
1.609438	0.4	14.30697	2.564949	1	4	2.995732	C27		2016
1.609438	0.4	14.11806	1.94591	1	4	3.044522	C27		2017
1.609438	0.4	14.50691	1.791759	1	4	3.091042	C27		2018
1.791759	0.333333	14.78754	1.791759	1	4	3.135494	C27		2019
2.197225	0.333333	13.18459	2.197225	1	1	2.890372	C30		2017
2.197225	0.333333	13.21876	2.397895	1	2	2.944439	C30		2018
2.197225	0.333333	13.48784	2.079442	1	1	2.995732	C30		2019
2.197225	0.333333	15.145	2.079442	1	2	2.70805	C35		2017
2.197225	0.333333	14.95843	1.609438	1	2	2.772589	C35		2018
2.197225	0.333333	14.69845	1.791759	1	2	2.833213	C35		2019
2.197225	0.333333	13.67969	1.791759	1	4	2.995732	C29		2017
2.197225	0.333333	13.86173	1.791759	1	4	3.044522	C29		2018
2.197225	0.333333	13.89108	1.791759	1	4	3.091042	C29		2019
2.197225	0.333333	14.44439	1.386294	1	1	3.091042	C26		2016
2.197225	0.333333	14.48759	2.302585	1	1	3.135494	C26		2017
2.197225	0.333333	14.54663	2.302585	1	1	3.178054	C26		2018
2.197225	0.333333	14.57734	1.791759	1	1	3.218876	C26		2019
2.197225	0.333333	14.23986	2.197225	1	2	2.890372	C36		2017
2.197225	0.333333	13.77708	2.484907	1	2	2.944439	C36		2018
2.197225	0.333333	15.35223	2.197225	1	2	2.995732	C36		2019
2.197225	0.333333	15.21232	1.386294	1	4	2.772589	C18		2018
2.197225	0.333333	15.02932	1.609438	1	4	2.833213	C18		2019
2.197225	0.333333	14.86672	2.079442	0	5	2.70805	C27		2018
2.197225	0.333333	14.93088	1.94591	0	5	2.772589	C27		2019
1.609438	0.4	13.94619	2.302585	1	3	2.197225	C39		2017
1.609438	0.4	13.9861	2.197225	1	3	2.302585	C39		2018
1.609438	0.4	14.38474	2.302585	1	3	2.397895	C39		2019
2.197225	0.333333	13.2213	2.079442	1	2	2.70805	C26		2015

2.197225	0.333333	13.15386	2.197225	1	2	2.772589	C26	2016
2.197225	0.333333	13.58232	2.302585	1	2	2.833213	C26	2017
2.197225	0.333333	13.61217	2.302585	1	2	2.890372	C26	2018
2.197225	0.333333	13.78711	1.94591	1	2	2.944439	C26	2019
1.609438	0.4	15.03708	1.94591	1	1	2.484907	C26	2018
1.609438	0.4	15.12678	1.94591	1	1	2.564949	C26	2019
2.197225	0.333333	14.15108	1.94591	1	3	2.772589	C38	2014
2.197225	0.333333	14.09367	1.386294	1	3	2.833213	C38	2015
2.079442	0.375	13.88326	2.079442	1	3	2.890372	C38	2016
2.197225	0.333333	13.94777	2.079442	1	3	2.944439	C38	2017
2.197225	0.333333	14.34802	2.302585	1	2	2.995732	C38	2018
2.197225	0.333333	14.34596	2.079442	1	2	3.044522	C38	2019
1.94591	0.428571	16.30777	1.94591	1	3	2.639057	C21	2019
2.397895	0.363636	14.11606	2.197225	1	1	2.70805	C34	2015
2.397895	0.363636	14.11562	2.197225	1	1	2.772589	C34	2016
2.397895	0.363636	13.8643	2.197225	1	1	2.833213	C34	2017
2.397895	0.363636	14.28551	1.94591	1	1	2.890372	C34	2018
2.397895	0.363636	14.98178	2.564949	1	1	2.944439	C34	2019
2.197225	0.333333	13.95109	1.791759	1	1	2.639057	C34	2017
2.197225	0.333333	14.19873	2.079442	1	1	2.70805	C34	2018
2.197225	0.333333	14.1868	1.791759	1	1	2.772589	C34	2019
2.197225	0.333333	13.78009	2.197225	1	2	2.564949	C38	2015
2.197225	0.333333	13.67831	1.94591	1	2	2.639057	C38	2016
2.197225	0.333333	13.75395	2.397895	1	2	2.70805	C38	2017
2.197225	0.333333	13.72591	2.639057	1	2	2.772589	C38	2018
2.197225	0.333333	13.94777	1.791759	1	2	2.833213	C38	2019
2.197225	0.333333	14.53335	2.197225	1	2	1.94591	C33	2017
2.197225	0.333333	14.51861	2.302585	1	2	2.079442	C33	2018
2.197225	0.333333	14.51365	2.197225	1	2	2.197225	C33	2019
1.94591	0.428571	14.14474	2.484907	1	4	2.772589	C26	2017
1.94591	0.428571	14.19948	2.484907	1	4	2.833213	C26	2018
1.94591	0.428571	14.13018	2.079442	1	4	2.890372	C26	2019
1.94591	0.428571	13.73213	1.791759	1	3	2.639057	C35	2017
1.94591	0.428571	13.76422	1.791759	1	3	2.70805	C35	2018
2.197225	0.333333	14.47023	1.94591	1	3	2.772589	C35	2019
2.197225	0.333333	14.60451	2.397895	1	4	2.890372	C26	2017
2.197225	0.333333	14.5433	2.833213	1	4	2.944439	C26	2018
2.197225	0.333333	14.64459	2.397895	1	4	2.995732	C26	2019
1.94591	0.428571	14.99198	1.098612	1	2	2.484907	C29	2017
1.94591	0.428571	15.01854	1.791759	1	2	2.564949	C29	2018
1.94591	0.428571	15.00006	1.386294	1	2	2.639057	C29	2019
1.94591	0.428571	15.15628	1.386294	1	1	2.890372	C29	2018
1.94591	0.428571	14.506	1.94591	1	1	2.944439	C29	2019
2.197225	0.333333	14.24729	1.791759	0	2	2.944439	C21	2017
2.197225	0.333333	14.24729	2.302585	0	2	2.995732	C21	2018
2.197225	0.333333	14.3343	1.94591	0	2	3.044522	C21	2019
2.197225	0.333333	13.58484	2.197225	1	4	3.218876	C30	2016
2.197225	0.333333	13.83727	2.197225	1	4	3.258097	C30	2017
2.197225	0.333333	13.97669	2.197225	1	4	3.295837	C30	2018
2.197225	0.333333	13.9955	1.94591	1	4	3.332205	C30	2019
2.197225	0.333333	13.95501	2.397895	1	3	2.397895	C17	2017
2.197225	0.333333	14.10758	2.70805	1	3	2.484907	C17	2018
2.197225	0.333333	14.1743	2.302585	1	3	2.564949	C17	2019
2.197225	0.333333	14.18314	2.079442	1	5	2.484907	C13	2017
2.197225	0.333333	14.27376	2.70805	1	5	2.564949	C13	2018
2.197225	0.333333	14.58659	2.397895	1	5	2.639057	C13	2019
2.197225	0.333333	14.67409	2.302585	1	2	3.044522	C38	2017
2.197225	0.333333	14.66061	2.639057	1	2	3.091042	C38	2018

2.197225	0.333333	14.76906	2.772589	1	2	3.135494	C38	2019
2.197225	0.333333	14.40939	1.94591	1	4	2.079442	C39	2015
1.94591	0.428571	14.64275	2.302585	1	4	2.197225	C39	2016
1.94591	0.428571	14.7767	1.94591	1	4	2.302585	C39	2017
1.94591	0.428571	14.69493	1.94591	1	4	2.397895	C39	2018
1.94591	0.428571	14.75848	2.397895	1	4	2.484907	C39	2019
2.197225	0.333333	14.25891	2.197225	1	3	2.564949	C38	2017
2.397895	0.363636	14.01992	2.397895	1	3	2.639057	C38	2018
2.397895	0.363636	14.14121	2.484907	1	2	2.70805	C38	2019
2.197225	0.333333	14.71608	2.397895	1	2	2.70805	C37	2018
2.197225	0.333333	14.94691	2.564949	1	2	2.772589	C37	2019
2.197225	0.333333	14.24859	2.079442	1	4	2.484907	C26	2018
1.94591	0.428571	14.6515	2.197225	1	4	2.564949	C26	2019
2.197225	0.333333	14.37787	2.302585	1	2	2.833213	C38	2018
2.197225	0.333333	14.38971	1.609438	1	2	2.890372	C38	2019
1.94591	0.428571	14.15889	2.397895	1	1	2.70805	C22	2019
1.94591	0.428571	13.84313	2.197225	1	3	2.995732	C14	2015
1.94591	0.428571	13.88223	2.197225	1	3	3.044522	C14	2016
1.94591	0.428571	13.88223	2.197225	1	3	3.091042	C14	2017
1.94591	0.428571	13.97508	2.197225	1	3	3.135494	C14	2018
1.94591	0.428571	14.29485	2.197225	1	3	3.178054	C14	2019
1.94591	0.428571	14.36813	2.079442	1	3	2.484907	C14	2019
2.197225	0.333333	13.55233	2.397895	1	3	2.772589	C38	2016
2.197225	0.333333	13.66143	1.791759	1	3	2.833213	C38	2017
2.079442	0.375	13.687	2.302585	1	1	2.890372	C38	2018
2.197225	0.333333	13.98306	2.397895	1	1	2.944439	C38	2019
2.197225	0.333333	14.05461	2.397895	1	2	2.564949	C38	2016
2.197225	0.333333	14.09692	2.302585	1	2	2.639057	C38	2017
2.197225	0.333333	14.06354	2.197225	1	2	2.70805	C38	2018
2.197225	0.333333	14.17305	1.791759	1	2	2.772589	C38	2019
2.197225	0.333333	13.54894	2.079442	1	2	2.833213	D45	2018
2.197225	0.333333	13.96935	2.197225	1	2	2.890372	D45	2019
1.791759	0.333333	14.90002	1.791759	1	4	2.833213	C27	2017
1.791759	0.333333	14.93979	1.791759	1	4	2.890372	C27	2018
1.791759	0.333333	14.99047	1.791759	1	4	2.944439	C27	2019
2.079442	0.375	14.36755	2.197225	1	1	2.564949	C15	2018
2.197225	0.333333	14.41528	2.197225	1	1	2.639057	C15	2019
1.94591	0.428571	14.57963	2.484907	1	3	3.044522	G58	2018
1.94591	0.428571	14.89137	2.639057	1	3	3.091042	G58	2019
2.197225	0.333333	13.59798	2.197225	1	4	3.526361	C27	2015
2.197225	0.333333	13.61974	2.302585	1	4	3.555348	C27	2016
2.079442	0.375	13.91155	2.079442	1	4	2.890372	C26	2017
2.079442	0.375	13.99014	1.94591	1	4	2.944439	C26	2018
2.079442	0.375	13.99014	2.197225	1	4	2.995732	C26	2019
2.197225	0.333333	13.4997	1.94591	1	3	2.890372	C29	2016
2.197225	0.333333	14.23711	1.94591	1	3	2.944439	C29	2017
2.197225	0.333333	14.69696	1.791759	1	3	2.995732	C29	2018
2.197225	0.333333	14.4127	1.94591	1	3	3.044522	C29	2019
2.197225	0.333333	14.78606	2.397895	1	2	2.772589	C36	2017
2.197225	0.333333	14.88053	2.484907	1	2	2.833213	C36	2018
2.197225	0.333333	14.88132	2.397895	1	2	2.890372	C36	2019
1.94591	0.428571	14.62127	1.791759	1	2	2.484907	C36	2017
1.94591	0.428571	14.47179	1.098612	1	2	2.564949	C36	2018
1.94591	0.428571	14.47732	1.94591	1	2	2.639057	C36	2019
2.397895	0.363636	14.40773	2.302585	1	4	2.639057	F52	2016
2.397895	0.363636	14.71372	2.302585	1	4	2.70805	F52	2017
2.397895	0.363636	14.9034	2.197225	1	4	2.772589	F52	2018
2.397895	0.363636	14.80394	2.079442	1	4	2.833213	F52	2019

2.197225	0.333333	13.04007	2.302585	1	2	2.197225	C15	2016
2.197225	0.333333	13.28125	2.197225	1	2	2.302585	C15	2017
2.197225	0.333333	13.4508	1.386294	1	2	2.397895	C15	2018
1.94591	0.428571	13.69085	2.079442	1	2	2.772589	C36	2015
1.94591	0.428571	13.73571	2.079442	1	2	2.833213	C36	2016
1.94591	0.428571	13.83913	2.079442	1	2	2.890372	C36	2017
2.197225	0.333333	13.92517	2.397895	1	2	2.944439	C36	2018
2.197225	0.333333	13.92643	2.079442	1	2	2.995732	C36	2019
1.791759	0.5	15.23221	1.791759	1	2	2.397895	C35	2015
2.197225	0.333333	14.4116	2.484907	1	2	2.484907	C35	2016
2.197225	0.333333	14.34949	2.197225	1	2	2.564949	C35	2017
2.197225	0.333333	14.09458	2.197225	1	2	2.639057	C35	2018
2.197225	0.333333	14.27609	1.94591	1	2	2.70805	C35	2019
2.197225	0.333333	14.30163	2.197225	1	4	2.944439	C26	2018
2.197225	0.333333	14.74807	2.397895	1	4	2.995732	C26	2019
1.94591	0.428571	13.24352	2.639057	1	2	2.397895	N77	2017
1.94591	0.428571	13.50094	2.079442	1	2	2.484907	N77	2018
1.94591	0.428571	13.61962	2.484907	1	2	2.564949	N77	2019
2.397895	0.363636	14.69215	2.639057	1	5	2.564949	C32	2015
2.302585	0.4	14.85991	2.484907	1	5	2.639057	C32	2016
1.94591	0.428571	15.45069	2.484907	1	5	2.70805	C32	2017
1.94591	0.428571	15.79271	2.484907	1	5	2.772589	C32	2018
1.94591	0.428571	16.20289	2.397895	1	5	2.833213	C32	2019
2.197225	0.333333	14.93065	2.079442	1	2	2.397895	C36	2017
2.079442	0.375	14.82711	2.484907	1	2	2.484907	C36	2018
2.197225	0.333333	14.82347	1.609438	1	2	2.564949	C36	2019
2.197225	0.333333	15.00262	1.94591	1	2	3.091042	C26	2018
2.197225	0.333333	14.90188	1.609438	1	2	3.135494	C26	2019
2.197225	0.333333	14.89426	2.079442	1	2	2.302585	C21	2016
1.609438	0.4	14.9462	2.484907	1	2	2.397895	C21	2017
1.609438	0.4	14.99281	3.178054	1	2	2.484907	C21	2018
1.609438	0.4	15.17864	2.833213	1	2	2.564949	C21	2019
1.94591	0.428571	13.57979	1.609438	1	2	2.890372	C38	2016
1.94591	0.428571	13.57979	2.197225	1	2	2.944439	C38	2017
1.94591	0.428571	13.56191	2.302585	1	2	2.995732	C38	2018
1.94591	0.428571	13.71547	1.94591	1	2	3.044522	C38	2019
2.197225	0.333333	14.6784	1.098612	1	2	3.044522	C26	2016
2.197225	0.333333	14.68261	2.302585	1	2	3.091042	C26	2017
2.197225	0.333333	14.6827	1.94591	1	2	3.135494	C26	2018
2.197225	0.333333	14.71343	1.609438	1	2	3.178054	C26	2019
1.94591	0.428571	15.62096	2.079442	1	3	3.135494	I64	2017
1.94591	0.428571	15.70983	2.639057	1	3	3.178054	I64	2018
1.94591	0.428571	15.95598	1.791759	1	3	3.218876	I64	2019
1.609438	0.4	15.19694	2.397895	1	4	2.484907	C30	2017
1.609438	0.4	14.85115	1.94591	1	4	2.564949	C30	2018
1.609438	0.4	14.94926	1.791759	1	4	2.639057	C30	2019
2.197225	0.333333	14.37054	2.302585	1	4	2.70805	E50	2015
2.197225	0.333333	14.37054	2.484907	1	3	2.772589	E50	2016
2.197225	0.333333	14.37054	2.302585	1	4	2.833213	E50	2017
2.197225	0.333333	14.37054	2.639057	1	3	2.890372	E50	2018
2.197225	0.333333	14.37054	2.302585	1	4	2.944439	E50	2019
2.197225	0.333333	13.93613	1.94591	1	4	1.94591	C38	2017
2.197225	0.333333	13.91996	2.484907	1	4	2.079442	C38	2018
2.197225	0.333333	14.049	1.609438	1	4	2.197225	C38	2019
1.791759	0.5	13.39392	1.609438	1	1	2.890372	C30	2015
1.791759	0.5	13.9622	2.564949	1	1	2.944439	C30	2016
1.609438	0.6	13.93329	2.397895	1	1	2.995732	C30	2017
1.609438	0.6	14.1789	2.397895	1	1	3.044522	C30	2018

1.94591	0.428571	14.12615	2.484907	1	1	3.091042	C30	2019
1.94591	0.428571	15.54819	2.302585	1	3	2.197225	C18	2017
1.94591	0.428571	15.63099	2.302585	1	3	2.302585	C18	2018
1.94591	0.428571	15.34133	1.609438	1	3	2.397895	C18	2019
2.079442	0.375	14.24917	2.197225	1	3	2.197225	C35	2019
2.197225	0.333333	14.57669	1.94591	1	2	2.197225	C29	2017
2.197225	0.333333	14.75481	2.197225	1	2	2.302585	C29	2018
2.197225	0.333333	14.69381	1.791759	1	2	2.397895	C29	2019
2.70805	0.333333	16.62452	1.791759	1	3	2.70805	C27	2016
2.70805	0.333333	16.23537	1.791759	1	3	2.772589	C27	2017
2.70805	0.333333	15.6063	1.791759	1	3	2.833213	C27	2018
2.70805	0.333333	15.58439	1.791759	1	3	2.890372	C27	2019
2.197225	0.444444	14.34502	2.079442	1	4	3.295837	C38	2016
2.079442	0.5	14.38534	2.484907	1	4	3.332205	C38	2017
2.079442	0.5	14.60174	1.791759	1	4	3.367296	C38	2018
2.079442	0.5	14.87549	2.484907	1	4	3.401197	C38	2019
2.197225	0.444444	14.3096	2.772589	1	2	3.091042	C14	2019
2.302585	0.4	15.10306	2.302585	1	5	3.367296	N78	2015
2.197225	0.333333	15.53626	2.197225	1	5	3.401197	N78	2016
2.197225	0.333333	15.59063	1.791759	1	5	3.433987	N78	2017
2.197225	0.333333	15.66676	2.397895	1	5	3.465736	N78	2018
2.197225	0.333333	15.86926	2.302585	1	5	3.496508	I65	2019
2.197225	0.333333	15.75042	2.564949	1	2	2.70805	C32	2018
2.197225	0.333333	15.73264	2.484907	1	2	2.772589	C32	2019
2.197225	0.333333	13.15522	1.94591	1	3	2.70805	C31	2016
2.197225	0.333333	14.11027	2.197225	1	3	2.772589	C31	2017
1.94591	0.428571	14.11613	2.079442	1	3	2.833213	C31	2018
2.197225	0.333333	14.24781	1.609438	1	3	2.890372	C31	2019
2.197225	0.333333	13.61162	2.079442	1	2	1.791759	C26	2017
2.197225	0.333333	13.77469	2.197225	1	2	1.94591	C26	2018
2.197225	0.333333	13.79316	2.197225	1	2	2.079442	C26	2019
2.197225	0.333333	14.46575	2.079442	0	1	2.639057	C14	2016
2.197225	0.333333	14.50264	2.079442	0	1	2.70805	C14	2017
2.079442	0.375	14.38343	2.079442	0	1	2.772589	C14	2018
2.197225	0.333333	14.60578	2.079442	0	1	2.833213	C14	2019
1.94591	0.428571	14.06487	1.386294	1	3	3.091042	C35	2018
1.94591	0.428571	14.06829	2.302585	1	4	3.135494	C35	2019
2.079442	0.375	14.20944	1.94591	1	3	2.995732	C27	2017
2.197225	0.333333	14.43307	2.302585	1	3	3.044522	C27	2018
2.197225	0.333333	14.53661	2.70805	1	3	3.091042	C27	2019
2.197225	0.333333	14.95584	1.791759	1	4	2.833213	C35	2017
2.197225	0.333333	15.02459	1.94591	1	4	2.890372	C35	2018
2.197225	0.333333	15.02459	1.94591	1	4	2.944439	C35	2019
2.397895	0.363636	14.98861	2.079442	1	3	1.94591	C15	2016
2.397895	0.363636	15.32063	1.386294	1	3	2.079442	C15	2017
2.397895	0.363636	15.18025	1.609438	1	3	2.197225	C15	2018
2.397895	0.363636	15.05617	1.609438	1	3	2.302585	C15	2019
1.94591	0.428571	14.20525	1.791759	1	2	2.079442	C36	2017
2.079442	0.375	13.96376	1.94591	1	2	2.197225	C36	2018
1.94591	0.428571	13.94601	2.397895	1	2	2.302585	C36	2019
1.94591	0.428571	14.67056	1.791759	1	2	2.639057	E49	2016
2.079442	0.375	14.48129	1.791759	1	2	2.70805	E49	2017
1.94591	0.428571	14.45156	1.791759	1	2	2.772589	E49	2018
2.197225	0.333333	14.49805	1.791759	1	2	2.833213	E49	2019
2.197225	0.333333	14.11953	2.397895	1	1	3.044522	C27	2017
2.197225	0.333333	14.21335	1.386294	1	1	3.091042	C27	2018
2.197225	0.333333	14.19072	1.609438	1	1	3.135494	C27	2019
2.197225	0.333333	14.55759	1.791759	1	3	2.639057	C26	2015

2.197225	0.333333	14.88022	2.079442	1	3	2.70805	C26	2016
2.302585	0.4	14.91078	1.94591	1	3	2.772589	C26	2017
2.197225	0.333333	15.09311	2.079442	1	3	2.833213	C26	2018
2.197225	0.333333	15.07624	1.94591	1	3	2.890372	C26	2019
2.197225	0.333333	13.09561	2.079442	1	1	2.833213	C33	2015
2.197225	0.333333	13.00987	2.564949	1	1	2.890372	C33	2016
2.197225	0.333333	13.24032	1.609438	1	1	2.944439	C33	2017
2.197225	0.333333	13.01098	1.791759	1	1	2.995732	C33	2018
2.197225	0.333333	13.45669	1.609438	1	1	3.044522	C33	2019
2.197225	0.333333	13.89764	1.94591	1	4	3.332205	C27	2017
2.197225	0.333333	14.097	1.386294	1	4	3.367296	C27	2018
2.197225	0.333333	14.01649	1.791759	1	4	3.401197	C27	2019
2.197225	0.333333	15.03028	2.079442	1	2	2.639057	C26	2017
2.079442	0.375	14.90022	2.197225	1	2	2.70805	C26	2018
2.079442	0.375	14.62893	1.098612	1	2	2.772589	C26	2019
2.397895	0.363636	14.99352	2.197225	1	5	2.890372	C35	2016
2.197225	0.333333	14.56872	2.639057	1	5	2.944439	C35	2017
2.197225	0.333333	15.15385	2.833213	1	5	2.995732	C35	2018
2.197225	0.333333	14.90094	2.302585	1	5	3.044522	C35	2019
1.94591	0.428571	14.07841	1.94591	1	2	2.70805	C38	2018
1.94591	0.428571	13.91082	2.484907	1	1	3.091042	C39	2015
1.791759	0.5	14.05058	1.386294	1	1	3.135494	C39	2016
1.94591	0.428571	14.00613	2.197225	1	1	3.178054	C39	2017
1.94591	0.428571	13.96823	2.397895	1	1	3.218876	C39	2018
1.94591	0.428571	14.01436	1.609438	1	1	3.258097	C39	2019
2.197225	0.333333	14.13353	1.791759	1	3	2.772589	C31	2019
2.197225	0.333333	14.61675	2.70805	1	5	2.772589	C36	2019
1.94591	0.428571	15.28927	2.079442	1	2	2.079442	C35	2019
1.94591	0.428571	15.14144	2.302585	1	1	2.70805	C40	2019
1.94591	0.428571	14.18168	2.397895	1	3	2.484907	C39	2019
2.197225	0.333333	14.88935	2.639057	1	4	2.564949	C39	2019
2.197225	0.333333	15.39916	2.70805	1	5	2.302585	C37	2019
2.197225	0.333333	15.00943	2.079442	1	3	2.890372	C27	2019
2.197225	0.333333	14.34006	2.079442	1	4	2.70805	M73	2019
1.94591	0.428571	14.90565	2.397895	1	3	2.995732	C26	2019
1.94591	0.428571	14.7895	2.484907	1	2	2.833213	C30	2019
2.197225	0.333333	13.28073	2.639057	1	4	3.044522	C26	2019
1.609438	0.4	15.01182	2.079442	1	3	3.135494	C35	2019
2.197225	0.333333	14.81515	2.484907	1	3	2.890372	C39	2019
2.197225	0.333333	13.52342	2.079442	1	4	2.890372	C27	2014
2.197225	0.333333	14.87784	1.94591	1	2	2.639057	C35	2016
2.397895	0.363636	13.90763	2.079442	1	2	2.639057	C34	2014
2.197225	0.333333	14.53335	1.609438	1	2	1.791759	C33	2016
1.94591	0.428571	13.59237	1.386294	1	3	2.564949	C35	2016
2.197225	0.333333	14.19737	2.079442	1	5	2.397895	C13	2016
2.197225	0.333333	14.6718	1.94591	1	2	2.995732	C38	2016
2.197225	0.333333	14.26938	1.386294	1	4	1.94591	C39	2014
2.197225	0.333333	14.08079	2.564949	1	2	2.484907	C38	2015
2.397895	0.363636	14.98452	2.70805	1	5	2.484907	C32	2014
1.94591	0.428571	14.94433	1.94591	1	3	2.079442	C18	2016
2.197225	0.333333	12.78885	2.079442	1	1	2.772589	C33	2014
1.94591	0.428571	14.37564	1.609438	1	4	2.890372	C29	2019
2.197225	0.333333	14.05011	1.94591	1	3	2.70805	C39	2019
2.079442	0.5	14.82817	1.791759	1	3	2.564949	C33	2019
1.94591	0.428571	13.68164	2.302585	1	3	2.484907	C30	2019

Sonnum	Girlnum	Ownership	Roe	Inst	Family year
0	2	15.4757	0.005614	2.09491	2.197225
1	0	56.1129	0.036318	6.623	3.091042
1	0	57.77	0.011522	5.77374	3.135494
1	0	31.67	0.052909	4.21495	3.178054
1	0	40.7286	0.00549	5.261	1.098612
1	0	40.7286	0.012643	5.211	1.386294
1	0	40.7286	0.015522	5.21836	1.609438
1	0	40.7286	0.004756	5.163	1.791759
1	0	40.7286	-0.00891	5.2085	1.94591
1	0	40.7286	0.006612	5.19	2.079442
1	0	40.7286	-0.00364	5.34962	2.197225
1	0	40.7286	0.008629	5.34001	2.302585
1	0	20.9814	0.001547	5.401	2.397895
1	0	41.21	0.00534	5.48	2.484907
1	0	42.13	0.00889	5.56878	2.564949
0	1	47.4143	0.017167	8.63146	1.791759
0	1	61.4629	0.005347	8.4562	1.94591
0	1	58.9568	0.010474	8.46506	2.079442
0	1	58.9658	0.025151	8.60467	2.197225
0	1	58.9658	0.028942	8.43419	2.302585
0	1	58.9581	0.023477	8.35079	2.397895
0	1	59.083	0.022474	8.54066	2.484907
0	1	53.2412	0.018214	8.53925	2.564949
0	1	53.6437	0.01647	8.11612	2.639057
0	1	55.0268	0.004871	8.1721	2.70805
0	1	55.0268	0.00802	7.94923	2.772589
0	2	24.1	0.024975	3.428	2.302585
0	2	26.36	-0.00747	2.809	2.397895
0	2	26.36	0.060601	3.02062	2.484907
0	2	28.54	0.108088	4.242	2.564949
0	2	29.85	0.003064	6.93696	2.639057
0	2	29.85	-0.03554	6.83621	2.70805
1	2	34.687	0.085589	6.92985	0
1	2	34.687	0.096817	6.168	0.693147
1	2	34.2801	0.105747	5.98436	1.098612
1	2	34.0561	0.092977	5.12664	1.386294
1	2	32.5628	0.075013	5.87026	1.609438
1	2	32.0706	0.082102	6.19255	1.791759
1	2	30.6507	0.083712	6.29355	1.94591
1	2	17.56	0.024321	3.74536	0
0	2	19.69	0.028455	2.323	2.302585
1	1	14.1801	0.079211	2.61876	0
1	1	16.8285	0.02966	2.60441	0.693147
1	1	16.8285	0.058487	2.57436	1.098612
1	1	16.8285	0.078558	2.571	1.386294
1	1	17.4028	0.022552	2.748	1.609438
1	1	17.4028	0.106606	2.69909	1.791759
1	2	10.2884	0.057158	6.24977	0.693147
1	2	14.6419	0.076202	7.37242	1.098612
1	2	10.7179	0.058891	6.91058	1.386294
1	2	10.0401	0.04508	7.09797	1.609438
1	0	14.2244	0.051478	7.08129	1.791759
1	0	18.6943	0.069512	6.92136	1.94591
1	2	17.4647	0.073503	7.30924	2.079442
1	2	17.3053	0.071099	7.5128	2.197225
1	2	16.7874	0.080063	7.81454	2.302585
1	2	16.5334	0.090442	7.79525	2.397895

1	2	29.718	0.059165	5.06383	0
1	2	29.718	0.031829	5.33099	0.693147
1	2	27.4591	0.011735	6.01193	1.098612
1	2	30.51	0.040158	6.10151	1.386294
1	2	30.51	-0.0071	6.31033	1.609438
1	2	32.11	0.009137	5.58096	1.791759
1	2	29.76	0.059322	5.53154	1.94591
1	2	29.76	-0.0191	5.26524	2.079442
1	2	30.27	0.011026	5.12185	2.197225
1	0	18.1427	0.061405	2.722	1.386294
1	0	20.6398	0.025901	2.5626	2.079442
1	0	20.6398	0.013899	3.97503	2.197225
1	0	20.6398	-0.05981	3.48091	2.302585
1	0	22.65	0.183224	3.41099	2.564949
1	0	22.65	-0.0399	3.32747	2.639057
2	0	13.7821	0.003877	2.87331	1.94591
2	0	12.6842	0.006962	2.80853	2.079442
1	0	8.5408	0.035801	2.43276	1.609438
1	0	8.5408	0.010793	2.18182	1.791759
1	0	9.2294	-0.00404	2.4454	1.94591
1	0	9.2714	0.018788	2.64832	2.079442
1	0	6.1431	0.013759	2.36276	2.197225
2	0	28.8277	0.164447	7.0651	2.833213
2	0	28.2042	0.123564	7.65599	2.890372
2	0	28.2208	0.084251	6.5153	2.944439
2	0	29.2331	0.084353	7.47868	2.995732
2	0	29.2334	0.079803	6.9823	3.044522
2	0	29.2334	0.075812	8.5264	3.091042
1	0	18.0288	0.032674	3.70137	1.791759
1	0	18.0209	0.056245	3.6089	1.94591
1	0	18.0209	0.040461	5.1854	2.079442
1	0	18.0209	0.030509	3.88969	2.197225
1	0	18.0209	0.033648	4.16055	2.302585
1	0	18.0209	0.099697	4.06716	2.397895
1	0	16.3104	0.045356	3.77683	2.484907
1	0	16.4293	0.036235	3.14909	2.564949
1	0	16.4293	0.033738	3.02434	2.639057
1	0	19.1893	-0.04113	3.16218	2.772589
2	0	12.3723	0.003779	4.051	0
2	0	8.982738	0.036178	2.973	0
1	2	34.71105	0.079408	8.42238	2.833213
1	2	37.4666	0.096144	7.63473	2.890372
1	2	27.1033	0.076254	7.28485	2.944439
1	2	25.0402	0.067798	6.84482	2.995732
1	0	11.1156	0.047887	4.133	2.995732
1	0	11.1156	0.041	4.13777	3.044522
1	0	16.3252	0.041274	4.23644	3.091042
0	1	30.668	0.072999	6.31765	1.386294
0	1	30.668	0.045693	5.80435	1.609438
0	1	30.668	0.029409	5.59553	1.791759
0	1	31.0644	0.016925	5.58131	1.94591
0	1	30.855	0.029677	5.17523	2.079442
0	1	29.6432	0.036924	6.23238	2.197225
0	1	31.2537	0.04719	5.44883	2.302585
0	1	33.5852	0.041496	5.74794	2.397895
0	1	33.5777	0.019756	5.63922	2.484907
0	1	33.7722	0.014207	5.66802	2.564949
0	1	34.5352	0.009606	5.49072	2.639057

1	0	14.7422	-0.06557	3.01	1.791759
1	1	6.6886	0.012788	2.55014	0
1	1	12.4215	0.071563	2.59753	0.693147
1	1	44.5716	0.059904	6.37274	0
1	1	40.6979	0.066355	5.81676	0.693147
1	1	40.61	0.064026	6.479	1.098612
1	1	37.9787	0.050302	6.40526	1.386294
1	1	37.9788	0.052907	6.29084	1.609438
1	1	32.9688	0.063265	4.86544	1.791759
0	1	65.93	0.055504	7.05175	2.772589
0	1	65.93	0.044057	6.933	2.833213
0	1	65.93	0.011226	7.36584	2.890372
0	1	65.94	0.008478	5.286	2.944439
0	1	65.94	0.009705	5.31457	2.995732
0	1	59.33	0.027255	6.59223	3.044522
0	1	53.86	0.021603	5.55681	3.135494
0	1	53.86	-0.01971	4.78796	3.218876
1	0	48.8384	0.051788	6.84094	1.609438
1	0	41.4795	0.058474	6.99507	1.791759
1	0	41.224	0.057858	6.65191	1.94591
1	0	41.224	0.044579	5.98844	2.079442
1	0	41.224	0.061956	5.73316	2.197225
1	0	41.224	0.065718	5.62907	2.302585
1	0	41.224	0.065783	6.09885	2.397895
1	0	41.484	0.077317	5.99488	2.484907
1	0	45.2	0.080103	6.64443	2.564949
1	0	56.51	0.062125	6.64573	2.639057
1	0	56.51	0.045383	6.71724	2.70805
0	1	6.7098	0.048213	1.98339	1.609438
0	1	7.288	0.021594	2.26805	1.791759
0	1	7.368	0.007192	2.379	1.94591
3	0	8.2482	0.009282	2.065	1.386294
3	0	8.2482	0.018522	1.9637	1.609438
3	0	9.7468	0.024238	2.01586	1.791759
3	0	9.7468	0.024134	2.078	1.94591
3	0	9.7468	0.037072	1.972	2.079442
3	0	11.3988	0.001806	2.12021	2.197225
3	0	11.3988	-0.03102	1.8785	2.302585
3	0	11.3988	0.069721	2.01374	2.397895
3	0	11.4195	0.01076	2.16789	2.484907
1	1	20.9828	0.024348	4.697	1.098612
1	1	20.9828	0.006871	4.604	1.386294
1	1	22.8928	0.09055	4.67552	1.609438
1	1	22.8928	0.06495	4.9403	1.791759
1	1	23.9086	0.064595	4.83165	2.079442
1	1	23.9086	0.068172	4.74565	2.197225
1	0	27.8375	0.011719	5.45087	1.791759
1	0	27.8435	0.046545	3.91984	1.94591
1	0	11.466	0.003181	3.9319	2.079442
1	0	11.466	0.008734	4.40529	2.197225
1	0	11.466	0.115232	3.39378	2.302585
1	0	11.6165	0.046816	3.46158	2.397895
1	0	11.7285	0.004593	3.46518	2.484907
1	1	12.0279	0.092251	5.47472	0.693147
1	0	20.0679	-0.11331	4.11533	1.098612
1	0	16.055	0.019681	3.122	1.386294
1	0	26.9102	-0.07033	5.443	1.791759
1	0	26.1982	-0.05312	5.31703	2.079442

1	0	29.0007	0.116654	5.01476	2.197225
1	0	29.0007	0.109511	5.09696	2.302585
1	0	30.3784	0.150308	5.21209	2.397895
1	0	27.5784	0.128803	4.77581	2.484907
1	0	9.9607	-0.03013	2.88276	1.791759
1	0	12.601	-0.11197	2.75741	1.94591
1	0	12.896	0.024427	2.4293	2.197225
1	0	12.896	0.018093	2.40783	2.302585
0	2	21.83	0.02565	2.47777	1.94591
0	2	21.83	0.014717	2.60058	2.079442
0	2	21.83	-0.0569	2.6129	2.197225
0	2	21.83	0.006686	2.57712	2.302585
0	2	21.83	-0.04657	2.47533	2.397895
0	2	23.2069	0.039894	2.49172	2.484907
0	2	20.3871	0.103992	2.51329	2.564949
1	0	58.92	0.039641	8.28896	0.693147
1	0	58.92	0.044466	7.98775	1.098612
1	0	58.92	0.049029	7.97263	1.386294
1	0	58.92	0.052735	8.03109	1.609438
1	0	58.92	0.047337	8.01499	1.791759
1	0	55.096	0.048891	7.44593	1.94591
1	0	55.096	0.027488	7.99616	2.079442
1	0	49.064	0.021704	8.09275	2.197225
1	0	49.064	0.055338	7.86764	2.302585
1	0	10.5225	-0.01683	2.936	0
1	0	33.5187	0.093073	7.138	0.693147
1	0	33.5187	0.080941	7.78199	1.098612
1	0	33.5187	0.099987	6.40344	1.386294
1	0	33.9822	0.125978	6.32417	1.609438
1	0	33.9822	0.063182	6.21912	1.791759
1	0	34.5529	0.083949	6.31441	1.94591
1	0	41.8496	0.015438	7.56606	2.079442
1	0	42.5273	0.051379	7.53947	2.197225
1	0	35.1005	0.051762	6.99701	2.302585
1	0	35.0734	0.156026	8.27487	1.098612
1	0	33.2897	0.152685	8.02078	1.386294
1	0	32.5632	0.131817	6.50776	1.791759
1	0	32.5632	0.092756	5.2727	1.94591
1	0	22.6479	0.124698	2.71127	2.079442
1	0	19.206	0.138007	2.92089	2.197225
1	0	19.206	0.111463	3.10166	2.302585
1	0	19.206	0.105858	2.81008	2.397895
1	0	19.206	0.126517	4.11921	2.484907
1	0	19.206	0.124247	2.68625	2.564949
0	2	48.27	0.028444	5.453	1.098612
0	2	48.27	0.023993	5.33069	1.386294
0	2	48.27	0.015166	4.51992	1.609438
0	2	40.57	0.010516	3.08287	1.386294
0	2	30.35	0.012911	2.74667	1.609438
0	2	26.24	0.016388	4.30573	1.791759
0	2	26.58	0.014523	4.76613	1.94591
0	2	29.98	0.017428	5.02529	2.079442
0	2	29.98	0.019766	5.28254	2.197225
0	2	6.2323	0.032897	5.87206	1.098612
0	2	6.2323	0.020798	4.07589	1.386294
0	2	6.2323	0.015215	4.41486	1.609438
0	2	6.1843	0.012542	4.243	1.791759
0	2	6.2323	0.003381	3.73518	1.94591

0	2	9.348	0.004701	3.6321	2.079442
0	2	9.348	0.024338	2.81669	2.197225
0	2	10.008	0.036803	1.96852	2.302585
0	2	9.348	0.03751	1.95211	2.397895
0	2	10.008	0.006455	2.13139	2.484907
0	2	10.382	-0.00755	2.08738	2.564949
1	1	39.28	0.067963	5.078	1.098612
1	1	39.28	0.004362	4.8882	1.386294
1	1	39.28	0.004151	4.53	1.609438
1	1	39.28	0.003495	4.827	1.791759
1	1	38.4944	0.020214	4.42417	2.079442
1	1	38.4944	0.003906	4.8198	2.397895
1	1	39.7194	0.015547	4.06904	2.484907
1	0	14.1284	0.009154	3.59917	1.098612
1	0	14.1284	0.007338	3.46115	1.386294
1	1	14.9293	0.00425	3.03418	0
1	1	16.4472	0.000331	2.99965	0.693147
1	1	13.6986	-0.07729	3.00199	1.098612
1	1	14.9293	-0.07663	2.993	1.386294
1	0	57.92	0.093863	1.24788	0
1	0	55.4	0.061748	1.27439	0.693147
1	0	55.4	0.065433	0.88958	1.098612
1	0	55.4	0.076246	1.40862	1.386294
1	0	55.4	0.079255	2.38733	1.609438
1	0	45.18	0.052818	1.55348	0
0	2	40.99	0.149832	7.68952	1.94591
0	2	40.99	0.11892	7.77808	2.079442
0	2	40.99	0.17269	7.76761	2.197225
1	0	32.478	0.022831	4.06813	2.397895
1	0	30.5675	0.015998	3.646	2.484907
1	0	30.5675	0.025595	4.15	2.564949
1	0	28.2661	0.045551	3.94623	2.639057
1	0	29.7723	0.0562	3.77638	2.70805
1	0	29.7723	0.063341	4.17562	2.772589
1	0	27.9264	0.065034	5.6849	2.833213
1	0	27.3224	0.031372	4.06701	2.890372
1	0	27.5523	0.017851	3.49595	2.944439
1	0	35.4205	0.001377	4.31101	2.995732
1	1	35.4205	0.012958	4.28771	3.044522
1	0	55.5339	0.112039	8.20901	0
1	0	55.5339	0.021354	8.25558	0.693147
1	0	55.5339	0.021092	8.03224	1.098612
1	0	54.6445	-0.01483	7.39228	1.386294
1	0	57.7045	0.006432	7.88591	1.609438
1	0	44.2647	0.032274	8.09499	1.791759
1	0	39.0226	0.05325	7.1131	1.94591
1	0	37.7295	0.037626	7.51632	2.079442
1	0	37.7295	0.047174	7.22096	2.197225
1	0	22.5066	-0.00231	3.1526	2.079442
1	0	22.5066	0.009048	3.0931	2.197225
1	0	22.5066	0.020335	3.952	2.302585
1	0	22.5066	0.000119	2.964	2.397895
1	0	22.5066	-0.02687	3.1933	2.484907
1	0	60.91	0.087912	4.19139	1.609438
1	0	63.6	0.057797	4.15066	1.791759
1	0	63.6	0.046948	4.26583	1.94591
1	0	53.81	0.044866	4.37043	2.079442
1	0	63.6	0.031606	3.52074	2.197225

1	0	63.6	0.035675	3.35319	2.302585
1	0	53.95	0.035602	4.07766	2.397895
1	0	53.95	0.050056	3.96242	2.484907
1	0	53.95	0.054265	3.19631	2.564949
1	0	53.95	0.051823	3.16047	2.639057
1	0	55.66	0.067226	3.18677	2.70805
1	1	29.73	-0.00434	4.77169	1.791759
1	1	29.73	0.010216	4.458	1.94591
1	1	29.73	0.022727	4.4226	2.079442
1	1	29.73	-0.04254	4.2719	2.197225
1	1	29.73	0.04644	4.33527	2.302585
1	1	29.7278	0.011944	4.383	2.397895
1	1	57.38	0.055098	7.47685	2.772589
0	1	6.6722	0.044892	6.27896	1.609438
0	1	31.0071	0.054897	6.38008	1.791759
1	0	8.2995	0.002309	2.881	0
1	0	8.2995	0.021708	4.18862	0.693147
1	0	8.3083	0.002681	3.92895	1.098612
1	0	16.2809	-0.05704	3.63501	1.386294
1	1	11.81	0.051024	1.217	1.791759
1	1	11.81	0.027507	1.181	1.94591
1	1	11.81	0.005118	1.18368	2.079442
1	1	11.81	-0.0535	1.294	2.197225
1	1	11.81	0.010841	1.396	2.302585
1	0	8.54	0.013233	1.27541	0
1	0	10.93	0.012254	3.36345	0.693147
1	0	17.01	-0.05918	2.5599	1.098612
1	0	18.18	0.094105	2.82624	1.386294
0	1	27.2736	0.026675	3.38981	1.386294
0	1	27.2736	0.003815	3.426	1.609438
0	1	27.2736	-0.0279	3.432	1.791759
0	1	21.7845	0.025329	2.756	1.94591
0	1	20.8606	0.007366	2.66147	2.079442
0	1	20.8606	-0.08172	2.836	2.197225
0	1	17.0743	0.039784	4.3242	2.302585
1	0	22.2336	0.029333	5.283	0
1	0	22.2336	0.027224	5.59392	0.693147
1	0	44.3557	0.014849	6.73265	1.098612
1	0	44.3557	0.019396	5.98539	1.386294
1	0	44.3557	0.024637	5.98413	1.609438
1	0	45.8281	0.004143	6.1816	1.791759
1	0	45.8281	0.004777	6.18844	1.94591
1	0	42.55	0.126607	7.39829	1.386294
1	0	42.55	0.130552	7.1769	1.609438
1	0	42.55	0.129904	7.22609	1.791759
1	0	42.55	0.147124	7.29499	1.94591
1	0	40.68	0.147182	6.52341	2.397895
1	0	40.68	0.148716	6.01219	2.484907
1	0	40.68	0.145152	6.47331	2.564949
1	0	40.68	0.150008	6.10566	2.639057
0	1	28.4894	0.062777	6.32677	2.484907
0	1	21.035	0.154194	8.73089	2.639057
0	1	21.035	0.08883	7.96447	2.70805
0	1	21.035	0.084356	8.02956	2.772589
0	1	24.5138	0.078243	7.67565	2.833213
0	1	24.5321	0.084041	7.33458	2.890372
0	1	24.5321	0.083789	7.07115	2.944439
0	1	24.3087	0.069072	7.0178	2.995732

0	1	28.7744	0.056771	7.06116	3.044522
0	1	28.9672	0.096244	7.07417	3.091042
1	1	31.8248	0.129326	8.23988	1.386294
1	1	30.5619	0.144912	8.21476	1.609438
1	1	30.5619	0.109515	8.64704	1.791759
1	1	30.9964	0.140442	7.70151	1.94591
1	1	30.7285	0.097465	6.24061	2.079442
1	1	27.3268	0.1083	5.4004	2.197225
1	1	24.2596	0.100377	5.3546	2.302585
1	1	24.1637	0.078278	5.75456	2.397895
1	1	18.6563	0.075158	5.50217	2.484907
1	1	25.1802	0.070792	5.80764	2.564949
1	1	25.1802	0.032654	5.84065	2.639057
1	1	16.5007	0.06564	5.37498	1.386294
0	1	19.83	0.134506	3.03095	0
0	1	19.49	0.107532	2.4832	0.693147
0	1	19.4	0.075595	2.46961	1.098612
0	1	19.8	0.038042	2.08418	1.386294
0	1	19.8	0.037982	2.19492	1.609438
2	1	15.31	0.035231	6.25079	1.791759
2	1	15.3099	0.037965	5.91934	1.94591
2	1	15.3099	0.023776	5.8901	2.079442
2	1	15.3099	0.027671	5.63915	2.197225
2	1	15.3099	0.029747	5.96342	2.302585
2	1	15.3099	0.033455	5.95336	2.397895
2	1	26.5265	0.060308	6.94714	2.484907
2	1	26.4153	0.09005	7.41246	2.564949
2	1	25.248	0.116282	7.11237	2.639057
2	1	36.7578	0.110249	7.02385	2.70805
2	1	36.7738	0.092085	6.31348	2.772589
1	1	29.77	0.020035	5.5625	0.693147
1	1	29.77	0.008427	5.04585	1.098612
1	1	29.77	0.03642	5.07103	1.386294
0	2	46.0225	0.033085	8.88717	0
0	2	52.73	0.032063	8.64443	0.693147
0	2	34.2816	0.029591	8.76494	1.098612
0	2	34.2816	0.022406	9.07142	1.386294
0	2	34.2816	0.021745	8.13291	1.609438
0	1	34.28161	0.01111	8.50616	1.791759
0	1	32.0632	0.004588	7.91299	1.94591
0	1	25.61091	0.003065	7.4892	2.079442
0	1	25.61091	0.003072	7.71938	2.197225
0	1	25.7402	0.009812	8.04852	2.302585
0	1	25.4186	0.015909	8.17057	2.397895
0	1	17.395	0.083176	8.80677	0
0	1	17.395	0.094905	9.02307	0.693147
0	1	18.105	0.097435	8.45272	1.098612
0	1	18.105	0.104667	8.09651	1.386294
0	1	18.105	0.100915	7.25749	1.609438
0	1	21.3027	0.10621	7.47359	1.791759
0	1	38.8461	0.118109	7.66652	1.94591
0	1	38.8461	0.124636	7.70098	2.079442
0	1	38.8461	0.136294	6.99175	2.197225
0	1	18.872	0.006315	6.73126	0.693147
0	1	18.872	0.099592	6.91771	1.098612
0	1	18.872	0.026232	6.64485	1.386294
0	1	16.896	0.047726	5.62543	1.609438
1	0	66.1234	0.017607	6.681	1.609438

1	0	66.1163	0.028366	3.87217	1.791759
1	0	48.19	0.022909	5.08617	1.098612
1	0	48.19	0.033511	5.43641	1.386294
1	0	33.17	0.045697	4.52385	1.609438
1	0	33.35	0.055291	4.72896	1.791759
1	0	33.35	0.032931	4.80124	1.94591
0	1	42.33	0.105356	3.94468	0
0	1	42.33	0.135887	3.84964	0.693147
0	1	42.33	0.121251	3.32845	1.098612
0	1	42.33	0.063376	2.67962	1.386294
0	1	42.33	0.038063	2.355	1.609438
0	1	13.524	0.158401	6.26567	2.564949
0	1	13.524	0.10744	5.18292	2.639057
0	1	15.894	0.092913	4.54078	2.70805
0	1	13.68	0.07489	4.65784	2.772589
0	1	20.4332	0.065238	4.26757	2.833213
0	1	20.4332	0.073377	4.72003	2.890372
0	1	20.4332	0.06823	4.88034	2.944439
0	1	21.2548	0.056479	4.3244	2.995732
0	1	21.4048	0.048576	4.05027	3.044522
0	1	21.132	0.050296	4.15535	3.091042
0	1	21.1079	0.055158	3.99898	3.135494
1	0	24.6675	0.113077	3.72254	1.791759
1	0	24.6675	0.099518	4.84218	1.94591
1	0	19.4412	0.073623	3.62997	2.079442
1	0	19.4827	0.062613	3.15477	2.197225
1	0	16.8544	0.041449	2.85641	2.302585
1	0	16.9145	0.032871	2.33571	2.397895
1	0	14.0387	0.033324	1.887	2.484907
1	0	12.1187	0.025247	2.09231	2.564949
2	1	17.3213	0.020408	2.24817	2.639057
2	1	15.3915	0.030989	2.06739	2.70805
2	1	15.8262	0.034986	2.23165	2.772589
1	0	25.95	0.03213	4.29301	1.791759
1	0	28.95	0.037761	5.06454	1.94591
1	0	28.95	0.056848	5.69377	2.079442
1	0	23.99	0.017286	5.72947	2.197225
1	0	23.99	0.000424	4.42294	2.302585
1	0	27.19	0.001033	2.99279	2.397895
1	0	25.04	0.001052	2.70193	2.484907
1	0	23.4	0.002057	2.36213	2.564949
1	0	18.52	-0.06925	4.22682	2.639057
1	0	9.064	0.005238	3.5101	1.791759
1	0	9.064	0.056303	4.26944	1.94591
1	0	8.6154	0.146103	4.36118	2.079442
1	0	8.6154	-0.09177	3.67764	2.197225
1	0	8.8966	0.008971	3.79741	2.484907
1	0	9.0783	0.035499	3.49624	2.564949
1	0	9.4804	0.052819	3.18312	2.639057
1	0	9.7947	0.003478	3.44718	2.70805
1	0	9.7947	-0.07132	3.32883	2.772589
0	1	34.7443	0.140964	7.29147	2.079442
0	1	34.7443	0.105996	6.74107	2.197225
0	1	34.4589	0.13053	6.71484	2.302585
0	1	34.6622	0.142884	6.41817	2.397895
0	1	35.9904	0.144722	4.42866	2.484907
0	1	45.3492	0.165868	4.72077	2.564949
0	1	45.4492	0.153985	4.52267	2.639057

0	1	45.4692	0.186282	5.30353	2.70805
0	1	45.214	0.181833	5.36326	2.772589
1	0	21.11	0.003851	1.67025	1.791759
1	0	21.11	0.031338	0.78513	1.94591
1	0	21.11	0.013622	0.73147	2.079442
1	0	21.11	0.010971	0.35743	2.197225
1	0	17.65	0.009267	2.93369	2.302585
1	0	17.65	0.023919	4.31659	2.397895
1	0	15.53	0.026219	2.92259	2.484907
1	0	24.449	0.020554	2.32321	2.564949
1	0	25.508	0.017577	2.79468	2.639057
1	0	25.858	0.023339	1.84925	2.70805
1	0	25.872	0.010032	1.43711	2.772589
2	0	35.3302	0.130505	3.56019	1.791759
2	0	42.4	0.090881	4.07345	1.94591
2	0	43.15	0.066904	3.93098	2.079442
2	0	43.49	0.056386	3.72715	2.197225
2	0	43.49	0.063714	3.33081	2.302585
2	0	43.49	0.065768	3.56736	2.397895
2	0	61.6801	0.042701	8.14968	2.484907
2	0	63.63	0.044621	8.17568	2.564949
2	0	63.5332	0.022413	8.18596	2.639057
2	0	64.91	0.030031	8.07809	2.70805
2	0	64.91	0.053735	7.47336	2.772589
1	1	21.51	0.009212	2.90176	0
1	1	31.4378	0.034734	7.6907	0.693147
1	0	67.3982	0.001537	0.21837	2.079442
0	2	22.82	0.002994	1.23376	1.791759
0	2	15.51	-0.08779	1.15972	1.94591
1	0	36.542	0.026629	3.59461	1.791759
1	0	30.294	0.024137	5.05145	2.302585
0	1	32.28	-0.03973	1.989	1.791759
0	1	30.6	0.025076	1.989	1.94591
0	1	30.6	0.029694	2.32622	2.079442
0	1	30.6	0.007819	2.04898	2.197225
0	1	30.6	0.014351	2.21397	2.302585
0	1	40.1374	0.002426	3.77688	2.397895
2	0	34.87	0.061555	1.77092	1.791759
2	0	31.12	0.043984	1.74709	1.94591
2	0	27.76	0.037785	2.35356	2.079442
2	0	27.76	0.044511	1.68336	2.197225
2	0	24.32	0.050782	3.63665	2.302585
2	0	24.05	0.00711	0.74225	2.70805
0	1	17.3077	0.047024	4.96411	1.791759
0	1	18.3717	0.059972	4.735	1.94591
0	1	18.3717	0.080142	4.74769	2.079442
0	1	18.8658	0.07443	4.95957	2.197225
0	1	18.8658	0.044116	4.84416	2.302585
0	1	12.1555	0.049883	6.11721	2.397895
0	1	12.1555	0.039618	6.42651	2.484907
0	1	13.6105	0.037436	5.79338	2.564949
0	1	15.1741	0.04198	4.81616	2.639057
0	1	32	0.050204	4.09357	2.70805
0	1	34	-0.04192	4.17745	2.772589
1	0	23.24	0.099271	5.73618	1.609438
2	0	38.6745	0.104207	8.77818	1.791759
2	0	38.6745	0.118684	8.60335	1.94591
2	0	38.6745	0.129866	9.04476	2.079442

2	0	24.003	0.102154	8.37688	2.197225
2	0	33.2955	0.055124	5.58809	2.302585
2	0	33.2955	0.042567	5.01407	2.397895
2	0	33.7586	0.03786	5.08805	2.484907
1	0	34.29	0.035053	5.65381	2.564949
1	0	34.29	0.039991	5.59784	2.639057
1	0	36.28	0.042427	5.93613	2.70805
1	0	37.92	0.038285	4.92891	2.772589
1	0	6.5828	0.080605	4.49721	0
2	0	46.856	0.034814	0.71644	1.791759
2	0	46.856	0.014129	1.284	1.94591
2	0	45.826	0.097708	0.72694	2.079442
2	0	46.204	0.011634	0.87075	2.197225
2	0	43.9024	0.02081	0.80925	2.302585
2	0	43.3051	-0.03597	1.15134	2.397895
2	0	43.9254	0.0519	1.69754	2.484907
2	0	50.8543	0.021076	1.59674	2.564949
2	0	51.2726	0.054824	5.17014	2.639057
2	0	51.2726	0.050218	5.20841	2.70805
2	0	51.2726	0.045953	5.20011	2.772589
1	0	51.2232	0.117829	7.03087	1.609438
1	0	51.2232	0.196927	7.46042	1.791759
1	0	51.3228	0.18043	6.88625	1.94591
1	0	51.3477	0.144046	6.38464	2.079442
1	0	51.3477	0.161656	7.27922	2.197225
1	0	51.3477	0.135593	6.57202	2.302585
1	0	51.4441	0.114356	6.24393	2.397895
1	0	51.4641	0.116978	6.88484	2.484907
1	0	51.4724	0.030685	6.6352	2.564949
1	0	51.489	-0.00657	6.31743	2.639057
1	0	51.489	-0.01091	6.14829	2.70805
0	1	7.2628	0.062727	5.77946	1.609438
0	1	8.1153	0.04217	5.44388	1.791759
0	1	8.1153	0.030547	4.82694	1.94591
0	1	8.6153	-0.07714	3.67644	2.079442
0	1	8.7653	0.028026	3.99592	2.197225
0	1	8.7653	0.035335	3.58291	2.302585
0	1	8.8988	0.015248	4.4813	2.397895
0	1	8.8988	0.018408	4.79585	2.484907
0	1	9.3539	0.030725	4.28398	2.564949
0	1	8.3152	-0.0459	3.31703	2.639057
0	1	8.3152	0.082515	3.66861	2.70805
1	0	28.8507	0.071617	3.724	1.609438
2	0	37.0302	0.117607	8.27808	1.609438
2	0	33.0792	0.084831	8.05652	1.791759
2	0	30.9908	0.090014	8.05064	1.94591
2	0	32.0126	0.057659	7.08589	2.079442
2	0	29.9541	0.049063	6.87247	2.197225
2	0	28.0426	0.06962	6.06931	2.302585
2	0	30.0662	0.075671	6.03052	2.397895
2	0	31.8066	0.102615	7.14737	2.484907
2	0	34.7778	0.101303	7.5588	2.564949
2	0	34.6183	0.094121	7.53711	2.639057
2	0	33.3374	0.096944	7.7253	2.70805
2	0	30.891	0.050647	4.93295	1.386294
2	0	30.891	0.039712	4.54041	1.609438
2	0	31.0106	0.013662	4.56984	1.791759
2	0	31.2631	0.009143	4.5003	1.94591

2	0	31.2631	0.013311	4.50913	2.079442
2	0	31.2631	0.014303	4.59913	2.197225
2	0	31.2631	0.014287	4.53043	2.302585
2	0	31.2631	0.017204	4.82916	2.397895
2	0	29.0531	0.020936	4.49468	2.484907
2	0	29.0531	0.013628	4.60786	2.564949
2	0	28.408	0.024646	4.37302	2.639057
2	0	39.038	0.062248	5.20382	1.386294
2	0	35.6864	0.164459	3.36384	1.609438
2	0	35.6864	0.023702	3.33644	1.791759
2	0	35.6864	0.007587	3.37361	1.94591
2	0	35.6864	0.099649	4.50959	2.079442
2	0	32.3783	0.092646	4.04424	2.197225
2	0	32.3783	0.047191	4.01592	2.302585
2	0	32.3783	-0.0564	4.35389	2.397895
2	0	32.3783	0.069308	3.93384	2.484907
2	0	32.3783	0.074973	4.10507	2.564949
2	0	50.7333	0.108442	5.60723	2.639057
2	0	46.032	0.128267	6.69678	2.079442
2	0	48.96	0.131073	5.20385	2.197225
2	0	46.44	0.102086	4.3565	2.302585
2	0	41.3	0.071648	4.22915	2.397895
2	0	41.43	0.047004	3.93673	2.484907
2	0	36.9087	0.049845	4.10441	2.564949
2	0	37.1607	0.034318	4.61511	2.639057
1	0	50.19	0.041967	2.082	1.386294
1	0	39.48	0.041651	3.2189	1.609438
1	0	38.97	0.034301	2.20546	1.791759
1	0	32.67	0.012338	2.279	1.94591
1	0	32.67	0.012986	1.38881	2.079442
1	0	32.67	0.002598	1.52467	2.197225
1	0	27.35	-0.05595	0.39515	2.302585
1	0	24.52	-0.01786	0.07999	2.397895
1	0	21.298	0.003137	4.894	1.386294
1	0	21.298	0.028579	4.8716	1.609438
1	0	21.298	0.012113	4.70658	1.791759
1	0	21.298	-0.10719	4.76427	1.94591
1	0	21.298	-0.0002	4.95526	2.079442
1	0	34.4	0.149797	5.54135	0
1	0	34.85	0.099581	5.1862	0.693147
1	0	36.06	0.102756	5.07461	1.098612
1	0	36.86	0.058572	4.74275	1.386294
1	0	37.12	-0.12624	4.66059	1.609438
1	0	10.23	-0.05691	3.08882	1.098612
1	0	10.23	0.004114	3.60592	1.386294
1	0	31.675	0.087484	5.09414	0
1	0	31.6931	0.101114	4.00258	0.693147
1	0	31.7534	0.049142	3.72839	1.098612
1	0	34.4034	0.028255	3.305	1.386294
1	0	34.4034	0.00192	3.60072	1.609438
1	0	28.5051	0.053181	8.51363	0.693147
1	0	28.5051	0.001778	8.17114	1.098612
1	0	28.6176	0.004878	7.89518	1.386294
1	0	28.6176	0.009735	7.8416	1.609438
1	0	7.5856	-0.01976	1.98939	1.791759
1	0	7.7611	0.055389	2.07578	1.94591
1	0	7.7611	0.146724	2.07444	2.079442
2	0	10.165	0.083389	3.07558	2.302585

1	0	47.55	0.037304	0	1.386294
1	0	44.86	0.033123	0.149	1.609438
1	0	42.15	0.047493	0.16951	1.791759
1	0	42.15	0.025856	0.16673	1.94591
1	0	39.66	0.02811	0.14783	2.079442
1	0	39.66	0.027368	0.08532	2.197225
1	0	32.35	0.03629	0.26111	2.302585
1	0	32.94	0.015842	0.44299	2.397895
1	0	33.11	0.015913	0.15765	2.484907
1	2	45.9068	0.063716	8.34983	1.386294
1	2	45.9068	0.064896	7.57004	1.609438
1	2	45.9068	0.037055	7.53884	1.791759
1	2	45.9068	0.014454	7.51839	1.94591
1	2	49.3816	0.02001	8.01324	2.079442
1	2	44.3088	0.033569	6.65784	2.197225
1	2	37.9984	0.038295	6.55349	2.302585
1	2	32.912	0.057353	7.02343	2.397895
1	2	32.1912	0.086356	6.76398	2.484907
1	2	32.4088	0.075947	6.50606	2.564949
1	2	32.4088	0.068046	7.12909	2.639057
1	0	18.88	0.109993	1.42552	1.386294
1	0	18.88	0.11809	0.46	1.609438
1	0	18.88	0.112329	0.852	1.791759
1	0	18.88	0.058055	0.50119	1.94591
1	0	18.88	0.0757	0.53052	2.079442
1	0	18.88	0.07308	0.55685	2.197225
1	0	18.88	0.042314	1.61384	2.302585
1	0	18.88	0.04425	2.00117	2.397895
0	1	16.2447	0.015758	2.29871	1.386294
0	1	16.2642	0.026028	2.27766	1.609438
0	1	16.8589	0.018687	2.42685	1.791759
0	1	15.4461	0.00231	2.48113	1.94591
0	1	13.7581	0.013353	2.92411	2.079442
0	1	13.8872	0.009668	3.12229	2.197225
0	1	14.3733	0.040797	3.02203	2.302585
0	1	13.5605	0.053441	3.71087	2.397895
0	1	16.6736	0.05588	3.59764	2.484907
0	1	15.2936	0.048028	3.66734	2.564949
0	1	12.8572	0.036432	3.71272	2.639057
1	2	21.851	0.026161	5.83226	2.197225
1	2	22.7236	0.02536	5.44239	2.302585
1	2	22.7236	0.046566	5.62152	2.397895
1	2	21.8202	0.03965	5.84547	2.484907
1	2	21.8202	0.014556	5.70593	2.564949
1	2	25.1986	0.044597	5.71632	2.639057
1	0	44.6329	0.055878	4.86113	1.386294
1	0	44.6329	0.106984	4.9317	1.609438
1	0	49.2494	0.112992	6.11595	1.791759
1	0	49.2494	0.107683	6.14713	1.94591
1	0	47.9694	0.09176	7.26498	2.079442
1	0	43.4061	0.104605	6.96429	2.197225
1	0	37.8714	0.103134	7.11098	2.302585
1	0	37.8714	0.116529	7.1957	2.397895
1	0	35.1214	0.099493	7.23687	2.484907
1	0	35.4442	0.074902	7.1097	2.564949
1	0	35.4442	0.057998	7.1202	2.639057
1	0	14.539	0.034762	2.81683	1.386294
1	0	14.539	0.039885	5.14548	1.609438

1	0	15.3021	0.045945	5.20224	1.791759
1	0	15.3021	0.043453	3.34389	1.94591
1	0	15.3021	0.02339	2.56312	2.079442
1	0	15.3021	0.016471	3.23234	2.197225
1	0	19.2206	0.013356	5.57113	2.302585
1	0	19.2206	0.019214	5.21368	2.397895
1	0	15.7332	0.029973	4.85057	2.484907
2	0	38.86	0.102685	2.05831	1.386294
2	0	35.37	0.109098	3.24623	1.609438
2	0	35.37	0.116359	2.42455	1.791759
2	0	35.81	0.063177	1.70125	1.94591
2	0	34.36	0.038731	0.68948	2.079442
2	0	34.51	0.041525	1.66665	2.197225
2	0	34.51	0.0324	2.09808	2.302585
2	0	34.51	0.010627	1.82024	2.397895
2	0	34.51	-0.0359	1.37295	2.484907
2	0	34.51	-0.11062	1.01379	2.564949
0	1	69.35	0.128544	0.8294	1.386294
0	1	59.92	0.072094	1.45904	1.609438
0	1	59.92	0.062964	1.08488	1.791759
0	1	59.92	0.031967	0.32007	1.94591
0	1	59.92	0.047572	0.25787	2.079442
0	1	56.98	0.018954	0.20248	2.197225
0	1	56.98	0.013749	0.14722	2.302585
0	1	56.98	0.017212	0.31353	2.397895
0	1	50.1301	0.033791	1.40652	2.484907
0	1	52.19	0.02317	1.3195	2.564949
0	1	51.8321	0.024172	1.18116	2.639057
1	0	26.51	0.038775	2.08107	1.386294
1	0	26.41	0.046172	1.62039	1.609438
1	0	26.35	0.041928	1.39601	1.791759
1	0	26.57	0.001026	1.44188	1.94591
1	0	26.57	0.004623	0.9067	2.079442
1	0	21.71	0.000621	2.06345	2.197225
1	0	21.71	-0.00558	1.14874	2.302585
1	0	26.69	0.009255	0.90439	2.397895
1	0	24.79	0.016958	2.71954	2.484907
1	0	24.79	0.03224	2.43934	2.564949
1	0	35.98	0.030453	1.21831	1.386294
1	0	29.03	0.075302	3.16489	1.609438
1	0	28.87	0.072498	4.20653	1.791759
1	0	28.72	0.010544	1.16885	1.94591
1	0	22.62	-0.04706	0.58241	2.079442
1	1	48.05	0.010716	5.59301	0
1	1	45.73	0.105469	4.80132	0.693147
1	1	42.81	0.032519	4.6997	1.098612
1	1	42.85	0.04585	4.46043	1.386294
1	1	42.92	0.085995	4.35667	1.609438
1	1	41.56	0.114237	4.27821	1.791759
3	0	50.52	0.005314	0.843	1.386294
3	0	42.1	-0.05156	1.41619	1.609438
3	0	42.1	-0.07042	0.71129	1.791759
3	0	42.1	0.012704	0.66869	2.079442
3	0	24.22	0.000717	1.46999	2.197225
3	0	34.59	0.039268	1.6707	2.302585
3	0	24.65	0.034765	1.155	2.397895
3	0	20.88	0.033878	2.15562	2.484907
2	0	30.94	0.15849	1.48457	1.098612

2	0	30	0.141797	1.57806	1.386294
2	0	30	0.134335	0.90862	1.609438
2	0	30	0.117914	0.9592	1.791759
2	0	30	0.163339	1.03455	1.94591
2	0	22.25	0.184531	0.81142	2.079442
2	0	21.56	0.194808	1.26795	2.197225
2	0	20.17	0.075102	0.49009	2.302585
0	1	15.6956	0.044647	6.424	1.098612
0	1	15.6956	0.034341	6.121	1.386294
0	1	15.6956	0.005504	6.54575	1.609438
0	1	15.6956	0.011092	6.08892	1.791759
0	1	15.6956	-0.0044	5.791	1.94591
0	1	15.6956	-0.00266	5.70499	2.079442
0	1	15.6956	-0.05286	5.27823	2.197225
1	1	15.6956	0.010493	5.93027	2.302585
1	1	15.6956	0.029575	5.06273	2.397895
1	1	11.9581	-0.0047	5.19338	2.484907
1	1	11.9581	0.014517	5.11298	2.564949
1	1	52.1728	0.055632	4.978	1.098612
1	1	50.9193	0.053871	5.50934	1.386294
1	1	32.2257	0.047973	5.83458	1.609438
1	1	32.4956	0.048788	4.2695	1.791759
1	1	31.2791	0.044523	4.46247	1.94591
1	1	30.8146	0.044345	4.72987	2.079442
1	1	27.4775	0.053346	5.30251	2.197225
1	1	25.1829	0.043967	4.62776	2.302585
1	1	25.1829	0.026243	4.3066	2.397895
1	1	25.9965	0.051342	3.83093	2.484907
1	1	26.5043	0.069041	3.52262	2.564949
1	0	54.5127	0.093231	6.1005	1.098612
1	0	43.9129	0.067399	7.28581	1.386294
1	1	43.9128	0.077902	6.6626	1.609438
1	0	43.9129	0.02834	5.05107	1.791759
1	0	43.9129	0.013357	5.02251	1.94591
1	0	43.9129	0.011585	5.35802	2.079442
1	0	39.3931	0.007849	5.13663	2.197225
1	0	40.14	0.024522	5.35892	2.302585
1	0	40.14	0.041828	5.72626	2.397895
1	0	40.0782	0.017541	5.28914	2.484907
1	0	28.8277	0.00659	4.54897	2.564949
0	1	12.9096	0.043559	4.35943	1.098612
0	1	11.5881	0.048115	4.5656	1.386294
0	1	11.5881	0.004939	3.60284	1.609438
0	1	11.5881	0.003659	3.27082	1.791759
0	1	13.6719	0.013571	3.26171	1.94591
1	0	21.7178	0.004857	4.0491	0
1	0	27.5624	0.102267	5.56164	1.098612
1	0	29.7063	0.10717	4.97884	1.386294
1	0	29.412	0.061765	4.96897	1.609438
1	0	29.732	0.031041	4.89735	1.791759
1	0	29.732	0.003829	4.52869	1.94591
1	0	29.732	0.004713	4.50323	2.079442
1	0	31.1049	0.007072	4.81813	2.197225
1	0	29.97	-0.03874	4.83475	0
1	0	29.6703	0.005229	4.506	0.693147
1	0	10.4892	0.052388	4.91582	1.098612
1	0	10.4907	0.082761	2.23292	1.386294
1	0	8.7494	0.046134	3.85897	1.609438

1	0	8.7494	0.018553	3.01279	1.791759
1	0	8.5145	0.033582	2.36439	1.94591
1	0	8.493	0.046427	2.4776	2.079442
1	0	12.1507	0.054208	4.21564	2.197225
1	0	7.9327	0.056358	3.82486	2.302585
1	0	7.138	0.0514	3.85266	2.397895
1	0	7.138	0.050686	3.61727	2.484907
1	0	7.225	0.041478	3.8681	2.564949
1	0	38.38	0.054216	0.1243	1.098612
1	0	34.66	0.038673	1.61428	1.386294
1	0	34.66	0.023811	0.38042	1.609438
1	0	34.76	0.011348	0.40219	1.791759
1	0	34.76	0.010163	0.39236	1.94591
1	0	34.76	0.013748	0.3967	2.079442
1	0	34.54	0.014612	0.31228	2.197225
1	0	29.12	0.025274	1.65001	2.302585
1	0	29.12	0.011612	1.5969	2.397895
1	0	21.16	-0.03929	0.90404	2.484907
2	0	21.16	0.017635	0.67377	2.564949
1	0	41.1068	0.025219	3.55471	1.098612
1	0	35.7168	0.031126	2.6353	1.386294
1	0	35.04	0.048821	2.62338	1.609438
1	0	35.04	0.038153	2.58627	1.791759
1	0	35.04	0.030314	2.25884	1.94591
1	0	31.56	0.017532	3.93019	2.079442
1	0	31.56	-0.00664	4.17863	2.197225
1	0	31.56	0.049901	2.30773	2.302585
1	0	31.58	0.053344	2.37625	2.484907
2	0	32.47	0.03224	2.33293	2.564949
1	0	35.18	0.026193	0.748	1.098612
1	0	24.95	0.026986	1.71923	1.386294
1	0	24.95	0.048348	1.19293	1.609438
1	0	24.95	0.074997	1.11749	1.791759
1	0	21.36	0.037264	2.29146	1.94591
1	0	21.36	0.053586	0.13436	2.079442
1	0	21.36	0.060235	0.20561	2.197225
1	0	21.36	0.048885	0.19308	2.302585
1	0	21.36	0.046813	0.2746	2.397895
1	0	22	0.047988	0.28058	2.484907
1	0	22	0.041458	0.27421	2.564949
0	1	7.8369	0.072515	7.454	1.098612
0	1	8.6187	0.072962	7.044	1.386294
0	1	8.6187	0.053859	6.98204	1.609438
0	1	8.6187	0.05082	6.9088	1.791759
0	1	8.6187	0.056076	6.878	1.94591
0	1	8.6187	0.067979	6.979	2.079442
0	1	8.5135	0.077888	6.78238	2.197225
0	1	7.767	0.077854	7.03502	2.302585
0	1	8.5644	0.056578	6.4841	2.397895
0	1	8.5644	0.090125	6.24981	2.484907
0	1	8.5644	0.105087	6.06612	2.564949
0	1	24.1	0.092916	6.53213	1.098612
0	1	21.4876	0.094089	6.41871	1.386294
0	1	21.1228	0.087969	6.04185	1.609438
0	1	20.988	0.098323	5.77518	1.791759
0	1	21.4544	0.034753	4.07877	1.94591
0	1	18.4904	0.030851	1.327	2.079442
0	1	15.6939	0.022061	0.15587	2.197225

0	1	15.9239	-0.09303	0.38099	2.302585
0	1	16.8157	0.007149	0.10319	2.397895
0	1	21.17	0.012138	0.55559	2.484907
0	1	25.79	0.046238	0.11046	2.564949
1	0	18.5976	0.028739	7.76005	1.098612
1	0	15.8946	0.038269	7.4045	1.386294
1	0	15.8946	0.014462	6.5357	1.609438
1	0	15.8946	0.011187	6.31063	1.791759
1	0	15.8946	0.016456	6.54335	1.94591
1	0	15.8946	0.030549	6.43666	2.079442
1	0	13.3812	0.022624	6.46958	2.197225
1	0	13.45	0.016231	5.76169	2.397895
1	0	11.7838	0.002317	6.97373	2.564949
1	0	38.5985	0.034167	5.961	1.098612
1	0	36.3341	0.028322	6.43911	1.386294
1	0	36.3341	0.055205	6.51998	1.609438
1	0	36.7171	0.018059	5.52513	1.791759
1	0	37.1275	-0.00524	5.81725	1.94591
1	0	51.3446	0.005493	7.48736	2.079442
1	0	54.7528	0.034403	7.19599	2.197225
1	0	43.5359	0.084696	5.6753	2.302585
1	0	39.0647	0.033493	5.47747	2.397895
1	0	38.6606	0.009029	5.6972	2.484907
1	0	39.9048	0.05491	4.91196	2.564949
1	0	8.3938	0.102384	7.96595	1.098612
1	0	8.3938	0.154965	8.60022	1.386294
1	0	8.3938	0.145088	7.82607	1.609438
1	0	8.3938	0.106645	7.30882	1.791759
1	0	8.3938	0.130139	7.65632	1.94591
1	0	8.4994	0.14505	7.49327	2.079442
1	0	7.6128	0.068763	7.46154	2.197225
1	0	7.6128	0.06926	7.24202	2.302585
1	0	7.6128	0.071931	6.23246	2.397895
1	0	7.5435	0.062333	6.22311	2.484907
1	0	7.5458	0.069984	6.32011	2.564949
1	0	15.5811	0.141145	6.70993	1.098612
1	0	15.5811	0.085771	4.81031	1.386294
1	0	14.6336	0.053753	4.7194	1.609438
1	0	14.5384	0.047951	4.12918	1.791759
1	0	14.5429	0.024633	3.85636	1.94591
2	0	14.6036	0.056046	3.42455	2.079442
2	0	14.6632	0.060672	3.58139	2.197225
2	0	16.5804	0.054129	4.27756	2.302585
2	0	16.04	0.016246	4.11659	2.397895
2	0	16.04	0.008524	3.99461	2.484907
2	0	16.04	0.023523	4.11986	2.564949
1	0	27.25	0.088694	0.034	1.098612
1	0	25.95	0.050263	0.96282	1.386294
1	0	26.47	0.050517	0.73383	1.609438
1	0	42.8	0.045218	0.57047	1.791759
1	0	42.52	0.023006	0.23807	1.94591
1	0	24.5	-0.07516	0.44742	2.079442
0	2	25.9333	0.07644	5.42638	1.098612
0	2	24.3388	0.030148	5.50552	1.386294
0	2	22.6163	0.014216	4.91466	1.791759
0	2	21.7023	0.007896	4.72526	1.94591
0	2	20.7058	-0.05506	4.55659	2.079442
0	2	23.7669	0.029043	6.4311	2.197225

0	2	22.5152	0.066324	5.54375	2.302585
0	2	22.4518	0.029415	5.04256	2.397895
0	2	21.2675	-0.0071	4.9487	2.484907
0	2	18.3584	-0.08968	4.19776	2.564949
1	0	17.02	0.030277	3.51921	2.397895
1	0	21.95	0.00702	2.83596	2.484907
2	0	51.0147	0.028602	5.9	1.098612
2	0	51.0147	0.024847	5.9	1.386294
2	0	46.2429	0.001614	4.936	1.609438
2	0	46.2429	0.008449	5.07736	1.791759
2	0	46.2429	-0.0588	6.6253	1.94591
0	1	22.84	0.011474	0.8292	1.098612
0	1	22.84	0.014244	0.35	1.386294
0	1	18.04	0.020238	0.31251	1.609438
0	1	18.04	0.020956	0.58497	1.791759
0	1	13.08	0.016459	2.38508	1.94591
0	1	15.09	0.019432	2.67502	2.079442
0	1	15.22	0.033389	2.5558	2.197225
0	1	12.1	0.020303	3.41512	2.302585
2	0	26.1819	0.059181	5.47682	1.098612
2	0	26.1819	0.064926	5.24019	1.386294
2	0	24.0585	0.03458	5.64087	1.609438
2	0	24.3377	0.047643	4.65392	1.791759
2	0	19.3214	0.056387	6.0436	1.94591
2	0	17.3303	0.073539	4.80342	2.079442
2	0	14.8239	0.046449	5.00697	2.197225
2	0	14.25	0.053832	4.52022	2.302585
2	0	15.2466	0.058402	4.12562	2.397895
2	0	15.4242	0.034497	3.57372	2.484907
2	0	15.4006	0.018256	3.96105	2.564949
0	1	8.9095	0.023296	6.61368	1.609438
0	1	8.885	0.009334	6.08538	1.791759
2	0	59.6	0.146139	1.19196	1.098612
2	0	59.6	0.090495	0.47038	1.386294
2	0	59.6	0.069433	0.27131	1.609438
2	0	59.6	0.017691	0.03853	1.791759
2	0	59.6	0.021218	0.05226	1.94591
2	0	59.6	0.05355	0.03604	2.079442
2	0	58.7	0.030058	0.40343	2.197225
2	0	57.09	-0.0648	0.56843	2.302585
2	0	37.13	0.107239	2.04444	2.564949
1	0	24.86	0.008772	2.223	1.098612
1	0	24.86	0.039695	2.223	1.386294
1	0	24.86	0.057139	2.223	1.609438
1	0	24.86	0.030264	1.95846	1.791759
1	0	24.56	0.006022	2.72015	1.94591
1	0	17.37	0.008011	3.2214	2.079442
1	0	17.65	0.020737	2.71452	2.197225
1	0	17.66	0.040918	1.27418	2.302585
1	0	16.47	0.040121	0.87488	2.397895
1	0	24.2135	0.014735	5.1357	1.098612
1	0	24.2135	0.015217	5.103	1.386294
1	0	24.2135	0.016797	5.11052	1.609438
1	0	24.2135	0.016982	5.11743	1.791759
1	0	24.0996	0.000842	5.05832	1.94591
1	0	28.9094	0.006527	5.38105	2.079442
1	0	26.8756	0.007194	5.26964	2.197225
1	0	28.6329	0.001617	4.92573	2.302585

1	0	28.6329	-0.00131	5.06119	2.397895
1	0	35.5067	0.00802	2.31707	2.484907
2	1	26.3467	0.039742	7.19501	0.693147
1	1	36.008	0.044041	7.2459	1.098612
1	1	26.202	0.033509	6.87179	1.386294
1	1	37.824	0.036286	7.01666	1.609438
1	1	37.068	0.039337	7.26279	1.791759
1	1	38.8769	0.054999	6.89497	1.94591
1	1	42.2697	0.051915	7.10148	2.079442
1	1	46.0515	0.044289	6.97398	2.197225
1	1	42.4365	0.047144	6.74637	2.302585
1	1	43.1777	0.04821	7.22035	2.397895
1	1	43.1777	0.044166	6.8529	2.484907
1	0	31.92	0.107798	3.58225	0.693147
1	0	31.92	0.082925	3.09534	1.098612
1	0	25.4	0.075821	3.48205	1.386294
1	0	25.4	0.028021	2.27544	1.609438
1	0	25.4	0.030824	2.13659	1.791759
1	0	25.4	0.043375	1.84478	1.94591
1	0	25.28	0.054408	2.41836	2.079442
1	0	23.34	0.061084	2.60036	2.197225
1	0	23.24	0.071965	2.51637	2.302585
2	0	33	0.073702	2.01733	2.397895
2	0	33.44	0.059301	2.24575	2.484907
1	1	28.9525	0.026795	5.83292	0.693147
1	1	24.4799	0.036917	5.70153	1.098612
1	1	24.29	-0.00748	5.66176	1.386294
1	1	24.29	0.001696	5.11737	1.609438
1	1	19.5559	0.011104	4.48546	1.94591
1	1	19.5559	-0.05807	3.9619	2.079442
1	1	19.5559	0.011074	3.85691	2.197225
1	1	19.5559	0.020134	3.82865	2.302585
1	1	19.5559	0.010774	3.82107	2.397895
2	1	69.11	0.106345	0.11564	0.693147
2	1	62.47	0.085386	1.10841	1.098612
2	1	62.47	0.080138	0.54434	1.386294
2	1	60.35	0.04621	0.55946	1.609438
2	1	57.41	0.017198	0.35289	1.791759
1	1	54.43	0.017212	0.04107	1.94591
1	1	54.63	0.017867	0.41616	2.079442
1	1	54.63	0.007857	0.1349	2.197225
1	1	23.8	0.044282	3.14091	2.302585
2	1	21.58	0.048957	3.41355	2.397895
2	1	12.864	0.036289	4.00798	2.484907
2	0	60.9506	0.054941	3.50023	0.693147
2	0	60.9506	0.064497	4.31689	1.098612
2	0	56.956	0.048466	4.19252	1.386294
2	0	56.956	0.043688	4.04911	1.609438
2	0	55.956	0.031854	3.58686	1.791759
2	0	57.255	0.022855	3.69731	1.94591
2	0	55.495	0.009086	3.20892	2.079442
2	0	54.7896	0.009505	3.42451	2.197225
2	0	54.9369	0.016528	3.02486	2.302585
2	0	62.8357	0.022018	3.33779	2.397895
2	0	60.0476	0.041481	3.78878	2.484907
0	2	71.5525	0.036971	7.3151	0.693147
0	2	71.56	0.070094	7.32828	1.098612
0	2	59.574	-0.06927	6.88909	1.386294

0	2	61.0239	0.002311	6.23159	1.609438
0	2	58.114	0.005061	5.78207	1.791759
0	2	57.604	-0.00155	5.78493	1.94591
0	2	45.65	0.007311	5.89736	2.079442
0	2	45.65	0.026793	5.04683	2.197225
0	2	45.666	0.028376	4.70732	2.302585
0	2	45.686	0.01404	4.72165	2.397895
0	2	44.846	0.012425	4.39483	2.484907
0	1	28.564	0.024545	6.761	0.693147
0	1	53.09	0.020134	6.4793	1.098612
0	1	50.77	0.020354	5.87421	1.386294
0	1	51.95	0.018735	5.24309	1.609438
0	1	44	0.01219	5.20377	1.94591
1	0	56.086	0.158459	5.75563	0.693147
1	0	67.13	0.151685	5.97763	1.098612
1	0	62.24	0.175697	5.86711	1.386294
1	0	62.24	0.159155	6.06008	1.609438
1	0	62.24	0.142586	6.12178	1.791759
1	0	50.46	0.139311	5.42965	1.94591
1	0	50.93	0.125324	4.61229	2.079442
1	0	44.57	0.086369	4.37706	2.197225
1	0	44.57	0.094672	3.82983	2.302585
1	0	44.57	0.108644	4.97051	2.397895
1	0	44.57	0.095606	4.51323	2.484907
0	2	51.83	0.083446	0	0.693147
0	2	51.83	0.103172	0.0279	1.098612
0	2	51.83	0.063375	0.00635	1.386294
0	2	51.83	0.083141	0.07549	1.609438
0	2	37.96	0.12501	2.25891	1.791759
0	2	36.59	0.142051	0.11473	1.94591
0	2	36.49	0.154533	1.45687	2.079442
0	2	37.86	0.126101	0.11704	2.197225
0	2	37.84	0.081058	0.00384	2.302585
0	2	37.81	0.030135	0.00101	2.397895
0	2	34.58	0.046104	0.12246	2.484907
2	1	36.6	0.08543	0.14836	0.693147
2	1	33.23	0.069379	0.07685	1.098612
2	1	33.21	0.043627	0.03429	1.386294
2	1	31.66	0.033149	0.04453	1.609438
2	1	30.62	0.034092	0.01651	1.791759
2	1	25.69	0.033705	0.13538	1.94591
2	1	30.4398	0.017227	0.86674	2.079442
2	1	30.75	-0.0377	0.3146	2.197225
2	1	30.42	0.00522	0.20157	2.302585
2	1	30.634	0.043557	0.24377	2.397895
2	1	26.3206	0.045738	1.24244	2.484907
1	1	67.3	0.059127	7.564	0.693147
1	1	58.87	0.044678	8.03448	1.098612
1	1	57.26	0.04206	6.94688	1.386294
1	1	40.8199	0.030542	6.64663	1.609438
1	1	40.8199	0.046194	6.73407	1.791759
1	1	40.8199	0.053857	6.72813	1.94591
1	1	38.0766	0.041281	6.45888	2.079442
1	1	50.04	0.080886	5.2895	2.197225
1	1	45.08	0.029413	5.03151	2.302585
1	1	45.079	0.040688	5.77229	2.397895
1	0	66.59	0.070849	1.3196	1.386294
1	0	61.69	0.063812	0.93047	1.609438

1	0	56.78	-0.05976	0.09679	1.791759
1	0	46.97	0.005377	0.16856	1.94591
1	0	46.97	0.005567	0.10474	2.079442
1	0	46.97	0.003517	0.13565	2.197225
0	1	59.25	0.052705	0.88556	0.693147
0	1	59.25	0.064995	0.34703	1.098612
0	1	52.98	0.116407	0.29523	1.386294
0	1	52.98	0.03923	0.13146	1.609438
0	1	52.98	0.072975	0.13639	1.791759
0	1	52.98	0.112346	0.1368	1.94591
0	1	52.94	0.063044	0.03608	2.079442
0	1	29.99	0.067521	0.25462	2.197225
0	1	17.73	0.073702	1.85356	2.302585
1	1	27.29	0.157545	1.85337	2.397895
1	1	26.17	0.147582	1.5036	2.484907
1	0	53.17	-0.04133	1.29591	0.693147
1	0	53.17	0.044573	1.57877	1.098612
1	0	37.85	0.089112	2.82384	1.386294
1	0	37.85	-0.03993	1.76733	1.609438
1	0	37.85	0.070588	2.13328	2.197225
1	0	42.32	0.144969	1.03768	2.397895
0	1	25.524	0.053754	8.0151	0.693147
0	1	25.524	0.018188	7.31	1.098612
0	1	25.524	0.017476	7.392	1.386294
0	1	25.524	0.015791	7.053	1.609438
0	1	23.136	0.065857	6.09321	1.791759
0	1	25.524	0.008092	4.39513	1.94591
0	1	26.1802	0.033341	6.37174	2.079442
0	1	26.3373	0.054027	6.48972	2.197225
0	1	27.1388	0.042317	5.83901	2.302585
0	2	51.53	-0.01639	1.66566	0.693147
0	2	51.53	0.005445	0.86321	1.098612
0	2	51.47	0.003666	0.42559	1.386294
0	2	48.67	-0.04482	0.20964	1.609438
0	1	36.01	0.002185	0.11473	1.791759
0	1	33.52	0.003833	0.12002	1.94591
0	1	28.53	-0.0828	0.16357	2.079442
0	1	15.38	0.008593	1.39384	2.197225
1	0	46.8484	0.033042	6.20706	0.693147
1	0	46.8484	0.048225	6.2023	1.098612
1	0	46.8484	0.022498	6.6237	1.386294
1	0	46.8484	0.036807	5.67131	1.791759
1	0	46.8484	0.021074	5.55509	1.94591
1	0	41.7284	0.035611	5.7274	2.079442
1	0	36.8392	0.039782	5.7986	2.197225
1	0	37.3064	0.038343	5.83666	2.302585
1	0	38.548	0.036004	5.85794	2.397895
1	0	39.9956	0.03065	5.88477	2.484907
0	2	20.3765	0.055535	6.865	0.693147
0	2	20.3765	0.095034	6.89366	1.098612
0	2	18.3302	0.084384	6.72509	1.386294
0	2	17.4304	0.084591	6.42733	1.609438
0	2	17.4304	0.073789	6.77367	1.791759
0	2	13.835	0.082256	6.17607	1.94591
0	2	13.8103	0.1122	5.3376	2.079442
0	2	13.3912	0.052941	4.87315	2.197225
0	2	13.2269	0.081772	4.54949	2.302585
0	2	13.2392	0.050412	3.97302	2.397895

0	2	12.125	0.021257	3.51089	2.484907
1	1	41.95	0.073569	3.84	0.693147
1	1	41.95	0.073808	4.15315	1.098612
1	1	41.95	0.066057	3.84436	1.386294
1	1	41.95	0.08405	4.00591	1.609438
1	1	32.626	0.071889	5.28864	1.791759
1	1	31.79	0.080843	3.35961	1.94591
1	1	21.798	0.064786	2.60106	2.079442
2	2	21.975	0.052057	2.87092	2.197225
0	1	46.12	0.10174	0.22265	0.693147
0	1	46.12	0.119026	1.74945	1.098612
0	1	38.98	0.107654	2.71551	1.386294
0	1	38.98	0.095465	3.36813	1.609438
0	1	36.17	0.098595	3.31065	1.791759
0	1	34.85	0.096394	2.51572	1.94591
0	1	30.49	0.101585	1.27796	2.079442
0	1	30.5	0.044855	2.67263	2.197225
0	1	27.49	0.023983	1.80546	2.302585
0	1	27.49	0.005451	1.80471	2.397895
0	1	27.55	0.020391	1.99992	2.484907
1	0	36.9572	0.054571	6.19861	0.693147
1	0	37.7496	0.044744	6.86217	1.098612
1	0	38.9467	0.050203	6.99163	1.386294
1	0	38.9467	0.056398	6.74649	1.609438
1	0	32.2236	0.049224	6.90386	1.791759
1	0	32.2236	0.039207	5.84716	1.94591
1	0	27.1725	0.017469	4.71573	2.079442
1	0	25.8782	0.009597	5.03428	2.197225
1	0	25.8782	0.009468	5.1702	2.302585
1	0	24.479	0.007663	5.74512	2.397895
1	0	24.479	0.00745	6.1295	2.484907
2	0	55.8495	0.165737	7.81862	0.693147
2	0	55.9767	0.1853	7.74795	1.609438
2	0	56.378	0.09048	8.3861	1.791759
2	0	53.4255	0.054429	8.21062	1.94591
2	0	53.2499	0.128109	8.15668	2.079442
2	0	43.638	0.124789	8.31831	2.197225
2	0	44.2721	0.057553	8.04726	2.302585
2	0	39.7943	0.05112	7.43565	2.484907
1	0	14.851	0.075399	4.85477	0.693147
1	0	14.851	0.087749	5.43245	1.098612
1	0	12.9254	0.059689	4.46137	1.386294
1	0	12.9113	0.066896	3.88603	1.609438
1	0	11.6747	0.07335	4.59947	1.791759
1	0	11.4773	0.014481	3.32113	1.94591
1	0	10.702	0.023047	4.2188	2.079442
1	0	9.7216	0.028265	2.70512	2.197225
1	0	9.7216	0.032022	2.3541	2.302585
1	0	10.8416	0.033806	2.32733	2.397895
1	0	10.8416	0.033259	2.30384	2.484907
1	0	25.9212	0.061245	2.48611	0.693147
1	0	25.9212	0.065673	2.93761	1.098612
1	0	25.9213	0.067764	2.57638	1.386294
1	0	27.012	0.065164	2.00156	1.609438
1	0	25.3152	0.036034	1.84726	1.791759
1	0	25.3146	0.020292	0.43196	1.94591
1	0	20.8635	0.028557	0.18962	2.079442
1	0	15.6939	0.022457	0.45185	2.197225

1	0	13.5668	0.015275	0.72583	2.302585
1	0	11.5536	-0.01914	1.32573	2.397895
1	0	30.6523	0.009768	5.062	0.693147
1	0	25.8188	0.018149	4.2324	1.098612
1	0	25.8188	-0.00681	5.20476	1.386294
1	0	34.5095	0.005817	3.72985	1.609438
1	0	29.3804	0.005002	3.54713	1.791759
1	0	31.9839	0.005784	3.62359	1.94591
1	0	28.4351	0.005914	3.54465	2.079442
1	0	24.745	0.024505	4.44621	2.197225
1	0	24.745	0.035248	4.40625	2.302585
2	0	48.71	0.130002	3.39617	0.693147
2	0	48.71	0.135486	2.70658	1.098612
2	0	48.71	0.08234	1.46672	1.386294
2	0	47.79	0.040552	0.25541	1.609438
2	0	47.09	0.048195	0.24038	1.791759
2	0	46.03	0.055875	0.09706	1.94591
2	0	34.64	0.049543	1.14489	2.079442
2	0	33.55	0.048709	1.00637	2.197225
2	0	33.59	0.015158	0.79568	2.302585
0	1	15.95	0.007023	0.52532	2.484907
1	0	42.9255	0.030714	7.0264	0.693147
1	0	44.9141	0.041713	6.95961	1.098612
1	0	30.3792	0.028004	5.89773	1.386294
1	0	35.7758	-0.02387	5.59671	1.609438
1	0	30.144	0.002299	4.61396	1.791759
1	0	31.152	-0.00246	4.4113	1.94591
1	0	22.912	-0.00082	3.56675	2.079442
1	0	18.512	-0.04844	3.13653	2.197225
0	1	58.3649	0.020845	6.61963	1.94591
0	1	48.8686	-0.0621	6.58438	2.079442
0	1	48.8686	0.005844	7.04076	2.197225
0	1	49.1305	-0.04607	7.03674	2.302585
0	1	49.1305	0.005599	7.23523	2.397895
2	0	33.34	0.099948	5.94272	0
2	0	33.34	0.102948	5.94553	0.693147
2	0	33.34	0.096199	5.94263	1.098612
2	0	33.34	0.042285	5.80328	1.386294
2	0	33.34	0.041001	4.80822	1.609438
0	2	26.758	0.122437	5.61801	0.693147
0	2	26.758	0.166562	6.3516	1.098612
0	2	25.5255	0.117327	6.32322	1.386294
0	2	25.534	0.127288	6.20668	1.609438
0	2	21.233	0.093289	3.98902	1.791759
0	2	20.774	0.087003	3.24103	1.94591
0	2	19.329	0.049374	4.60273	2.079442
0	2	18.0795	0.075863	3.38775	2.197225
0	2	16.762	0.070349	4.34743	2.302585
0	2	9.401	0.084455	3.71771	2.397895
0	2	9.1035	0.078114	4.06552	2.484907
2	0	39.9594	0.149298	6.1455	0
2	0	39.9594	0.129908	6.39389	0.693147
2	0	39.9594	0.132621	6.30958	1.098612
2	0	39.9594	0.133252	6.31795	1.386294
2	0	39.9594	0.156282	6.20184	1.609438
2	0	41.26916	0.159815	6.34861	1.791759
2	0	41.26916	0.13649	6.44263	1.94591
2	0	41.3425	0.137171	6.36549	2.079442

2	0	41.3425	0.143797	6.29804	2.197225
2	0	41.46567	0.121354	6.40486	2.302585
2	0	41.79709	0.114809	6.39264	2.397895
0	1	50.9668	0.05836	5.99583	0
0	1	50.9668	0.052053	5.98584	0.693147
0	1	54.1218	0.043583	6.0843	1.098612
0	1	48.921	0.045089	6.26203	1.386294
0	1	48.4039	0.05439	6.03751	1.609438
0	1	44.0346	0.050614	5.75929	1.791759
0	1	40.2409	0.054757	5.06911	1.94591
0	1	35.9763	0.041515	4.14518	2.079442
0	1	33.5709	0.015507	3.74521	2.197225
0	2	34.2104	0.015687	3.0118	2.302585
0	2	37.46	0.030651	2.82251	2.397895
1	0	25.1044	0.057306	6.22454	0
1	0	25.1044	0.067423	6.2119	0.693147
1	0	25.2759	0.060234	6.61035	1.098612
1	0	25.2759	0.073605	5.70564	1.386294
1	0	25.1473	0.062459	4.1921	1.609438
1	0	22.0467	0.04237	3.70338	1.791759
1	0	22.4083	0.028572	4.02358	1.94591
1	0	18.6153	0.018821	5.78886	2.079442
1	0	18.7134	0.020959	4.34148	2.197225
1	0	18.7134	0.025312	3.75546	2.302585
1	0	19.6683	0.012619	3.65819	2.397895
2	0	50.23	0.062145	0.07759	0
2	0	50.23	0.061254	0.17971	0.693147
2	0	50.23	0.053756	0.51338	1.098612
2	0	49.47	0.033589	0.2988	1.386294
2	0	47.38	0.035042	0.00344	1.609438
2	0	45.23	0.030304	0.02812	1.791759
2	0	60.63	0.066881	0.0832	0
2	0	60.63	0.077356	0.37955	0.693147
2	0	60.63	0.06175	0.0718	1.098612
2	0	60.63	0.006278	0.07256	1.386294
2	0	57.53	0.014942	0.06	1.609438
2	0	55.14	0.030183	0.70337	1.791759
2	0	54.67	0.006368	0.3009	1.94591
2	0	54.14	0.01212	0.26266	2.079442
2	0	44.3	0.021702	0.11314	2.197225
2	0	45.62	0.032597	0.32141	2.302585
2	0	45.62	0.026366	0.23832	2.397895
2	0	30.88	0.012421	1.56112	2.302585
2	0	23.96	0.005524	1.51428	2.397895
3	0	70.89	0.071981	1.44645	1.609438
3	0	68.92	0.10038	0.99051	1.791759
3	0	67.02	0.099953	1.52214	1.94591
3	0	60.21	0.057036	0.54511	2.079442
3	0	60.26	0.008196	0.55262	2.197225
3	0	54.92	0.016665	1.66402	2.397895
2	1	31.3445	0.093828	7.87967	0
2	1	31.3445	0.129524	8.91121	0.693147
2	1	31.3445	0.173701	9.55647	1.098612
2	1	31.3445	0.163301	8.98145	1.386294
2	1	31.3445	0.125557	8.25296	1.609438
2	1	31.3445	0.127831	7.98969	1.791759
2	1	16.511	0.121408	4.54436	1.94591
2	1	16.851	0.080511	4.55681	2.079442

2	1	16.7795	0.098748	4.86696	2.197225
2	1	17.931	0.108813	4.67362	2.302585
2	1	17.898	0.1041	4.74845	2.397895
1	1	67.6629	0.184012	7.89373	2.302585
1	1	67.6629	0.088169	7.46795	2.397895
1	0	14.679	0.025042	4.8771	0
1	0	18.21	0.114798	1.64748	0.693147
1	0	18.21	0.099099	1.06835	1.098612
1	0	18.21	0.026449	0.34917	1.386294
1	0	16.11	0.045711	1.09355	1.609438
1	0	16.11	0.052041	0.9122	1.791759
1	0	15.31	0.034962	0.42461	1.94591
1	0	15.31	0.038063	1.69112	2.079442
1	0	12.61	-0.06507	0.43173	2.197225
1	0	10.75	0.016815	0.54264	2.302585
1	0	9.31	0.035206	0.09667	2.397895
0	1	63.45	0.091955	7.75926	0
0	1	63.45	0.086784	7.50536	0.693147
0	1	57.11	0.093607	7.36415	1.098612
0	1	57.11	-0.00516	6.88059	1.386294
0	1	57.11	-0.03191	6.77976	1.609438
0	1	57.11	0.003783	6.5408	1.791759
0	1	46.65	-0.04553	7.68113	1.94591
0	1	45.4	0.06031	7.62644	2.079442
0	1	49.9951	0.020132	6.94708	2.197225
0	1	49.995	0.105844	7.37349	2.302585
1	1	70.087	0.073017	2.47675	0
1	1	71.1225	0.052178	2.4286	0.693147
1	1	70.087	0.060632	2.5121	1.098612
1	1	49.52	0.068559	2.63378	1.386294
1	0	17.728	0.038049	4.45513	2.302585
1	0	56.2225	0.071099	1.68073	0
1	0	56.2225	0.057319	1.57233	0.693147
1	0	56.3812	0.029574	1.60373	1.098612
1	0	56.472	0.024993	1.16863	1.386294
1	0	56.45	0.016879	1.81443	1.609438
1	0	48.6809	0.014589	2.10803	1.791759
1	0	48.4011	0.024651	1.07639	1.94591
1	0	44.6915	0.003708	1.83742	2.079442
1	0	43.3917	0.012065	2.52074	2.197225
1	0	32.1457	-0.00811	4.24097	2.302585
1	0	32.1457	0.005649	3.62956	2.397895
0	1	37.33	0.05922	1.26831	0
0	1	37.33	0.05893	1.1569	0.693147
0	1	37.33	0.049141	1.37947	1.098612
0	1	35.63	0.043944	1.54518	1.386294
0	1	36.01	0.049515	1.77054	1.609438
0	1	36.16	0.033249	1.1856	1.791759
0	1	30.53	-0.00552	2.63524	1.94591
0	1	25.54	-0.06125	0.24207	2.302585
0	1	25.54	0.010814	0.08001	2.397895
1	1	33.2535	0.03085	7.62564	0
1	1	33.2535	0.03499	7.07075	0.693147
1	1	33.2535	0.025727	5.9392	1.098612
1	1	19.1959	0.006958	4.22166	1.791759
0	2	32.74	0.071498	0.11763	0
0	2	32.74	0.088727	0.27451	0.693147
0	2	32.74	0.0959	0.53057	1.098612

0	2	32.74	0.10449	1.26269	1.386294
0	2	32.74	0.094585	1.58613	1.609438
0	2	31.9	0.093645	1.01071	1.791759
0	2	31.92	0.088671	0.66842	1.94591
0	2	27.09	0.099514	0.67342	2.079442
0	2	27.1	0.087107	0.88813	2.197225
0	2	26.05	0.082089	1.15374	2.302585
1	0	29.85	0.07454	3.43571	0
1	0	29.85	0.06011	3.4335	0.693147
1	0	29.85	0.055571	3.46111	1.098612
1	0	29.85	0.04314	3.51	1.386294
1	0	29.85	0.02948	3.40988	1.609438
1	0	29.85	0.012863	3.62153	1.791759
1	0	43.4986	0.046156	3.16953	0
1	0	43.4986	0.057728	4.47569	0.693147
1	0	42.5123	0.068083	4.7944	1.098612
1	0	42.5256	0.081881	4.75582	1.386294
1	0	39.4501	0.07598	3.47299	1.609438
1	0	33.2407	0.054333	2.65164	1.791759
1	0	32.5323	0.050859	2.0623	1.94591
1	0	32.5623	0.012925	1.52135	2.079442
1	0	32.2258	0.011457	0.79527	2.197225
1	0	33.4058	-0.03524	0.72499	2.302585
2	0	31.64	0.006822	0.67839	2.397895
2	0	61.95	0.045308	0.9412	0
2	0	61.95	0.047201	2.35459	0.693147
2	0	61.95	0.038259	2.28175	1.098612
2	0	61.95	0.036411	1.22257	1.386294
2	0	57.79	0.009628	1.02305	1.609438
2	0	48.71	0.03296	2.03222	1.791759
2	0	49.02	0.041703	1.20711	1.94591
2	0	48.73	0.061804	1.00339	2.079442
2	0	47.33	0.031913	1.11192	2.197225
2	0	37.47	0.064824	1.55008	2.302585
2	0	34.08	0.037947	1.60649	2.397895
0	2	62.4	0.067961	0.1776	0
0	2	62.4	0.088063	1.284	0.693147
0	2	62.4	0.122222	1.77069	1.098612
0	2	62.5	0.125473	1.47871	1.386294
0	2	62.39	0.132196	1.13617	1.609438
0	2	61.05	0.134987	0.86377	1.791759
0	2	54.29	0.137039	0.62255	1.94591
0	2	54.91	0.124431	0.97002	2.079442
0	2	50.69	0.12298	1.40956	2.197225
0	2	49.96	0.118848	1.25565	2.302585
0	2	51.2	0.107789	1.36989	2.397895
2	0	65.95	0.085801	0.33536	0
2	0	65.95	0.065729	0.49958	0.693147
2	0	65.95	0.022359	0.70463	1.098612
1	0	66.4	0.014703	0.06828	1.386294
1	0	51.4	0.024975	0.29422	1.609438
1	0	51.4	0.04044	0.23219	1.791759
0	1	67.6267	0.043115	7.851	0
0	1	67.6267	0.034279	8.18104	0
0	1	67.6267	0.029033	7.82185	0.693147
0	1	67.6267	0.028715	7.59038	1.098612
0	1	67.6267	0.025565	7.37049	1.386294
0	1	67.6267	0.022025	7.46048	1.609438

0	1	67.6267	0.012865	7.31438	1.791759
0	1	67.6267	0.004647	7.45735	1.94591
0	1	67.6267	0.003492	7.3397	2.079442
0	1	67.6267	0.004437	7.29592	2.197225
0	1	59.7182	0.004297	6.89377	2.302585
0	2	70.35	0.072661	7.5	0
0	2	70.35	0.049901	7.5	0
0	2	69.94	0.0622	7.08937	0.693147
0	2	69.96	0.058173	7.0192	1.098612
0	2	70.23	0.05811	7.03927	1.386294
0	2	65.46	0.029662	6.60734	1.609438
0	2	65.46	0.126792	6.82781	1.791759
0	2	65.46	0.079911	7.04602	1.94591
0	2	65.46	-0.02487	6.57165	2.079442
0	2	65.46	-0.08691	6.58807	2.197225
1	0	68.32	0.069354	10	0
1	0	71.3381	0.000991	7.761	1.386294
1	0	66.834	0.135129	9	0
1	0	50.1255	0.057083	6.976	0
1	0	50.1255	0.022096	6.92629	0.693147
1	0	50.1255	0.007539	6.875	1.098612
1	0	46.5025	0.02645	6.232	1.386294
1	0	46.5292	0.030803	6.77346	1.609438
1	0	24.9545	-0.0571	3.33257	1.791759
1	0	19.4042	0.010692	3.40435	1.94591
1	0	19.4042	0.008376	3.34004	2.079442
1	0	19.4042	0.006016	3.33808	2.197225
1	0	19.4042	0.004977	3.36527	2.302585
1	0	70.15	0.059562	5.54006	0
1	0	70.15	0.045757	5.28553	0.693147
1	0	70.15	0.029251	5.41462	1.098612
1	0	68.57	0.016132	5.28608	1.386294
1	0	35.1986	0.007979	2.6719	1.609438
1	0	32.4686	0.006994	2.50862	1.791759
1	0	32.5386	0.005069	2.74794	1.94591
1	0	31.0086	-0.00418	2.39502	2.079442
1	0	32.7686	0.004459	2.41839	2.197225
1	0	32.7686	0.004544	2.34979	2.302585
1	2	27.4305	0.170912	10	0
1	2	20.5743	0.074773	8.17877	0
1	2	20.6143	0.086767	7.88547	0.693147
1	2	18.2687	0.063533	7.466	1.098612
1	2	14.3113	0.05524	7.06035	1.386294
1	2	15.5652	0.051476	6.51743	1.609438
1	2	20.1801	0.05843	6.33895	1.791759
1	2	21.5625	0.042782	6.27447	1.94591
1	2	21.5131	0.052532	5.83329	2.079442
1	2	21.6431	0.013134	5.66504	2.197225
1	2	21.9736	0.015236	5.23302	2.302585
1	1	63.64	0.045986	0.5216	0
1	1	63.64	0.057606	0.4232	0.693147
1	1	63.64	0.034653	0.39604	1.098612
1	1	54.81	0.008808	0.23741	1.386294
1	1	45.67	0.004938	0.48084	1.609438
1	1	41.16	0.006713	1.18961	1.791759
1	1	41.16	0.005526	0.07636	1.94591
1	1	41.16	-0.05385	0.41988	2.079442
1	1	41.16	0.010765	0.43994	2.197225

1	1	41.37	0.010675	0.03406	2.302585
1	0	68.024	0.073538	5.286	0
1	0	68.024	0.054123	5.44919	0.693147
1	0	49.5081	0.055743	3.72642	1.098612
1	0	45.2381	0.038395	4.24621	1.386294
1	0	43.4912	0.044979	4.58345	1.609438
1	0	39.4918	0.032198	3.65902	1.791759
1	0	28.0986	0.036039	3.62699	1.94591
1	0	39.8542	0.016498	3.59489	2.079442
0	1	61.5595	0.112618	0	0
0	1	46.14	0.029299	0.0741	0
0	1	46.14	0.040069	0.08484	0.693147
0	1	46.14	0.046032	0.04033	1.098612
0	1	46.14	0.03499	0.00112	1.386294
0	1	45.88	0.025027	0.30641	1.609438
0	1	42.9	0.028383	1.42979	1.791759
1	1	43.5	0.054131	0.01826	0
1	0	52.112	0.112198	5.366	0
1	0	38.702	0.066927	4.0233	0
1	0	38.702	0.108956	3.98845	0.693147
1	0	38.702	0.082823	4.07229	1.098612
1	0	38.702	0.063934	3.98618	1.386294
1	0	38.702	0.099217	4.27367	1.609438
1	0	38.6103	0.066842	4.62866	1.791759
1	0	38.6103	0.033695	5.77269	1.94591
1	0	37.0203	0.038184	4.32652	2.079442
0	1	35.25	0.047049	0.0331	0
0	1	35.25	0.063284	0.02888	0.693147
0	1	35.25	0.05718	0.01493	1.098612
0	1	35.25	0.052239	0	1.386294
0	1	29.52	0.047432	1.19557	1.609438
0	1	26.49	0.049785	0.9669	1.791759
0	1	22.78	0.064959	0.16829	1.94591
0	1	22.78	0.040893	0.01081	2.079442
0	1	27.28	0.023495	0.00023	2.197225
0	1	27.28	0.036171	0.00066	2.302585
1	1	43.9987	0.031954	7.5087	0
1	1	43.9987	0.030889	7.265	0.693147
1	1	43.9987	0.01988	7.29481	1.098612
1	1	35.3398	0.023265	7.15522	1.386294
1	1	35.3978	0.01926	7.00582	1.609438
1	1	45.35	0.024247	5.14023	1.791759
1	1	45.64	0.021781	4.80838	1.94591
1	1	42.317	0.016186	5.182	2.079442
1	1	42.317	0.022498	5.02485	2.197225
1	1	42.317	0.017139	4.81888	2.302585
0	1	54.0081	0.04861	5.84464	0
0	1	54.0081	0.050236	5.84744	0.693147
0	1	54.2081	-0.03144	5.85204	1.098612
0	1	54.2581	0.006834	5.85751	1.386294
0	2	48.73	0.04781	4.89402	1.609438
0	2	48.73	0.027409	5.94659	1.791759
0	2	45.86	0.040615	4.80142	1.94591
0	2	45.86	0.049299	4.8582	2.079442
0	2	28.2186	0.043106	4.89223	2.197225
1	1	50.48	0.051567	1.869	0
1	1	50.48	0.020934	1.6491	0.693147
1	1	40.61	0.036636	1.89094	1.098612

1	1	38.79	0.031748	1.78858	1.386294
1	1	28.6	0.012134	1.32556	1.609438
1	1	28.66	0.0325	1.47513	1.791759
1	1	23.68	0.02977	0.68305	1.94591
1	1	21.97	0.014518	0.73791	2.079442
2	0	56.57	0.058768	6.31437	0
2	0	56.57	0.069588	5.95633	0.693147
2	0	56.4	0.065798	6.18428	1.098612
2	0	55.84	0.072528	5.93319	1.386294
2	0	51.71	0.060259	4.52702	1.609438
2	0	52.12	0.01857	3.97271	2.079442
2	0	53.02	0.017851	3.80844	2.197225
2	0	53.02	0.020447	3.9906	2.302585
1	0	70.9888	0.020573	8.42385	1.609438
1	0	66.2982	-0.05114	7.61138	1.791759
1	0	66.2982	0.014266	7.08579	1.94591
1	0	66.2982	0.008752	7.0635	2.079442
1	0	66.0068	0.011988	7.05469	2.197225
1	0	60.3668	-0.07976	6.44794	2.302585
0	1	65.74	0.056586	0.07857	0
0	1	65.74	0.044176	0.11311	0.693147
0	1	65.74	0.030352	0.0355	1.098612
0	1	63.26	0.030189	0.02781	1.386294
0	1	63.26	0.038828	0.12965	1.609438
0	1	62.04	0.048685	0.70501	1.791759
0	1	51.89	0.038087	1.17209	1.94591
0	1	51.89	0.046567	0.79219	2.079442
0	1	51.89	0.047124	0.54744	2.197225
0	1	51.89	0.045487	0.56725	2.302585
0	2	30.77	0.036555	1.1498	0
0	2	30.77	0.03262	0.7598	0.693147
0	2	30.77	0.082582	0.22721	1.098612
0	2	30.77	0.1048	0.31981	1.386294
0	2	30.77	0.088678	0.76936	1.609438
0	2	30.9963	0.069799	1.1142	1.791759
0	2	33.7557	0.013515	2.20233	1.94591
0	2	33.7557	0.04995	2.04951	2.079442
0	2	33.7557	0.071864	1.96486	2.197225
0	2	34.231	0.105725	1.70729	2.302585
2	0	64.14	0.053456	1.64021	0
2	0	64.14	0.025634	0.50029	0.693147
2	0	64.35	-0.04178	0.20552	1.098612
2	0	64.35	0.007426	0.82295	1.386294
2	0	61.02	0.008689	1.17882	1.609438
2	0	55.29	0.005705	1.86606	1.791759
2	0	55.29	0.009851	1.94835	1.94591
2	0	43.76	0.023987	3.91693	2.079442
2	0	38.7	0.026049	3.24336	2.197225
2	0	32.09	0.022281	2.75032	2.302585
0	1	47.19	0.059696	0.6276	0
0	1	47.19	0.071189	0.15671	0.693147
0	1	46.9	0.034979	0.14968	1.098612
0	1	47.1	0.008098	0.00819	1.386294
0	1	43.41	0.005648	0.28793	1.609438
2	0	68.47	0.04134	6.395	0
2	0	68.47	0.041685	6.52402	0.693147
2	0	68.47	0.029602	6.48171	1.098612
2	0	65.21	0.027166	5.79663	1.386294

2	0	65.66	0.016366	5.69	1.609438
2	0	56.15	0.005724	4.82829	1.791759
2	0	56.99	0.008006	4.82583	1.94591
2	0	55.136	0.037614	5.25176	2.079442
2	0	55.136	0.01108	5.34923	2.197225
1	3	53.4662	0.018208	5.10689	2.302585
1	0	44.4	0.076446	0.31807	0
1	0	44.4	0.106	0.18725	0.693147
1	0	35.82	0.038086	1.41711	1.098612
1	0	35.82	0.020431	0.08619	1.386294
1	0	30.7	0.019249	0.31759	1.609438
1	0	42.09	0.03109	0.94902	1.791759
1	0	21.08	0.0355	2.46976	1.94591
1	0	34.45	0.051584	0.74886	0
1	0	34.45	0.050872	0.68537	0.693147
1	0	34.7	0.044114	0.67849	1.098612
1	0	31.49	0.047971	0.18544	1.386294
1	0	26.5	0.050352	0.30719	1.609438
1	0	24.63	-0.00974	0.05698	1.791759
1	0	24.63	0.030322	0.08668	1.94591
1	0	24.8	0.031201	0.05974	2.079442
1	0	24.8	0.064459	0.05913	2.197225
1	0	24.8	0.074649	0.09466	2.302585
1	0	21.1646	0.088597	7.579	0
1	0	22.8057	0.115529	7.4589	0.693147
1	0	22.8057	0.062933	7.3226	1.098612
1	0	23.2105	0.072431	5.40471	1.386294
1	0	23.2104	0.07344	4.73089	1.609438
1	0	23.9904	0.065732	6.02747	1.791759
1	0	24.1004	0.081176	6.06114	1.94591
1	0	23.2104	0.085018	5.0003	2.079442
1	0	23.2105	0.091919	5.09637	2.197225
1	0	16.5443	0.08661	4.74794	2.302585
1	0	44.3012	0.04707	7.6396	0
1	0	44.3012	0.041651	7.522	0.693147
1	0	44.3012	0.027882	7.48332	1.098612
1	0	44.3012	0.016253	7.2718	1.386294
1	0	44.3012	0.001559	7.2052	1.609438
1	0	44.3012	0.026264	7.60128	1.791759
1	0	44.3012	0.043682	7.23933	1.94591
1	0	44.3012	0.023591	6.5511	2.079442
1	0	44.3012	0.027555	6.5493	2.197225
1	0	44.3012	0.022803	6.61653	2.302585
1	1	52.544	0.032279	5.57719	0
1	1	52.544	0.034035	4.93402	0.693147
1	1	52.544	0.014763	4.28344	1.098612
1	1	52.544	0.020822	4.28364	1.386294
1	1	52.544	0.032697	4.34889	1.609438
1	1	52.544	0.041488	4.52981	1.791759
1	1	53.2745	0.03878	4.64656	1.94591
1	1	53.2745	0.037342	4.24425	2.079442
1	1	53.2745	0.028562	4.32456	2.197225
1	1	53.2745	0.024318	4.36568	2.302585
1	0	26.18	0.02897	2.50441	0
1	0	26.16	0.036273	1.85637	0.693147
1	0	26.2	0.01764	1.16647	1.098612
1	0	25.37	0.006051	0.57591	1.386294
1	0	19.32	0.002433	0.29866	1.609438

1	0	17.49	0.008261	1.27376	1.791759
1	0	16.74	0.090419	0.28595	1.94591
1	0	16.26	0.042765	0.21509	2.079442
1	0	14.9609	0.016142	1.01108	2.197225
1	0	16.5872	-0.04653	1.06353	2.302585
1	0	69.56	0.022605	4.67322	1.386294
1	0	69.56	0.026966	4.65795	1.609438
1	0	69.56	0.018994	4.79387	1.791759
1	0	66.85	0.017441	4.97717	1.94591
1	0	66.85	0.014166	4.47482	2.079442
1	0	66.85	0.029995	4.51214	2.197225
1	0	66.85	0.050929	5.05145	2.302585
1	0	25.81	0.075278	1.2689	0
1	0	25.81	0.091826	1.86994	0.693147
1	0	25.81	0.073248	2.32712	1.098612
1	0	25.81	0.060818	1.67636	1.386294
1	0	26.12	0.046441	0.8088	1.609438
1	0	26.33	0.023995	0.37699	1.791759
1	0	26.33	0.026669	0.31667	1.94591
1	0	26.33	0.028979	1.17853	2.079442
1	0	26.33	0.043169	1.41138	2.197225
1	0	26.33	0.032445	0.86405	2.302585
1	0	32.4211	0.05686	6.72126	0
1	0	32.4211	0.053198	6.52915	0.693147
1	0	23.3224	0.041557	6.40815	1.098612
1	0	23.3224	0.05372	6.00077	1.386294
1	0	19.5706	0.042814	5.38584	1.609438
1	0	22.6012	0.033325	4.67114	1.791759
1	0	22.6289	0.03676	4.54875	1.94591
1	0	22.6913	0.03609	4.09843	2.079442
1	0	22.7537	0.031721	4.03407	2.197225
1	0	30.06	0.011564	2.80704	2.302585
2	1	25.9315	0.03956	3.85614	0
2	1	26.37639	0.04192	3.7984	0.693147
2	1	26.37639	0.038005	3.679	1.098612
2	1	26.7064	0.042088	3.76328	1.386294
2	1	26.7064	0.035135	3.90183	1.609438
2	1	23.7064	0.029862	3.35867	1.791759
2	1	9.7759	0.050837	7.87312	1.94591
2	1	23.3212	0.048499	7.9535	2.079442
2	1	7.6038	0.047914	7.38691	2.197225
0	4	51.61	0.101789	0.28222	0
0	1	51.61	0.094971	0.55064	0.693147
0	1	51.77	0.063097	0.51366	1.098612
0	1	51.83	0.145021	0.94998	1.386294
0	1	46.89	0.177761	0.23996	1.609438
0	2	45.16	0.098762	1.16138	1.791759
0	2	45.39	0.085336	1.30044	1.94591
0	2	45.66	0.105046	0.99394	2.079442
0	2	44.79	0.127131	1.05609	2.197225
0	2	43.59	0.12318	1.1827	2.302585
2	0	38.87	0.063212	0.85182	0
2	0	38.87	0.070145	0.54692	0.693147
2	0	39.01	0.06666	0.38428	1.098612
2	0	39.01	0.053689	0.04086	1.386294
2	0	34.01	0.057133	0.72729	1.609438
2	0	33.8614	0.046844	0.66001	1.791759
2	0	29.321	0.03084	1.278	1.94591

2	0	29.101	0.037223	1.13413	2.079442
2	0	29.071	0.036633	0.9971	2.197225
2	0	29.091	0.040121	0.98137	2.302585
1	1	50.34	0.042034	0.01473	0
1	1	50.34	0.056101	0.05093	0.693147
1	1	50.34	0.015459	0.01925	1.098612
1	1	50.34	0.006032	0.02479	1.386294
1	1	48.34	0.004746	0.00003	1.609438
1	1	36.26	-0.02912	1.64503	1.791759
1	1	27.19	0.011786	2.79458	1.94591
1	1	20.4	0.016699	2.43711	2.079442
1	1	20.4	0.045557	3.21825	2.197225
1	1	20.4	0.007219	1.65307	2.302585
0	1	28.2123	0.039572	6.1054	0
0	1	28.2123	0.030151	5.67884	0.693147
0	1	28.2123	0.043127	5.33026	1.098612
0	1	23.2112	0.04183	5.12634	1.386294
0	1	21.7544	0.026979	3.87533	1.609438
0	1	8.6824	0.03301	3.08221	1.791759
0	1	8.6824	0.018964	2.39566	1.94591
0	2	63.67068	0.094241	5.35545	0
0	2	63.67068	0.086245	4.97964	0.693147
0	2	63.67068	0.074017	5.1938	1.098612
0	2	58.7463	0.092606	5.65342	1.386294
0	2	54.8963	0.089469	5.9811	1.609438
0	2	51.4963	0.087083	5.98865	1.791759
0	2	45.3862	0.089083	5.74135	1.94591
0	2	45.3862	0.070213	5.21057	2.079442
0	2	45.7714	0.079241	5.53243	2.197225
0	2	45.7714	0.081173	5.37138	2.302585
1	1	48.51	0.071884	0.1135	0
1	1	48.51	0.074849	0.13215	0.693147
1	1	62.06	0.090398	0.21588	1.098612
1	1	62.06	0.068119	0.35019	1.386294
1	1	52.51	0.071178	1.86018	1.609438
1	1	28.9187	0.075998	1.17231	1.791759
1	1	33.22	0.102104	0.5838	1.94591
1	1	33.26	0.130267	0.33455	2.079442
1	0	26.576	0.061209	7.68914	0
1	0	27.536	0.068756	6.71804	0.693147
1	0	22.904	0.073131	6.96535	1.098612
1	0	22.8	0.082834	6.26379	1.386294
1	0	18.816	0.091961	4.27229	1.609438
1	0	12.224	-0.12147	4.3877	1.791759
1	0	17.808	-0.10498	5.12364	1.94591
1	0	19.248	-0.12411	5.50587	2.079442
1	0	24.35	0.039523	0.54082	0
1	0	24.35	0.035297	0.57985	0.693147
1	0	24.43	0.038732	0.26089	1.098612
1	0	21.28	0.055322	1.0302	1.386294
1	0	21.18	0.062894	1.34226	1.609438
1	0	21.07	0.036849	0.49557	1.791759
1	0	17.96	0.042621	0.71617	1.94591
1	0	18.33	0.02165	0.14813	2.079442
1	0	19.72	-0.1007	0.11087	2.197225
1	0	19.72	0.04776	0.29029	2.302585
1	1	33.71	0.054963	1.58862	0
1	1	33.71	0.063964	1.19386	0.693147

1	1	32.85	0.064889	0.82497	1.098612
1	1	25.91	0.038723	1.84988	1.386294
1	1	27.15	0.043183	0.85983	1.609438
1	0	23.79	0.049375	0.5057	1.791759
1	0	23.89	0.122197	0.32434	1.94591
2	1	22.11	0.090519	1.93131	2.197225
2	1	22.5	0.024864	2.7419	2.302585
1	0	34.096	0.081317	8.61137	0
1	0	34.096	0.077381	8.73406	0.693147
1	0	34.096	0.06327	8.56163	1.098612
1	0	32.4276	0.037442	7.04312	1.386294
2	0	21.0089	0.033963	4.84864	2.079442
2	0	20.4561	0.086457	5.94969	2.197225
2	0	20.3885	0.146424	5.11413	2.302585
1	1	51.9226	0.083671	7.4221	0
1	1	51.9226	0.061927	7.36847	0.693147
1	1	51.9226	0.057515	7.52642	1.098612
1	1	48.1454	0.037473	7.49652	1.386294
0	1	62.8575	0.037046	6.54479	0
0	1	62.8575	0.035828	6.63012	0.693147
0	1	62.8575	0.026605	6.49275	1.098612
0	1	62.8575	-0.07883	6.54169	1.386294
0	2	41.3929	0.045936	7.78356	1.609438
0	2	40.4982	0.056654	5.96115	1.791759
0	2	40.4687	0.15943	4.3538	1.94591
0	2	40.4687	0.146393	5.39181	2.079442
0	2	40.518	0.06283	4.9748	2.197225
0	2	40.528	-0.11766	5.28482	2.302585
1	0	20	0.012436	3.73108	0
0	3	40.5583	0.087382	1.92746	0
0	3	40.5583	0.090076	1.87567	0.693147
0	3	40.7983	0.061652	2.01324	1.098612
0	3	40.7983	0.030899	0.97923	1.386294
0	3	37.7248	0.050984	1.46956	1.609438
0	3	37.7355	0.035927	1.24529	1.791759
0	3	31.7903	0.047566	1.55497	1.94591
0	3	30.9015	0.032104	1.18264	2.079442
0	3	30.7721	0.02461	0.80322	2.197225
0	3	30.7721	0.009193	0.93125	2.302585
0	1	52.2631	0.071258	7.51467	0
0	1	52.2631	0.04043	7.50667	0.693147
0	1	56.0131	0.043514	6.957	1.098612
0	1	56.0131	0.006414	7.2381	1.386294
0	1	56.0131	-0.02278	7.19323	1.609438
2	1	16.3078	0.070343	7.88759	0
2	1	16.3068	0.090426	7.3891	0.693147
2	1	16.1316	0.088698	7.25974	1.098612
2	1	15.7332	0.05048	6.76529	1.386294
2	1	15.8003	0.05729	6.21853	1.609438
2	1	15.9387	0.04673	5.82693	1.791759
2	1	15.9387	0.059839	5.33613	1.94591
2	1	15.9387	0.080273	5.33993	2.079442
2	1	14.1981	0.029883	5.12875	2.197225
2	1	10.0076	0.037329	4.97401	2.302585
3	0	64.644	0.060211	6.91362	0
3	0	64.644	0.052517	7.28076	0.693147
2	0	51.6	0.053294	7.45799	1.098612
2	0	51.082	0.05977	6.95619	1.386294

2	0	51.082	0.048527	5.39397	1.609438
2	0	42.24	0.022762	6.25563	1.791759
2	0	50.56	0.025272	6.35698	1.94591
2	0	52.2	0.041733	6.26933	0
2	0	52.2	0.015177	6.29211	0.693147
2	0	52.2	0.005425	6.04751	1.098612
1	0	11.9512	0.0457	4.58309	0
1	0	11.2244	0.053008	4.19315	0.693147
1	0	11.2175	0.063558	4.18196	1.098612
1	0	11.1998	0.059143	3.94153	1.386294
1	0	26.25	0.045938	1.09459	0
1	0	26.25	0.043409	0.7815	0.693147
1	0	26.25	0.028847	0.66832	1.098612
1	0	26.25	0.017448	0.46417	1.386294
1	0	23.23	0.017637	2.02554	1.609438
1	0	23.23	0.011715	2.40351	1.791759
1	0	38.15	-0.03299	0.75508	1.94591
1	0	28.79	0.007535	2.63386	2.079442
1	0	33.8	0.022288	2.64035	2.197225
1	0	33.76	0.002996	1.42248	2.302585
3	0	64.3801	0.060177	4.71786	0
3	0	64.38	0.067486	4.73568	0.693147
3	0	64.5399	0.044456	4.5699	1.098612
3	0	65.1	0.053451	4.59799	1.386294
3	0	55.78	0.057224	4.39862	1.609438
3	0	55.89	0.086278	3.88026	1.791759
3	0	56.3799	0.006633	4.0359	1.94591
3	0	56.38	0.00829	4.36883	2.079442
3	0	57.33	-0.01282	3.87874	2.197225
3	0	55.97	0.0727	4.1067	2.302585
2	0	35.03	0.060392	3.45768	0
2	0	35.03	0.036051	3.27473	0.693147
2	0	35.03	0.026518	3.22553	1.098612
2	0	35.03	-0.03066	3.21496	1.386294
2	0	33.29	0.002359	3.22337	1.609438
2	0	33.29	-0.04896	3.43232	1.791759
2	0	33.29	-0.11273	3.37707	1.94591
2	0	33.56	0.015498	3.31	2.197225
2	0	29.53	-0.03921	3.30904	2.302585
1	1	50.25	0.046949	7.81742	0
1	1	50.25	0.056315	7.28847	0.693147
1	1	50.25	0.063741	6.38878	1.098612
1	1	49	0.044468	5.48942	1.386294
1	1	46.5	0.035951	5.01982	1.609438
1	1	43.34	0.021131	5.78852	1.791759
1	1	43.34	0.027723	5.73294	1.94591
1	1	37.3176	0.038166	5.1901	2.079442
1	1	42.6	0.033711	4.89724	2.197225
1	1	42.6	0.030092	4.59693	2.302585
0	1	48.557	0.175552	7.77995	0
0	1	48.557	0.127311	7.74179	0.693147
0	1	57.127	0.016207	7.68552	1.098612
0	1	57.127	0.010644	7.66254	1.386294
0	1	57.127	-0.01956	7.64917	1.609438
0	1	57.4128	0.007702	7.85499	1.791759
0	1	52.1848	0.048154	7.9045	1.94591
0	1	52.1848	0.033367	7.75826	2.079442
0	1	47.9438	0.016206	7.64252	2.197225

0	1	48.0507	0.016193	7.62267	2.302585
1	0	42.29	0.060014	1.92434	0.693147
1	0	42.29	0.064819	1.24521	1.098612
1	0	42.29	0.057146	0.54865	1.386294
1	0	35.81	0.044864	1.36984	1.609438
1	0	33.58	0.005749	4.24787	1.791759
1	0	31.31	0.003994	2.27699	1.94591
1	0	31.31	-0.02958	0.64276	2.079442
1	0	31.31	0.005147	0.37281	2.197225
1	0	31.31	0.004782	0.2509	2.302585
3	0	62.5	0.054735	1.0959	0
3	0	62.5	0.053386	0.74163	0.693147
3	0	61.99	0.043812	0.42866	1.098612
3	0	62.2	0.03157	0.00015	1.386294
3	0	53.86	0.032798	0.0925	1.609438
3	0	46.48	0.033705	0.35019	1.791759
3	0	45.31	0.027496	0.33772	1.94591
3	0	46.01	0.017484	0.30194	2.079442
3	0	44.24	0.027325	0.17702	2.197225
3	0	43.26	0.022055	0.00401	2.302585
0	3	53.94	0.054114	1.69985	0
0	3	53.94	0.02829	1.0684	0.693147
0	3	52.81	0.042921	0.56768	1.098612
0	3	52.83	0.038061	1.57317	1.386294
0	3	52.83	0.038465	1.87117	1.609438
0	3	53.03	0.022483	0.36822	1.791759
0	3	42.2	0.021071	0.28802	1.94591
0	3	42.2	0.053614	0.67814	2.079442
0	3	47.05	-0.10827	0.203	2.302585
0	1	21.51	0.092264	0.47898	0
0	1	21.51	0.098337	0.31948	0.693147
0	1	21.51	0.088687	0.54099	1.098612
0	1	21.51	0.087741	0.13547	1.386294
0	1	18.39	0.085518	1.03778	1.609438
0	1	14.7	0.044171	0.36338	1.791759
0	1	14.7	0.045592	0.48022	1.94591
0	1	14.32	0.061771	0.63073	2.079442
0	1	14.32	0.048278	0.29449	2.197225
0	1	14.34	0.014705	0.37014	2.302585
1	0	30.191	0.097518	9.21382	0
1	0	30.191	0.063535	9.15084	0.693147
1	0	29.7498	0.053962	8.8463	1.098612
1	0	29.7498	0.086412	8.82164	1.386294
1	0	24.2514	0.04041	8.81025	1.609438
1	0	20.7347	0.056183	7.03994	1.791759
1	0	19.8929	0.064747	7.03758	1.94591
1	0	19.8929	0.040279	6.98163	2.079442
1	0	19.8929	0.033276	6.99788	2.197225
1	0	19.8929	0.068508	7.04766	2.302585
2	0	38.06	0.052255	3.41275	0
2	0	38.06	0.063518	3.10103	0.693147
2	0	38.06	0.056125	2.23744	1.098612
2	0	27.41	0.063352	3.50717	1.386294
2	0	27.41	0.059367	1.48759	1.609438
2	0	27.94	0.054206	0.50186	1.791759
2	0	28.42	0.045517	0.79478	1.94591
2	0	22.26	0.03436	2.54457	2.079442
0	1	37.38	0.049125	1.12083	0

0	1	37.38	-0.0083	0.89692	0.693147
1	0	40.5862	0.079756	7.38053	0
1	0	40.5862	0.097422	7.54788	0.693147
1	0	40.5862	0.112481	7.15728	1.098612
1	0	40.1226	0.13474	8.15971	1.386294
1	0	39.7629	0.156569	7.34828	1.609438
1	0	39.2665	0.164712	6.80522	1.791759
1	0	38.718	0.188118	6.82477	1.94591
1	0	38.5719	0.18434	7.40578	2.079442
1	0	38.5719	0.156932	7.04423	2.197225
1	0	38.5719	0.151545	7.78159	2.302585
2	0	52.867	0.036951	7.74682	0
2	0	53.0181	0.028606	7.81156	0.693147
2	0	53.0329	0.046377	8.09475	1.098612
2	0	53.2208	0.025809	6.77539	1.386294
2	0	51.8865	0.014836	6.16882	1.609438
2	0	52.3891	0.019408	6.81446	1.791759
2	0	50.6649	0.057711	9.60021	1.94591
2	0	50.6933	0.060268	7.03011	2.079442
2	0	50.8714	0.079091	7.19907	2.197225
2	0	50.0087	0.1002	6.97346	2.302585
0	1	54.87	0.0678	2.31702	0
0	1	54.87	0.067292	1.80227	0.693147
0	1	54.87	0.069053	0.66743	1.098612
0	1	56.13	0.052272	0.46609	1.386294
0	1	56.45	0.049572	1.94206	1.609438
0	1	53.28	0.038582	1.01473	1.791759
0	1	48.56	0.042925	1.58787	1.94591
0	1	48.6	0.076627	1.4867	2.079442
0	1	49.6	0.052234	1.18698	2.197225
0	1	50.665	0.04023	1.0465	2.302585
1	0	29.41	-0.06371	1.018	1.609438
1	0	47.8131	0.055457	5.52508	0
1	0	35.1401	0.103407	5.36573	0.693147
1	0	35.4468	0.070906	5.10613	1.098612
1	0	25.8725	0.007577	5.63774	1.386294
1	0	35.4418	0.037479	6.76856	1.609438
1	0	31.8244	0.018698	5.74219	1.791759
1	0	31.8556	0.022443	5.53017	1.94591
1	0	32.4085	0.022932	5.20636	2.079442
1	0	32.2571	0.032693	5.02116	2.197225
1	0	34.3247	0.030374	6.30744	2.302585
2	0	31.19	0.057417	3.3096	0
2	0	31.19	0.030543	3.49303	0.693147
2	0	31.19	0.049742	1.92936	1.098612
2	0	30.58	0.054114	1.128	1.386294
2	0	25.59	0.067752	1.05126	1.609438
2	0	16.31	0.065921	0.58883	1.791759
2	0	16.31	0.033606	0.79799	1.94591
2	0	16.31	0.035985	1.54918	2.079442
2	0	16.31	0.013385	0.95263	2.197225
2	0	16.31	0.030565	0.8059	2.302585
1	1	56.6751	0.067399	1.70955	0
1	1	56.6751	0.083495	1.29795	0.693147
1	1	56.6751	0.077305	1.23907	1.098612
1	1	54.8	0.056378	0.61564	1.386294
1	1	46.42	0.034239	1.12062	1.609438
1	1	46.42	0.035133	1.19997	1.791759

1	1	45.07	0.045084	0.95501	1.94591
1	1	43.95	0.008576	0.30468	2.079442
1	1	41.04	-0.00707	0.03972	2.197225
1	1	39.05	-0.00384	0.141	2.302585
1	0	23.8551	0.048582	2.33238	0
1	0	23.9651	0.051346	2.36317	0.693147
1	0	23.97	0.014545	0.03961	1.098612
1	0	23.97	0.013585	0.00805	1.386294
1	0	23.97	0.004397	0.00263	1.609438
1	0	24.02	-0.09644	0.17141	1.791759
1	0	24.02	0.003825	0.31664	1.94591
1	0	20.86	0.026449	0.01267	2.079442
1	0	20.79	0.054323	0.05342	2.197225
1	0	20.79	0.055746	0.05209	2.302585
0	2	39.07	0.061968	0.35825	0
0	2	39.07	0.086652	0.64976	0.693147
0	2	37.8	0.086879	0.4989	1.098612
0	2	37.76	0.098502	0.18874	1.386294
0	2	33.75	0.082125	0.24264	1.609438
0	2	31.54	0.056126	0.28354	1.791759
0	2	29.98	0.038217	0.154	1.94591
0	2	29.98	0.021988	0.10476	2.079442
0	2	29.98	-0.04456	0.0001	2.197225
0	2	29.98	0.009526	0.00612	2.302585
1	2	34.4448	0.044926	7.56269	0
1	2	34.4448	0.038729	7.63691	0.693147
1	2	34.4448	0.006525	6.94165	1.098612
1	2	34.4448	0.007748	5.579	1.386294
1	2	34.4448	0.00285	5.19705	1.609438
1	2	34.4448	0.002299	5.27423	1.791759
1	2	33.4242	-0.02012	6.95387	1.94591
1	2	27.7512	0.003691	5.27283	2.079442
1	2	25.6041	-0.09113	4.5533	2.197225
2	0	39.52	0.046697	2.86698	1.94591
2	0	25.4344	0.06726	1.91192	2.079442
2	0	26.0718	0.066514	1.95459	2.197225
2	0	26.0718	0.067869	1.90806	2.302585
1	0	65.03	0.083583	0.911	0
1	0	48.23	0.064309	1.48601	0
1	0	48.24	0.052326	1.02694	0.693147
1	0	48.4	0.04143	1.13763	1.098612
1	0	48.61	0.00831	0.05471	1.386294
1	0	44.99	-0.04382	0.68542	1.609438
1	0	38.43	0.002184	0.69477	1.791759
2	0	29.78	0.003229	0.2504	1.94591
1	0	38.61	-0.01684	0.07297	2.079442
1	0	34.5437	0.081927	5.757	0
1	0	25.9138	0.040515	4.46852	0
1	0	32.3735	0.017867	4.39982	0.693147
1	0	32.3735	0.011624	4.34576	1.098612
1	0	27.5374	0.016324	4.31823	1.386294
1	0	24.3663	0.019454	5.12705	1.609438
1	0	20.4919	0.037923	3.83824	1.791759
1	0	21.3021	0.076532	4.91177	1.94591
1	0	22.1973	0.065068	4.35878	2.079442
1	0	21.9445	0.018134	3.91911	2.197225
2	0	55	0.09679	10	0
2	0	41.25	0.047344	7.574	0

2	0	41.25	0.037285	7.51102	0.693147
2	0	40.58	0.029684	7.39217	1.098612
2	0	40.88	0.018988	7.44917	1.386294
2	0	36.48	0.032794	7.6909	1.609438
2	0	26.4065	0.018306	8.27764	1.791759
2	0	26.4065	0.023444	7.58994	1.94591
2	0	26.4864	0.014708	7.36765	2.079442
1	1	26.6913	0.109746	8.35	0
1	1	19.8394	0.080319	6.6487	0
1	1	19.8394	0.078069	6.7555	0.693147
1	1	19.8394	0.041704	6.26235	1.098612
1	1	17.0007	0.042631	4.9173	1.386294
1	1	14.0135	0.060085	5.55167	1.609438
1	0	64.5	0.055574	0.81814	0
1	0	64.5	0.028366	0.84042	0.693147
1	0	64.5	0.02532	0.42046	1.098612
1	0	64.5	0.028015	1.35479	1.386294
1	0	58.07	0.027789	2.02692	1.609438
1	0	51.14	0.017117	2.09761	1.791759
1	0	51.14	0.011029	1.8758	1.94591
2	1	51.37	0.016218	1.58846	2.079442
2	1	44.44	0.022503	1.72006	2.197225
2	1	63.7525	0.077038	3.98796	0
2	1	63.7525	0.070591	4.00161	0.693147
2	1	63.7525	0.06975	3.95768	1.098612
2	1	63.7525	0.071052	3.99042	1.386294
2	1	63.9125	0.074961	3.97031	1.609438
2	1	63.9125	0.084534	4.29206	1.791759
2	1	63.9125	0.064564	4.01484	1.94591
2	1	32.5125	0.07277	4.25858	2.079442
2	1	32.5125	0.087413	3.95963	2.197225
1	0	22.8	0.081937	0.1115	0
1	0	22.8	0.082352	0.00135	0.693147
1	0	22.8	0.08793	0.60566	1.098612
1	0	22.8	0.104796	1.06435	1.386294
1	0	22.8	0.09258	0.75036	1.609438
1	0	22.8	0.074058	0.60563	1.791759
1	0	19.95	0.057455	0.00952	1.94591
1	0	19.95	0.066401	0.03772	2.079442
1	0	19.95	0.087556	0.02948	2.197225
2	0	54.61	0.033358	2.43489	0
1	0	42.38	0.039151	0.90462	0.693147
1	0	42.38	0.038375	2.09843	1.098612
1	0	40.78	0.047106	1.50129	1.386294
1	0	40.78	0.04849	1.5527	1.609438
1	0	41.91	0.033592	1.25531	1.791759
1	0	39.58	0.043888	0.66492	1.94591
1	0	39.58	0.032821	0.67612	2.079442
1	0	39.58	0.016192	0.66333	2.197225
0	2	41.02	0.07707	3.16802	2.197225
0	2	58.4649	0.066425	0.44101	0
0	2	58.4649	0.065287	0.4881	0.693147
0	2	58.4649	0.072745	0.68566	1.098612
0	2	49.37	0.096795	0.11171	1.386294
0	2	49.37	0.091892	0.08337	1.609438
0	2	49.37	0.096009	0.95871	1.791759
0	2	49.37	0.100415	0.16078	1.94591
0	2	49.37	0.112279	0.10115	2.079442

0	2	49.37	0.053708	0.10448	2.197225
1	1	56.045	0.048419	2.1869	0
1	1	56.04	0.015684	1.93031	0.693147
1	1	56.04	0.004595	2.15973	1.098612
1	1	30.65	0.01716	0.88491	1.386294
1	1	30.69	0.199367	0.29597	1.609438
1	1	25.86	0.190515	0.86541	1.791759
1	1	25.09	0.200488	1.16051	1.94591
1	1	21.04	0.137144	1.35025	2.079442
0	1	34.1166	0.071853	8.55305	0
0	1	28.2116	0.089599	8.54838	0.693147
0	1	29.8246	0.066251	8.56773	1.098612
0	1	44.1728	0.068275	7.54338	1.386294
0	1	36.8274	0.079714	7.65186	1.609438
0	1	41.5107	0.085077	7.18236	1.791759
0	1	41.3109	0.068827	7.42906	1.94591
0	1	40.1117	0.086107	7.60687	2.079442
0	1	36.6381	0.111575	7.41296	2.197225
0	1	35.8279	0.129837	8.10513	0
0	1	35.8279	0.106766	8.60135	0.693147
0	1	35.8279	0.108691	8.4344	1.098612
0	1	35.8279	0.085937	7.99122	1.386294
1	1	67.09	0.092289	1.98444	2.197225
1	0	69.98	0.050999	2.572	0
1	0	52.29	0.034912	2.83034	0
1	0	52.39	0.021386	1.56824	0.693147
1	0	43.04	0.020868	1.97032	1.098612
1	0	43.04	0.009498	0.6205	1.386294
1	0	38.57	0.001543	0.22621	1.609438
1	0	29.73	-0.01799	1.02686	1.791759
2	0	24.93	0.01419	1.77442	1.94591
2	0	38.6	-0.06321	2.39146	2.197225
1	0	14.8657	0.061553	7.7993	0
1	0	14.9496	0.073994	6.9115	0.693147
1	0	14.71	0.046798	7.18715	1.098612
1	0	14.71	0.037171	6.9148	1.386294
1	0	14.1892	0.037655	6.19115	1.609438
1	0	15.834	0.054301	6.91117	1.791759
1	0	19.2377	0.063935	5.5251	1.94591
1	0	14.4317	0.02986	5.66267	2.079442
1	0	13.6267	0.039581	4.76118	2.197225
2	0	58.4841	0.061097	7.05036	0
2	0	58.4841	0.045376	7.13782	0.693147
2	0	58.5574	0.006441	7.034	1.098612
2	0	58.5574	-0.12665	7.60494	1.386294
1	0	28.373	0.010402	4.74124	0
0	1	29.4515	0.052764	5.39323	2.079442
1	2	65.5624	0.068137	7.8615	0
1	2	65.7823	0.0171	7.11043	0.693147
1	2	65.7823	0.017157	7.49662	1.098612
1	2	51.76	0.039864	5.24065	1.386294
1	2	51.76	0.01147	5.93527	1.609438
1	2	51.76	0.00862	5.69077	1.791759
1	2	52.89	0.022397	5.34448	1.94591
1	0	52.89	0.02264	5.31517	2.079442
1	0	52.89	0.012621	5.28961	2.197225
1	0	35.82	0.055905	1.3507	0
1	0	35.82	0.017326	0.10405	0.693147

1	0	35.82	0.009204	0.14324	1.098612
1	0	32.01	-0.01455	1.5642	1.386294
1	0	32.01	0.007773	1.01678	1.609438
1	0	24.01	-0.00571	0.94542	1.791759
1	0	29.98	-0.0011	3.90466	0
1	0	30	0.013617	3.09911	0.693147
1	0	30.27	0.023698	3.04199	1.098612
2	0	42.73	0.055696	0.698	0
2	0	42.73	0.034972	0.7201	0.693147
2	0	42.73	0.037682	0.69809	1.098612
2	0	32.05	0.026758	1.25939	1.386294
2	0	32.05	0.014385	0.10424	1.609438
2	0	27.86	0.009862	1.02886	1.791759
2	0	27.86	0.022088	0.14061	1.94591
2	0	53.86	0.019798	0.17612	2.079442
2	0	53.86	0.027184	0.68854	2.197225
2	0	61	0.072536	1.47985	0
2	0	61	0.046248	0.87739	0.693147
2	0	61	0.030361	0.68561	1.098612
2	0	61	0.049023	1.11929	1.386294
2	0	53.58	0.040436	2.70016	1.609438
2	0	46.13	0.038211	1.89159	1.791759
2	0	46.13	0.034221	1.79845	1.94591
2	0	44.12	0.018029	1.33334	2.079442
2	0	43.23	0.00823	0.63507	2.197225
1	0	61.3498	0.09209	3.80093	0
1	0	61.3498	0.029337	3.4376	0.693147
1	0	61.5998	0.021515	3.24032	1.098612
1	1	40.9848	0.017852	3.69734	1.386294
1	1	42.5948	0.020736	3.10313	1.609438
1	1	43.2648	0.01989	2.22788	1.791759
1	1	33.915	0.008249	2.57571	1.94591
1	1	33.915	0.034544	1.96654	2.079442
1	1	32.665	0.019286	1.70569	2.197225
1	0	40.5693	0.044445	5.49125	0
1	0	40.5693	0.042925	5.72489	0.693147
1	0	40.5693	0.039377	5.69565	1.098612
1	0	30.5715	0.03241	4.99534	1.386294
1	0	30.5715	0.014433	4.44216	1.609438
1	0	30.5715	0.016223	5.57468	1.791759
1	0	49.2264	0.0251	5.91711	1.94591
1	0	27.1588	0.023148	5.50342	2.079442
1	0	53.36	0.069923	0.51191	0
1	0	53.36	0.071802	1.41041	0.693147
1	0	53.36	0.092508	0.52925	1.098612
1	0	52.12	0.097223	1.45798	1.386294
1	0	51.05	0.098497	1.28005	1.609438
1	0	51.14	0.073516	0.57238	1.791759
1	0	51.49	0.03604	0.36418	1.94591
1	0	51.5	0.025876	0.51231	2.079442
1	0	51.5	0.015746	0.13509	2.197225
2	0	53.3902	0.027317	5.3086	0
2	0	53.3902	0.007229	5.25064	0.693147
2	0	53.3902	0.019025	5.32291	1.098612
2	0	53.8423	0.040822	5.34504	1.386294
2	0	51.1968	0.046545	6.16228	1.609438
2	0	42.1163	0.04156	5.49674	1.791759
2	0	42.1163	0.037952	4.61166	1.94591

2	0	51.8904	-0.01005	4.64528	2.079442
2	0	44.4093	0.035652	4.69568	2.197225
2	0	46.93	-0.07329	0.20253	1.609438
2	0	42.7	0.007957	1.2727	1.791759
2	0	36.28	-0.00094	1.06611	1.94591
2	0	36.26	0.021006	0.70739	2.079442
2	0	36.26	0.052654	0.23362	2.197225
2	0	37.81	0.101885	0.59247	0
2	0	37.81	0.101745	0.344	0.693147
2	0	37.81	0.097725	0.35184	1.098612
2	0	41.36	0.057652	0.24404	1.386294
2	0	41.52	0.049195	0.09741	1.609438
2	0	44.63	0.051979	0.51323	1.791759
2	0	42.16	0.052449	0.95475	1.94591
2	0	42.16	0.003007	0.32713	2.079442
2	0	59.15	0.040189	0.2305	0
2	0	59.15	0.02084	0.17767	0.693147
2	0	59.15	0.013835	0.11468	1.098612
2	0	59.15	0.013979	0.40406	1.386294
2	0	53.8	0.011786	1.39802	1.609438
2	0	52.94	0.019598	0.65105	1.791759
2	0	52.79	0.017561	0.00121	1.94591
2	0	57.98	0.020304	0.00773	2.079442
2	0	58	0.020323	0.00061	2.197225
1	0	28.5239	0.073913	7.39101	0
1	0	28.6094	0.052093	7.58335	0.693147
1	0	28.6901	0.050815	7.02838	1.098612
1	0	13.0546	0.04092	6.05944	1.386294
1	0	24.9172	0.050535	5.50135	1.609438
1	0	25.3503	0.131172	6.22623	1.791759
1	0	22.0498	0.160077	5.20598	2.079442
1	0	22.0598	0.131454	6.22465	2.197225
2	0	28.98	0.040157	0.705	0
2	0	28.98	0.035105	0.6306	0.693147
2	0	28.98	0.019329	0	1.098612
2	0	28.98	0.010549	0.60893	1.386294
2	0	27.03	0.015709	1.37387	1.609438
2	0	27.03	0.026291	1.73552	1.791759
2	0	24.15	0.039426	0.88033	1.94591
2	0	24.1	0.05175	0.75118	2.079442
2	0	24.22	0.05566	0.65458	2.197225
1	0	52	0.082253	7.446	0
1	0	52	0.049299	7.46193	0.693147
1	0	52	0.039989	7.16368	1.098612
1	0	26.58	0.050281	4.29068	1.386294
1	0	26.58	0.080885	4.28961	1.609438
1	0	28.75	0.092153	4.31569	1.791759
2	0	28.75	0.12234	4.62487	1.94591
2	0	24.31	0.05742	5.38563	2.079442
2	0	22.026	0.084535	4.59743	2.197225
1	1	58.77	0.107462	4.24001	0
1	1	58.77	0.044604	3.99891	0.693147
1	1	57.59	0.052445	3.8456	1.098612
1	1	57.65	0.069674	3.54364	1.386294
1	1	57.81	0.074787	3.56347	1.609438
1	1	49.77	0.085742	3.65007	1.791759
1	1	54.11	0.068056	3.90443	1.94591
1	1	46.63	0.069293	3.68389	2.079442

1	1	54.21	0.066492	3.68205	2.197225
2	1	70.25	0.081648	0.0571	0
2	1	70.269	0.099556	0.04306	0.693147
2	1	70.269	0.106853	0.52078	1.098612
2	1	70.27	0.084908	0.16789	1.386294
2	1	70.27	0.06199	0.31837	1.609438
2	1	61.9	0.050209	0.90444	1.791759
2	1	59.9	0.041422	0.56806	1.94591
2	1	57.8	0.072501	0.3448	2.079442
2	1	53.87	0.146606	0.52487	2.197225
0	1	42	0.041635	0.1374	0
0	1	42	0.027827	0.0522	0.693147
0	1	42	0.02201	0.08961	1.098612
0	1	32	0.018758	1.10517	1.386294
0	1	27.6	0.026432	1.22146	1.609438
0	2	21.06	0.068549	2.95357	0
0	2	21.06	0.041379	3.29147	0.693147
1	2	23.03	-0.01534	2.47105	1.098612
1	1	31.6451	0.054477	4.64187	0
1	1	32.0303	0.032382	3.128	0.693147
1	1	32.608	0.015353	2.56868	1.098612
1	1	32.5745	-0.0173	3.11278	1.386294
1	1	32.1726	0.003838	3.15258	1.609438
1	1	25.1922	0.015105	3.75854	1.791759
1	1	25.1922	0.01526	2.85521	1.94591
1	1	25.1922	0.160077	2.6352	2.079442
1	1	61.5	0.028061	0.69332	2.079442
1	1	60.07	0.007686	1.08188	2.197225
2	0	60.56	0.036953	4.6424	0
2	0	60.56	0.021336	4.41649	0.693147
2	0	60.56	0.017656	4.36642	1.098612
2	0	60.56	-0.01894	4.35314	1.386294
2	0	60.83	0.046681	4.8122	1.609438
2	0	52.33	0.018308	5.41579	1.791759
2	0	52.33	0.050103	4.54806	1.94591
2	0	25.23	0.004236	5.00116	2.197225
2	0	40.711	0.06227	4.3833	0
2	0	40.711	0.027078	3.7917	0.693147
2	0	40.711	0.031996	3.27272	1.098612
2	0	40.711	0.006898	3.9073	1.386294
2	0	33.94	0.043182	6.48371	1.609438
2	0	29.59	0.05526	6.31716	1.791759
2	0	21.71	0.059733	6.05594	1.94591
2	0	19.9	0.093784	5.99913	2.079442
1	0	52.49	0.089162	0.5017	0
1	0	52.49	0.055533	0.2329	0.693147
1	0	52.49	0.046502	0.00593	1.098612
1	0	52.49	0.053083	0.4466	1.386294
1	0	52.49	0.047724	0.41869	1.609438
1	1	36.657	0.07673	1.10267	0
1	1	36.657	0.046257	0.37349	0.693147
1	1	36.657	0.031136	0.37108	1.098612
1	1	36.657	0.033627	0.36215	1.386294
1	1	27.49	0.066854	1.04482	1.609438
1	0	56.26	0.077281	0.5401	0
1	0	56.26	0.041042	0.0024	0.693147
1	0	56.26	0.022002	0	1.098612
0	1	48.75	0.097506	1.046	0

0	1	48.75	0.072274	0.39646	0.693147
0	1	48.75	0.059769	0.00929	1.098612
0	1	43.52	0.061548	0.3482	1.386294
0	1	32.64	0.05732	0.25472	1.609438
0	1	32.64	0.089136	0.1296	1.791759
1	0	50.1605	0.075021	6.00164	0
1	0	50.1605	0.039139	6.23711	0.693147
1	0	50.1605	0.017278	5.53342	1.098612
1	0	50.2166	0.02592	5.27979	1.386294
1	0	50.27	0.028758	4.79921	1.609438
1	0	50.27	0.027582	4.54209	1.791759
1	0	48.84	0.054201	4.42307	1.94591
2	0	47.49	0.089647	4.37921	2.079442
2	0	49.43	0.068592	4.34042	2.197225
1	0	55.05	0.042337	1.175	0
1	0	55.05	0.006869	0.6186	0.693147
1	0	55.05	-0.03035	0.6832	1.098612
1	0	55.05	-0.04017	0.035	1.386294
1	0	41.28	-0.04234	2.14005	1.791759
1	0	41.28	0.008828	2.145	1.94591
2	0	41.28	0.006555	1.757	2.079442
2	0	41.28	-0.04714	1.72917	2.197225
0	1	24.9719	0.05831	2.98637	0.693147
0	1	26.0068	0.062731	5.55391	2.079442
0	1	28.3618	0.071578	5.1054	2.197225
1	1	64.6	0.040353	0.78592	0
1	1	64.6	0.011742	0.21861	0.693147
1	1	64.6	0.039771	0.06017	1.098612
1	1	64.6	-0.02209	0.06017	1.386294
1	1	56.73	0.002933	0.0811	1.609438
2	0	42.21	0.07566	7.04616	0
2	0	42.21	0.094191	7.61428	0
2	0	42.21	0.076339	6.74114	0.693147
2	0	42.21	0.057828	5.98705	1.098612
2	0	42.21	0.054623	5.05152	1.386294
2	0	42.3	0.052655	2.4027	1.609438
2	0	42.3	0.055377	2.33357	1.791759
2	0	42.3	0.044009	1.93568	1.94591
2	0	42.3	0.059626	1.96067	2.079442
0	1	71.22	0.174924	0.197	0
0	1	71.22	0.111094	0.12835	0
0	1	71.22	0.123278	0.10161	0.693147
0	1	71.22	0.112036	0.37321	1.098612
0	1	67.24	0.057991	0.08422	1.386294
0	1	67.24	0.038589	0.02062	1.609438
0	1	64.26	0.052278	0.13746	1.791759
0	1	63.08	0.047421	0.15139	1.94591
0	1	63.08	0.06693	0.1325	2.079442
0	2	56.5516	0.12674	2.443	0
0	2	42.4197	0.059029	1.69689	0
0	2	42.4197	0.055636	1.51408	0.693147
0	2	42.4197	0.04013	1.17898	1.098612
0	2	39.1355	0.027147	1.04744	1.386294
0	2	35.4694	0.044258	0.63012	1.609438
0	2	33.6829	0.032739	1.06023	1.791759
1	0	23.1133	0.094144	7.09641	0
1	0	46.8266	0.087956	6.38954	0.693147
1	0	45.2856	0.062284	6.24887	1.098612

1	0	30.6588	0.04311	6.49883	1.386294
1	0	23.16	0.055384	6.68139	1.609438
1	0	23.3856	0.037495	6.54444	1.791759
1	0	18.4284	0.046712	6.48046	1.94591
1	0	14.9428	0.050152	5.46139	2.079442
2	0	36.63	0.068129	6.2243	0
2	0	36.63	0.084464	5.90954	0.693147
2	0	36.63	0.098709	5.44468	1.098612
2	0	36.63	0.074441	5.68303	1.386294
2	0	36.63	0.086878	3.57375	1.609438
1	0	30.97	0.079937	3.89586	1.791759
1	0	31.25	0.082799	3.25508	1.94591
1	0	31.26	0.0794	3.11004	2.079442
2	1	64.19	0.092354	1.06571	0
2	1	64.19	0.090379	0.54413	0.693147
2	1	64.19	0.081212	1.26558	1.098612
2	1	54.52	0.056463	1.57867	1.386294
2	1	54.52	0.042777	0.91732	1.609438
2	1	54.52	0.044369	0.38597	1.791759
2	1	54.52	0.038555	0.34705	1.94591
2	1	53.77	0.053761	0.21025	2.079442
0	2	43.84	0.081685	1.25249	0
0	2	43.84	0.066149	0.47958	0.693147
0	2	43.84	0.052971	0.33017	1.098612
0	2	38.69	0.007096	1.4595	1.386294
0	2	38.69	-0.02825	0.48333	1.609438
0	2	38.69	0.026042	0.26454	1.791759
0	2	46.63	0.012167	0.11702	1.94591
0	2	39.57	0.011613	1.2224	2.079442
0	1	17.97	0.053646	1.3675	0
0	1	52.67	0.061598	0.2083	0.693147
0	1	52.67	0.090134	0.13218	1.098612
0	1	43.59	0.124362	0.80275	1.386294
0	1	33.49	0.046014	1.91295	1.609438
0	2	34.18	0.024585	1.34908	1.791759
0	2	31.97	0.03529	1.16803	1.94591
2	0	38.4	0.059759	7.51662	0
2	0	38.4	0.066291	6.809	0.693147
2	0	38.4	0.068731	5.91897	1.098612
0	1	24.5451	0.089797	7.98623	0
0	1	24.7693	0.092644	7.12123	0.693147
0	1	24.6944	0.051728	7.26044	1.098612
0	1	24.2965	0.005216	7.53641	1.386294
0	1	19.4301	0.023498	5.65785	1.609438
0	1	19.4911	0.017045	5.4756	1.791759
1	1	19.5009	0.039045	5.62196	1.94591
1	1	19.5009	0.039593	5.45697	2.079442
0	2	26.63	0.096417	7.62635	0
0	2	26.63	0.084478	6.93327	0.693147
0	2	26.63	0.077414	7.02981	1.098612
0	1	25.2088	0.043777	5.85055	1.386294
0	1	23.881	0.046935	4.73469	1.609438
0	1	24.131	0.050203	4.50507	1.791759
0	1	22.932	0.055802	3.79566	1.94591
0	1	10.6468	0.040252	4.53224	2.079442
1	1	54.75	0.08785	7.1214	0
1	1	54.75	0.100346	6.81	0.693147
1	1	54.75	0.121762	6.92267	1.098612

1	1	55.8125	0.128429	6.8935	1.386294
1	1	55.31	0.145	0.58631	1.609438
1	1	55.29	0.18213	0.94341	1.791759
2	0	40.75	0.079328	1.81991	0
2	0	36.82	0.106145	1.7197	0.693147
2	0	36.74	0.125862	1.73633	1.098612
0	1	50.25	0.07116	0.12062	0
0	1	50.25	0.108434	0.00007	0.693147
0	1	49.72	0.104601	0.00134	1.098612
0	1	44.95	0.094097	1.2526	1.386294
0	1	44.54	0.093262	1.21163	1.609438
0	1	37.7	0.05061	0.91156	1.791759
0	1	37.75	-0.10165	1.04081	1.94591
2	0	51.55	0.056436	4.8146	0
2	0	51.55	-0.01949	4.564	0.693147
2	0	51.55	0.010464	4.82718	1.098612
2	0	54.1205	-0.00025	5.40199	1.386294
2	0	41.0031	0.012393	4.84257	1.609438
2	0	39.9831	-0.01596	4.87348	1.791759
1	0	50.25	0.048981	7.88009	0
1	0	50.25	0.010093	7.43922	0.693147
1	0	50.25	0.010878	5.10911	1.098612
2	0	50.25	0.01872	5.25754	1.386294
1	0	56.33	0.027607	4.34374	1.609438
1	0	38.54	0.027819	3.34739	1.791759
1	0	19.5783	0.054425	3.1427	1.94591
1	0	37.85	0.049429	3.0583	2.079442
0	2	40.5182	0.083817	5.30714	0
0	2	40.5182	0.05555	5.08473	0.693147
0	2	40.5182	0.058422	5.32563	1.098612
0	2	29.117	0.04405	0.29107	1.386294
0	2	29.11	0.048496	0.11599	1.609438
0	2	29.11	0.053479	0.02124	1.791759
0	2	23.39	0.074718	0.12	1.94591
0	2	23.72	0.086392	0.20304	2.079442
1	0	45.35	0.089259	4.841	0
1	0	45.35	0.089128	4.301	0.693147
1	0	45.35	0.052731	4.2734	1.098612
1	0	45.35	0.061403	5.08976	1.386294
1	0	42.62	0.065444	5.58975	1.609438
1	0	42.62	0.037179	4.88974	1.791759
1	0	44.2	0.049291	4.90261	1.94591
1	0	44.13	0.056912	4.65618	2.079442
1	0	41.0146	0.082354	6.9615	0
1	0	41.0146	0.045753	6.70004	0.693147
1	0	41.0146	0.040498	6.477	1.098612
1	0	41.1146	0.023927	6.98071	1.386294
1	0	30.19	0.014839	4.13803	0
1	0	31.47	0.014093	4.138	0.693147
1	0	50.48	0.076692	7.1775	0
1	0	50.48	0.072812	6.66064	0.693147
1	0	50.48	0.062459	6.06893	1.098612
1	0	49.27	0.033983	6.2815	1.386294
1	0	51	0.000965	5.1285	1.791759
1	0	51	-0.08226	5.09587	1.94591
1	0	51	0.009495	5.08818	2.079442
1	1	41.07	0.063562	4.78414	0
1	1	41.21	0.044946	4.75392	0.693147

1	0	41.21	0.007841	4.22902	1.098612
2	0	41.13	0.008485	4.51543	1.386294
2	0	64.58	0.068928	6.90352	0
2	0	64.58	0.082079	6.24474	0.693147
2	0	63.17	0.061644	5.78973	1.098612
2	0	63.17	0.035467	5.18558	1.386294
2	0	64	0.048684	5.19271	1.609438
2	0	64	0.07065	5.2013	1.791759
2	0	62.2799	0.072062	5.06224	1.94591
2	0	63.5699	0.081836	6.08938	2.079442
2	0	53.96	0.066337	1.68587	0
2	0	53.96	0.041125	0.76714	0.693147
2	0	53.96	0.035302	0.65614	1.098612
2	0	53.96	0.032501	1.33533	1.386294
2	0	53.96	0.035127	0.39784	1.609438
2	0	43.2	0.034929	2.90419	1.791759
2	0	41.25	0.019508	3.23627	1.94591
2	0	46.7349	-0.09541	0.48971	2.079442
1	0	55.35	0.065433	1.7324	0
1	0	55.35	0.054878	0.287	0.693147
1	0	55.35	0.055118	0.13607	1.098612
1	0	55.48	0.04914	0.61045	1.386294
1	0	42.48	0.036195	1.81117	1.609438
1	0	31.98	0.038282	2.66248	1.791759
1	0	24.08	0.067148	4.21517	1.94591
1	0	24.08	0.101024	5.05825	2.079442
1	0	36.7922	0.084581	7.74715	0
1	0	36.1842	0.112933	6.9541	0.693147
1	0	37.9622	0.119037	5.53966	1.098612
1	0	37.9778	0.096615	6.47818	1.386294
1	0	38.3054	0.077165	5.85747	1.609438
1	0	27.9158	0.048076	5.98604	1.791759
1	0	27.5414	0.016635	6.16559	1.94591
1	0	32.1668	0.047036	5.67645	2.079442
1	1	63.37	0.058275	9.083	0
1	1	63.37	0.034645	9.069	0.693147
1	1	55.7	0.017362	9.1984	1.098612
1	1	55.7	0.027541	8.75369	1.386294
1	1	47.35	0.031807	7.34781	1.609438
1	1	46.9	0.012704	7.22891	1.791759
1	1	46.62	0.000961	7.06962	1.94591
2	0	67.49	0.061575	8.07786	1.386294
2	0	67.49	0.07062	8.20691	1.609438
2	0	68.49	0.08447	8.60828	1.791759
0	2	63.478	0.067064	6.454	0
0	2	47.577	0.055781	4.89083	0
0	2	47.587	0.032721	4.83094	0.693147
0	2	41.4475	0.026921	5.06467	1.098612
0	2	38.1975	0.005138	4.76897	1.386294
0	2	38.2499	-0.04151	4.66739	1.609438
1	0	48.0289	0.065888	6.65422	1.386294
2	0	9.548	0.119456	7.94	0
2	0	59.01	0.052379	6.5348	0
2	0	59.47	0.049491	6.06944	0.693147
2	0	59.41	0.020606	5.89162	1.098612
2	0	59.41	0.013203	5.65342	1.386294
2	0	54.107	-0.05784	6.13544	1.791759
2	1	39.2997	0.043257	0.75113	0.693147

2	1	58.6374	0.000409	1.22169	1.098612
2	1	58.6377	0.007358	0.85491	1.386294
2	0	70.61	0.154534	0.533	0
2	0	52.96	0.089292	0.41646	0
2	0	53.12	0.054703	0.59679	0.693147
2	0	53.12	0.043642	0.39704	1.098612
2	0	51.31	0.014241	0.53272	1.386294
2	0	50.31	0.038845	0.54022	1.609438
2	0	48.36	0.02926	0.3437	1.791759
1	0	37.093	0.059488	7.54228	0
1	0	37.1117	0.06021	7.6454	0.693147
1	0	36.6131	0.102005	7.64018	1.098612
1	0	36.6131	0.078006	8.0772	1.386294
1	0	21.6233	0.054782	7.69875	1.609438
2	0	67.5	0.097133	0.33847	0
2	0	67.5	0.059118	0.21559	1.098612
2	0	67.5	0.055129	0.00478	1.386294
2	0	64.1	0.075231	0.06665	1.609438
2	0	55.12	0.076086	0.5018	1.791759
0	1	69.43	0.050285	8.0164	1.386294
0	1	68.43	0.042656	7.03276	1.609438
0	1	68.43	0.043973	7.1016	1.791759
1	1	61.7775	0.043585	5.11902	0
1	1	61.7775	0.043013	5.32724	0.693147
1	1	61.7775	0.031508	5.11405	1.098612
1	1	61.3939	0.039209	5.12061	1.386294
1	1	59.5989	0.036249	5.11403	1.609438
1	1	59.5989	0.031657	5.11404	1.791759
1	0	21.9903	0.050291	3.25758	0
1	0	21.9903	0.025075	3.40969	0.693147
1	0	21.9903	0.019087	3.38355	1.098612
1	0	21.9903	0.021493	3.44702	1.386294
1	0	21.9903	0.009158	3.18154	1.609438
1	0	21.9903	0.009293	3.19777	1.791759
1	1	33.28	0.052274	1.94	0
1	1	33.28	0.058675	1.8932	0
1	1	34.46	0.054469	1.35766	0.693147
1	1	34.63	0.059417	1.40113	1.098612
1	1	34.2	0.076462	1.44492	1.386294
1	1	31.99	0.072593	1.7011	1.609438
1	2	37.6551	0.088691	4.67521	0
1	2	37.6551	0.086998	4.80714	0.693147
1	2	37.6551	0.080319	4.84528	1.098612
0	2	37.6551	0.10511	5.32045	1.386294
0	2	35.8202	0.120138	6.0896	1.609438
0	2	34.4702	0.119623	5.90666	1.791759
1	0	59.2284	0.076849	7.33477	0
1	0	59.2284	0.071648	7.04691	0.693147
1	0	61.72	0.065488	7.01669	1.098612
1	0	52.9506	0.055932	7.25415	1.386294
2	0	48.94	0.064374	1.40923	0
2	0	57.17	0.057576	0.68984	0
2	0	57.17	0.043049	0.40009	0.693147
2	0	56.6	0.044779	0.54285	1.098612
1	0	63.48	0.027895	0.03592	1.386294
1	0	63.48	0.028885	0.03652	1.609438
1	0	55.22	0.136278	1.256	1.386294
1	0	37.76	0.147027	0.51981	0

1	0	37.76	0.139985	0.08659	0.693147
1	0	37.76	0.188022	0.04782	1.098612
1	0	37.36	0.149871	0.715	1.609438
1	2	46.8	0.145947	10	0
1	2	35.056	0.088377	7.52033	0
1	2	35.056	0.119789	7.79723	0.693147
1	2	35.526	0.044776	7.516	1.098612
1	2	36.126	0.017558	7.51641	1.386294
1	2	33.904	-0.02967	6.87742	1.609438
0	1	28.41	0.024129	1.25143	0
0	1	28.41	-0.00735	0.22699	0.693147
0	1	27.94	0.01132	0.85309	1.098612
0	1	30.84	0.035972	0.55404	1.386294
0	1	27.84	0.020709	0.23644	1.609438
0	1	39.17	0.027775	1.88547	0
1	0	46.19	0.009563	1.26517	0.693147
1	0	46.19	-0.09287	1.2522	1.098612
1	0	46.19	0.033758	0.61677	1.386294
0	2	59.71	0.03677	0.04946	1.386294
0	2	62.16	0.043139	0.03255	1.609438
0	1	70.05	0.087838	0.49771	0
0	1	70.05	0.100162	0.15493	0.693147
0	1	70.05	0.109097	0.0684	1.098612
0	2	70.05	0.080792	0.17328	1.386294
0	2	33.62	0.083631	0.01452	1.609438
2	0	60.4821	0.046286	1.15451	0
2	0	58.9298	0.057283	0.80843	0.693147
2	0	58.6558	0.051229	0.53574	1.098612
2	0	59.7179	0.001154	0.15902	1.386294
2	0	52.98	-0.04595	0.56898	1.609438
1	1	71.56	0.072524	0.03902	0
1	1	71.56	0.05885	0.035	0.693147
1	1	71.56	0.056283	0.03503	1.098612
1	1	69.78	0.030907	0.03571	1.386294
1	1	69	0.030299	0.068	1.609438
2	0	62.6341	0.136836	4.45031	0
2	0	62.0349	0.133076	4.43866	0.693147
2	0	62.0268	0.140466	4.39837	1.098612
2	0	60.486	0.133775	4.4984	1.386294
2	0	60.486	0.123252	4.39279	1.609438
0	1	68.7546	0.046717	4.89002	0.693147
0	1	68.7174	0.012509	4.887	1.098612
2	0	50.9	0.038577	0.18363	1.609438
1	0	37.67	0.023427	1.10026	1.098612
1	0	28.26	0.020041	1.87513	1.386294
1	0	26.23	0.037794	6.23214	0
1	0	26.23	0.030288	6.01769	0.693147
1	0	26.23	0.032202	5.74035	1.098612
2	1	26.23	0.03231	4.49032	1.386294
2	1	25.03	0.015781	4.68503	1.609438
2	0	60.88	0.064859	1.03128	0
2	0	59.49	0.089017	0.92559	0.693147
2	0	59.49	0.068912	0.51801	1.098612
2	0	56.59	0.02333	0.61529	1.386294
2	0	52.99	0.028862	0.60823	1.609438
0	1	60.3	0.044198	0.547	0
0	1	45.27	0.027715	0.41199	0
0	1	45.27	0.024941	0.30269	0.693147

0	1	45.59	0.02271	0.25698	1.098612
0	1	43.43	0.004437	0.33407	1.386294
1	1	39.1389	0.123464	0.32275	0
1	1	39.1389	0.128878	0.30909	0.693147
1	1	67.3689	0.079434	0.30997	1.098612
0	2	57.05	0.078454	1.55686	0
0	2	55.66	0.036819	0.91032	0.693147
0	2	42.68	0.025377	1.23792	1.098612
0	2	40.02	0.045352	0.84771	1.386294
2	0	45.33	0.064544	1.15692	1.098612
2	0	44.58	0.079665	1.48968	1.386294
0	1	41.1622	0.118436	0.51054	0
0	1	40.9508	0.109827	0.49715	0.693147
0	1	41.1622	0.089729	0.50017	1.098612
0	1	41.1622	0.132511	0.57104	1.386294
0	1	47.86	0.064919	2.25755	0
0	1	45.93	0.064556	1.98908	0.693147
0	1	45.44	0.048093	1.54151	1.098612
0	1	47.11	0.075897	1.0148	1.386294
1	0	40.96	0.051562	0.04113	0
1	0	40.96	0.042057	0.27028	0.693147
1	0	39.44	0.058032	0.72602	1.098612
1	0	39.38	0.109339	1.36094	1.386294
0	1	27.06	0.087512	4.33384	0
0	1	27.06	0.076509	4.92833	0.693147
0	1	17.01	0.088328	3.56687	1.098612
0	1	17.01	0.076729	3.70375	1.386294
1	0	44.02	0.074761	0.03035	0
1	0	44.02	0.062838	0.00252	0.693147
1	0	44.02	0.043674	0.01232	1.098612
1	0	43.51	0.043756	0.0165	1.386294
1	0	45.24	0.046241	1.18837	0
1	0	45.24	0.038794	1.52582	0.693147
1	0	45.24	0.03481	1.10817	1.098612
1	0	38.75	0.040086	0.45564	1.386294
1	1	57.76	0.077587	6.259	0
1	1	57.76	0.045416	4.69842	0
1	1	57.76	0.058482	4.66823	0.693147
1	1	57.2088	0.028487	4.06705	1.098612
1	1	70	0.064971	0.00194	0
1	1	69.05	0.039414	0.00039	0.693147
1	1	69.08	0.056248	0.07623	1.098612
1	0	61.5002	0.024988	7.50067	0
1	0	61.5002	0.010155	7.20455	0.693147
1	0	58.6908	0.013739	6.627	1.098612
0	1	55.7766	0.0303	2.45652	0
0	1	56.7666	0.018328	2.29902	0.693147
0	1	56.7666	-0.01156	2.299	1.098612
0	2	35.55	0.091767	2.2024	0
0	2	35.55	0.07163	1.97719	0.693147
0	2	35.55	0.050914	1.9886	1.098612
0	1	49.7143	0.125543	6.98157	0
0	1	49.1902	0.130208	6.92947	0.693147
0	1	49.3969	0.056045	6.71939	1.098612
1	0	46.0997	0.112538	2.25898	0
1	0	45.0345	0.112813	1.81367	0.693147
1	0	44.852	0.141779	1.6591	1.098612
2	0	63.1093	0.135148	8.62783	0

2	0	62.1418	0.136923	8.89535	0.693147
2	0	61.8946	0.167396	8.40975	1.098612
0	1	55.4982	0.063633	2.69235	0
0	1	49.8882	0.012017	2.63091	0.693147
1	0	32.38	0.051716	1.74348	0
1	0	32.47	0.003342	1.08183	0.693147
1	0	35.152	0.077203	5.90078	0
1	0	35.232	0.071517	5.8568	0.693147
1	0	35.232	0.065396	5.58337	1.098612
1	0	63.3902	0.10171	4.43805	0
1	0	63.33	0.097869	5.45358	0.693147
1	0	63.33	0.100479	5.45753	1.098612
1	0	33.97	0.071674	0.13408	0
1	0	33.97	0.070498	0.00482	0.693147
1	0	33.97	0.080257	0.44575	1.098612
1	1	57.88	0.113314	0.00117	0
1	1	56.83	0.105661	0.00011	0.693147
2	0	33.7506	0.053234	1.20471	0.693147
2	0	33.7506	0.032606	0.49721	1.098612
2	0	66.32	0.057689	0.70294	0.693147
2	0	66.32	0.055413	0.65735	1.098612
1	0	58.7294	0.096987	0.94541	0
1	0	58.7294	0.093001	0.72394	0.693147
1	0	58.7294	0.104868	0.71802	1.098612
2	0	37.9437	0.007273	5.52375	1.098612
2	0	50.69	0.08292	0.99976	0
2	0	50.69	0.081314	0.94524	0.693147
2	0	50.69	0.065065	0.92279	1.098612
1	0	50.57	0.104338	1.06635	0
1	1	50.57	0.048608	0.94487	0.693147
1	1	50.57	0.005902	0.04469	1.098612
2	1	65.48	0.069604	1.13774	0
2	1	65.48	0.053793	0.99878	0.693147
2	1	65.48	0.03432	1.26464	1.098612
1	0	42.03	0.094913	3.28294	0
1	0	42.03	0.087373	3.25673	0.693147
1	0	42.03	0.079634	3.05702	1.098612
2	0	66.3525	0.085273	0.47783	0
2	0	66.3525	0.083057	0.52338	0.693147
2	0	66.1589	0.083562	1.10323	1.098612
0	2	53.4276	0.052605	3.60357	0
0	2	62.4446	0.04976	3.51565	0.693147
0	2	44.01	0.09097	1.14563	0
0	2	44.01	0.081639	1.25051	0.693147
2	0	64.31	0.1151	5.836	0
2	0	64.31	0.118109	5.83556	0.693147
1	0	35.567	0.095523	1.49179	0
1	0	35.567	0.014035	1.42029	0.693147
1	1	63.7659	0.112788	3.295	0
1	1	47.8182	0.078977	2.47232	0
0	1	36.65	0.088559	3.91939	0
2	1	64.79	0.086972	0.01038	0
0	1	55.9634	0.09514	6.7525	0
2	0	48.7429	0.094366	1.52819	0
0	1	69.1728	0.051425	1.6218	0
2	0	56.73	0.056499	0.64533	0
2	0	56.73	0.063923	1.12599	0.693147
2	0	56.73	0.074682	1.71259	1.098612

2	0	56.73	0.036469	0.89576	1.386294
2	0	56.73	0.038441	0.35878	1.609438
2	0	36.01	0.028537	0.79918	1.791759
2	0	33.15	0.010625	0.88043	1.94591
2	0	33.15	0.022352	0.31613	2.079442
2	0	33.15	0.006042	0.07174	2.197225
2	0	30.21	0.005543	1.16503	2.397895
1	0	33.99	0.076721	0.21949	0
1	0	33.99	0.087899	0.83055	0.693147
1	0	33.99	0.113965	0.97543	1.098612
1	0	33.99	0.123639	0.66201	1.386294
1	0	31.58	0.132402	1.44756	1.609438
1	0	30.02	0.093666	1.90893	1.791759
1	0	28.05	0.096467	2.58627	1.94591
2	0	25.68	0.035591	2.98924	2.079442
1	0	19.01	0.039145	2.32251	2.197225
1	0	18.14	0.109144	4.90858	2.397895
0	1	17.7357	0.024622	2.77983	2.302585
1	0	21.13	0.077455	4.39309	0
1	0	21.13	0.089987	4.0337	0.693147
1	0	21.13	0.084268	4.11599	1.098612
1	0	21.13	0.108018	5.66571	1.386294
1	0	21.13	0.106313	3.87633	1.609438
1	0	23.43	0.103819	5.05575	1.791759
1	0	21.52	0.029443	4.30392	1.94591
1	0	23.9	0.016217	2.52071	2.079442
1	0	24.01	0.104144	2.18547	2.197225
1	0	23.9	0.114658	2.14818	2.302585
0	2	41.97	0.053448	1.5756	0
0	2	41.97	0.049223	1.2189	0.693147
0	2	41.97	0.065142	1.13682	1.098612
0	2	41.97	0.105608	2.31361	1.386294
0	2	38.74	0.170419	2.47747	1.609438
0	2	30.25	0.144089	1.87217	2.079442
0	2	30.09	0.079588	1.15904	2.197225
1	0	45.97	0.084111	0.70098	0
1	0	45.97	0.063688	1.00497	0.693147
1	0	45.97	0.052153	0.62496	1.098612
1	0	45.21	0.056675	0.34269	1.386294
1	0	40.22	0.049527	0.00719	1.609438
1	0	39.84	0.05853	0.58385	1.791759
1	0	32.42	0.048551	1.96633	1.94591
1	0	30.57	0.069077	1.23213	2.079442
1	0	30.21	0.060823	0.5828	0
1	1	24.2	0.05276	1.81809	2.197225
1	1	23.84	0.064044	1.81939	2.302585
1	1	32.15	0.11477	2.35809	2.397895
1	0	52.77	0.042791	1.92946	0
1	0	52.77	0.037341	2.07349	0.693147
1	0	52.77	0.026763	0.87315	1.098612
1	0	52.77	0.024215	0.45252	1.386294
1	0	51.07	0.012169	1.17517	1.609438
1	0	51.07	0.013494	0.87421	1.791759
1	0	52.08	0.036232	0.26117	1.94591
1	0	45.48	0.024943	2.09601	2.079442
1	0	45.48	0.03454	1.30181	0
1	0	46.04	0.028691	1.00488	0.693147
1	0	46.04	0.034145	0.66684	1.098612

1	1	26.7	-0.05362	2.10692	2.302585
0	1	27.98	-0.10587	0	2.302585
1	1	43.2	0.061513	4.25638	0.693147
1	1	43.2	0.048483	4.10727	1.098612
1	1	25.31	0.005064	2.05828	1.791759
0	1	40.2165	0.045603	6.59019	0.693147
0	1	40.9342	0.046066	6.65566	1.098612
0	1	40.9342	0.023708	7.27531	1.386294
0	1	37.5295	0.007729	6.48787	1.609438
0	1	31.6119	0.023818	5.20625	1.791759
0	1	31.6119	0.073668	5.11636	1.94591
0	1	27.4373	0.014374	6.04086	2.079442
0	1	27.8098	-0.06097	5.82285	2.197225
2	0	29.93	0.082143	2.37857	1.609438
2	0	34.49	0.047149	1.25069	1.791759
2	0	35.43	0.052849	2.17984	1.94591
2	0	33.36	0.049407	1.4671	2.079442
2	0	27.04	0.04928	1.41177	2.197225
2	0	27.04	0.058899	0.74336	2.302585
2	0	62.06	0.074608	1.36486	0
2	0	62.06	0.086008	1.88018	0.693147
2	0	62.06	0.074891	2.40029	1.098612
2	0	60.99	0.079332	1.07813	1.386294
2	0	52.14	0.095303	1.73133	1.609438
2	0	46.97	0.121795	1.35275	1.791759
2	0	45.67	0.088945	0.74591	1.94591
2	0	45.67	0.041384	0.82437	2.079442
2	0	46.05	0.040433	0.42265	2.197225
2	0	46.13	0.045367	0.61519	2.302585
1	0	21.9908	0.072877	4.49	0
1	0	21.9908	0.060639	4.86803	0.693147
1	0	21.9908	0.075728	4.80446	1.098612
1	0	22.9214	0.090207	6.0801	1.386294
1	0	22.9214	0.101687	6.1405	1.609438
1	0	22.4433	0.091263	4.96053	1.791759
1	0	25.6189	0.077536	4.55527	1.94591
1	0	25.6189	0.04561	4.32512	2.079442
1	0	24.651	0.053887	4.35916	2.197225
1	0	24.651	0.091447	4.2137	2.302585
1	0	46.12	0.04693	0.1455	0
1	0	46.12	0.031779	0.0779	0.693147
1	0	46.12	0.014344	0.046	1.098612
1	0	40.99	0.03136	0.00901	1.386294
1	0	40.99	0.023261	0.207	1.609438
1	0	37.39	0.015247	0.23867	1.791759
1	0	28.04	0.029093	2.29478	1.94591
1	0	28.04	0.025055	2.504	2.079442
2	0	28.04	0.041391	2.258	2.302585
1	1	52.4	0.058108	0.3868	0
1	1	52.41	0.059106	1.18744	0.693147
1	1	52.41	0.085271	1.09478	1.098612
1	1	42.23	0.097372	2.05181	1.386294
1	1	40.51	0.026822	0.27682	1.609438
1	1	18.88	0.054448	0.39224	1.94591
1	1	18.28	0.021016	0.24764	2.079442
0	2	38.6936	0.017066	3.25185	1.609438
0	2	39.0236	0.004374	3.42783	1.791759
0	2	39.0236	-0.01912	3.06371	1.94591

1	2	33.859	0.017017	3.01971	2.197225
1	0	61.5	0.052616	8.82927	0
1	0	61.5	0.04051	8.54031	0.693147
1	0	61.5	0.046948	8.62578	1.098612
1	0	58.63	0.046897	7.31952	1.386294
1	0	50.755	0.019465	6.57522	1.609438
1	0	29.08	-0.05375	5.13353	1.791759
1	0	24.09	0.013632	4.21615	1.94591
1	0	20.4379	0.041169	3.02877	2.079442
0	1	35.07	0.037764	2.8114	0
0	1	35.07	0.014267	1.9291	0.693147
0	1	35.07	0.026238	1.94523	1.098612
0	1	34.316	0.063561	1.76501	1.386294
0	1	34.167	0.04097	2.01324	1.609438
0	1	34.167	0.047975	1.79204	1.791759
0	1	27.923	0.026306	1.91654	1.94591
0	1	23.424	0.022056	1.33013	2.079442
0	1	18.294	-0.03719	2.03957	2.197225
0	1	34.184	0.037815	2.03003	2.302585
1	1	64.17	0.09221	6.6605	0
1	1	63.6557	0.104674	6.32039	0.693147
1	1	63.6557	0.074739	6.13436	1.098612
1	0	65.17	0.055649	5.87548	1.386294
1	0	54.22	0.036989	7.65741	1.609438
1	0	54.09	0.060511	8.15687	1.791759
1	0	52.53	0.064151	8.11744	1.94591
1	0	52.17	0.032592	6.98908	2.079442
1	0	54.13	-0.01381	6.50616	2.197225
0	2	29.83	0.053182	3.52494	1.609438
0	2	29.9	0.037255	4.32507	1.791759
0	2	29.9	0.023697	3.26918	1.94591
0	2	29.9	0.042352	3.64743	2.079442
0	2	29.9	0.018025	3.18355	2.197225
0	2	29.9	0.045623	3.18837	2.302585
0	1	40.36	0.064366	0.82636	0
0	1	40.36	0.02595	0.3309	0.693147
0	1	40.36	0.014712	0.25012	1.098612
0	1	40.36	0.042394	0.18963	1.386294
0	1	40.36	0.044409	0.6894	1.609438
0	1	39.83	0.0245	0.18134	1.791759
0	1	39.64	0.024118	0.69783	1.94591
0	1	39.47	0.080337	0.19046	2.079442
0	1	39.26	0.175071	0.07381	2.302585
1	0	39.32	0.064354	1.16209	0
1	0	39.32	0.063943	0.89038	0.693147
1	0	39.38	0.049004	0.834	1.098612
1	0	39.38	0.047712	0.834	1.386294
1	0	39.38	0.063456	1.0414	1.609438
1	0	34.96	0.046106	2.07746	1.791759
1	0	34.96	0.061244	0.86871	1.94591
1	0	35.12	0.047301	0.86939	2.079442
1	0	35.79	0.03933	0.52475	2.197225
1	0	24.2	0.029963	2.81262	2.302585
1	0	42.7104	0.066264	7.09552	0
1	0	42.7104	0.07019	6.97721	0.693147
1	0	43.1433	0.054889	7.12507	1.098612
1	0	42.0106	0.051341	6.29358	1.386294
1	0	41.4755	0.02142	6.07763	1.609438

1	0	35.852	0.008068	5.2738	1.791759
2	0	41.66	0.009008	4.48644	2.197225
2	0	41.66	0.004185	4.21193	2.302585
1	2	49.21	0.012861	1.88781	0.693147
1	2	49.21	0.018346	1.50868	1.386294
1	0	17.78	0.062983	3.2704	0
1	0	17.78	0.063701	3.59508	0.693147
1	0	17.78	0.032352	3.1261	1.098612
1	0	17.55	0.048848	3.04742	1.386294
1	0	31.64	0.006965	2.068	1.609438
1	0	25.91	0.006586	2.88437	1.791759
1	0	26.13	0.022838	2.9341	1.94591
1	0	25.18	0.022207	4.11846	2.079442
1	0	24.59	0.047613	4.19714	2.197225
1	0	20.63	0.035553	5.47403	2.302585
1	0	62.1	0.116232	0.11015	0
1	0	62.1	0.081681	0.2643	0.693147
1	0	62.1	0.085897	0.62708	1.098612
1	0	62.03	0.050369	1.0046	1.386294
1	0	60.075	0.054524	0.76682	1.609438
1	0	60.355	0.073613	1.02759	1.791759
1	0	60.36	0.012002	1.18946	1.94591
1	0	60.36	0.105282	0.81719	2.079442
2	0	32.3888	0.032805	5.82749	0
2	0	32.3888	0.035452	5.48006	0.693147
2	0	32.3888	0.039613	4.29923	1.098612
2	0	31.9658	0.045244	4.48704	1.386294
2	0	31.9658	0.014466	4.22573	1.609438
2	0	32.9488	0.008147	4.20649	1.791759
2	0	44.36	0.007036	5.65207	1.94591
2	0	32.49	0.015762	6.68009	2.079442
2	0	27.72	0.023244	6.37826	2.197225
1	0	19.0339	0.036738	5.8774	2.302585
3	0	41.26	0.074599	0.54182	0
3	0	41.55	0.033239	0.06945	0.693147
3	0	41.55	0.02922	0.08352	1.098612
3	0	41.55	0.029177	0.09382	1.386294
1	0	36.62	0.034832	0.06914	1.609438
1	0	33.99	0.054424	0.14268	1.791759
3	0	23.09	0.071719	2.46238	1.94591
3	0	23.24	0.04776	2.3762	2.079442
3	0	23.51	0.002649	1.34205	2.197225
3	0	23.51	0.035998	1.69785	2.302585
1	0	18.7638	0.099586	1.49697	0
0	2	58.5612	0.079419	4.05293	0
0	2	58.5612	0.1059	4.46165	0.693147
0	2	58.5612	0.125927	5.17858	1.098612
0	2	58.0044	0.130076	5.15603	1.386294
0	2	50.1648	0.080933	3.81792	1.609438
0	2	44.743	0.061296	4.2453	1.791759
0	2	44.743	0.047271	3.85434	1.94591
0	2	44.1604	0.050715	3.83639	2.079442
0	2	45.6611	0.019426	3.759	2.197225
1	0	34.81	0.008723	0.00255	1.94591
1	0	35.25	0.011698	0.03031	2.079442
1	0	35.38	0.013728	0.00138	2.197225
1	0	35.38	0.011929	0.00276	2.302585
1	0	47.14	0.063195	0.18154	0

1	0	47.14	0.049587	0.54761	0.693147
1	0	47.14	0.069799	0.09368	1.098612
1	0	47.6	0.078913	0.0247	1.386294
1	0	47.21	0.063444	0.14313	1.609438
1	0	45.41	0.026247	0.145	1.791759
1	0	45.41	0.034392	0.6392	1.94591
1	0	44.57	0.007258	0.05941	2.079442
1	0	44.51	0.006059	0.035	2.197225
1	0	44.52	0.004646	0.04749	2.302585
1	0	47.5	0.063394	1.30991	0
1	0	47.5	0.079329	1.76035	0.693147
1	0	47.5	0.083064	2.16752	1.098612
1	0	42.5	0.091479	2.58221	1.386294
1	0	39.25	0.095597	1.99414	1.609438
1	0	31.33	0.052568	3.37885	1.791759
1	0	28.29	0.070744	3.8949	1.94591
1	0	28.78	0.060816	3.29222	2.079442
1	0	57.49	0.052522	0.709	0
1	0	57.49	0.098426	1.10949	0.693147
1	0	57.49	0.133202	1.37457	1.098612
1	0	57.49	0.171743	2.24009	1.386294
1	0	57.33	0.186065	0.58145	1.609438
1	0	48.81	0.12646	1.77388	1.791759
1	0	48.33	0.095274	1.02424	1.94591
1	0	48.34	0.125426	0.99428	2.079442
1	0	48.38	0.092788	2.26237	2.197225
1	0	44.9	-0.04982	1.81686	2.302585
0	1	55.5053	0.058731	7.091	0
0	1	55.5053	0.057483	6.562	0.693147
0	1	55.5053	0.033735	6.57501	1.098612
0	1	55.5053	0.031948	6.62046	1.386294
0	1	32.7138	0.058658	4.19278	1.609438
0	1	32.7138	0.084859	4.38082	1.791759
0	2	23.5329	0.051866	3.65724	1.94591
0	2	20.9778	0.028812	2.59727	2.079442
0	3	20.9778	0.00892	2.20668	2.302585
2	0	57.978	0.097906	10	0
2	0	57.978	0.076132	7.6521	0
2	0	57.978	0.039261	7.36597	0.693147
2	0	57.978	0.022592	6.8313	1.098612
2	0	52.0935	0.024088	6.38086	1.386294
2	0	54.4482	0.039374	6.88873	1.609438
2	0	52.2485	0.069693	6.43538	1.791759
2	0	48.7114	0.05393	5.36872	1.94591
2	0	45.79	0.070202	4.61265	2.197225
1	0	69.25	0.025117	2.3668	0.693147
1	0	69.25	0.019925	2.395	1.098612
1	0	62.68	0.035057	3.87332	1.386294
1	0	62.295	0.01461	2.20738	1.609438
1	0	53.0517	0.02035	2.32848	1.791759
1	0	53.0517	0.01598	1.82331	1.94591
1	0	52.0417	-0.02742	1.80428	2.079442
1	0	47.7417	0.032913	1.82912	2.197225
0	1	64.13	0.087156	2.39496	0
0	1	64.13	0.074273	2.02678	0.693147
0	1	64.13	0.073975	0.8215	1.098612
0	1	62.51	0.084298	0.90143	1.386294
0	1	60.63	0.097295	0.89819	1.609438

0	1	60.65	0.057958	0.203	1.791759
0	1	61.25	0.031147	0.14518	1.94591
0	1	61.25	0.018593	0.00119	2.079442
0	1	61.25	0.02876	0.01463	2.197225
1	1	37.178	0.12391	10	0
1	1	27.804	0.074303	7.79281	0
1	1	27.804	0.027939	6.75742	0.693147
1	1	27.804	0.015603	4.68392	1.098612
1	1	35.49	0.059637	3.706	1.386294
1	1	28.06	0.023861	3.28278	1.609438
1	1	28.96	0.032233	2.95504	1.791759
1	1	28.96	0.04407	3.10371	1.94591
1	1	28.96	0.041423	3.034	2.079442
1	1	23.94	0.017484	1.46434	2.197225
2	1	33.15	0.088445	10.001	0
2	1	24.8455	0.038107	7.69601	0
2	1	24.8455	0.042118	7.1878	0.693147
2	1	24.8455	0.017359	6.84633	1.098612
2	1	24.8455	0.008783	6.64716	1.386294
2	1	24.8455	0.018827	5.29435	1.609438
2	1	24.8455	0.027679	3.75998	1.791759
1	0	30.11	0.073799	0.71216	0
1	0	30.2	0.076476	0.86967	0.693147
1	0	29.92	0.093868	1.46383	1.098612
1	0	29.75	0.080891	0.52054	1.386294
1	0	26.77	0.000084	0.95514	1.609438
1	0	26.18	0.009035	0.61991	1.791759
1	0	26.16	0.034946	0.60633	1.94591
1	0	26.17	0.051704	0.27631	2.079442
1	0	25.96	-0.05066	0.15216	2.197225
2	0	56.4678	0.17135	1.992	0
2	0	42.176	0.062924	1.62375	0
2	0	42.176	0.064508	1.72651	0.693147
2	0	42.176	0.064674	1.487	1.098612
2	0	42.176	0.06532	2.13718	1.386294
2	0	42.176	0.044986	2.00696	1.609438
2	0	35.0682	0.026165	3.57857	1.791759
2	0	28.1114	0.038635	2.44504	1.94591
2	0	28.1114	0.039991	1.87708	2.079442
2	0	28.1114	0.018865	1.90407	2.197225
2	0	42.958	0.118966	3	0
2	0	32.221	0.060348	2.4426	0
2	0	33.94	0.071747	2.15289	0.693147
2	0	33.94	0.085994	2.35165	1.098612
2	0	33.94	0.090356	1.10405	1.386294
2	0	31.11	0.050599	1.80639	1.609438
2	0	28.47	0.040225	1.30614	1.791759
2	0	28.47	0.024705	1.14271	1.94591
1	0	28.46	0.012388	0.94132	2.079442
1	0	28.46	0.011652	0.82002	2.197225
0	1	58.12	0.091659	1.28875	0
0	1	57.09	0.12305	1.53027	0.693147
0	1	54.64	0.108321	1.89048	1.098612
0	1	53.9	0.113009	1.00248	1.386294
0	1	44.79	0.089045	1.94038	1.609438
0	1	36.36	0.078436	2.21809	1.791759
0	1	33.34	0.071459	1.86544	1.94591
0	1	33.57	0.005698	1.41134	2.079442

2	0	34.06	0.068954	2	0
2	0	16.65	0.031352	1.60812	0
2	0	16.65	0.021663	1.55824	0.693147
2	0	16.65	0.009043	1.52775	1.098612
2	0	16.65	0.01076	1.50847	1.386294
2	0	16.65	0.015516	1.67216	1.609438
2	0	13.76	0.020976	1.32391	1.791759
2	0	13.76	0.022374	0.94111	1.94591
2	0	13.76	0.023203	0.83766	2.079442
2	0	10.32	0.020488	1.17511	2.197225
1	1	58.3203	0.034683	7.65235	0
1	1	58.3203	0.044048	6.38089	0.693147
1	1	59.94	0.039203	6.92656	1.098612
1	1	60.2316	0.034974	6.68688	1.386294
1	1	62.67	0.047664	7.13817	1.609438
1	1	62.55	0.05663	6.66005	1.791759
1	1	62.56	0.045541	6.5109	1.94591
1	1	62.59	-0.05198	6.50503	2.079442
1	1	61.34	0.019226	6.35794	2.197225
1	1	44.11	0.074926	1.93856	0
1	1	39.67	0.084735	2.26364	0.693147
1	1	39.67	0.060147	2.09411	1.098612
1	1	38.97	0.093895	2.31017	1.386294
1	1	27.28	0.069229	2.03154	1.609438
1	1	23.15	0.068047	0.80297	1.791759
1	1	23.45	0.08429	1.10109	1.94591
1	1	22.83	0.087713	2.55185	2.079442
1	0	36.13	0.045493	7.99958	0
1	0	35.2519	0.060063	7.57877	0.693147
1	0	35.2092	0.02415	7.50868	1.098612
1	0	37.241	0.050064	6.48871	1.386294
1	0	42.1233	0.045759	6.07429	1.609438
1	0	35.9273	0.020926	6.58142	1.791759
1	0	32.4238	0.023481	5.47155	1.94591
1	0	32.4238	0.034246	5.05106	2.079442
1	0	28.5148	0.039644	5.72958	2.197225
1	0	57.06	0.070667	0.77575	0
1	0	57.08	0.072685	0.63883	0.693147
1	0	57.08	0.07392	0.01623	1.098612
1	0	47.08	0.019688	0.10721	1.386294
1	0	47.08	-0.03827	0.12111	1.609438
1	0	47.08	0.008874	0.10296	1.791759
1	0	47.08	-0.01331	0.16258	1.94591
1	0	47.08	0.050018	0.09595	2.079442
1	0	47.08	0.015257	0.15306	2.197225
1	0	46.12	0.05254	0.20206	0
1	0	46.12	0.049593	0.04023	0.693147
1	0	46.12	0.020713	0.21431	1.098612
1	0	46.12	0.025034	0.77809	1.386294
1	0	45.42	0.026444	0.68757	1.609438
1	0	35.76	0.050511	2.33281	1.791759
1	0	35.76	0.06074	1.67619	1.94591
1	0	29.01	-0.08385	2.33486	2.079442
1	0	24	0.061874	2.17191	2.197225
0	1	39.76	0.025436	3.61576	1.94591
0	1	39.96	0.014084	2.78469	2.079442
2	0	56.26	0.065042	2.18685	0
2	0	56.26	0.077513	2.24102	0.693147

2	0	56.26	0.07506	2.0381	1.098612
2	0	56.26	0.096887	2.23886	1.386294
2	0	56.26	0.099098	1.89504	1.609438
2	0	52.32	0.089519	1.92313	1.791759
1	0	50.34	0.064924	1.63495	1.94591
1	0	50.34	0.083629	1.9404	2.079442
1	0	51.46	0.091089	1.8453	2.197225
1	0	32.624	0.074999	1.9493	0
1	0	32.35	0.085164	1.8301	0.693147
1	0	22.66	0.068895	1.14246	1.098612
1	0	22.8	0.031956	0.35618	1.386294
1	0	23.11	0.02559	0.99156	1.609438
2	0	57.4	0.096527	3.53896	0
2	0	57.4	0.153675	3.83578	0.693147
2	0	55.58	0.142176	3.56279	1.609438
2	0	55.58	0.116996	3.65915	1.791759
2	0	55.62	0.020754	2.86102	1.94591
2	0	55.62	-0.05784	2.70536	2.079442
2	0	49.49	0.019907	2.7865	2.197225
2	0	32.8226	0.090706	7.39096	0
2	0	32.8226	0.111823	7.33815	0.693147
2	0	32.8226	0.069815	7.23673	1.098612
2	0	32.8226	0.107775	7.24643	1.386294
2	0	32.8066	0.101126	7.42525	1.609438
2	0	32.567	0.108428	7.12792	1.791759
2	0	32.618	0.099046	6.72467	1.94591
2	0	32.696	0.048359	6.53851	2.079442
2	0	32.7385	0.014658	6.41264	2.197225
1	0	20.226	0.058047	6.43476	0
1	0	13.484	0.052733	5.7454	0.693147
1	0	15.252	0.030898	4.48912	1.098612
1	0	14.764	0.081566	3.9447	1.386294
1	0	14.57	0.158433	1.22814	1.609438
2	0	35.43	0.095021	2.09245	1.791759
1	0	44.44	0.049273	3.50785	0
1	0	44.44	0.063855	3.53371	0.693147
1	0	44.44	0.064147	3.96134	1.098612
1	0	44.44	0.023746	2.92533	1.386294
1	0	44.85	0.007002	3.14127	1.609438
1	0	36.79	0.019049	3.54777	1.791759
1	0	34.84	0.033334	2.70597	1.94591
1	0	34.84	0.03481	2.53411	2.079442
1	0	34.84	0.045828	2.72404	2.197225
0	1	34.16	0.064811	1.4829	0
0	1	33.107	0.07449	0.33413	0.693147
0	1	32.94	0.068553	0.23811	1.098612
0	1	32.111	0.078062	0.0009	1.386294
0	1	32.18	0.012076	0.41184	1.609438
0	1	32.73	0.011399	0.65632	1.791759
0	1	33.23	-0.07675	0.38677	1.94591
2	0	59.55	0.092604	1.72393	0
2	0	59.77	0.071151	1.34926	0.693147
2	0	59.77	0.058003	2.24048	1.098612
2	0	59.77	0.063256	0.30779	1.386294
2	0	39	0.034412	2.07074	1.609438
2	0	39	0.016779	0.43182	1.791759
2	0	39	0.03473	0.32065	1.94591
2	0	40.69	0.043491	0.07547	2.079442

2	0	40.69	-0.03256	0.03919	2.197225
1	0	54.85	0.050118	0.4018	0
1	0	54.85	0.034336	0.4169	0.693147
1	0	54.85	0.016438	0.85349	1.098612
1	0	54.85	0.013627	0.96195	1.386294
1	0	49.87	-0.03297	0.89649	1.609438
1	0	50.42	0.012291	0.81534	1.791759
1	0	49.64	0.022085	0.38734	2.079442
1	0	46.75	0.041312	0.90794	2.197225
2	0	36.59	0.064303	2.0451	0
2	0	36.59	0.067877	1.98674	0.693147
2	0	36.69	0.076682	1.88743	1.098612
2	0	36.69	0.085713	2.31811	1.386294
2	0	29.54	0.05885	1.00931	1.609438
2	0	19.54	0.0517	2.55766	1.791759
2	0	19.54	0.032118	2.96816	1.94591
0	2	30.9	0.088536	1.17205	2.079442
0	2	30.9	0.060462	0.69739	2.197225
1	0	30.144	0.040045	6.13297	0
1	0	30.144	0.02831	6.41239	0.693147
1	0	30.1441	0.040993	5.41525	1.098612
1	0	28.643	0.060887	5.50522	1.386294
1	0	28.5711	0.08476	4.74522	1.609438
1	0	28.5711	0.069232	5.17658	1.791759
1	0	20.8716	0.059189	6.58289	1.94591
1	0	20.8716	0.053402	6.52094	2.079442
1	0	16.2662	0.038268	5.9058	2.197225
2	0	37.7909	0.096957	7.9996	0
2	0	37.7909	0.091872	8.0475	0.693147
2	0	33.9661	0.046391	6.00742	1.098612
2	0	26.2787	0.064819	6.23212	1.386294
2	0	26.5757	0.035729	5.79495	1.609438
2	0	23.4455	0.036275	6.07237	1.791759
2	0	24.2455	0.047375	5.06607	1.94591
2	0	29.9888	0.004004	4.73219	2.079442
2	0	28.3533	0.113261	4.62568	2.197225
1	0	38.5	0.081487	5.6641	0
1	0	38.5	0.068325	3.99805	0.693147
1	0	38.11	0.070869	5.21829	1.098612
1	0	33.14	0.080872	6.05976	1.386294
1	0	29.97	0.077417	5.1271	1.609438
1	0	20.96	0.029512	4.74763	1.791759
1	0	24.38	0.019501	3.99549	1.94591
2	1	37.75	0.065334	4.8995	0
2	1	37.75	0.072917	4.49371	0.693147
2	1	37.75	0.082062	4.52265	1.098612
2	1	37.75	0.009213	5.21638	1.386294
2	1	34.07	0.00755	5.42131	1.609438
2	1	35.24	0.023078	4.86018	1.791759
2	1	35.24	0.023073	4.33756	1.94591
2	1	31.8762	0.028344	4.33562	2.079442
2	1	28.2868	0.027091	4.60225	2.197225
1	0	8.1111	0.06998	3.94046	0
1	0	8.3641	0.006941	3.008	1.098612
2	0	36.19	0.078077	2.02463	0
2	0	36.19	0.030662	1.87547	0.693147
2	0	27.74	0.02452	1.042	1.098612
2	0	27.74	0.024615	0.24604	1.386294

2	0	21.15	0.021379	1.3768	1.609438
2	0	17.56	0.027086	0.45602	1.791759
2	0	17.56	0.021964	0.57103	1.94591
0	1	49.74	0.066157	1.346	0
0	1	49.74	0.059466	1.276	0.693147
0	1	49.74	0.026171	1.276	1.098612
0	1	44.32	0.019331	1.62735	1.386294
0	1	34.98	0.017255	1.24658	1.609438
0	1	34.98	0.018001	1.25968	1.791759
0	1	34.5	0.035083	0.67639	1.94591
0	1	40.5	0.012649	0.84124	2.079442
0	1	41.28	-0.00098	0.58566	2.197225
2	0	28.98	0.084978	1.24634	0
2	0	28.98	0.084272	2.5411	0.693147
2	0	28.72	0.105756	3.37446	1.098612
2	0	26.08	0.100689	3.32717	1.386294
2	0	26.07	0.093086	1.98917	1.609438
2	0	24.57	0.157014	1.51083	1.791759
2	0	20.93	0.061885	1.32416	1.94591
2	0	17.1	0.072411	3.53939	2.079442
2	0	16.91	0.077277	2.86387	2.197225
1	1	32.71	0.059685	2.3433	0
1	1	31.83	0.069791	2.85858	0.693147
1	1	31.83	0.070029	3.72913	1.098612
1	1	31.94	0.065689	2.73817	1.386294
1	1	31.94	0.067292	2.38298	1.609438
1	1	38.28	0.056934	1.9466	1.791759
1	1	36.41	0.057312	1.93452	1.94591
1	1	35.69	0.034387	1.82169	2.079442
1	1	35.69	0.056417	1.68979	2.197225
0	2	26.9822	0.082341	6.15846	0
0	2	26.9822	0.094041	6.93549	0.693147
0	2	26.9822	0.091057	5.85588	1.098612
0	2	26.9822	0.079083	6.73811	1.386294
0	2	26.0921	0.077543	5.76236	1.609438
0	2	26.0921	0.093213	6.53358	1.791759
0	2	25.1456	0.104707	5.75539	1.94591
0	2	25.2717	0.092887	5.73621	2.079442
0	2	25.661	0.053646	6.061	2.197225
1	0	62.52	0.07726	1.24631	0
1	0	61.24	0.047455	1.26482	0.693147
1	0	52.4	0.055343	1.09091	1.098612
1	0	52.2	0.055222	1.32254	1.386294
1	0	55.53	0.049793	1.26224	1.609438
1	0	55.36	0.004899	0.54495	1.791759
1	0	49.4	0.015363	0.27938	1.94591
1	0	36.69	0.033481	1.01723	2.079442
1	0	24.72	0.044152	1.48539	2.197225
0	2	50.13	0.066996	6.11673	0
0	2	41.78	0.053437	5.98096	0.693147
0	2	41.78	0.030013	6.06185	1.098612
0	2	41.78	0.052935	4.9201	1.386294
0	2	42.29	0.054963	4.3797	1.609438
0	2	38.78	0.031014	5.27986	1.791759
0	2	37.82	0.038075	4.92888	1.94591
0	1	49.24	0.105373	2.33029	0
0	1	49.24	0.123669	3.07471	0.693147
0	1	49.24	0.133858	2.25563	1.098612

0	1	49.24	0.129432	2.94775	1.386294
0	1	45.82	0.121853	2.58843	1.609438
0	1	45.74	0.143874	2.4355	1.791759
0	1	41.44	0.089999	2.39111	1.94591
0	1	41.44	0.034829	1.75019	2.079442
0	2	52.1177	0.025229	1.13003	2.197225
1	0	39.77	0.081235	1.0521	0
1	0	39.77	0.063633	0.83407	0.693147
1	0	39.77	0.032539	0.00254	1.098612
1	0	39.77	0.036464	0.129	1.386294
1	0	39.77	0.026382	0.20164	1.609438
1	0	39.77	0.016732	0.11773	1.791759
1	0	31.22	0.013933	0.52129	1.94591
1	0	27.17	0.046372	1.21383	2.079442
1	0	28.98	0.04936	1.22218	2.197225
1	0	62.5	0.064261	1.76888	0
1	0	62.5	0.037626	1.3893	0.693147
1	0	62.5	0.002283	1.424	1.098612
1	0	62.5	0.003435	1.34	1.386294
1	0	62.5	0.007586	0.5078	1.609438
2	0	13.0876	0.002704	5.32795	0
2	0	13.0876	0.004225	4.02982	0.693147
2	0	11.4612	0.051801	4.8011	1.098612
2	0	10.335	0.104814	4.11646	1.386294
1	1	45.22	0.068552	1.12205	0
1	1	45.22	0.076716	0.675	0.693147
1	1	45.22	0.060951	0.6985	1.098612
1	1	44.78	0.060189	1.35506	1.386294
1	1	44.42	0.028577	0.40706	1.609438
1	1	44.56	0.027718	0.60386	1.791759
1	1	38.84	0.02299	0.96602	1.94591
1	0	37.26	0.013579	2.59446	2.079442
2	0	57.16	0.012748	0.127	1.94591
2	0	49.63	0.016665	0.17198	2.079442
2	0	60	0.086965	5.36974	0
2	0	60	0.088483	5.12347	0.693147
2	0	58.6	0.088926	4.31544	1.098612
2	0	54.23	0.107388	4.29943	1.386294
2	0	53.01	0.055104	4.42294	1.609438
2	0	37.0942	0.057597	4.14466	1.791759
1	0	68.4318	0.006017	6.84631	1.386294
1	0	68.4318	0.012907	6.89172	1.609438
1	0	68.4318	0.005742	6.87996	1.791759
1	0	68.4318	0.009573	6.79406	1.94591
1	0	52.9618	0.007904	6.32802	2.079442
0	1	68.893	0.176886	6.804	0
0	1	51.408	0.046264	5.59166	0
0	1	37.6848	0.030239	4.06406	0.693147
0	1	51.408	0.033758	4.31928	1.098612
0	1	38.0499	0.044916	6.27467	1.386294
0	1	33.3179	0.052975	4.48039	1.609438
0	1	34.4448	-0.07148	3.96826	1.791759
0	1	34.6243	0.02626	3.25189	1.94591
2	0	64.87	0.042424	0.02633	0
2	0	64.23	0.020975	0.13456	0.693147
2	0	64.45	0.029117	0.2203	1.098612
2	0	57.66	0.043256	0.24437	1.386294
2	0	41.58	-0.00203	1.10391	1.609438

2	0	41.58	0.026265	1.16829	1.791759
1	0	25.38	0.091539	1.86102	0
1	0	25.38	0.103722	3.18349	0.693147
1	0	25.54	0.123191	2.23265	1.098612
1	0	23.13	0.044935	1.21134	1.386294
1	0	22.94	0.053656	1.52251	1.609438
1	0	22.92	0.013589	0.91618	1.791759
1	0	22.5	0.047023	0.44901	1.94591
1	0	22.4	0.07423	1.04375	2.079442
1	0	24.9154	0.052821	7.576	0
1	0	24.9154	0.019244	7.119	0.693147
1	0	24.9154	0.012452	6.918	1.098612
1	0	33.2864	0.008723	6.77961	1.386294
1	0	33.2864	0.015905	5.14832	1.609438
1	0	18.3629	0.077116	5.42276	1.94591
1	0	18.3629	0.048945	5.62416	2.079442
2	0	65.89	0.079811	1.46596	0
2	0	65.89	0.055064	1.6462	0.693147
2	0	65.89	0.066793	0.74987	1.098612
2	0	65.89	0.033904	0.46451	1.386294
1	0	44.29	0.03708	0.91632	1.791759
2	0	66.06	0.059691	0.06627	1.386294
2	0	66.06	0.074092	0.28305	1.609438
2	0	66.06	0.054228	0.03463	1.791759
2	0	66.06	0.032525	0.01292	1.94591
2	0	66.06	0.064299	0.05479	2.079442
1	0	35.5308	0.093888	6.13582	0
1	0	35.5308	0.023636	6.07815	0.693147
1	0	35.5308	0.033959	6.64854	1.098612
1	0	34.3878	0.047913	6.16091	1.386294
1	0	31.6802	0.044539	6.66851	1.609438
1	0	31.6855	0.061763	6.58503	1.791759
1	1	34.9368	0.089722	6.64941	1.94591
1	1	34.9427	0.079372	6.19636	2.079442
0	2	52.5433	0.005704	4.1721	0
0	2	52.5433	0.017871	4.6793	0.693147
0	2	52.51	0.004445	3.92025	1.098612
0	2	41.51	0.021034	6.7744	1.386294
0	1	30.69	0.03743	7.18112	1.609438
0	1	30.9348	0.031989	6.56453	1.791759
1	1	38.77	0.068764	0.0568	0
1	1	38.77	0.084654	0.03243	0.693147
1	1	38.77	0.082531	0.55404	1.098612
1	1	33.82	0.064047	0.41424	1.386294
1	1	34.06	0.06917	0.94149	1.609438
1	1	34.06	0.086752	0.51232	1.791759
1	1	29.92	0.066382	0.83392	1.94591
1	1	29.92	0.080693	0.85906	2.079442
2	0	48.73	0.048315	7.76665	0
2	0	48.73	0.048742	5.924	0.693147
2	0	48.73	0.038941	6.45717	1.098612
2	0	48.73	0.035171	5.78569	1.386294
2	0	44.66	0.035692	5.40819	1.609438
2	0	44.66	0.040782	4.78541	1.791759
2	0	44.26	0.048423	4.6174	1.94591
2	0	44.27	0.041271	4.57024	2.079442
1	0	59.85	0.050478	1.53301	0
1	0	59.85	0.033319	1.15868	0.693147

1	0	59.85	0.021356	0.41554	1.098612
1	0	60.25	-0.07655	0.32406	1.386294
1	0	52.35	0.002722	0.76028	1.609438
1	0	52.35	0.00274	0.65314	1.791759
1	0	52.35	-0.02429	0.3936	1.94591
1	0	30.35	0.009635	2.49312	2.079442
2	0	61.19	0.071413	0.8673	0
2	0	61.19	0.056148	0.61722	0.693147
2	0	61.19	0.05449	0.204	1.098612
2	0	61.35	0.00199	0.42857	1.386294
2	0	61.53	0.00403	1.41681	1.609438
2	0	32.32	0.055444	2.68692	1.791759
2	0	29.12	0.034975	1.23552	1.94591
1	0	56.5451	0.099917	6.8342	0
1	0	56.5451	0.117972	6.12792	0.693147
1	0	56.5451	0.100497	6.22138	1.098612
1	0	56.7351	0.119708	7.09009	1.386294
1	0	52.2331	0.112514	6.69461	1.609438
1	0	51.6366	0.107877	6.03369	1.791759
1	0	53.0166	0.051059	5.55653	1.94591
1	0	33.59	0.061555	2.78627	0
1	0	33.59	0.027432	2.53097	0.693147
1	0	35.13	0.014107	2.98884	1.098612
1	0	37.4	0.023201	1.49006	1.386294
1	0	27.56	0.00865	1.89994	1.609438
1	0	27.56	-0.05462	0.81333	1.791759
1	0	14.81	0.081869	2.44511	0
0	1	42.11	0.071707	0.0866	0
0	1	42.11	0.045676	0	0.693147
0	1	42.11	0.041853	0.044	1.098612
0	1	24.62	0.041013	2.10894	1.386294
0	1	24.62	0.051825	1.89803	1.609438
0	1	22.47	0.030992	1.29289	1.791759
2	0	66.351	0.055646	2.28372	0
2	0	66.351	0.06423	2.56604	0.693147
2	0	66.351	0.098185	2.03416	1.098612
2	0	66.351	0.08768	2.43323	1.386294
2	0	51.639	0.050277	2.23665	1.609438
2	0	52.189	0.048771	1.21911	1.791759
0	2	64.7224	0.091116	0.9616	0
0	2	64.7224	0.04934	0.11	0.693147
0	2	64.7224	0.0206	0.57096	1.098612
0	2	64.7224	0.022361	1.16851	1.386294
0	2	59.97	0.005206	1.23571	1.609438
0	2	59.12	0.011423	0.24027	1.791759
0	2	57.95	0.045256	0.18397	1.94591
0	2	56.49	0.071594	0.23448	2.079442
2	0	37.49	0.024266	1.467	0
2	0	37.49	0.021041	1.468	0
1	0	37.5	0.014031	2.72736	0.693147
1	0	32.38	0.031422	3.42285	1.098612
1	0	32.26	0.013532	3.11464	1.386294
1	0	50.01	0.115227	0.649	0
1	0	35.83	0.10715	0.60317	0
1	0	35.75	0.087884	0.92826	0.693147
1	0	35.77	0.100388	1.44074	1.098612
1	0	35.34	0.063805	0.70653	1.386294
1	0	35.93	0.046278	0.46737	1.609438

1	0	29.34	0.048731	1.66295	1.791759
2	0	58.752	0.068589	7.40624	0
2	0	58.752	0.062804	7.89771	0.693147
2	0	51.093	0.051303	7.67725	1.098612
2	0	50.544	0.066394	7.49398	1.386294
2	0	50.571	0.048502	7.37923	1.609438
2	0	50.436	0.033336	7.48169	1.791759
1	0	30.7	0.153592	3.108	0
1	0	20.56	0.058837	2.80124	0
1	0	20.56	0.076593	4.02833	0.693147
1	0	17.72	0.056696	1.49884	1.098612
1	0	35.42	0.041859	4.67374	1.386294
1	0	34.53	0.060067	5.27562	1.609438
1	0	33.17	0.065089	5.46716	1.791759
0	2	28.1353	0.020517	7.40588	0
0	2	26.746	0.020489	6.72243	0.693147
0	2	24.3801	0.02714	6.41489	1.098612
0	2	23.9848	0.02566	6.64025	1.386294
0	2	22.8629	0.03063	6.39201	1.609438
0	2	20.3407	0.023874	6.8366	1.791759
0	2	50.93	0.102113	0.58498	0
0	2	44.16	0.049979	1.12575	0.693147
0	2	44.16	0.017338	0.63305	1.098612
0	2	44.16	0.03353	0.26887	1.386294
0	2	44.16	0.046928	0.27648	1.609438
0	2	44.16	0.056549	0.08299	1.791759
0	3	32.8414	0.04919	5.14389	0
0	3	33.2746	0.015326	5.89647	0.693147
0	3	32.8414	0.049904	5.40576	1.098612
0	1	35.5311	0.09349	4.59995	1.386294
0	1	35.017	0.186727	4.29004	1.609438
0	1	35.017	0.165275	4.1003	1.791759
1	0	49.95	0.027149	0.60847	0
1	0	49.95	-0.02236	1.3669	0.693147
1	0	43.46	0.009362	1.20226	1.098612
1	0	43.46	0.012361	0.99299	1.386294
1	0	49.76	-0.05796	0.77348	1.609438
1	0	41.91	0.005821	1.23496	1.791759
1	1	40.9587	0.058692	7.51817	0
1	1	38.5095	0.050569	7.03026	0.693147
1	1	33.9156	0.047031	7.06418	1.098612
1	1	47.3536	0.070095	6.04376	1.386294
1	1	22.9245	0.005897	5.56835	1.609438
1	0	32.28	0.047592	1.07496	0
1	0	32.28	0.051107	1.03987	0.693147
1	0	32.28	0.048369	0.16529	1.098612
1	0	26.25	0.018513	1.82756	1.386294
2	0	32.5687	0.093947	4.85194	0
2	0	32.5687	0.095641	4.90813	0.693147
2	0	29.5076	0.059089	4.80351	1.098612
2	0	29.4071	0.039641	4.70857	1.386294
2	0	26.5058	0.033651	4.17129	1.609438
2	0	23.9456	0.038881	3.35363	1.791759
0	1	41.4809	0.100728	6.33789	0
2	0	45	0.059434	9.87	0
2	0	33.748	0.053659	7.60367	0
2	0	24.4681	0.062739	7.5704	0.693147
2	0	33.75	0.118884	7.82193	1.098612

2	0	33.75	0.068276	4.78325	1.386294
2	0	33.75	0.068495	4.33793	1.609438
1	0	54.86	0.094051	0.5	0
1	0	36.27	0.015325	0.86691	0
1	0	34.44	0.04855	1.3433	0.693147
1	0	32.68	0.03527	0.6513	1.098612
1	0	27.81	0.058695	1.03515	1.386294
1	0	26.03	0.054576	0.44519	1.609438
1	0	41.52	0.071892	1.18377	0
1	0	41.52	0.053907	1.34284	0.693147
1	0	41.38	0.053961	0.9556	1.098612
1	0	36.75	0.02296	1.19115	1.386294
1	0	34.25	0.026592	0.93179	1.609438
2	0	57.71	0.099437	1.74424	0
2	0	57.71	0.094749	0.74961	0.693147
2	0	46.44	0.04316	1.99904	1.098612
2	0	48.76	0.071177	2.03701	1.386294
2	0	37.31	0.053144	1.8421	1.609438
2	0	51.27	0.131721	2.59707	0
2	0	49.31	0.105975	1.48635	0.693147
2	0	49.31	0.097645	0.47074	1.098612
2	0	46.76	0.072712	1.40015	1.386294
2	0	43.82	0.070195	0.25525	1.609438
1	0	30.4798	0.059038	3.17355	0
1	0	30.4798	0.057128	3.66606	0.693147
1	0	26.335	0.04684	3.74406	1.098612
1	0	26.409	0.04893	3.70965	1.386294
1	0	26.409	0.03548	2.77913	1.609438
1	0	35.245	0.130212	4.45279	0
1	0	31.0221	0.051258	3.41599	0.693147
1	0	31.0221	0.067435	3.41216	1.098612
2	0	50.4824	0.188915	3.60312	1.609438
1	2	63.63	0.099586	5.88281	0
1	2	63.63	0.055787	5.89477	0.693147
1	2	62.52	0.045965	5.7373	1.098612
1	2	62.42	0.04551	5.816	1.386294
1	2	39.35	0.026852	4.34366	1.609438
1	0	47.345	0.050919	6.42057	0
1	0	47.09	0.053225	6.44768	0.693147
1	0	45.4195	0.068333	6.37016	1.098612
1	0	45.9495	0.053801	6.21095	1.386294
1	0	41.5465	-0.04137	5.35189	1.609438
2	0	37.05	0.087588	0.30022	0
2	0	37.05	0.058558	0.18538	0.693147
2	0	37.05	0.06318	0.17206	1.098612
1	1	44.31	0.049436	0.00193	1.386294
1	1	37.81	0.058076	0.77654	1.609438
0	1	43.9096	0.063197	4.35786	0
0	1	29.3711	0.040891	4.2846	0.693147
0	1	30.0311	0.038129	4.05688	1.098612
0	1	24.1184	0.070376	4.75014	1.386294
2	0	57.5176	0.053152	2.35843	0
2	0	55.8578	0.040777	1.93615	0.693147
2	0	55.35	0.014173	1.79148	1.098612
2	0	47.31	0.084033	1.0036	0
2	0	47.31	0.066499	0.59513	0.693147
2	0	47.31	0.056136	0.32374	1.098612
2	0	47.31	0.015765	0.25555	1.386294

2	0	50.1013	0.124089	7.32777	0
2	0	50.1	0.123748	5.77784	0.693147
2	0	49.68	0.116326	5.54912	1.098612
2	0	46.95	0.115757	6.10742	1.386294
2	0	46.95	0.148216	5.52484	1.609438
2	0	44.77	0.040571	1.88325	0
2	0	44.77	0.028088	0.11345	0.693147
2	0	44.77	0.011179	0.07163	1.098612
2	0	46.77	0.006126	0.10729	1.386294
2	0	22.2946	0.044043	4.88761	1.609438
1	1	64.8	0.107583	1.06997	0
1	1	64.8	0.116552	0.36164	0.693147
1	1	64.8	0.114751	0.21511	1.098612
1	1	64.24	0.102574	0.16117	1.386294
1	1	62.84	0.052595	0.10192	1.609438
2	0	35.49	0.038405	3.92486	0
2	0	34.85	-0.0475	1.82625	0.693147
2	0	36.14	0.031413	1.49143	1.098612
2	0	28.57	0.032123	1.18311	1.386294
2	0	22.92	0.038406	1.77382	1.609438
2	0	71.31	0.091633	5.05417	1.098612
2	0	71.43	0.062468	5.05576	1.386294
0	2	38.88	0.087934	0.72354	0
0	2	38.08	0.079217	0.91669	0.693147
0	2	38.02	0.023075	1.23143	1.098612
0	2	32.99	0.127828	1.36978	1.386294
1	2	41.77	0.116853	6.099	0
1	2	60.59	0.051579	4.58238	0
1	2	60.59	0.04294	4.17569	0.693147
1	0	62.997	0.052969	1.58484	0
1	0	62.997	0.047642	1.52851	0.693147
1	0	62.997	0.03376	1.179	1.098612
1	0	61.997	0.036648	0.729	1.386294
2	0	63.8195	0.078964	4.53606	0
2	0	63.819	0.088291	4.48138	0.693147
2	0	62.1469	0.092849	4.53622	1.098612
2	0	63.8269	0.055273	4.48503	1.386294
0	1	50.5199	0.092697	1.59635	0
0	1	50.5199	0.058797	1.62239	0.693147
0	1	50.5199	0.046413	1.74174	1.098612
1	0	31.05	0.066003	4.57743	0
1	0	31.05	0.043554	4.312	0.693147
1	0	30.762	0.057374	3.97824	1.098612
1	0	30.771	0.068531	3.90811	1.386294
2	0	64.4724	0.052566	7.88039	0
2	0	33.68	0.050939	7.56198	0.693147
2	0	65.0234	0.021572	7.14568	1.098612
0	2	50.02	0.1049	7.60946	0
0	2	50.02	0.065171	7.506	0.693147
0	1	50.02	0.109429	7.82021	1.098612
0	1	43.6668	0.029121	5.81401	1.386294
1	0	61.67	0.064658	1.02175	0
1	0	61.67	0.052739	0.69819	0.693147
1	0	61.67	0.042975	0.50598	1.098612
1	0	61.67	0.037371	0.15368	1.386294
1	0	69.18	0.072853	0.0619	0
1	0	69.18	0.041495	0.00003	0.693147
1	0	69.18	0.010441	0.00491	1.098612

1	0	65.59	0.009823	0.02543	1.386294
1	0	45.64	0.084184	0.36556	0
1	0	45.02	0.084336	0.34035	0.693147
1	0	45.04	0.098786	1.07489	1.098612
1	0	44.54	0.110348	0.78155	1.386294
1	1	24.52	0.102387	1.40312	0
1	1	23.89	0.124316	1.60755	0.693147
1	1	23.82	0.141203	1.38948	1.098612
1	1	24.41	0.115542	0.89038	1.386294
0	1	50.4733	0.087824	7.54619	0
0	1	55.2017	0.048197	7.42314	0.693147
0	1	53.1419	0.040892	7.80639	1.098612
0	1	52.7003	0.043395	6.86031	1.386294
0	2	51.6637	0.058552	1.54306	0
0	2	51.6637	0.061851	1.581	0.693147
0	2	51.6637	0.06961	1.35305	1.098612
0	2	51.0056	0.075851	1.0531	1.386294
0	1	63.2266	0.092711	6.46296	0
0	1	63.2621	0.093616	6.18036	0.693147
0	1	62.9959	0.090526	6.05147	1.098612
1	0	48.6586	0.078817	6.61391	0
1	0	48.6586	0.069663	6.69647	0.693147
1	0	48.6586	0.084469	6.63081	1.098612
1	0	48.6586	0.060059	6.56502	1.386294
1	1	53.2338	0.042357	4.24106	0
1	1	53.2338	0.048931	3.84415	0.693147
1	1	53.3319	0.016419	3.73822	1.098612
2	0	43.09	0.0748	0.80865	0
2	0	43.09	0.071696	0.80272	0
2	0	42.08	0.046711	0.63649	0.693147
2	0	51.36	0.066362	0.34739	1.098612
2	0	48.3525	0.062549	1.9724	0
2	0	48.3515	0.079215	2.13195	0
2	0	47.371	0.018799	1.32316	0.693147
2	0	47.371	0.005811	0.73563	1.098612
1	0	63.33	0.036651	0.00075	0
1	0	63.33	0.038488	0.00022	0.693147
1	0	63.33	0.023155	0	1.098612
2	1	68.42	0.087066	0.359	0
2	1	68.42	0.079868	0.2702	0
2	1	68.42	0.114171	0.3085	0.693147
2	1	68.42	0.117676	0.29128	1.098612
1	0	64.59	0.127487	0	0
1	0	48.45	0.093975	0.87654	0
1	0	48.45	0.089209	0.0048	0.693147
1	0	48.45	0.096021	0	1.098612
1	0	41.7463	0.00388	3.52513	1.098612
2	0	41.97	0.031704	6.093	1.098612
1	0	44.87	0.105887	4.93926	0
1	0	44.4	0.106159	4.3563	0.693147
1	0	44.41	0.077239	4.33003	1.098612
1	1	46.64	0.050842	0.68524	0
2	0	43.395	0.100499	7.37029	0
2	0	43.2893	0.171737	6.2504	0.693147
2	0	43.3087	0.091971	6.00015	1.098612
1	0	19.4511	0.085789	6.67884	0
1	0	19.5079	0.089832	6.38379	0.693147
1	0	16.9012	0.105597	6.83605	1.098612

0	1	41.4718	0.073599	6.09552	0
0	1	41.4718	0.061151	6.05622	0.693147
0	1	41.4718	0.020842	5.66048	1.098612
1	1	29.0506	0.02279	3.00941	0
1	1	29.1287	0.028289	2.97601	0.693147
2	0	64.57	0.051785	1.23126	0
2	0	64.76	0.038245	0.84621	0.693147
2	0	64.76	0.047263	0.27402	1.098612
0	2	51.485	0.08358	4.88785	0
0	2	53.3652	0.058401	4.877	0.693147
0	2	53.3652	0.042886	4.91856	1.098612
1	0	30.7866	0.049489	5.83229	0
1	0	30.7866	0.049948	5.64371	0.693147
1	0	30.7866	0.046649	5.14301	1.098612
1	0	27.93	0.092064	0.84619	0
1	0	27.93	0.088733	1.52906	0.693147
1	0	27.93	0.05766	0.743	1.098612
2	2	68.27	0.08868	1.06169	0
2	2	68.27	0.033098	0.76729	0.693147
2	2	67.5	0.053221	0.43961	1.098612
2	0	66.5301	0.07612	7.76674	0
2	0	53.5142	0.066602	7.36135	0.693147
2	0	65.3388	0.071686	7.48659	1.098612
1	0	40.75	0.039566	1.51565	0
1	0	37.28	0.006729	0.80587	0.693147
1	0	37.28	0.030408	0.41655	1.098612
2	0	51.08	0.073974	5.90121	0
2	0	51.08	0.042436	5.54085	0.693147
2	0	51.08	0.028203	4.50058	1.098612
2	0	56.03	0.098689	1.5535	0
2	0	55.6	0.156134	1.96771	0.693147
2	0	55.6	0.118918	1.64574	1.098612
0	1	38.5706	0.087851	4.83231	0
0	1	38.5706	0.083381	4.82322	0.693147
0	1	38.5706	0.065326	4.92466	1.098612
0	2	38.09	0.104378	1.86513	0
0	2	38.09	0.080841	1.69802	0.693147
0	2	38.09	0.117537	1.41086	1.098612
0	1	31.6253	0.04003	5.60018	0
0	1	31.6253	0.05263	5.34848	0.693147
0	1	32.0581	0.048084	0.15244	0
0	1	32.0581	0.006761	0.1501	0.693147
0	1	32.0581	0.014121	0.15021	1.098612
2	0	29.9616	0.074683	7.52635	0
2	0	29.7781	0.104979	7.70929	0.693147
2	0	29.7781	0.128439	6.97513	1.098612
0	1	30.5985	0.097993	1.69502	0
0	1	30.693	0.022507	1.82243	0.693147
0	1	30.4233	0.07813	2.39176	1.098612
0	1	44.09	0.071109	1.63307	0
0	1	44.09	0.020953	1.43812	0.693147
0	1	44.09	0.02141	0.80079	1.098612
0	1	40.238	0.066959	4.59734	0
0	1	39.7188	0.071552	4.53973	0.693147
0	1	40.1178	0.066078	4.75355	1.098612
1	0	30.6984	0.076479	7.38903	0
1	0	30.6984	0.078164	7.33209	0.693147
1	0	30.6984	0.078727	6.57806	1.098612

0	1	40.5	0.11127	0.49498	0
0	1	40.5	0.119336	0.46571	0.693147
0	1	55.5	0.097803	0.54028	1.098612
1	0	38.281	0.080656	1.2006	0
1	0	38.0852	0.047656	1.01466	0.693147
1	0	37.9372	0.056742	1.26609	1.098612
1	0	44.92	0.050846	1.2372	0
1	0	44.51	0.025854	1.26469	0.693147
1	0	44.68	0.003642	0.403	1.098612
1	1	27.2845	0.0689	2.9061	0
1	1	36.2108	0.062293	3.41498	0.693147
2	0	57.52	0.046293	0.8639	0
2	0	57.7	0.013814	0.24148	0.693147
1	0	39.4623	0.142176	4.198	0
1	0	29.61	0.074876	3.14822	0
2	0	59.1534	0.09855	2.18839	0
1	0	39.2	0.08919	7.5	0
0	2	58.2438	0.074293	3.48694	0
0	2	49.8697	0.097366	2.28	0
1	0	46.4945	0.05987	6.94667	0
0	2	63.7226	0.122133	1.41001	0
0	2	55.2749	0.088255	1.87515	0
0	2	49.9804	0.080074	1.94855	0
0	2	64.8958	0.108371	1.873	0
2	0	35.3692	0.149647	8.61009	1.94591
2	0	36.6057	0.093245	6.38693	2.302585
2	0	35.8672	0.048457	5.88259	2.397895
2	0	32.1167	0.011998	5.46111	2.484907
2	0	29.7991	0.002256	5.21133	2.564949
2	0	29.826	0.002661	5.65277	2.639057
2	0	24.8649	0.038241	5.15731	0
2	0	22.6509	0.085442	5.70988	0.693147
2	0	20.412	0.126953	6.03567	1.098612
0	1	18.9934	0.028835	4.39062	0
0	1	18.9934	0.039802	3.47798	0.693147
0	1	18.9934	0.002779	3.68341	1.098612
0	1	18.9934	0.005778	3.35362	1.386294
0	1	18.4725	0.031874	3.73405	1.609438
0	1	18.4725	0.025659	3.58432	1.791759
0	1	18.4725	0.030277	3.5799	1.94591
0	1	18.4725	0.081906	4.0909	2.079442
0	1	18.2896	0.055435	3.40897	2.197225
0	1	18.2896	0.037154	3.14921	2.302585
2	0	39.2793	0.051537	6.10443	2.564949
2	0	39.2793	0.016179	5.81757	2.639057
2	0	39.2793	0.034019	5.40275	2.70805
2	0	39.2793	0.003664	5.30857	2.772589
2	0	39.2793	0.004948	5.28597	2.833213
2	0	39.2793	0.022959	5.49301	2.890372
2	0	39.2793	-0.11178	5.68615	2.944439
2	0	36.5511	0.079589	4.89835	2.995732
2	0	35.4858	0.035749	4.64191	3.044522
2	0	40.2663	0.01686	4.45424	3.091042
1	0	52.07	0.032067	6.69473	0
1	0	47.39	0.03104	6.78944	0.693147
1	0	25.39	0.043546	4.45402	1.098612
1	0	27.43	0.061168	4.33726	1.386294
1	0	27.43	0.064305	4.02317	1.609438

0	3	26.6047	0.032385	7.18521	2.079442
0	3	24.803	0.059253	6.13381	2.197225
0	3	24.9478	0.088037	5.21153	2.302585
0	3	27.8522	0.058245	5.41381	2.397895
2	0	24.4845	0.081742	4.60732	2.484907
2	0	26.3402	0.045847	5.64632	2.564949
2	0	22.1763	0.022894	4.01095	2.639057
2	0	23.4381	0.018336	4.55062	2.70805
2	0	22.0017	0.034971	4.29133	2.772589
0	3	23.1301	0.035722	4.33334	2.833213
0	3	23.3336	0.021114	5.07814	2.890372
2	0	27.26	0.07356	1.39097	0
2	0	27.38	0.105843	1.66286	0.693147
2	0	27.38	0.095071	1.43365	1.098612
2	0	20.95	0.079572	1.63147	1.386294
0	1	55.86	0.029547	7.16572	0.693147
0	1	55.86	0.037552	7.12729	1.098612
0	1	51.93	0.032215	7.02927	1.386294
0	1	44.75	0.003987	6.07648	1.609438
0	1	44.75	0.005968	5.90833	1.791759
0	1	44.75	-0.0196	5.08203	1.94591
0	1	44.75	0.008928	5.03563	2.079442
0	1	44.75	0.016626	5.264	2.197225
0	1	44.75	0.015535	4.36506	2.302585
0	1	13.3722	0.011579	5.54808	2.484907
0	1	13.3722	0.011907	4.27611	2.564949
0	1	13.1529	0.009884	4.27895	2.639057
0	1	13.1478	0.008534	4.3272	2.70805
0	1	13.1478	0.009947	4.21135	2.772589
0	1	13.1478	0.007887	4.44137	2.833213
0	1	13.1478	0.005307	4.21955	2.890372
0	1	13.1478	0.009845	4.24335	2.944439
0	1	13.1478	0.010912	4.24422	2.995732
0	1	13.1478	0.009587	4.24306	3.044522
1	0	47.25	0.012173	5.06423	1.609438
1	0	42.14	0.028658	6.10192	1.791759
1	0	42.14	0.02476	5.91294	1.94591
1	0	42.14	0.032493	5.99573	2.079442
1	0	42.14	0.030367	5.37647	2.197225
2	0	42.14	0.016028	5.28205	2.302585
1	0	44.2772	0.031298	6.69088	2.397895
2	1	57.67	0.024424	7.51735	1.386294
2	1	57.67	0.027227	7.4928	1.609438
2	1	43.33	0.027326	7.97301	1.791759
2	1	22.27	0.017564	4.23115	1.94591
2	1	18.68	0.020138	4.83423	2.079442
2	1	18.68	0.026075	4.62928	2.197225
2	1	17.03	0.015939	2.68217	2.302585
2	1	17.03	0.023791	2.45191	2.397895
3	0	34.787	0.035695	3.93	2.564949
3	0	30.1834	0.072321	4.37316	2.639057
3	0	30.1834	0.017807	3.93127	2.70805
3	0	31.93	0.007012	3.5413	2.772589
3	0	31.93	0.05336	3.219	2.833213
3	0	31.93	0.086648	3.38459	2.890372
3	0	37.19	0.058827	6.08453	2.944439
1	0	37.19	0.07151	5.92961	2.995732
1	0	36.46	0.071259	5.12015	3.044522

1	0	36.46	0.044804	4.48419	3.091042
1	0	36.69	0.012883	4.5028	3.135494
1	0	42.48	0.058004	6.0252	1.098612
1	0	42.48	0.07442	5.80986	1.386294
1	0	42.48	0.052161	5.83628	1.609438
1	0	42.48	0.016824	5.76521	1.791759
1	0	42.48	0.013735	4.379	1.94591
1	0	42.6799	0.003266	4.965	2.079442
1	0	42.6799	-0.03795	5.54894	2.197225
1	0	42.6799	0.02297	6.27091	2.302585
1	0	42.6799	0.035211	6.71066	2.397895
1	0	42.6799	0.041859	6.5724	2.484907
1	0	42.6799	0.022844	6.54295	2.564949
1	1	20.2	0.035697	1.53177	1.791759
1	1	20.2	0.060904	1.92749	1.94591
1	1	20.2	0.092387	2.04029	2.079442
1	1	18.2418	0.061076	1.90094	2.197225
1	1	17.13	0.05644	0.75918	2.302585
1	1	17.63	0.035118	0.73899	2.397895
1	1	17.76	0.047041	0.68386	2.484907
1	1	18.79	0.036352	1.57857	2.564949
1	1	18.79	0.02658	1.69453	2.639057
1	1	18.79	0.027826	2.04355	2.70805
1	1	19.83	0.043341	1.76121	2.772589
0	2	39.29	-0.08012	4.73058	1.94591
1	0	52.89	0.06229	6.51809	1.791759
1	0	52.89	0.032893	5.52547	1.94591
1	0	52.89	0.014025	5.40992	2.079442
1	0	52.89	0.003526	5.52785	2.197225
1	0	52.5991	0.028074	6.11613	2.302585
1	0	52.5991	0.017977	5.4064	2.397895
1	0	49.9501	0.019043	7.13731	2.484907
1	0	55.2119	0.040549	6.81347	2.564949
1	0	45.8356	0.046139	6.79327	2.639057
1	0	50.5736	0.018658	6.89237	2.70805
1	0	63.3331	0.020184	7.04834	2.772589
3	0	46.97	0.036276	6.058	1.609438
3	0	46.97	0.037115	6.058	1.791759
3	0	46.97	0.045026	6.05993	1.94591
3	0	46.97	0.049008	6.2049	2.079442
3	0	46.97	0.051161	6.64488	2.197225
3	0	46.97	0.056272	6.15091	2.302585
3	0	46.97	0.054675	5.77073	2.397895
0	1	32.5973	0.050335	6.6705	1.098612
0	1	32.1809	0.09892	5.44271	1.386294
0	1	32.1672	0.040703	4.95207	1.609438
0	1	32.5704	0.021976	5.06289	1.791759
0	1	35.0201	0.017894	4.43009	1.94591
0	1	34.6661	0.057086	4.857	2.079442
0	1	34.8942	0.013749	4.9007	2.197225
0	1	34.4849	0.019686	4.45845	2.302585
0	1	34.4849	0.061713	4.55551	2.397895
0	1	34.3388	0.110263	4.50789	2.484907
0	1	35.6246	0.066381	5.49428	2.564949
0	1	8.5426	0.083326	5.81756	2.484907
0	1	8.5529	0.060799	4.23136	2.564949
0	1	9.8073	0.042068	4.75159	2.639057
0	1	9.8096	0.033251	4.41814	2.70805

0	1	34.2393	0.028109	4.18182	2.772589
0	1	34.2393	0.067505	3.83092	2.833213
0	1	34.3298	0.066026	4.07155	2.890372
0	1	31.4315	0.057694	5.01912	2.944439
0	1	31.4315	0.004392	5.23396	2.995732
0	1	33.4227	0.04865	5.60448	3.044522
0	1	33.4126	0.04899	6.03885	3.091042
2	0	26.142	0.015498	4.70529	2.397895
2	0	22.52	0.131685	4.79467	2.484907
2	0	27.3179	-0.03992	4.57307	2.564949
2	0	31.7447	0.100876	3.63887	2.639057
2	0	31.7447	0.06584	3.51088	2.70805
2	0	32.4347	0.159612	3.33928	2.833213
1	0	8.6334	0.086068	2.67812	2.397895
1	0	8.6334	0.076976	2.6129	2.484907
1	0	8.7034	0.05675	2.72213	2.564949
1	0	8.7034	0.019179	2.91704	2.639057
1	0	8.7034	0.023725	2.70538	2.70805
1	0	8.7034	0.016145	3.06762	2.772589
1	0	10.2035	0.010592	2.71231	2.833213
1	0	10.1497	0.021734	2.76928	2.890372
1	0	9.7783	0.05681	2.63151	2.944439
1	0	9.7839	0.043687	2.65525	2.995732
1	0	9.7896	0.048221	2.82962	3.044522
2	0	18.9671	0.034087	3.10553	0
2	0	19.3371	0.02706	2.96166	0.693147
1	0	13.284	0.019181	4.73218	2.772589
1	0	12.918	0.050411	4.56941	2.833213
1	0	13.506	0.024459	5.12016	2.890372
1	0	12.942	0.032565	4.58403	2.944439
1	0	12.942	0.028174	5.85845	2.995732
1	0	29.7346	0.032878	1.56871	2.70805
1	0	29.7346	0.023578	1.51635	2.772589
1	0	29.7346	0.029864	1.46309	2.833213
1	0	29.7346	0.039685	1.47423	2.890372
2	0	29.7345	0.028796	1.35409	2.944439
2	0	27.2462	0.030975	1.35641	2.995732
2	0	31.2206	0.019532	1.50658	3.044522
3	0	28.3338	-0.07805	2.94656	0
3	0	28.62	0.005013	2.94	0.693147
3	0	43.94	0.005309	5.9632	1.386294
3	0	31.29	0.029469	4.884	1.609438
0	1	25.3866	0.015884	4.2028	1.609438
0	1	26.0236	0.004928	4.3161	1.791759
0	1	26.3074	-0.03633	3.949	1.94591
0	1	27.8804	-0.00083	6.33833	2.302585
0	1	30.6006	0.038414	6.24812	2.890372
0	1	34.8481	0.038098	5.93222	2.944439
1	0	18.6705	0.085162	5.31848	1.386294
1	0	17.8524	0.134442	4.47142	1.609438
1	0	17.8516	0.096091	4.03673	1.791759
1	0	18.2412	0.14694	3.30803	1.94591
1	0	18.2412	0.122408	4.84746	2.079442
1	0	18.2412	0.084837	3.37778	2.302585
1	0	21.72	0.061826	3.9056	2.197225
1	0	21.72	0.015427	3.22447	2.302585
1	0	21.72	0.013075	2.22283	2.397895
1	0	21.72	0.112705	2.20597	2.484907

1	0	22.19	0.002372	2.28118	2.564949
1	0	22.19	0.003345	2.92375	2.639057
1	0	21.74	0.004665	3.47376	2.70805
1	0	21.74	0.005713	2.92816	2.772589
1	0	21.74	0.009433	3.01244	2.833213
1	0	21.74	-0.09199	2.80938	2.890372
1	1	31.5453	0.059074	6.44531	2.302585
1	1	31.5453	0.067681	7.42196	2.397895
1	1	31.5453	0.06965	6.94311	2.484907
1	1	27.6737	0.051663	6.2481	2.564949
1	1	27.6737	0.052923	6.78353	2.639057
1	1	36.2743	0.066111	4.24717	2.70805
1	0	42.2064	0.07102	4.85238	2.772589
1	0	42.2064	0.082073	5.13129	2.833213
1	0	42.2099	0.063775	4.91992	2.890372
1	0	42.2099	0.060029	5.02278	2.944439
1	0	42.2099	0.078804	5.38842	2.995732
1	0	46.2014	0.011948	6.84053	1.609438
1	0	46.8402	0.006895	6.76808	1.791759
1	0	46.8402	0.008026	7.01862	1.94591
1	0	69.3102	0.006716	5.04806	2.079442
1	0	70.4102	0.056449	3.79691	2.197225
1	0	71	0.030132	3.81979	2.302585
1	0	56.74	0.007346	2.24336	2.397895
1	0	56.01	0.00736	1.9937	2.484907
1	0	56.01	0.013691	2.13661	2.564949
1	0	56.01	-0.02184	1.90414	2.639057
1	0	56.01	-0.03904	1.74279	2.70805
1	1	10.611	0.024563	2.21135	0
1	1	9.1396	0.032262	3.23422	0.693147
1	1	12.5836	0.063945	2.73501	1.098612
1	1	12.5902	0.030802	2.51176	1.386294
1	1	12.5902	0.049363	3.78787	1.609438
1	1	10.9925	0.116626	5.04019	1.791759
1	1	10.9925	0.068004	4.36636	1.94591
1	1	11.1039	0.079025	4.34427	2.079442
1	1	16.4119	0.088409	4.69265	2.197225
2	0	33.4679	0.050174	5.59933	1.098612
2	0	33.4679	0.049245	5.00906	1.386294
2	0	27.7398	0.015476	5.85096	1.609438
2	0	27.6372	0.009073	4.58067	1.791759
2	0	25.5789	0.009995	4.53032	1.94591
2	0	25.5789	0.019553	4.11779	2.079442
2	0	23.5792	0.008072	5.84441	2.197225
2	0	22.723	-0.00684	5.69235	2.302585
2	0	25.0476	0.008473	5.53825	2.397895
2	0	25.0476	-0.01101	5.66853	2.484907
0	1	8.971	0.028931	5.48608	2.397895
0	1	10.6431	0.019728	5.51267	2.484907
0	1	14.245	0.010971	5.84792	2.564949
0	1	14.245	0.012782	6.20852	2.639057
0	1	10.6431	0.022449	6.13866	2.70805
0	1	10.6431	0.032567	6.28489	2.772589
0	1	19.005	0.031267	7.4109	2.833213
1	0	16.1889	0.032131	2.3723	2.302585
1	0	16.2658	0.037193	3.64199	2.397895
1	0	15.8964	0.022785	3.13117	2.484907
1	0	15.8964	0.019187	2.23782	2.564949

1	0	15.8964	-0.04049	2.90359	2.639057
1	0	23.6848	0.002837	2.51792	2.70805
1	0	22.4541	0.010719	4.50329	2.772589
1	0	20.6127	0.007359	3.06169	2.833213
1	0	15.4959	0.040835	3.00157	2.890372
1	0	19.5723	-0.02059	2.9775	0
1	0	19.5723	0.008726	2.96859	0.693147
0	1	19.76277	0.050491	3.65067	1.386294
0	1	19.76277	-0.08265	3.0979	1.609438
1	0	13.756	0.084867	4.43317	1.386294
1	0	13.756	0.005906	3.75956	1.609438
1	0	13.7678	0.003833	3.66634	1.791759
1	0	13.7678	0.003069	3.66276	1.94591
1	0	13.7678	0.003285	3.56354	2.079442
1	0	13.756	0.003032	3.60558	2.197225
1	0	14.256	0.002932	3.94667	2.302585
1	0	14.256	0.012085	3.97873	2.397895
1	0	14.256	0.047874	5.40681	2.484907
1	0	14.256	0.051193	4.66527	2.564949
1	0	14.256	0.047716	3.85192	2.639057
0	1	21.329	0.016308	3.878	2.197225
0	1	21.329	0.015224	4.384	2.302585
0	1	17.336	0.001416	4.63812	2.397895
0	1	17.256	0.001261	3.553	2.484907
0	1	17.256	0.022864	3.03364	2.564949
0	1	17.256	0.001733	3.20363	2.639057
0	1	25.4915	-0.01673	4.56142	2.70805
2	1	27.8052	0.091821	7.32717	1.609438
2	1	25.0271	0.091489	8.21641	1.791759
2	1	25.0271	0.064807	7.66288	1.94591
2	1	25.0285	0.026317	6.64117	2.079442
2	1	25.0271	0.044245	5.04155	2.197225
1	0	41.085	0.024492	5.94936	2.197225
1	0	36.24	-0.0247	4.60022	2.302585
1	0	26.395	0.016245	3.52316	2.397895
1	0	15.0953	-0.05206	3.46783	2.484907
1	0	15.0953	0.017476	3.15656	2.564949
1	0	16.10985	-0.02691	6.36422	2.639057
1	0	15.26515	0.00425	6.51573	2.70805
1	0	14.5561	0.013697	6.28504	2.772589
1	0	14.5561	0.038184	6.33957	2.833213
1	0	14.5561	0.018079	5.23327	2.944439
0	1	28.1353	0.042758	3.47331	1.609438
0	1	28.1353	0.020771	3.291	1.791759
0	1	45.3318	0.115631	8.48038	1.94591
0	1	41.6145	0.188007	7.93132	2.302585
0	1	42.5256	0.143633	7.40532	2.397895
1	0	11.836	0.035368	3.80255	0
1	0	11.836	0.051839	3.85202	0.693147
1	0	13.6235	0.051283	5.18832	1.098612
1	0	13.6235	0.00643	4.7161	1.386294
1	0	13.8735	0.04574	4.37203	1.609438
1	0	13.8735	0.058849	4.01263	1.791759
1	0	9.467	0.002504	3.62502	1.94591
2	0	34.58	0.073986	2.24271	1.94591
2	0	25	0.063555	2.02843	2.079442
2	0	25	0.046962	1.13964	2.197225
2	0	25	0.047189	0.94268	2.302585

2	0	23.56	0.077091	3.29448	2.397895
2	0	24.56	0.121945	0.70805	2.484907
2	0	26.33	0.108074	1.18229	2.564949
2	0	26.33	0.062157	0.74308	2.639057
2	0	26.82	0.058933	1.32789	2.70805
2	0	25.93	0.080675	0.84387	2.772589
2	0	25.93	0.102867	1.06432	2.833213
1	1	14.57	0.061126	4.06529	2.890372
1	1	15.225	-0.08644	4.05908	2.944439
1	0	7.5627	0.046663	2.95231	1.791759
1	0	10.0827	0.053056	2.83878	1.94591
1	0	17.3847	0.074567	5.45225	2.397895
1	0	15.5793	0.13325	4.29298	2.564949
1	0	15.5793	0.094414	4.4427	2.639057
1	0	15.5793	0.055765	4.05716	2.70805
1	0	14.4137	0.075969	4.51021	2.772589
1	0	14.3872	0.146178	3.87599	2.833213
1	0	14.5814	0.068761	4.5248	2.890372
1	0	14.6477	0.016494	4.64785	2.944439
1	0	14.8375	0.00797	3.84126	2.995732
2	1	7.8105	0.027007	5.064	1.386294
2	1	7.8105	0.043687	5.05	1.609438
2	1	7.8105	0.024432	5.14447	1.791759
2	1	6.9993	0.024299	5.09541	1.94591
2	1	7.007	0.024961	5.11538	2.079442
2	1	7.007	0.019543	5.161	2.197225
2	1	6.9993	0.02085	5.29764	2.302585
2	1	6.9993	0.038002	5.15577	2.397895
2	1	6.9993	0.040227	5.12259	2.484907
2	1	7.6046	0.040899	5.503	2.564949
2	1	7.6046	0.046423	5.472	2.639057
1	0	55.08	0.060677	6.363	1.791759
1	0	55.08	0.061479	6.266	1.94591
1	0	55.08	0.086686	7.00866	2.079442
1	0	55.08	0.086997	6.14462	2.197225
1	0	46.08	-0.03796	5.19495	2.302585
1	0	46.322	0.003302	4.90116	2.397895
1	0	46.322	0.009337	5.20356	2.484907
1	0	46.322	0.075538	5.22677	2.564949
1	0	48.76	0.081464	5.08246	2.639057
1	0	48.76	0.073811	5.12483	2.70805
1	0	48.76	0.082374	4.904	2.772589
2	0	64.57	0.127651	7.48112	2.197225
2	0	64.57	0.141192	7.54845	2.302585
2	0	64.49	0.055709	6.64625	2.397895
2	0	64.49	0.045294	6.6343	2.484907
2	0	64.49	0.053185	6.61536	2.564949
2	0	64.49	0.056586	6.74989	2.639057
1	0	6.2228	0.049152	3.73564	2.70805
1	0	16.0744	0.030799	4.92519	2.772589
1	0	17.0754	0.059784	4.67269	2.833213
1	0	32.8738	0.06275	4.99473	2.890372
1	0	32.8738	0.077141	4.98378	2.944439
1	1	14.984	0.020259	3.51404	2.70805
1	1	14.984	0.008305	3.88826	2.772589
1	1	53.8998	0.128382	9.38161	0
1	1	53.8998	0.126018	9.0385	0.693147
1	1	54.0964	0.128119	9.01062	1.098612

1	1	54.2198	0.132652	8.75517	1.386294
1	1	48.9098	0.116788	8.80272	1.609438
1	1	25.9126	0.109545	8.88616	1.791759
2	1	12.9019	0.006826	4.73107	2.197225
2	1	16.7095	0.014671	4.813	2.302585
2	1	16.7095	0.006655	4.72281	2.397895
2	1	16.8732	0.003236	4.92749	2.484907
2	1	17.1342	0.006276	4.89697	2.564949
2	1	37.502	0.012707	5.17412	2.639057
2	1	44.617	0.013168	7.3698	2.70805
2	1	39.7115	0.017918	6.91369	2.772589
2	1	50.7872	0.111881	7.48066	2.833213
2	1	42.2578	0.037932	7.39103	2.890372
2	1	41.1744	0.031347	7.49925	2.944439
2	1	31.36	-0.06561	1.4534	1.94591
2	1	31.36	0.003429	0.21094	2.079442
2	1	31.49	-0.06029	0.14429	2.197225
2	1	31.57	0.003421	0.21561	2.302585
2	1	31.57	-0.04741	0.07346	2.397895
2	1	31.57	-0.11044	0.04851	2.484907
2	1	31.57	-0.09289	0.06985	2.639057
2	1	31.57	-0.04962	0.117	2.70805
1	0	17.5	0.039722	3.592	1.791759
1	0	17.5	0.013587	4.88744	1.94591
1	0	11.7319	0.053816	5.67289	2.079442
1	0	13.0403	0.069688	6.60382	2.197225
1	0	13.4113	0.089243	7.85661	2.302585
1	0	12.8357	0.093496	8.03692	2.397895
1	0	10.3245	0.081655	6.92402	2.484907
1	0	10.3245	0.101749	4.96272	2.70805
1	0	16.5124	0.087165	6.27663	2.772589
1	0	16.5124	0.075805	5.20753	2.833213
1	0	16.5124	0.0529	4.8708	2.890372
1	0	9.0194	0.04958	4.89762	2.944439
1	0	17.938	0.062913	4.59384	2.995732
1	0	43.98	0.011265	0.2976	2.484907
1	0	43.87	0.022348	1.71707	2.564949
1	0	43.65	0.070089	1.11002	2.639057
1	0	42.1	0.072674	0.45814	2.772589
1	0	41.99	0.05467	0.3133	2.833213
0	1	25.8977	0.007914	3.94821	1.098612
0	1	23.5345	0.008519	2.87592	1.386294
0	1	23.6989	0.001111	2.19983	1.609438
0	1	23.6989	-0.02832	2.49704	1.791759
0	1	23.8253	0.016101	2.59124	1.94591
0	1	23.8253	-0.01709	2.09626	2.079442
0	1	21.2851	0.014959	3.20544	2.197225
0	1	21.2851	-0.04824	3.36601	2.302585
0	1	20.9504	0.006082	3.4996	2.397895
0	1	21.3059	0.000837	2.74172	2.484907
0	1	18	-0.06386	3.56111	2.564949
1	0	7.8246	0.107732	6.8441	1.94591
1	0	7.82	0.047257	6.99253	2.079442
1	0	12.92	0.029788	6.77124	2.197225
1	0	12.9276	0.028669	6.20295	2.302585
1	0	12.16	0.076881	6.48727	2.397895
1	0	12.924	0.04038	5.41693	2.484907
1	0	7.8959	0.075744	5.86484	2.564949

1	0	7.8959	0.033104	5.82799	2.639057
1	0	7.82	0.024254	5.68982	2.70805
1	0	10.0653	0.065882	5.61766	2.772589
1	0	11.3336	0.055641	5.56537	2.833213
1	0	44.23	0.022924	1.30489	1.791759
1	0	44.23	0.019902	1.48584	1.94591
1	0	44.23	0.025523	1.63338	2.079442
1	0	44.23	0.028141	0.99866	2.197225
1	0	44.23	0.012305	0.43091	2.302585
1	2	44.23	0.011657	0.71002	2.397895
1	2	43.92	0.008134	0.53276	2.484907
1	2	35.04	0.012769	1.32003	2.564949
1	0	32.79	0.016392	1.08822	2.639057
1	0	27.06	0.018315	2.44447	2.70805
1	0	27.06	0.017383	2.00539	2.772589
2	0	35.906	0.017185	5.44565	1.791759
2	0	35.906	0.068439	5.443	1.94591
2	0	35.906	-0.0136	5.40749	2.079442
2	0	20.906	0.00943	3.869	2.197225
2	0	20.9078	0.011875	3.91651	2.302585
2	0	20.906	0.009193	4.063	2.397895
2	1	13.7843	0.013001	0.92423	2.484907
2	0	15.7772	0.055735	1.10921	2.564949
2	0	15.7772	0.022809	1.15142	2.639057
2	0	15.78	0.022292	1.06239	2.70805
2	0	15.7772	0.015372	0.99236	2.772589
1	0	14.7954	0.013606	3.98609	2.70805
1	1	15.29	0.013267	0	2.079442
1	1	14.85	0.006454	0.02053	2.197225
1	1	14.72	0.005539	0.00485	2.302585
1	1	14.69	0.004037	0.00253	2.397895
1	1	14.69	0.026501	0.00466	2.484907
1	1	14.68	0.002031	0.00129	2.564949
1	1	11.24	-0.01124	2.4013	2.639057
1	1	11.01	0.007674	3.35433	2.70805
1	1	11.01	0.012949	2.96505	2.772589
1	0	17.09	0.009094	4.57089	0.693147
1	0	17.09	0.007168	4.535	1.098612
1	0	27.0941	-0.08301	4.854	1.386294
1	0	20.688	0.103298	6.03926	1.386294
1	0	20.688	0.080683	5.01829	1.609438
1	0	15.9835	0.054037	4.34434	1.791759
1	0	15.9835	0.056771	3.71023	1.94591
1	0	15.9835	0.061618	2.78911	2.079442
1	0	13.052	0.050121	3.84549	2.197225
1	0	19.123	0.064369	4.652	2.302585
1	0	19.123	0.078009	4.374	2.397895
1	0	16.2825	0.067053	5.11704	2.484907
1	0	16.2825	0.066562	4.11069	2.564949
1	0	16.2825	0.048969	3.96637	2.639057
1	0	17.89	0.079997	0.83872	1.94591
1	0	17.89	0.093739	1.30216	2.079442
1	0	15.67	0.107073	1.65055	2.197225
1	0	15.77	0.125448	2.47258	2.302585
1	0	15.77	0.124345	2.6415	2.397895
1	0	15.77	0.016694	0.91874	2.484907
1	0	15.89	-0.01043	0.40273	2.564949
1	0	15.89	0.015953	2.02281	2.639057

1	0	29.88	0.027277	1.00872	2.70805
1	0	29.88	0.035355	0.21408	2.772589
1	0	29.88	0.033081	0.17441	2.833213
1	0	7.8673	0.080399	7.30017	2.079442
1	0	7.4348	0.084257	7.11743	2.197225
1	0	7.4348	0.096465	7.26425	2.302585
1	0	7.4348	0.10581	7.87279	2.397895
1	0	7.4348	0.114208	7.37488	2.484907
1	0	7.4348	0.112247	6.43848	2.564949
1	0	49.0832	0.098858	6.22128	2.639057
2	0	32.7502	0.071191	6.52357	2.70805
2	0	33.6882	0.065125	6.45388	2.772589
2	0	11.56952	0.063243	6.23899	2.833213
2	0	11.56952	0.041777	5.74984	2.890372
1	0	39.56	0.021952	2.08355	0
1	0	39.56	0.013924	1.93646	1.098612
1	0	39.56	0.021466	1.0967	2.484907
1	0	33.33	0.034442	1.19285	2.564949
1	0	30.361	0.050647	0.57147	2.639057
1	0	22.77	0.007377	0.95908	2.70805
1	0	22.77	0.01027	1.09115	2.772589
1	0	43.1991	0.002561	5.12928	2.302585
0	1	25.1745	0.039512	4.2266	2.079442
0	1	23.9283	0.05597	3.48219	2.197225
0	1	22.9429	0.062629	3.73131	2.302585
0	1	22.7629	0.078459	4.68971	2.397895
0	1	22.788	0.099961	4.88256	2.484907
0	1	22.8206	0.098953	6.00754	2.564949
0	1	22.5677	0.085904	3.64228	2.639057
0	1	22.5677	0.082286	3.15537	2.70805
0	1	19.2768	-0.00285	2.33294	2.833213
0	1	18.3568	0.026945	2.34681	2.890372
1	0	32.0741	0.05456	4.91	1.791759
1	0	28.4611	0.081522	5.68368	1.94591
1	0	28.6096	0.10351	4.60204	2.079442
1	0	34.2468	0.075529	5.54396	2.197225
1	0	33.5364	0.101984	5.94992	2.302585
1	0	33.9624	0.115537	5.30829	2.397895
1	0	32.4624	0.052641	5.10834	2.484907
1	0	32.4624	0.047573	5.06126	2.564949
1	0	27.6467	0.07749	5.09864	2.639057
1	0	26.8385	0.075451	4.66735	2.70805
1	0	24.6385	-0.02754	3.68655	2.772589
0	1	14.6105	0.084832	5.10888	2.079442
0	1	14.8857	0.059947	5.27925	2.197225
0	1	13.6466	0.027006	3.55804	2.302585
0	1	14.8416	0.028416	2.99567	2.397895
0	1	27.8233	0.045736	6.34959	2.484907
0	1	45.9287	0.0486	6.69349	2.564949
0	1	45.9487	0.030901	6.38639	2.639057
0	1	41.797	0.017903	6.23694	2.70805
0	1	41.797	0.040023	6.10156	2.772589
0	1	41.6567	0.037282	6.14225	2.833213
0	1	41.4013	0.051165	5.76864	2.890372
1	1	16.965	0.040737	5.02997	2.197225
1	1	16.635	0.045117	4.26755	2.302585
1	1	16.635	0.028356	4.52249	2.397895
1	1	16.935	0.033851	4.59484	2.484907

1	1	16.935	0.01589	3.85401	2.564949
1	1	17.1675	0.008287	3.85269	2.639057
1	1	17.1675	0.005066	5.59684	2.70805
1	0	30.89	0.019934	3.79999	2.772589
1	0	30.89	0.022819	3.51512	2.833213
1	0	30.89	0.02689	3.23173	2.890372
0	1	23.02	0.096365	2.7609	1.791759
0	1	23.02	0.125808	2.9635	1.94591
0	1	23.02	0.148476	4.10858	2.079442
0	1	24.08	0.148782	4.92482	2.197225
0	1	24.08	0.134654	4.10062	2.302585
0	1	23.15	0.084417	2.95401	2.397895
0	1	23.15	0.031097	2.38799	2.484907
0	1	23.15	0.057911	2.38585	2.564949
0	1	23.15	0.056679	1.27435	2.639057
0	1	23.42	0.02357	0.8733	2.772589
2	0	14.98	0.052216	7.08482	1.609438
2	0	14.98	0.027304	3.58743	1.791759
2	0	14.98	0.003317	3.16547	1.94591
2	0	14.98	0.020045	3.43119	2.079442
2	0	14.98	0.06033	3.16781	2.197225
2	0	14.98	-0.03293	2.92762	2.397895
2	0	14.42	0.057926	2.42184	2.890372
2	0	14.4099	0.123763	2.75677	2.944439
2	0	14.42	0.040402	2.57717	2.995732
2	0	39.73	0.028236	4.34204	1.791759
2	0	39.73	0.078832	4.32576	1.94591
2	0	39.73	0.060199	4.218	2.079442
2	0	41.07	0.080273	7.60354	2.302585
2	0	41.07	0.081722	7.11503	2.397895
2	0	41.07	0.058835	4.98598	2.484907
2	0	41.07	0.038644	4.8885	2.564949
2	0	42.94	0.031486	4.13778	2.639057
2	0	44.81	0.022656	4.14204	2.70805
1	0	19.8096	0.03744	4.667	1.386294
1	0	24.1597	0.033779	4.866	1.609438
1	0	24.1633	0.023274	4.3496	1.791759
1	0	24.1633	0.022154	4.22546	1.94591
1	0	24.1633	0.013094	4.3367	2.079442
1	0	19.0788	0.012042	3.46952	2.197225
1	0	12.9291	0.003733	3.27559	2.302585
2	0	25.08	0.021009	3.29176	2.890372
1	0	15.048	0.012203	3.20474	3.044522
1	0	15.048	0.122542	3.30784	3.091042
1	0	15.048	0.090349	3.39001	3.135494
1	0	15.048	0.009105	5.04839	3.178054
1	0	15.048	0.017493	6.26474	3.218876
1	0	15.048	0.020407	6.33677	3.258097
1	0	15.048	0.022735	6.28713	3.295837
1	0	15.048	0.019509	5.83407	3.332205
3	0	46.4397	0.05435	5.25954	1.609438
3	0	46.4397	0.038001	5.13242	1.791759
3	0	46.4397	0.017038	5.18503	1.94591
3	0	46.7624	0.015385	5.4211	2.079442
3	0	46.7624	0.024315	5.05861	2.197225
3	0	46.7624	0.067097	5.2158	2.302585
1	0	39.11	0.123521	7.45281	2.833213
1	0	35.08	0.169184	5.76761	2.890372

1	1	20.1	0.12386	5.23249	2.944439
1	1	20.1	0.116911	5.36206	2.995732
1	1	20.1	0.131426	4.99967	3.044522
1	0	21.3089	0.13139	5.08687	3.091042
1	0	17.43	0.105027	6.40501	3.135494
1	0	17.43	0.105228	6.86262	3.178054
1	0	17.43	0.099301	6.78772	3.218876
1	0	16.95	0.119081	6.87589	3.258097
1	0	16.95	0.074645	6.52371	3.295837
1	0	16.77	-0.08022	5.86067	0
1	0	24.63	0.008579	5.68062	0.693147
1	0	24.63	0.017826	5.50769	1.098612
1	0	27.25	-0.01387	5.53313	1.386294
1	0	25.35	0.098804	5.21777	1.609438
1	0	29.2	0.014363	5.74222	0
1	0	29.2	0.032042	5.90659	0.693147
1	0	45.62	0.0078	5.36048	1.098612
1	0	45.62	0.03477	6.38439	1.386294
1	0	45.62	0.058425	6.17894	1.609438
1	0	45.62	0.032838	4.90104	1.791759
1	0	52.62	0.037268	5.64296	1.94591
2	0	49.1438	0.087368	8.40636	0.693147
2	0	41.5793	0.071544	7.14477	1.098612
2	0	42.0393	0.112654	6.35415	1.386294
2	0	42.0393	0.070346	7.43302	1.609438
2	0	42.0393	0.077192	8.25967	1.791759
2	0	47.1741	0.088764	6.5891	1.94591
2	0	34.6725	0.084097	6.69912	2.079442
2	0	34.3898	0.091912	6.73058	2.197225
2	0	34.7722	0.125375	7.17539	2.302585
2	0	35.4884	0.091916	7.12322	2.397895
2	0	18.8558	0.043748	6.63986	2.484907
0	1	42.6	0.009714	5.863	1.94591
0	1	41.775	0.011403	5.592	2.079442
0	1	41.775	0.020085	5.57	2.197225
0	1	21.1725	0.00371	5.57	2.302585
0	1	8.3925	-0.03718	2.87996	2.484907
0	1	12.8919	0.100762	5.11149	2.397895
0	1	12.8919	0.098704	5.17097	2.484907
0	1	12.8919	0.093925	4.97905	2.564949
1	0	20.536	0.026437	3.56593	1.791759
1	0	19.608	0.040308	3.55656	1.94591
1	0	13.698	0.043397	2.87999	2.079442
1	0	13.698	0.048794	2.791	2.197225
1	0	27.018	0.062672	4.77307	2.302585
1	0	23.448	0.055191	4.30354	2.397895
1	0	21.018	0.04173	3.91207	2.484907
1	0	21.09	0.025943	4.78748	2.564949
1	0	34.69	0.028685	6.94959	2.639057
2	0	63	0.066205	5.93964	2.772589
1	2	18.01	0.004723	3.61206	2.079442
1	2	18.01	0.033948	4.7446	2.197225
1	2	15.72	0.011113	4.54726	2.302585
1	2	16.72	0.010636	3.11328	2.397895
1	2	16.72	0.006892	2.8399	2.484907
1	2	16.1	-0.02587	3.96261	2.564949
1	2	12.236	0.001749	4.14348	2.639057
0	1	26.3419	0.090041	3.43417	1.609438

0	1	26.3419	0.158868	5.64635	2.197225
0	1	26.3419	0.143521	6.13183	2.302585
0	1	26.3419	0.105212	6.03746	2.397895
0	1	26.3419	0.103295	6.0637	2.484907
0	1	26.3419	0.126321	5.49988	2.564949
2	0	11.5355	0.006672	3.80024	1.94591
2	0	41.8354	0.028691	5.22589	2.079442
2	0	45.8718	0.030589	4.73579	2.197225
2	0	41.1826	0.091944	8.56351	2.302585
2	0	40.894	0.097304	8.46304	2.397895
2	0	41.819	0.077756	8.27322	2.484907
2	0	46.5703	0.031053	8.02924	2.564949
2	0	38.8233	0.032127	7.0256	2.639057
2	0	39.3003	0.059781	6.68355	2.70805
2	0	40.7154	0.048613	6.88775	2.772589
2	0	40.07	0.053818	5.12106	1.94591
2	0	40.71	0.02861	5.11137	2.079442
2	0	40.81	0.032295	4.719	2.197225
2	0	40.92	0.022007	4.764	2.302585
2	0	36.02	0.038507	4.46378	2.397895
2	0	40.92	0.019311	4.72024	2.484907
2	0	40.92	0.045721	5.0167	2.564949
2	0	40.92	0.083064	4.37763	2.639057
2	0	40.92	0.02127	4.2902	2.70805
2	0	40.92	-0.00444	4.224	2.772589
2	0	39.98	-0.08263	4.18763	2.833213
1	1	37.1348	0.013893	8.59417	2.079442
1	1	38.4062	0.039591	8.76455	2.197225
1	1	40.7757	0.037445	9.00055	2.302585
1	1	40.9922	0.037779	8.61894	2.397895
1	1	41.4052	0.037362	7.97927	2.484907
1	1	41.6707	0.044276	7.77084	2.564949
1	1	38.86	0.04467	8.35291	2.639057
1	1	39.8498	0.034251	7.80809	2.70805
1	1	39.9912	0.040403	7.43128	2.772589
1	1	41.0281	0.043632	7.58534	2.833213
1	1	41.5991	0.030114	7.88179	2.890372
0	1	54.151	0.084498	8.33665	1.609438
0	1	54.1591	0.084986	8.45713	1.791759
0	1	51.67	0.094595	8.41041	1.94591
0	1	53.1493	0.066894	7.74582	2.079442
0	1	54.6469	0.088314	6.99319	2.197225
0	1	55.6431	0.083784	6.84001	2.302585
0	1	55.6431	0.026507	7.08694	2.397895
0	1	69.78982	0.036095	8.96229	2.484907
0	1	66.6565	0.061176	8.6063	2.564949
0	1	67.21952	0.067109	8.62416	2.639057
0	1	67.81553	0.072921	8.83804	2.70805
2	0	16.3394	0.012638	2.669	2.772589
2	0	10.1983	0.008224	2.66666	2.833213
2	0	10.1983	0.001891	2.67446	2.890372
2	0	9.5797	0.002936	2.621	2.944439
2	0	9.5797	0.004729	2.621	2.995732
2	0	9.5797	0.009624	2.75155	3.044522
2	0	9.7479	0.007287	3.16185	3.091042
2	0	15.6322	-0.00408	2.83944	3.135494
2	0	15.6438	0.008314	2.70039	3.178054
2	0	16.1066	-0.00587	2.72305	3.218876

2	0	11.5484	-0.0336	2.72304	3.258097
1	0	15.78	0.055405	1.76175	0
1	0	15.78	0.05168	2.15805	0.693147
2	0	15.78	0.044425	2.961	1.386294
2	0	15.78	0.029905	2.911	1.609438
1	0	25.3487	0.056002	4.21635	0
1	0	25.3487	0.090946	4.13334	0.693147
2	0	10.356	0.030061	4.22763	2.772589
2	0	10.356	0.079291	3.87948	2.833213
2	0	10.4224	0.152684	4.19247	2.890372
2	0	22.5399	0.02506	5.23236	2.944439
2	0	18.506	0.066605	6.05189	2.995732
2	0	18.7215	0.093545	4.6571	3.044522
2	0	19.0808	0.129368	5.70401	3.091042
2	0	19.2963	0.136728	6.33371	3.135494
2	0	15.4909	0.176762	6.58494	3.178054
2	0	16.0941	0.153556	6.69914	3.218876
2	0	15.9329	0.151338	5.65868	3.258097
2	0	14.2037	0.00656	6.08062	2.079442
2	0	48.5002	0.036456	8.95746	2.197225
2	0	42.3411	0.030406	8.47154	2.302585
2	0	42.7363	-0.01557	8.44853	2.397895
2	0	35.9168	0.029593	8.35081	2.484907
2	0	35.9168	0.016282	7.88664	2.564949
2	0	34.5881	0.034286	7.37508	2.639057
2	0	34.1873	0.022637	7.08057	2.70805
2	0	39.6817	0.004742	6.99936	2.772589
2	0	38.2091	0.010176	6.52007	2.833213
2	0	36.3767	0.003099	6.3318	2.890372
1	0	38.3024	0.107905	7.93951	0.693147
1	0	34.3132	0.124737	7.62039	1.098612
2	0	23.8679	0.007312	4.267	1.791759
2	0	23.843	-0.05237	3.55107	1.94591
2	0	23.843	0.022889	3.266	2.079442
2	0	23.843	0.027148	3.4725	2.197225
2	0	37.0638	0.077986	4.41172	2.302585
2	0	37.0639	0.05753	4.33473	2.397895
2	0	62.33	0.082449	6.57396	2.484907
2	0	62.33	0.070809	4.87589	2.564949
2	0	62.33	0.064864	4.79077	2.639057
2	0	62.33	0.057166	4.78579	2.70805
2	0	62.33	0.066517	4.89844	2.772589
1	0	10.0642	0.035817	3.86743	1.386294
1	0	12.4574	0.063568	4.87814	1.609438
1	0	10.6648	0.067002	5.05913	1.791759
1	0	11.4595	0.065453	5.65048	1.94591
1	0	12.101	0.066558	4.88469	2.079442
1	0	14.6451	0.0769	5.65973	2.197225
1	0	13.664	0.054304	4.8535	2.302585
1	0	13.6967	0.035991	6.02077	2.397895
1	0	13.6967	0.047523	4.99525	2.484907
1	0	14.3403	0.047207	4.5249	2.564949
1	0	15.9932	0.048033	4.78358	2.639057
1	0	11.0257	0.034372	3.81198	0
1	0	11.0257	0.003615	3.82832	0.693147
1	0	11.0257	-0.00268	3.40726	1.098612
1	0	13.5718	-0.00209	3.464	1.386294
1	0	13.5718	0.018013	3.417	1.609438

1	0	24.5168	0.010788	3.525	1.791759
1	0	12.9656	0.013796	4.09771	1.94591
1	0	13.1127	0.049863	3.5882	2.079442
1	0	10.3005	0.045676	2.97322	2.197225
1	0	10.3904	0.027755	2.41191	2.302585
1	0	37.8345	0.044372	8.41102	0
1	0	40.8175	0.031723	8.48261	0.693147
1	0	40.8175	0.027871	8.76996	1.098612
1	0	41.4115	0.025321	8.59497	1.386294
1	0	35.7996	0.023555	7.84661	1.609438
1	0	35.9061	0.021601	7.97485	1.791759
1	0	26.5374	0.023351	8.11401	1.94591
1	0	26.0414	0.028299	7.26651	2.079442
1	0	26.4616	0.02072	7.33263	2.197225
1	0	23.7008	0.025039	7.05497	2.302585
1	0	59.1996	-0.00801	6.12741	1.098612
1	0	59.2	0.060943	6.48153	1.386294
1	0	58.28	0.018008	6.66412	1.609438
1	0	58.28	-0.00282	6.1359	1.791759
1	0	61.13	0.020422	6.25272	1.94591
1	0	46.13	0.033632	4.73146	2.079442
1	0	41.35	-0.00505	4.94473	2.197225
1	0	41.35	0.019345	4.53954	2.302585
1	0	41.35	0.037949	4.50361	2.397895
1	0	41.35	0.040565	4.45095	2.484907
1	0	41.35	0.03158	4.43036	2.564949
0	2	41.3273	0.046053	5.30455	0
0	2	41.3273	0.014394	5.21075	0.693147
0	2	41.3273	0.001418	4.82044	1.098612
0	2	41.3273	0.011733	4.82595	1.386294
0	2	30.8848	0.011166	5.9999	1.609438
0	2	28.8989	0.009926	3.7586	1.791759
0	2	24.536	0.015214	3.74277	1.94591
0	2	24.536	0.030416	3.45263	2.079442
0	2	24.656	0.004669	3.10313	2.197225
2	0	27.4544	0.01954	6.83793	0
2	0	27.4544	-0.0505	6.52858	0.693147
2	0	34.959	0.021776	6.58436	1.098612
2	0	34.5073	0.022608	7.4794	1.386294
2	0	39.3346	0.031317	7.11729	1.609438
2	0	39.3346	0.004656	6.64522	1.791759
1	0	45.4	0.072232	8.90799	0
1	0	43.111	0.101142	8.34679	0.693147
1	0	43.149	0.055827	7.59341	1.098612
1	0	36.822	0.023625	6.92706	1.386294
2	0	33.054	0.078514	10	0
2	0	24.7905	0.082841	8.99129	0
2	0	24.7727	0.048381	8.63937	0.693147
2	0	24.7727	0.061235	8.72474	1.098612
2	0	21.4201	0.05655	8.07035	1.386294
2	0	21.2664	0.062594	7.87054	1.609438
1	2	67.71	0.05165	0.3701	0
1	2	67.71	0.042388	0	0.693147
1	2	67.15	0.047429	1.46525	1.098612
1	2	54.37	0.028649	1.23593	1.386294
1	2	50.61	0.039433	0.58177	1.609438
1	1	63.05	0.050088	0.00376	0.693147
1	1	63.05	0.041555	0.00044	1.098612

2	0	53.3473	0.095169	8.49951	0
2	0	53.6673	0.072412	8.88786	0.693147
2	0	53.8273	0.057591	8.67913	1.098612
2	0	69.0106	0.06189	8.92941	1.94591
2	0	69.1644	0.115103	9.15328	2.079442
2	0	69.1644	0.154049	8.93023	2.197225
1	0	33.5286	0.032018	7.67261	0
1	0	32.9136	0.046443	7.49384	0.693147
1	0	32.0958	0.019118	7.60786	1.098612
1	0	29.2579	0.002853	7.54284	1.386294
1	0	52.663	0.125366	10	0
1	0	39.5459	0.063445	7.63325	0
1	0	40.0806	0.046982	7.69172	0.693147
1	0	40.0806	0.032251	7.56979	1.098612
1	0	55.1503	0.024965	7.472	1.386294
1	0	55.1503	0.025872	7.57375	1.609438
1	0	46.66	0.046642	7.11634	1.791759
1	0	37.8039	0.061778	6.91935	1.94591
1	0	43.3439	0.063184	7.0136	2.079442
1	0	43.5376	0.058538	7.01835	2.197225
1	0	49.5367	0.035391	6.966	0
1	0	48.6878	0.03059	7.27502	0.693147
1	0	48.695	0.034111	7.69352	1.098612
1	0	47.8452	0.036961	7.83573	1.386294
1	0	47.8452	0.028846	7.43446	1.609438
1	0	24.1579	0.062429	6.63673	0
1	0	24.1579	0.033187	6.67124	0.693147
1	0	25.3381	0.056212	6.40083	1.386294
1	1	23.4238	0.020665	3.19779	0
1	1	24.9327	0.034835	2.83024	0.693147
1	1	24.9327	0.010301	2.83035	1.098612
1	1	25.1412	0.022275	3.15321	1.386294
1	0	43.8476	0.044175	0.72828	0
1	0	43.8476	0.002988	0.44548	0.693147
1	0	44.8581	0.005227	0.01617	1.098612
1	0	36.79	0.024103	0.32023	1.386294
1	0	36.9025	0.032643	0.065	1.609438
1	0	36.6	0.030467	0.04615	1.791759
1	0	36.6	0.000487	0.03216	1.94591
1	0	36.6	-0.0149	0.00705	2.079442
1	0	36.6	0.014402	0.00252	2.197225
2	0	23.2846	0.072838	10	0
2	0	22.9214	0.079564	9.28142	0
2	0	20.5852	0.066547	9.52739	0.693147
2	0	19.3125	0.056869	9.23567	1.098612
2	0	17.6829	0.052989	8.76672	1.386294
2	0	17.9849	0.052339	8.9276	1.609438
2	0	18.4997	0.075682	8.85378	1.791759
2	0	18.4873	0.058555	8.64133	1.94591
2	0	18.5697	0.057508	8.5813	2.079442
1	0	21.03	0.001942	3.18703	1.386294
1	0	37.1	0.0036	0.88577	1.609438
1	0	36.7	0.0052	0.50594	1.791759
1	0	35.1	0.003058	0.32952	1.94591
1	1	41.7977	0.081699	3.77907	0
1	1	41.2504	0.100844	3.2025	0.693147
1	1	41.2504	0.100806	2.97548	1.098612
1	1	42.772	0.115732	2.92049	1.386294

1	1	41.4765	0.093981	3.11124	1.609438
1	1	41.6389	0.078502	3.11587	1.791759
1	1	42.3104	0.054981	2.70919	1.94591
1	1	42.3104	0.053617	2.8851	2.079442
1	1	38.7409	0.052832	2.13636	2.197225
1	0	38.44	0.105367	0	0
1	0	28.93	0.066996	0.36852	0
1	0	28.01	0.075437	0.20243	0.693147
1	0	29.33	0.085977	0.24009	1.098612
1	0	29.33	0.083524	0.41389	1.386294
1	0	29.57	0.060149	0.09345	1.609438
1	0	29.57	0.026909	0.00657	1.791759
0	2	12.3949	0.050443	4.34718	0
0	2	15.9148	0.015709	4.36482	0.693147
0	2	16.3836	0.022202	4.29402	1.098612
0	2	38.73	0.019548	4.53452	1.94591
0	2	38.73	0.023167	4.61734	2.079442
2	1	57.0234	0.045826	7.59782	0
2	1	61.1772	0.0368	7.6635	0.693147
2	1	60.8735	0.033474	7.9049	1.098612
2	1	51.6571	0.015025	7.69566	1.386294
2	1	54.4	0.18563	8.54036	1.098612
2	1	54.4	0.172278	8.16223	1.386294
2	1	64.05	0.102655	7.05332	1.609438
2	1	64.05	0.117508	6.9513	1.791759
2	1	64.05	0.128511	6.96343	1.94591
1	0	10.0615	0.051348	7.5803	0
1	0	10.0615	0.047598	7.46208	0.693147
1	0	10.1922	0.038761	7.1898	1.098612
1	0	10.1922	0.045958	7.24641	1.386294
1	0	10.1922	0.040933	7.54069	1.609438
1	0	10.6441	0.037164	7.27272	1.791759
1	0	38.0652	0.01893	8.462	0
1	0	30.4571	0.019061	7.09441	0
1	1	51	0.113744	5.05	0
1	1	40.67	0.057242	4.42403	0
1	1	33	0.01494	3.45405	0.693147
1	1	33	0.017003	3.47328	1.098612
1	1	33	0.013485	3.26905	1.386294
1	1	16.72	0.003102	3.37896	1.609438
1	1	12.024	-0.04092	3.26776	1.791759
1	1	16.71	0.008557	3.22731	1.94591
1	1	16.71	0.044557	2.65859	2.079442
2	1	25.4999	0.051996	2.284	2.197225
1	1	26.41	0.081427	0.43818	0
1	1	26.41	0.019739	0.31114	0.693147
1	1	26.41	0.020549	0.0452	1.098612
1	1	25.35	0.036478	0.16989	1.386294
1	1	21.94	0.034618	0.84626	1.609438
1	1	20.74	0.048413	0.75536	1.791759
1	1	17.95	0.042614	1.54433	1.94591
1	1	17.96	0.056548	1.5228	2.079442
1	1	17.19	0.083591	1.61746	2.197225
0	2	33	0.088793	2.53825	0
0	2	33	0.143817	2.34587	0.693147
0	2	32.61	0.068301	2.51532	1.098612
0	2	32.61	0.039368	2.29874	1.386294
0	2	30.96	0.050512	0.81719	1.609438

0	2	30.2	0.052158	1.22246	1.791759
0	2	30.13	0.050793	1.2885	1.94591
0	2	24.1	0.105376	1.97215	2.079442
0	2	24.1051	0.066738	1.93765	2.197225
0	2	24.1038	0.040778	1.93039	2.302585
1	1	58.3893	0.040339	0.2673	0
1	1	58.3893	0.047488	0.1418	0.693147
1	1	56.56	0.063606	0.31391	1.098612
1	1	56.56	0.047982	0.46296	1.386294
1	1	56.58	0.051208	0.07719	1.609438
1	1	56.58	0.042153	0.24664	1.791759
1	1	56.58	0.030562	0.20616	1.94591
1	1	56.58	0.006463	0.04455	2.079442
1	1	55.68	-0.06072	0.00409	2.197225
1	2	65.666	0.037446	7.94641	0
1	2	65.6727	0.034789	7.72896	0.693147
1	2	65.6727	0.021747	7.65375	1.098612
1	2	61.3135	0.023212	7.17173	1.386294
1	2	61.659	0.018109	6.48356	1.609438
1	2	49.9	0.013571	6.98834	1.791759
1	2	49.9	0.00313	5.5198	1.94591
1	2	47.9681	0.005183	5.35819	2.079442
1	2	22.78153	0.008847	5.28944	2.197225
0	1	66.02	0.086225	2.0484	1.609438
0	1	57.29	0.065043	2.90628	1.791759
0	1	57.29	0.072621	2.75821	1.94591
0	1	57.11	0.083916	2.90621	2.079442
0	1	57.11	0.092297	3.08365	2.197225
1	2	62.9648	0.044097	9.999	0
1	2	62.468	0.042445	9.18004	0
1	2	64.7596	0.038319	9.09498	0.693147
0	1	24.41	0.058577	2.499	0
0	1	57.39	0.076365	2.4849	0
1	0	25.0717	0.074045	7.20804	1.94591
1	0	26.6634	0.067853	6.87171	2.079442
1	0	21.2471	0.079057	6.89581	2.197225
1	0	18.1494	0.071832	6.95552	2.302585
1	1	50.4025	0.033436	8.26451	0
1	1	50.4025	0.048833	8.26161	0.693147
1	1	50.78	0.048017	8.26349	1.098612
1	1	51.4341	0.034945	8.26195	1.386294
1	0	38.11	0.043199	0.16728	0
1	0	38.11	0.027319	0.10262	0.693147
1	0	38.11	0.022048	0.00179	1.098612
1	0	38.11	-0.00532	0.00189	1.386294
1	0	37.98	0.007246	0.36815	1.609438
1	0	37.83	0.043867	0.27825	1.791759
1	0	37.83	0.012512	0.01772	1.94591
1	0	37.31	0.007198	0.00161	2.079442
1	0	37.31	-0.00632	0.0012	2.197225
1	0	36.81	0.023108	0.00457	2.302585
1	0	59.2691	0.056233	8.21545	0
1	0	59.2691	0.054043	7.91916	0.693147
1	0	61.3881	0.045801	7.93495	1.098612
1	0	59.5423	0.062732	8.28014	1.386294
1	0	53.937	0.047853	7.80662	0
1	0	53.937	0.041332	6.83276	0.693147
1	0	53.94	0.047375	6.24262	1.098612

1	0	53.94	0.042228	5.75036	1.386294
1	0	49.66	0.025708	4.98805	1.609438
1	0	48.61	0.040919	5.03827	1.791759
1	0	48.61	0.052059	4.956	1.94591
1	0	40.52	0.037406	5.60847	2.079442
1	0	40.65	0.041941	5.61714	2.197225
1	0	60.767	0.100741	6.4769	0
1	0	60.767	0.057387	5.77246	0.693147
1	0	60.767	0.054496	5.8666	1.098612
1	0	44.387	0.06907	4.45616	1.386294
1	0	44.557	0.055042	4.24994	1.609438
1	0	44.557	0.042234	4.18734	1.791759
1	0	42.83	0.02794	3.78371	1.94591
1	0	42.83	0.004642	3.93779	2.079442
1	1	28.2052	0.041046	4.16405	2.079442
2	0	66.18	0.035799	5.9024	0
2	0	66.18	-0.04269	6.1771	0.693147
2	0	67.01	0.006097	5.9802	1.098612
2	0	67.82	-0.05566	6.52553	1.386294
2	0	30.69	0.006488	6.27733	1.609438
2	0	30.85	0.011725	5.75712	1.791759
2	0	32.73	0.016435	4.96357	1.94591
2	0	32.73	0.000917	4.47991	2.079442
1	0	51.175	0.090069	5.27681	0
1	0	57.7307	0.104665	7.39594	0.693147
1	0	56.556	0.115675	6.81103	1.098612
1	0	56.5738	0.06874	6.42349	1.386294
1	0	56.7262	0.050724	5.99425	1.609438
1	0	56.8833	0.038818	6.03214	1.791759
0	1	37.4341	0.047743	6.05976	0
0	1	37.4341	0.072699	5.68948	0.693147
0	1	36.5903	0.032931	5.42007	1.098612
0	1	37.0211	0.024794	5.21294	1.386294
1	1	29.908	0.081493	5.54551	0
1	1	29.908	0.079564	6.75593	0.693147
1	1	29.908	0.046876	6.6445	1.098612
1	1	29.908	0.05898	5.36276	1.386294
1	1	37.276	0.046494	6.1461	1.609438
1	1	36.206	0.048786	6.04411	1.791759
1	1	36.15	-0.07073	5.78909	1.94591
1	1	18.424	0.061957	4.9655	2.079442
1	1	52.82	0.049036	0.01319	0
1	1	51.21	0.043634	0.58529	0.693147
1	1	48.2	0.032241	0.77169	1.098612
1	1	48.23	0.036186	0.44204	1.386294
1	1	46.96	0.018786	0.11323	1.609438
1	1	43.62	-0.04472	0.25671	1.791759
3	0	33.9064	0.061641	4.11853	0
3	0	32.1048	0.076602	4.40507	0.693147
3	0	32.1048	0.106257	3.32364	1.098612
3	0	32.1048	0.050351	3.55704	1.386294
3	0	33.2433	0.049831	3.08777	1.609438
3	0	29.0948	0.074508	3.21979	1.791759
1	0	19.6524	0.115294	4.1625	0
1	0	14.7292	0.039154	3.13074	0
1	0	14.7292	0.025542	3.23609	0.693147
1	0	14.7292	0.035281	3.21094	1.098612
1	0	14.2141	0.044441	2.92728	1.386294

1	0	10.0704	0.063189	2.82613	1.609438
1	0	52.44	0.075717	0.64302	0
1	0	52.17	0.051013	0.7821	0.693147
1	0	52.44	0.066249	0.57454	1.098612
1	0	39.33	0.064951	2.06512	1.386294
1	0	39.31	0.028585	1.04019	0
1	0	35.04	0.01931	2.06246	0.693147
1	0	35.04	0.006892	1.49744	1.098612
1	0	35.04	0.003849	1.38844	1.386294
0	2	62.165	0.048816	1.25914	0
0	2	62.165	0.041189	0.51545	0.693147
0	2	62.165	0.031807	0.3911	1.098612
0	2	62.165	0.038207	0.38401	1.386294
0	2	62.165	0.034955	0.38159	1.609438
0	1	45.21	0.154942	0.64628	0
0	1	45.22	0.155598	0.04798	0.693147
0	1	45.22	0.111205	0.00048	1.098612
0	1	45.22	0.077086	0.00676	1.386294
0	1	43.1	0.027726	0.00015	1.609438
1	1	25.5915	0.102054	7.363	0
1	1	27.416	0.054581	7.38644	0
1	1	27.4158	0.00834	6.53574	1.098612
2	0	38.494	0.072305	1.49939	0
2	0	38.494	0.071803	1.50461	0
2	0	39.18	0.07375	1.29718	0.693147
2	0	39.18	0.034253	0.79163	1.098612
1	2	26.34	0.043316	1.6489	0
1	2	26.34	0.049718	0.90566	0.693147
1	0	59.39	0.079631	8.79154	0.693147
1	0	59.39	0.04266	8.67658	1.098612
1	0	45.9	0.110639	8.40055	0
1	0	45.9	0.098517	8.32826	0.693147
1	0	45.69	0.110813	7.81892	1.098612
1	0	46.43	0.119511	7.68751	1.386294
0	2	27.1382	0.024638	6.6955	1.098612
1	0	33.09	0.076235	2.479	0
1	0	33.09	0.068767	2.48007	0
1	0	33.09	0.053063	1.56289	0.693147
2	0	34.6525	0.069845	1.13584	0.693147
2	0	34.6525	0.054285	0.55751	1.098612
2	0	64.8398	0.063452	6.36359	0
2	0	59.0933	0.032317	6.80106	0.693147
2	0	59.0933	0.045946	5.89933	1.098612
2	0	59.6233	0.015725	5.75291	1.386294
2	0	53.7179	0.063506	5.09138	0
2	0	53.7179	0.036665	5.384	0.693147
2	0	53.7179	0.028923	4.96978	1.098612
2	0	53.7179	0.042621	4.98477	1.386294
2	0	53.7179	0.05132	4.55936	1.609438
2	0	53.7179	0.07716	4.5132	1.791759
1	0	70.51	0.068448	5.17139	0
2	0	70.51	0.06699	5.4249	0.693147
2	0	70.51	0.066093	5.11636	1.098612
0	1	59.32	0.076937	1.42847	0
0	1	59.32	0.042507	1.30533	0.693147
0	1	59.32	0.051493	1.02513	1.098612
0	1	59.32	0.043396	0.57033	1.386294
0	1	62.39	0.045338	0.73552	0

0	1	62.39	0.041898	0.75112	0.693147
0	1	62.39	0.016868	0.75761	1.098612
0	1	62.39	0.007288	0.72836	1.386294
2	0	36.2029	0.025829	2.86079	0
2	0	37.11	0.01585	2.84317	0.693147
2	0	24.0202	0.054566	2.10417	0
2	0	27.5496	0.032181	2.10105	0.693147
2	0	27.5496	0.041728	1.74495	1.098612
2	0	27.6796	0.057278	1.55414	1.386294
0	2	50.08	0.128033	5.24249	0
0	2	50.08	0.186927	2.1402	0.693147
0	2	50.08	0.087999	1.379	1.098612
0	1	31.9224	0.021329	4.9716	0
0	1	29.9301	0.018806	4.69691	0.693147
1	1	57.4	0.054489	0.84563	0
1	1	49.9	0.049026	0.97215	0.693147
1	1	49.96	0.01376	0.60112	1.098612
1	1	49.96	0.023922	0.42903	1.386294
2	0	44.27	0.08125	0.54338	0
2	0	44.09	0.094784	1.59023	0.693147
2	0	43.89	0.125693	1.00458	1.098612
2	1	19.062	0.0579	5.43004	0
2	1	18.8838	0.05704	5.36518	0.693147
2	1	54	0.068559	6.97341	1.098612
2	0	71.0052	0.088105	0.73873	0
2	0	71.0052	0.079934	0.69386	0.693147
2	0	64.3568	0.056382	0.7167	1.098612
2	0	42.8184	0.054569	1.05136	1.386294
0	2	51.49	0.099564	7.78524	0
0	2	65.0034	0.099859	7.70715	0.693147
0	2	68.436	0.090856	5.72813	1.098612
0	2	64.746	0.077571	5.383	1.386294
0	2	65.09	0.055296	5.30287	1.609438
1	0	61.79	0.084236	0.06071	0
1	0	61.79	0.081558	0.00391	0.693147
1	0	61.79	0.075752	0.01013	1.098612
1	0	61.79	0.039984	0.0152	1.386294
1	0	64	0.050742	3.52	0
1	0	47.97	0.031651	2.70551	0
1	0	47.97	0.024311	1.65013	0.693147
1	0	38.42	0.15048	1.54242	0
1	0	38.42	0.138069	1.70281	0.693147
1	0	38.36	0.149674	1.63173	1.098612
1	0	38.47	0.07829	1.4483	1.386294
1	0	38.63	-0.07672	1.46292	1.609438
1	0	28.97	0.004944	2.40684	1.791759
1	1	50.99	0.080216	4.26692	0
1	1	50.87	0.072165	4.52416	0.693147
1	1	50.89	0.037482	5.152	1.098612
2	0	46.8225	0.096765	3.43658	0
2	0	46.8625	0.10428	3.51081	0.693147
2	0	46.8975	0.110589	3.70628	1.098612
2	0	68.41	0.115012	0.60012	0
2	0	68.41	0.067081	0.49366	0.693147
0	1	16.8462	0.155275	3.61732	1.098612
0	1	24.7219	0.111808	3.72224	1.386294
0	1	29.3225	0.024791	3.61033	1.609438
1	0	18.1658	0.101055	8.99257	0

1	0	27.9272	0.120925	8.4213	0.693147
1	0	27.8114	0.11325	8.28982	1.098612
1	0	27.9447	0.121363	8.39015	1.386294
1	0	26.1907	0.133673	8.30194	1.609438
0	2	62.0791	0.061268	4.96478	0
0	2	62.0791	0.04296	4.58838	0.693147
0	2	62.0791	0.03549	4.40147	1.098612
0	2	62.0791	0.020257	4.31297	1.386294
0	2	62.0791	0.035229	4.54722	1.609438
2	0	45.3349	0.136854	3.77002	0
2	0	45.3349	0.082884	3.95989	0.693147
2	0	39.7132	0.059155	4.38856	1.098612
1	0	39.715	0.081603	7.57937	0
1	0	39.279	0.092766	7.26537	0.693147
1	0	39.933	0.098976	7.25935	1.098612
1	0	63.9074	0.08151	0.92333	0
1	0	63.9074	0.080417	1.20803	0.693147
1	0	62.87	0.080322	1.2001	1.098612
1	0	65.7625	0.119335	9.58333	0
1	0	49.3225	0.04212	7.1897	0
1	0	49.3219	0.048326	7.27119	0.693147
1	0	49.3219	0.066097	7.22773	1.098612
1	1	48.989	0.054997	9.00813	0
1	1	30.3471	0.109624	8.97478	0
1	1	30.3471	0.177543	9.07398	0.693147
1	1	30.3471	0.064061	7.74152	1.098612
0	1	31.95	0.118098	0.78679	0
0	2	51.82	0.057323	1.33247	0
0	2	52.82	0.04625	0.36009	0.693147
0	2	52	0.045438	0.16315	1.098612
1	0	58.4	0.046655	1.2557	0
1	0	59.42	0.064095	1.26053	0.693147
1	0	59.77	0.03748	1.17206	1.098612
2	0	50.5382	0.088317	4.42712	0
1	0	45.3934	0.059166	1.76055	0
1	0	45.3934	0.034943	1.12578	0.693147
1	0	45.3934	0.034816	0.8887	1.098612
1	0	29.5	0.105036	7.50244	0
1	0	29.14	0.109834	7.22489	0.693147
0	1	50.7	0.062772	6.64453	0
0	1	50.7	0.082708	6.34986	0.693147
0	1	50.7	0.08962	5.86221	1.098612
0	1	50.7	0.064327	5.822	1.386294
0	1	50.7	0.066187	5.71892	1.609438
0	1	20.68	0.058745	0.23444	0
0	1	20.68	0.09919	0.01819	0.693147
0	1	21.31	0.071855	0.22548	1.098612
0	1	21.37	0.051555	0.24799	1.386294
0	1	65.915	0.070921	5.93424	0
0	1	65.915	0.060108	5.79428	0.693147
0	1	65.915	0.037824	5.55213	1.098612
0	2	62.19	0.032701	0.0382	0.693147
0	2	62.19	0.024508	0.02677	1.098612
1	0	48.135	0.113164	0.89	0
1	0	36.101	0.037182	0.668	0
1	0	36.1	0.039069	0.54943	0.693147
1	0	36.101	0.017887	0.443	1.098612
2	1	62.75	0.060428	4.085	0

2	1	61.78	0.065225	3.88594	0.693147
2	1	62.18	0.049246	3.9142	1.098612
0	1	28.2803	0.155383	6.9056	0
0	1	21.2106	0.106021	5.17947	0
0	1	48.8402	0.01701	7.50053	0
0	1	50.1103	0.021227	7.33676	0.693147
0	1	50.1103	0.014756	6.43047	1.098612
1	0	59.78	0.077435	0.13306	0
1	0	59.78	0.07911	0.08445	0.693147
1	0	59.78	0.064211	0.01209	1.098612
1	0	59.78	0.072978	0.0646	1.386294
1	0	58.1263	0.114848	1.79114	0
1	0	58.1279	0.127606	2.30865	0.693147
1	0	53.384	0.101904	3.18319	1.098612
1	0	52.79	0.13223	3.13372	1.386294
1	0	52.5068	0.142831	3.35003	1.609438
0	1	40.41	0.080723	0.12708	0
0	1	39.74	0.094898	0.14818	0.693147
0	1	39.95	0.088836	0.18946	1.098612
0	1	39.96	0.052522	0.00187	1.386294
2	0	68.2	0.043469	1.55013	0
2	0	67.85	0.01807	1.55206	0.693147
2	0	32.32	0.053166	0.00251	0.693147
0	1	56.09	0.057772	0.0651	0
0	1	56.09	0.045417	0.06527	0.693147
0	1	56.09	0.03642	0.30087	1.098612
1	0	47.81	0.062807	1.77162	0
1	0	47.81	0.068876	1.75938	0.693147
1	0	47.81	0.062999	1.7451	1.098612
1	0	71.5395	0.156371	10	0
1	0	53.65	0.128164	7.57058	0
1	0	55.5	0.161654	7.23518	0.693147
1	0	39.015	0.09778	4.29708	0
1	0	39.015	0.103024	4.31014	0.693147
1	0	39.015	0.111531	4.35603	1.098612
0	1	23.4879	0.095849	6.83858	0.693147
0	1	23.4879	0.093028	6.73876	1.098612
0	1	42.7711	0.023094	6.20826	0.693147
0	1	42.768	0.035903	7.06983	1.098612
1	0	61.9	0.120438	2.21626	0
2	0	28.6226	0.062412	3.93251	0
2	0	29.6526	0.058734	3.89562	0.693147
0	2	66.1993	0.074951	7.56777	0
0	2	66.1993	0.057493	7.49955	0.693147
0	2	62.49	0.067143	0.32041	0
0	2	50.56	0.062089	6.07	0
0	2	53.75	0.059874	6.45206	0.693147
0	2	53.75	0.03714	6.16473	1.098612
0	2	53.18	0.04671	5.19643	1.386294
0	2	47.5794	0.047179	4.92692	1.609438
0	2	41.58	0.050739	5.17731	1.791759
1	0	67.9389	0.115289	9.08602	0
1	0	67.909	0.027992	8.75776	0.693147
2	1	69.1974	0.089811	0.78823	0
2	1	69.2416	0.089716	0.56649	0.693147
2	1	69.2549	0.092363	0.42908	1.098612
0	1	61.3	0.043684	0.66448	0
0	1	61.3	0.025149	0.82853	0.693147

0	1	40.1812	0.040356	4.20404	1.098612
0	1	56.26	0.106275	1.67677	0
0	1	56.2506	0.073277	1.49661	0.693147
0	1	56.2506	0.046871	1.41717	1.098612
0	1	56.26	0.066724	1.83613	1.386294
0	2	46.22	0.161711	1.1784	1.609438
1	0	44.81	0.085643	0.73834	0
1	0	44.81	0.080093	1.86886	0.693147
1	0	44.81	0.08819	1.67321	1.098612
1	0	45.23	0.096577	0.86176	1.386294
1	0	33.92	0.109304	2.03482	1.609438
1	1	54.972	0.049697	3.8	0
1	1	41.234	0.040087	3.05485	0
1	1	41.234	0.040442	2.13381	0.693147
1	1	41.234	0.031153	2.65621	1.098612
1	1	41.254	0.05249	3.14299	1.386294
1	2	56.06	0.070007	1.32658	0
1	2	56.06	0.073255	1.30703	0.693147
1	2	56.06	0.06181	1.22329	1.098612
0	1	59.91	0.146215	9.89999	0
2	0	56.6297	0.098853	7.18688	0
2	0	55.6152	0.087127	7.64628	0.693147
2	0	56.8396	0.051508	7.12652	1.098612
1	0	21.0391	0.073904	7.1034	1.386294
1	0	54	0.084553	1.3821	0
1	0	43.72	0.04218	2.58562	0.693147
1	0	40.09	0.040052	2.34456	1.098612
1	0	35.42	0.057028	2.60772	1.386294
1	0	35.42	0.072411	1.51503	1.609438
2	0	67.83	0.097445	8.59197	0
2	0	67.83	0.077373	8.56175	0.693147
2	0	67.83	0.07598	8.49353	1.098612
2	0	68.04	0.056637	8.50156	1.386294
1	0	68.04	0.045001	8.52512	1.609438
1	0	63.75	0.091949	1.13102	0
1	0	62.64	0.094531	0.13108	0.693147
1	0	62.64	0.066125	1.0073	1.098612
1	1	47.502	0.1036	2.93442	0
1	1	48.718	0.115349	2.68728	0.693147
1	1	48.718	0.117074	1.80512	1.098612
2	1	67.69	0.070716	0.731	0
2	1	67.69	0.083831	0.64249	0.693147
2	1	67.7	0.076559	0.30175	1.098612
0	3	45.55	0.135849	1.56869	0
0	3	45.55	0.151378	1.61428	0.693147
0	3	45.55	0.173209	1.31441	1.098612
0	3	45.55	0.142927	1.15777	1.386294
1	0	30.7516	0.061711	5.84213	0
1	1	30.7753	0.030796	5.30556	0.693147
1	1	25.8975	0.072361	5.76508	1.098612
0	1	53.86	0.160872	1.38785	0
0	1	53.86	0.159092	1.65911	0.693147
2	0	45.675	0.090381	4.01596	0
2	0	45.675	0.083835	3.30757	0.693147
1	0	63.3	0.140115	0.7888	0
1	0	63.3	0.079918	0.71685	0.693147
1	0	59.11	-0.07875	1.18823	1.098612
2	0	58.5849	0.042838	7.17746	0

2	0	58.5849	0.053571	7.02333	0.693147
2	0	47.4624	0.0614	5.91547	1.098612
2	0	47.4624	0.079504	6.57171	1.386294
2	0	47.458	0.076629	6.93783	1.609438
1	1	36.0819	0.100215	1.25948	0.693147
1	1	36.126	0.122969	0.91755	1.098612
2	0	30.2162	0.039534	6.22503	0
2	0	28.08	0.028886	5.52598	0.693147
2	0	27.9639	0.0252	5.64364	1.098612
2	0	24.2612	0.01698	6.14919	1.386294
2	0	26.0752	0.050232	6.19782	1.609438
2	0	26.0752	0.114677	5.3361	1.791759
0	1	31.4778	0.113288	3.22258	0
1	0	29.59	0.083046	0.37838	0
1	0	29.59	0.058572	0.74334	0.693147
1	0	27.05	0.056134	0.50603	1.098612
1	0	27.08	0.056441	0.60981	1.386294
1	0	28.12	0.048906	0.65596	1.609438
1	0	69.5599	0.076119	6.88053	0
1	0	64.969	0.081497	6.4437	0.693147
1	0	69.08	0.082236	6.4342	1.098612
2	1	30.3556	0.043286	6.60198	0
2	1	25.1145	0.03647	5.52084	0.693147
2	1	29.2135	0.025549	5.62629	1.098612
2	1	29.2534	0.019824	5.34692	1.386294
2	1	33.716	0.018142	5.21782	1.609438
0	2	52.36	0.064641	1.78622	0
0	2	52.55	0.025087	1.03999	0.693147
0	2	51.66	-0.04144	0.77944	1.098612
0	2	60.41	0.073381	3.57559	0
0	2	60.86	0.064283	3.24436	0.693147
0	2	60.86	0.026147	2.741	1.098612
2	0	62.5	0.142664	3.33908	0
2	0	62.5	0.133424	2.74407	0.693147
2	0	59.29	0.128544	2.81248	1.098612
2	0	65.3716	0.124565	1.50758	0
2	0	64.8209	0.113741	1.08509	0.693147
2	0	64.7246	0.090299	1.01148	1.098612
2	0	60.9425	0.070873	1.45594	0
2	0	60.9425	0.058749	1.32831	0.693147
2	0	60.9425	0.043923	1.03818	1.098612
1	0	64.7067	0.105301	5.30314	0
1	0	64.7067	0.107018	5.34394	0.693147
2	0	70.29	0.064102	1.50549	0
2	0	70.29	0.059879	1.4743	0.693147
2	0	70.29	0.066093	1.15771	1.098612
1	1	51.0436	0.080937	7.54561	0
1	1	51.0436	0.094157	7.45184	0.693147
1	1	50.4563	0.086203	7.16196	1.098612
1	1	52.4963	0.103828	7.14943	1.386294
2	0	59.3055	0.064319	7.51851	0
2	0	59.4555	0.063886	7.75503	0.693147
2	0	59.4555	0.035671	7.53464	1.098612
1	0	36.72	0.054604	1.72821	0
1	0	36.69	0.034495	1.71399	0.693147
1	0	32.3	0.027827	1.39237	1.098612
2	0	48.3332	0.071851	5.32001	0
2	0	48.1682	0.038772	5.59442	0.693147

2	0	48.7869	0.042997	5.23663	1.098612
1	1	51.3	0.092591	2.10677	0
1	1	46.86	0.064971	2.05203	0.693147
1	1	46.86	0.068969	1.87789	1.098612
1	1	47.33	0.089392	1.42614	1.386294
1	1	43.27	0.087563	1.6577	1.609438
2	0	45.26	0.071578	1.58457	0
2	0	45.13	0.082911	1.88657	0.693147
2	0	44.65	0.076005	1.521	1.098612
1	0	66.31	0.059464	2.9132	0
1	0	66.33	0.041926	2.95044	0.693147
1	0	57.43	0.019121	0.85437	0.693147
1	0	57.43	0.00226	0.70269	1.098612
2	0	50.9991	0.077376	7.50166	0.693147
2	0	50.9991	0.080087	7.50311	1.098612
1	1	65.49	0.046516	8.78334	0
1	1	71.25	0.075701	2.13338	0
1	1	71.25	0.054791	2.54182	0.693147
1	1	71.25	0.053931	3.00087	1.098612
1	1	71.25	0.056736	2.09163	1.386294
1	1	55.13	0.067165	1.95259	1.609438
0	2	68.04	0.102686	0.06137	0
0	2	49.59	0.090766	1.37168	0
0	2	48.8	0.094871	0.99614	0.693147
0	2	48.34	0.121454	0.60953	1.098612
0	2	48.46	0.059182	0.2466	1.386294
2	0	52.57	0.031899	4.92981	0
2	0	52.57	0.022728	3.5	0.693147
2	0	53.4	-0.06909	3.45604	1.098612
2	0	53.399	0.01512	3.42796	1.386294
1	0	62.3494	0.069838	2.58859	0
1	0	62.3494	0.079788	2.59231	0.693147
1	0	48.54	0.116605	3.42994	0
1	0	48.53	0.124782	3.39121	0.693147
1	0	48.52	0.119376	3.40571	1.098612
0	2	69.0857	0.094232	1.25503	0.693147
0	2	69.0857	0.088724	1.03179	1.098612
0	1	45.7287	0.070917	3.56598	0
0	1	45.9415	0.076355	3.82023	0.693147
0	1	37.9281	0.073637	8.42973	0
0	1	37.9847	0.047331	7.37968	0.693147
2	0	39.56	0.075559	0.46491	0
2	0	39.56	0.049486	0.47788	0.693147
2	0	39.56	0.05696	0.60241	1.098612
2	0	57.53	0.060646	0.00879	0
2	0	57.53	0.088602	0.05335	0.693147
2	0	57.53	0.079079	0.06799	1.098612
2	0	57.53	0.064558	0.00871	1.386294
1	1	66.5694	0.152738	5.69419	0
1	1	66.4286	0.1085	5.78477	0.693147
1	1	68.0416	0.118877	5.94663	1.098612
1	2	57.0912	0.043675	6.07603	0
1	2	57.0912	0.030508	5.96035	0.693147
1	2	55.6942	0.039285	5.65535	1.098612
2	0	66.2131	0.048433	6.73265	0
2	0	65.2019	0.033774	6.82013	0.693147
2	0	66.4187	0.003558	6.60328	1.098612
2	0	66.8685	0.003618	6.58256	1.386294

0	1	52.64	0.038985	0.23541	0
0	1	47.23	0.033685	1.16312	0.693147
0	1	47.23	0.024509	1.32734	1.098612
2	0	63.77	0.110866	5.60977	0
2	0	63.57	0.132678	5.41562	0.693147
2	0	54.7	0.103749	5.57759	1.098612
2	0	49.64	0.096558	5.75006	1.386294
2	0	51.21	0.082289	5.78118	1.609438
0	2	60	0.070694	4.76315	0
0	2	59.66	0.02894	4.29413	0.693147
0	2	59.69	0.025489	4.16168	1.098612
0	2	60.14	-0.02672	3.88625	1.386294
0	2	60.14	0.008251	3.63468	1.609438
0	1	60.13	0.104903	0.26799	0
0	1	60.13	0.089895	0.16703	0.693147
1	1	69.76	0.022833	7.6067	0
1	1	69.76	0.019399	7.52902	0.693147
1	1	65.36	0.032582	7.07418	1.098612
0	1	47.276	-0.02654	8.00388	0
0	1	43.764	0.005479	6.60751	0.693147
0	1	43.764	0.113469	6.45653	1.098612
0	1	40.274	0.079994	5.05994	1.386294
0	1	40.274	0.004647	6.26426	1.609438
0	1	41.48	0.068595	0.03648	0
0	1	41.48	0.071171	0.01516	0.693147
0	1	41.48	0.051387	0.00698	1.098612
1	0	48.45	0.093066	1.04108	0
1	0	46.69	0.022588	0.976	0.693147
2	0	66.6792	0.109673	8.13324	0
2	0	64.247	0.126638	8.45451	0.693147
1	0	56.4078	0.095862	8.74519	1.098612
1	0	56.6865	0.099548	8.83693	1.386294
1	0	42.46	0.058735	2.52177	0
1	0	42.17	0.034126	2.48804	0.693147
1	0	42.17	0.038736	2.17777	1.098612
1	0	42.38	0.083749	2.07038	1.386294
1	0	35.025	0.08571	7.28746	0
1	0	35.025	0.074747	7.02498	0.693147
1	0	35.075	0.092831	6.94206	1.098612
1	0	35.075	0.092142	6.60077	1.386294
1	0	58.76	0.026051	3.53359	0
1	0	57.2	0.01871	2.88214	0.693147
1	0	56.97	0.032035	2.68634	1.098612
1	0	48.95	0.093682	0.39435	0
1	0	48.95	0.123252	0.93772	0.693147
1	0	50.6315	0.082759	1.33556	1.098612
1	0	55.5604	0.023916	4.78619	0
1	0	55.923	0.017867	4.34112	0.693147
1	0	56.2486	0.017713	4.63978	1.098612
1	0	56.2486	0.017066	4.636	1.386294
1	0	56.7558	0.010303	4.61564	1.609438
1	0	26.1616	0.062074	7.50507	0
1	0	26.1616	0.064326	7.50709	0.693147
1	0	26.1616	0.04766	7.52942	1.098612
4	0	48.13	0.075165	0.88332	0
4	0	48.13	0.076477	0.80011	0.693147
4	0	48.13	0.052971	0.52899	1.098612
4	0	48.13	0.051134	0.49352	1.386294

4	0	36.12	0.048537	0.45297	1.609438
0	1	37.11	0.086641	0.40221	0
0	1	37.39	0.076158	0.73337	0.693147
0	1	37.62	0.082862	0.80723	1.098612
1	0	35.84	0.067649	0.38862	1.098612
1	1	34.6449	0.070419	6.42877	0
1	1	34.6449	0.08189	6.17646	0.693147
1	1	35.1751	0.096448	5.91096	1.098612
0	1	49.79	0.094775	6.78695	0
0	1	49.79	0.085376	7.10952	0.693147
0	1	49.79	0.095256	6.57044	1.098612
0	1	50.26	0.09267	6.20578	1.386294
1	0	70.4	0.058409	1.76135	0
4	1	70.4	0.040518	1.3932	0.693147
4	1	65.05	0.03426	1.72347	1.098612
4	1	63.72	0.022626	1.55526	1.386294
2	0	69.84	0.131769	0.82985	1.609438
1	0	49.4149	0.077036	7.61804	0
1	0	36.5132	0.034644	6.61228	0.693147
1	0	35.0819	0.044908	6.35332	1.098612
1	0	36.0675	0.052852	6.31533	1.386294
1	0	20.337	0.030865	5.49547	1.609438
0	1	45.05	0.033442	6.92165	0
0	2	44.68	0.022708	6.56692	0.693147
0	2	39.76	0.052561	2.1096	0
0	2	39.76	0.049862	2.10742	0.693147
0	2	39.28	0.069054	1.90195	1.098612
0	2	39.28	0.100522	1.74215	1.386294
1	2	36.45	0.076518	0.73614	0
1	2	36.45	0.06231	0.44977	0.693147
1	2	36.67	0.041767	0.51372	1.098612
2	0	44.0022	0.070077	7.61462	0
2	0	44.0022	0.103262	7.48508	0.693147
2	0	44.0055	0.109622	7.84046	1.098612
2	0	44.0055	0.103577	7.1659	1.386294
1	0	62.35	0.032626	6.97873	0
1	0	62.35	-0.03217	6.968	0.693147
1	0	52.55	0.095947	4.62374	0
1	0	51.25	0.090349	4.24181	0.693147
1	0	51.06	0.092405	4.18272	1.098612
1	0	48.81	0.073838	0.7364	0
1	0	48.81	0.078976	0.85867	0.693147
1	0	48.81	0.058034	0.3383	1.098612
1	0	55.28	0.098246	7.50236	0
1	0	55.28	0.108426	7.94792	0.693147
1	0	55.28	0.095767	7.60386	1.098612
1	0	51.57	0.085139	7.84313	1.386294
0	1	45.64	0.0766	3.37612	0
0	1	44.3	0.050724	3.28728	0.693147
0	1	44.51	0.058645	3.32585	1.098612
1	0	14.4488	0.115676	7.64177	0
1	0	14.6186	0.077104	7.82612	0.693147
1	0	14.6266	0.083522	7.13129	1.098612
1	0	14.6266	0.04753	7.02017	1.386294
2	0	40.5	0.072544	3.38839	0
2	0	40.5	0.017999	3.36906	0.693147
2	0	40.5	0.02406	3.225	1.098612
1	0	33.18	0.062048	2.91407	0

1	0	33.18	0.084712	2.85254	0.693147
1	0	33.18	0.088825	2.34758	1.098612
1	0	33.92	0.102154	2.08952	1.386294
1	0	33.92	0.105787	1.88634	1.609438
3	0	33.5	0.084263	1.18648	0
3	0	33.5	0.084364	0.91405	0.693147
3	0	33.42	0.055564	0.01603	1.098612
3	0	33.42	0.05646	0.07034	1.386294
3	0	33.42	0.056112	0.2635	1.609438
2	0	50.2743	0.077401	4.12656	0
2	0	50.2743	0.073475	4.3794	0.693147
2	0	50.2743	0.049202	4.21302	1.098612
2	0	32.8256	0.090824	5.13158	0
2	0	32.8256	0.148625	5.14718	0.693147
2	0	32.8256	0.079919	5.1185	1.098612
2	0	26.1486	0.078423	7.50879	0
2	0	26.1487	0.089243	7.4159	0.693147
2	0	26.9185	0.082996	6.43224	1.098612
2	0	27.2147	0.065936	5.63078	1.386294
2	0	64.22	0.050252	0.54237	1.609438
0	2	43.4825	0.111236	5.37253	0
0	2	43.4825	0.115425	6.13551	0.693147
0	2	43.4825	0.125485	5.84045	1.098612
0	2	43.4825	0.090335	5.6615	1.386294
0	2	43.4825	0.087943	5.84352	1.609438
2	0	47.29	0.074705	1.60019	0
2	0	61.9751	0.020589	8.73487	1.609438
1	0	69.6876	0.076074	3.17319	0
1	0	22.6844	0.045421	7.21454	0
1	0	56.2839	0.046418	1.18833	0
1	0	31.759	0.035451	3.86707	0
0	1	29.653	0.105805	2.62577	0
1	0	41.5251	0.078156	3.81447	0
3	0	31.78	0.060726	0.97805	0
0	1	36.72	0.051099	5.03597	0
1	2	37.92	0.072964	5.46144	0
0	1	37.103	0.074331	2.83256	0
0	2	71.0117	0.081466	5.66447	0
2	1	27.43	0.124248	4.87275	0
0	2	62.0791	0.091397	6.509	0
1	1	64.484	0.193497	2.38	0
2	0	55.8125	0.090835	0	0
0	2	52.46	0.126739	1.734	0
2	0	69.4389	0.119686	4.167	0
1	0	48.97	0.081387	2.304	0
2	0	61.256	0.097272	7	0
1	1	68.41	0.137078	0.968	0
2	0	52.5686	0.04483	4.18259	0
0	1	56.959	0.01794	9.536	0
0	1	50.1	0.132231	0	0
3	0	44.56	0.104266	1.5625	0
1	0	43.9894	0.166476	5.54501	0
1	0	43.45	0.088982	9.101	0
1	0	48.6436	0.057574	4.15	0
1	0	53.32	0.141107	0.25	0
